# Differentially Expressed Circular RNAs and Their Therapeutic Mechanism in Non-segmental Vitiligo Patients Treated With Methylprednisolone

**DOI:** 10.3389/fmed.2022.839066

**Published:** 2022-05-16

**Authors:** Jiaqi Zhang, Ning Liang, Yan Cao, Min Li

**Affiliations:** ^1^Department of Dermatology, The Affiliated Changzhou No. 2 People’s Hospital of Nanjing Medical University, Changzhou, China; ^2^Department of Dermatology, Children’s Hospital of Nanjing Medical University, Nanjing, China

**Keywords:** vitiligo, circRNA, methylprednisolone, ferroptosis, treatment

## Abstract

Vitiligo is characterized by chronic skin depigmentation arising from the autoimmune destruction of epidermal melanocytes. Systemic corticosteroid therapy is an effective immunosuppressive treatment for progressive generalized vitiligo. Circular RNAs (circRNAs) play various roles in diseases. In systemic corticosteroid therapy, however, how circRNAs function to counter vitiligo is still unclear. In this article, we identified the differentially expressed circRNAs (DEcircRNAs) in vitiligo patients before and after the administration of methylprednisolone. Total RNA was extracted from the peripheral blood of patients with vitiligo, and samples were hybridized into a circRNA array. A total of 375 (51 upregulated and 324 downregulated) circRNAs were differentially expressed. Box, scatter, volcano, and heatmap plots were generated to classify the samples. Gene Ontology (GO) and Kyoto Encyclopedia of Genes and Genomes (KEGG) enrichment analyses were performed on DEcircRNAs. These DEcircRNAs were enriched in vitiligo-related biological processes, such as ferroptosis, organic substance transport, protein metabolic process, and cellular component organization or biogenesis. Two different databases, TargetScan and miRanda, were used to predict circRNA/miRNA interactions. Several circRNA/miRNA interactions were involved in ferroptosis. These circRNAs might serve as therapeutic targets in the treatment of vitiligo.

## Introduction

As a multifactorial disease, vitiligo brings not only white skin lesions, but also depression and self-abasement (1). Oxidative stress is involved in active vitiligo. Excessive oxygen radicals cause cellular oxidation, and even cell death (2, 3). Moreover, excessive generation of reactive oxygen species (ROS) is associated with autoimmune diseases (4), as evidenced by the destruction or apoptosis of vitiliginous melanocytes in vitiligo (5). Methylprednisolone exerts a moderate efficacy by suppressing inflammation and immune responses. However, the mechanism of systemic glucocorticoids in treating vitiligo remains a conundrum.

High-throughput sequencing techniques have discovered the roles of circular RNAs (circRNAs) in various diseases. circRNAs are generated from precursor mRNAs (pre-mRNAs), with a closed-loop without 5′-cap and 3′-end polyadenylated tails (6–8). circRNAs can function as miRNAs sponges to affect the stability or translation of target RNAs (9, 10). Moreover, circRNAs can also modify gene expression (11). Aberrant expression of circRNAs has been observed in cancer, neurodegenerative disorders, and metabolic disorders (12–14). For example, circAGO2 is upregulated to promote the proliferation, invasion, and metastasis of cancer *in vitro* and *in vivo* (15).

Increasing evidence suggests that dysregulation of circRNAs is implicated in the pathogenesis of vitiligo. Li et al. found circ_0087961-miR-27a-3p-PAXILLIN played a regulatory role in the pathology of vitiligo (16). Ouyang et al. demonstrated for the first time that UVB-induced circRNA ciRS-7 triggered melanogenesis in monocytes *via* the miR-7/STAT3 and AKT/FGF2 paracrine axis in both keratinocytes and fibroblasts (17). Jiang et al. verified that a ceRNA network of ENST00000606533, circ_0091223, and TYR mRNA was linked by miR-1291, suggesting that they may be novel biomarkers for skin pigmentation disorders (18). To date, treatment options for these disorders include topical agents, such as corticosteroids, calcineurin inhibitors, and vitamin D analogs (19). Repigmentation can be promoted with narrow-band ultraviolet B light (nb-UVB) (20, 21). Systemic corticosteroids are indicated for generalized and progressive vitiligo. Wada-Irimada et al. reported that methylprednisolone pulse therapy was safe and effective for progressive generalized vitiligo (22). The results of the initial screen of the microarray and the subsequent bioinformatics analysis provide an important basis for the in-depth study of the actions of circRNAs in the development of vitiligo (Table 1). However, we knew a little about the functions of circRNAs in the treatment of vitiligo with systemic glucocorticoid therapy.

**TABLE 1:** Circular RNAs (circRNAs) expression profiles in vitiligo.

Species	Sample	Circular RNA	Function of circRNAs	References
Human	Skin tissue	hsa_circ_0007716	Upregulated in vitiligo	(16)
Human	Skin tissue	hsa_circ_0087961	Downregulated in vitiligo	(16)
Human	Keratinocytes, Fibroblasts, and Melanocytes	ciRS-7	Triggered melanogenesis in monocytes	(17)
Human	Melanocytes	circ_0091223	Novel biomarker for skin pigmentation disorders	(18)
Human	Melanocytes	hsa_circ_0048910	Oxidative stress injury of melanocytes	(43)
Human	Melanocytes	hsa_circ_0048909	Oxidative stress injury of melanocytes	(43)
Mice	Skin tissue	circBub1b	Upregulated in black mice skin	(44)
Mice	Skin tissue	circTmem26	Upregulated in black mice skin	(44)
				

In this study, we explored the mechanism of circRNAs in the pathogenesis and treatment of vitiligo. We used Arraystar Circular RNA Microarray to explore the differentially expressed circRNAs (DEcircRNAs) in vitiligo patients before and after systemic glucocorticoids. Then, GO and KEGG analyses were performed to evaluate their enriched functions. Finally, we teased out functional circRNA-miRNA pairs. Our findings can provide novel insights into the pathogenesis and treatment of vitiligo.

## Materials and Methods

### Patients and Samples

Peripheral blood specimens were obtained from the Department of Dermatology, The Affiliated Changzhou No.2 People’s Hospital of Nanjing Medical University, with written consent from all patients. All experiments were conducted in full compliance with governmental policies and guidelines. All four patients (two men and two women, aged 50–60 years) enrolled in this study were diagnosed with non-segmental vitiligo, without a history of systemic therapy or topical steroids before blood sampling. Under sterile conditions, whole blood (3 ml) was sampled by venipuncture into heparinized vacutainers from the patients before and after systemic glucocorticoid therapy (oral methylprednisolone tablets, 12 mg daily for 8 weeks). All patients showed improvement in symptoms at week 8 after glucocorticoid therapy. These patients were hence considered glucocorticoid therapy responders and evaluated by genomic and bioinformatic analysis. The experimental protocols described in this study were approved by the ethics review committee of The Affiliated Changzhou No.2 People’s Hospital of Nanjing Medical University and were conducted in compliance with the guidelines. The certificate number is [2020]KY205-1.

### RNA Isolation

Total RNA of 4 pairs of vitiligo samples was extracted using Trizol reagent (Invitrogen, Carlsbad, CA, United States) according to the manufacturer’s protocol. After the addition of chloroform, the samples were centrifuged at 12,000 rpm for 10 min. The aqueous phase was mixed with isopropanol and RNA pellets were collected by centrifugation (12,000 rpm, 15 min, 4^°^C). RNA pellets were washed with 70% ethanol and dissolved in RNase-free, DEPC (diethylpyrocarbonate)-treated water (Waltham, MA, United States).

### RNA Sample QC, Labeling, and Array Hybridization

Total RNA from each sample was quantified using the NanoDrop ND-1000, and the concentration of RNA was determined by OD260. First, total RNA was digested with Rnase R (Epicenter, Inc.) to remove linear RNA and enrich circular RNA. Then, the enriched circular RNA was amplified and transcribed into fluorescent cRNA utilizing a random priming method (Arraystar Super RNA Labeling Kit; Arraystar). The labeled cRNA was hybridized onto the Arraystar Human circRNA Array V2 (8 × 15K, Arraystar). Finally, the slides were washed and scanned by the Agilent Scanner G2505C. The sample preparation and microarray hybridization were performed based on Arraystar’s standard protocols.

### Microarray Differential Expression Analysis

Array image analysis was based on Agilent Feature Extraction software (version 11.0.1.1). The Limma R package was used to conduct differential expression analysis. circRNAs having a fold change > 1.5 and a *P*-value < 0.05 were considered significantly differentially expressed. The volcano plot and hierarchical clustering heat map showed the DEcircRNAs between the two groups. The statistical significance of the difference was estimated by a *t*-test.

### Gene Ontology and Kyoto Encyclopedia of Genes and Genomes Pathway Enrichment Analyses

The overlapped DEcircRNAs were subjected to Gene Ontology (GO) and Kyoto Encyclopedia of Genes and Genomes (KEGG) pathways analyses performed on the R software clusterProfiler package. In GO analysis, the biological processes (BPs), cellular compositions (CCs), and molecular functions (MFs) involved in circRNAs were analyzed. The pathway diagram was derived from the KEGG database. A *P*-value < 0.05 was deemed to be significant functional terms and pathways.

### Annotation With Circular RNAs-miRNA Interaction

Arraystar’s homemade miRNA target prediction software based on TargetScan (23) and miRanda (24) was used to predict circRNA/miRNA interaction. Upregulation and downregulation of circRNA and miRNA were identified.

### Statistical Analysis

The data from this study were presented as means ± standard deviation (SD). The difference between groups was assessed using a two-sided student *t*-test. *P* < 0.05 was considered statistically significant.

## Results

### Differentially Expressed CircRNAs

**FIGURE 1:**
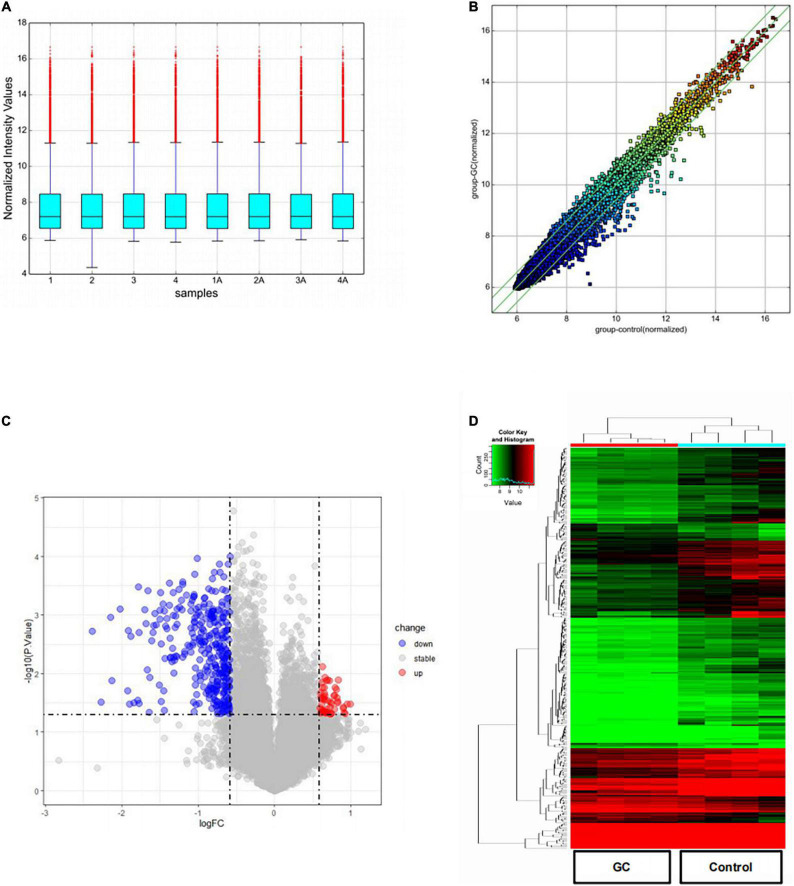
DEcircRNAs in patients with vitiligo before and after systemic glucocorticoid therapy. **(A)** A box plot shows the distribution of expression data in each sample after quantile normalization. **(B)** Scatter plot was performed to evaluate the centralized tendency of chip data in two different groups. **(C)** Volcano plot of the *P*-value as a function of weighted fold change for DEcircRNAs (DEcircRNAs) (fold change > 1.5, *P* < 0.05). **(D)** Heat map for potential DEcircRNAs (*n* = 375) shows 51 upregulated and 324 downregulated.

The box plot shows the distribution of expression data from all the samples. After normalization, the total gene expression was basically the same, indicating insignificant batch effects and system deviations (Figure 1A). The scatter plot shows circRNA expression variation between the two groups of samples (Figure 1B). The circRNAs above the top green line and below the bottom green line showed fold changes of more than 1.5.

The volcano plot visualizes the circRNA microarray results after a 2-month treatment of systemic glucocorticoids (Figure 1C). The vertical lines correspond to 1.5-fold up or down, respectively, and the horizontal line represents a *P*-value of 0.05. The red and blue points in the plot represent the up-regulated and down-regulated circRNAs with statistical significance, separately. Compared to the control group, 51 up-regulated and 324 down-regulated circRNAs were detected in the group treated with systemic glucocorticoid.

The number of down-regulated circRNAs was largely more than that of up-regulated circRNAs. Tables 2, 3 demonstrate the top 10 upregulated and downregulated circRNAs. The heatmap of hierarchical clustering analysis shows that these DEcircRNAs could distinguish samples collected before and after treatment (Figure 1D).

**TABLE 2:** Top 10 upregulated circRNAs.

CircRNA	*P*-value	FC	Regulation
hsa_circRNA_105055	0.007582547	1.5388999	Up
hsa_circRNA_103796	0.011590286	1.5802079	Up
hsa_circRNA_009550	0.012917238	1.779194	Up
hsa_circRNA_104747	0.012932469	1.6126129	Up
hsa_circRNA_103948	0.013086105	1.6376076	Up
hsa_circRNA_100982	0.013325807	1.5911831	Up
hsa_circRNA_403865	0.014330979	1.5287481	Up
hsa_circRNA_044065	0.017245821	1.5710328	Up
hsa_circRNA_100412	0.017501176	1.5438505	Up
hsa_circRNA_025984	0.017539967	1.7253039	Up

**TABLE 3:** Top 10 downregulated circRNAs.

circRNA	*P*-value	FC	Regulation
hsa_circRNA_011228	0.000100102	1.5007234	Down
hsa_circRNA_403694	0.00010878	2.0222611	Down
hsa_circRNA_101738	0.000133793	1.652627	Down
hsa_circRNA_100966	0.00013521	1.5250118	Down
hsa_circRNA_002122	0.000184899	1.644309	Down
hsa_circRNA_061482	0.000188585	1.7258389	Down
hsa_circRNA_102949	0.000201268	2.0667206	Down
hsa_circRNA_102479	0.000221586	1.5848103	Down
hsa_circRNA_102849	0.000240042	1.7620554	Down
hsa_circRNA_402533	0.000272795	2.3031702	Down

### Gene Ontology Enrichment Analysis

**FIGURE 2:**
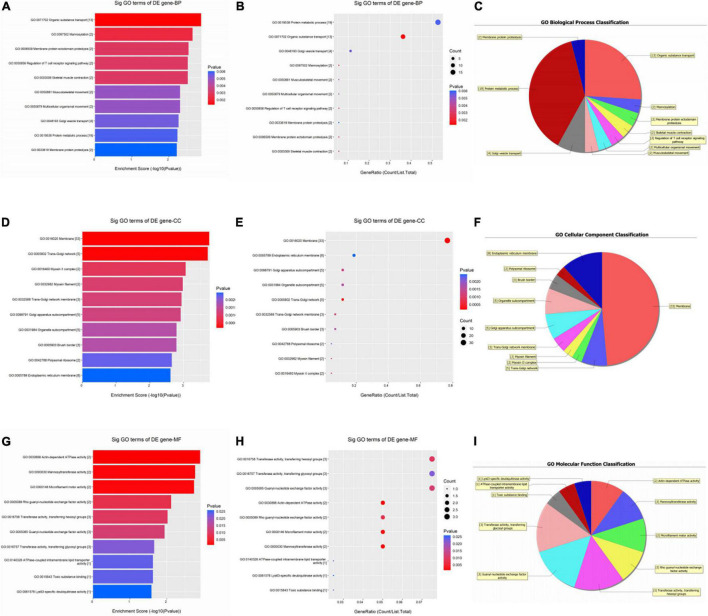
Gene ontology (GO) enrichment analysis of upregulated circRNAs. For bubble plot, the y-axis shows the enriched terms in three GO categories and the x-axis shows the Enrichment Score [−log10(*P*-value)] or GeneRatio (Count/List. Total): **(A,B)** biological process, **(D,E)** cellular component, **(G,H)** molecular function. Pie chart shows the distribution of significantly GO terms: **(C)** biological process, **(F)** cellular component, **(I)** molecular function.

As shown in Figure 2, up-regulated circRNAs were significantly enriched in skin epidermis-related biological processes, such as organic substance transport, mannosylation, membrane protein ectodomain proteolysis, protein metabolic process, and Golgi vesicle transport (Figures 2A–C). As for CCs, these DEcircRNAs were significantly enriched in membrane, trans-Golgi network, myosin II complex, endoplasmic reticulum membrane, and Golgi apparatus subcompartment (Figures 2D–F). For MFs, they were significantly enriched in actin-dependent ATPase activity, mannosyltransferase activity, microfilament motor activity, transferase activity, transferring hexosyl, and glycosyl groups (Figures 2G–I).

**FIGURE 3:**
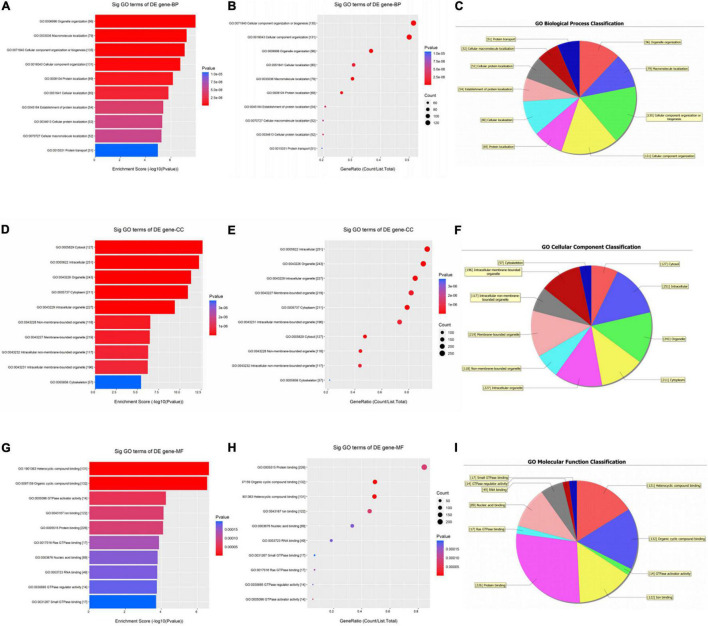
Gene ontology (GO) enrichment analysis of downregulated circRNAs. For bubble plot, the y-axis shows the enriched terms in three GO categories and the x-axis shows the Enrichment Score [−log10(*P*-value)] or GeneRatio (Count/List. Total): **(A,B)** biological process, **(D,E)** cellular component, **(G,H)** molecular function. Pie chart shows the distribution of significantly GO terms: **(C)** biological process, **(F)** cellular component, **(I)** molecular function.

As shown in Figure 3, the down-regulated circRNAs were enriched in biological processes such as organelle organization, macromolecule localization, and cellular component organization (Figures 3A–C). For CCs, these genes were significantly enriched in the cytosol and intracellular organelle (Figures 3D–F). As for MFs, they were significantly enriched in heterocyclic compound binding, organic cyclic compound binding, GTPase activator activity, and protein binding (Figures 3G–I).

### Kyoto Encyclopedia of Genes and Genomes Pathway Enrichment Analysis

**FIGURE 4:**
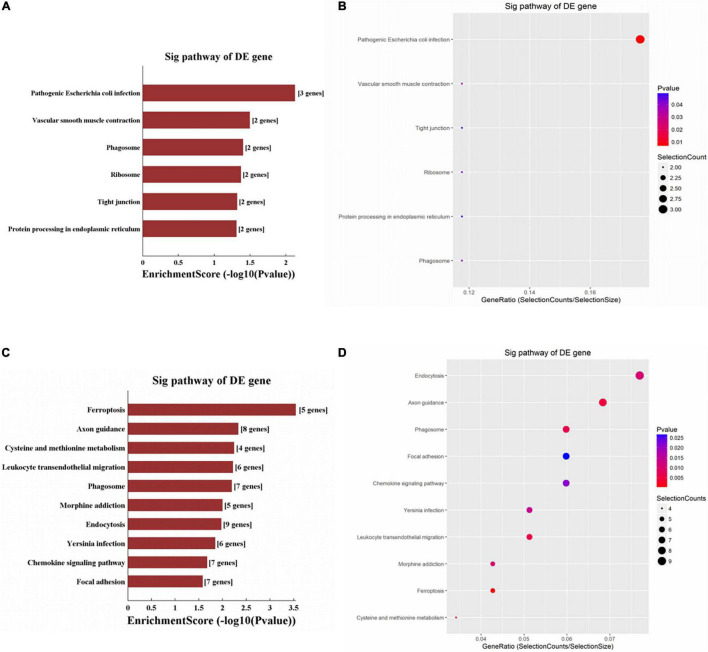
KEGG enrichment analysis of circRNAs. For bar plot and bubble plot, the y-axis shows the enriched pathways of genes and the x-axis shows the Enrichment Score [−log10(*P*-value)] or GeneRatio (Count/List. Total): **(A,B)** upregulated circRNAs, **(C,D)** downregulated circRNAs.

**FIGURE 5:**
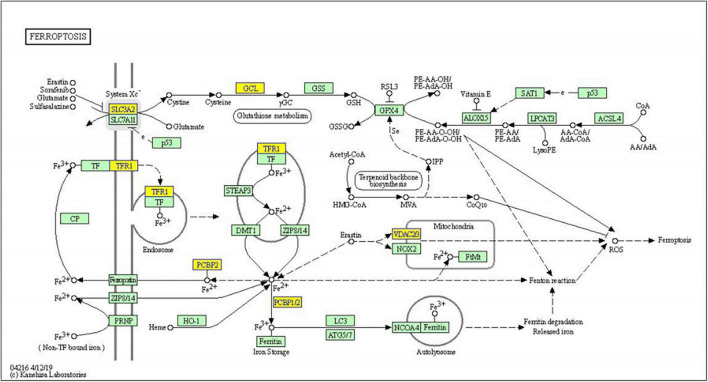
KEGG pathway diagram of ferroptosis involved in differentially expressed genes. Yellow nodes are associated with down-regulated genes, and green nodes have no significance.

Significantly enriched pathways are shown in Figure 4. The up-regulated circRNAs were significantly involved in the processes of pathogenic *Escherichia coli* infection, vascular smooth muscle contraction, phagosome, and ribosome (Figures 4A,B). The down-regulated circRNAs were significantly enriched in ferroptosis, axon guidance, cysteine and methionine metabolism, endocytosis, and phagosome (Figures 4C,D). The genes involved in the ferroptosis pathway included SLC3A2, GCLM, PCBP2, TFRC, and VDAC3 (Figure 5), respectively, corresponding to hsa_circRNA_100842, hsa_circRNA_100283, hsa_circRNA_101076, hsa_circRNA_103556, and hsa_circRNA_104600.

### Annotation With Circular RNAs/miRNA Interaction

**FIGURE 6:**
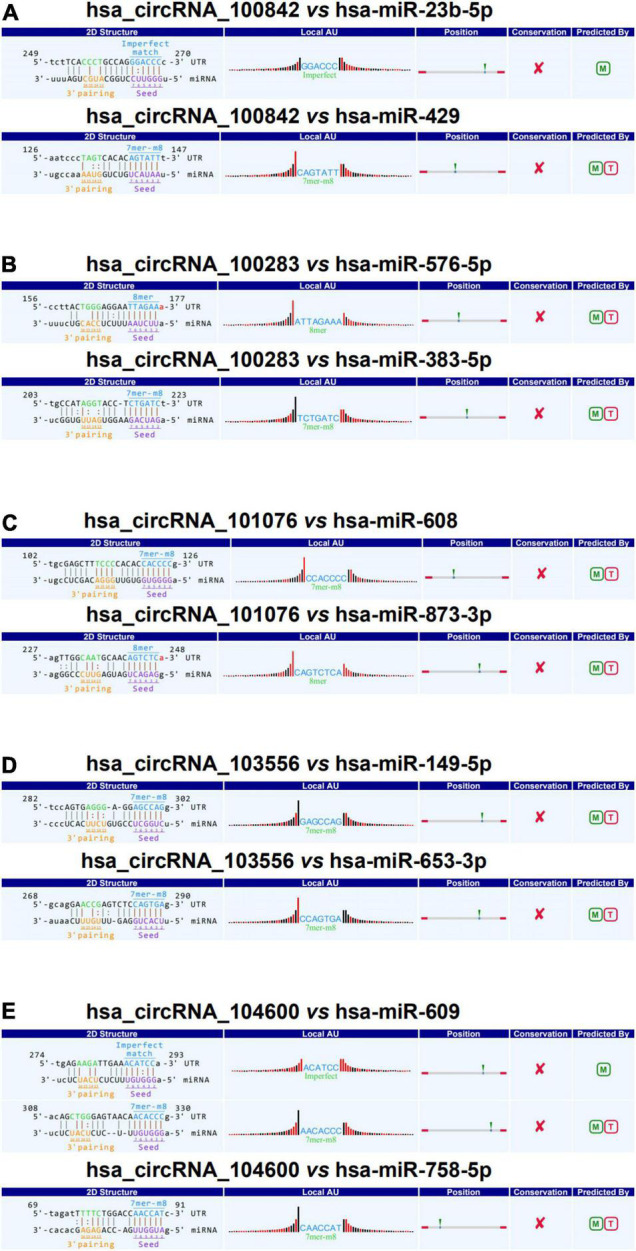
Complementary miRNAs of DEcircRNAs. **(A)** The 2D structure of the binding sites of hsa_circRNA_100842 (circSLC3A2) with hsa-miR-23b-5p and hsa-miR-429. **(B)** The 2D structure of the binding sites of hsa_circRNA_100283 (circGCLM) with hsa-miR-576-5p and hsa-miR-383-5p. **(C)** The 2D structure of the binding sites of hsa_circRNA_101076 (circPCBP2) with hsa-miR-608 and hsa-miR-873-3p. **(D)** The 2D structure of the binding sites of hsa_circRNA_103556 (circTFRC) with hsa-miR-149-5p and hsa-miR-653-3p. **(E)** The 2D structure of the binding sites of hsa_circRNA_104600 (circVDAC3) with hsa-miR-609 and hsa-miR-758-5p.

It is reported that the circRNA ciRS-7 can act as an endogenous “sponge” of miRNA-7 (25). As shown in Figure 6, the circRNA/miRNA interaction was predicted, and the DEcircRNAs involved in ferroptosis were annotated in detail with circRNA/miRNA interaction. The circRNAs and corresponding miRNAs involved in ferroptosis pathway were demonstrated, including hsa-miR-23b-5p and hsa-miR-429 containing conserved binding sites with mmu_hsa_circRNA_100842 (circSLC3A2); hsa-miR-576-5p and hsa-miR-383-5p containing conserved binding sites with mmu_hsa_circRNA_100283 (circGCLM); hsa-miR-608 and hsa-miR-873-3p containing conserved binding sites with mmu_hsa_circRNA_101076 (circPCBP2); hsa-miR-149-5p and hsa-miR-653-3p containing conserved binding sites with mmu_hsa_circRNA_103556 (circTFRC); hsa-miR-609 and hsa-miR-758-5p containing conserved binding sites with mmu_hsa_circRNA_104600 (circVDAC3).

## Discussion

In this study, we explored the function of DEcircRNAs in patients with vitiligo treated with systemic glucocorticoid therapy. A total of 51 up-regulated and 324 down-regulated circRNAs were detected in the group of patients with vitiligo treated with systemic glucocorticoids. These DEcircRNAs provide clues to design new targeted therapies for vitiligo.

According to KEGG enrichment analysis, the down-regulated DEcircRNAs were significantly enriched in ferroptosis. Ferroptosis regulates cell death in an iron and lipid peroxidation−dependent manner (26, 27). The ferroptosis is repressed after systemic glucocorticoid therapy in patients with vitiligo. It is reported that ferroptosis is implicated in IFN−γ−associated apoptosis, and that IFN−γ can lead to melanocyte death in the case of vitiligo (28–30). Increased ROS and lip-ROS caused by oxidative stress play a vital role in the process of ferroptosis (26, 31, 32). Previous studies have provided that circRNAs can regulate ferroptosis. circKIF4A can suppress ferroptosis by sponging miR-1231 and upregulating GPX4 in the malignant progression of papillary thyroid cancer (33). GPX4 has been known as a biomarker of ferroptosis (34, 35). circEPSTI1 modulates the proliferation of cervical cancer *via* the miR-375/409-3P/515-5p-SLC7A11 axis relative to ferroptosis (36).

After systemic glucocorticoid therapy, we detected more down-regulated circRNAs in patients with vitiligo. They were also significantly enriched in the pathway of ferroptosis. Moreover, we elucidated that several miRNAs interacted with the DEcircRNAs involved in the ferroptosis pathway. circRNAs interact with miRNAs to regulate disease development (37). ciRS-7 functions in neurological disorders and tumor. miR-7 directly targeting a-synuclein protein in Parkinson’s disease and ubiquitin protein ligaseA (UBE2A) in Alzheimer’s disease (38).

As we all know, circRNAs play critical roles in autoimmune diseases. circPTPN22, synthesized from the protein tyrosine phosphatase non-receptor type 22 (PTPN22) gene, shows an expression negatively correlated with the activity of systemic lupus erythematosus (39). Lodde et al. have described several circRNAs that function mainly as biomarkers for autoimmune diseases, including ciRS-7 for rheumatoid arthritis (40) and the circ_0000479 for systemic lupus erythematosus (41, 42). However, we found that circSLC3A2 and circTFRC were down-regulated after treatment with systemic glucocorticoid therapy. Furthermore, downregulation of circGCLM, circPCBP2, and circVDAC3, three circRNAs involved in the ferroptosis, was first reported in the treatment of vitiligo.

Our findings demonstrated the DEcircRNAs in the treatment of vitiligo with systemic glucocorticoid therapy for the first time. Additional experiments will be required to address the significance of these findings, the binding characteristics of the identified circRNAs, and their functional activity after exposure to glucocorticoid. Moreover, it would be interesting to compare differential circRNA expression in cases where remission is induced by a different therapy, to control for confounding effects independent from glucocorticoid. A limitation of this study mainly lies in that most of the patients with vitiligo in this study were between 50 and 60 years old, while no younger patients were included. We will explore the circRNA data of younger patients and compare the difference in expression profile among patients of different age groups in a further study. Moreover, the data were obtained from a circRNA microarray; although enrichment analyses provided several related pathways, there is still a lack of verification with wet-lab biochemical experiments. In addition, molecular biology experiments should be carried out to verify our results.

## Data Availability Statement

The data presented in the study are deposited in the Gene Expression Omnibus repository, accession number GSE197415.

## Ethics Statement

The studies involving human participants were reviewed and approved by Institutional Review Board (IRB) of The Affiliated Changzhou No. 2 People’s Hospital of Nanjing Medical University. The patients/participants provided their written informed consent to participate in this study.

## Author Contributions

JZ and YC conceived the main idea and analyzed the data. JZ drafted the manuscript. YC reviewed drafts of the article and improved the manuscript. NL collected the data and performed the statistical analysis. ML provided statistical advice. YC and ML supervised the study and provided funding. All authors read and commented on the manuscript.

## Conflict of Interest

The authors declare that the research was conducted in the absence of any commercial or financial relationships that could be construed as a potential conflict of interest.

## Publisher’s Note

All claims expressed in this article are solely those of the authors and do not necessarily represent those of their affiliated organizations, or those of the publisher, the editors and the reviewers. Any product that may be evaluated in this article, or claim that may be made by its manufacturer, is not guaranteed or endorsed by the publisher.
