# Supplementary material for: Differentially Expressed Circular RNAs and Their Therapeutic Mechanism in Non-segmental Vitiligo Patients Treated With Methylprednisolone
Source: Front Med (Lausanne). 2022 May 16;9:839066. doi: 10.3389/fmed.2022.839066 (PMC9149005; doi:10.3389/fmed.2022.839066)
Supplement: Supplementary file 1 [file Data_Sheet_1.ZIP › Additional files/GO Analysis Report/GO_GC_vs_control_down/BP_result(Human).html]

| GO.ID | Term | Ontology | Count | Pop.Hits | List.Total | Pop.Total | Fold.Enrichment | Pvalue | FDR | Enrichment.Score | Gene.Ratio | GENES |
| --- | --- | --- | --- | --- | --- | --- | --- | --- | --- | --- | --- | --- |
| GO:0006996 | organelle organization | Biological process | 96 | 4048 | 262 | 18866 | 1.70769091512537 | 1.10689133232186e-08 | 7.81354591486001e-05 | 7.95589501334846 | 0.366412213740458 | RPS27L//RPS14//GABARAP//AMBRA1//MYH9//TUBA1B//TTLL11//TBCEL//GAPDH//CFAP44//PARD3//DNM2//TERF2IP//NUP155//CCSER2//RFX2//TMF1//GATAD1//RBL1//NASP//MORC2//SCMH1//TADA2A//CRAT//PITRM1//PITPNB//SIRT5//CHCHD10//LARP4//ZMYM4//CORO1C//MYO19//TUBGCP2//MIA3//HUWE1//PLEKHM2//GOLPH3L//RAB2A//ABCD4//HOOK3//CLN6//PPT1//DYNC1H1//DDX11//PDS5B//NEK9//KPNB1//SDCCAG8//GCLM//TFRC//GSK3B//TRRAP//PRDM5//EYA3//CTCF//DOCK2//FLNB//PIP5K1C//NF1//OPHN1//BCR//MOB2//STARD13//WDR1//HECW2//HABP4//ICAM1//DYRK1A//SIRT6//SMG1//NAT10//NDUFB2//DDX6//LSM14A//TTC19//SETD2//SRPK2//ANKRD27//DYNLRB1//PDE3A//RABL2B//ACTB//SPI1//KRT17//TRAPPC4//STARD9//SH3PXD2B//GNAI1//TTC39C//CEP83//ARL6IP1//RNF4//ATP6V0B//VDAC3//PDE4DIP//UBL5// |
| GO:0033036 | macromolecule localization | Biological process | 79 | 3173 | 262 | 18866 | 1.79281533357552 | 5.5964306468236e-08 | 0.000189382079750682 | 7.25208887381869 | 0.301526717557252 | NFASC//CDC40//NUP155//SMG1//ALKBH5//FIP1L1//GABARAP//KPNB1//RANBP17//KPNA5//PARD3//ARL6IP1//RPL13A//RPL18A//RPL37A//RPS14//CRAT//PITRM1//ATP8A1//RABL2B//AP3S1//TBC1D1//RPH3AL//FAF1//MIA3//CANX//THBS1//SETD2//FTO//AGAP1//SLC15A4//GDI2//MYH9//NASP//PPT1//RAB2A//ANKRD27//HOOK3//PKDCC//ABCD4//OSBPL10//PITPNB//LDLR//NRIP1//PLEKHM2//DNM2//ATP6V0B//TFRC//MDM2//DYNLRB1//RAB11FIP5//KLHL21//NF1//ZDHHC17//GOLPH3L//ACTB//CORO1C//MCC//RANGAP1//GSK3B//GAPDH//IGF2BP3//CHCHD10//KLF7//BCR//TERF2IP//CTCF//CEP83//HK1//SH3PXD2B//OPHN1//GPR137B//TMEM59//STAC//HUWE1//UBL5//GNAI1//NEDD4L//TMF1// |
| GO:0071840 | cellular component organization or biogenesis | Biological process | 135 | 6717 | 262 | 18866 | 1.44722800868708 | 8.04853717597458e-08 | 0.000189382079750682 | 7.09428304561119 | 0.515267175572519 | RPS27L//RPS14//GABARAP//AMBRA1//NAT10//MYH9//TUBA1B//TTLL11//TBCEL//GAPDH//CFAP44//PARD3//SRPK2//DNM2//RCL1//NOL10//TERF2IP//NUP155//PAPPA2//DDR1//CCSER2//RFX2//TMF1//DOCK2//PLCG2//KPNB1//GATAD1//RBL1//NASP//MORC2//SCMH1//TADA2A//DDX17//TEX10//CRAT//PITRM1//DOCK1//OPHN1//PITPNB//SIRT5//CHCHD10//LARP4//ZMYM4//CORO1C//MYO19//TUBGCP2//MIA3//HUWE1//PLEKHM2//GOLPH3L//RAB2A//ABCD4//HOOK3//CLN6//PPT1//DYNC1H1//DDX11//PDS5B//NEK9//SDCCAG8//ACTB//KLF7//NFASC//SLIT3//TRIO//GCLM//TFRC//GSK3B//MOB2//ENC1//MDM2//PTPRG//TRAPPC4//STRN//TRRAP//PRDM5//EYA3//CTCF//SH3PXD2B//FLNB//PIP5K1C//NF1//BCR//STARD13//WDR1//HECW2//COL6A2//ICAM1//NID1//THBS1//ADAM19//KRT17//CRLF3//BMPR2//HABP4//MTR//DYRK1A//FRYL//FAF1//SIRT6//SMG1//NDUFB2//FBLIM1//DDX6//LSM14A//TTC19//PDE4DIP//SETD2//ANKRD27//DYNLRB1//PDE3A//RABL2B//SPI1//PI4KA//ATP8A1//NEDD4L//TAOK3//TIAM2//CAPRIN1//STARD9//TMEM120B//GNAI1//TTC39C//CEP83//TBC1D1//LDLR//LPXN//BRF1//ARL6IP1//RNF4//ATP6V0B//RPL13A//NFATC2//VDAC3//UBL5// |
| GO:0016043 | cellular component organization | Biological process | 131 | 6526 | 262 | 18866 | 1.44544897333742 | 1.7623349561906e-07 | 0.000311008061393736 | 6.75391154439155 | 0.5 | RPS27L//RPS14//GABARAP//AMBRA1//MYH9//TUBA1B//TTLL11//TBCEL//GAPDH//CFAP44//PARD3//SRPK2//DNM2//TERF2IP//NUP155//PAPPA2//DDR1//CCSER2//RFX2//TMF1//DOCK2//PLCG2//KPNB1//GATAD1//RBL1//NASP//MORC2//SCMH1//TADA2A//CRAT//PITRM1//DOCK1//OPHN1//PITPNB//SIRT5//CHCHD10//LARP4//ZMYM4//CORO1C//MYO19//TUBGCP2//MIA3//HUWE1//PLEKHM2//GOLPH3L//RAB2A//ABCD4//HOOK3//CLN6//PPT1//DYNC1H1//DDX11//PDS5B//NEK9//SDCCAG8//ACTB//KLF7//NFASC//SLIT3//TRIO//GCLM//TFRC//GSK3B//MOB2//ENC1//MDM2//PTPRG//TRAPPC4//STRN//TRRAP//PRDM5//EYA3//CTCF//SH3PXD2B//FLNB//PIP5K1C//NF1//BCR//STARD13//WDR1//HECW2//COL6A2//ICAM1//NID1//THBS1//ADAM19//KRT17//CRLF3//BMPR2//HABP4//MTR//DYRK1A//FRYL//FAF1//SIRT6//SMG1//NAT10//NDUFB2//FBLIM1//DDX6//LSM14A//TTC19//PDE4DIP//SETD2//ANKRD27//DYNLRB1//PDE3A//RABL2B//SPI1//PI4KA//ATP8A1//NEDD4L//TAOK3//TIAM2//CAPRIN1//STARD9//TMEM120B//GNAI1//TTC39C//CEP83//TBC1D1//LDLR//LPXN//BRF1//ARL6IP1//RNF4//ATP6V0B//RPL13A//NFATC2//VDAC3//UBL5// |
| GO:0008104 | protein localization | Biological process | 69 | 2788 | 262 | 18866 | 1.78211144819127 | 6.91481430902308e-07 | 0.000976233484147878 | 6.16021947797686 | 0.263358778625954 | NFASC//SMG1//CDC40//ALKBH5//FIP1L1//NUP155//GABARAP//KPNB1//RANBP17//KPNA5//PARD3//ARL6IP1//RPL13A//RPL18A//RPL37A//RPS14//CRAT//PITRM1//RABL2B//AP3S1//TBC1D1//RPH3AL//FAF1//MIA3//CANX//SETD2//AGAP1//SLC15A4//GDI2//MYH9//NASP//PPT1//RAB2A//ANKRD27//HOOK3//PKDCC//PLEKHM2//DNM2//ATP6V0B//TFRC//MDM2//DYNLRB1//RAB11FIP5//KLHL21//NF1//ZDHHC17//GOLPH3L//ACTB//CORO1C//MCC//RANGAP1//GSK3B//GAPDH//CHCHD10//KLF7//BCR//TERF2IP//CTCF//CEP83//HK1//SH3PXD2B//OPHN1//GPR137B//TMEM59//STAC//HUWE1//UBL5//GNAI1//NEDD4L// |
| GO:0051641 | cellular localization | Biological process | 80 | 3493 | 262 | 18866 | 1.64918714200484 | 1.53483950658525e-06 | 0.00180573867949755 | 5.81393703064185 | 0.305343511450382 | DOCK2//NFASC//HABP4//THBS1//WDR1//CDC40//NUP155//SMG1//ALKBH5//FIP1L1//GABARAP//KPNB1//RANBP17//KPNA5//PARD3//ARL6IP1//RPL13A//RPL18A//RPL37A//RPS14//CRAT//PITRM1//RABL2B//AP3S1//TBC1D1//RPH3AL//DYNC1H1//MIA3//TRAPPC4//PPT1//HOOK3//SETD2//PDE4D//ABCD4//PIP5K1C//DNM2//CORO1C//MYO19//TUBA1B//SLC30A7//PLEKHM2//MYH9//SPAG9//MDM2//ANKRD27//DYNLRB1//RAB11FIP5//KLHL21//NF1//PKDCC//GOLPH3L//HUWE1//ATP8A1//SLC15A4//GDI2//BCR//RANGAP1//GSK3B//CEP83//OPHN1//ACTB//CANX//NEMF//BACH2//PLCG2//CHCHD10//KLF7//TERF2IP//CTCF//HK1//SH3PXD2B//LDLR//SDCCAG8//GPR137B//TFRC//TMEM59//STAC//UBL5//GNAI1//NEDD4L// |
| GO:0045184 | establishment of protein localization | Biological process | 54 | 2080 | 262 | 18866 | 1.86942894891368 | 3.9961329033407e-06 | 0.00402981459495457 | 5.39836007644514 | 0.206106870229008 | SMG1//CDC40//ALKBH5//FIP1L1//NUP155//GABARAP//KPNB1//RANBP17//KPNA5//PARD3//ARL6IP1//RPL13A//RPL18A//RPL37A//RPS14//CRAT//PITRM1//RABL2B//AP3S1//TBC1D1//RPH3AL//MIA3//CANX//SETD2//AGAP1//SLC15A4//GDI2//MYH9//NASP//PPT1//RAB2A//ANKRD27//HOOK3//PKDCC//DNM2//ATP6V0B//TFRC//DYNLRB1//RAB11FIP5//NF1//ZDHHC17//GOLPH3L//RANGAP1//GSK3B//MDM2//GAPDH//KLF7//BCR//HK1//OPHN1//HUWE1//UBL5//CORO1C//MCC// |
| GO:0034613 | cellular protein localization | Biological process | 52 | 1984 | 262 | 18866 | 1.88729684806698 | 4.79979429485713e-06 | 0.00423521849092456 | 5.31877737481666 | 0.198473282442748 | NFASC//SMG1//CDC40//ALKBH5//FIP1L1//NUP155//GABARAP//KPNB1//RANBP17//KPNA5//PARD3//ARL6IP1//RPL13A//RPL18A//RPL37A//RPS14//CRAT//PITRM1//RABL2B//AP3S1//TBC1D1//RPH3AL//SETD2//MDM2//DYNLRB1//KLHL21//NF1//PKDCC//GOLPH3L//RANGAP1//GSK3B//CHCHD10//BCR//TERF2IP//MIA3//CTCF//CEP83//HOOK3//HK1//SH3PXD2B//DNM2//OPHN1//GPR137B//TFRC//ACTB//TMEM59//STAC//HUWE1//UBL5//GNAI1//RAB11FIP5//NEDD4L// |
| GO:0070727 | cellular macromolecule localization | Biological process | 52 | 1993 | 262 | 18866 | 1.87877418292267 | 5.46552163226379e-06 | 0.0042867908002389 | 5.26236838244204 | 0.198473282442748 | NFASC//SMG1//CDC40//ALKBH5//FIP1L1//NUP155//GABARAP//KPNB1//RANBP17//KPNA5//PARD3//ARL6IP1//RPL13A//RPL18A//RPL37A//RPS14//CRAT//PITRM1//RABL2B//AP3S1//TBC1D1//RPH3AL//SETD2//MDM2//DYNLRB1//KLHL21//NF1//PKDCC//GOLPH3L//RANGAP1//GSK3B//CHCHD10//BCR//TERF2IP//MIA3//CTCF//CEP83//HOOK3//HK1//SH3PXD2B//DNM2//OPHN1//GPR137B//TFRC//ACTB//TMEM59//STAC//HUWE1//UBL5//GNAI1//RAB11FIP5//NEDD4L// |
| GO:0015031 | protein transport | Biological process | 51 | 1985 | 262 | 18866 | 1.85007018286 | 1.05500116915163e-05 | 0.00744725325304136 | 4.97674705908115 | 0.194656488549618 | SMG1//CDC40//ALKBH5//FIP1L1//NUP155//GABARAP//KPNB1//RANBP17//KPNA5//PARD3//ARL6IP1//RPL13A//RPL18A//RPL37A//RPS14//CRAT//PITRM1//RABL2B//AP3S1//TBC1D1//RPH3AL//MIA3//CANX//SETD2//DNM2//ATP6V0B//TFRC//DYNLRB1//RAB11FIP5//NF1//ZDHHC17//PKDCC//GOLPH3L//RANGAP1//GSK3B//MDM2//GAPDH//KLF7//BCR//OPHN1//HUWE1//UBL5//AGAP1//SLC15A4//GDI2//MYH9//NASP//PPT1//RAB2A//ANKRD27//HOOK3// |
| GO:0015833 | peptide transport | Biological process | 51 | 2026 | 262 | 18866 | 1.81263046050202 | 1.84587550098741e-05 | 0.0118454865104274 | 4.73379759424052 | 0.194656488549618 | SMG1//CDC40//ALKBH5//FIP1L1//NUP155//GABARAP//KPNB1//RANBP17//KPNA5//PARD3//ARL6IP1//RPL13A//RPL18A//RPL37A//RPS14//CRAT//PITRM1//SLC15A4//RABL2B//AP3S1//TBC1D1//RPH3AL//MIA3//CANX//SETD2//AGAP1//GDI2//MYH9//NASP//PPT1//RAB2A//ANKRD27//HOOK3//PKDCC//DNM2//ATP6V0B//TFRC//DYNLRB1//RAB11FIP5//NF1//ZDHHC17//GOLPH3L//RANGAP1//GSK3B//MDM2//GAPDH//KLF7//BCR//OPHN1//HUWE1//UBL5// |
| GO:0044265 | cellular macromolecule catabolic process | Biological process | 35 | 1203 | 262 | 18866 | 2.09498518335205 | 2.69499581166688e-05 | 0.0147420143539421 | 4.56944190542024 | 0.133587786259542 | SMG1//RPL13A//RPL18A//RPL37A//RPS14//CNOT11//CNOT8//KPNB1//HUWE1//NEDD4L//MDM2//RNF111//HECW2//USP42//UBE3B//PSMB5//ENC1//GSK3B//FAF1//PCBP2//RNF216//RNF4//IGF2BP3//ALKBH5//PUM1//DDX6//PPT1//TAF15//TMF1//FTO//SIRT6//OPHN1//LDLR//NEMF//CLN6// |
| GO:0071702 | organic substance transport | Biological process | 64 | 2800 | 262 | 18866 | 1.64588876772083 | 2.73879488226186e-05 | 0.0147420143539421 | 4.56244049233076 | 0.244274809160305 | ARL6IP1//CDC40//NUP155//SMG1//ALKBH5//FIP1L1//GABARAP//KPNB1//RANBP17//KPNA5//PARD3//RPL13A//RPL18A//RPL37A//RPS14//CRAT//PITRM1//SLC15A4//SLC3A2//ATP8A1//RABL2B//AP3S1//TBC1D1//RPH3AL//MIA3//CANX//THBS1//SETD2//AGAP1//GDI2//MYH9//NASP//PPT1//RAB2A//ANKRD27//HOOK3//PKDCC//SLC23A2//VDAC3//ABCD4//OSBPL10//PITPNB//LDLR//DNM2//ATP6V0B//TFRC//DYNLRB1//RAB11FIP5//NF1//ZDHHC17//GOLPH3L//SIRT6//RANGAP1//GSK3B//MDM2//GAPDH//IGF2BP3//ACTB//KLF7//BCR//OPHN1//HUWE1//UBL5//TMF1// |
| GO:0042886 | amide transport | Biological process | 51 | 2061 | 262 | 18866 | 1.78184828383168 | 2.92375975287137e-05 | 0.0147420143539421 | 4.5340583164915 | 0.194656488549618 | SMG1//CDC40//ALKBH5//FIP1L1//NUP155//GABARAP//KPNB1//RANBP17//KPNA5//PARD3//ARL6IP1//RPL13A//RPL18A//RPL37A//RPS14//CRAT//PITRM1//SLC15A4//RABL2B//AP3S1//TBC1D1//RPH3AL//MIA3//CANX//SETD2//AGAP1//GDI2//MYH9//NASP//PPT1//RAB2A//ANKRD27//HOOK3//PKDCC//DNM2//ATP6V0B//TFRC//DYNLRB1//RAB11FIP5//NF1//ZDHHC17//GOLPH3L//RANGAP1//GSK3B//MDM2//GAPDH//KLF7//BCR//OPHN1//HUWE1//UBL5// |
| GO:0071705 | nitrogen compound transport | Biological process | 56 | 2370 | 262 | 18866 | 1.70144619447934 | 4.04728325307037e-05 | 0.0190465149889492 | 4.39283640002141 | 0.213740458015267 | ARL6IP1//CDC40//NUP155//SMG1//ALKBH5//FIP1L1//GABARAP//KPNB1//RANBP17//KPNA5//PARD3//RPL13A//RPL18A//RPL37A//RPS14//CRAT//PITRM1//SLC15A4//SLC3A2//RABL2B//AP3S1//TBC1D1//RPH3AL//MIA3//CANX//SETD2//AGAP1//GDI2//MYH9//NASP//PPT1//RAB2A//ANKRD27//HOOK3//PKDCC//SLC23A2//VDAC3//DNM2//ATP6V0B//TFRC//DYNLRB1//RAB11FIP5//NF1//ZDHHC17//GOLPH3L//RANGAP1//GSK3B//MDM2//GAPDH//IGF2BP3//ACTB//KLF7//BCR//OPHN1//HUWE1//UBL5// |
| GO:0051649 | establishment of localization in cell | Biological process | 63 | 2795 | 262 | 18866 | 1.62307009518087 | 4.94463216702364e-05 | 0.0218150990418874 | 4.30586601018886 | 0.240458015267176 | HABP4//THBS1//WDR1//CDC40//NUP155//SMG1//ALKBH5//FIP1L1//GABARAP//KPNB1//RANBP17//KPNA5//PARD3//ARL6IP1//RPL13A//RPL18A//RPL37A//RPS14//CRAT//PITRM1//RABL2B//AP3S1//TBC1D1//RPH3AL//DYNC1H1//MIA3//TRAPPC4//PPT1//HOOK3//SETD2//PDE4D//ABCD4//PIP5K1C//DNM2//CORO1C//MYO19//TUBA1B//ANKRD27//DYNLRB1//RAB11FIP5//SPAG9//NF1//HUWE1//ATP8A1//SLC15A4//DOCK2//NFASC//GDI2//BCR//RANGAP1//GSK3B//MDM2//OPHN1//ACTB//CANX//NEMF//BACH2//PLCG2//MYH9//CEP83//KLF7//LDLR//UBL5// |
| GO:0043547 | positive regulation of GTPase activity | Biological process | 17 | 406 | 262 | 18866 | 3.01509795810928 | 5.69505267462999e-05 | 0.0236478687236548 | 4.24450225469369 | 0.0648854961832061 | TBC1D1//CORO1C//TIAM2//RALGAPA2//RANGAP1//BCR//AGAP1//DOCK1//DOCK2//GDI2//GSK3B//ICAM1//MYO9B//NF1//OPHN1//ANKRD27//STARD13// |
| GO:0006417 | regulation of translation | Biological process | 18 | 450 | 262 | 18866 | 2.88030534351145 | 6.2204823104205e-05 | 0.0243946581273657 | 4.20617594060927 | 0.0687022900763359 | IGF2BP3//RPL13A//GAPDH//CAPRIN1//ENC1//CNOT8//LARP4//KRT17//RPS27L//NAT10//THBS1//HABP4//FTO//PUM1//NEMF//LSM14A//CNOT11//RPS14// |
| GO:0051128 | regulation of cellular component organization | Biological process | 56 | 2429 | 262 | 18866 | 1.66011835360891 | 7.98126085093479e-05 | 0.0296524843930256 | 4.09792849494641 | 0.213740458015267 | PAPPA2//DDR1//PLCG2//DNM2//HUWE1//TFRC//GSK3B//MOB2//ENC1//MDM2//PTPRG//HECW2//OPHN1//KRT17//CRLF3//PPT1//SLIT3//BMPR2//WDR1//ICAM1//CTCF//DYRK1A//FAF1//SIRT6//SMG1//TERF2IP//NAT10//FBLIM1//TADA2A//ANKRD27//PDE3A//NEDD4L//DDX11//TIAM2//CAPRIN1//SH3PXD2B//CORO1C//THBS1//DYNC1H1//GNAI1//ATP8A1//MYO19//RNF4//SPI1//MORC2//CHCHD10//RPL13A//NFATC2//SDCCAG8//VDAC3//PDE4DIP//ARL6IP1//PLEKHM2//UBL5//MYH9//LDLR// |
| GO:0033365 | protein localization to organelle | Biological process | 29 | 982 | 262 | 18866 | 2.12649834424216 | 0.00010753363484205 | 0.0379539964175015 | 3.96845567396627 | 0.110687022900763 | KPNB1//RANBP17//NUP155//KPNA5//RPL13A//RPL18A//RPL37A//RPS14//CRAT//PITRM1//MDM2//KLHL21//NF1//RANGAP1//CHCHD10//TERF2IP//MIA3//CTCF//CEP83//HOOK3//HK1//DNM2//OPHN1//GPR137B//GSK3B//TFRC//HUWE1//UBL5//SETD2// |
| GO:0007010 | cytoskeleton organization | Biological process | 37 | 1399 | 262 | 18866 | 1.90441918709656 | 0.000115676875466055 | 0.0388839554245182 | 3.93675345056629 | 0.141221374045802 | MYH9//TUBA1B//GABARAP//TTLL11//TBCEL//GAPDH//CFAP44//PARD3//CCSER2//CORO1C//MYO19//TUBGCP2//DYNC1H1//SDCCAG8//DOCK2//FLNB//PIP5K1C//NF1//OPHN1//BCR//MOB2//STARD13//WDR1//ICAM1//KPNB1//DYRK1A//HOOK3//KRT17//STARD9//SH3PXD2B//GNAI1//GSK3B//RNF4//ACTB//SETD2//LARP4//ZMYM4// |
| GO:0006913 | nucleocytoplasmic transport | Biological process | 15 | 354 | 262 | 18866 | 3.05117091473671 | 0.00013654018205512 | 0.0422720031456739 | 3.86473952241974 | 0.0572519083969466 | CDC40//NUP155//SMG1//ALKBH5//FIP1L1//KPNB1//RANBP17//KPNA5//SETD2//NF1//RANGAP1//GSK3B//MDM2//NEMF//BACH2// |
| GO:0051168 | nuclear export | Biological process | 11 | 204 | 262 | 18866 | 3.88276455620416 | 0.000137732833595481 | 0.0422720031456739 | 3.8609625176256 | 0.0419847328244275 | CDC40//NUP155//SMG1//ALKBH5//FIP1L1//RANBP17//SETD2//RANGAP1//GSK3B//MDM2//NEMF// |
| GO:0043087 | regulation of GTPase activity | Biological process | 18 | 481 | 262 | 18866 | 2.6946723587945 | 0.000143888570139539 | 0.0422791521915665 | 3.84197370309243 | 0.0687022900763359 | AGAP1//DOCK1//DOCK2//GDI2//GSK3B//ICAM1//MYO9B//NF1//OPHN1//ANKRD27//STARD13//TBC1D1//CORO1C//TIAM2//RALGAPA2//RANGAP1//BCR//GPR137B// |
| GO:0051169 | nuclear transport | Biological process | 15 | 357 | 262 | 18866 | 3.02553082301623 | 0.000149734920638782 | 0.0422791521915665 | 3.82467690324994 | 0.0572519083969466 | CDC40//NUP155//SMG1//ALKBH5//FIP1L1//KPNB1//RANBP17//KPNA5//SETD2//NF1//RANGAP1//GSK3B//MDM2//NEMF//BACH2// |
| GO:0044248 | cellular catabolic process | Biological process | 53 | 2316 | 262 | 18866 | 1.64784308296747 | 0.000157024867636673 | 0.0426322515633567 | 3.8040315640101 | 0.202290076335878 | GABARAP//AMBRA1//SMG1//RPL13A//RPL18A//RPL37A//RPS14//CNOT11//CNOT8//PHKG1//PDE4D//KPNB1//HUWE1//NEDD4L//MDM2//RNF111//HECW2//USP42//UBE3B//ABCD4//IFI16//TRAPPC4//GPR137B//TMEM59//BCKDHA//PLCG2//PSMB5//ENC1//GSK3B//LDLR//SESN1//GAPDH//ATP6V0B//PCCB//PPT1//CRAT//CYP24A1//HBG2//FAF1//PCBP2//RNF216//RNF4//IGF2BP3//ALKBH5//PUM1//DDX6//CLN6//TAF15//TMF1//FTO//SIRT6//OPHN1//NEMF// |
| GO:0048523 | negative regulation of cellular process | Biological process | 98 | 5131 | 262 | 18866 | 1.37531633046249 | 0.000183002581204515 | 0.0456375135127311 | 3.73754278461399 | 0.374045801526718 | ATRIP//CTCF//TCFL5//PRDM5//NEDD4L//IFI16//RBPJ//MDM2//NFATC2//SIRT6//OVOL2//RBL1//BACH2//RPS14//SPI1//NR2C2//NRIP1//KLF11//KLF7//GTF2IRD1//NCOR2//CORO1C//TERF2IP//NF1//THBS1//DNM2//BCR//BMPR2//TAOK3//PLAGL1//CNOT11//CNOT8//MIA3//LPXN//PDS5B//AMBRA1//SLIT3//STRN//DDR1//TRIM24//SMG1//MCC//PTPRG//HUWE1//TFRC//SIRT5//PARD3//PDE4D//GAPDH//TMEM59//PSMB5//IGF2BP3//RPL13A//CAPRIN1//ENC1//CRLF3//PPT1//DYRK1A//GSK3B//RPS27L//NAT10//EFEMP1//RNF4//ANKRD27//PKDCC//ARL6IP1//PDE3A//TMF1//FANK1//PLCG2//GCLM//TRIO//MEIS1//DDX6//GPR137B//LATS2//RPH3AL//SCMH1//GNAI2//RANGAP1//TAF15//HOOK3//GNAI1//PHLPP2//FTO//PUM1//LDLR//RAB11FIP5//STARD13//MORC2//OPHN1//ICAM1//CCAR1//MYH9//SESN1//ITFG2//HECW2//EYA3// |
| GO:0009057 | macromolecule catabolic process | Biological process | 37 | 1433 | 262 | 18866 | 1.85923408426245 | 0.000187073608513208 | 0.0456375135127311 | 3.72798747645256 | 0.141221374045802 | SMG1//RPL13A//RPL18A//RPL37A//RPS14//CNOT11//CNOT8//PPT1//PHKG1//KPNB1//HUWE1//NEDD4L//MDM2//RNF111//HECW2//USP42//UBE3B//PSMB5//ENC1//CLN6//TRIM24//GSK3B//LDLR//FAF1//PCBP2//RNF216//RNF4//IGF2BP3//ALKBH5//PUM1//DDX6//TAF15//TMF1//FTO//SIRT6//OPHN1//NEMF// |
| GO:0046907 | intracellular transport | Biological process | 43 | 1762 | 262 | 18866 | 1.75728050185857 | 0.000187489430779034 | 0.0456375135127311 | 3.72702320944944 | 0.16412213740458 | CDC40//NUP155//SMG1//ALKBH5//FIP1L1//GABARAP//KPNB1//RANBP17//KPNA5//PARD3//ARL6IP1//RPL13A//RPL18A//RPL37A//RPS14//CRAT//PITRM1//RABL2B//AP3S1//TBC1D1//RPH3AL//DYNC1H1//MIA3//TRAPPC4//HOOK3//SETD2//ABCD4//CORO1C//MYO19//TUBA1B//ANKRD27//DYNLRB1//SPAG9//NF1//RANGAP1//GSK3B//MDM2//NEMF//BACH2//BCR//LDLR//HUWE1//UBL5// |
| GO:0009653 | anatomical structure morphogenesis | Biological process | 61 | 2812 | 262 | 18866 | 1.56204326061698 | 0.000200737411242448 | 0.047233512865348 | 3.69737168091537 | 0.232824427480916 | FRYL//ARHGEF28//SETD2//RBPJ//MEIS1//MYH9//OVOL2//SRPK2//RFX2//TMF1//BMPR2//ACTB//THBS1//MIA3//SLIT3//MDM2//SIRT6//FLNB//PARD3//KLF7//NFASC//OPHN1//TRIO//ICAM1//FBLIM1//WDR1//HUWE1//TFRC//NF1//LARP4//ZMYM4//DNM2//GSK3B//KRT17//TTC39C//LPXN//PKDCC//AFF3//SDCCAG8//BCR//LMBR1//DDR1//EFEMP1//SH3PXD2B//TAOK3//ANKRD27//NEDD4L//HECW2//LDLR//TIAM2//CAPRIN1//SPI1//PSMB5//PAPPA2//STARD13//MYO19//CORO1C//DOCK1//NFATC2//IGF2BP3//EYA3// |
| GO:0000226 | microtubule cytoskeleton organization | Biological process | 20 | 591 | 262 | 18866 | 2.43680655119412 | 0.000236662934657992 | 0.0538904405080892 | 3.6258697544663 | 0.0763358778625954 | MYH9//CCSER2//TUBGCP2//DYNC1H1//SDCCAG8//CFAP44//KPNB1//DYRK1A//HOOK3//TTLL11//STARD9//GNAI1//GSK3B//RNF4//SETD2//TUBA1B//GABARAP//TBCEL//GAPDH//PARD3// |
| GO:0006611 | protein export from nucleus | Biological process | 10 | 184 | 262 | 18866 | 3.9134583471623 | 0.000259226510965315 | 0.057183748153255 | 3.5863205854512 | 0.0381679389312977 | SMG1//CDC40//ALKBH5//FIP1L1//NUP155//SETD2//RANGAP1//GSK3B//MDM2//RANBP17// |
| GO:0010628 | positive regulation of gene expression | Biological process | 52 | 2313 | 262 | 18866 | 1.61884865826411 | 0.000284293908863495 | 0.0581009745097646 | 3.54623244518227 | 0.198473282442748 | IFI16//TMF1//RBPJ//PLCG2//SETD2//PDE4D//THBS1//DYRK1A//HABP4//LSM14A//LARP4//KRT17//RPS27L//NAT10//ACTB//CTCF//DNM2//NFATC2//CRLF3//RNF111//GPBP1L1//RNF4//NPAS3//SPI1//TAF15//TRIM24//FANK1//DDX17//MEIS1//PLAGL1//OVOL2//RBL1//RFX2//BMPR2//NR2C2//NRIP1//KLF7//BRF1//TRA2B//GAPDH//PUM1//DDX11//CHCHD10//NCOR2//MYH9//SH3PXD2B//GSK3B//LDLR//MDM2//SRPK2//TFRC//ADAM19// |
| GO:0034248 | regulation of cellular amide metabolic process | Biological process | 18 | 509 | 262 | 18866 | 2.54643890880187 | 0.000287039599314487 | 0.0581009745097646 | 3.54205818487774 | 0.0687022900763359 | LSM14A//CNOT11//RPS14//IGF2BP3//RPL13A//GAPDH//CAPRIN1//ENC1//CNOT8//LARP4//KRT17//RPS27L//NAT10//THBS1//HABP4//FTO//PUM1//NEMF// |
| GO:0000902 | cell morphogenesis | Biological process | 29 | 1041 | 262 | 18866 | 2.00597634394409 | 0.000288076796690999 | 0.0581009745097646 | 3.5404917208063 | 0.110687022900763 | FLNB//PARD3//ACTB//KLF7//NFASC//OPHN1//SLIT3//TRIO//ICAM1//MYH9//FBLIM1//WDR1//LARP4//ZMYM4//DNM2//GSK3B//LPXN//TAOK3//ANKRD27//NEDD4L//HECW2//BMPR2//TIAM2//CAPRIN1//CORO1C//DOCK1//DDR1//FRYL//ARHGEF28// |
| GO:0061024 | membrane organization | Biological process | 27 | 944 | 262 | 18866 | 2.05954036744728 | 0.000312101660521387 | 0.061197933933902 | 3.50570392068629 | 0.103053435114504 | DOCK2//MYH9//DNM2//DOCK1//NUP155//NEK9//PPT1//ANKRD27//THBS1//PI4KA//NFASC//ATP8A1//GOLPH3L//TRAPPC4//HUWE1//MIA3//CORO1C//PITPNB//GSK3B//CHCHD10//PLEKHM2//ARL6IP1//TBC1D1//PIP5K1C//LDLR//ACTB//TFRC// |
| GO:0007049 | cell cycle | Biological process | 44 | 1882 | 262 | 18866 | 1.68349409025643 | 0.000399082197868942 | 0.0761384117501854 | 3.39893764465326 | 0.16793893129771 | ATRIP//ACTB//LATS2//NASP//CRLF3//KLF11//SDCCAG8//DYNC1H1//DNM2//MYH9//TUBA1B//TUBGCP2//PRDM5//TTC19//MDM2//PLAGL1//CNOT11//CNOT8//THBS1//DDX11//PDS5B//NEK9//KPNB1//TAOK3//RBL1//PSMB5//HECW2//RPS27L//SETD2//MYO19//KLHL21//CSPP1//PDE3A//SRPK2//STARD9//BCR//PUM1//GNAI1//CTCF//RNF4//CCDC124//GNAI2//CCAR1//PARD3// |
| GO:0048522 | positive regulation of cellular process | Biological process | 105 | 5722 | 262 | 18866 | 1.32135643598699 | 0.000460242090675758 | 0.0854960241600046 | 3.3370136661708 | 0.400763358778626 | THBS1//TFRC//MOB2//NF1//BMPR2//GSK3B//ARL6IP1//PLCG2//MIA3//ICAM1//RPS27L//MDM2//PLAGL1//CNOT11//CNOT8//RBPJ//SPAG9//GNAI2//MEIS1//CCAR1//SRPK2//AMBRA1//TMEM59//DNM2//DOCK1//NID1//LDLR//FAF1//ENC1//SESN1//KRT17//ATP8A1//RNF111//WDR1//NFATC2//HUWE1//PARD3//SIRT6//RPH3AL//DDX11//TAOK3//PKDCC//DYNC1H1//CSPP1//DYRK1A//HABP4//TERF2IP//DDR1//WDFY1//TADA2A//TIAM2//LATS2//TRIO//KLF11//FANK1//ZDHHC17//SH3PXD2B//OVOL2//LARP4//NAT10//EYA3//NEDD4L//CLN6//EVC//CTCF//CRLF3//GPBP1L1//RNF4//NPAS3//SPI1//TAF15//TRIM24//DDX17//IFI16//RBL1//RFX2//TMF1//NR2C2//NRIP1//KLF7//BRF1//TRA2B//PPT1//GOLPH3L//GAPDH//DOCK2//BCR//CAPRIN1//ANKRD27//PDE3A//LSM14A//FTO//PUM1//PSMB5//MORC2//GPR137B//CHCHD10//STAC//PLEKHM2//MYH9//UBL5//GNAI1//ADAM19//CD99L2//CORO1C// |
| GO:0030154 | cell differentiation | Biological process | 84 | 4349 | 262 | 18866 | 1.39081196168637 | 0.000486205907154849 | 0.0880032691950277 | 3.31317976900323 | 0.320610687022901 | PDE3A//CYP24A1//NF1//RFX2//TMF1//CORO1C//OVOL2//SDCCAG8//DDX17//GSK3B//RBPJ//SH3PXD2B//BMPR2//ACP6//ITFG2//PLCG2//SPI1//SIRT6//FLNB//CFAP44//PARD3//ACTB//KLF7//NFASC//OPHN1//SLIT3//TRIO//MYH9//MOB2//ENC1//MDM2//PTPRG//NFATC2//TRAPPC4//STRN//IFI16//BCR//RPS14//WDR1//TFRC//DNM2//MTR//FRYL//KRT17//EFEMP1//PKDCC//LPXN//SETD2//ANKRD27//MEIS1//GTF3C5//DOCK2//TMEM120B//FAM120B//TCFL5//THBS1//DDX6//SPAG9//GPR137B//PPT1//TAOK3//NEDD4L//HECW2//PUM1//HOOK3//TIAM2//CAPRIN1//ICAM1//PDE4D//DDR1//LDLR//FTO//DOCK1//PSMB5//HUWE1//EYA3//ALKBH5//AMBRA1//NHSL1//RBL1//ARHGEF28//SRPK2//NR2C2//USP42// |
| GO:0016032 | viral process | Biological process | 26 | 924 | 262 | 18866 | 2.02618882389875 | 0.000516473422941305 | 0.0911446473135668 | 3.28695202180536 | 0.099236641221374 | KPNB1//KPNA5//NEDD4L//DDX6//RPL13A//RPL18A//RPL37A//RPS14//NUP155//PCBP2//PI4KA//SRPK2//IFI16//MORC2//ICAM1//LDLR//TFRC//DOCK2//DDX11//DYRK1A//SETD2//MDM2//RNF216//PSMB5//RBL1//CANX// |
| GO:0044403 | symbiotic process | Biological process | 27 | 983 | 262 | 18866 | 1.97782920332678 | 0.000582932139684612 | 0.100363853025212 | 3.23438199940198 | 0.103053435114504 | DDX11//DYRK1A//SETD2//MDM2//RNF216//PSMB5//RBL1//CANX//NUP155//KPNB1//KPNA5//NEDD4L//DDX6//RPL13A//RPL18A//RPL37A//RPS14//PCBP2//PI4KA//ICAM1//SRPK2//IFI16//MORC2//LDLR//TFRC//DOCK2//GAPDH// |
| GO:0032502 | developmental process | Biological process | 118 | 6649 | 262 | 18866 | 1.27792160676174 | 0.00062295053174374 | 0.104700185799501 | 3.20554643912528 | 0.450381679389313 | FRYL//ARHGEF28//EVC//SH3PXD2B//PKDCC//SETD2//RBPJ//MEIS1//MYH9//OVOL2//SRPK2//ICAM1//NRIP1//PDE3A//MDM2//BMPR2//CYP24A1//NF1//RFX2//TMF1//ACTB//CORO1C//SDCCAG8//NASP//DDX17//GSK3B//ADAM19//THBS1//MIA3//PDS5B//ACP6//ITFG2//PLCG2//SPI1//SLIT3//SIRT6//DNM2//FLNB//TCFL5//DDX11//EYA3//LSM14A//G2E3//DDR1//ENC1//ZMYM4//GTF2IRD1//SCMH1//ALKBH5//NR2C2//USP42//PUM1//CFAP44//RNF111//IGF2BP3//DYRK1A//MTR//OPHN1//PPT1//PARD3//KLF7//NFASC//TRIO//PTPRG//BCR//UGP2//GCLM//PDE4D//RPN2//CANX//NCOR2//FBLIM1//WDR1//HUWE1//TFRC//MOB2//NFATC2//TRAPPC4//STRN//DDX6//COX6B1//PHLPP2//TRA2B//AMBRA1//HOOK3//LARP4//IFI16//NHSL1//RBL1//RPS14//KRT17//EFEMP1//TTC39C//NID1//LPXN//AFF3//ANKRD27//GTF3C5//FTO//LMBR1//DOCK2//TMEM120B//FAM120B//SPAG9//GPR137B//LATS2//TAOK3//NEDD4L//HECW2//LDLR//TIAM2//CAPRIN1//GNB1//PSMB5//PAPPA2//STARD13//MYO19//DOCK1// |
| GO:0044260 | cellular macromolecule metabolic process | Biological process | 143 | 8408 | 262 | 18866 | 1.22467787857439 | 0.000663832848520661 | 0.106499226767707 | 3.17794126114963 | 0.545801526717557 | ACTB//KLF11//CTCF//TCFL5//PRDM5//NEDD4L//IFI16//RBPJ//MDM2//NFATC2//SIRT6//OVOL2//RBL1//BACH2//RPS14//SPI1//NR2C2//NRIP1//KLF7//GTF2IRD1//NCOR2//NF1//TAOK3//PSMB5//SRPK2//SMG1//RPL13A//RPL18A//RPL37A//THBS1//HUWE1//WSB1//RNF111//HECW2//UBE3B//CNOT11//CNOT8//TERF2IP//CORO1C//TFRC//MOB2//DNM2//PPT1//PLCG2//GSK3B//UGP2//PHKG1//POLI//NASP//ATRIP//DDX11//PDS5B//TRRAP//SETD2//EYA3//ALKBH5//FTO//KPNB1//GPBP1L1//BRF1//ZNF483//EFEMP1//CCAR1//GATAD1//ZNF236//ZNF646//DDX17//ZNF652//LCORL//MEIS1//THAP4//PLAGL1//RFX2//NPAS3//TADA2A//PHRF1//TAF15//TRIM24//FIP1L1//GTF3C5//IGF2BP3//LARP4//RPS27L//LSM14A//UBL5//RPN1//RPN2//DYRK1A//LATS2//BCR//BMPR2//TRIO//NEK9//PHLPP2//PTPRG//SIRT5//GALNT1//GALNT7//USP42//FAF1//SPAG9//ENC1//PARD3//GAPDH//TMEM59//G2E3//DCAF17//KLHL21//RANGAP1//NUP155//CAPRIN1//TTLL11//CLK1//PKDCC//ZDHHC17//MTO1//NAT10//PDE4D//RNF4//DDR1//ARL6IP1//PCBP2//RNF216//PUM1//MIA3//RAB2A//DDX6//CLN6//PAPPA2//KRT17//SCMH1//CRLF3//FANK1//TMF1//HABP4//ICAM1//GNAI2//NEMF//MORC2//OPHN1//CHCHD10//MYH9//LDLR// |
| GO:0009893 | positive regulation of metabolic process | Biological process | 76 | 3880 | 262 | 18866 | 1.41045880223499 | 0.000677454635578597 | 0.106499226767707 | 3.16911978115837 | 0.290076335877863 | THBS1//IFI16//TMF1//TFRC//MOB2//PLCG2//RPS27L//RBPJ//SPAG9//GSK3B//AMBRA1//TMEM59//CTCF//SH3PXD2B//LDLR//MDM2//OVOL2//SRPK2//ADAM19//BMPR2//SESN1//HUWE1//RNF111//DDX11//TAOK3//SIRT6//SETD2//PDE4D//GNAI2//DYRK1A//HABP4//TERF2IP//DDR1//TADA2A//LSM14A//DNM2//ICAM1//LARP4//KRT17//NAT10//NEDD4L//HECW2//EYA3//FAF1//ACTB//CLN6//NFATC2//CRLF3//GPBP1L1//RNF4//NPAS3//SPI1//TAF15//TRIM24//FANK1//DDX17//MEIS1//PLAGL1//RBL1//RFX2//NR2C2//NRIP1//KLF7//BRF1//TRA2B//GAPDH//CNOT8//FTO//PUM1//MORC2//DYNC1H1//ELOVL6//CHCHD10//CCAR1//NCOR2//MYH9// |
| GO:0016071 | mRNA metabolic process | Biological process | 25 | 890 | 262 | 18866 | 2.02268633673557 | 0.000678915597754187 | 0.106499226767707 | 3.16818421350744 | 0.0954198473282443 | SMG1//RPL13A//RPL18A//RPL37A//RPS14//SRPK2//CNOT11//CNOT8//DDX17//DYRK1A//TRA2B//PCBP2//CDC40//CCAR1//UBL5//FIP1L1//HABP4//ALKBH5//PHRF1//IGF2BP3//PSMB5//PUM1//DDX6//TAF15//FTO// |
| GO:0120036 | plasma membrane bounded cell projection organization | Biological process | 37 | 1547 | 262 | 18866 | 1.72222523771693 | 0.000804533318714389 | 0.122983205187828 | 3.09445596544417 | 0.141221374045802 | OPHN1//CFAP44//PARD3//ACTB//KLF7//NFASC//SLIT3//TRIO//DNM2//GSK3B//MOB2//ENC1//MDM2//PTPRG//TRAPPC4//STRN//MTR//FRYL//MYH9//DYNLRB1//RABL2B//TAOK3//ANKRD27//NEDD4L//HECW2//BMPR2//TIAM2//CAPRIN1//TTC39C//CEP83//RFX2//DDR1//SDCCAG8//DYNC1H1//CORO1C//ICAM1//VDAC3// |
| GO:0006402 | mRNA catabolic process | Biological process | 14 | 376 | 262 | 18866 | 2.68113529316225 | 0.000822915932748434 | 0.122983205187828 | 3.08464452907484 | 0.0534351145038168 | SMG1//RPL13A//RPL18A//RPL37A//RPS14//CNOT11//CNOT8//IGF2BP3//ALKBH5//PSMB5//PUM1//DDX6//TAF15//FTO// |
| GO:0007017 | microtubule-based process | Biological process | 24 | 852 | 262 | 18866 | 2.02838404472637 | 0.000836264888655012 | 0.122983205187828 | 3.07765613708283 | 0.0916030534351145 | MYH9//TUBA1B//GABARAP//TTLL11//TBCEL//GAPDH//CFAP44//PARD3//CCSER2//DYNC1H1//STARD9//DYNLRB1//TUBGCP2//KPNB1//SDCCAG8//AP3S1//TMF1//DYRK1A//HOOK3//GSK3B//RABL2B//GNAI1//RNF4//SETD2// |
| GO:0048869 | cellular developmental process | Biological process | 84 | 4429 | 262 | 18866 | 1.365690047725 | 0.000879257985729032 | 0.124481541793384 | 3.05588367857849 | 0.320610687022901 | PDE3A//CYP24A1//NF1//RFX2//TMF1//CORO1C//OVOL2//SDCCAG8//DDX17//GSK3B//RBPJ//SH3PXD2B//BMPR2//ACP6//ITFG2//PLCG2//SPI1//SIRT6//FLNB//CFAP44//PARD3//ACTB//KLF7//NFASC//OPHN1//SLIT3//TRIO//MYH9//ICAM1//MOB2//ENC1//MDM2//PTPRG//NFATC2//TRAPPC4//STRN//IFI16//HUWE1//TCFL5//EYA3//ALKBH5//AMBRA1//NHSL1//RBL1//ARHGEF28//SRPK2//NR2C2//USP42//PKDCC//BCR//RPS14//WDR1//TFRC//DNM2//MTR//FRYL//KRT17//EFEMP1//LPXN//SETD2//ANKRD27//MEIS1//GTF3C5//DOCK2//TMEM120B//FAM120B//THBS1//DDX6//SPAG9//GPR137B//PPT1//TAOK3//NEDD4L//HECW2//PUM1//HOOK3//TIAM2//CAPRIN1//PDE4D//DDR1//LDLR//FTO//DOCK1//PSMB5// |
| GO:0051234 | establishment of localization | Biological process | 98 | 5352 | 262 | 18866 | 1.31852542817695 | 0.000893619490429736 | 0.124481541793384 | 3.04884736754042 | 0.374045801526718 | DNM2//ARL6IP1//PLCG2//HABP4//THBS1//WDR1//CDC40//NUP155//SMG1//ALKBH5//FIP1L1//GABARAP//KPNB1//RANBP17//KPNA5//PARD3//RPL13A//RPL18A//RPL37A//RPS14//CRAT//PITRM1//SLC15A4//NEDD4L//SLC23A2//SLC3A2//TFRC//SLC30A7//ATP8A1//RABL2B//AP3S1//TBC1D1//RPH3AL//MIA3//DYNC1H1//TRAPPC4//PITPNB//GOLPH3L//LDLR//PPT1//PIP5K1C//CORO1C//DOCK1//MYH9//DDR1//NCOR2//HOOK3//CANX//SETD2//PDE4D//AGAP1//GDI2//NASP//RAB2A//ANKRD27//PKDCC//HBG2//VDAC3//ABCD4//OSBPL10//SPAG9//ACTB//MYO19//OPHN1//TUBA1B//TMF1//ATP6V0B//TMEM120B//NALCN//ATP13A1//DYNLRB1//RAB11FIP5//GNAI2//NF1//ZDHHC17//HUWE1//DOCK2//NFASC//BCR//MCC//MDM2//SIRT6//RANGAP1//GSK3B//CEP83//GAPDH//IGF2BP3//NEMF//BACH2//ICAM1//PSMB5//KLF7//HK1//CHCHD10//STAC//COX6B1//UBL5//HECW2// |
| GO:0006810 | transport | Biological process | 96 | 5224 | 262 | 18866 | 1.32326432320587 | 0.000924034145942697 | 0.124481541793384 | 3.03431197994819 | 0.366412213740458 | DNM2//ARL6IP1//PLCG2//HABP4//THBS1//WDR1//CDC40//NUP155//SMG1//ALKBH5//FIP1L1//GABARAP//KPNB1//RANBP17//KPNA5//PARD3//RPL13A//RPL18A//RPL37A//RPS14//CRAT//PITRM1//SLC15A4//NEDD4L//SLC23A2//SLC3A2//TFRC//SLC30A7//ATP8A1//RABL2B//AP3S1//TBC1D1//RPH3AL//MIA3//DYNC1H1//TRAPPC4//PITPNB//GOLPH3L//LDLR//PPT1//PIP5K1C//CORO1C//DOCK1//MYH9//DDR1//NCOR2//HOOK3//CANX//SETD2//PDE4D//AGAP1//GDI2//NASP//RAB2A//ANKRD27//PKDCC//HBG2//VDAC3//ABCD4//OSBPL10//SPAG9//ACTB//MYO19//OPHN1//TUBA1B//TMF1//ATP6V0B//TMEM120B//NALCN//ATP13A1//DYNLRB1//RAB11FIP5//GNAI2//NF1//ZDHHC17//HUWE1//DOCK2//NFASC//BCR//SIRT6//RANGAP1//GSK3B//MDM2//CEP83//GAPDH//IGF2BP3//NEMF//BACH2//ICAM1//PSMB5//KLF7//CHCHD10//STAC//COX6B1//UBL5//HECW2// |
| GO:0010468 | regulation of gene expression | Biological process | 99 | 5425 | 262 | 18866 | 1.31405635487389 | 0.000932229891669894 | 0.124481541793384 | 3.03047697565879 | 0.377862595419847 | KLF11//CTCF//TCFL5//PRDM5//NEDD4L//IFI16//RBPJ//MDM2//NFATC2//SIRT6//OVOL2//RBL1//BACH2//RPS14//SPI1//NR2C2//NRIP1//KLF7//GTF2IRD1//NCOR2//SMG1//RPL13A//RPL18A//RPL37A//CNOT11//CNOT8//DDX17//DYRK1A//TRA2B//TMF1//ZNF483//DNM2//EFEMP1//SETD2//TERF2IP//CCAR1//GATAD1//GPBP1L1//ZNF236//TRRAP//ZNF646//ZNF652//LCORL//MEIS1//THAP4//PLAGL1//RFX2//NPAS3//TADA2A//LSM14A//FAF1//PUM1//SH3PXD2B//GSK3B//LDLR//SRPK2//TFRC//ADAM19//SLIT3//THBS1//TMEM59//SCMH1//IGF2BP3//GAPDH//CAPRIN1//ENC1//PCBP2//RNF216//PLCG2//PDE4D//HUWE1//HABP4//CLK1//ALKBH5//PSMB5//DDX6//LARP4//KRT17//RPS27L//NAT10//MORC2//ACTB//TRIM24//CRLF3//RNF111//RNF4//TAF15//FANK1//BMPR2//BRF1//ICAM1//NUP155//FTO//NEMF//DDX11//CHCHD10//MYH9//AFF3//NF1// |
| GO:0034645 | cellular macromolecule biosynthetic process | Biological process | 92 | 4963 | 262 | 18866 | 1.3348181120444 | 0.000947230865476218 | 0.124481541793384 | 3.02354415892394 | 0.351145038167939 | KLF11//CTCF//TCFL5//PRDM5//NEDD4L//IFI16//RBPJ//MDM2//NFATC2//SIRT6//OVOL2//RBL1//BACH2//RPS14//SPI1//NR2C2//NRIP1//KLF7//GTF2IRD1//NCOR2//RPL18A//PHKG1//UGP2//POLI//NASP//ATRIP//GPBP1L1//CNOT8//BRF1//ZNF483//DNM2//EFEMP1//SETD2//TERF2IP//CCAR1//GATAD1//ZNF236//TRRAP//ZNF646//DDX17//ZNF652//LCORL//MEIS1//THAP4//PLAGL1//RFX2//NPAS3//TADA2A//PHRF1//TAF15//TRIM24//FIP1L1//GTF3C5//IGF2BP3//LARP4//RPL13A//RPS27L//RPL37A//LSM14A//CNOT11//RPN1//RPN2//GALNT1//GALNT7//FAF1//GAPDH//CAPRIN1//ENC1//ZDHHC17//BMPR2//DDX11//NAT10//GSK3B//KRT17//THBS1//SCMH1//DYRK1A//CRLF3//RNF111//RNF4//FANK1//TMF1//HABP4//ICAM1//TFRC//FTO//PUM1//PSMB5//NEMF//TMEM59//MORC2//CHCHD10// |
| GO:0044238 | primary metabolic process | Biological process | 179 | 11109 | 262 | 18866 | 1.1602634271504 | 0.000957517450867949 | 0.124481541793384 | 3.0188533022193 | 0.683206106870229 | ACTB//KLF11//MTR//CTCF//TCFL5//PRDM5//NEDD4L//IFI16//RBPJ//MDM2//NFATC2//SIRT6//OVOL2//RBL1//BACH2//RPS14//SPI1//NR2C2//NRIP1//KLF7//GTF2IRD1//NCOR2//NAT10//NF1//TAOK3//PSMB5//SRPK2//SMG1//RPL13A//RPL18A//RPL37A//THBS1//HUWE1//WSB1//RNF111//HECW2//UBE3B//CNOT11//CNOT8//TRA2B//DDX17//DYRK1A//PCBP2//CDC40//CCAR1//UBL5//FIP1L1//RCL1//NOL10//TERF2IP//CLN6//CORO1C//TFRC//MOB2//PPT1//MTO1//GALNT7//PHKG1//SIAE//SLC3A2//GSK3B//UGP2//HK1//COQ2//GAPDH//MDH1//NUP155//SLC23A2//PDE4D//DCTD//POLI//DDX11//PDS5B//TRRAP//ATRIP//SETD2//EYA3//ALKBH5//FTO//KPNB1//GPBP1L1//BRF1//ZNF483//DNM2//EFEMP1//GATAD1//ZNF236//ZNF646//ZNF652//LCORL//MEIS1//THAP4//PLAGL1//RFX2//NPAS3//TADA2A//TEX10//PHRF1//TAF15//TRIM24//GTF3C5//HABP4//IGF2BP3//LARP4//RPS27L//LSM14A//RPN1//RPN2//LATS2//BCR//BMPR2//TRIO//NEK9//PHLPP2//PTPRG//SIRT5//GALNT1//PITRM1//PRSS57//DPP8//CFAP44//PAPPA2//TMEM59//MYH9//ADAM19//USP42//GCLM//PITPNB//LDLR//PDE3A//MORC2//ABCD4//ELOVL6//ACP6//PIP5K1C//PI4KA//PLCG2//FAF1//SPAG9//RPPH1//MBOAT2//BCKDHA//CYP24A1//ENC1//PARD3//G2E3//DCAF17//KLHL21//RANGAP1//CAPRIN1//TTLL11//CLK1//PKDCC//ZDHHC17//TIAM2//PCCB//RNF4//CRAT//SAGE1//PUM1//OSBPL10//DDR1//ARL6IP1//RNF216//AMBRA1//MIA3//RAB2A//DDX6//KRT17//SCMH1//CRLF3//FANK1//TMF1//ICAM1//GNAI2//NEMF//OPHN1//CHCHD10//LPXN// |
| GO:2000112 | regulation of cellular macromolecule biosynthetic process | Biological process | 77 | 3991 | 262 | 18866 | 1.38927281038826 | 0.000969894432445973 | 0.124481541793384 | 3.01327553367314 | 0.293893129770992 | KLF11//CTCF//TCFL5//PRDM5//NEDD4L//IFI16//RBPJ//MDM2//NFATC2//SIRT6//OVOL2//RBL1//BACH2//RPS14//SPI1//NR2C2//NRIP1//KLF7//GTF2IRD1//NCOR2//ZNF483//DNM2//EFEMP1//SETD2//TERF2IP//CCAR1//GATAD1//GPBP1L1//ZNF236//TRRAP//CNOT8//ZNF646//DDX17//ZNF652//LCORL//MEIS1//THAP4//PLAGL1//RFX2//NPAS3//TADA2A//LSM14A//CNOT11//FAF1//IGF2BP3//RPL13A//GAPDH//CAPRIN1//ENC1//NAT10//GSK3B//LARP4//KRT17//RPS27L//THBS1//SCMH1//TRIM24//DYRK1A//CRLF3//RNF111//RNF4//TAF15//FANK1//BMPR2//TMF1//BRF1//HABP4//ICAM1//TFRC//FTO//PUM1//PSMB5//NEMF//TMEM59//MORC2//DDX11//CHCHD10// |
| GO:0009056 | catabolic process | Biological process | 56 | 2683 | 262 | 18866 | 1.50295470775849 | 0.0010090112977221 | 0.124829766428048 | 2.99610397101691 | 0.213740458015267 | GABARAP//AMBRA1//SMG1//RPL13A//RPL18A//RPL37A//RPS14//CNOT11//CNOT8//PPT1//PHKG1//GAPDH//HK1//NUP155//PDE4D//KPNB1//HUWE1//NEDD4L//MDM2//RNF111//HECW2//USP42//UBE3B//ABCD4//IFI16//TRAPPC4//GPR137B//TMEM59//BCKDHA//PLCG2//PSMB5//ENC1//GSK3B//LDLR//SESN1//ATP6V0B//PCCB//CLN6//TRIM24//CRAT//FAF1//CYP24A1//HBG2//PCBP2//RNF216//RNF4//IGF2BP3//ALKBH5//PUM1//DDX6//SIRT6//TAF15//TMF1//FTO//OPHN1//NEMF// |
| GO:0051179 | localization | Biological process | 120 | 6865 | 262 | 18866 | 1.25869133729561 | 0.00101949829937042 | 0.124829766428048 | 2.99161349433377 | 0.458015267175573 | CORO1C//OVOL2//SDCCAG8//DOCK2//DNM2//ARL6IP1//MIA3//PLCG2//NFASC//HABP4//THBS1//WDR1//BCR//ICAM1//CDC40//NUP155//SMG1//ALKBH5//FIP1L1//GABARAP//KPNB1//RANBP17//KPNA5//PARD3//RPL13A//RPL18A//RPL37A//RPS14//CRAT//PITRM1//SLC15A4//NEDD4L//SLC23A2//SLC3A2//TFRC//SLC30A7//ATP8A1//RABL2B//AP3S1//TBC1D1//RPH3AL//DYNC1H1//TRAPPC4//PITPNB//GOLPH3L//LDLR//PPT1//PIP5K1C//DOCK1//MYH9//OPHN1//FAF1//CFAP44//DDR1//NCOR2//HOOK3//CANX//BMPR2//MCC//PTPRG//SETD2//PDE4D//FTO//AGAP1//GDI2//NASP//RAB2A//ANKRD27//PKDCC//HBG2//VDAC3//ABCD4//OSBPL10//SPAG9//NFATC2//NRIP1//ACTB//MYO19//TMF1//GNAI2//CCAR1//NF1//TUBA1B//PLEKHM2//ATP6V0B//TMEM120B//NALCN//ATP13A1//MDM2//DYNLRB1//RAB11FIP5//KLHL21//ZDHHC17//HUWE1//STARD13//LPXN//SIRT6//RANGAP1//GSK3B//CEP83//GAPDH//CD99L2//IGF2BP3//NEMF//BACH2//CHCHD10//PSMB5//KLF7//TERF2IP//CTCF//HK1//SH3PXD2B//PDS5B//GPR137B//STAC//COX6B1//TMEM59//UBL5//GNAI1//HECW2// |
| GO:0006974 | cellular response to DNA damage stimulus | Biological process | 24 | 865 | 262 | 18866 | 1.99789966023916 | 0.001028929159282 | 0.124829766428048 | 2.98761452493824 | 0.0916030534351145 | ATRIP//POLI//DDX11//PDS5B//SMG1//TAOK3//TRRAP//HUWE1//SIRT6//SETD2//EYA3//ALKBH5//FTO//MDM2//PLAGL1//CNOT11//CNOT8//RPS27L//TERF2IP//IFI16//DYRK1A//RNF111//MORC2//NFATC2// |
| GO:0006886 | intracellular protein transport | Biological process | 30 | 1183 | 262 | 18866 | 1.82606002335891 | 0.00105259776961965 | 0.124829766428048 | 2.97773755455711 | 0.114503816793893 | SMG1//CDC40//ALKBH5//FIP1L1//NUP155//GABARAP//KPNB1//RANBP17//KPNA5//PARD3//ARL6IP1//RPL13A//RPL18A//RPL37A//RPS14//CRAT//PITRM1//SETD2//DYNLRB1//RABL2B//NF1//RANGAP1//GSK3B//MDM2//BCR//HUWE1//UBL5//AP3S1//TBC1D1//RPH3AL// |
| GO:0048468 | cell development | Biological process | 48 | 2208 | 262 | 18866 | 1.56538333886492 | 0.00106102648897618 | 0.124829766428048 | 2.97427377361743 | 0.183206106870229 | PDE3A//RFX2//TMF1//CORO1C//OVOL2//BMPR2//SIRT6//FLNB//CFAP44//NF1//PARD3//ACTB//KLF7//NFASC//OPHN1//SLIT3//TRIO//GSK3B//MOB2//ENC1//MDM2//PTPRG//NFATC2//TRAPPC4//STRN//MYH9//WDR1//DNM2//MTR//FRYL//LPXN//MEIS1//DDX6//SPAG9//PPT1//TAOK3//ANKRD27//NEDD4L//HECW2//SETD2//HOOK3//TIAM2//CAPRIN1//ICAM1//PDE4D//DDR1//LDLR//DOCK1// |
| GO:0031669 | cellular response to nutrient levels | Biological process | 10 | 221 | 262 | 18866 | 3.25826396324825 | 0.00108203816879587 | 0.125214875959509 | 2.96575741926174 | 0.0381679389312977 | GABARAP//AMBRA1//BMPR2//SESN1//ITFG2//IFI16//CYP24A1//TRIM24//MDM2//ICAM1// |
| GO:0009889 | regulation of biosynthetic process | Biological process | 82 | 4342 | 262 | 18866 | 1.35988621699642 | 0.00119341003957012 | 0.135021541552909 | 2.92321031297125 | 0.312977099236641 | KLF11//CTCF//TCFL5//PRDM5//NEDD4L//IFI16//RBPJ//MDM2//NFATC2//SIRT6//OVOL2//RBL1//BACH2//RPS14//SPI1//NR2C2//NRIP1//KLF7//GTF2IRD1//NCOR2//ZNF483//DNM2//EFEMP1//SETD2//TERF2IP//CCAR1//GATAD1//GPBP1L1//ZNF236//TRRAP//CNOT8//ZNF646//DDX17//ZNF652//LCORL//MEIS1//THAP4//PLAGL1//RFX2//NPAS3//TADA2A//LSM14A//CNOT11//FAF1//SIRT5//LDLR//IGF2BP3//RPL13A//GAPDH//CAPRIN1//ENC1//NAT10//ICAM1//KPNB1//ELOVL6//GSK3B//LARP4//KRT17//RPS27L//THBS1//SCMH1//TRIM24//DYRK1A//CRLF3//RNF111//RNF4//TAF15//FANK1//BMPR2//TMF1//BRF1//HABP4//TFRC//FTO//PUM1//PSMB5//NEMF//TMEM59//MORC2//DDX11//CHCHD10//LPXN// |
| GO:0010604 | positive regulation of macromolecule metabolic process | Biological process | 70 | 3574 | 262 | 18866 | 1.41033417771266 | 0.0012050371324314 | 0.135021541552909 | 2.9189995703824 | 0.267175572519084 | THBS1//IFI16//TMF1//TFRC//MOB2//PLCG2//RPS27L//RBPJ//SPAG9//CTCF//SH3PXD2B//GSK3B//LDLR//MDM2//OVOL2//SRPK2//ADAM19//BMPR2//HUWE1//RNF111//DDX11//TAOK3//SIRT6//SETD2//PDE4D//DYRK1A//HABP4//TERF2IP//TADA2A//LSM14A//LARP4//KRT17//NAT10//NEDD4L//HECW2//EYA3//FAF1//ACTB//CLN6//DNM2//NFATC2//CRLF3//GPBP1L1//RNF4//NPAS3//SPI1//TAF15//TRIM24//FANK1//DDX17//MEIS1//PLAGL1//RBL1//RFX2//NR2C2//NRIP1//KLF7//BRF1//TRA2B//GAPDH//ICAM1//CNOT8//FTO//PUM1//GNAI2//MORC2//CHCHD10//CCAR1//NCOR2//MYH9// |
| GO:0048518 | positive regulation of biological process | Biological process | 112 | 6346 | 262 | 18866 | 1.27085643899024 | 0.00122850784745007 | 0.135500576486719 | 2.91062206499312 | 0.427480916030534 | THBS1//IFI16//TMF1//TFRC//MOB2//NF1//BMPR2//GSK3B//ARL6IP1//PLCG2//PSMB5//MIA3//ICAM1//RPS27L//MDM2//PLAGL1//CNOT11//CNOT8//RBPJ//SPAG9//GNAI2//MEIS1//CCAR1//SRPK2//AMBRA1//TMEM59//DNM2//CTCF//SH3PXD2B//LDLR//OVOL2//ADAM19//DOCK1//NID1//FAF1//ENC1//SESN1//KRT17//ATP8A1//PKDCC//RNF111//WDR1//NFATC2//HUWE1//PARD3//SIRT6//RPH3AL//DDX11//TAOK3//DYNC1H1//CSPP1//SETD2//PDE4D//DYRK1A//HABP4//TERF2IP//DDR1//WDFY1//TADA2A//ACTB//LSM14A//NR2C2//TIAM2//LATS2//TRIO//KLF11//FANK1//ZDHHC17//LARP4//NAT10//NEDD4L//HECW2//EYA3//CLN6//EVC//CRLF3//GPBP1L1//RNF4//NPAS3//SPI1//TAF15//TRIM24//DDX17//RBL1//RFX2//NRIP1//KLF7//BRF1//TRA2B//PPT1//GOLPH3L//GAPDH//DOCK2//BCR//CAPRIN1//ANKRD27//LPXN//PDE3A//FTO//PUM1//MORC2//ELOVL6//GPR137B//CHCHD10//STAC//PLEKHM2//NCOR2//MYH9//UBL5//GNAI1//CD99L2//CORO1C// |
| GO:0010556 | regulation of macromolecule biosynthetic process | Biological process | 78 | 4094 | 262 | 18866 | 1.37190899361195 | 0.00128800345485145 | 0.135563069814729 | 2.89008297205314 | 0.297709923664122 | KLF11//CTCF//TCFL5//PRDM5//NEDD4L//IFI16//RBPJ//MDM2//NFATC2//SIRT6//OVOL2//RBL1//BACH2//RPS14//SPI1//NR2C2//NRIP1//KLF7//GTF2IRD1//NCOR2//ZNF483//DNM2//EFEMP1//SETD2//TERF2IP//CCAR1//GATAD1//GPBP1L1//ZNF236//TRRAP//CNOT8//ZNF646//DDX17//ZNF652//LCORL//MEIS1//THAP4//PLAGL1//RFX2//NPAS3//TADA2A//LSM14A//CNOT11//FAF1//IGF2BP3//RPL13A//GAPDH//CAPRIN1//ENC1//NAT10//GSK3B//LARP4//KRT17//RPS27L//THBS1//SCMH1//TRIM24//DYRK1A//CRLF3//RNF111//RNF4//TAF15//FANK1//BMPR2//TMF1//BRF1//HABP4//ICAM1//TFRC//FTO//PUM1//PSMB5//NEMF//TMEM59//MORC2//DDX11//CHCHD10//LPXN// |
| GO:1903729 | regulation of plasma membrane organization | Biological process | 3 | 16 | 262 | 18866 | 13.5014312977099 | 0.00129694861230799 | 0.135563069814729 | 2.88707723118901 | 0.0114503816793893 | DNM2//PLEKHM2//MYH9// |
| GO:0030030 | cell projection organization | Biological process | 37 | 1589 | 262 | 18866 | 1.6767038657949 | 0.00130398809398559 | 0.135563069814729 | 2.88472637389548 | 0.141221374045802 | OPHN1//CFAP44//PARD3//ACTB//KLF7//NFASC//SLIT3//TRIO//DNM2//GSK3B//MOB2//ENC1//MDM2//PTPRG//TRAPPC4//STRN//MTR//FRYL//MYH9//DYNLRB1//RABL2B//TAOK3//ANKRD27//NEDD4L//HECW2//BMPR2//TIAM2//CAPRIN1//TTC39C//CEP83//RFX2//DDR1//SDCCAG8//DYNC1H1//CORO1C//ICAM1//VDAC3// |
| GO:0051028 | mRNA transport | Biological process | 8 | 152 | 262 | 18866 | 3.78987545198875 | 0.00130589159192542 | 0.135563069814729 | 2.88409287434941 | 0.0305343511450382 | SMG1//CDC40//ALKBH5//FIP1L1//NUP155//SETD2//IGF2BP3//RANBP17// |
| GO:0009059 | macromolecule biosynthetic process | Biological process | 93 | 5091 | 262 | 18866 | 1.31540167426127 | 0.0014337698365113 | 0.146680888057004 | 2.84352056049238 | 0.354961832061069 | KLF11//CTCF//TCFL5//PRDM5//NEDD4L//IFI16//RBPJ//MDM2//NFATC2//SIRT6//OVOL2//RBL1//BACH2//RPS14//SPI1//NR2C2//NRIP1//KLF7//GTF2IRD1//NCOR2//RPL18A//PHKG1//UGP2//POLI//NASP//ATRIP//GPBP1L1//CNOT8//BRF1//ZNF483//DNM2//EFEMP1//SETD2//TERF2IP//CCAR1//GATAD1//ZNF236//TRRAP//ZNF646//DDX17//ZNF652//LCORL//MEIS1//THAP4//PLAGL1//RFX2//NPAS3//TADA2A//PHRF1//TAF15//TRIM24//FIP1L1//GTF3C5//IGF2BP3//LARP4//RPL13A//RPS27L//RPL37A//LSM14A//CNOT11//RPN1//RPN2//GALNT1//GALNT7//FAF1//GAPDH//CAPRIN1//ENC1//ZDHHC17//BMPR2//DDX11//NAT10//GSK3B//KRT17//THBS1//SCMH1//DYRK1A//CRLF3//RNF111//RNF4//FANK1//TMF1//HABP4//ICAM1//TFRC//FTO//PUM1//PSMB5//NEMF//TMEM59//MORC2//CHCHD10//LPXN// |
| GO:0008625 | extrinsic apoptotic signaling pathway via death domain receptors | Biological process | 6 | 89 | 262 | 18866 | 4.85444720816537 | 0.00150382172446956 | 0.151649679329009 | 2.82280364557163 | 0.0229007633587786 | GSK3B//ICAM1//FAF1//THBS1//GABARAP//NF1// |
| GO:0031326 | regulation of cellular biosynthetic process | Biological process | 80 | 4255 | 262 | 18866 | 1.35384504982912 | 0.00161073306453791 | 0.160143164824973 | 2.79297642618767 | 0.305343511450382 | KLF11//CTCF//TCFL5//PRDM5//NEDD4L//IFI16//RBPJ//MDM2//NFATC2//SIRT6//OVOL2//RBL1//BACH2//RPS14//SPI1//NR2C2//NRIP1//KLF7//GTF2IRD1//NCOR2//ZNF483//DNM2//EFEMP1//SETD2//TERF2IP//CCAR1//GATAD1//GPBP1L1//ZNF236//TRRAP//CNOT8//ZNF646//DDX17//ZNF652//LCORL//MEIS1//THAP4//PLAGL1//RFX2//NPAS3//TADA2A//LSM14A//CNOT11//FAF1//SIRT5//LDLR//IGF2BP3//RPL13A//GAPDH//CAPRIN1//ENC1//NAT10//ICAM1//GSK3B//LARP4//KRT17//RPS27L//THBS1//SCMH1//TRIM24//DYRK1A//CRLF3//RNF111//RNF4//TAF15//FANK1//BMPR2//TMF1//BRF1//HABP4//TFRC//FTO//PUM1//PSMB5//NEMF//TMEM59//MORC2//DDX11//CHCHD10//LPXN// |
| GO:0048488 | synaptic vesicle endocytosis | Biological process | 5 | 62 | 262 | 18866 | 5.80706722482147 | 0.00168777234149318 | 0.163205273405484 | 2.77268613442906 | 0.0190839694656489 | DNM2//PIP5K1C//OPHN1//ACTB//CANX// |
| GO:0140238 | presynaptic endocytosis | Biological process | 5 | 62 | 262 | 18866 | 5.80706722482147 | 0.00168777234149318 | 0.163205273405484 | 2.77268613442906 | 0.0190839694656489 | DNM2//PIP5K1C//OPHN1//ACTB//CANX// |
| GO:0022604 | regulation of cell morphogenesis | Biological process | 16 | 499 | 262 | 18866 | 2.30886199880677 | 0.00172294138580152 | 0.164354638410445 | 2.76372949693152 | 0.0610687022900763 | ICAM1//MYH9//FBLIM1//WDR1//DNM2//GSK3B//NEDD4L//HECW2//BMPR2//TIAM2//CAPRIN1//ANKRD27//CORO1C//DOCK1//LARP4//ZMYM4// |
| GO:1901576 | organic substance biosynthetic process | Biological process | 109 | 6204 | 262 | 18866 | 1.26512444569128 | 0.00180012239584392 | 0.168377329953239 | 2.74469796487866 | 0.416030534351145 | KLF11//CTCF//TCFL5//PRDM5//NEDD4L//IFI16//RBPJ//MDM2//NFATC2//SIRT6//OVOL2//RBL1//BACH2//RPS14//SPI1//NR2C2//NRIP1//KLF7//GTF2IRD1//NCOR2//RPL18A//PHKG1//UGP2//GAPDH//MDH1//PDE4D//DCTD//POLI//NASP//ATRIP//GPBP1L1//CNOT8//BRF1//ZNF483//DNM2//EFEMP1//SETD2//TERF2IP//CCAR1//GATAD1//ZNF236//TRRAP//ZNF646//DDX17//ZNF652//LCORL//MEIS1//THAP4//PLAGL1//RFX2//NPAS3//TADA2A//PHRF1//TAF15//TRIM24//FIP1L1//GTF3C5//IGF2BP3//LARP4//RPL13A//RPS27L//RPL37A//LSM14A//CNOT11//RPN1//RPN2//GALNT1//GALNT7//ELOVL6//ACP6//PIP5K1C//PI4KA//PLCG2//COQ2//GCLM//FAF1//MBOAT2//MTR//SIRT5//LDLR//CAPRIN1//ENC1//ZDHHC17//BMPR2//DDX11//NAT10//KPNB1//GSK3B//KRT17//THBS1//SCMH1//DYRK1A//CRLF3//RNF111//RNF4//FANK1//TMF1//HABP4//PPT1//ICAM1//TFRC//FTO//PUM1//PSMB5//NEMF//TMEM59//MORC2//CHCHD10//LPXN// |
| GO:0031571 | mitotic G1 DNA damage checkpoint | Biological process | 5 | 63 | 262 | 18866 | 5.71489155458621 | 0.00181281726539824 | 0.168377329953239 | 2.74164597121 | 0.0190839694656489 | MDM2//PLAGL1//CNOT11//CNOT8//RPS27L// |
| GO:0048519 | negative regulation of biological process | Biological process | 107 | 6078 | 262 | 18866 | 1.26765659656024 | 0.00191412591379604 | 0.173751014184156 | 2.71802949714006 | 0.408396946564886 | ATRIP//CTCF//TCFL5//PRDM5//NEDD4L//IFI16//RBPJ//MDM2//NFATC2//SIRT6//OVOL2//RBL1//BACH2//RPS14//SPI1//NR2C2//NRIP1//KLF11//KLF7//GTF2IRD1//NCOR2//SMG1//RPL13A//RPL18A//RPL37A//CNOT11//CNOT8//CORO1C//TERF2IP//NF1//THBS1//DNM2//BCR//BMPR2//TAOK3//PLAGL1//MIA3//LPXN//FAF1//PDS5B//AMBRA1//SLIT3//STRN//DDR1//TRIM24//LDLR//TMF1//MCC//PTPRG//HUWE1//TFRC//SIRT5//PARD3//PDE4D//GAPDH//TMEM59//PSMB5//PUM1//SCMH1//IGF2BP3//CAPRIN1//ENC1//CRLF3//PPT1//DDX17//DYRK1A//GSK3B//RPS27L//NAT10//EFEMP1//PCBP2//RNF216//RNF4//ANKRD27//PKDCC//ARL6IP1//PDE3A//FANK1//PLCG2//ALKBH5//GCLM//DDX6//SRPK2//TRIO//MEIS1//GPR137B//LATS2//RPH3AL//MORC2//GNAI2//RANGAP1//TAF15//HOOK3//GNAI1//PHLPP2//ICAM1//NUP155//FTO//RAB11FIP5//STARD13//OPHN1//CCAR1//MYH9//SESN1//ITFG2//HECW2//EYA3// |
| GO:0044783 | G1 DNA damage checkpoint | Biological process | 5 | 64 | 262 | 18866 | 5.6255963740458 | 0.00194451482087382 | 0.173751014184156 | 2.71118874236009 | 0.0190839694656489 | MDM2//PLAGL1//CNOT11//CNOT8//RPS27L// |
| GO:0044819 | mitotic G1/S transition checkpoint | Biological process | 5 | 64 | 262 | 18866 | 5.6255963740458 | 0.00194451482087382 | 0.173751014184156 | 2.71118874236009 | 0.0190839694656489 | MDM2//PLAGL1//CNOT11//CNOT8//RPS27L// |
| GO:0000904 | cell morphogenesis involved in differentiation | Biological process | 21 | 753 | 262 | 18866 | 2.00818101639245 | 0.00198402287620919 | 0.175065218539508 | 2.70245332464447 | 0.0801526717557252 | FLNB//PARD3//ACTB//KLF7//NFASC//OPHN1//SLIT3//TRIO//MYH9//WDR1//DNM2//GSK3B//LPXN//NEDD4L//HECW2//BMPR2//TIAM2//CAPRIN1//ANKRD27//CORO1C//DOCK1// |
| GO:0006401 | RNA catabolic process | Biological process | 14 | 415 | 262 | 18866 | 2.42917318127472 | 0.00208189580705462 | 0.181433364222204 | 2.68154100948136 | 0.0534351145038168 | SMG1//RPL13A//RPL18A//RPL37A//RPS14//CNOT11//CNOT8//IGF2BP3//ALKBH5//PSMB5//PUM1//DDX6//TAF15//FTO// |
| GO:1901575 | organic substance catabolic process | Biological process | 47 | 2222 | 262 | 18866 | 1.52311376175786 | 0.00211185250555698 | 0.181799595569838 | 2.67533641675354 | 0.179389312977099 | SMG1//RPL13A//RPL18A//RPL37A//RPS14//CNOT11//CNOT8//PPT1//PHKG1//GAPDH//HK1//NUP155//PDE4D//KPNB1//HUWE1//NEDD4L//MDM2//RNF111//HECW2//USP42//UBE3B//ABCD4//BCKDHA//PLCG2//PSMB5//ENC1//LDLR//PCCB//CLN6//TRIM24//GSK3B//CRAT//FAF1//CYP24A1//PCBP2//RNF216//RNF4//IGF2BP3//ALKBH5//PUM1//DDX6//SIRT6//TAF15//TMF1//FTO//OPHN1//NEMF// |
| GO:1903827 | regulation of cellular protein localization | Biological process | 17 | 560 | 262 | 18866 | 2.18594601962923 | 0.00222065983177806 | 0.18782984423461 | 2.65351796306173 | 0.0648854961832061 | SETD2//NF1//PKDCC//RANGAP1//GSK3B//MDM2//OPHN1//GPR137B//TFRC//ACTB//TMEM59//STAC//HUWE1//UBL5//GNAI1//RAB11FIP5//NEDD4L// |
| GO:0044249 | cellular biosynthetic process | Biological process | 107 | 6104 | 262 | 18866 | 1.26225701079507 | 0.00223511926841015 | 0.18782984423461 | 2.65069929748489 | 0.408396946564886 | KLF11//CTCF//TCFL5//PRDM5//NEDD4L//IFI16//RBPJ//MDM2//NFATC2//SIRT6//OVOL2//RBL1//BACH2//RPS14//SPI1//NR2C2//NRIP1//KLF7//GTF2IRD1//NCOR2//RPL18A//PHKG1//UGP2//PDE4D//DCTD//POLI//NASP//ATRIP//GPBP1L1//CNOT8//BRF1//ZNF483//DNM2//EFEMP1//SETD2//TERF2IP//CCAR1//GATAD1//ZNF236//TRRAP//ZNF646//DDX17//ZNF652//LCORL//MEIS1//THAP4//PLAGL1//RFX2//NPAS3//TADA2A//PHRF1//TAF15//TRIM24//FIP1L1//GTF3C5//IGF2BP3//LARP4//RPL13A//RPS27L//RPL37A//LSM14A//CNOT11//RPN1//RPN2//GALNT1//GALNT7//ELOVL6//ACP6//PIP5K1C//PI4KA//PLCG2//COQ2//GCLM//FAF1//MBOAT2//MTR//SIRT5//LDLR//GAPDH//CAPRIN1//ENC1//ZDHHC17//BMPR2//DDX11//NAT10//ICAM1//GSK3B//KRT17//THBS1//SCMH1//DYRK1A//CRLF3//RNF111//RNF4//FANK1//TMF1//HABP4//PPT1//TFRC//FTO//PUM1//PSMB5//NEMF//TMEM59//MORC2//CHCHD10//LPXN// |
| GO:0043043 | peptide biosynthetic process | Biological process | 21 | 763 | 262 | 18866 | 1.9818614748932 | 0.00232168554342862 | 0.18929368284229 | 2.63419660285836 | 0.0801526717557252 | RPL18A//IGF2BP3//LARP4//RPL13A//RPS27L//RPL37A//RPS14//LSM14A//CNOT11//GCLM//GAPDH//CAPRIN1//ENC1//CNOT8//KRT17//NAT10//THBS1//HABP4//FTO//PUM1//NEMF// |
| GO:1990823 | response to leukemia inhibitory factor | Biological process | 6 | 97 | 262 | 18866 | 4.45408042811049 | 0.00233298631637332 | 0.18929368284229 | 2.63208780845914 | 0.0229007633587786 | PRDM5//GCLM//ICAM1//ABCD4//RFX2//TFRC// |
| GO:1990830 | cellular response to leukemia inhibitory factor | Biological process | 6 | 97 | 262 | 18866 | 4.45408042811049 | 0.00233298631637332 | 0.18929368284229 | 2.63208780845914 | 0.0229007633587786 | PRDM5//GCLM//ICAM1//ABCD4//RFX2//TFRC// |
| GO:0010256 | endomembrane system organization | Biological process | 15 | 468 | 262 | 18866 | 2.30793697396751 | 0.0023903730211403 | 0.191745944957152 | 2.62153432148619 | 0.0572519083969466 | RFX2//TMF1//MYH9//NUP155//MIA3//HUWE1//PLEKHM2//GOLPH3L//RAB2A//HOOK3//NEK9//ARL6IP1//CORO1C//DNM2//PDE4DIP// |
| GO:0006928 | movement of cell or subcellular component | Biological process | 48 | 2298 | 262 | 18866 | 1.50407589739501 | 0.00243043401207295 | 0.19276891787891 | 2.61431616581825 | 0.183206106870229 | CORO1C//OVOL2//SDCCAG8//MIA3//BCR//ICAM1//OPHN1//DYNC1H1//STARD9//DYNLRB1//KPNB1//CFAP44//NFASC//SLIT3//TRIO//KLF7//AP3S1//BMPR2//THBS1//MCC//PTPRG//DOCK1//PLCG2//DDR1//NFATC2//MYH9//MYO9B//MYO19//TMF1//ATP8A1//GNAI2//CCAR1//SPAG9//NF1//PIP5K1C//PDE4D//SETD2//RABL2B//STARD13//LPXN//ACTB//SLC3A2//CD99L2//NEDD4L//NUP155//PDS5B//MDM2//WDR1// |
| GO:0046825 | regulation of protein export from nucleus | Biological process | 4 | 41 | 262 | 18866 | 7.02513498417427 | 0.00245860333351192 | 0.192836454791785 | 2.60931153387334 | 0.0152671755725191 | SETD2//RANGAP1//GSK3B//MDM2// |
| GO:0009894 | regulation of catabolic process | Biological process | 26 | 1032 | 262 | 18866 | 1.81414580744423 | 0.00248950251905725 | 0.193114266835441 | 2.60388742993799 | 0.099236641221374 | NUP155//IFI16//GPR137B//SMG1//GSK3B//AMBRA1//TMEM59//LDLR//SESN1//GAPDH//ATP6V0B//MDM2//FAF1//NEDD4L//IGF2BP3//ALKBH5//PSMB5//PUM1//HUWE1//HECW2//SIRT6//TAF15//CNOT8//TMF1//FTO//OPHN1// |
| GO:0022402 | cell cycle process | Biological process | 33 | 1422 | 262 | 18866 | 1.67106322672078 | 0.00254102826983539 | 0.19496867996487 | 2.59499050324352 | 0.125954198473282 | LATS2//NASP//CRLF3//KLF11//SDCCAG8//DYNC1H1//DNM2//MYH9//TTC19//MDM2//PLAGL1//CNOT11//CNOT8//THBS1//DDX11//PDS5B//NEK9//KPNB1//TAOK3//PSMB5//HECW2//RPS27L//SETD2//MYO19//KLHL21//CSPP1//PDE3A//TUBGCP2//STARD9//GNAI1//CTCF//RNF4//RBL1// |
| GO:0031329 | regulation of cellular catabolic process | Biological process | 23 | 874 | 262 | 18866 | 1.89493772599437 | 0.00256903891402819 | 0.194998340797043 | 2.59022931729079 | 0.0877862595419847 | IFI16//GPR137B//SMG1//GSK3B//AMBRA1//TMEM59//LDLR//SESN1//GAPDH//ATP6V0B//MDM2//IGF2BP3//ALKBH5//PSMB5//PUM1//TAF15//CNOT8//TMF1//FTO//HUWE1//SIRT6//OPHN1//FAF1// |
| GO:0031503 | protein-containing complex localization | Biological process | 11 | 291 | 262 | 18866 | 2.72193803940086 | 0.00259834039914535 | 0.19512430720816 | 2.58530395419198 | 0.0419847328244275 | SMG1//CDC40//ALKBH5//FIP1L1//NUP155//SETD2//DYNLRB1//KLHL21//RABL2B//DNM2//OPHN1// |
| GO:0031667 | response to nutrient levels | Biological process | 15 | 473 | 262 | 18866 | 2.28354017720252 | 0.00264345204697091 | 0.196422399995449 | 2.57782856342222 | 0.0572519083969466 | GABARAP//GCLM//GNAI2//AMBRA1//BMPR2//ICAM1//CYP24A1//SESN1//ITFG2//IFI16//LDLR//TRIM24//MDM2//SIRT5//SIRT6// |
| GO:0070925 | organelle assembly | Biological process | 23 | 879 | 262 | 18866 | 1.88415878557347 | 0.0027580528177917 | 0.202803071258246 | 2.5593974211711 | 0.0877862595419847 | RPS27L//RPS14//GABARAP//AMBRA1//RFX2//TMF1//CFAP44//DDX6//DYNC1H1//LSM14A//DYNLRB1//WDR1//TUBGCP2//STARD9//RABL2B//TTC39C//CEP83//SH3PXD2B//RNF4//KPNB1//SDCCAG8//VDAC3//DNM2// |
| GO:0044773 | mitotic DNA damage checkpoint | Biological process | 6 | 101 | 262 | 18866 | 4.27768120323483 | 0.00285807556499079 | 0.206574576855692 | 2.54392629303116 | 0.0229007633587786 | MDM2//PLAGL1//CNOT11//CNOT8//TAOK3//RPS27L// |
| GO:0044237 | cellular metabolic process | Biological process | 180 | 11384 | 262 | 18866 | 1.13856061540772 | 0.00292153764402498 | 0.206574576855692 | 2.53438851343141 | 0.687022900763359 | GABARAP//AMBRA1//ACTB//KLF11//MTR//CTCF//TCFL5//PRDM5//NEDD4L//IFI16//RBPJ//MDM2//NFATC2//SIRT6//OVOL2//RBL1//BACH2//RPS14//SPI1//NR2C2//NRIP1//KLF7//GTF2IRD1//NCOR2//NAT10//NF1//TAOK3//PSMB5//SRPK2//SMG1//RPL13A//RPL18A//RPL37A//THBS1//HUWE1//WSB1//RNF111//HECW2//UBE3B//CNOT11//CNOT8//TRA2B//DDX17//DYRK1A//PCBP2//CDC40//CCAR1//UBL5//FIP1L1//RCL1//NOL10//TERF2IP//CLN6//CORO1C//TFRC//MOB2//DNM2//PPT1//PLCG2//MTO1//GSK3B//UGP2//PHKG1//HK1//COQ2//GAPDH//MDH1//NUP155//CHCHD10//NDUFB2//COX6B1//SLC23A2//PDE4D//DCTD//POLI//NASP//ATRIP//DDX11//PDS5B//TRRAP//SETD2//EYA3//ALKBH5//FTO//KPNB1//GPBP1L1//BRF1//ZNF483//EFEMP1//GATAD1//ZNF236//ZNF646//ZNF652//LCORL//MEIS1//THAP4//PLAGL1//RFX2//NPAS3//TADA2A//TEX10//PHRF1//TAF15//TRIM24//GTF3C5//HABP4//IGF2BP3//LARP4//RPS27L//LSM14A//RPN1//RPN2//LATS2//BCR//BMPR2//TRIO//NEK9//PHLPP2//PTPRG//SIRT5//GALNT1//GALNT7//USP42//GCLM//MORC2//ABCD4//ELOVL6//ACP6//PIP5K1C//PI4KA//AHCYL2//PCCB//SH3PXD2B//TRAPPC4//GPR137B//TMEM59//FAF1//SPAG9//RPPH1//MBOAT2//BCKDHA//CYP24A1//ENC1//PARD3//LDLR//SESN1//ATP6V0B//G2E3//DCAF17//KLHL21//RANGAP1//CAPRIN1//TTLL11//CLK1//PKDCC//ZDHHC17//CRAT//GNAI2//RNF4//DDR1//SAGE1//PUM1//OSBPL10//HBG2//ARL6IP1//RNF216//MIA3//RAB2A//DDX6//PAPPA2//ICAM1//KRT17//SCMH1//CRLF3//FANK1//TMF1//NEMF//OPHN1//LPXN//MYH9// |
| GO:0031668 | cellular response to extracellular stimulus | Biological process | 10 | 253 | 262 | 18866 | 2.84615152520894 | 0.00292587995326802 | 0.206574576855692 | 2.53374349663272 | 0.0381679389312977 | GABARAP//AMBRA1//BMPR2//ICAM1//SESN1//ITFG2//IFI16//CYP24A1//TRIM24//MDM2// |
| GO:0033962 | P-body assembly | Biological process | 3 | 21 | 262 | 18866 | 10.2868047982552 | 0.002926400012122 | 0.206574576855692 | 2.53366631006868 | 0.0114503816793893 | DDX6//DYNC1H1//LSM14A// |
| GO:0009058 | biosynthetic process | Biological process | 109 | 6290 | 262 | 18866 | 1.24782703673588 | 0.00297777590698975 | 0.208120001261789 | 2.526107988256 | 0.416030534351145 | KLF11//CTCF//TCFL5//PRDM5//NEDD4L//IFI16//RBPJ//MDM2//NFATC2//SIRT6//OVOL2//RBL1//BACH2//RPS14//SPI1//NR2C2//NRIP1//KLF7//GTF2IRD1//NCOR2//RPL18A//PHKG1//UGP2//GAPDH//MDH1//PDE4D//DCTD//POLI//NASP//ATRIP//GPBP1L1//CNOT8//BRF1//ZNF483//DNM2//EFEMP1//SETD2//TERF2IP//CCAR1//GATAD1//ZNF236//TRRAP//ZNF646//DDX17//ZNF652//LCORL//MEIS1//THAP4//PLAGL1//RFX2//NPAS3//TADA2A//PHRF1//TAF15//TRIM24//FIP1L1//GTF3C5//IGF2BP3//LARP4//RPL13A//RPS27L//RPL37A//LSM14A//CNOT11//RPN1//RPN2//GALNT1//GALNT7//ELOVL6//ACP6//PIP5K1C//PI4KA//PLCG2//COQ2//GCLM//FAF1//MBOAT2//MTR//SIRT5//LDLR//CAPRIN1//ENC1//ZDHHC17//BMPR2//DDX11//NAT10//ICAM1//KPNB1//GSK3B//KRT17//THBS1//SCMH1//DYRK1A//CRLF3//RNF111//RNF4//FANK1//TMF1//HABP4//PPT1//TFRC//FTO//PUM1//PSMB5//NEMF//TMEM59//MORC2//CHCHD10//LPXN// |
| GO:0019058 | viral life cycle | Biological process | 12 | 341 | 262 | 18866 | 2.53399297083119 | 0.00303363506699294 | 0.20994539154807 | 2.51803666413056 | 0.0458015267175573 | DDX6//PCBP2//PI4KA//SRPK2//IFI16//MORC2//ICAM1//LDLR//TFRC//KPNB1//NUP155//NEDD4L// |
| GO:0044267 | cellular protein metabolic process | Biological process | 95 | 5350 | 262 | 18866 | 1.27864022258686 | 0.00314421876013167 | 0.215485827454072 | 2.50248724538933 | 0.362595419847328 | ACTB//NF1//TAOK3//PSMB5//SRPK2//THBS1//HUWE1//NEDD4L//WSB1//MDM2//RNF111//HECW2//UBE3B//CORO1C//TERF2IP//TFRC//MOB2//PPT1//RPL18A//IGF2BP3//LARP4//RPL13A//RPS27L//RPL37A//RPS14//LSM14A//CNOT11//UBL5//RPN1//RPN2//DYRK1A//LATS2//GSK3B//PHKG1//BCR//BMPR2//TRIO//NEK9//PHLPP2//PTPRG//SIRT5//SIRT6//GALNT1//GALNT7//USP42//SPAG9//ENC1//PARD3//GAPDH//TMEM59//G2E3//DCAF17//TRIM24//KLHL21//TRRAP//PRDM5//EYA3//RANGAP1//NUP155//CAPRIN1//CNOT8//SETD2//TTLL11//CLK1//SMG1//EFEMP1//PKDCC//ZDHHC17//CTCF//PDE4D//RNF4//TADA2A//DDR1//ARL6IP1//FAF1//PCBP2//RNF216//MIA3//RAB2A//SPI1//KRT17//NAT10//CLN6//HABP4//ICAM1//TMF1//FTO//PUM1//GNAI2//NEMF//IFI16//OPHN1//MYH9//LDLR//PAPPA2// |
| GO:0010498 | proteasomal protein catabolic process | Biological process | 15 | 483 | 262 | 18866 | 2.23626191266417 | 0.00321639672699887 | 0.218312927845048 | 2.49263038849323 | 0.0572519083969466 | PSMB5//ENC1//GSK3B//MDM2//HUWE1//FAF1//NEDD4L//PCBP2//RNF216//HECW2//RNF4//TMF1//SIRT6//OPHN1//NEMF// |
| GO:0080090 | regulation of primary metabolic process | Biological process | 107 | 6174 | 262 | 18866 | 1.24794570681781 | 0.00335174735181176 | 0.224445375774896 | 2.47472872508033 | 0.408396946564886 | ACTB//KLF11//CTCF//TCFL5//PRDM5//NEDD4L//IFI16//RBPJ//MDM2//NFATC2//SIRT6//OVOL2//RBL1//BACH2//RPS14//SPI1//NR2C2//NRIP1//KLF7//GTF2IRD1//NCOR2//THBS1//DDX17//DYRK1A//TRA2B//CORO1C//TERF2IP//TFRC//MOB2//NUP155//ZNF483//DNM2//EFEMP1//SETD2//CCAR1//GATAD1//GPBP1L1//ZNF236//TRRAP//CNOT8//ZNF646//ZNF652//LCORL//MEIS1//THAP4//PLAGL1//RFX2//NPAS3//TADA2A//LSM14A//CNOT11//NF1//TAOK3//PSMB5//RPS27L//FAF1//SPAG9//PARD3//BMPR2//LDLR//GAPDH//TMEM59//IGF2BP3//RPL13A//CAPRIN1//ENC1//TIAM2//HUWE1//RNF111//DDX11//SMG1//NAT10//GSK3B//HABP4//PDE4D//RNF4//ARL6IP1//CLK1//ALKBH5//PUM1//AMBRA1//KPNB1//ELOVL6//LARP4//KRT17//HECW2//LATS2//EYA3//CLN6//SCMH1//TRIM24//CRLF3//TAF15//FANK1//TMF1//BRF1//SRPK2//ICAM1//PCBP2//FTO//GNAI2//NEMF//MORC2//OPHN1//CHCHD10//LPXN//MYH9// |
| GO:0001974 | blood vessel remodeling | Biological process | 4 | 45 | 262 | 18866 | 6.40067854113656 | 0.00346410140959664 | 0.224445375774896 | 2.46040940274488 | 0.0152671755725191 | BCR//RBPJ//MDM2//BMPR2// |
| GO:0006412 | translation | Biological process | 20 | 738 | 262 | 18866 | 1.95142638449285 | 0.00349135431232206 | 0.224445375774896 | 2.45700607554039 | 0.0763358778625954 | RPL18A//RPL13A//RPL37A//RPS14//LSM14A//CNOT11//IGF2BP3//GAPDH//CAPRIN1//ENC1//CNOT8//LARP4//KRT17//RPS27L//NAT10//THBS1//HABP4//FTO//PUM1//NEMF// |
| GO:0031323 | regulation of cellular metabolic process | Biological process | 110 | 6387 | 262 | 18866 | 1.24015025750062 | 0.00349575598155548 | 0.224445375774896 | 2.45645889055784 | 0.419847328244275 | ACTB//KLF11//CTCF//TCFL5//PRDM5//NEDD4L//IFI16//RBPJ//MDM2//NFATC2//SIRT6//OVOL2//RBL1//BACH2//RPS14//SPI1//NR2C2//NRIP1//KLF7//GTF2IRD1//NCOR2//THBS1//DDX17//DYRK1A//TRA2B//CORO1C//TERF2IP//TFRC//MOB2//PLCG2//NUP155//ZNF483//DNM2//EFEMP1//SETD2//CCAR1//GATAD1//GPBP1L1//ZNF236//TRRAP//CNOT8//ZNF646//ZNF652//LCORL//MEIS1//THAP4//PLAGL1//RFX2//NPAS3//TADA2A//LSM14A//CNOT11//NF1//TAOK3//PSMB5//RPS27L//FAF1//SPAG9//GPR137B//SMG1//GSK3B//AMBRA1//TMEM59//SIRT5//PARD3//BMPR2//LDLR//GAPDH//SESN1//ATP6V0B//IGF2BP3//RPL13A//CAPRIN1//ENC1//HUWE1//RNF111//DDX11//NAT10//GNAI2//HABP4//PDE4D//RNF4//DDR1//ARL6IP1//CLK1//ALKBH5//PUM1//ICAM1//LARP4//KRT17//LATS2//EYA3//CLN6//SCMH1//TRIM24//CRLF3//TAF15//FANK1//TMF1//BRF1//SRPK2//PCBP2//FTO//NEMF//MORC2//OPHN1//CHCHD10//LPXN//MYH9//BCR// |
| GO:0048856 | anatomical structure development | Biological process | 106 | 6115 | 262 | 18866 | 1.24821081934674 | 0.00353314357454703 | 0.224445375774896 | 2.45183871400399 | 0.404580152671756 | FRYL//ARHGEF28//EVC//SH3PXD2B//PKDCC//SETD2//RBPJ//MEIS1//MYH9//OVOL2//SRPK2//ICAM1//NRIP1//PDE3A//MDM2//BMPR2//NF1//RFX2//TMF1//ACTB//CORO1C//SDCCAG8//NASP//DDX17//GSK3B//ADAM19//THBS1//MIA3//PDS5B//ACP6//ITFG2//PLCG2//SPI1//SLIT3//SIRT6//DNM2//FLNB//TCFL5//DDX11//EYA3//LSM14A//G2E3//DDR1//ENC1//ZMYM4//GTF2IRD1//CFAP44//RNF111//IGF2BP3//DYRK1A//MTR//OPHN1//PPT1//NR2C2//PARD3//KLF7//NFASC//TRIO//PTPRG//BCR//UGP2//NCOR2//FBLIM1//WDR1//SCMH1//HUWE1//TFRC//MOB2//NFATC2//TRAPPC4//STRN//COX6B1//PHLPP2//TRA2B//AMBRA1//HOOK3//LARP4//IFI16//RPS14//KRT17//EFEMP1//TTC39C//NID1//LPXN//AFF3//GTF3C5//LMBR1//DOCK2//DDX6//SPAG9//GPR137B//TAOK3//ANKRD27//NEDD4L//HECW2//LDLR//TIAM2//CAPRIN1//GNB1//PSMB5//PAPPA2//FTO//PDE4D//STARD13//MYO19//DOCK1// |
| GO:0010564 | regulation of cell cycle process | Biological process | 21 | 791 | 262 | 18866 | 1.91170708640141 | 0.0035362921357688 | 0.224445375774896 | 2.45145186478123 | 0.0801526717557252 | MDM2//PLAGL1//CNOT11//CNOT8//TAOK3//SDCCAG8//DYNC1H1//PSMB5//HECW2//RPS27L//SETD2//MYO19//KLHL21//CSPP1//PDE3A//DDX11//GNAI1//CTCF//CRLF3//RNF4//RBL1// |
| GO:0033554 | cellular response to stress | Biological process | 44 | 2100 | 262 | 18866 | 1.50873137041076 | 0.00354838900935008 | 0.224445375774896 | 2.44996877459197 | 0.16793893129771 | ATRIP//POLI//DDX11//PDS5B//SMG1//TAOK3//TRRAP//HUWE1//SIRT6//SETD2//EYA3//ALKBH5//FTO//MORC2//NFATC2//MDM2//PLAGL1//CNOT11//CNOT8//RPS27L//GSK3B//GABARAP//SPAG9//AMBRA1//BMPR2//TERF2IP//CANX//MTR//SESN1//ITFG2//NF1//STAC//THBS1//IFI16//DYRK1A//PSMB5//RBPJ//RNF111//GNB1//ICAM1//RCSD1//DNM2//NUP155//RBL1// |
| GO:0051171 | regulation of nitrogen compound metabolic process | Biological process | 104 | 5980 | 262 | 18866 | 1.25230667109194 | 0.00356111093452166 | 0.224445375774896 | 2.44841449712041 | 0.396946564885496 | ACTB//KLF11//CTCF//TCFL5//PRDM5//NEDD4L//IFI16//RBPJ//MDM2//NFATC2//SIRT6//OVOL2//RBL1//BACH2//RPS14//SPI1//NR2C2//NRIP1//KLF7//GTF2IRD1//NCOR2//THBS1//DDX17//DYRK1A//TRA2B//CORO1C//TERF2IP//TFRC//MOB2//NUP155//ZNF483//DNM2//EFEMP1//SETD2//CCAR1//GATAD1//GPBP1L1//ZNF236//TRRAP//CNOT8//ZNF646//ZNF652//LCORL//MEIS1//THAP4//PLAGL1//RFX2//NPAS3//TADA2A//LSM14A//CNOT11//NF1//TAOK3//PSMB5//RPS27L//FAF1//SPAG9//PARD3//BMPR2//LDLR//GAPDH//TMEM59//IGF2BP3//RPL13A//CAPRIN1//ENC1//HUWE1//RNF111//DDX11//SMG1//NAT10//GSK3B//HABP4//PDE4D//RNF4//ARL6IP1//CLK1//ALKBH5//PUM1//ICAM1//LARP4//KRT17//HECW2//LATS2//EYA3//CLN6//SCMH1//TRIM24//CRLF3//TAF15//FANK1//TMF1//BRF1//SRPK2//PCBP2//FTO//GNAI2//NEMF//MORC2//OPHN1//CHCHD10//LPXN//MYH9//BCR// |
| GO:0048699 | generation of neurons | Biological process | 35 | 1570 | 262 | 18866 | 1.60526571692517 | 0.00361483115447163 | 0.224466976510693 | 2.44191198340231 | 0.133587786259542 | SDCCAG8//NF1//PARD3//ACTB//KLF7//NFASC//OPHN1//SLIT3//TRIO//RBPJ//GSK3B//MOB2//ENC1//MDM2//PTPRG//TRAPPC4//STRN//DNM2//MTR//FRYL//DDX6//MEIS1//SPAG9//PPT1//WDR1//TAOK3//ANKRD27//NEDD4L//HECW2//BMPR2//HOOK3//TIAM2//CAPRIN1//DDR1//LDLR// |
| GO:0036465 | synaptic vesicle recycling | Biological process | 5 | 74 | 262 | 18866 | 4.8653806478234 | 0.00367321510704952 | 0.224466976510693 | 2.43495363820271 | 0.0190839694656489 | DNM2//PIP5K1C//OPHN1//ACTB//CANX// |
| GO:0038202 | TORC1 signaling | Biological process | 4 | 46 | 262 | 18866 | 6.26153335545967 | 0.00375325033095047 | 0.224466976510693 | 2.42559246843403 | 0.0152671755725191 | SESN1//ITFG2//GPR137B//SMG1// |
| GO:0031175 | neuron projection development | Biological process | 25 | 1010 | 262 | 18866 | 1.78236716801451 | 0.00377561135721115 | 0.224466976510693 | 2.4230127162027 | 0.0954198473282443 | PARD3//ACTB//KLF7//NFASC//OPHN1//SLIT3//TRIO//GSK3B//MOB2//ENC1//MDM2//PTPRG//TRAPPC4//STRN//DNM2//MTR//TAOK3//ANKRD27//NEDD4L//HECW2//BMPR2//TIAM2//CAPRIN1//DDR1//FRYL// |
| GO:0050794 | regulation of cellular process | Biological process | 180 | 11433 | 262 | 18866 | 1.13368092764817 | 0.00378071249621242 | 0.224466976510693 | 2.42242634724891 | 0.687022900763359 | ATRIP//ACTB//KLF11//CTCF//TCFL5//PRDM5//NEDD4L//IFI16//RBPJ//MDM2//NFATC2//SIRT6//OVOL2//RBL1//BACH2//RPS14//SPI1//NR2C2//NRIP1//KLF7//GTF2IRD1//NCOR2//NF1//TAOK3//PSMB5//SRPK2//THBS1//DDX17//DYRK1A//TRA2B//PAPPA2//DDR1//ICAM1//CORO1C//TERF2IP//TFRC//MOB2//BMPR2//GSK3B//GNAI2//DNM2//ARL6IP1//PLCG2//MIA3//BCR//NUP155//ZNF483//EFEMP1//SETD2//CCAR1//GATAD1//GPBP1L1//ZNF236//TRRAP//CNOT8//ZNF646//ZNF652//LCORL//MEIS1//THAP4//PLAGL1//RFX2//NPAS3//TADA2A//LSM14A//CNOT11//RPS27L//LPXN//DOCK1//PHLPP2//FLNB//GDI2//GNB1//MCC//OPHN1//PDE3A//PDE4D//PI4KA//RANGAP1//LMBR1//STAC//STARD13//PTPRG//PARD3//TRIO//TIAM2//GNAI1//EVC//MYH9//FAF1//SPAG9//DOCK2//MYO9B//PDS5B//AMBRA1//SLIT3//STRN//TRIM24//AP3S1//ATP6V0B//GABARAP//LATS2//SDCCAG8//DYNC1H1//GPR137B//SMG1//TMEM59//SIRT5//HUWE1//AKAP7//NID1//LDLR//GAPDH//ENC1//PIP5K1C//SESN1//IGF2BP3//RPL13A//CAPRIN1//RPH3AL//KPNB1//KPNA5//HECW2//KRT17//CRLF3//PPT1//ATP8A1//RNF111//TMF1//WDR1//CANX//ITFG2//DDX11//NAT10//PKDCC//MYO19//KLHL21//CSPP1//HABP4//RNF4//FBLIM1//WDFY1//NALCN//ARHGEF28//FAM120B//ANKRD27//WSB1//USP42//FANK1//ZDHHC17//CLK1//ALKBH5//PUM1//GCLM//SH3PXD2B//DDX6//LARP4//EYA3//CLN6//SCMH1//TAF15//BRF1//GOLPH3L//HOOK3//RALGAPA2//PCBP2//IFI30//FTO//RAB11FIP5//CYP24A1//NEMF//MORC2//RNF216//CHCHD10//VDAC3//PDE4DIP//PLEKHM2//UBL5//ADAM19//CD99L2// |
| GO:0030330 | DNA damage response, signal transduction by p53 class mediator | Biological process | 6 | 107 | 262 | 18866 | 4.03781122922166 | 0.00380530487356784 | 0.224466976510693 | 2.41961054267684 | 0.0229007633587786 | MDM2//PLAGL1//CNOT11//CNOT8//RPS27L//DYRK1A// |
| GO:0006907 | pinocytosis | Biological process | 3 | 23 | 262 | 18866 | 9.39230003318951 | 0.00381786896309687 | 0.224466976510693 | 2.41817898154229 | 0.0114503816793893 | DNM2//DOCK2//PPT1// |
| GO:0034063 | stress granule assembly | Biological process | 3 | 23 | 262 | 18866 | 9.39230003318951 | 0.00381786896309687 | 0.224466976510693 | 2.41817898154229 | 0.0114503816793893 | DDX6//DYNC1H1//LSM14A// |
| GO:0042274 | ribosomal small subunit biogenesis | Biological process | 5 | 75 | 262 | 18866 | 4.80050890585242 | 0.00389186178581992 | 0.224466976510693 | 2.40984259150789 | 0.0190839694656489 | RPS27L//RPS14//RCL1//NOL10//NAT10// |
| GO:1901983 | regulation of protein acetylation | Biological process | 5 | 75 | 262 | 18866 | 4.80050890585242 | 0.00389186178581992 | 0.224466976510693 | 2.40984259150789 | 0.0190839694656489 | CTCF//TADA2A//SPI1//GSK3B//TERF2IP// |
| GO:0018193 | peptidyl-amino acid modification | Biological process | 30 | 1291 | 262 | 18866 | 1.67329899894159 | 0.00391123928471671 | 0.224466976510693 | 2.40768561364307 | 0.114503816793893 | PARD3//TRRAP//MDM2//RANGAP1//NUP155//SETD2//TTLL11//CLK1//DYRK1A//SMG1//LATS2//GSK3B//SRPK2//EFEMP1//PKDCC//GALNT1//RPN1//RPN2//PDE4D//TERF2IP//TFRC//RNF4//CTCF//TADA2A//GAPDH//SIRT5//DDR1//SPI1//ICAM1//PRDM5// |
| GO:0044774 | mitotic DNA integrity checkpoint | Biological process | 6 | 108 | 262 | 18866 | 4.00042408821035 | 0.00398336135273204 | 0.225499981978409 | 2.39975029454995 | 0.0229007633587786 | MDM2//PLAGL1//CNOT11//CNOT8//TAOK3//RPS27L// |
| GO:0043604 | amide biosynthetic process | Biological process | 23 | 906 | 262 | 18866 | 1.82800835818883 | 0.00399475054594112 | 0.225499981978409 | 2.39851033522452 | 0.0877862595419847 | RPL18A//IGF2BP3//LARP4//RPL13A//RPS27L//RPL37A//RPS14//LSM14A//CNOT11//GCLM//GAPDH//CAPRIN1//ENC1//CNOT8//ELOVL6//KRT17//NAT10//THBS1//HABP4//PPT1//FTO//PUM1//NEMF// |
| GO:0032989 | cellular component morphogenesis | Biological process | 21 | 800 | 262 | 18866 | 1.89020038167939 | 0.00402507405146332 | 0.225499981978409 | 2.39522612527225 | 0.0801526717557252 | RFX2//TMF1//PARD3//ACTB//KLF7//NFASC//OPHN1//SLIT3//TRIO//DNM2//GSK3B//WDR1//TAOK3//ANKRD27//NEDD4L//HECW2//BMPR2//TIAM2//CAPRIN1//DDR1//CORO1C// |
| GO:0003281 | ventricular septum development | Biological process | 5 | 76 | 262 | 18866 | 4.73734431498594 | 0.00411966773714426 | 0.228982161862215 | 2.38513780963282 | 0.0190839694656489 | RBPJ//SLIT3//BMPR2//DNM2//MDM2// |
| GO:0090630 | activation of GTPase activity | Biological process | 6 | 109 | 262 | 18866 | 3.9637229497864 | 0.00416751336014456 | 0.229831850072347 | 2.38012299924375 | 0.0229007633587786 | TBC1D1//CORO1C//TIAM2//RALGAPA2//RANGAP1//BCR// |
| GO:0072331 | signal transduction by p53 class mediator | Biological process | 10 | 267 | 262 | 18866 | 2.69691511564743 | 0.00428109805911994 | 0.234265668211842 | 2.36844482447894 | 0.0381679389312977 | MDM2//PLAGL1//CNOT11//CNOT8//RPS27L//IFI16//DYRK1A//TAF15//ATRIP//TRIM24// |
| GO:0003181 | atrioventricular valve morphogenesis | Biological process | 3 | 24 | 262 | 18866 | 9.00095419847328 | 0.00431894630880174 | 0.234518784567934 | 2.36462219487264 | 0.0114503816793893 | BMPR2//MDM2//SLIT3// |
| GO:0009896 | positive regulation of catabolic process | Biological process | 14 | 454 | 262 | 18866 | 2.22049971416081 | 0.00463830281889913 | 0.249937248844343 | 2.33364090117371 | 0.0534351145038168 | GSK3B//AMBRA1//TMEM59//SESN1//MDM2//HUWE1//NEDD4L//HECW2//CNOT8//FTO//PUM1//SIRT6//FAF1//LDLR// |
| GO:0044085 | cellular component biogenesis | Biological process | 62 | 3257 | 262 | 18866 | 1.37073174161583 | 0.00485021417708644 | 0.251510753128841 | 2.31423908327958 | 0.236641221374046 | RPS27L//RPS14//GABARAP//AMBRA1//NAT10//SRPK2//RCL1//NOL10//RFX2//TMF1//DNM2//NASP//DDX17//TEX10//OPHN1//TUBGCP2//TBCEL//CFAP44//CTCF//ICAM1//NFASC//DYRK1A//GSK3B//FAF1//TFRC//NDUFB2//FBLIM1//DDX6//DYNC1H1//LSM14A//ACTB//PIP5K1C//TTC19//PDE4DIP//ANKRD27//DYNLRB1//WDR1//BCR//TRAPPC4//STARD9//TMEM120B//SH3PXD2B//CORO1C//THBS1//RABL2B//TTC39C//CEP83//CHCHD10//MDM2//PARD3//LPXN//STRN//BRF1//ARL6IP1//RNF4//KPNB1//MORC2//SDCCAG8//RPL13A//VDAC3//TRRAP//SIRT6// |
| GO:0033043 | regulation of organelle organization | Biological process | 30 | 1311 | 262 | 18866 | 1.64777193564728 | 0.00486517572465584 | 0.251510753128841 | 2.31290146888584 | 0.114503816793893 | HUWE1//TFRC//GSK3B//HECW2//WDR1//ICAM1//CTCF//DYRK1A//SIRT6//SMG1//TERF2IP//NAT10//TADA2A//ANKRD27//PDE3A//DDX11//SH3PXD2B//DYNC1H1//GNAI1//MYO19//RNF4//SPI1//MORC2//CHCHD10//SDCCAG8//VDAC3//DNM2//PDE4DIP//ARL6IP1//UBL5// |
| GO:0034655 | nucleobase-containing compound catabolic process | Biological process | 16 | 555 | 262 | 18866 | 2.07589574307132 | 0.00487393151317119 | 0.251510753128841 | 2.31212057766685 | 0.0610687022900763 | SMG1//RPL13A//RPL18A//RPL37A//RPS14//CNOT11//CNOT8//PDE4D//KPNB1//IGF2BP3//ALKBH5//PSMB5//PUM1//DDX6//TAF15//FTO// |
| GO:0010558 | negative regulation of macromolecule biosynthetic process | Biological process | 36 | 1659 | 262 | 18866 | 1.56255262758307 | 0.00488287631686771 | 0.251510753128841 | 2.31132427624468 | 0.137404580152672 | CTCF//TCFL5//PRDM5//NEDD4L//IFI16//RBPJ//MDM2//NFATC2//SIRT6//OVOL2//RBL1//BACH2//RPS14//SPI1//NR2C2//NRIP1//KLF11//KLF7//GTF2IRD1//NCOR2//IGF2BP3//RPL13A//GAPDH//CAPRIN1//ENC1//CNOT8//NAT10//GSK3B//SCMH1//TRIM24//FTO//PUM1//TMEM59//MORC2//CCAR1//BMPR2// |
| GO:2000113 | negative regulation of cellular macromolecule biosynthetic process | Biological process | 35 | 1602 | 262 | 18866 | 1.57320048412766 | 0.0049621403019542 | 0.251510753128841 | 2.30433096043804 | 0.133587786259542 | CTCF//TCFL5//PRDM5//NEDD4L//IFI16//RBPJ//MDM2//NFATC2//SIRT6//OVOL2//RBL1//BACH2//RPS14//SPI1//NR2C2//NRIP1//KLF11//KLF7//GTF2IRD1//NCOR2//IGF2BP3//RPL13A//GAPDH//CAPRIN1//ENC1//CNOT8//NAT10//GSK3B//SCMH1//TRIM24//FTO//PUM1//TMEM59//MORC2//BMPR2// |
| GO:0006406 | mRNA export from nucleus | Biological process | 6 | 113 | 262 | 18866 | 3.82341417280281 | 0.00496755391542192 | 0.251510753128841 | 2.30385741057081 | 0.0229007633587786 | SETD2//SMG1//CDC40//ALKBH5//FIP1L1//NUP155// |
| GO:0071427 | mRNA-containing ribonucleoprotein complex export from nucleus | Biological process | 6 | 113 | 262 | 18866 | 3.82341417280281 | 0.00496755391542192 | 0.251510753128841 | 2.30385741057081 | 0.0229007633587786 | SMG1//CDC40//ALKBH5//FIP1L1//NUP155//SETD2// |
| GO:0044271 | cellular nitrogen compound biosynthetic process | Biological process | 88 | 4960 | 262 | 18866 | 1.27755478946072 | 0.00500175864531364 | 0.251510753128841 | 2.30087726853063 | 0.33587786259542 | KLF11//CTCF//TCFL5//PRDM5//NEDD4L//IFI16//RBPJ//MDM2//NFATC2//SIRT6//OVOL2//RBL1//BACH2//RPS14//SPI1//NR2C2//NRIP1//KLF7//GTF2IRD1//NCOR2//RPL18A//UGP2//PDE4D//DCTD//GPBP1L1//CNOT8//BRF1//ZNF483//DNM2//EFEMP1//SETD2//TERF2IP//CCAR1//GATAD1//ZNF236//TRRAP//ZNF646//DDX17//ZNF652//LCORL//MEIS1//THAP4//PLAGL1//RFX2//NPAS3//TADA2A//PHRF1//TAF15//TRIM24//FIP1L1//GTF3C5//IGF2BP3//LARP4//RPL13A//RPS27L//RPL37A//LSM14A//CNOT11//GCLM//FAF1//GAPDH//CAPRIN1//ENC1//POLI//NAT10//ELOVL6//ICAM1//KRT17//THBS1//SCMH1//DYRK1A//CRLF3//RNF111//RNF4//FANK1//BMPR2//TMF1//HABP4//PPT1//TFRC//FTO//PUM1//PSMB5//NEMF//MORC2//DDX11//CHCHD10//LPXN// |
| GO:0009991 | response to extracellular stimulus | Biological process | 15 | 507 | 262 | 18866 | 2.13040336058539 | 0.00501709973282187 | 0.251510753128841 | 2.29954726572854 | 0.0572519083969466 | GABARAP//GCLM//GNAI2//AMBRA1//BMPR2//SIRT5//SIRT6//ICAM1//CYP24A1//SESN1//ITFG2//IFI16//LDLR//TRIM24//MDM2// |
| GO:0008152 | metabolic process | Biological process | 196 | 12706 | 262 | 18866 | 1.11077413688069 | 0.00502380169870613 | 0.251510753128841 | 2.29896751155935 | 0.748091603053435 | GABARAP//AMBRA1//ACTB//KLF11//MTR//CTCF//TCFL5//PRDM5//NEDD4L//IFI16//RBPJ//MDM2//NFATC2//SIRT6//OVOL2//RBL1//BACH2//RPS14//SPI1//NR2C2//NRIP1//KLF7//GTF2IRD1//NCOR2//NAT10//NF1//TAOK3//PSMB5//SRPK2//SMG1//RPL13A//RPL18A//RPL37A//THBS1//HUWE1//WSB1//RNF111//HECW2//UBE3B//CNOT11//CNOT8//TRA2B//DDX17//DYRK1A//PCBP2//CDC40//CCAR1//UBL5//FIP1L1//RCL1//NOL10//TERF2IP//CLN6//TMF1//CORO1C//TFRC//MOB2//DNM2//PPT1//PLCG2//MTO1//GALNT7//PHKG1//SIAE//SLC3A2//GSK3B//UGP2//HK1//COQ2//GAPDH//MDH1//NUP155//CHCHD10//NDUFB2//COX6B1//SLC23A2//PDE4D//DCTD//POLI//NASP//ATRIP//DDX11//PDS5B//TRRAP//SETD2//EYA3//ALKBH5//FTO//KPNB1//GPBP1L1//BRF1//ZNF483//EFEMP1//GATAD1//ZNF236//ZNF646//ZNF652//LCORL//MEIS1//THAP4//PLAGL1//RFX2//NPAS3//TADA2A//TEX10//PHRF1//TAF15//TRIM24//GTF3C5//HABP4//IGF2BP3//LARP4//RPS27L//LSM14A//RPN1//RPN2//LATS2//BCR//BMPR2//TRIO//NEK9//PHLPP2//PTPRG//SIRT5//GALNT1//PITRM1//PRSS57//DPP8//CFAP44//PAPPA2//TMEM59//MYH9//ADAM19//USP42//GCLM//PITPNB//LDLR//PDE3A//MORC2//ABCD4//ELOVL6//ACP6//PIP5K1C//PI4KA//AHCYL2//CYP24A1//PCCB//SH3PXD2B//TRAPPC4//GPR137B//FAF1//SPAG9//RPPH1//MBOAT2//BCKDHA//AFF3//ENC1//PUM1//SLIT3//PARD3//SESN1//ATP6V0B//SCMH1//G2E3//DCAF17//KLHL21//RANGAP1//CAPRIN1//TTLL11//CLK1//PKDCC//ZDHHC17//TIAM2//CRAT//SMYD4//RNF216//GNAI2//RNF4//DDR1//SAGE1//OSBPL10//HBG2//ARL6IP1//MIA3//RAB2A//DDX6//ICAM1//KRT17//CRLF3//FANK1//IFI30//TTC19//NEMF//OPHN1//DYNC1H1//LPXN// |
| GO:0051172 | negative regulation of nitrogen compound metabolic process | Biological process | 51 | 2572 | 262 | 18866 | 1.42783410302376 | 0.00509503061393926 | 0.253280430308431 | 2.29285320215315 | 0.194656488549618 | CTCF//TCFL5//PRDM5//NEDD4L//IFI16//RBPJ//MDM2//NFATC2//SIRT6//OVOL2//RBL1//BACH2//RPS14//SPI1//NR2C2//NRIP1//KLF11//KLF7//GTF2IRD1//NCOR2//CORO1C//TERF2IP//NF1//TAOK3//THBS1//PARD3//GAPDH//TMEM59//IGF2BP3//RPL13A//CAPRIN1//ENC1//CNOT8//NAT10//GSK3B//PDE4D//RNF4//ARL6IP1//LATS2//SCMH1//TRIM24//DYRK1A//TAF15//LDLR//FTO//PUM1//MORC2//OPHN1//CCAR1//GNAI2//BMPR2// |
| GO:0007264 | small GTPase mediated signal transduction | Biological process | 15 | 509 | 262 | 18866 | 2.12203242400156 | 0.0051981976504504 | 0.253958738548676 | 2.28414721139368 | 0.0572519083969466 | GNB1//NF1//MYO9B//KPNB1//DNM2//OPHN1//BCR//ARHGEF28//STARD13//TIAM2//GDI2//RALGAPA2//TRIO//DOCK1//DOCK2// |
| GO:0010631 | epithelial cell migration | Biological process | 12 | 365 | 262 | 18866 | 2.36737425494092 | 0.00520022555377734 | 0.253958738548676 | 2.28397781893511 | 0.0458015267175573 | MIA3//BMPR2//THBS1//CORO1C//MCC//PTPRG//DOCK1//PLCG2//MYH9//NF1//STARD13//LPXN// |
| GO:0000077 | DNA damage checkpoint | Biological process | 7 | 151 | 262 | 18866 | 3.33810221930135 | 0.00521660533922057 | 0.253958738548676 | 2.28261201845917 | 0.0267175572519084 | MDM2//PLAGL1//CNOT11//CNOT8//TAOK3//RPS27L//ATRIP// |
| GO:0071158 | positive regulation of cell cycle arrest | Biological process | 5 | 81 | 262 | 18866 | 4.44491565356705 | 0.00540332717120888 | 0.256283844913949 | 2.26733873515397 | 0.0190839694656489 | MDM2//PLAGL1//CNOT11//CNOT8//CRLF3// |
| GO:0003171 | atrioventricular valve development | Biological process | 3 | 26 | 262 | 18866 | 8.30857310628303 | 0.00543600233747703 | 0.256283844913949 | 2.26472036519319 | 0.0114503816793893 | MDM2//SLIT3//BMPR2// |
| GO:0043951 | negative regulation of cAMP-mediated signaling | Biological process | 3 | 26 | 262 | 18866 | 8.30857310628303 | 0.00543600233747703 | 0.256283844913949 | 2.26472036519319 | 0.0114503816793893 | GNAI2//PDE3A//PDE4D// |
| GO:0044068 | modulation by symbiont of host cellular process | Biological process | 3 | 26 | 262 | 18866 | 8.30857310628303 | 0.00543600233747703 | 0.256283844913949 | 2.26472036519319 | 0.0114503816793893 | KPNB1//KPNA5//GAPDH// |
| GO:0030490 | maturation of SSU-rRNA | Biological process | 4 | 51 | 262 | 18866 | 5.64765753629696 | 0.0054458955570325 | 0.256283844913949 | 2.26393069194196 | 0.0152671755725191 | RCL1//RPS14//NOL10//NAT10// |
| GO:0034504 | protein localization to nucleus | Biological process | 10 | 277 | 262 | 18866 | 2.59955355912586 | 0.00552143505407286 | 0.257334482771045 | 2.25794803186017 | 0.0381679389312977 | KPNB1//RANBP17//NUP155//KPNA5//NF1//RANGAP1//CHCHD10//GSK3B//TFRC//MDM2// |
| GO:0090132 | epithelium migration | Biological process | 12 | 368 | 262 | 18866 | 2.34807500829738 | 0.00554113066740315 | 0.257334482771045 | 2.25640160846434 | 0.0458015267175573 | MIA3//BMPR2//THBS1//CORO1C//MCC//PTPRG//DOCK1//PLCG2//MYH9//NF1//STARD13//LPXN// |
| GO:0001678 | cellular glucose homeostasis | Biological process | 7 | 153 | 262 | 18866 | 3.29446689617323 | 0.00559927186844205 | 0.258335033459689 | 2.25186844515054 | 0.0267175572519084 | RAB11FIP5//KLF7//GCLM//ICAM1//TRA2B//ZNF236//HK1// |
| GO:1903828 | negative regulation of cellular protein localization | Biological process | 6 | 116 | 262 | 18866 | 3.72453277178205 | 0.00563766253940655 | 0.258417271855005 | 2.24890092379252 | 0.0229007633587786 | NF1//PKDCC//RANGAP1//GSK3B//TMEM59//NEDD4L// |
| GO:0051056 | regulation of small GTPase mediated signal transduction | Biological process | 11 | 323 | 262 | 18866 | 2.45227235128684 | 0.00567770932854141 | 0.258573871936605 | 2.24582684503345 | 0.0419847328244275 | DNM2//NF1//MYO9B//OPHN1//BCR//ARHGEF28//STARD13//TIAM2//GDI2//RALGAPA2//TRIO// |
| GO:0051345 | positive regulation of hydrolase activity | Biological process | 20 | 773 | 262 | 18866 | 1.86306943306045 | 0.00579607865405864 | 0.262272559096153 | 2.2368657296895 | 0.0763358778625954 | RPS27L//DDX11//AGAP1//DOCK1//DOCK2//GDI2//GSK3B//ICAM1//MYO9B//NF1//OPHN1//ANKRD27//STARD13//TBC1D1//CORO1C//TIAM2//RALGAPA2//RANGAP1//BCR//IFI16// |
| GO:0051130 | positive regulation of cellular component organization | Biological process | 28 | 1217 | 262 | 18866 | 1.65670808583239 | 0.0060248527479276 | 0.27088812450714 | 2.22005356311724 | 0.106870229007634 | PLCG2//DNM2//GSK3B//MOB2//ENC1//WDR1//ICAM1//FAF1//TFRC//SIRT6//TADA2A//NEDD4L//DDX11//PPT1//BMPR2//TIAM2//CAPRIN1//ANKRD27//SH3PXD2B//ATP8A1//MORC2//HUWE1//CHCHD10//NFATC2//PLEKHM2//UBL5//DYNC1H1//CORO1C// |
| GO:0071496 | cellular response to external stimulus | Biological process | 11 | 326 | 262 | 18866 | 2.42970542780874 | 0.00607273454835541 | 0.271312868207853 | 2.21661570236238 | 0.0419847328244275 | GABARAP//AMBRA1//BMPR2//ICAM1//SESN1//ITFG2//IFI16//CYP24A1//TRIM24//HABP4//MDM2// |
| GO:0045935 | positive regulation of nucleobase-containing compound metabolic process | Biological process | 39 | 1863 | 262 | 18866 | 1.50740617816622 | 0.00617910481619913 | 0.273480968273427 | 2.20907443778254 | 0.148854961832061 | RBPJ//DDX11//SIRT6//DYRK1A//HABP4//EYA3//TFRC//CTCF//DNM2//NFATC2//CRLF3//RNF111//GPBP1L1//RNF4//NPAS3//SPI1//TAF15//TRIM24//FANK1//DDX17//IFI16//MEIS1//PLAGL1//OVOL2//RBL1//RFX2//BMPR2//TMF1//NR2C2//NRIP1//KLF7//BRF1//TRA2B//CNOT8//FTO//PUM1//CHCHD10//CCAR1//TADA2A// |
| GO:1901701 | cellular response to oxygen-containing compound | Biological process | 28 | 1220 | 262 | 18866 | 1.65263421349018 | 0.00622391799993591 | 0.273480968273427 | 2.2059361378066 | 0.106870229007634 | GNB1//GSK3B//GNAI2//AP3S1//ATP6V0B//RAB11FIP5//KLF7//MDM2//CYP24A1//TRIM24//ICAM1//PDE4D//BCR//SESN1//DNM2//PDE3A//GCLM//TRA2B//ZNF236//SPI1//NRIP1//LDLR//MTR//ACTB//KLF11//RANGAP1//GNAI1//DDX11// |
| GO:0043949 | regulation of cAMP-mediated signaling | Biological process | 4 | 53 | 262 | 18866 | 5.43453838398387 | 0.00624535872838802 | 0.273480968273427 | 2.20444261104725 | 0.0152671755725191 | PDE3A//PDE4D//GNAI2//GNAI1// |
| GO:0090130 | tissue migration | Biological process | 12 | 374 | 262 | 18866 | 2.31040535575785 | 0.00627623131609224 | 0.273480968273427 | 2.20230105848039 | 0.0458015267175573 | MIA3//BMPR2//THBS1//CORO1C//MCC//PTPRG//DOCK1//PLCG2//MYH9//NF1//STARD13//LPXN// |
| GO:0000278 | mitotic cell cycle | Biological process | 25 | 1053 | 262 | 18866 | 1.70958294367964 | 0.00636360492293566 | 0.275116344809058 | 2.19629679086 | 0.0954198473282443 | LATS2//NASP//CRLF3//KLF11//SDCCAG8//DYNC1H1//DNM2//TTC19//MDM2//PLAGL1//CNOT11//CNOT8//PDS5B//NEK9//KPNB1//TAOK3//RBL1//PSMB5//HECW2//RPS27L//GNAI1//SETD2//TUBA1B//TUBGCP2//PRDM5// |
| GO:0009749 | response to glucose | Biological process | 8 | 197 | 262 | 18866 | 2.92416786143294 | 0.00639170995164833 | 0.275116344809058 | 2.19438294102458 | 0.0305343511450382 | RAB11FIP5//KLF7//GCLM//ICAM1//TRA2B//ZNF236//COL6A2//THBS1// |
| GO:0051254 | positive regulation of RNA metabolic process | Biological process | 36 | 1692 | 262 | 18866 | 1.53207731037843 | 0.00663721011677779 | 0.283951916450512 | 2.17801443350867 | 0.137404580152672 | RBPJ//DYRK1A//HABP4//CTCF//DNM2//NFATC2//CRLF3//RNF111//GPBP1L1//RNF4//NPAS3//SPI1//TAF15//TRIM24//FANK1//DDX17//IFI16//MEIS1//PLAGL1//OVOL2//RBL1//RFX2//BMPR2//TMF1//NR2C2//NRIP1//KLF7//BRF1//TRA2B//CNOT8//FTO//PUM1//DDX11//CHCHD10//CCAR1//TADA2A// |
| GO:0051726 | regulation of cell cycle | Biological process | 28 | 1227 | 262 | 18866 | 1.64320598244337 | 0.00670985869599277 | 0.285330677921765 | 2.17328662561294 | 0.106870229007634 | ATRIP//ACTB//MDM2//PLAGL1//CNOT11//CNOT8//THBS1//TAOK3//RBL1//SDCCAG8//DYNC1H1//PSMB5//HECW2//RPS27L//SETD2//MYO19//KLHL21//CSPP1//PDE3A//LATS2//SRPK2//DDX11//GNAI1//CTCF//CRLF3//RNF4//BCR//PUM1// |
| GO:0006915 | apoptotic process | Biological process | 41 | 1996 | 262 | 18866 | 1.47911471798559 | 0.00687042242445504 | 0.286954936103349 | 2.16301655974251 | 0.156488549618321 | KPNB1//RPS27L//GABARAP//NF1//GCLM//GNB1//SIRT5//AMBRA1//IFI16//USP42//TRIM24//FANK1//FAF1//DNM2//TIAM2//LATS2//CCAR1//TRIO//KLF11//ARL6IP1//GSK3B//PDE3A//PPT1//TFRC//THBS1//TMF1//MDM2//SRPK2//GAPDH//SLIT3//BMPR2//CHCHD10//ICAM1//SPI1//GNAI2//EYA3//DOCK1//PLAGL1//RNF216//DPP8//G2E3// |
| GO:0035023 | regulation of Rho protein signal transduction | Biological process | 5 | 86 | 262 | 18866 | 4.18649032487129 | 0.00694671709833994 | 0.286954936103349 | 2.15822038719612 | 0.0190839694656489 | MYO9B//OPHN1//BCR//ARHGEF28//STARD13// |
| GO:0050657 | nucleic acid transport | Biological process | 8 | 200 | 262 | 18866 | 2.88030534351145 | 0.00697915999803988 | 0.286954936103349 | 2.15619684530937 | 0.0305343511450382 | CDC40//NUP155//SMG1//ALKBH5//FIP1L1//SETD2//IGF2BP3//RANBP17// |
| GO:0050658 | RNA transport | Biological process | 8 | 200 | 262 | 18866 | 2.88030534351145 | 0.00697915999803988 | 0.286954936103349 | 2.15619684530937 | 0.0305343511450382 | CDC40//NUP155//SMG1//ALKBH5//FIP1L1//SETD2//IGF2BP3//RANBP17// |
| GO:0032880 | regulation of protein localization | Biological process | 23 | 950 | 262 | 18866 | 1.74334270791483 | 0.00699115018123347 | 0.286954936103349 | 2.15545136842164 | 0.0877862595419847 | SETD2//RPH3AL//NF1//PKDCC//RANGAP1//GSK3B//MDM2//GOLPH3L//GAPDH//KLF7//RAB11FIP5//OPHN1//GPR137B//TFRC//ACTB//TMEM59//STAC//HUWE1//UBL5//GNAI1//NEDD4L//PLEKHM2//TBC1D1// |
| GO:0000082 | G1/S transition of mitotic cell cycle | Biological process | 10 | 287 | 262 | 18866 | 2.50897678006224 | 0.00702686039565677 | 0.286954936103349 | 2.15323867461109 | 0.0381679389312977 | KLF11//MDM2//PLAGL1//CNOT11//CNOT8//RPS27L//RBL1//LATS2//NASP//CRLF3// |
| GO:0120039 | plasma membrane bounded cell projection morphogenesis | Biological process | 18 | 681 | 262 | 18866 | 1.90328546928069 | 0.00703261141038098 | 0.286954936103349 | 2.15288337903046 | 0.0687022900763359 | PARD3//ACTB//KLF7//NFASC//OPHN1//SLIT3//TRIO//DNM2//GSK3B//TAOK3//ANKRD27//NEDD4L//HECW2//BMPR2//TIAM2//CAPRIN1//DDR1//CORO1C// |
| GO:0071407 | cellular response to organic cyclic compound | Biological process | 16 | 580 | 262 | 18866 | 1.98641747828376 | 0.00733325433538914 | 0.292732114345376 | 2.13470325233692 | 0.0610687022900763 | GNB1//GSK3B//DDX17//TMF1//NCOR2//CYP24A1//TRIM24//MDM2//PDE3A//NRIP1//ICAM1//PDE4D//ACTB//GCLM//DNM2//GNAI1// |
| GO:0031570 | DNA integrity checkpoint | Biological process | 7 | 161 | 262 | 18866 | 3.13076667772984 | 0.00734314541476634 | 0.292732114345376 | 2.13411787146462 | 0.0267175572519084 | ATRIP//MDM2//PLAGL1//CNOT11//CNOT8//TAOK3//RPS27L// |
| GO:0009746 | response to hexose | Biological process | 8 | 202 | 262 | 18866 | 2.85178746882322 | 0.00739288035097302 | 0.292732114345376 | 2.13118632252326 | 0.0305343511450382 | COL6A2//THBS1//RAB11FIP5//KLF7//GCLM//ICAM1//TRA2B//ZNF236// |
| GO:0001773 | myeloid dendritic cell activation | Biological process | 3 | 29 | 262 | 18866 | 7.4490655435641 | 0.00740980380789369 | 0.292732114345376 | 2.1301932908435 | 0.0114503816793893 | DOCK2//RBPJ//SPI1// |
| GO:0042593 | glucose homeostasis | Biological process | 9 | 245 | 262 | 18866 | 2.64517837669419 | 0.00743895627094768 | 0.292732114345376 | 2.12848799423448 | 0.0343511450381679 | HK1//RAB11FIP5//KLF7//GCLM//ICAM1//TRA2B//ZNF236//SIRT6//RPH3AL// |
| GO:0048858 | cell projection morphogenesis | Biological process | 18 | 685 | 262 | 18866 | 1.89217139354767 | 0.00744976462300621 | 0.292732114345376 | 2.12785744866707 | 0.0687022900763359 | PARD3//ACTB//KLF7//NFASC//OPHN1//SLIT3//TRIO//DNM2//GSK3B//TAOK3//ANKRD27//NEDD4L//HECW2//BMPR2//TIAM2//CAPRIN1//DDR1//CORO1C// |
| GO:0060341 | regulation of cellular localization | Biological process | 24 | 1012 | 262 | 18866 | 1.70769091512537 | 0.00755560264618656 | 0.292732114345376 | 2.1217308899649 | 0.0916030534351145 | SETD2//PDE4D//DYNC1H1//NF1//PKDCC//BCR//RANGAP1//GSK3B//MDM2//PLCG2//WDR1//KLF7//OPHN1//GPR137B//TFRC//ACTB//TMEM59//STAC//HUWE1//UBL5//GNAI1//RAB11FIP5//NEDD4L//PARD3// |
| GO:0006977 | DNA damage response, signal transduction by p53 class mediator resulting in cell cycle arrest | Biological process | 4 | 56 | 262 | 18866 | 5.14340239912759 | 0.00758481143447048 | 0.292732114345376 | 2.12005521171138 | 0.0152671755725191 | MDM2//PLAGL1//CNOT11//CNOT8// |
| GO:2001236 | regulation of extrinsic apoptotic signaling pathway | Biological process | 7 | 162 | 262 | 18866 | 3.11144095749694 | 0.00758649186815129 | 0.292732114345376 | 2.11995900335496 | 0.0267175572519084 | GSK3B//ICAM1//FAF1//THBS1//GCLM//EYA3//NF1// |
| GO:0051236 | establishment of RNA localization | Biological process | 8 | 203 | 262 | 18866 | 2.83773925469108 | 0.00760654806253634 | 0.292732114345376 | 2.11881238626598 | 0.0305343511450382 | CDC40//NUP155//SMG1//ALKBH5//FIP1L1//SETD2//IGF2BP3//RANBP17// |
| GO:0033500 | carbohydrate homeostasis | Biological process | 9 | 246 | 262 | 18866 | 2.63442561906535 | 0.00763065208295668 | 0.292732114345376 | 2.11743834750657 | 0.0343511450381679 | HK1//RAB11FIP5//SIRT6//KLF7//RPH3AL//GCLM//ICAM1//TRA2B//ZNF236// |
| GO:0044257 | cellular protein catabolic process | Biological process | 20 | 795 | 262 | 18866 | 1.81151279466129 | 0.00780094770891976 | 0.292732114345376 | 2.10785263323871 | 0.0763358778625954 | HUWE1//NEDD4L//MDM2//RNF111//HECW2//USP42//UBE3B//PSMB5//ENC1//GSK3B//FAF1//PCBP2//RNF216//RNF4//TMF1//SIRT6//OPHN1//LDLR//NEMF//PPT1// |
| GO:1903047 | mitotic cell cycle process | Biological process | 22 | 905 | 262 | 18866 | 1.75046181097381 | 0.00790384162682444 | 0.292732114345376 | 2.10216177050612 | 0.083969465648855 | LATS2//NASP//CRLF3//KLF11//SDCCAG8//DYNC1H1//DNM2//TTC19//MDM2//PLAGL1//CNOT11//CNOT8//PDS5B//NEK9//KPNB1//TAOK3//PSMB5//HECW2//RPS27L//GNAI1//SETD2//RBL1// |
| GO:0072594 | establishment of protein localization to organelle | Biological process | 16 | 585 | 262 | 18866 | 1.96943955111894 | 0.0079281775599676 | 0.292732114345376 | 2.10082663192709 | 0.0610687022900763 | KPNB1//RANBP17//NUP155//KPNA5//RPL13A//RPL18A//RPL37A//RPS14//CRAT//PITRM1//NF1//HK1//DNM2//OPHN1//HUWE1//UBL5// |
| GO:0022607 | cellular component assembly | Biological process | 57 | 3012 | 262 | 18866 | 1.36269426112344 | 0.00800856367937159 | 0.292732114345376 | 2.09644536681929 | 0.217557251908397 | RPS27L//RPS14//GABARAP//AMBRA1//SRPK2//RFX2//TMF1//DNM2//NASP//OPHN1//TUBGCP2//TBCEL//CFAP44//CTCF//ICAM1//NFASC//DYRK1A//GSK3B//FAF1//TFRC//NDUFB2//FBLIM1//DDX6//DYNC1H1//LSM14A//ACTB//PIP5K1C//TTC19//PDE4DIP//ANKRD27//DYNLRB1//WDR1//BCR//TRAPPC4//STARD9//TMEM120B//SH3PXD2B//CORO1C//THBS1//RABL2B//TTC39C//CEP83//CHCHD10//MDM2//PARD3//LPXN//STRN//BRF1//ARL6IP1//RNF4//KPNB1//MORC2//SDCCAG8//RPL13A//VDAC3//TRRAP//SIRT6// |
| GO:0006307 | DNA dealkylation involved in DNA repair | Biological process | 2 | 10 | 262 | 18866 | 14.4015267175573 | 0.00803290251541966 | 0.292732114345376 | 2.09512750345514 | 0.00763358778625954 | ALKBH5//FTO// |
| GO:0015851 | nucleobase transport | Biological process | 2 | 10 | 262 | 18866 | 14.4015267175573 | 0.00803290251541966 | 0.292732114345376 | 2.09512750345514 | 0.00763358778625954 | VDAC3//SLC23A2// |
| GO:0044351 | macropinocytosis | Biological process | 2 | 10 | 262 | 18866 | 14.4015267175573 | 0.00803290251541966 | 0.292732114345376 | 2.09512750345514 | 0.00763358778625954 | DNM2//DOCK2// |
| GO:0044380 | protein localization to cytoskeleton | Biological process | 4 | 57 | 262 | 18866 | 5.05316726931833 | 0.00807006797880431 | 0.292732114345376 | 2.09312280695138 | 0.0152671755725191 | KLHL21//CEP83//HOOK3//GSK3B// |
| GO:0072431 | signal transduction involved in mitotic G1 DNA damage checkpoint | Biological process | 4 | 57 | 262 | 18866 | 5.05316726931833 | 0.00807006797880431 | 0.292732114345376 | 2.09312280695138 | 0.0152671755725191 | MDM2//PLAGL1//CNOT11//CNOT8// |
| GO:1902400 | intracellular signal transduction involved in G1 DNA damage checkpoint | Biological process | 4 | 57 | 262 | 18866 | 5.05316726931833 | 0.00807006797880431 | 0.292732114345376 | 2.09312280695138 | 0.0152671755725191 | MDM2//PLAGL1//CNOT11//CNOT8// |
| GO:0031328 | positive regulation of cellular biosynthetic process | Biological process | 40 | 1955 | 262 | 18866 | 1.47330196599051 | 0.00808652249572861 | 0.292732114345376 | 2.09223820095922 | 0.152671755725191 | RBPJ//LDLR//DNM2//ICAM1//LARP4//KRT17//RPS27L//NAT10//THBS1//FAF1//CTCF//DYRK1A//NFATC2//CRLF3//RNF111//GPBP1L1//RNF4//NPAS3//SPI1//TAF15//TRIM24//FANK1//DDX17//IFI16//MEIS1//PLAGL1//OVOL2//RBL1//RFX2//BMPR2//TMF1//NR2C2//NRIP1//KLF7//BRF1//HABP4//DDX11//CHCHD10//CCAR1//TADA2A// |
| GO:0012501 | programmed cell death | Biological process | 43 | 2139 | 262 | 18866 | 1.44755878647724 | 0.00815187555958011 | 0.293592293750388 | 2.08874245856495 | 0.16412213740458 | KPNB1//FAF1//DOCK1//ARL6IP1//PLAGL1//RNF216//DPP8//G2E3//CCAR1//KLF11//RPS27L//GABARAP//NF1//GCLM//GNB1//SIRT5//AMBRA1//IFI16//USP42//TRIM24//FANK1//DNM2//TIAM2//LATS2//TRIO//GSK3B//PDE3A//PPT1//TFRC//THBS1//TMF1//PLCG2//MDM2//SRPK2//GAPDH//SLIT3//KRT17//BMPR2//CHCHD10//ICAM1//SPI1//GNAI2//EYA3// |
| GO:0006518 | peptide metabolic process | Biological process | 22 | 909 | 262 | 18866 | 1.7427590087253 | 0.00829887607677067 | 0.297369371705199 | 2.0809807204803 | 0.083969465648855 | RPL18A//IGF2BP3//LARP4//RPL13A//RPS27L//RPL37A//RPS14//LSM14A//CNOT11//GCLM//GAPDH//CAPRIN1//ENC1//CNOT8//DYRK1A//KRT17//NAT10//THBS1//HABP4//FTO//PUM1//NEMF// |
| GO:2000134 | negative regulation of G1/S transition of mitotic cell cycle | Biological process | 6 | 126 | 262 | 18866 | 3.42893493275173 | 0.00835073635323258 | 0.29771640362358 | 2.07827522750493 | 0.0229007633587786 | MDM2//PLAGL1//CNOT11//CNOT8//RPS27L//RBL1// |
| GO:0034284 | response to monosaccharide | Biological process | 8 | 207 | 262 | 18866 | 2.78290371353763 | 0.00850801720452661 | 0.301799464556549 | 2.07017164055781 | 0.0305343511450382 | COL6A2//THBS1//RAB11FIP5//KLF7//GCLM//ICAM1//TRA2B//ZNF236// |
| GO:0010557 | positive regulation of macromolecule biosynthetic process | Biological process | 38 | 1841 | 262 | 18866 | 1.48630639670607 | 0.00857468613785075 | 0.302643547235442 | 2.0667817676227 | 0.145038167938931 | RBPJ//LARP4//KRT17//RPS27L//NAT10//THBS1//FAF1//CTCF//DNM2//DYRK1A//NFATC2//CRLF3//RNF111//GPBP1L1//RNF4//NPAS3//SPI1//TAF15//TRIM24//FANK1//DDX17//IFI16//MEIS1//PLAGL1//OVOL2//RBL1//RFX2//BMPR2//TMF1//NR2C2//NRIP1//KLF7//BRF1//HABP4//DDX11//CHCHD10//CCAR1//TADA2A// |
| GO:0031331 | positive regulation of cellular catabolic process | Biological process | 12 | 390 | 262 | 18866 | 2.21561949500881 | 0.00861822606100212 | 0.302666954052806 | 2.06458211830542 | 0.0458015267175573 | GSK3B//AMBRA1//TMEM59//SESN1//MDM2//CNOT8//FTO//PUM1//HUWE1//SIRT6//FAF1//LDLR// |
| GO:0031327 | negative regulation of cellular biosynthetic process | Biological process | 36 | 1724 | 262 | 18866 | 1.50363968048742 | 0.00881839089720575 | 0.305913339307138 | 2.05461065389518 | 0.137404580152672 | CTCF//TCFL5//PRDM5//NEDD4L//IFI16//RBPJ//MDM2//NFATC2//SIRT6//OVOL2//RBL1//BACH2//RPS14//SPI1//NR2C2//NRIP1//KLF11//KLF7//GTF2IRD1//NCOR2//IGF2BP3//RPL13A//GAPDH//CAPRIN1//ENC1//CNOT8//NAT10//GSK3B//SCMH1//TRIM24//FTO//PUM1//TMEM59//MORC2//CCAR1//BMPR2// |
| GO:0035556 | intracellular signal transduction | Biological process | 54 | 2837 | 262 | 18866 | 1.37060705454369 | 0.00889573354553539 | 0.305913339307138 | 2.05081823401704 | 0.206106870229008 | NF1//TAOK3//PSMB5//SRPK2//THBS1//MDM2//PLAGL1//CNOT11//CNOT8//RPS27L//GNAI2//GNB1//SPAG9//DOCK1//DOCK2//TIAM2//GDI2//BCR//MYO9B//AKAP7//PIP5K1C//PLCG2//PDE3A//PDE4D//KPNB1//ITFG2//NFATC2//DNM2//OPHN1//ARHGEF28//STARD13//LATS2//SMG1//LSM14A//IFI16//ZDHHC17//TERF2IP//TFRC//DYRK1A//GNAI1//PI4KA//RALGAPA2//TRIO//PHLPP2//ICAM1//GSK3B//PUM1//TAF15//ATRIP//TRIM24//SESN1//GPR137B//WSB1//STAC// |
| GO:1903311 | regulation of mRNA metabolic process | Biological process | 11 | 344 | 262 | 18866 | 2.30256967867921 | 0.00891783443732132 | 0.305913339307138 | 2.04974059473873 | 0.0419847328244275 | DDX17//DYRK1A//TRA2B//IGF2BP3//ALKBH5//PSMB5//PUM1//SRPK2//TAF15//CNOT8//FTO// |
| GO:0071539 | protein localization to centrosome | Biological process | 3 | 31 | 262 | 18866 | 6.96848066978577 | 0.00893178245491616 | 0.305913339307138 | 2.04906186327414 | 0.0114503816793893 | GSK3B//CEP83//HOOK3// |
| GO:0031324 | negative regulation of cellular metabolic process | Biological process | 53 | 2775 | 262 | 18866 | 1.37528092978475 | 0.00897364144461692 | 0.305913339307138 | 2.04703128734755 | 0.202290076335878 | CTCF//TCFL5//PRDM5//NEDD4L//IFI16//RBPJ//MDM2//NFATC2//SIRT6//OVOL2//RBL1//BACH2//RPS14//SPI1//NR2C2//NRIP1//KLF11//KLF7//GTF2IRD1//NCOR2//CORO1C//TERF2IP//NF1//TAOK3//SMG1//THBS1//PARD3//GAPDH//TMEM59//IGF2BP3//RPL13A//CAPRIN1//ENC1//CNOT8//NAT10//GSK3B//PDE4D//RNF4//ARL6IP1//LATS2//SCMH1//TRIM24//DYRK1A//TAF15//FTO//PUM1//MORC2//OPHN1//CCAR1//GNAI2//BMPR2//SIRT5//BCR// |
| GO:0022603 | regulation of anatomical structure morphogenesis | Biological process | 27 | 1198 | 262 | 18866 | 1.62287654997515 | 0.00902961759853445 | 0.305913339307138 | 2.04433064153178 | 0.103053435114504 | BMPR2//ICAM1//MYH9//FBLIM1//WDR1//HUWE1//TFRC//NF1//THBS1//LARP4//ZMYM4//DNM2//GSK3B//NEDD4L//HECW2//TIAM2//CAPRIN1//ANKRD27//PSMB5//BCR//STARD13//MYO19//CORO1C//DOCK1//NFATC2//SIRT6//RBPJ// |
| GO:0072413 | signal transduction involved in mitotic cell cycle checkpoint | Biological process | 4 | 59 | 262 | 18866 | 4.88187346357873 | 0.00910069432702917 | 0.305913339307138 | 2.04092547241581 | 0.0152671755725191 | MDM2//PLAGL1//CNOT11//CNOT8// |
| GO:1902402 | signal transduction involved in mitotic DNA damage checkpoint | Biological process | 4 | 59 | 262 | 18866 | 4.88187346357873 | 0.00910069432702917 | 0.305913339307138 | 2.04092547241581 | 0.0152671755725191 | MDM2//PLAGL1//CNOT11//CNOT8// |
| GO:1902403 | signal transduction involved in mitotic DNA integrity checkpoint | Biological process | 4 | 59 | 262 | 18866 | 4.88187346357873 | 0.00910069432702917 | 0.305913339307138 | 2.04092547241581 | 0.0152671755725191 | MDM2//PLAGL1//CNOT11//CNOT8// |
| GO:0051252 | regulation of RNA metabolic process | Biological process | 69 | 3807 | 262 | 18866 | 1.30510289402607 | 0.00914503603038366 | 0.30594696368947 | 2.03881457911419 | 0.263358778625954 | KLF11//CTCF//TCFL5//PRDM5//NEDD4L//IFI16//RBPJ//MDM2//NFATC2//SIRT6//OVOL2//RBL1//BACH2//RPS14//SPI1//NR2C2//NRIP1//KLF7//GTF2IRD1//NCOR2//DDX17//DYRK1A//TRA2B//ZNF483//DNM2//EFEMP1//SETD2//TERF2IP//CCAR1//GATAD1//GPBP1L1//ZNF236//TRRAP//CNOT8//ZNF646//ZNF652//LCORL//MEIS1//THAP4//PLAGL1//RFX2//NPAS3//TADA2A//FAF1//HABP4//CLK1//IGF2BP3//ALKBH5//PSMB5//PUM1//SCMH1//TRIM24//CRLF3//RNF111//RNF4//TAF15//FANK1//BMPR2//TMF1//BRF1//SRPK2//ICAM1//TFRC//FTO//MORC2//DDX11//CHCHD10//LPXN//PCBP2// |
| GO:0000956 | nuclear-transcribed mRNA catabolic process | Biological process | 8 | 210 | 262 | 18866 | 2.74314794620138 | 0.0092349569289792 | 0.306270378510455 | 2.03456512573 | 0.0305343511450382 | SMG1//RPL13A//RPL18A//RPL37A//RPS14//CNOT11//CNOT8//DDX6// |
| GO:0048666 | neuron development | Biological process | 26 | 1143 | 262 | 18866 | 1.63796891800739 | 0.0092414776346121 | 0.306270378510455 | 2.03425858318984 | 0.099236641221374 | PARD3//ACTB//KLF7//NFASC//OPHN1//SLIT3//TRIO//GSK3B//MOB2//ENC1//MDM2//PTPRG//TRAPPC4//STRN//DNM2//MTR//FRYL//TAOK3//ANKRD27//NEDD4L//HECW2//BMPR2//TIAM2//CAPRIN1//DDR1//PPT1// |
| GO:0019219 | regulation of nucleobase-containing compound metabolic process | Biological process | 73 | 4075 | 262 | 18866 | 1.28995269985482 | 0.00945770002479688 | 0.311623996339962 | 2.02421446486505 | 0.278625954198473 | KLF11//CTCF//TCFL5//PRDM5//NEDD4L//IFI16//RBPJ//MDM2//NFATC2//SIRT6//OVOL2//RBL1//BACH2//RPS14//SPI1//NR2C2//NRIP1//KLF7//GTF2IRD1//NCOR2//DDX17//DYRK1A//TRA2B//NUP155//ZNF483//DNM2//EFEMP1//SETD2//TERF2IP//CCAR1//GATAD1//GPBP1L1//ZNF236//TRRAP//CNOT8//ZNF646//ZNF652//LCORL//MEIS1//THAP4//PLAGL1//RFX2//NPAS3//TADA2A//FAF1//DDX11//SMG1//NAT10//HABP4//CLK1//IGF2BP3//ALKBH5//PSMB5//PUM1//EYA3//TFRC//SCMH1//TRIM24//CRLF3//RNF111//RNF4//TAF15//FANK1//BMPR2//TMF1//BRF1//SRPK2//ICAM1//PCBP2//FTO//MORC2//CHCHD10//LPXN// |
| GO:0022008 | neurogenesis | Biological process | 35 | 1674 | 262 | 18866 | 1.50553594717594 | 0.009609887816524 | 0.311623996339962 | 2.01728168214924 | 0.133587786259542 | SDCCAG8//NF1//PARD3//ACTB//KLF7//NFASC//OPHN1//SLIT3//TRIO//RBPJ//GSK3B//MOB2//ENC1//MDM2//PTPRG//TRAPPC4//STRN//DNM2//MTR//FRYL//DDX6//MEIS1//SPAG9//PPT1//WDR1//TAOK3//ANKRD27//NEDD4L//HECW2//BMPR2//HOOK3//TIAM2//CAPRIN1//DDR1//LDLR// |
| GO:0071426 | ribonucleoprotein complex export from nucleus | Biological process | 6 | 130 | 262 | 18866 | 3.32342924251321 | 0.00966185576314968 | 0.311623996339962 | 2.01493945015893 | 0.0229007633587786 | SMG1//CDC40//ALKBH5//FIP1L1//NUP155//SETD2// |
| GO:0003157 | endocardium development | Biological process | 2 | 11 | 262 | 18866 | 13.0922970159611 | 0.00972859252060504 | 0.311623996339962 | 2.01194998653015 | 0.00763358778625954 | RBPJ//OVOL2// |
| GO:0086023 | adenylate cyclase-activating adrenergic receptor signaling pathway involved in heart process | Biological process | 2 | 11 | 262 | 18866 | 13.0922970159611 | 0.00972859252060504 | 0.311623996339962 | 2.01194998653015 | 0.00763358778625954 | PDE4D//GNAI2// |
| GO:0019538 | protein metabolic process | Biological process | 100 | 5894 | 262 | 18866 | 1.22171078364076 | 0.00974027715107675 | 0.311623996339962 | 2.01142868547551 | 0.381679389312977 | ACTB//NF1//TAOK3//PSMB5//SRPK2//THBS1//HUWE1//NEDD4L//WSB1//MDM2//RNF111//HECW2//UBE3B//CORO1C//TERF2IP//TFRC//MOB2//PPT1//RPL18A//IGF2BP3//LARP4//RPL13A//RPS27L//RPL37A//RPS14//LSM14A//CNOT11//UBL5//RPN1//RPN2//DYRK1A//LATS2//GSK3B//PHKG1//BCR//BMPR2//TRIO//NEK9//PHLPP2//PTPRG//SIRT5//SIRT6//GALNT1//GALNT7//PITRM1//PRSS57//DPP8//CFAP44//PAPPA2//TMEM59//MYH9//ADAM19//USP42//SPAG9//ENC1//PARD3//GAPDH//G2E3//DCAF17//TRIM24//KLHL21//TRRAP//PRDM5//EYA3//RANGAP1//NUP155//CAPRIN1//CNOT8//SETD2//TTLL11//CLK1//SMG1//EFEMP1//PKDCC//ZDHHC17//CLN6//CTCF//PDE4D//RNF4//TADA2A//DDR1//LDLR//FAF1//ARL6IP1//PCBP2//RNF216//MIA3//RAB2A//SPI1//KRT17//NAT10//HABP4//ICAM1//TMF1//FTO//PUM1//GNAI2//NEMF//IFI16//OPHN1// |
| GO:0003176 | aortic valve development | Biological process | 3 | 32 | 262 | 18866 | 6.75071564885496 | 0.00975618404747578 | 0.311623996339962 | 2.01072001544633 | 0.0114503816793893 | SLIT3//RBPJ//BMPR2// |
| GO:0071295 | cellular response to vitamin | Biological process | 3 | 32 | 262 | 18866 | 6.75071564885496 | 0.00975618404747578 | 0.311623996339962 | 2.01072001544633 | 0.0114503816793893 | CYP24A1//TRIM24//MDM2// |
| GO:0032990 | cell part morphogenesis | Biological process | 18 | 705 | 262 | 18866 | 1.83849277245412 | 0.00984670389090562 | 0.31309857101758 | 2.00670912194437 | 0.0687022900763359 | PARD3//ACTB//KLF7//NFASC//OPHN1//SLIT3//TRIO//DNM2//GSK3B//TAOK3//ANKRD27//NEDD4L//HECW2//BMPR2//TIAM2//CAPRIN1//DDR1//CORO1C// |
| GO:0071166 | ribonucleoprotein complex localization | Biological process | 6 | 131 | 262 | 18866 | 3.29805955363907 | 0.0100113047601052 | 0.314141329752066 | 1.9995093178067 | 0.0229007633587786 | SMG1//CDC40//ALKBH5//FIP1L1//NUP155//SETD2// |
| GO:1901361 | organic cyclic compound catabolic process | Biological process | 17 | 653 | 262 | 18866 | 1.87462445787499 | 0.0100114560370451 | 0.314141329752066 | 1.99950275540096 | 0.0648854961832061 | SMG1//RPL13A//RPL18A//RPL37A//RPS14//CNOT11//CNOT8//PDE4D//KPNB1//CYP24A1//IGF2BP3//ALKBH5//PSMB5//PUM1//DDX6//TAF15//FTO// |
| GO:0050793 | regulation of developmental process | Biological process | 53 | 2791 | 262 | 18866 | 1.36739683989705 | 0.0100130045607331 | 0.314141329752066 | 1.9994355860217 | 0.202290076335878 | BMPR2//NF1//ICAM1//MYH9//FBLIM1//WDR1//HUWE1//TFRC//OVOL2//GSK3B//MOB2//ENC1//MDM2//PTPRG//THBS1//LARP4//ZMYM4//PKDCC//DNM2//PARD3//EFEMP1//FTO//SH3PXD2B//NR2C2//NFATC2//TCFL5//RBPJ//TRIO//KLF7//MEIS1//SPI1//DDX6//SPAG9//GPR137B//LATS2//NEDD4L//HECW2//HOOK3//TIAM2//CAPRIN1//ANKRD27//KRT17//PSMB5//PDE3A//BCR//LDLR//STARD13//MYO19//CORO1C//DOCK1//SIRT6//RBL1//DDX17// |
| GO:0003170 | heart valve development | Biological process | 4 | 61 | 262 | 18866 | 4.72181203854336 | 0.0102135284960583 | 0.315088045232175 | 1.99082419506934 | 0.0152671755725191 | RBPJ//BMPR2//SLIT3//MDM2// |
| GO:1902041 | regulation of extrinsic apoptotic signaling pathway via death domain receptors | Biological process | 4 | 61 | 262 | 18866 | 4.72181203854336 | 0.0102135284960583 | 0.315088045232175 | 1.99082419506934 | 0.0152671755725191 | GSK3B//ICAM1//FAF1//THBS1// |
| GO:0044772 | mitotic cell cycle phase transition | Biological process | 16 | 602 | 262 | 18866 | 1.91382414851259 | 0.0102463005516461 | 0.315088045232175 | 1.98943290924369 | 0.0610687022900763 | LATS2//NASP//CRLF3//KLF11//SDCCAG8//DYNC1H1//DNM2//MDM2//PLAGL1//CNOT11//CNOT8//TAOK3//PSMB5//HECW2//RPS27L//RBL1// |
| GO:0046700 | heterocycle catabolic process | Biological process | 16 | 602 | 262 | 18866 | 1.91382414851259 | 0.0102463005516461 | 0.315088045232175 | 1.98943290924369 | 0.0610687022900763 | SMG1//RPL13A//RPL18A//RPL37A//RPS14//CNOT11//CNOT8//PDE4D//KPNB1//IGF2BP3//ALKBH5//PSMB5//PUM1//DDX6//TAF15//FTO// |
| GO:1902807 | negative regulation of cell cycle G1/S phase transition | Biological process | 6 | 132 | 262 | 18866 | 3.27307425399028 | 0.0103696563344572 | 0.315088045232175 | 1.98423563652533 | 0.0229007633587786 | MDM2//PLAGL1//CNOT11//CNOT8//RPS27L//RBL1// |
| GO:0044270 | cellular nitrogen compound catabolic process | Biological process | 16 | 603 | 262 | 18866 | 1.91065031078703 | 0.0103978194929969 | 0.315088045232175 | 1.98305772622499 | 0.0610687022900763 | SMG1//RPL13A//RPL18A//RPL37A//RPS14//CNOT11//CNOT8//PDE4D//KPNB1//IGF2BP3//ALKBH5//PSMB5//PUM1//DDX6//TAF15//FTO// |
| GO:0051301 | cell division | Biological process | 16 | 603 | 262 | 18866 | 1.91065031078703 | 0.0103978194929969 | 0.315088045232175 | 1.98305772622499 | 0.0610687022900763 | TTC19//PARD3//SETD2//MYO19//KLHL21//CSPP1//MYH9//RBL1//TUBA1B//CCDC124//DYNC1H1//PDS5B//LATS2//GNAI1//GNAI2//NEK9// |
| GO:0048814 | regulation of dendrite morphogenesis | Biological process | 5 | 95 | 262 | 18866 | 3.78987545198875 | 0.0104577261595853 | 0.315088045232175 | 1.98056273456011 | 0.0190839694656489 | CAPRIN1//ANKRD27//NEDD4L//GSK3B//HECW2// |
| GO:0002691 | regulation of cellular extravasation | Biological process | 3 | 33 | 262 | 18866 | 6.54614850798057 | 0.0106234530054197 | 0.315088045232175 | 1.97373429893648 | 0.0114503816793893 | BCR//ICAM1//CD99L2// |
| GO:0009303 | rRNA transcription | Biological process | 3 | 33 | 262 | 18866 | 6.54614850798057 | 0.0106234530054197 | 0.315088045232175 | 1.97373429893648 | 0.0114503816793893 | GTF3C5//DDX11//BRF1// |
| GO:0010955 | negative regulation of protein processing | Biological process | 3 | 33 | 262 | 18866 | 6.54614850798057 | 0.0106234530054197 | 0.315088045232175 | 1.97373429893648 | 0.0114503816793893 | THBS1//MDM2//TMEM59// |
| GO:1903318 | negative regulation of protein maturation | Biological process | 3 | 33 | 262 | 18866 | 6.54614850798057 | 0.0106234530054197 | 0.315088045232175 | 1.97373429893648 | 0.0114503816793893 | THBS1//MDM2//TMEM59// |
| GO:1905508 | protein localization to microtubule organizing center | Biological process | 3 | 33 | 262 | 18866 | 6.54614850798057 | 0.0106234530054197 | 0.315088045232175 | 1.97373429893648 | 0.0114503816793893 | CEP83//HOOK3//GSK3B// |
| GO:0006997 | nucleus organization | Biological process | 6 | 133 | 262 | 18866 | 3.24846467313321 | 0.0107370265761062 | 0.315802794169724 | 1.96911597193066 | 0.0229007633587786 | NUP155//NEK9//TMF1//HABP4//SRPK2//PITPNB// |
| GO:0042770 | signal transduction in response to DNA damage | Biological process | 6 | 133 | 262 | 18866 | 3.24846467313321 | 0.0107370265761062 | 0.315802794169724 | 1.96911597193066 | 0.0229007633587786 | MDM2//PLAGL1//CNOT11//CNOT8//RPS27L//DYRK1A// |
| GO:0009891 | positive regulation of biosynthetic process | Biological process | 40 | 1991 | 262 | 18866 | 1.44666265369736 | 0.0108088219995004 | 0.316595329852586 | 1.96622163509331 | 0.152671755725191 | RBPJ//LDLR//DNM2//ICAM1//LARP4//KRT17//RPS27L//NAT10//THBS1//FAF1//CTCF//DYRK1A//NFATC2//CRLF3//RNF111//GPBP1L1//RNF4//NPAS3//SPI1//TAF15//TRIM24//FANK1//DDX17//IFI16//MEIS1//PLAGL1//OVOL2//RBL1//RFX2//BMPR2//TMF1//NR2C2//NRIP1//KLF7//BRF1//HABP4//DDX11//CHCHD10//CCAR1//TADA2A// |
| GO:0006807 | nitrogen compound metabolic process | Biological process | 166 | 10609 | 262 | 18866 | 1.12671007404774 | 0.0109437655171888 | 0.319223309032379 | 1.96083322078496 | 0.633587786259542 | ACTB//KLF11//MTR//CTCF//TCFL5//PRDM5//NEDD4L//IFI16//RBPJ//MDM2//NFATC2//SIRT6//OVOL2//RBL1//BACH2//RPS14//SPI1//NR2C2//NRIP1//KLF7//GTF2IRD1//NCOR2//NAT10//NF1//TAOK3//PSMB5//SRPK2//SMG1//RPL13A//RPL18A//RPL37A//THBS1//HUWE1//WSB1//RNF111//HECW2//UBE3B//CNOT11//CNOT8//TRA2B//DDX17//DYRK1A//PCBP2//CDC40//CCAR1//UBL5//FIP1L1//RCL1//NOL10//TERF2IP//CLN6//CORO1C//TFRC//MOB2//PPT1//MTO1//UGP2//GAPDH//HK1//NUP155//SLC23A2//PDE4D//DCTD//POLI//DDX11//PDS5B//TRRAP//ATRIP//SETD2//EYA3//ALKBH5//FTO//KPNB1//GPBP1L1//BRF1//ZNF483//DNM2//EFEMP1//GATAD1//ZNF236//ZNF646//ZNF652//LCORL//MEIS1//THAP4//PLAGL1//RFX2//NPAS3//TADA2A//TEX10//PHRF1//TAF15//TRIM24//GTF3C5//HABP4//IGF2BP3//LARP4//RPS27L//LSM14A//RPN1//RPN2//LATS2//GSK3B//PHKG1//BCR//BMPR2//TRIO//NEK9//PHLPP2//PTPRG//SIRT5//GALNT1//GALNT7//PITRM1//PRSS57//DPP8//CFAP44//PAPPA2//TMEM59//MYH9//ADAM19//USP42//GCLM//PCCB//FAF1//SPAG9//RPPH1//BCKDHA//ABCD4//ENC1//PARD3//LDLR//G2E3//DCAF17//KLHL21//RANGAP1//CAPRIN1//TTLL11//CLK1//PKDCC//ZDHHC17//CRAT//ELOVL6//RNF4//SAGE1//PUM1//OSBPL10//MBOAT2//DDR1//ARL6IP1//RNF216//MIA3//RAB2A//DDX6//ICAM1//KRT17//SCMH1//CRLF3//FANK1//TMF1//GNAI2//NEMF//MORC2//OPHN1//CHCHD10//LPXN// |
| GO:0031325 | positive regulation of cellular metabolic process | Biological process | 62 | 3382 | 262 | 18866 | 1.320068977659 | 0.0109906965381408 | 0.319272950052411 | 1.95897478318295 | 0.236641221374046 | THBS1//TFRC//MOB2//PLCG2//RPS27L//RBPJ//SPAG9//GSK3B//AMBRA1//TMEM59//BMPR2//LDLR//SESN1//HUWE1//RNF111//DDX11//TAOK3//SIRT6//MDM2//GNAI2//DYRK1A//HABP4//TERF2IP//DDR1//TADA2A//DNM2//ICAM1//LARP4//KRT17//NAT10//EYA3//FAF1//CLN6//CTCF//NFATC2//CRLF3//GPBP1L1//RNF4//NPAS3//SPI1//TAF15//TRIM24//FANK1//DDX17//IFI16//MEIS1//PLAGL1//OVOL2//RBL1//RFX2//TMF1//NR2C2//NRIP1//KLF7//BRF1//TRA2B//CNOT8//FTO//PUM1//CHCHD10//CCAR1//MYH9// |
| GO:0010810 | regulation of cell-substrate adhesion | Biological process | 8 | 217 | 262 | 18866 | 2.65465930277553 | 0.0111105151292613 | 0.320205769046141 | 1.95426580491068 | 0.0305343511450382 | NF1//DDR1//THBS1//GSK3B//NID1//CORO1C//DNM2//DOCK1// |
| GO:0071333 | cellular response to glucose stimulus | Biological process | 6 | 134 | 262 | 18866 | 3.22422239945312 | 0.0111135307290416 | 0.320205769046141 | 1.95414794533096 | 0.0229007633587786 | RAB11FIP5//KLF7//GCLM//ICAM1//TRA2B//ZNF236// |
| GO:0033044 | regulation of chromosome organization | Biological process | 11 | 356 | 262 | 18866 | 2.22495497040913 | 0.0113249890509734 | 0.322764561962373 | 1.94596220919188 | 0.0419847328244275 | HECW2//CTCF//SIRT6//SMG1//TERF2IP//NAT10//TADA2A//DDX11//RNF4//SPI1//MORC2// |
| GO:0031122 | cytoplasmic microtubule organization | Biological process | 4 | 63 | 262 | 18866 | 4.57191324366897 | 0.0114109146902323 | 0.322764561962373 | 1.94267954147147 | 0.0152671755725191 | KPNB1//TUBGCP2//DYNC1H1//HOOK3// |
| GO:0009890 | negative regulation of biosynthetic process | Biological process | 36 | 1755 | 262 | 18866 | 1.47707966333921 | 0.0114720353249759 | 0.322764561962373 | 1.94035952438387 | 0.137404580152672 | CTCF//TCFL5//PRDM5//NEDD4L//IFI16//RBPJ//MDM2//NFATC2//SIRT6//OVOL2//RBL1//BACH2//RPS14//SPI1//NR2C2//NRIP1//KLF11//KLF7//GTF2IRD1//NCOR2//IGF2BP3//RPL13A//GAPDH//CAPRIN1//ENC1//CNOT8//NAT10//GSK3B//SCMH1//TRIM24//FTO//PUM1//TMEM59//MORC2//CCAR1//BMPR2// |
| GO:0032879 | regulation of localization | Biological process | 54 | 2875 | 262 | 18866 | 1.35249120477929 | 0.0114802801018321 | 0.322764561962373 | 1.94004751566661 | 0.206106870229008 | ARL6IP1//PLCG2//MIA3//BCR//ICAM1//BMPR2//THBS1//CORO1C//MCC//PTPRG//DOCK1//SETD2//PDE4D//FTO//RPH3AL//ACTB//OPHN1//ATP8A1//GNAI2//CCAR1//SPAG9//NF1//DYNC1H1//PLEKHM2//TBC1D1//NEDD4L//NALCN//ANKRD27//PKDCC//SIRT6//RANGAP1//GSK3B//MDM2//PPT1//GOLPH3L//GAPDH//DNM2//DOCK2//WDR1//PARD3//KLF7//RAB11FIP5//STARD13//GPR137B//TFRC//CHCHD10//STAC//TMEM59//HUWE1//UBL5//GNAI1//CD99L2//HECW2//TMF1// |
| GO:0051173 | positive regulation of nitrogen compound metabolic process | Biological process | 59 | 3196 | 262 | 18866 | 1.32930237224011 | 0.0115295778896895 | 0.322764561962373 | 1.93818659240472 | 0.225190839694656 | THBS1//TFRC//MOB2//RPS27L//RBPJ//SPAG9//BMPR2//HUWE1//RNF111//DDX11//TAOK3//SIRT6//GSK3B//MDM2//DYRK1A//HABP4//TERF2IP//TADA2A//DNM2//ICAM1//LARP4//KRT17//NAT10//NEDD4L//HECW2//EYA3//CLN6//CTCF//NFATC2//CRLF3//GPBP1L1//RNF4//NPAS3//SPI1//TAF15//TRIM24//FANK1//DDX17//IFI16//MEIS1//PLAGL1//OVOL2//RBL1//RFX2//TMF1//NR2C2//NRIP1//KLF7//BRF1//TRA2B//CNOT8//FTO//PUM1//GNAI2//CHCHD10//FAF1//CCAR1//MYH9//LDLR// |
| GO:0001768 | establishment of T cell polarity | Biological process | 2 | 12 | 262 | 18866 | 12.001272264631 | 0.0115681306384021 | 0.322764561962373 | 1.93673681555293 | 0.00763358778625954 | DOCK2//MYH9// |
| GO:0034333 | adherens junction assembly | Biological process | 2 | 12 | 262 | 18866 | 12.001272264631 | 0.0115681306384021 | 0.322764561962373 | 1.93673681555293 | 0.00763358778625954 | PIP5K1C//ACTB// |
| GO:2000650 | negative regulation of sodium ion transmembrane transporter activity | Biological process | 2 | 12 | 262 | 18866 | 12.001272264631 | 0.0115681306384021 | 0.322764561962373 | 1.93673681555293 | 0.00763358778625954 | NEDD4L//HECW2// |
| GO:0010769 | regulation of cell morphogenesis involved in differentiation | Biological process | 10 | 310 | 262 | 18866 | 2.32282688992859 | 0.011689194014215 | 0.323584394299387 | 1.93221543299952 | 0.0381679389312977 | DNM2//GSK3B//NEDD4L//HECW2//BMPR2//TIAM2//CAPRIN1//ANKRD27//CORO1C//DOCK1// |
| GO:0044843 | cell cycle G1/S phase transition | Biological process | 10 | 310 | 262 | 18866 | 2.32282688992859 | 0.011689194014215 | 0.323584394299387 | 1.93221543299952 | 0.0381679389312977 | LATS2//NASP//CRLF3//KLF11//MDM2//PLAGL1//CNOT11//CNOT8//RPS27L//RBL1// |
| GO:0045727 | positive regulation of translation | Biological process | 6 | 136 | 262 | 18866 | 3.17680736416704 | 0.0118943972843591 | 0.326702530857163 | 1.92465755973926 | 0.0229007633587786 | HABP4//LARP4//KRT17//RPS27L//NAT10//THBS1// |
| GO:0071331 | cellular response to hexose stimulus | Biological process | 6 | 136 | 262 | 18866 | 3.17680736416704 | 0.0118943972843591 | 0.326702530857163 | 1.92465755973926 | 0.0229007633587786 | RAB11FIP5//KLF7//GCLM//ICAM1//TRA2B//ZNF236// |
| GO:0007163 | establishment or maintenance of cell polarity | Biological process | 8 | 220 | 262 | 18866 | 2.61845940319223 | 0.0119951224718624 | 0.326924978875972 | 1.92099531346668 | 0.0305343511450382 | DOCK2//MYH9//SDCCAG8//GSK3B//PARD3//WDR1//OPHN1//ACTB// |
| GO:0017148 | negative regulation of translation | Biological process | 8 | 220 | 262 | 18866 | 2.61845940319223 | 0.0119951224718624 | 0.326924978875972 | 1.92099531346668 | 0.0305343511450382 | FTO//PUM1//RPL13A//IGF2BP3//GAPDH//CAPRIN1//ENC1//CNOT8// |
| GO:0071310 | cellular response to organic substance | Biological process | 52 | 2756 | 262 | 18866 | 1.35863459599597 | 0.0121221070456044 | 0.328646792879893 | 1.91642188522371 | 0.198473282442748 | PARD3//LPXN//GNB1//GSK3B//AP3S1//ATP6V0B//LATS2//ICAM1//BMPR2//RNF111//THBS1//DNM2//RBPJ//DDX17//TMF1//CANX//SLIT3//PSMB5//IFI16//GCLM//RAB11FIP5//DDX11//GNAI2//IFI30//LSM14A//NCOR2//KLF7//SPI1//CYP24A1//TRIM24//PDE4D//BCR//SESN1//MDM2//PDE3A//TRA2B//ZNF236//FLNB//RPL13A//GAPDH//TUBA1B//NRIP1//LDLR//OVOL2//ACTB//KLF11//RANGAP1//GNAI1//PRDM5//ABCD4//RFX2//TFRC// |
| GO:0048812 | neuron projection morphogenesis | Biological process | 17 | 667 | 262 | 18866 | 1.83527701797956 | 0.0121514113814495 | 0.328646792879893 | 1.91537327600997 | 0.0648854961832061 | PARD3//ACTB//KLF7//NFASC//OPHN1//SLIT3//TRIO//DNM2//GSK3B//NEDD4L//HECW2//BMPR2//TIAM2//CAPRIN1//ANKRD27//DDR1//TAOK3// |
| GO:0071326 | cellular response to monosaccharide stimulus | Biological process | 6 | 137 | 262 | 18866 | 3.15361898924611 | 0.0122989856010584 | 0.330163072095796 | 1.91013070693703 | 0.0229007633587786 | RAB11FIP5//KLF7//GCLM//ICAM1//TRA2B//ZNF236// |
| GO:0048771 | tissue remodeling | Biological process | 7 | 178 | 262 | 18866 | 2.8317608714298 | 0.0123406472281869 | 0.330163072095796 | 1.90866206232394 | 0.0267175572519084 | RBPJ//MDM2//BMPR2//NF1//GPR137B//TFRC//BCR// |
| GO:0051239 | regulation of multicellular organismal process | Biological process | 62 | 3402 | 262 | 18866 | 1.31230843105313 | 0.012417901080745 | 0.330163072095796 | 1.90595180400507 | 0.236641221374046 | IFI16//TMF1//MDM2//PDE4D//BMPR2//NF1//THBS1//CORO1C//MCC//PTPRG//DOCK1//PLCG2//OVOL2//GSK3B//MOB2//ENC1//GTF2IRD1//RBPJ//PKDCC//DNM2//PARD3//EFEMP1//PCBP2//RNF216//SETD2//GNAI2//LSM14A//FTO//SH3PXD2B//NR2C2//IGF2BP3//NFATC2//KLF7//MEIS1//SPI1//DDX6//SPAG9//GPR137B//TFRC//ICAM1//LATS2//WDR1//NEDD4L//HECW2//GAPDH//HOOK3//TIAM2//CAPRIN1//ANKRD27//KRT17//PSMB5//PDE3A//BCR//LDLR//RAB11FIP5//NUP155//STARD13//DYNC1H1//SIRT6//ELOVL6//PUM1//DDX17// |
| GO:0006281 | DNA repair | Biological process | 15 | 563 | 262 | 18866 | 1.91849823058045 | 0.012461527897791 | 0.330163072095796 | 1.90442870591932 | 0.0572519083969466 | HUWE1//SIRT6//SETD2//EYA3//ALKBH5//FTO//TERF2IP//POLI//ATRIP//RNF111//DDX11//PDS5B//SMG1//TAOK3//TRRAP// |
| GO:0030182 | neuron differentiation | Biological process | 30 | 1406 | 262 | 18866 | 1.53643599404949 | 0.0124879335467192 | 0.330163072095796 | 1.90350942099384 | 0.114503816793893 | PARD3//ACTB//KLF7//NFASC//OPHN1//SLIT3//TRIO//RBPJ//GSK3B//MOB2//ENC1//MDM2//PTPRG//TRAPPC4//STRN//DNM2//MTR//FRYL//DDX6//MEIS1//SPAG9//PPT1//TAOK3//ANKRD27//NEDD4L//HECW2//BMPR2//TIAM2//CAPRIN1//DDR1// |
| GO:0039694 | viral RNA genome replication | Biological process | 3 | 35 | 262 | 18866 | 6.17208287895311 | 0.0124881059993735 | 0.330163072095796 | 1.90350342362696 | 0.0114503816793893 | MORC2//PCBP2//PI4KA// |
| GO:0007399 | nervous system development | Biological process | 47 | 2449 | 262 | 18866 | 1.38193498514739 | 0.0128744845950502 | 0.339108159539028 | 1.89027014807365 | 0.179389312977099 | SDCCAG8//OVOL2//SETD2//NF1//PARD3//ACTB//KLF7//NFASC//OPHN1//SLIT3//TRIO//MEIS1//PPT1//PTPRG//BCR//BMPR2//UGP2//RBPJ//GSK3B//MOB2//ENC1//MDM2//TRAPPC4//STRN//CORO1C//COX6B1//PHLPP2//TRA2B//AMBRA1//HOOK3//DNM2//MTR//FRYL//DDX6//SPAG9//WDR1//TAOK3//ANKRD27//NEDD4L//HECW2//TIAM2//CAPRIN1//DDR1//LDLR//IGF2BP3//DYRK1A//NR2C2// |
| GO:0030163 | protein catabolic process | Biological process | 22 | 948 | 262 | 18866 | 1.67106322672078 | 0.0130609236772077 | 0.341223143220145 | 1.88402610838029 | 0.083969465648855 | PPT1//HUWE1//NEDD4L//MDM2//RNF111//HECW2//USP42//UBE3B//PSMB5//ENC1//GSK3B//LDLR//FAF1//PCBP2//RNF216//RNF4//TMF1//SIRT6//OPHN1//NEMF//CLN6//TRIM24// |
| GO:0040011 | locomotion | Biological process | 40 | 2016 | 262 | 18866 | 1.42872288864655 | 0.0131162900227931 | 0.341223143220145 | 1.88218898892962 | 0.152671755725191 | CORO1C//OVOL2//SDCCAG8//MIA3//BCR//ICAM1//OPHN1//DOCK2//CFAP44//NFASC//SLIT3//TRIO//KLF7//BMPR2//THBS1//MCC//PTPRG//DOCK1//PLCG2//DDR1//NFATC2//TMF1//ATP8A1//GNAI2//CCAR1//SPAG9//NF1//PIP5K1C//PDE4D//CLN6//SETD2//MYH9//STARD13//LPXN//ACTB//SLC3A2//CD99L2//PDS5B//MDM2//WDR1// |
| GO:0019222 | regulation of metabolic process | Biological process | 123 | 7553 | 262 | 18866 | 1.17263854512084 | 0.0131261311536552 | 0.341223143220145 | 1.88186326067454 | 0.469465648854962 | ACTB//KLF11//CTCF//TCFL5//PRDM5//NEDD4L//IFI16//RBPJ//MDM2//NFATC2//SIRT6//OVOL2//RBL1//BACH2//RPS14//SPI1//NR2C2//NRIP1//KLF7//GTF2IRD1//NCOR2//SMG1//RPL13A//RPL18A//RPL37A//THBS1//CNOT11//CNOT8//DDX17//DYRK1A//TRA2B//TMF1//CORO1C//TERF2IP//TFRC//MOB2//PLCG2//NUP155//ZNF483//DNM2//EFEMP1//SETD2//CCAR1//GATAD1//GPBP1L1//ZNF236//TRRAP//ZNF646//ZNF652//LCORL//MEIS1//THAP4//PLAGL1//RFX2//NPAS3//TADA2A//LSM14A//NF1//TAOK3//PSMB5//RPS27L//FAF1//SPAG9//IGF2BP3//AFF3//PCBP2//SRPK2//GPR137B//GSK3B//AMBRA1//TMEM59//SIRT5//PUM1//SH3PXD2B//LDLR//ADAM19//SLIT3//PARD3//BMPR2//GAPDH//SESN1//ATP6V0B//SCMH1//CAPRIN1//ENC1//TIAM2//HUWE1//RNF111//DDX11//NAT10//RNF216//PDE4D//GNAI2//HABP4//RNF4//DDR1//ARL6IP1//CLK1//ALKBH5//DDX6//ICAM1//KPNB1//ELOVL6//LARP4//KRT17//HECW2//LATS2//EYA3//MORC2//CLN6//TRIM24//CRLF3//TAF15//FANK1//BRF1//BCR//FTO//NEMF//OPHN1//DYNC1H1//CHCHD10//LPXN//MYH9// |
| GO:0019439 | aromatic compound catabolic process | Biological process | 16 | 620 | 262 | 18866 | 1.85826151194287 | 0.0132563657370857 | 0.341223143220145 | 1.87757552243671 | 0.0610687022900763 | SMG1//RPL13A//RPL18A//RPL37A//RPS14//CNOT11//CNOT8//PDE4D//KPNB1//IGF2BP3//ALKBH5//PSMB5//PUM1//DDX6//TAF15//FTO// |
| GO:0033572 | transferrin transport | Biological process | 3 | 36 | 262 | 18866 | 6.00063613231552 | 0.0134861413650683 | 0.341223143220145 | 1.87011229225338 | 0.0114503816793893 | DNM2//ATP6V0B//TFRC// |
| GO:0007275 | multicellular organism development | Biological process | 95 | 5618 | 262 | 18866 | 1.21764421339261 | 0.0135283127863278 | 0.341223143220145 | 1.86875636402905 | 0.362595419847328 | EVC//SH3PXD2B//PKDCC//SETD2//RBPJ//MEIS1//MYH9//OVOL2//SRPK2//ICAM1//NRIP1//MDM2//BMPR2//NF1//CORO1C//SDCCAG8//NASP//DDX17//GSK3B//ADAM19//THBS1//MIA3//PDS5B//ACP6//ITFG2//PLCG2//SPI1//SLIT3//SIRT6//DNM2//FLNB//RNF111//IGF2BP3//DYRK1A//MTR//OPHN1//PPT1//NR2C2//ENC1//PARD3//ACTB//KLF7//NFASC//TRIO//PTPRG//BCR//UGP2//DDR1//NCOR2//SCMH1//MOB2//TRAPPC4//STRN//COX6B1//PHLPP2//TRA2B//AMBRA1//HOOK3//IFI16//RPS14//WDR1//TFRC//KRT17//FRYL//EFEMP1//TTC39C//NID1//TMF1//AFF3//GTF3C5//LMBR1//DOCK2//NFATC2//DDX6//SPAG9//GPR137B//TAOK3//ANKRD27//NEDD4L//HECW2//LDLR//TIAM2//CAPRIN1//GNB1//PSMB5//PAPPA2//FTO//STARD13//TCFL5//DDX11//EYA3//LSM14A//G2E3//ZMYM4//GTF2IRD1// |
| GO:0001767 | establishment of lymphocyte polarity | Biological process | 2 | 13 | 262 | 18866 | 11.078097475044 | 0.013547225796159 | 0.341223143220145 | 1.8681496306031 | 0.00763358778625954 | DOCK2//MYH9// |
| GO:0010635 | regulation of mitochondrial fusion | Biological process | 2 | 13 | 262 | 18866 | 11.078097475044 | 0.013547225796159 | 0.341223143220145 | 1.8681496306031 | 0.00763358778625954 | HUWE1//TFRC// |
| GO:0015801 | aromatic amino acid transport | Biological process | 2 | 13 | 262 | 18866 | 11.078097475044 | 0.013547225796159 | 0.341223143220145 | 1.8681496306031 | 0.00763358778625954 | SLC15A4//SLC3A2// |
| GO:0031453 | positive regulation of heterochromatin assembly | Biological process | 2 | 13 | 262 | 18866 | 11.078097475044 | 0.013547225796159 | 0.341223143220145 | 1.8681496306031 | 0.00763358778625954 | MORC2//SIRT6// |
| GO:0072683 | T cell extravasation | Biological process | 2 | 13 | 262 | 18866 | 11.078097475044 | 0.013547225796159 | 0.341223143220145 | 1.8681496306031 | 0.00763358778625954 | CD99L2//ICAM1// |
| GO:1902306 | negative regulation of sodium ion transmembrane transport | Biological process | 2 | 13 | 262 | 18866 | 11.078097475044 | 0.013547225796159 | 0.341223143220145 | 1.8681496306031 | 0.00763358778625954 | NEDD4L//HECW2// |
| GO:0045732 | positive regulation of protein catabolic process | Biological process | 8 | 225 | 262 | 18866 | 2.56027141645462 | 0.0135831850467291 | 0.341223143220145 | 1.86699838277781 | 0.0305343511450382 | GSK3B//MDM2//SIRT6//FAF1//LDLR//HUWE1//NEDD4L//HECW2// |
| GO:0060840 | artery development | Biological process | 5 | 102 | 262 | 18866 | 3.5297859601856 | 0.0139119690477188 | 0.34717280004975 | 1.85661139725174 | 0.0190839694656489 | DNM2//RBPJ//LDLR//NF1//BMPR2// |
| GO:2001020 | regulation of response to DNA damage stimulus | Biological process | 8 | 226 | 262 | 18866 | 2.54894278186854 | 0.0139183882156225 | 0.34717280004975 | 1.85641105420749 | 0.0305343511450382 | SETD2//SIRT6//TERF2IP//DYRK1A//MDM2//EYA3//DDX11//SMG1// |
| GO:0030010 | establishment of cell polarity | Biological process | 6 | 141 | 262 | 18866 | 3.06415462075686 | 0.0140142946318841 | 0.348334175374894 | 1.85342875628326 | 0.0229007633587786 | DOCK2//MYH9//OPHN1//PARD3//SDCCAG8//GSK3B// |
| GO:0070613 | regulation of protein processing | Biological process | 4 | 67 | 262 | 18866 | 4.29896319927082 | 0.0140679972863993 | 0.348442080156816 | 1.85176772412691 | 0.0152671755725191 | THBS1//MDM2//TMEM59//MYH9// |
| GO:0007051 | spindle organization | Biological process | 7 | 183 | 262 | 18866 | 2.75439035581696 | 0.014189939247143 | 0.35023350050903 | 1.8480194639285 | 0.0267175572519084 | MYH9//DYNC1H1//KPNB1//TUBGCP2//STARD9//GNAI1//RNF4// |
| GO:0051603 | proteolysis involved in cellular protein catabolic process | Biological process | 18 | 734 | 262 | 18866 | 1.76585477463236 | 0.0143804163434685 | 0.353698114872976 | 1.84222854002731 | 0.0687022900763359 | HUWE1//NEDD4L//MDM2//RNF111//HECW2//USP42//UBE3B//PSMB5//ENC1//GSK3B//FAF1//PCBP2//RNF216//RNF4//TMF1//SIRT6//OPHN1//NEMF// |
| GO:0033120 | positive regulation of RNA splicing | Biological process | 3 | 37 | 262 | 18866 | 5.83845677738808 | 0.0145283495621685 | 0.354863735499472 | 1.83778371927078 | 0.0114503816793893 | TRA2B//DYRK1A//HABP4// |
| GO:1905314 | semi-lunar valve development | Biological process | 3 | 37 | 262 | 18866 | 5.83845677738808 | 0.0145283495621685 | 0.354863735499472 | 1.83778371927078 | 0.0114503816793893 | RBPJ//BMPR2//SLIT3// |
| GO:0034641 | cellular nitrogen compound metabolic process | Biological process | 115 | 7016 | 262 | 18866 | 1.18028475806662 | 0.0146491241593564 | 0.35657988772723 | 1.83418834009685 | 0.438931297709924 | KLF11//CTCF//TCFL5//PRDM5//NEDD4L//IFI16//RBPJ//MDM2//NFATC2//SIRT6//OVOL2//RBL1//BACH2//RPS14//SPI1//NR2C2//NRIP1//KLF7//GTF2IRD1//NCOR2//NAT10//SMG1//RPL13A//RPL18A//RPL37A//SRPK2//CNOT11//CNOT8//TRA2B//DDX17//DYRK1A//PCBP2//CDC40//CCAR1//UBL5//FIP1L1//RCL1//NOL10//TERF2IP//CLN6//MTO1//UGP2//GAPDH//HK1//NUP155//SLC23A2//PDE4D//DCTD//POLI//DDX11//PDS5B//TAOK3//TRRAP//ATRIP//HUWE1//SETD2//EYA3//ALKBH5//FTO//KPNB1//GPBP1L1//BRF1//ZNF483//DNM2//EFEMP1//GATAD1//ZNF236//ZNF646//ZNF652//LCORL//MEIS1//THAP4//PLAGL1//RFX2//NPAS3//TADA2A//TEX10//PHRF1//TAF15//TRIM24//GTF3C5//HABP4//IGF2BP3//LARP4//RPS27L//LSM14A//PSMB5//GCLM//PCCB//FAF1//RPPH1//CAPRIN1//ENC1//CRAT//SAGE1//PUM1//ELOVL6//CLK1//DDX6//ICAM1//KRT17//THBS1//TFRC//SCMH1//CRLF3//RNF111//RNF4//FANK1//BMPR2//TMF1//PPT1//NEMF//MORC2//CHCHD10//LPXN// |
| GO:0002504 | antigen processing and presentation of peptide or polysaccharide antigen via MHC class II | Biological process | 5 | 104 | 262 | 18866 | 3.46190546095126 | 0.0150243274129318 | 0.364456107243593 | 1.82320496074888 | 0.0190839694656489 | THBS1//IFI30//DYNC1H1//DNM2//CANX// |
| GO:0046483 | heterocycle metabolic process | Biological process | 106 | 6399 | 262 | 18866 | 1.19281280829901 | 0.0152526316441294 | 0.366078748231764 | 1.81665521796172 | 0.404580152671756 | KLF11//CTCF//TCFL5//PRDM5//NEDD4L//IFI16//RBPJ//MDM2//NFATC2//SIRT6//OVOL2//RBL1//BACH2//RPS14//SPI1//NR2C2//NRIP1//KLF7//GTF2IRD1//NCOR2//NAT10//SMG1//RPL13A//RPL18A//RPL37A//SRPK2//CNOT11//CNOT8//TRA2B//DDX17//DYRK1A//PCBP2//CDC40//CCAR1//UBL5//FIP1L1//RCL1//NOL10//TERF2IP//MTO1//UGP2//GAPDH//HK1//NUP155//SLC23A2//PDE4D//DCTD//POLI//DDX11//PDS5B//TAOK3//TRRAP//ATRIP//HUWE1//SETD2//EYA3//ALKBH5//FTO//KPNB1//GPBP1L1//BRF1//ZNF483//DNM2//EFEMP1//GATAD1//ZNF236//ZNF646//ZNF652//LCORL//MEIS1//THAP4//PLAGL1//RFX2//NPAS3//TADA2A//TEX10//PHRF1//TAF15//TRIM24//GTF3C5//HABP4//PCCB//FAF1//RPPH1//MTR//ABCD4//SAGE1//PUM1//ELOVL6//CLK1//IGF2BP3//PSMB5//DDX6//TFRC//SCMH1//CRLF3//RNF111//RNF4//FANK1//BMPR2//TMF1//PPT1//ICAM1//MORC2//CHCHD10//LPXN// |
| GO:0097191 | extrinsic apoptotic signaling pathway | Biological process | 8 | 230 | 262 | 18866 | 2.50461334218387 | 0.0153197879993322 | 0.366078748231764 | 1.81474724458313 | 0.0305343511450382 | GABARAP//NF1//GSK3B//ICAM1//FAF1//THBS1//GCLM//EYA3// |
| GO:0006405 | RNA export from nucleus | Biological process | 6 | 144 | 262 | 18866 | 3.00031806615776 | 0.015405610858465 | 0.366078748231764 | 1.81232107649661 | 0.0229007633587786 | SMG1//CDC40//ALKBH5//FIP1L1//NUP155//SETD2// |
| GO:0043603 | cellular amide metabolic process | Biological process | 26 | 1194 | 262 | 18866 | 1.56800542150958 | 0.0154791060141324 | 0.366078748231764 | 1.81025412532888 | 0.099236641221374 | CLN6//RPL18A//IGF2BP3//LARP4//RPL13A//RPS27L//RPL37A//RPS14//LSM14A//CNOT11//GCLM//PCCB//GAPDH//CAPRIN1//ENC1//CNOT8//DYRK1A//ELOVL6//KRT17//NAT10//THBS1//HABP4//PPT1//FTO//PUM1//NEMF// |
| GO:1903317 | regulation of protein maturation | Biological process | 4 | 69 | 262 | 18866 | 4.17435557030645 | 0.015531653766465 | 0.366078748231764 | 1.80878229936406 | 0.0152671755725191 | THBS1//MDM2//TMEM59//MYH9// |
| GO:0000462 | maturation of SSU-rRNA from tricistronic rRNA transcript (SSU-rRNA, 5.8S rRNA, LSU-rRNA) | Biological process | 3 | 38 | 262 | 18866 | 5.68481317798313 | 0.0156149573482516 | 0.366078748231764 | 1.80645919769342 | 0.0114503816793893 | RCL1//RPS14//NOL10// |
| GO:0044003 | modulation by symbiont of host process | Biological process | 3 | 38 | 262 | 18866 | 5.68481317798313 | 0.0156149573482516 | 0.366078748231764 | 1.80645919769342 | 0.0114503816793893 | KPNB1//KPNA5//GAPDH// |
| GO:0090224 | regulation of spindle organization | Biological process | 3 | 38 | 262 | 18866 | 5.68481317798313 | 0.0156149573482516 | 0.366078748231764 | 1.80645919769342 | 0.0114503816793893 | DYNC1H1//GNAI1//RNF4// |
| GO:1903432 | regulation of TORC1 signaling | Biological process | 3 | 38 | 262 | 18866 | 5.68481317798313 | 0.0156149573482516 | 0.366078748231764 | 1.80645919769342 | 0.0114503816793893 | SESN1//ITFG2//GPR137B// |
| GO:0035751 | regulation of lysosomal lumen pH | Biological process | 2 | 14 | 262 | 18866 | 10.2868047982552 | 0.0156616775699097 | 0.366078748231764 | 1.80516172118393 | 0.00763358778625954 | CLN6//PPT1// |
| GO:0045799 | positive regulation of chromatin assembly or disassembly | Biological process | 2 | 14 | 262 | 18866 | 10.2868047982552 | 0.0156616775699097 | 0.366078748231764 | 1.80516172118393 | 0.00763358778625954 | MORC2//SIRT6// |
| GO:0043161 | proteasome-mediated ubiquitin-dependent protein catabolic process | Biological process | 12 | 424 | 262 | 18866 | 2.03795189399395 | 0.0158274051188238 | 0.368731527174182 | 1.80059028128113 | 0.0458015267175573 | PSMB5//GSK3B//MDM2//SIRT6//NEMF//HUWE1//FAF1//NEDD4L//PCBP2//RNF216//HECW2//RNF4// |
| GO:0071322 | cellular response to carbohydrate stimulus | Biological process | 6 | 145 | 262 | 18866 | 2.97962621742564 | 0.0158899148371853 | 0.368970094854247 | 1.79887843040996 | 0.0229007633587786 | RAB11FIP5//KLF7//GCLM//ICAM1//TRA2B//ZNF236// |
| GO:0071156 | regulation of cell cycle arrest | Biological process | 5 | 106 | 262 | 18866 | 3.39658648998992 | 0.0161947180249331 | 0.374400940210387 | 1.79062660932603 | 0.0190839694656489 | MDM2//PLAGL1//CNOT11//CNOT8//CRLF3// |
| GO:0010605 | negative regulation of macromolecule metabolic process | Biological process | 64 | 3578 | 262 | 18866 | 1.2880068612684 | 0.016229875011245 | 0.374400940210387 | 1.78968482472926 | 0.244274809160305 | CTCF//TCFL5//PRDM5//NEDD4L//IFI16//RBPJ//MDM2//NFATC2//SIRT6//OVOL2//RBL1//BACH2//RPS14//SPI1//NR2C2//NRIP1//KLF11//KLF7//GTF2IRD1//NCOR2//SMG1//RPL13A//RPL18A//RPL37A//CNOT11//CNOT8//CORO1C//TERF2IP//NF1//TAOK3//LDLR//SLIT3//TMF1//THBS1//PARD3//GAPDH//TMEM59//PUM1//SCMH1//IGF2BP3//CAPRIN1//ENC1//DDX17//NAT10//PCBP2//RNF216//GSK3B//PDE4D//RNF4//ARL6IP1//ALKBH5//PSMB5//DDX6//LATS2//MORC2//TRIM24//DYRK1A//TAF15//NUP155//FTO//OPHN1//CCAR1//GNAI2//BMPR2// |
| GO:0048813 | dendrite morphogenesis | Biological process | 6 | 146 | 262 | 18866 | 2.95921781867615 | 0.0163846562582881 | 0.376695534216004 | 1.78556266546224 | 0.0229007633587786 | NEDD4L//GSK3B//HECW2//CAPRIN1//ANKRD27//KLF7// |
| GO:0009743 | response to carbohydrate | Biological process | 8 | 233 | 262 | 18866 | 2.47236510172657 | 0.0164360709078523 | 0.376695534216004 | 1.78420199378991 | 0.0305343511450382 | COL6A2//THBS1//RAB11FIP5//KLF7//GCLM//ICAM1//TRA2B//ZNF236// |
| GO:0051248 | negative regulation of protein metabolic process | Biological process | 26 | 1201 | 262 | 18866 | 1.55886633911944 | 0.0165509791393888 | 0.378101494320212 | 1.78117630870064 | 0.099236641221374 | CORO1C//TERF2IP//NF1//TAOK3//THBS1//PARD3//GAPDH//MDM2//TMEM59//IGF2BP3//RPL13A//CAPRIN1//ENC1//CNOT8//GSK3B//PDE4D//RNF4//ARL6IP1//LATS2//FTO//PUM1//SPI1//OPHN1//GNAI2//LDLR//IFI16// |
| GO:0035601 | protein deacylation | Biological process | 5 | 107 | 262 | 18866 | 3.36484269101805 | 0.0168020421990196 | 0.380146204752818 | 1.77463792887753 | 0.0190839694656489 | PPT1//SIRT5//SIRT6//PRDM5//DYRK1A// |
| GO:0051983 | regulation of chromosome segregation | Biological process | 5 | 107 | 262 | 18866 | 3.36484269101805 | 0.0168020421990196 | 0.380146204752818 | 1.77463792887753 | 0.0190839694656489 | HECW2//DDX11//CTCF//DYNC1H1//PUM1// |
| GO:2001237 | negative regulation of extrinsic apoptotic signaling pathway | Biological process | 5 | 107 | 262 | 18866 | 3.36484269101805 | 0.0168020421990196 | 0.380146204752818 | 1.77463792887753 | 0.0190839694656489 | GSK3B//ICAM1//EYA3//GCLM//THBS1// |
| GO:0007043 | cell-cell junction assembly | Biological process | 6 | 147 | 262 | 18866 | 2.93908708521577 | 0.0168899360885184 | 0.380913926034669 | 1.77237199379526 | 0.0229007633587786 | NFASC//PIP5K1C//ACTB//WDR1//PARD3//STRN// |
| GO:0001667 | ameboidal-type cell migration | Biological process | 13 | 481 | 262 | 18866 | 1.94615225912936 | 0.0173672036132769 | 0.387692512858442 | 1.76027010401064 | 0.049618320610687 | CORO1C//OVOL2//MIA3//BMPR2//THBS1//MCC//PTPRG//DOCK1//PLCG2//MYH9//NF1//STARD13//LPXN// |
| GO:1901564 | organonitrogen compound metabolic process | Biological process | 112 | 6846 | 262 | 18866 | 1.17803899530121 | 0.0175131992634973 | 0.387692512858442 | 1.75663451093726 | 0.427480916030534 | ACTB//MTR//NF1//TAOK3//PSMB5//SRPK2//THBS1//HUWE1//NEDD4L//WSB1//MDM2//RNF111//HECW2//UBE3B//CLN6//CORO1C//TERF2IP//TFRC//MOB2//PPT1//RPL18A//GAPDH//HK1//NUP155//PDE4D//DCTD//IGF2BP3//LARP4//RPL13A//RPS27L//RPL37A//RPS14//LSM14A//CNOT11//UBL5//RPN1//RPN2//DYRK1A//LATS2//GSK3B//PHKG1//BCR//BMPR2//TRIO//NEK9//PHLPP2//PTPRG//SIRT5//SIRT6//GALNT1//GALNT7//PITRM1//PRSS57//DPP8//CFAP44//PAPPA2//TMEM59//MYH9//ADAM19//USP42//GCLM//THAP4//PCCB//SPAG9//BCKDHA//ABCD4//ENC1//PARD3//LDLR//G2E3//DCAF17//TRIM24//KLHL21//TRRAP//PRDM5//EYA3//RANGAP1//CAPRIN1//CNOT8//SETD2//TTLL11//CLK1//SMG1//EFEMP1//PKDCC//ZDHHC17//CRAT//ELOVL6//CTCF//RNF4//TADA2A//OSBPL10//MBOAT2//DDR1//FAF1//ARL6IP1//PCBP2//RNF216//MIA3//RAB2A//SPI1//KRT17//NAT10//HABP4//ICAM1//TMF1//FTO//PUM1//GNAI2//NEMF//IFI16//OPHN1// |
| GO:0032269 | negative regulation of cellular protein metabolic process | Biological process | 25 | 1149 | 262 | 18866 | 1.56674572645314 | 0.017601317066217 | 0.387692512858442 | 1.75445483370919 | 0.0954198473282443 | CORO1C//TERF2IP//NF1//TAOK3//THBS1//PARD3//GAPDH//MDM2//TMEM59//IGF2BP3//RPL13A//CAPRIN1//ENC1//CNOT8//GSK3B//PDE4D//RNF4//ARL6IP1//LATS2//FTO//PUM1//SPI1//OPHN1//GNAI2//IFI16// |
| GO:0006403 | RNA localization | Biological process | 8 | 236 | 262 | 18866 | 2.44093673178936 | 0.0176099762001863 | 0.387692512858442 | 1.75424123097961 | 0.0305343511450382 | CDC40//NUP155//SMG1//ALKBH5//FIP1L1//SETD2//IGF2BP3//RANBP17// |
| GO:0030029 | actin filament-based process | Biological process | 19 | 807 | 262 | 18866 | 1.69534701136052 | 0.0178029398630083 | 0.387692512858442 | 1.74950827517101 | 0.0725190839694656 | CORO1C//MYO19//DOCK2//FLNB//PIP5K1C//NF1//OPHN1//BCR//MOB2//STARD13//WDR1//MYH9//MYO9B//ICAM1//SH3PXD2B//PDE4D//NEDD4L//NUP155//ACTB// |
| GO:0006139 | nucleobase-containing compound metabolic process | Biological process | 103 | 6225 | 262 | 18866 | 1.19145160795855 | 0.0178044445221667 | 0.387692512858442 | 1.74947157125797 | 0.393129770992366 | KLF11//CTCF//TCFL5//PRDM5//NEDD4L//IFI16//RBPJ//MDM2//NFATC2//SIRT6//OVOL2//RBL1//BACH2//RPS14//SPI1//NR2C2//NRIP1//KLF7//GTF2IRD1//NCOR2//NAT10//SMG1//RPL13A//RPL18A//RPL37A//SRPK2//CNOT11//CNOT8//TRA2B//DDX17//DYRK1A//PCBP2//CDC40//CCAR1//UBL5//FIP1L1//RCL1//NOL10//TERF2IP//MTO1//UGP2//GAPDH//HK1//NUP155//PDE4D//DCTD//POLI//DDX11//PDS5B//TAOK3//TRRAP//ATRIP//HUWE1//SETD2//EYA3//ALKBH5//FTO//KPNB1//GPBP1L1//BRF1//ZNF483//DNM2//EFEMP1//GATAD1//ZNF236//ZNF646//ZNF652//LCORL//MEIS1//THAP4//PLAGL1//RFX2//NPAS3//TADA2A//TEX10//PHRF1//TAF15//TRIM24//GTF3C5//HABP4//FAF1//RPPH1//SAGE1//PUM1//ELOVL6//CLK1//IGF2BP3//PSMB5//DDX6//TFRC//SCMH1//CRLF3//RNF111//RNF4//FANK1//BMPR2//TMF1//PPT1//ICAM1//MORC2//CHCHD10//LPXN//SLC23A2// |
| GO:0051051 | negative regulation of transport | Biological process | 13 | 483 | 262 | 18866 | 1.93809365764228 | 0.0179053189901866 | 0.387692512858442 | 1.7470179374589 | 0.049618320610687 | THBS1//ANKRD27//NF1//PKDCC//BCR//GNAI2//SIRT6//RANGAP1//ICAM1//KLF7//RAB11FIP5//NEDD4L//HECW2// |
| GO:0042159 | lipoprotein catabolic process | Biological process | 2 | 15 | 262 | 18866 | 9.60101781170483 | 0.0179073745099855 | 0.387692512858442 | 1.74696808357639 | 0.00763358778625954 | PPT1//LDLR// |
| GO:0043518 | negative regulation of DNA damage response, signal transduction by p53 class mediator | Biological process | 2 | 15 | 262 | 18866 | 9.60101781170483 | 0.0179073745099855 | 0.387692512858442 | 1.74696808357639 | 0.00763358778625954 | DYRK1A//MDM2// |
| GO:0048712 | negative regulation of astrocyte differentiation | Biological process | 2 | 15 | 262 | 18866 | 9.60101781170483 | 0.0179073745099855 | 0.387692512858442 | 1.74696808357639 | 0.00763358778625954 | LDLR//NF1// |
| GO:0090148 | membrane fission | Biological process | 2 | 15 | 262 | 18866 | 9.60101781170483 | 0.0179073745099855 | 0.387692512858442 | 1.74696808357639 | 0.00763358778625954 | DNM2//CORO1C// |
| GO:1902043 | positive regulation of extrinsic apoptotic signaling pathway via death domain receptors | Biological process | 2 | 15 | 262 | 18866 | 9.60101781170483 | 0.0179073745099855 | 0.387692512858442 | 1.74696808357639 | 0.00763358778625954 | FAF1//THBS1// |
| GO:0046777 | protein autophosphorylation | Biological process | 8 | 237 | 262 | 18866 | 2.43063742068477 | 0.0180143446214832 | 0.387692512858442 | 1.74438153328889 | 0.0305343511450382 | DYRK1A//DDR1//CLK1//SMG1//GSK3B//TAOK3//BCR//TRIM24// |
| GO:0051050 | positive regulation of transport | Biological process | 21 | 921 | 262 | 18866 | 1.64186786682249 | 0.0180552242579092 | 0.387692512858442 | 1.74339711297697 | 0.0801526717557252 | ARL6IP1//PLCG2//RPH3AL//DYNC1H1//GNAI2//NEDD4L//GSK3B//MDM2//PPT1//GOLPH3L//GAPDH//DNM2//DOCK2//BCR//ACTB//ATP8A1//CHCHD10//STAC//HUWE1//UBL5//TMF1// |
| GO:0006613 | cotranslational protein targeting to membrane | Biological process | 5 | 109 | 262 | 18866 | 3.30310245815533 | 0.0180616541681428 | 0.387692512858442 | 1.74324247754419 | 0.0190839694656489 | RPL13A//RPL18A//RPL37A//RPS14//ARL6IP1// |
| GO:0032268 | regulation of cellular protein metabolic process | Biological process | 51 | 2754 | 262 | 18866 | 1.33347469607012 | 0.0180692501388904 | 0.387692512858442 | 1.74305986997915 | 0.194656488549618 | ACTB//THBS1//CORO1C//TERF2IP//TFRC//MOB2//LSM14A//CNOT11//RPS14//NF1//TAOK3//RPS27L//SPAG9//PARD3//BMPR2//GAPDH//MDM2//TMEM59//IGF2BP3//RPL13A//CAPRIN1//ENC1//CNOT8//CTCF//HUWE1//RNF111//GSK3B//PDE4D//RNF4//TADA2A//ARL6IP1//LARP4//KRT17//NAT10//LATS2//FAF1//CLN6//HABP4//ICAM1//TMF1//FTO//PUM1//GNAI2//NEMF//SPI1//DYRK1A//IFI16//SIRT6//OPHN1//MYH9//LDLR// |
| GO:0006909 | phagocytosis | Biological process | 11 | 382 | 262 | 18866 | 2.07351824467447 | 0.0182188045168554 | 0.389716791165098 | 1.73948012400225 | 0.0419847328244275 | DOCK1//MYH9//PLCG2//ACTB//THBS1//DNM2//DOCK2//BCR//PIP5K1C//CORO1C//LDLR// |
| GO:0018209 | peptidyl-serine modification | Biological process | 10 | 333 | 262 | 18866 | 2.16239139903262 | 0.018386710575152 | 0.39153062774681 | 1.73549596009529 | 0.0381679389312977 | CLK1//DYRK1A//SMG1//LATS2//GSK3B//SRPK2//GALNT1//PDE4D//TERF2IP//TFRC// |
| GO:0034249 | negative regulation of cellular amide metabolic process | Biological process | 8 | 238 | 262 | 18866 | 2.42042465841298 | 0.018425338683732 | 0.39153062774681 | 1.73458452044997 | 0.0305343511450382 | IGF2BP3//RPL13A//GAPDH//CAPRIN1//ENC1//CNOT8//FTO//PUM1// |
| GO:1905039 | carboxylic acid transmembrane transport | Biological process | 6 | 150 | 262 | 18866 | 2.88030534351145 | 0.0184699956140654 | 0.39153062774681 | 1.73353320768848 | 0.0229007633587786 | ARL6IP1//THBS1//SLC23A2//ABCD4//SLC15A4//SLC3A2// |
| GO:0072401 | signal transduction involved in DNA integrity checkpoint | Biological process | 4 | 73 | 262 | 18866 | 3.94562375823486 | 0.0187379850170515 | 0.394530727050822 | 1.72727711264918 | 0.0152671755725191 | MDM2//PLAGL1//CNOT11//CNOT8// |
| GO:0072422 | signal transduction involved in DNA damage checkpoint | Biological process | 4 | 73 | 262 | 18866 | 3.94562375823486 | 0.0187379850170515 | 0.394530727050822 | 1.72727711264918 | 0.0152671755725191 | MDM2//PLAGL1//CNOT11//CNOT8// |
| GO:0046578 | regulation of Ras protein signal transduction | Biological process | 7 | 194 | 262 | 18866 | 2.59821358306445 | 0.0189415346640871 | 0.394530727050822 | 1.72258483688799 | 0.0267175572519084 | DNM2//NF1//MYO9B//OPHN1//BCR//ARHGEF28//STARD13// |
| GO:0060284 | regulation of cell development | Biological process | 22 | 983 | 262 | 18866 | 1.61156453604405 | 0.0190018938128317 | 0.394530727050822 | 1.72120311318004 | 0.083969465648855 | NF1//GSK3B//MOB2//ENC1//MDM2//PTPRG//DNM2//DDX6//MEIS1//SPAG9//WDR1//NEDD4L//HECW2//BMPR2//HOOK3//TIAM2//CAPRIN1//ANKRD27//PDE3A//LDLR//CORO1C//DOCK1// |
| GO:0007030 | Golgi organization | Biological process | 6 | 151 | 262 | 18866 | 2.86123047368687 | 0.0190184117633693 | 0.394530727050822 | 1.72082575402348 | 0.0229007633587786 | DNM2//PDE4DIP//HUWE1//PLEKHM2//GOLPH3L//RAB2A// |
| GO:1903825 | organic acid transmembrane transport | Biological process | 6 | 151 | 262 | 18866 | 2.86123047368687 | 0.0190184117633693 | 0.394530727050822 | 1.72082575402348 | 0.0229007633587786 | ARL6IP1//THBS1//SLC23A2//ABCD4//SLC15A4//SLC3A2// |
| GO:0044770 | cell cycle phase transition | Biological process | 16 | 647 | 262 | 18866 | 1.78071427728683 | 0.0190224513239895 | 0.394530727050822 | 1.72073351852981 | 0.0610687022900763 | LATS2//NASP//CRLF3//KLF11//SDCCAG8//DYNC1H1//DNM2//MDM2//PLAGL1//CNOT11//CNOT8//TAOK3//PSMB5//HECW2//RPS27L//RBL1// |
| GO:0051246 | regulation of protein metabolic process | Biological process | 53 | 2891 | 262 | 18866 | 1.32009843657996 | 0.0190586454064783 | 0.394530727050822 | 1.71990797008792 | 0.202290076335878 | ACTB//THBS1//CORO1C//TERF2IP//TFRC//MOB2//LSM14A//CNOT11//RPS14//NF1//TAOK3//RPS27L//SPAG9//PARD3//BMPR2//GAPDH//MDM2//TMEM59//IGF2BP3//RPL13A//CAPRIN1//ENC1//CNOT8//CTCF//HUWE1//RNF111//GSK3B//PDE4D//RNF4//TADA2A//FAF1//NEDD4L//ARL6IP1//LARP4//KRT17//NAT10//HECW2//LATS2//CLN6//HABP4//ICAM1//LDLR//TMF1//FTO//PUM1//GNAI2//NEMF//SPI1//DYRK1A//IFI16//SIRT6//OPHN1//MYH9// |
| GO:0006734 | NADH metabolic process | Biological process | 3 | 41 | 262 | 18866 | 5.2688512381307 | 0.0191428961254414 | 0.395116092834769 | 1.71799235725148 | 0.0114503816793893 | GAPDH//HK1//MDH1// |
| GO:0051049 | regulation of transport | Biological process | 36 | 1821 | 262 | 18866 | 1.42354465082938 | 0.0193374287391977 | 0.39772788439064 | 1.71360127371819 | 0.137404580152672 | ARL6IP1//PLCG2//THBS1//SETD2//PDE4D//RPH3AL//ACTB//OPHN1//DYNC1H1//NEDD4L//NALCN//ANKRD27//GNAI2//NF1//PKDCC//BCR//SIRT6//RANGAP1//GSK3B//MDM2//PPT1//GOLPH3L//GAPDH//DNM2//DOCK2//ICAM1//WDR1//ATP8A1//KLF7//RAB11FIP5//CHCHD10//STAC//HUWE1//UBL5//HECW2//TMF1// |
| GO:0098732 | macromolecule deacylation | Biological process | 5 | 111 | 262 | 18866 | 3.24358709854893 | 0.0193821210129452 | 0.39772788439064 | 1.71259869922461 | 0.0190839694656489 | PPT1//SIRT5//SIRT6//PRDM5//DYRK1A// |
| GO:0072395 | signal transduction involved in cell cycle checkpoint | Biological process | 4 | 74 | 262 | 18866 | 3.89230451825872 | 0.0195988290119088 | 0.400702837176887 | 1.70776987603427 | 0.0152671755725191 | MDM2//PLAGL1//CNOT11//CNOT8// |
| GO:0072593 | reactive oxygen species metabolic process | Biological process | 9 | 288 | 262 | 18866 | 2.25023854961832 | 0.0196406263866274 | 0.400702837176887 | 1.70684466563676 | 0.0343511450381679 | SH3PXD2B//GNAI2//HBG2//DNM2//ICAM1//SIRT5//BCR//THBS1//SESN1// |
| GO:0046822 | regulation of nucleocytoplasmic transport | Biological process | 5 | 112 | 262 | 18866 | 3.21462649945474 | 0.0200655013946718 | 0.404402781708482 | 1.697549983689 | 0.0190839694656489 | SETD2//NF1//RANGAP1//GSK3B//MDM2// |
| GO:0030048 | actin filament-based movement | Biological process | 6 | 153 | 262 | 18866 | 2.82382876814848 | 0.0201484049047457 | 0.404402781708482 | 1.69575933009297 | 0.0229007633587786 | MYO19//PDE4D//NEDD4L//NUP155//MYH9//MYO9B// |
| GO:0048820 | hair follicle maturation | Biological process | 2 | 16 | 262 | 18866 | 9.00095419847328 | 0.020280292495368 | 0.404402781708482 | 1.69292578562035 | 0.00763358778625954 | RBPJ//NF1// |
| GO:0071391 | cellular response to estrogen stimulus | Biological process | 2 | 16 | 262 | 18866 | 9.00095419847328 | 0.020280292495368 | 0.404402781708482 | 1.69292578562035 | 0.00763358778625954 | MDM2//TRIM24// |
| GO:0071732 | cellular response to nitric oxide | Biological process | 2 | 16 | 262 | 18866 | 9.00095419847328 | 0.020280292495368 | 0.404402781708482 | 1.69292578562035 | 0.00763358778625954 | DNM2//MTR// |
| GO:0086103 | G protein-coupled receptor signaling pathway involved in heart process | Biological process | 2 | 16 | 262 | 18866 | 9.00095419847328 | 0.020280292495368 | 0.404402781708482 | 1.69292578562035 | 0.00763358778625954 | PDE4D//GNAI2// |
| GO:1903358 | regulation of Golgi organization | Biological process | 2 | 16 | 262 | 18866 | 9.00095419847328 | 0.020280292495368 | 0.404402781708482 | 1.69292578562035 | 0.00763358778625954 | DNM2//PDE4DIP// |
| GO:1904262 | negative regulation of TORC1 signaling | Biological process | 2 | 16 | 262 | 18866 | 9.00095419847328 | 0.020280292495368 | 0.404402781708482 | 1.69292578562035 | 0.00763358778625954 | SESN1//ITFG2// |
| GO:0072595 | maintenance of protein localization in organelle | Biological process | 3 | 42 | 262 | 18866 | 5.14340239912759 | 0.0204086457626871 | 0.405815860391009 | 1.69018581239833 | 0.0114503816793893 | RANGAP1//CHCHD10//HK1// |
| GO:0048844 | artery morphogenesis | Biological process | 4 | 75 | 262 | 18866 | 3.84040712468193 | 0.0204837285739419 | 0.406164719110831 | 1.68859098755451 | 0.0152671755725191 | RBPJ//BMPR2//LDLR//NF1// |
| GO:0000209 | protein polyubiquitination | Biological process | 10 | 340 | 262 | 18866 | 2.11787157611136 | 0.0208969804015311 | 0.41319827634288 | 1.6799164645921 | 0.0381679389312977 | RNF4//NEDD4L//RNF216//HUWE1//WSB1//MDM2//RNF111//PSMB5//HECW2//UBE3B// |
| GO:1901360 | organic cyclic compound metabolic process | Biological process | 109 | 6678 | 262 | 18866 | 1.17532675367905 | 0.0210151467584947 | 0.414374080916799 | 1.67746757276861 | 0.416030534351145 | KLF11//CTCF//TCFL5//PRDM5//NEDD4L//IFI16//RBPJ//MDM2//NFATC2//SIRT6//OVOL2//RBL1//BACH2//RPS14//SPI1//NR2C2//NRIP1//KLF7//GTF2IRD1//NCOR2//NAT10//SMG1//RPL13A//RPL18A//RPL37A//SRPK2//CNOT11//CNOT8//TRA2B//DDX17//DYRK1A//PCBP2//CDC40//CCAR1//UBL5//FIP1L1//RCL1//NOL10//TERF2IP//MTO1//UGP2//GAPDH//HK1//NUP155//SLC23A2//PDE4D//DCTD//POLI//DDX11//PDS5B//TAOK3//TRRAP//ATRIP//HUWE1//SETD2//EYA3//ALKBH5//FTO//KPNB1//GPBP1L1//BRF1//ZNF483//DNM2//EFEMP1//GATAD1//ZNF236//ZNF646//ZNF652//LCORL//MEIS1//THAP4//PLAGL1//RFX2//NPAS3//TADA2A//TEX10//PHRF1//TAF15//TRIM24//GTF3C5//HABP4//PCCB//FAF1//RPPH1//LDLR//CLN6//MTR//ABCD4//SAGE1//PUM1//ELOVL6//CYP24A1//CLK1//IGF2BP3//PSMB5//DDX6//TFRC//SCMH1//CRLF3//RNF111//RNF4//FANK1//BMPR2//TMF1//PPT1//ICAM1//MORC2//CHCHD10//LPXN// |
| GO:0071495 | cellular response to endogenous stimulus | Biological process | 29 | 1406 | 262 | 18866 | 1.48522146091451 | 0.0210847032041048 | 0.414587520662328 | 1.67603250789203 | 0.110687022900763 | PARD3//LPXN//GNB1//GSK3B//AP3S1//ATP6V0B//LATS2//BMPR2//RNF111//THBS1//DNM2//RBPJ//DDX17//TMF1//SLIT3//GCLM//GNAI2//NCOR2//SESN1//MDM2//ICAM1//PDE3A//TRIM24//OVOL2//PDE4D//ACTB//KLF11//RANGAP1//DDX11// |
| GO:0003279 | cardiac septum development | Biological process | 5 | 114 | 262 | 18866 | 3.15822954332396 | 0.021479179433678 | 0.421170910062036 | 1.66798231395166 | 0.0190839694656489 | BMPR2//DNM2//MDM2//RBPJ//SLIT3// |
| GO:0001953 | negative regulation of cell-matrix adhesion | Biological process | 3 | 43 | 262 | 18866 | 5.02378838984555 | 0.021719396618841 | 0.422327778526278 | 1.66315224394266 | 0.0114503816793893 | CORO1C//THBS1//NF1// |
| GO:0030521 | androgen receptor signaling pathway | Biological process | 3 | 43 | 262 | 18866 | 5.02378838984555 | 0.021719396618841 | 0.422327778526278 | 1.66315224394266 | 0.0114503816793893 | NCOR2//DDX17//TMF1// |
| GO:0045124 | regulation of bone resorption | Biological process | 3 | 43 | 262 | 18866 | 5.02378838984555 | 0.021719396618841 | 0.422327778526278 | 1.66315224394266 | 0.0114503816793893 | GPR137B//TFRC//NF1// |
| GO:0006950 | response to stress | Biological process | 72 | 4162 | 262 | 18866 | 1.24568707792422 | 0.0218550697386543 | 0.422327778526278 | 1.66044780335578 | 0.274809160305344 | ATRIP//TRA2B//VDAC3//NF1//ALKBH5//THBS1//MYH9//IFI16//PLCG2//PSMB5//RBPJ//ICAM1//SIRT5//POLI//DDX11//PDS5B//SMG1//TAOK3//TRRAP//HUWE1//SIRT6//SETD2//EYA3//FTO//HK1//MORC2//NFATC2//MDM2//PLAGL1//CNOT11//CNOT8//RPS27L//GCLM//SLC23A2//GSK3B//GABARAP//SPAG9//DOCK1//HBG2//AMBRA1//BMPR2//PAPPA2//TERF2IP//GNB1//CANX//MTR//SESN1//ITFG2//STAC//LSM14A//MIA3//TMF1//DYRK1A//DDR1//PCBP2//SRPK2//RANGAP1//RNF216//BCR//LDLR//GAPDH//DDX17//IFI30//ACTB//RNF111//FLNB//RPL13A//RCSD1//DNM2//NUP155//PUM1//RBL1// |
| GO:0043484 | regulation of RNA splicing | Biological process | 6 | 156 | 262 | 18866 | 2.76952436876101 | 0.0219276628378285 | 0.422327778526278 | 1.65900765517726 | 0.0229007633587786 | DDX17//DYRK1A//TRA2B//HABP4//SRPK2//CLK1// |
| GO:0030705 | cytoskeleton-dependent intracellular transport | Biological process | 7 | 200 | 262 | 18866 | 2.52026717557252 | 0.0219566986025058 | 0.422327778526278 | 1.65843295959991 | 0.0267175572519084 | AP3S1//DYNC1H1//MYO19//DYNLRB1//RABL2B//TUBA1B//HOOK3// |
| GO:1901700 | response to oxygen-containing compound | Biological process | 34 | 1715 | 262 | 18866 | 1.42755658424766 | 0.0219838312238363 | 0.422327778526278 | 1.65789661881077 | 0.129770992366412 | TRA2B//GNB1//GSK3B//GNAI2//AP3S1//ATP6V0B//COL6A2//THBS1//ICAM1//NCOR2//PLCG2//CYP24A1//NASP//RAB11FIP5//DNM2//MDM2//GNAI1//SLIT3//KLF7//TRIM24//PDE4D//BCR//SESN1//PDE3A//GCLM//ZNF236//SPI1//NRIP1//LDLR//MTR//ACTB//KLF11//RANGAP1//DDX11// |
| GO:0048667 | cell morphogenesis involved in neuron differentiation | Biological process | 15 | 604 | 262 | 18866 | 1.78826904605429 | 0.0220168044337258 | 0.422327778526278 | 1.65724571522394 | 0.0572519083969466 | PARD3//ACTB//KLF7//NFASC//OPHN1//SLIT3//TRIO//DNM2//GSK3B//NEDD4L//HECW2//BMPR2//TIAM2//CAPRIN1//ANKRD27// |
| GO:0051225 | spindle assembly | Biological process | 5 | 115 | 262 | 18866 | 3.13076667772984 | 0.0222097161955573 | 0.423103328705402 | 1.65345699100087 | 0.0190839694656489 | RNF4//KPNB1//DYNC1H1//TUBGCP2//STARD9// |
| GO:0060411 | cardiac septum morphogenesis | Biological process | 4 | 77 | 262 | 18866 | 3.74065629027461 | 0.0223263226486628 | 0.423103328705402 | 1.65118280334644 | 0.0152671755725191 | BMPR2//RBPJ//SLIT3//MDM2// |
| GO:0016358 | dendrite development | Biological process | 8 | 247 | 262 | 18866 | 2.33223104737769 | 0.0224313574555662 | 0.423103328705402 | 1.64914444386584 | 0.0305343511450382 | KLF7//NEDD4L//GSK3B//HECW2//CAPRIN1//ANKRD27//TRAPPC4//STRN// |
| GO:0010770 | positive regulation of cell morphogenesis involved in differentiation | Biological process | 6 | 157 | 262 | 18866 | 2.75188408615744 | 0.0225435739498404 | 0.423103328705402 | 1.64697723187231 | 0.0229007633587786 | BMPR2//TIAM2//CAPRIN1//ANKRD27//DNM2//DOCK1// |
| GO:1901699 | cellular response to nitrogen compound | Biological process | 17 | 716 | 262 | 18866 | 1.70967845110666 | 0.0226037585349923 | 0.423103328705402 | 1.64581934070702 | 0.0648854961832061 | GNB1//GSK3B//GNAI2//AP3S1//ATP6V0B//SESN1//MDM2//ICAM1//PDE3A//DNM2//MTR//PDE4D//DDX11//ACTB//GCLM//KLF11//RANGAP1// |
| GO:0008219 | cell death | Biological process | 43 | 2281 | 262 | 18866 | 1.35744333374608 | 0.0227549964392289 | 0.423103328705402 | 1.64292322811731 | 0.16412213740458 | KPNB1//FAF1//DOCK1//ARL6IP1//PLAGL1//RNF216//DPP8//G2E3//CCAR1//KLF11//RPS27L//GABARAP//NF1//GCLM//GNB1//SIRT5//AMBRA1//IFI16//USP42//TRIM24//FANK1//DNM2//TIAM2//LATS2//TRIO//GSK3B//PDE3A//PPT1//TFRC//THBS1//TMF1//PLCG2//MDM2//SRPK2//GAPDH//SLIT3//KRT17//BMPR2//CHCHD10//ICAM1//SPI1//GNAI2//EYA3// |
| GO:0030575 | nuclear body organization | Biological process | 2 | 17 | 262 | 18866 | 8.47148630444544 | 0.0227764931163129 | 0.423103328705402 | 1.64251314318393 | 0.00763358778625954 | HABP4//SRPK2// |
| GO:0035020 | regulation of Rac protein signal transduction | Biological process | 2 | 17 | 262 | 18866 | 8.47148630444544 | 0.0227764931163129 | 0.423103328705402 | 1.64251314318393 | 0.00763358778625954 | NF1//DNM2// |
| GO:0042953 | lipoprotein transport | Biological process | 2 | 17 | 262 | 18866 | 8.47148630444544 | 0.0227764931163129 | 0.423103328705402 | 1.64251314318393 | 0.00763358778625954 | ZDHHC17//MIA3// |
| GO:0046459 | short-chain fatty acid metabolic process | Biological process | 2 | 17 | 262 | 18866 | 8.47148630444544 | 0.0227764931163129 | 0.423103328705402 | 1.64251314318393 | 0.00763358778625954 | PCCB//CRAT// |
| GO:0099558 | maintenance of synapse structure | Biological process | 2 | 17 | 262 | 18866 | 8.47148630444544 | 0.0227764931163129 | 0.423103328705402 | 1.64251314318393 | 0.00763358778625954 | OPHN1//CHCHD10// |
| GO:2000136 | regulation of cell proliferation involved in heart morphogenesis | Biological process | 2 | 17 | 262 | 18866 | 8.47148630444544 | 0.0227764931163129 | 0.423103328705402 | 1.64251314318393 | 0.00763358778625954 | BMPR2//RBPJ// |
| GO:0031670 | cellular response to nutrient | Biological process | 3 | 44 | 262 | 18866 | 4.90961138098543 | 0.0230751711350031 | 0.425294081049574 | 1.63685506938279 | 0.0114503816793893 | CYP24A1//TRIM24//MDM2// |
| GO:0045197 | establishment or maintenance of epithelial cell apical/basal polarity | Biological process | 3 | 44 | 262 | 18866 | 4.90961138098543 | 0.0230751711350031 | 0.425294081049574 | 1.63685506938279 | 0.0114503816793893 | OPHN1//WDR1//PARD3// |
| GO:0060612 | adipose tissue development | Biological process | 3 | 44 | 262 | 18866 | 4.90961138098543 | 0.0230751711350031 | 0.425294081049574 | 1.63685506938279 | 0.0114503816793893 | KLF7//SH3PXD2B//FTO// |
| GO:0071417 | cellular response to organonitrogen compound | Biological process | 16 | 663 | 262 | 18866 | 1.73774078039906 | 0.0232497548306175 | 0.426395963735043 | 1.6335816223986 | 0.0610687022900763 | GNB1//GSK3B//AP3S1//ATP6V0B//GNAI2//SESN1//MDM2//ICAM1//PDE3A//PDE4D//ACTB//GCLM//KLF11//DNM2//RANGAP1//DDX11// |
| GO:0050896 | response to stimulus | Biological process | 147 | 9398 | 262 | 18866 | 1.1263164649292 | 0.0232557651279206 | 0.426395963735043 | 1.63346936738155 | 0.561068702290076 | ATRIP//NF1//TAOK3//PSMB5//SRPK2//THBS1//TRA2B//VDAC3//ALKBH5//MYH9//GNAI2//ICAM1//DNM2//IFI16//PLCG2//DOCK2//ITFG2//RBPJ//SIRT5//POLI//DDX11//PDS5B//SMG1//TRRAP//HUWE1//SIRT6//SETD2//EYA3//FTO//HK1//DPP8//MORC2//NFATC2//MDM2//PLAGL1//CNOT11//CNOT8//RPS27L//GCLM//SLC23A2//GSK3B//GABARAP//DOCK1//PHLPP2//FLNB//CORO1C//GDI2//GNB1//MCC//OPHN1//PDE3A//PDE4D//PI4KA//RANGAP1//BCR//LMBR1//STAC//STARD13//LPXN//PTPRG//DDR1//EFEMP1//BMPR2//PARD3//TRIO//TIAM2//GNAI1//EVC//SPAG9//MYO9B//NFASC//SLIT3//KLF7//HBG2//DYNLRB1//AP3S1//ATP6V0B//AMBRA1//PAPPA2//COL6A2//LATS2//NEDD4L//NCOR2//TERF2IP//AKAP7//PIP5K1C//GTF2IRD1//STRN//RNF111//DDX17//TMF1//CANX//MTR//KPNB1//CYP24A1//NASP//WDFY1//SESN1//AFF3//ARHGEF28//FAM120B//WSB1//TFRC//ANKRD27//RAB11FIP5//ACTB//LSM14A//MIA3//RPN2//RPH3AL//ZDHHC17//ATP8A1//SLC15A4//DYNC1H1//SLC3A2//DYRK1A//PCBP2//CRLF3//RNF4//RNF216//LDLR//GAPDH//RALGAPA2//IFI30//OVOL2//SPI1//TRIM24//HABP4//ZNF236//RPL13A//TUBA1B//NRIP1//RCSD1//BACH2//RPL18A//NUP155//PUM1//CHCHD10//KLF11//TAF15//FAF1//GPR137B//PRDM5//ABCD4//RFX2//RBL1//PPT1// |
| GO:0010506 | regulation of autophagy | Biological process | 10 | 347 | 262 | 18866 | 2.07514794201113 | 0.0236488858142383 | 0.432480530991472 | 1.62618931565034 | 0.0381679389312977 | SMG1//GSK3B//AMBRA1//TMEM59//SESN1//GAPDH//ATP6V0B//HUWE1//IFI16//GPR137B// |
| GO:0007005 | mitochondrion organization | Biological process | 14 | 556 | 262 | 18866 | 1.81314185292987 | 0.0238468824735302 | 0.434974530699353 | 1.62256838865905 | 0.0534351145038168 | DNM2//GABARAP//AMBRA1//PITRM1//GCLM//HUWE1//TFRC//GSK3B//NDUFB2//TTC19//MYO19//CHCHD10//UBL5//SIRT5// |
| GO:0006259 | DNA metabolic process | Biological process | 21 | 948 | 262 | 18866 | 1.59510580732438 | 0.0239197931242089 | 0.435179947587089 | 1.62124258076235 | 0.0801526717557252 | TERF2IP//POLI//DDX11//PDS5B//SMG1//TAOK3//TRRAP//ATRIP//HUWE1//SIRT6//SETD2//EYA3//CTCF//ALKBH5//FTO//KPNB1//NAT10//SPI1//TFRC//RNF111//BMPR2// |
| GO:1901991 | negative regulation of mitotic cell cycle phase transition | Biological process | 8 | 251 | 262 | 18866 | 2.29506401873422 | 0.0243955963210432 | 0.43963311591775 | 1.61268856161512 | 0.0305343511450382 | MDM2//PLAGL1//CNOT11//CNOT8//TAOK3//PSMB5//RPS27L//RBL1// |
| GO:0003197 | endocardial cushion development | Biological process | 3 | 45 | 262 | 18866 | 4.80050890585242 | 0.024475961829675 | 0.43963311591775 | 1.61126023275821 | 0.0114503816793893 | RBPJ//MDM2//BMPR2// |
| GO:0051293 | establishment of spindle localization | Biological process | 3 | 45 | 262 | 18866 | 4.80050890585242 | 0.024475961829675 | 0.43963311591775 | 1.61126023275821 | 0.0114503816793893 | KPNB1//MYH9//DYNC1H1// |
| GO:0060412 | ventricular septum morphogenesis | Biological process | 3 | 45 | 262 | 18866 | 4.80050890585242 | 0.024475961829675 | 0.43963311591775 | 1.61126023275821 | 0.0114503816793893 | RBPJ//SLIT3//BMPR2// |
| GO:0072583 | clathrin-dependent endocytosis | Biological process | 3 | 45 | 262 | 18866 | 4.80050890585242 | 0.024475961829675 | 0.43963311591775 | 1.61126023275821 | 0.0114503816793893 | DNM2//PIP5K1C//CANX// |
| GO:0051336 | regulation of hydrolase activity | Biological process | 27 | 1306 | 262 | 18866 | 1.48867236360661 | 0.0249762431438315 | 0.441485196543038 | 1.60247288640248 | 0.103053435114504 | RPS27L//GAPDH//DDX11//PPT1//GSK3B//NF1//GPR137B//ARL6IP1//MDM2//THBS1//AGAP1//DOCK1//DOCK2//GDI2//ICAM1//MYO9B//OPHN1//ANKRD27//STARD13//TBC1D1//CORO1C//TIAM2//RALGAPA2//RANGAP1//BCR//IFI16//GNAI2// |
| GO:0050789 | regulation of biological process | Biological process | 187 | 12364 | 262 | 18866 | 1.08908342614979 | 0.0251390239611217 | 0.441485196543038 | 1.59965158808712 | 0.713740458015267 | ATRIP//ACTB//KLF11//CTCF//TCFL5//PRDM5//NEDD4L//IFI16//RBPJ//MDM2//NFATC2//SIRT6//OVOL2//RBL1//BACH2//RPS14//SPI1//NR2C2//NRIP1//KLF7//GTF2IRD1//NCOR2//NF1//TAOK3//PSMB5//SRPK2//SMG1//RPL13A//RPL18A//RPL37A//THBS1//CNOT11//CNOT8//DDX17//DYRK1A//TRA2B//PAPPA2//DDR1//TMF1//ICAM1//CORO1C//TERF2IP//TFRC//MOB2//BMPR2//GSK3B//GNAI2//PDE4D//DNM2//ARL6IP1//PLCG2//SIAE//MIA3//BCR//NUP155//ZNF483//EFEMP1//SETD2//CCAR1//GATAD1//GPBP1L1//ZNF236//TRRAP//ZNF646//ZNF652//LCORL//MEIS1//THAP4//PLAGL1//RFX2//NPAS3//TADA2A//LSM14A//RPS27L//LPXN//DOCK1//PHLPP2//FLNB//GDI2//GNB1//MCC//OPHN1//PDE3A//PI4KA//RANGAP1//LMBR1//STAC//STARD13//PTPRG//PARD3//TRIO//TIAM2//GNAI1//EVC//MYH9//FAF1//SPAG9//DOCK2//MYO9B//PDS5B//AMBRA1//SLIT3//STRN//TRIM24//AP3S1//ATP6V0B//FBLIM1//WDR1//GABARAP//LATS2//SDCCAG8//DYNC1H1//IGF2BP3//AFF3//PCBP2//GPR137B//TMEM59//SIRT5//PUM1//SH3PXD2B//LDLR//ADAM19//HUWE1//AKAP7//NID1//FTO//GAPDH//ENC1//PIP5K1C//SESN1//SCMH1//CAPRIN1//RPH3AL//KPNB1//KPNA5//LARP4//ZMYM4//HECW2//KRT17//CRLF3//PPT1//ATP8A1//PKDCC//RNF111//CANX//ITFG2//DDX11//NAT10//MYO19//KLHL21//CSPP1//RNF216//PLEKHM2//TBC1D1//HABP4//RNF4//WDFY1//NALCN//ARHGEF28//FAM120B//ANKRD27//WSB1//USP42//FANK1//ZDHHC17//CLK1//ALKBH5//GCLM//DDX6//ELOVL6//EYA3//MORC2//CLN6//TAF15//BRF1//GOLPH3L//HOOK3//RALGAPA2//IFI30//RAB11FIP5//CYP24A1//NEMF//CHCHD10//VDAC3//PDE4DIP//UBL5//CD99L2// |
| GO:0010632 | regulation of epithelial cell migration | Biological process | 9 | 301 | 262 | 18866 | 2.15305216707667 | 0.0252281190887578 | 0.441485196543038 | 1.59811512759673 | 0.0343511450381679 | BMPR2//THBS1//CORO1C//MCC//PTPRG//DOCK1//PLCG2//NF1//STARD13// |
| GO:0032204 | regulation of telomere maintenance | Biological process | 4 | 80 | 262 | 18866 | 3.60038167938931 | 0.025274316528362 | 0.441485196543038 | 1.5973205798309 | 0.0152671755725191 | TERF2IP//SIRT6//NAT10//SMG1// |
| GO:0071704 | organic substance metabolic process | Biological process | 184 | 12140 | 262 | 18866 | 1.09138423230253 | 0.0252754215469689 | 0.441485196543038 | 1.59730159245325 | 0.702290076335878 | ACTB//KLF11//MTR//CTCF//TCFL5//PRDM5//NEDD4L//IFI16//RBPJ//MDM2//NFATC2//SIRT6//OVOL2//RBL1//BACH2//RPS14//SPI1//NR2C2//NRIP1//KLF7//GTF2IRD1//NCOR2//NAT10//NF1//TAOK3//PSMB5//SRPK2//SMG1//RPL13A//RPL18A//RPL37A//THBS1//HUWE1//WSB1//RNF111//HECW2//UBE3B//CNOT11//CNOT8//TRA2B//DDX17//DYRK1A//PCBP2//CDC40//CCAR1//UBL5//FIP1L1//RCL1//NOL10//TERF2IP//CLN6//TMF1//CORO1C//TFRC//MOB2//DNM2//PPT1//PLCG2//MTO1//GALNT7//PHKG1//SIAE//SLC3A2//GSK3B//UGP2//HK1//COQ2//GAPDH//MDH1//NUP155//SLC23A2//PDE4D//DCTD//POLI//NASP//ATRIP//DDX11//PDS5B//TRRAP//SETD2//EYA3//ALKBH5//FTO//KPNB1//GPBP1L1//BRF1//ZNF483//EFEMP1//GATAD1//ZNF236//ZNF646//ZNF652//LCORL//MEIS1//THAP4//PLAGL1//RFX2//NPAS3//TADA2A//TEX10//PHRF1//TAF15//TRIM24//GTF3C5//HABP4//IGF2BP3//LARP4//RPS27L//LSM14A//RPN1//RPN2//LATS2//BCR//BMPR2//TRIO//NEK9//PHLPP2//PTPRG//SIRT5//GALNT1//PITRM1//PRSS57//DPP8//CFAP44//PAPPA2//TMEM59//MYH9//ADAM19//USP42//GCLM//PITPNB//LDLR//PDE3A//MORC2//ABCD4//ELOVL6//ACP6//PIP5K1C//PI4KA//PCCB//FAF1//SPAG9//RPPH1//MBOAT2//BCKDHA//CYP24A1//AFF3//ENC1//PUM1//SH3PXD2B//SLIT3//PARD3//SCMH1//G2E3//DCAF17//KLHL21//RANGAP1//CAPRIN1//TTLL11//CLK1//PKDCC//ZDHHC17//TIAM2//CRAT//RNF216//RNF4//AHCYL2//SAGE1//OSBPL10//DDR1//ARL6IP1//AMBRA1//MIA3//RAB2A//DDX6//KRT17//CRLF3//FANK1//ICAM1//GNAI2//NEMF//OPHN1//CHCHD10//LPXN// |
| GO:0001675 | acrosome assembly | Biological process | 2 | 18 | 262 | 18866 | 8.0008481764207 | 0.0253921220847816 | 0.441485196543038 | 1.5953010025056 | 0.00763358778625954 | RFX2//TMF1// |
| GO:0010766 | negative regulation of sodium ion transport | Biological process | 2 | 18 | 262 | 18866 | 8.0008481764207 | 0.0253921220847816 | 0.441485196543038 | 1.5953010025056 | 0.00763358778625954 | NEDD4L//HECW2// |
| GO:0043011 | myeloid dendritic cell differentiation | Biological process | 2 | 18 | 262 | 18866 | 8.0008481764207 | 0.0253921220847816 | 0.441485196543038 | 1.5953010025056 | 0.00763358778625954 | RBPJ//SPI1// |
| GO:0044872 | lipoprotein localization | Biological process | 2 | 18 | 262 | 18866 | 8.0008481764207 | 0.0253921220847816 | 0.441485196543038 | 1.5953010025056 | 0.00763358778625954 | ZDHHC17//MIA3// |
| GO:0051895 | negative regulation of focal adhesion assembly | Biological process | 2 | 18 | 262 | 18866 | 8.0008481764207 | 0.0253921220847816 | 0.441485196543038 | 1.5953010025056 | 0.00763358778625954 | CORO1C//THBS1// |
| GO:0061323 | cell proliferation involved in heart morphogenesis | Biological process | 2 | 18 | 262 | 18866 | 8.0008481764207 | 0.0253921220847816 | 0.441485196543038 | 1.5953010025056 | 0.00763358778625954 | BMPR2//RBPJ// |
| GO:0150118 | negative regulation of cell-substrate junction organization | Biological process | 2 | 18 | 262 | 18866 | 8.0008481764207 | 0.0253921220847816 | 0.441485196543038 | 1.5953010025056 | 0.00763358778625954 | CORO1C//THBS1// |
| GO:1990000 | amyloid fibril formation | Biological process | 2 | 18 | 262 | 18866 | 8.0008481764207 | 0.0253921220847816 | 0.441485196543038 | 1.5953010025056 | 0.00763358778625954 | LDLR//MDM2// |
| GO:0006725 | cellular aromatic compound metabolic process | Biological process | 105 | 6444 | 262 | 18866 | 1.17330874095555 | 0.0254666306037103 | 0.44169274061816 | 1.59402851114217 | 0.400763358778626 | KLF11//CTCF//TCFL5//PRDM5//NEDD4L//IFI16//RBPJ//MDM2//NFATC2//SIRT6//OVOL2//RBL1//BACH2//RPS14//SPI1//NR2C2//NRIP1//KLF7//GTF2IRD1//NCOR2//NAT10//SMG1//RPL13A//RPL18A//RPL37A//SRPK2//CNOT11//CNOT8//TRA2B//DDX17//DYRK1A//PCBP2//CDC40//CCAR1//UBL5//FIP1L1//RCL1//NOL10//TERF2IP//MTO1//UGP2//GAPDH//HK1//NUP155//SLC23A2//PDE4D//DCTD//POLI//DDX11//PDS5B//TAOK3//TRRAP//ATRIP//HUWE1//SETD2//EYA3//ALKBH5//FTO//KPNB1//GPBP1L1//BRF1//ZNF483//DNM2//EFEMP1//GATAD1//ZNF236//ZNF646//ZNF652//LCORL//MEIS1//THAP4//PLAGL1//RFX2//NPAS3//TADA2A//TEX10//PHRF1//TAF15//TRIM24//GTF3C5//HABP4//FAF1//RPPH1//MTR//ABCD4//SAGE1//PUM1//ELOVL6//CLK1//IGF2BP3//PSMB5//DDX6//TFRC//SCMH1//CRLF3//RNF111//RNF4//FANK1//BMPR2//TMF1//PPT1//ICAM1//MORC2//CHCHD10//LPXN// |
| GO:0005978 | glycogen biosynthetic process | Biological process | 3 | 46 | 262 | 18866 | 4.69615001659476 | 0.0259217323993535 | 0.447387552584441 | 1.58633597709396 | 0.0114503816793893 | GSK3B//PHKG1//UGP2// |
| GO:0009250 | glucan biosynthetic process | Biological process | 3 | 46 | 262 | 18866 | 4.69615001659476 | 0.0259217323993535 | 0.447387552584441 | 1.58633597709396 | 0.0114503816793893 | PHKG1//UGP2//GSK3B// |
| GO:0000184 | nuclear-transcribed mRNA catabolic process, nonsense-mediated decay | Biological process | 5 | 120 | 262 | 18866 | 3.00031806615776 | 0.0261038468327271 | 0.449431840956636 | 1.58329548748468 | 0.0190839694656489 | SMG1//RPL13A//RPL18A//RPL37A//RPS14// |
| GO:0016070 | RNA metabolic process | Biological process | 87 | 5213 | 262 | 18866 | 1.20173875352722 | 0.0263867754301951 | 0.452674345763784 | 1.57861367906518 | 0.33206106870229 | KLF11//CTCF//TCFL5//PRDM5//NEDD4L//IFI16//RBPJ//MDM2//NFATC2//SIRT6//OVOL2//RBL1//BACH2//RPS14//SPI1//NR2C2//NRIP1//KLF7//GTF2IRD1//NCOR2//NAT10//SMG1//RPL13A//RPL18A//RPL37A//SRPK2//CNOT11//CNOT8//TRA2B//DDX17//DYRK1A//PCBP2//CDC40//CCAR1//UBL5//FIP1L1//RCL1//NOL10//MTO1//GPBP1L1//BRF1//ZNF483//DNM2//EFEMP1//SETD2//TERF2IP//GATAD1//ZNF236//TRRAP//ZNF646//ZNF652//LCORL//MEIS1//THAP4//PLAGL1//RFX2//NPAS3//TADA2A//TEX10//PHRF1//TAF15//TRIM24//GTF3C5//HABP4//ALKBH5//FAF1//RPPH1//SAGE1//PUM1//FTO//CLK1//IGF2BP3//PSMB5//DDX6//SCMH1//CRLF3//RNF111//RNF4//FANK1//BMPR2//TMF1//ICAM1//TFRC//MORC2//DDX11//CHCHD10//LPXN// |
| GO:0061013 | regulation of mRNA catabolic process | Biological process | 7 | 208 | 262 | 18866 | 2.42333382266588 | 0.0264736343821275 | 0.452674345763784 | 1.57718643332068 | 0.0267175572519084 | IGF2BP3//ALKBH5//PSMB5//PUM1//TAF15//CNOT8//FTO// |
| GO:0009267 | cellular response to starvation | Biological process | 6 | 163 | 262 | 18866 | 2.65058773942772 | 0.0264845593994111 | 0.452674345763784 | 1.577007247648 | 0.0229007633587786 | GABARAP//SESN1//ITFG2//IFI16//AMBRA1//BMPR2// |
| GO:0018205 | peptidyl-lysine modification | Biological process | 11 | 405 | 262 | 18866 | 1.9557628875695 | 0.0265750115201025 | 0.453123203672472 | 1.57552653854066 | 0.0419847328244275 | TRRAP//MDM2//RANGAP1//NUP155//SETD2//RNF4//CTCF//TADA2A//SIRT5//SPI1//PRDM5// |
| GO:0050767 | regulation of neurogenesis | Biological process | 19 | 845 | 262 | 18866 | 1.6191065540449 | 0.0270477652650034 | 0.46007271085701 | 1.5678686112661 | 0.0725190839694656 | NF1//GSK3B//MOB2//ENC1//MDM2//PTPRG//DNM2//DDX6//MEIS1//SPAG9//WDR1//NEDD4L//HECW2//BMPR2//HOOK3//TIAM2//CAPRIN1//ANKRD27//LDLR// |
| GO:0016925 | protein sumoylation | Biological process | 4 | 82 | 262 | 18866 | 3.51256749208713 | 0.0273638144722139 | 0.460610521480818 | 1.56282336277193 | 0.0152671755725191 | RNF4//MDM2//RANGAP1//NUP155// |
| GO:1903313 | positive regulation of mRNA metabolic process | Biological process | 4 | 82 | 262 | 18866 | 3.51256749208713 | 0.0273638144722139 | 0.460610521480818 | 1.56282336277193 | 0.0152671755725191 | TRA2B//CNOT8//FTO//PUM1// |
| GO:0010569 | regulation of double-strand break repair via homologous recombination | Biological process | 3 | 47 | 262 | 18866 | 4.59623193113529 | 0.0274124187907829 | 0.460610521480818 | 1.56205264190875 | 0.0114503816793893 | SETD2//SIRT6//TERF2IP// |
| GO:0002573 | myeloid leukocyte differentiation | Biological process | 7 | 210 | 262 | 18866 | 2.40025445292621 | 0.0276949152199421 | 0.460610521480818 | 1.55759995998354 | 0.0267175572519084 | IFI16//MYH9//SPI1//TFRC//RBPJ//NF1//GPR137B// |
| GO:0003159 | morphogenesis of an endothelium | Biological process | 2 | 19 | 262 | 18866 | 7.5797509039775 | 0.0281234076722245 | 0.460610521480818 | 1.55093205763589 | 0.00763358778625954 | RBPJ//STARD13// |
| GO:0007213 | G protein-coupled acetylcholine receptor signaling pathway | Biological process | 2 | 19 | 262 | 18866 | 7.5797509039775 | 0.0281234076722245 | 0.460610521480818 | 1.55093205763589 | 0.00763358778625954 | GNAI2//GNB1// |
| GO:0019054 | modulation by virus of host cellular process | Biological process | 2 | 19 | 262 | 18866 | 7.5797509039775 | 0.0281234076722245 | 0.460610521480818 | 1.55093205763589 | 0.00763358778625954 | KPNB1//KPNA5// |
| GO:0030011 | maintenance of cell polarity | Biological process | 2 | 19 | 262 | 18866 | 7.5797509039775 | 0.0281234076722245 | 0.460610521480818 | 1.55093205763589 | 0.00763358778625954 | WDR1//GSK3B// |
| GO:0031445 | regulation of heterochromatin assembly | Biological process | 2 | 19 | 262 | 18866 | 7.5797509039775 | 0.0281234076722245 | 0.460610521480818 | 1.55093205763589 | 0.00763358778625954 | MORC2//SIRT6// |
| GO:0032026 | response to magnesium ion | Biological process | 2 | 19 | 262 | 18866 | 7.5797509039775 | 0.0281234076722245 | 0.460610521480818 | 1.55093205763589 | 0.00763358778625954 | MDM2//THBS1// |
| GO:0035988 | chondrocyte proliferation | Biological process | 2 | 19 | 262 | 18866 | 7.5797509039775 | 0.0281234076722245 | 0.460610521480818 | 1.55093205763589 | 0.00763358778625954 | BMPR2//SIRT6// |
| GO:0061154 | endothelial tube morphogenesis | Biological process | 2 | 19 | 262 | 18866 | 7.5797509039775 | 0.0281234076722245 | 0.460610521480818 | 1.55093205763589 | 0.00763358778625954 | RBPJ//STARD13// |
| GO:0070989 | oxidative demethylation | Biological process | 2 | 19 | 262 | 18866 | 7.5797509039775 | 0.0281234076722245 | 0.460610521480818 | 1.55093205763589 | 0.00763358778625954 | FTO//ALKBH5// |
| GO:0097320 | plasma membrane tubulation | Biological process | 2 | 19 | 262 | 18866 | 7.5797509039775 | 0.0281234076722245 | 0.460610521480818 | 1.55093205763589 | 0.00763358778625954 | DNM2//PLEKHM2// |
| GO:1902170 | cellular response to reactive nitrogen species | Biological process | 2 | 19 | 262 | 18866 | 7.5797509039775 | 0.0281234076722245 | 0.460610521480818 | 1.55093205763589 | 0.00763358778625954 | DNM2//MTR// |
| GO:2000647 | negative regulation of stem cell proliferation | Biological process | 2 | 19 | 262 | 18866 | 7.5797509039775 | 0.0281234076722245 | 0.460610521480818 | 1.55093205763589 | 0.00763358778625954 | NF1//OVOL2// |
| GO:0051093 | negative regulation of developmental process | Biological process | 23 | 1082 | 262 | 18866 | 1.53066134243908 | 0.0282359814814718 | 0.461383780735439 | 1.54919711160768 | 0.0877862595419847 | BMPR2//NF1//HUWE1//TFRC//OVOL2//MDM2//PTPRG//THBS1//EFEMP1//RBPJ//TRIO//MEIS1//DDX6//GPR137B//HOOK3//BCR//LDLR//STARD13//CORO1C//KLF7//GSK3B//NFATC2//RBL1// |
| GO:0090304 | nucleic acid metabolic process | Biological process | 94 | 5709 | 262 | 18866 | 1.18562227312172 | 0.0284234825146416 | 0.463374972450012 | 1.5463227123257 | 0.358778625954198 | KLF11//CTCF//TCFL5//PRDM5//NEDD4L//IFI16//RBPJ//MDM2//NFATC2//SIRT6//OVOL2//RBL1//BACH2//RPS14//SPI1//NR2C2//NRIP1//KLF7//GTF2IRD1//NCOR2//NAT10//SMG1//RPL13A//RPL18A//RPL37A//SRPK2//CNOT11//CNOT8//TRA2B//DDX17//DYRK1A//PCBP2//CDC40//CCAR1//UBL5//FIP1L1//RCL1//NOL10//TERF2IP//MTO1//POLI//DDX11//PDS5B//TAOK3//TRRAP//ATRIP//HUWE1//SETD2//EYA3//ALKBH5//FTO//KPNB1//GPBP1L1//BRF1//ZNF483//DNM2//EFEMP1//GATAD1//ZNF236//ZNF646//ZNF652//LCORL//MEIS1//THAP4//PLAGL1//RFX2//NPAS3//TADA2A//TEX10//PHRF1//TAF15//TRIM24//GTF3C5//HABP4//FAF1//RPPH1//SAGE1//PUM1//CLK1//IGF2BP3//PSMB5//DDX6//TFRC//SCMH1//CRLF3//RNF111//RNF4//FANK1//BMPR2//TMF1//ICAM1//MORC2//CHCHD10//LPXN// |
| GO:0007093 | mitotic cell cycle checkpoint | Biological process | 6 | 166 | 262 | 18866 | 2.60268555136577 | 0.0286164080617779 | 0.464711912369364 | 1.54338487990008 | 0.0229007633587786 | MDM2//PLAGL1//CNOT11//CNOT8//TAOK3//RPS27L// |
| GO:1904375 | regulation of protein localization to cell periphery | Biological process | 5 | 123 | 262 | 18866 | 2.92713957673928 | 0.0286371556708703 | 0.464711912369364 | 1.54307011966689 | 0.0190839694656489 | PKDCC//ACTB//TMEM59//STAC//GNAI1// |
| GO:0035088 | establishment or maintenance of apical/basal cell polarity | Biological process | 3 | 48 | 262 | 18866 | 4.50047709923664 | 0.028947930245485 | 0.465942376557487 | 1.53838248254445 | 0.0114503816793893 | PARD3//OPHN1//WDR1// |
| GO:0061245 | establishment or maintenance of bipolar cell polarity | Biological process | 3 | 48 | 262 | 18866 | 4.50047709923664 | 0.028947930245485 | 0.465942376557487 | 1.53838248254445 | 0.0114503816793893 | PARD3//OPHN1//WDR1// |
| GO:0031589 | cell-substrate adhesion | Biological process | 10 | 359 | 262 | 18866 | 2.00578366539795 | 0.028961771088681 | 0.465942376557487 | 1.5381748834099 | 0.0381679389312977 | NF1//DDR1//THBS1//GSK3B//NID1//LPXN//BCR//CORO1C//DNM2//DOCK1// |
| GO:0009892 | negative regulation of metabolic process | Biological process | 66 | 3818 | 262 | 18866 | 1.24476265500102 | 0.0289770085435241 | 0.465942376557487 | 1.53794645116638 | 0.251908396946565 | CTCF//TCFL5//PRDM5//NEDD4L//IFI16//RBPJ//MDM2//NFATC2//SIRT6//OVOL2//RBL1//BACH2//RPS14//SPI1//NR2C2//NRIP1//KLF11//KLF7//GTF2IRD1//NCOR2//SMG1//RPL13A//RPL18A//RPL37A//CNOT11//CNOT8//CORO1C//TERF2IP//NF1//TAOK3//LDLR//SLIT3//TMF1//THBS1//PARD3//GAPDH//TMEM59//PUM1//SCMH1//IGF2BP3//CAPRIN1//ENC1//DDX17//NAT10//PCBP2//RNF216//GSK3B//PDE4D//RNF4//ARL6IP1//ALKBH5//PSMB5//DDX6//LATS2//MORC2//TRIM24//DYRK1A//TAF15//BCR//NUP155//FTO//OPHN1//CCAR1//GNAI2//BMPR2//SIRT5// |
| GO:0034250 | positive regulation of cellular amide metabolic process | Biological process | 6 | 167 | 262 | 18866 | 2.58710060794442 | 0.0293514194592861 | 0.470890159007047 | 1.53237089106275 | 0.0229007633587786 | LARP4//KRT17//RPS27L//NAT10//THBS1//HABP4// |
| GO:0010633 | negative regulation of epithelial cell migration | Biological process | 5 | 124 | 262 | 18866 | 2.90353361241074 | 0.0295149555614762 | 0.472440070994241 | 1.52995786632794 | 0.0190839694656489 | THBS1//STARD13//CORO1C//MCC//PTPRG// |
| GO:0018105 | peptidyl-serine phosphorylation | Biological process | 9 | 310 | 262 | 18866 | 2.09054420093573 | 0.0297044764600837 | 0.474219162905253 | 1.52717809763843 | 0.0343511450381679 | PDE4D//TERF2IP//TFRC//DYRK1A//CLK1//SMG1//LATS2//GSK3B//SRPK2// |
| GO:0045055 | regulated exocytosis | Biological process | 18 | 797 | 262 | 18866 | 1.62627026923482 | 0.0297912167980721 | 0.474219162905253 | 1.52591175801704 | 0.0687022900763359 | HABP4//THBS1//WDR1//PIP5K1C//RPH3AL//HUWE1//ATP8A1//SLC15A4//DYNC1H1//DOCK2//NFASC//GDI2//KPNB1//BCR//GNAI2//GSK3B//RAB11FIP5//MYH9// |
| GO:0015931 | nucleobase-containing compound transport | Biological process | 8 | 261 | 262 | 18866 | 2.2071305314264 | 0.029827639655749 | 0.474219162905253 | 1.5253811122109 | 0.0305343511450382 | CDC40//NUP155//SMG1//ALKBH5//FIP1L1//SETD2//IGF2BP3//RANBP17// |
| GO:0051716 | cellular response to stimulus | Biological process | 123 | 7748 | 262 | 18866 | 1.14312583005907 | 0.0302867638471575 | 0.478884917975387 | 1.51874712872741 | 0.469465648854962 | ATRIP//NF1//TAOK3//PSMB5//SRPK2//THBS1//GNAI2//DNM2//PLCG2//POLI//DDX11//PDS5B//SMG1//TRRAP//HUWE1//SIRT6//SETD2//EYA3//ALKBH5//FTO//MORC2//NFATC2//MDM2//PLAGL1//CNOT11//CNOT8//RPS27L//GSK3B//GABARAP//DOCK1//PHLPP2//FLNB//CORO1C//GDI2//GNB1//MCC//OPHN1//PDE3A//PDE4D//PI4KA//RANGAP1//BCR//LMBR1//STAC//STARD13//LPXN//PTPRG//DDR1//EFEMP1//BMPR2//PARD3//TRIO//TIAM2//GNAI1//RBPJ//EVC//MYH9//SPAG9//DOCK2//MYO9B//AP3S1//ATP6V0B//AMBRA1//LATS2//TERF2IP//AKAP7//PIP5K1C//STRN//ICAM1//RNF111//DDX17//TMF1//CANX//MTR//KPNB1//ITFG2//SLIT3//WDFY1//SESN1//ARHGEF28//FAM120B//IFI16//WSB1//TFRC//ANKRD27//GCLM//RAB11FIP5//ACTB//LSM14A//ZDHHC17//DYRK1A//RPH3AL//CRLF3//RALGAPA2//IFI30//OVOL2//NCOR2//KLF7//SPI1//CYP24A1//TRIM24//HABP4//TRA2B//ZNF236//RPL13A//GAPDH//TUBA1B//NRIP1//LDLR//RCSD1//HBG2//MIA3//NUP155//PUM1//CHCHD10//KLF11//TAF15//FAF1//GPR137B//PRDM5//ABCD4//RFX2//RBL1// |
| GO:0001952 | regulation of cell-matrix adhesion | Biological process | 5 | 125 | 262 | 18866 | 2.88030534351145 | 0.0304095773740067 | 0.478884917975387 | 1.51698961554848 | 0.0190839694656489 | NF1//THBS1//GSK3B//CORO1C//DDR1// |
| GO:0032479 | regulation of type I interferon production | Biological process | 5 | 125 | 262 | 18866 | 2.88030534351145 | 0.0304095773740067 | 0.478884917975387 | 1.51698961554848 | 0.0190839694656489 | PCBP2//RNF216//IFI16//PLCG2//SETD2// |
| GO:0034605 | cellular response to heat | Biological process | 5 | 125 | 262 | 18866 | 2.88030534351145 | 0.0304095773740067 | 0.478884917975387 | 1.51698961554848 | 0.0190839694656489 | GSK3B//NUP155//NF1//STAC//THBS1// |
| GO:0045595 | regulation of cell differentiation | Biological process | 37 | 1947 | 262 | 18866 | 1.36840392539707 | 0.0304921173483936 | 0.478884917975387 | 1.51581241752388 | 0.141221374045802 | NF1//OVOL2//GSK3B//MOB2//ENC1//MDM2//PTPRG//DNM2//EFEMP1//PKDCC//NFATC2//RBPJ//TRIO//SH3PXD2B//KLF7//MEIS1//SPI1//THBS1//DDX6//SPAG9//BMPR2//GPR137B//WDR1//NEDD4L//HECW2//HOOK3//TIAM2//CAPRIN1//ANKRD27//PDE3A//LDLR//FTO//CORO1C//DOCK1//PSMB5//DDX17//TCFL5// |
| GO:0042149 | cellular response to glucose starvation | Biological process | 3 | 49 | 262 | 18866 | 4.40863062782365 | 0.030528150317173 | 0.478884917975387 | 1.51529950855363 | 0.0114503816793893 | SESN1//IFI16//ITFG2// |
| GO:0006767 | water-soluble vitamin metabolic process | Biological process | 4 | 85 | 262 | 18866 | 3.38859452177818 | 0.0306863244588946 | 0.479236204325967 | 1.51305512730017 | 0.0152671755725191 | PCCB//MTR//ABCD4//SLC23A2// |
| GO:2000779 | regulation of double-strand break repair | Biological process | 4 | 85 | 262 | 18866 | 3.38859452177818 | 0.0306863244588946 | 0.479236204325967 | 1.51305512730017 | 0.0152671755725191 | SETD2//SIRT6//TERF2IP//DDX11// |
| GO:0006607 | NLS-bearing protein import into nucleus | Biological process | 2 | 20 | 262 | 18866 | 7.20076335877863 | 0.0309666591742681 | 0.479372033138506 | 1.50910564580377 | 0.00763358778625954 | KPNB1//KPNA5// |
| GO:0071731 | response to nitric oxide | Biological process | 2 | 20 | 262 | 18866 | 7.20076335877863 | 0.0309666591742681 | 0.479372033138506 | 1.50910564580377 | 0.00763358778625954 | DNM2//MTR// |
| GO:0098884 | postsynaptic neurotransmitter receptor internalization | Biological process | 2 | 20 | 262 | 18866 | 7.20076335877863 | 0.0309666591742681 | 0.479372033138506 | 1.50910564580377 | 0.00763358778625954 | OPHN1//DNM2// |
| GO:0140239 | postsynaptic endocytosis | Biological process | 2 | 20 | 262 | 18866 | 7.20076335877863 | 0.0309666591742681 | 0.479372033138506 | 1.50910564580377 | 0.00763358778625954 | DNM2//OPHN1// |
| GO:0048731 | system development | Biological process | 84 | 5045 | 262 | 18866 | 1.19893780403846 | 0.03112081208175 | 0.480704184868869 | 1.50694907884105 | 0.320610687022901 | EVC//SH3PXD2B//PKDCC//SETD2//RBPJ//MEIS1//MYH9//OVOL2//SRPK2//ICAM1//NRIP1//MDM2//BMPR2//NF1//CORO1C//SDCCAG8//DDX17//GSK3B//ADAM19//THBS1//MIA3//PDS5B//ACP6//ITFG2//PLCG2//SPI1//SLIT3//SIRT6//DNM2//FLNB//IGF2BP3//DYRK1A//MTR//OPHN1//PPT1//NR2C2//ENC1//PARD3//ACTB//KLF7//NFASC//TRIO//PTPRG//BCR//UGP2//DDR1//NCOR2//NASP//MOB2//TRAPPC4//STRN//COX6B1//PHLPP2//TRA2B//AMBRA1//HOOK3//IFI16//RPS14//WDR1//TFRC//KRT17//FRYL//EFEMP1//TTC39C//NID1//TMF1//GTF3C5//DOCK2//NFATC2//DDX6//SPAG9//GPR137B//TAOK3//ANKRD27//NEDD4L//HECW2//LDLR//TIAM2//CAPRIN1//GNB1//PSMB5//PAPPA2//FTO//STARD13// |
| GO:0018210 | peptidyl-threonine modification | Biological process | 5 | 126 | 262 | 18866 | 2.85744577729311 | 0.031321099730237 | 0.482638934448976 | 1.5041629976234 | 0.0190839694656489 | PARD3//CLK1//DYRK1A//GSK3B//GALNT1// |
| GO:0044093 | positive regulation of molecular function | Biological process | 35 | 1826 | 262 | 18866 | 1.38021203481518 | 0.0313828121422411 | 0.482638934448976 | 1.50330814286708 | 0.133587786259542 | THBS1//RPS27L//SPAG9//DDX11//GSK3B//TAOK3//PLCG2//DDR1//GNAI2//GCLM//PITRM1//SH3PXD2B//AGAP1//DOCK1//DOCK2//GDI2//ICAM1//MYO9B//NF1//OPHN1//ANKRD27//STARD13//AMBRA1//FANK1//TERF2IP//TFRC//TBC1D1//CORO1C//TIAM2//RALGAPA2//RANGAP1//BCR//IFI16//STAC//DNM2// |
| GO:0032870 | cellular response to hormone stimulus | Biological process | 15 | 633 | 262 | 18866 | 1.70634202814659 | 0.0315632509970043 | 0.484358671277942 | 1.50081827107272 | 0.0572519083969466 | AP3S1//GSK3B//ATP6V0B//LATS2//DDX17//TMF1//GNAI2//NCOR2//GCLM//MDM2//GNB1//TRIM24//ICAM1//RANGAP1//SLIT3// |
| GO:0051052 | regulation of DNA metabolic process | Biological process | 10 | 365 | 262 | 18866 | 1.97281187911743 | 0.0319145682913988 | 0.486431430610516 | 1.49601102589911 | 0.0381679389312977 | SETD2//SIRT6//TERF2IP//DDX11//SMG1//NAT10//SPI1//EYA3//TFRC//BMPR2// |
| GO:0019674 | NAD metabolic process | Biological process | 3 | 50 | 262 | 18866 | 4.32045801526718 | 0.0321529378626462 | 0.486431430610516 | 1.49277933878195 | 0.0114503816793893 | GAPDH//HK1//MDH1// |
| GO:0046850 | regulation of bone remodeling | Biological process | 3 | 50 | 262 | 18866 | 4.32045801526718 | 0.0321529378626462 | 0.486431430610516 | 1.49277933878195 | 0.0114503816793893 | NF1//GPR137B//TFRC// |
| GO:0051653 | spindle localization | Biological process | 3 | 50 | 262 | 18866 | 4.32045801526718 | 0.0321529378626462 | 0.486431430610516 | 1.49277933878195 | 0.0114503816793893 | KPNB1//DYNC1H1//MYH9// |
| GO:0061014 | positive regulation of mRNA catabolic process | Biological process | 3 | 50 | 262 | 18866 | 4.32045801526718 | 0.0321529378626462 | 0.486431430610516 | 1.49277933878195 | 0.0114503816793893 | FTO//PUM1//CNOT8// |
| GO:0030522 | intracellular receptor signaling pathway | Biological process | 8 | 265 | 262 | 18866 | 2.17381535359355 | 0.0322167760684204 | 0.486431430610516 | 1.49191792157504 | 0.0305343511450382 | DDX17//TMF1//FAM120B//LSM14A//NCOR2//CYP24A1//TRIM24//PUM1// |
| GO:0019079 | viral genome replication | Biological process | 5 | 127 | 262 | 18866 | 2.83494620424355 | 0.032249597609537 | 0.486431430610516 | 1.4914756998519 | 0.0190839694656489 | PCBP2//PI4KA//SRPK2//IFI16//MORC2// |
| GO:0032606 | type I interferon production | Biological process | 5 | 127 | 262 | 18866 | 2.83494620424355 | 0.032249597609537 | 0.486431430610516 | 1.4914756998519 | 0.0190839694656489 | PCBP2//RNF216//IFI16//PLCG2//SETD2// |
| GO:0051170 | import into nucleus | Biological process | 6 | 171 | 262 | 18866 | 2.52658363465917 | 0.032415239442521 | 0.487887367216963 | 1.4892507660345 | 0.0229007633587786 | KPNB1//RANBP17//NUP155//KPNA5//NF1//BACH2// |
| GO:0010467 | gene expression | Biological process | 111 | 6921 | 262 | 18866 | 1.15486885251326 | 0.0325133416905579 | 0.488322721263081 | 1.48793839183311 | 0.423664122137405 | KLF11//CTCF//TCFL5//PRDM5//NEDD4L//IFI16//RBPJ//MDM2//NFATC2//SIRT6//OVOL2//RBL1//BACH2//RPS14//SPI1//NR2C2//NRIP1//KLF7//GTF2IRD1//NCOR2//NAT10//SMG1//RPL13A//RPL18A//RPL37A//SRPK2//CNOT11//CNOT8//TRA2B//DDX17//DYRK1A//PCBP2//CDC40//CCAR1//UBL5//FIP1L1//RCL1//NOL10//TMF1//MTO1//GPBP1L1//BRF1//ZNF483//DNM2//EFEMP1//SETD2//TERF2IP//GATAD1//ZNF236//TRRAP//ZNF646//ZNF652//LCORL//MEIS1//THAP4//PLAGL1//RFX2//NPAS3//TADA2A//TEX10//PHRF1//TAF15//TRIM24//GTF3C5//HABP4//ALKBH5//NUP155//IGF2BP3//LARP4//RPS27L//LSM14A//FAF1//RPPH1//AFF3//NF1//PUM1//SH3PXD2B//GSK3B//LDLR//TFRC//ADAM19//SLIT3//THBS1//TMEM59//SCMH1//PITRM1//GAPDH//CAPRIN1//ENC1//RNF216//PLCG2//PDE4D//HUWE1//SAGE1//CLK1//PSMB5//DDX6//KRT17//MORC2//ACTB//CRLF3//RNF111//RNF4//FANK1//BMPR2//ICAM1//FTO//NEMF//DDX11//CHCHD10//MYH9// |
| GO:0000122 | negative regulation of transcription by RNA polymerase II | Biological process | 20 | 922 | 262 | 18866 | 1.561987713401 | 0.0328193850827719 | 0.491640729921451 | 1.48386956032317 | 0.0763358778625954 | CTCF//TCFL5//PRDM5//NEDD4L//IFI16//RBPJ//MDM2//NFATC2//SIRT6//OVOL2//RBL1//BACH2//RPS14//SPI1//NR2C2//NRIP1//KLF11//KLF7//GTF2IRD1//NCOR2// |
| GO:0006338 | chromatin remodeling | Biological process | 7 | 218 | 262 | 18866 | 2.31217172070873 | 0.0329619835489587 | 0.491640729921451 | 1.4819866617065 | 0.0267175572519084 | NASP//ACTB//MORC2//SIRT6//SCMH1//GATAD1//TADA2A// |
| GO:0006511 | ubiquitin-dependent protein catabolic process | Biological process | 15 | 637 | 262 | 18866 | 1.69562716454756 | 0.033087224347117 | 0.491640729921451 | 1.48033966382508 | 0.0572519083969466 | RNF111//PSMB5//GSK3B//MDM2//HUWE1//FAF1//NEDD4L//PCBP2//RNF216//HECW2//RNF4//SIRT6//NEMF//USP42//UBE3B// |
| GO:0000910 | cytokinesis | Biological process | 6 | 172 | 262 | 18866 | 2.51189419492278 | 0.0332124549947633 | 0.491640729921451 | 1.47869902108115 | 0.0229007633587786 | TTC19//SETD2//MYO19//KLHL21//CSPP1//MYH9// |
| GO:1990138 | neuron projection extension | Biological process | 6 | 172 | 262 | 18866 | 2.51189419492278 | 0.0332124549947633 | 0.491640729921451 | 1.47869902108115 | 0.0229007633587786 | DNM2//GSK3B//BMPR2//SLIT3//NEDD4L//DDR1// |
| GO:0000075 | cell cycle checkpoint | Biological process | 7 | 219 | 262 | 18866 | 2.30161385897034 | 0.0336642056173941 | 0.491640729921451 | 1.47283162922968 | 0.0267175572519084 | ATRIP//MDM2//PLAGL1//CNOT11//CNOT8//TAOK3//RPS27L// |
| GO:0010033 | response to organic substance | Biological process | 59 | 3385 | 262 | 18866 | 1.25508135352419 | 0.0338932702445284 | 0.491640729921451 | 1.46988652556706 | 0.225190839694656 | ICAM1//THBS1//PARD3//LPXN//GNB1//GSK3B//GNAI2//AP3S1//ATP6V0B//COL6A2//LATS2//NCOR2//BMPR2//RNF111//DNM2//RBPJ//DDX17//TMF1//CANX//PLCG2//SLIT3//PSMB5//CYP24A1//NASP//SETD2//AFF3//IFI16//GCLM//RAB11FIP5//DDX11//MDM2//SLC3A2//GNAI1//IFI30//LSM14A//KLF7//SPI1//TRIM24//PDE4D//BCR//SESN1//PDE3A//TRA2B//ZNF236//FLNB//RPL13A//GAPDH//TUBA1B//NRIP1//LDLR//OVOL2//ACTB//MIA3//KLF11//RANGAP1//PRDM5//ABCD4//RFX2//TFRC// |
| GO:0000028 | ribosomal small subunit assembly | Biological process | 2 | 21 | 262 | 18866 | 6.85786986550345 | 0.0339182654018624 | 0.491640729921451 | 1.46956636589828 | 0.00763358778625954 | RPS27L//RPS14// |
| GO:0002693 | positive regulation of cellular extravasation | Biological process | 2 | 21 | 262 | 18866 | 6.85786986550345 | 0.0339182654018624 | 0.491640729921451 | 1.46956636589828 | 0.00763358778625954 | CD99L2//ICAM1// |
| GO:0003177 | pulmonary valve development | Biological process | 2 | 21 | 262 | 18866 | 6.85786986550345 | 0.0339182654018624 | 0.491640729921451 | 1.46956636589828 | 0.00763358778625954 | RBPJ//BMPR2// |
| GO:0010847 | regulation of chromatin assembly | Biological process | 2 | 21 | 262 | 18866 | 6.85786986550345 | 0.0339182654018624 | 0.491640729921451 | 1.46956636589828 | 0.00763358778625954 | MORC2//SIRT6// |
| GO:0030220 | platelet formation | Biological process | 2 | 21 | 262 | 18866 | 6.85786986550345 | 0.0339182654018624 | 0.491640729921451 | 1.46956636589828 | 0.00763358778625954 | MYH9//WDR1// |
| GO:0046823 | negative regulation of nucleocytoplasmic transport | Biological process | 2 | 21 | 262 | 18866 | 6.85786986550345 | 0.0339182654018624 | 0.491640729921451 | 1.46956636589828 | 0.00763358778625954 | NF1//RANGAP1// |
| GO:0046827 | positive regulation of protein export from nucleus | Biological process | 2 | 21 | 262 | 18866 | 6.85786986550345 | 0.0339182654018624 | 0.491640729921451 | 1.46956636589828 | 0.00763358778625954 | GSK3B//MDM2// |
| GO:0060216 | definitive hemopoiesis | Biological process | 2 | 21 | 262 | 18866 | 6.85786986550345 | 0.0339182654018624 | 0.491640729921451 | 1.46956636589828 | 0.00763358778625954 | MEIS1//BCR// |
| GO:0060716 | labyrinthine layer blood vessel development | Biological process | 2 | 21 | 262 | 18866 | 6.85786986550345 | 0.0339182654018624 | 0.491640729921451 | 1.46956636589828 | 0.00763358778625954 | RBPJ//OVOL2// |
| GO:0098780 | response to mitochondrial depolarisation | Biological process | 2 | 21 | 262 | 18866 | 6.85786986550345 | 0.0339182654018624 | 0.491640729921451 | 1.46956636589828 | 0.00763358778625954 | HUWE1//AMBRA1// |
| GO:0000041 | transition metal ion transport | Biological process | 5 | 129 | 262 | 18866 | 2.7909935499142 | 0.0341578016137872 | 0.493240641444129 | 1.4665100881506 | 0.0190839694656489 | TFRC//SLC30A7//DNM2//ATP6V0B//ATP13A1// |
| GO:0120035 | regulation of plasma membrane bounded cell projection organization | Biological process | 16 | 696 | 262 | 18866 | 1.6553478985698 | 0.0341683912262614 | 0.493240641444129 | 1.46637546890406 | 0.0610687022900763 | DNM2//GSK3B//MOB2//ENC1//MDM2//PTPRG//NEDD4L//HECW2//BMPR2//TIAM2//CAPRIN1//ANKRD27//CORO1C//ICAM1//SDCCAG8//VDAC3// |
| GO:0061564 | axon development | Biological process | 13 | 530 | 262 | 18866 | 1.76622497479476 | 0.0345249022515101 | 0.497369969374306 | 1.46186754234209 | 0.049618320610687 | PARD3//ACTB//KLF7//NFASC//OPHN1//SLIT3//TRIO//DNM2//GSK3B//MTR//BMPR2//TIAM2//DDR1// |
| GO:0003205 | cardiac chamber development | Biological process | 6 | 174 | 262 | 18866 | 2.4830218478547 | 0.0348448168887179 | 0.500956338935763 | 1.45786181337764 | 0.0229007633587786 | BMPR2//RBPJ//DNM2//MDM2//OVOL2//SLIT3// |
| GO:0060255 | regulation of macromolecule metabolic process | Biological process | 112 | 7012 | 262 | 18866 | 1.15015045091729 | 0.0353626726307939 | 0.504718969870245 | 1.45145491942415 | 0.427480916030534 | ACTB//KLF11//CTCF//TCFL5//PRDM5//NEDD4L//IFI16//RBPJ//MDM2//NFATC2//SIRT6//OVOL2//RBL1//BACH2//RPS14//SPI1//NR2C2//NRIP1//KLF7//GTF2IRD1//NCOR2//SMG1//RPL13A//RPL18A//RPL37A//THBS1//CNOT11//CNOT8//DDX17//DYRK1A//TRA2B//TMF1//CORO1C//TERF2IP//TFRC//MOB2//PLCG2//ZNF483//DNM2//EFEMP1//SETD2//CCAR1//GATAD1//GPBP1L1//ZNF236//TRRAP//ZNF646//ZNF652//LCORL//MEIS1//THAP4//PLAGL1//RFX2//NPAS3//TADA2A//LSM14A//NF1//TAOK3//RPS27L//FAF1//SPAG9//IGF2BP3//AFF3//PCBP2//SRPK2//PUM1//SH3PXD2B//GSK3B//LDLR//ADAM19//SLIT3//PARD3//BMPR2//GAPDH//TMEM59//SCMH1//CAPRIN1//ENC1//HUWE1//RNF111//DDX11//NAT10//RNF216//PDE4D//HABP4//RNF4//ARL6IP1//CLK1//ALKBH5//PSMB5//DDX6//LARP4//KRT17//HECW2//LATS2//EYA3//MORC2//CLN6//TRIM24//CRLF3//TAF15//FANK1//BRF1//ICAM1//NUP155//FTO//GNAI2//NEMF//OPHN1//CHCHD10//LPXN//MYH9// |
| GO:1901988 | negative regulation of cell cycle phase transition | Biological process | 8 | 270 | 262 | 18866 | 2.13355951371219 | 0.035383110628595 | 0.504718969870245 | 1.45120398970073 | 0.0305343511450382 | MDM2//PLAGL1//CNOT11//CNOT8//TAOK3//PSMB5//RPS27L//RBL1// |
| GO:0019941 | modification-dependent protein catabolic process | Biological process | 15 | 643 | 262 | 18866 | 1.67980482708677 | 0.0354732484788025 | 0.504718969870245 | 1.45009903899297 | 0.0572519083969466 | HUWE1//NEDD4L//MDM2//RNF111//HECW2//USP42//UBE3B//PSMB5//GSK3B//FAF1//PCBP2//RNF216//RNF4//SIRT6//NEMF// |
| GO:0003179 | heart valve morphogenesis | Biological process | 3 | 52 | 262 | 18866 | 4.15428655314152 | 0.0355355330819538 | 0.504718969870245 | 1.44933716515713 | 0.0114503816793893 | SLIT3//MDM2//BMPR2// |
| GO:0048260 | positive regulation of receptor-mediated endocytosis | Biological process | 3 | 52 | 262 | 18866 | 4.15428655314152 | 0.0355355330819538 | 0.504718969870245 | 1.44933716515713 | 0.0114503816793893 | PLCG2//DNM2//PPT1// |
| GO:2001238 | positive regulation of extrinsic apoptotic signaling pathway | Biological process | 3 | 52 | 262 | 18866 | 4.15428655314152 | 0.0355355330819538 | 0.504718969870245 | 1.44933716515713 | 0.0114503816793893 | FAF1//THBS1//NF1// |
| GO:0043085 | positive regulation of catalytic activity | Biological process | 29 | 1472 | 262 | 18866 | 1.41862865084633 | 0.0356664418847004 | 0.505561070811446 | 1.44774021409637 | 0.110687022900763 | THBS1//RPS27L//SPAG9//DDX11//TAOK3//DDR1//GNAI2//GCLM//AGAP1//DOCK1//DOCK2//GDI2//GSK3B//ICAM1//MYO9B//NF1//OPHN1//ANKRD27//STARD13//AMBRA1//TBC1D1//CORO1C//TIAM2//RALGAPA2//RANGAP1//BCR//IFI16//PITRM1//SH3PXD2B// |
| GO:0003206 | cardiac chamber morphogenesis | Biological process | 5 | 131 | 262 | 18866 | 2.74838296136589 | 0.0361347162211344 | 0.508117453794796 | 1.44207535160266 | 0.0190839694656489 | BMPR2//RBPJ//OVOL2//MDM2//SLIT3// |
| GO:0003231 | cardiac ventricle development | Biological process | 5 | 131 | 262 | 18866 | 2.74838296136589 | 0.0361347162211344 | 0.508117453794796 | 1.44207535160266 | 0.0190839694656489 | RBPJ//DNM2//MDM2//SLIT3//BMPR2// |
| GO:0006766 | vitamin metabolic process | Biological process | 5 | 131 | 262 | 18866 | 2.74838296136589 | 0.0361347162211344 | 0.508117453794796 | 1.44207535160266 | 0.0190839694656489 | PCCB//MTR//ABCD4//SLC23A2//CYP24A1// |
| GO:0010508 | positive regulation of autophagy | Biological process | 5 | 131 | 262 | 18866 | 2.74838296136589 | 0.0361347162211344 | 0.508117453794796 | 1.44207535160266 | 0.0190839694656489 | SESN1//HUWE1//GSK3B//AMBRA1//TMEM59// |
| GO:0032465 | regulation of cytokinesis | Biological process | 4 | 90 | 262 | 18866 | 3.20033927056828 | 0.0367306830754473 | 0.510176140448102 | 1.43497099506502 | 0.0152671755725191 | CSPP1//SETD2//MYO19//KLHL21// |
| GO:0009719 | response to endogenous stimulus | Biological process | 32 | 1662 | 262 | 18866 | 1.38642856486712 | 0.0368441593317776 | 0.510176140448102 | 1.43363134826686 | 0.122137404580153 | PARD3//LPXN//GNB1//GSK3B//AP3S1//ATP6V0B//LATS2//BMPR2//RNF111//THBS1//DNM2//RBPJ//DDX17//TMF1//ICAM1//SLIT3//GNAI1//GCLM//GNAI2//MDM2//NCOR2//SESN1//PDE3A//TRIM24//LDLR//OVOL2//PDE4D//ACTB//MIA3//KLF11//RANGAP1//DDX11// |
| GO:0007420 | brain development | Biological process | 17 | 760 | 262 | 18866 | 1.61069706709522 | 0.0368620619102946 | 0.510176140448102 | 1.43342037581206 | 0.0648854961832061 | CORO1C//OPHN1//COX6B1//ACTB//NF1//PHLPP2//GSK3B//TRA2B//RBPJ//HOOK3//SETD2//MEIS1//PPT1//PTPRG//BCR//BMPR2//UGP2// |
| GO:0002313 | mature B cell differentiation involved in immune response | Biological process | 2 | 22 | 262 | 18866 | 6.54614850798057 | 0.0369746931984573 | 0.510176140448102 | 1.43209542092187 | 0.00763358778625954 | ITFG2//PLCG2// |
| GO:0007063 | regulation of sister chromatid cohesion | Biological process | 2 | 22 | 262 | 18866 | 6.54614850798057 | 0.0369746931984573 | 0.510176140448102 | 1.43209542092187 | 0.00763358778625954 | DDX11//CTCF// |
| GO:0009235 | cobalamin metabolic process | Biological process | 2 | 22 | 262 | 18866 | 6.54614850798057 | 0.0369746931984573 | 0.510176140448102 | 1.43209542092187 | 0.00763358778625954 | MTR//ABCD4// |
| GO:0010499 | proteasomal ubiquitin-independent protein catabolic process | Biological process | 2 | 22 | 262 | 18866 | 6.54614850798057 | 0.0369746931984573 | 0.510176140448102 | 1.43209542092187 | 0.00763358778625954 | PSMB5//ENC1// |
| GO:0034104 | negative regulation of tissue remodeling | Biological process | 2 | 22 | 262 | 18866 | 6.54614850798057 | 0.0369746931984573 | 0.510176140448102 | 1.43209542092187 | 0.00763358778625954 | GPR137B//BCR// |
| GO:0036344 | platelet morphogenesis | Biological process | 2 | 22 | 262 | 18866 | 6.54614850798057 | 0.0369746931984573 | 0.510176140448102 | 1.43209542092187 | 0.00763358778625954 | MYH9//WDR1// |
| GO:1903508 | positive regulation of nucleic acid-templated transcription | Biological process | 31 | 1601 | 262 | 18866 | 1.39427647796463 | 0.0371386261337729 | 0.510176140448102 | 1.43017416612013 | 0.118320610687023 | RBPJ//CTCF//DNM2//DYRK1A//NFATC2//CRLF3//RNF111//GPBP1L1//RNF4//NPAS3//SPI1//TAF15//TRIM24//FANK1//DDX17//IFI16//MEIS1//PLAGL1//OVOL2//RBL1//RFX2//BMPR2//TMF1//NR2C2//NRIP1//KLF7//BRF1//DDX11//CHCHD10//CCAR1//TADA2A// |
| GO:0006282 | regulation of DNA repair | Biological process | 5 | 132 | 262 | 18866 | 2.72756187832524 | 0.0371490886576178 | 0.510176140448102 | 1.43005183589593 | 0.0190839694656489 | SETD2//SIRT6//TERF2IP//EYA3//DDX11// |
| GO:0045744 | negative regulation of G protein-coupled receptor signaling pathway | Biological process | 3 | 53 | 262 | 18866 | 4.0759037879879 | 0.0372929435431677 | 0.510176140448102 | 1.42837333629268 | 0.0114503816793893 | DNM2//GNAI2//RPH3AL// |
| GO:0072698 | protein localization to microtubule cytoskeleton | Biological process | 3 | 53 | 262 | 18866 | 4.0759037879879 | 0.0372929435431677 | 0.510176140448102 | 1.42837333629268 | 0.0114503816793893 | CEP83//HOOK3//GSK3B// |
| GO:1900024 | regulation of substrate adhesion-dependent cell spreading | Biological process | 3 | 53 | 262 | 18866 | 4.0759037879879 | 0.0372929435431677 | 0.510176140448102 | 1.42837333629268 | 0.0114503816793893 | CORO1C//DNM2//DOCK1// |
| GO:1902680 | positive regulation of RNA biosynthetic process | Biological process | 31 | 1602 | 262 | 18866 | 1.3934061430845 | 0.0374047860474759 | 0.510716411429656 | 1.42707282504485 | 0.118320610687023 | RBPJ//CTCF//DNM2//DYRK1A//NFATC2//CRLF3//RNF111//GPBP1L1//RNF4//NPAS3//SPI1//TAF15//TRIM24//FANK1//DDX17//IFI16//MEIS1//PLAGL1//OVOL2//RBL1//RFX2//BMPR2//TMF1//NR2C2//NRIP1//KLF7//BRF1//DDX11//CHCHD10//CCAR1//TADA2A// |
| GO:0051235 | maintenance of location | Biological process | 9 | 324 | 262 | 18866 | 2.00021204410517 | 0.0377286193434288 | 0.514143482519814 | 1.42332908725862 | 0.0343511450381679 | FAF1//PDE4D//FTO//NRIP1//SLC30A7//RANGAP1//PLCG2//CHCHD10//HK1// |
| GO:0015914 | phospholipid transport | Biological process | 4 | 91 | 262 | 18866 | 3.16517070715544 | 0.0380160194339319 | 0.516067463815626 | 1.42003335902189 | 0.0152671755725191 | ATP8A1//OSBPL10//PITPNB//LDLR// |
| GO:0034103 | regulation of tissue remodeling | Biological process | 4 | 91 | 262 | 18866 | 3.16517070715544 | 0.0380160194339319 | 0.516067463815626 | 1.42003335902189 | 0.0152671755725191 | NF1//GPR137B//TFRC//BCR// |
| GO:0031344 | regulation of cell projection organization | Biological process | 16 | 706 | 262 | 18866 | 1.63190104448241 | 0.0381203979958388 | 0.516491150580856 | 1.41884257376051 | 0.0610687022900763 | DNM2//GSK3B//MOB2//ENC1//MDM2//PTPRG//NEDD4L//HECW2//BMPR2//TIAM2//CAPRIN1//ANKRD27//CORO1C//ICAM1//SDCCAG8//VDAC3// |
| GO:0060271 | cilium assembly | Biological process | 10 | 377 | 262 | 18866 | 1.91001680604208 | 0.0384448339584312 | 0.519889047725222 | 1.41516201045504 | 0.0381679389312977 | CFAP44//DYNLRB1//SDCCAG8//DYNC1H1//CEP83//VDAC3//DNM2//RABL2B//TTC39C//RFX2// |
| GO:0030099 | myeloid cell differentiation | Biological process | 11 | 431 | 262 | 18866 | 1.83778183170684 | 0.0390221410239905 | 0.526686985637378 | 1.40868890589497 | 0.0419847328244275 | RPS14//SPI1//MYH9//WDR1//IFI16//TFRC//MEIS1//RBPJ//THBS1//NF1//GPR137B// |
| GO:0003006 | developmental process involved in reproduction | Biological process | 21 | 1002 | 262 | 18866 | 1.50914202130091 | 0.0399301262816308 | 0.530995353851688 | 1.39869931631884 | 0.0801526717557252 | ICAM1//NRIP1//PDE3A//RFX2//TMF1//ADAM19//BMPR2//TCFL5//DNM2//SCMH1//ALKBH5//NR2C2//USP42//PUM1//CFAP44//NASP//DDX6//SETD2//RBPJ//OVOL2//SLIT3// |
| GO:0008380 | RNA splicing | Biological process | 12 | 487 | 262 | 18866 | 1.77431540668056 | 0.0400823256770851 | 0.530995353851688 | 1.39704708755443 | 0.0458015267175573 | SRPK2//TRA2B//DDX17//DYRK1A//PCBP2//CDC40//CCAR1//UBL5//FIP1L1//HABP4//CLK1//TAF15// |
| GO:0003283 | atrial septum development | Biological process | 2 | 23 | 262 | 18866 | 6.26153335545967 | 0.0401324859827774 | 0.530995353851688 | 1.39650393732167 | 0.00763358778625954 | BMPR2//MDM2// |
| GO:0007035 | vacuolar acidification | Biological process | 2 | 23 | 262 | 18866 | 6.26153335545967 | 0.0401324859827774 | 0.530995353851688 | 1.39650393732167 | 0.00763358778625954 | CLN6//PPT1// |
| GO:0007097 | nuclear migration | Biological process | 2 | 23 | 262 | 18866 | 6.26153335545967 | 0.0401324859827774 | 0.530995353851688 | 1.39650393732167 | 0.00763358778625954 | HOOK3//DYNC1H1// |
| GO:0051457 | maintenance of protein location in nucleus | Biological process | 2 | 23 | 262 | 18866 | 6.26153335545967 | 0.0401324859827774 | 0.530995353851688 | 1.39650393732167 | 0.00763358778625954 | RANGAP1//CHCHD10// |
| GO:0071305 | cellular response to vitamin D | Biological process | 2 | 23 | 262 | 18866 | 6.26153335545967 | 0.0401324859827774 | 0.530995353851688 | 1.39650393732167 | 0.00763358778625954 | CYP24A1//TRIM24// |
| GO:1901984 | negative regulation of protein acetylation | Biological process | 2 | 23 | 262 | 18866 | 6.26153335545967 | 0.0401324859827774 | 0.530995353851688 | 1.39650393732167 | 0.00763358778625954 | SPI1//GSK3B// |
| GO:1902001 | fatty acid transmembrane transport | Biological process | 2 | 23 | 262 | 18866 | 6.26153335545967 | 0.0401324859827774 | 0.530995353851688 | 1.39650393732167 | 0.00763358778625954 | THBS1//ABCD4// |
| GO:1903077 | negative regulation of protein localization to plasma membrane | Biological process | 2 | 23 | 262 | 18866 | 6.26153335545967 | 0.0401324859827774 | 0.530995353851688 | 1.39650393732167 | 0.00763358778625954 | PKDCC//TMEM59// |
| GO:0043632 | modification-dependent macromolecule catabolic process | Biological process | 15 | 654 | 262 | 18866 | 1.65155122907767 | 0.0401687942990227 | 0.530995353851688 | 1.39611120379583 | 0.0572519083969466 | HUWE1//NEDD4L//MDM2//RNF111//HECW2//USP42//UBE3B//PSMB5//GSK3B//FAF1//PCBP2//RNF216//RNF4//SIRT6//NEMF// |
| GO:0003158 | endothelium development | Biological process | 5 | 135 | 262 | 18866 | 2.66694939214023 | 0.040296492486433 | 0.531687739180805 | 1.39473275435718 | 0.0190839694656489 | BMPR2//RBPJ//ICAM1//PDE4D//STARD13// |
| GO:0045185 | maintenance of protein location | Biological process | 4 | 93 | 262 | 18866 | 3.09710251990479 | 0.040663364363713 | 0.533785790008359 | 1.39079669200026 | 0.0152671755725191 | FAF1//RANGAP1//CHCHD10//HK1// |
| GO:2000117 | negative regulation of cysteine-type endopeptidase activity | Biological process | 4 | 93 | 262 | 18866 | 3.09710251990479 | 0.040663364363713 | 0.533785790008359 | 1.39079669200026 | 0.0152671755725191 | ARL6IP1//MDM2//THBS1//IFI16// |
| GO:0034329 | cell junction assembly | Biological process | 11 | 434 | 262 | 18866 | 1.82507827065818 | 0.0406823565695562 | 0.533785790008359 | 1.39059389804251 | 0.0419847328244275 | NFASC//PIP5K1C//ACTB//WDR1//BCR//CORO1C//THBS1//PARD3//STRN//OPHN1//FBLIM1// |
| GO:0035065 | regulation of histone acetylation | Biological process | 3 | 55 | 262 | 18866 | 3.92768910478834 | 0.0409388383748122 | 0.534172384635489 | 1.38786448445237 | 0.0114503816793893 | TADA2A//SPI1//CTCF// |
| GO:0051452 | intracellular pH reduction | Biological process | 3 | 55 | 262 | 18866 | 3.92768910478834 | 0.0409388383748122 | 0.534172384635489 | 1.38786448445237 | 0.0114503816793893 | CLN6//PPT1//ATP6V0B// |
| GO:2000649 | regulation of sodium ion transmembrane transporter activity | Biological process | 3 | 55 | 262 | 18866 | 3.92768910478834 | 0.0409388383748122 | 0.534172384635489 | 1.38786448445237 | 0.0114503816793893 | DNM2//NEDD4L//HECW2// |
| GO:1902531 | regulation of intracellular signal transduction | Biological process | 35 | 1866 | 262 | 18866 | 1.35062549601957 | 0.0410511998690071 | 0.534486158044306 | 1.38667414454032 | 0.133587786259542 | THBS1//SPAG9//AKAP7//PIP5K1C//ITFG2//TAOK3//DNM2//NF1//MYO9B//OPHN1//BCR//ARHGEF28//STARD13//ZDHHC17//TERF2IP//TFRC//DYRK1A//MDM2//GNAI1//PDE3A//PDE4D//TIAM2//GDI2//RALGAPA2//TRIO//PHLPP2//GNAI2//ICAM1//GSK3B//PUM1//TAF15//ATRIP//TRIM24//SESN1//GPR137B// |
| GO:0014070 | response to organic cyclic compound | Biological process | 20 | 946 | 262 | 18866 | 1.52236011813502 | 0.041114319849562 | 0.534486158044306 | 1.38600688983934 | 0.0763358778625954 | GNB1//GSK3B//DDX17//TMF1//NCOR2//THBS1//CYP24A1//NASP//DNM2//MDM2//SLC3A2//SLIT3//TRIM24//PDE3A//NRIP1//ICAM1//PDE4D//ACTB//GCLM//GNAI1// |
| GO:0010629 | negative regulation of gene expression | Biological process | 49 | 2766 | 262 | 18866 | 1.27562329927749 | 0.0412480376168322 | 0.535238782237534 | 1.38459670826445 | 0.187022900763359 | CTCF//TCFL5//PRDM5//NEDD4L//IFI16//RBPJ//MDM2//NFATC2//SIRT6//OVOL2//RBL1//BACH2//RPS14//SPI1//NR2C2//NRIP1//KLF11//KLF7//GTF2IRD1//NCOR2//SMG1//RPL13A//RPL18A//RPL37A//CNOT11//CNOT8//THBS1//TMEM59//PUM1//SCMH1//IGF2BP3//GAPDH//CAPRIN1//ENC1//DDX17//PCBP2//RNF216//ALKBH5//PSMB5//DDX6//MORC2//TRIM24//DYRK1A//TAF15//NUP155//FTO//LDLR//SLIT3//TMF1// |
| GO:0043412 | macromolecule modification | Biological process | 74 | 4430 | 262 | 18866 | 1.20283631726776 | 0.0414127491157779 | 0.536390084418855 | 1.38286593863109 | 0.282442748091603 | ACTB//NAT10//NF1//TAOK3//PSMB5//SRPK2//THBS1//HUWE1//NEDD4L//WSB1//MDM2//RNF111//HECW2//UBE3B//CORO1C//TERF2IP//TFRC//MOB2//PPT1//MTO1//CTCF//ALKBH5//FTO//UBL5//RPN1//RPN2//DYRK1A//LATS2//GSK3B//PHKG1//BCR//BMPR2//TRIO//NEK9//PHLPP2//PTPRG//SIRT5//SIRT6//GALNT1//GALNT7//SPAG9//PARD3//G2E3//DCAF17//ENC1//TRIM24//KLHL21//TRRAP//PRDM5//EYA3//USP42//RANGAP1//NUP155//SETD2//TTLL11//CLK1//SMG1//EFEMP1//PKDCC//ZDHHC17//PDE4D//RNF4//TADA2A//GAPDH//DDR1//MIA3//RAB2A//SPI1//FAF1//ICAM1//GNAI2//RNF216//TMEM59//MORC2// |
| GO:0098876 | vesicle-mediated transport to the plasma membrane | Biological process | 4 | 94 | 262 | 18866 | 3.06415462075686 | 0.0420253886399512 | 0.542682193855149 | 1.3764882616266 | 0.0152671755725191 | DNM2//PKDCC//GOLPH3L//ANKRD27// |
| GO:0007160 | cell-matrix adhesion | Biological process | 7 | 230 | 262 | 18866 | 2.19153667441089 | 0.0420522963647495 | 0.542682193855149 | 1.37621028354482 | 0.0267175572519084 | NF1//DDR1//THBS1//GSK3B//BCR//CORO1C//NID1// |
| GO:1902017 | regulation of cilium assembly | Biological process | 3 | 56 | 262 | 18866 | 3.85755179934569 | 0.0428268021638882 | 0.551668606705998 | 1.3682843527439 | 0.0114503816793893 | DNM2//SDCCAG8//VDAC3// |
| GO:0090169 | regulation of spindle assembly | Biological process | 2 | 24 | 262 | 18866 | 6.00063613231552 | 0.0433882623167752 | 0.556868624898393 | 1.36262774284953 | 0.00763358778625954 | DYNC1H1//RNF4// |
| GO:1903306 | negative regulation of regulated secretory pathway | Biological process | 2 | 24 | 262 | 18866 | 6.00063613231552 | 0.0433882623167752 | 0.556868624898393 | 1.36262774284953 | 0.00763358778625954 | BCR//GNAI2// |
| GO:0097659 | nucleic acid-templated transcription | Biological process | 63 | 3701 | 262 | 18866 | 1.22574464091611 | 0.0434802230588512 | 0.557036106302052 | 1.36170823659633 | 0.240458015267176 | KLF11//CTCF//TCFL5//PRDM5//NEDD4L//IFI16//RBPJ//MDM2//NFATC2//SIRT6//OVOL2//RBL1//BACH2//RPS14//SPI1//NR2C2//NRIP1//KLF7//GTF2IRD1//NCOR2//GPBP1L1//CNOT8//BRF1//ZNF483//DNM2//EFEMP1//SETD2//TERF2IP//CCAR1//GATAD1//ZNF236//TRRAP//ZNF646//DDX17//ZNF652//LCORL//MEIS1//THAP4//PLAGL1//RFX2//NPAS3//TADA2A//PHRF1//TAF15//TRIM24//FIP1L1//GTF3C5//FAF1//SCMH1//DYRK1A//CRLF3//RNF111//RNF4//FANK1//BMPR2//TMF1//ICAM1//TFRC//PSMB5//MORC2//DDX11//CHCHD10//LPXN// |
| GO:0019882 | antigen processing and presentation | Biological process | 7 | 232 | 262 | 18866 | 2.17264411687286 | 0.0437106130980167 | 0.558973220758877 | 1.35941310192448 | 0.0267175572519084 | ICAM1//CANX//PSMB5//THBS1//IFI30//DYNC1H1//DNM2// |
| GO:0006508 | proteolysis | Biological process | 34 | 1814 | 262 | 18866 | 1.34964693604451 | 0.0441223137382159 | 0.563217744444966 | 1.35534172158559 | 0.129770992366412 | MYH9//ADAM19//HUWE1//NEDD4L//MDM2//RNF111//HECW2//USP42//UBE3B//RPS27L//PSMB5//ENC1//THBS1//GAPDH//TMEM59//PITRM1//TRRAP//ACTB//GSK3B//ARL6IP1//FAF1//PCBP2//RNF216//RNF4//CLN6//TMF1//IFI16//SIRT6//OPHN1//NEMF//PRSS57//DPP8//CFAP44//PAPPA2// |
| GO:0034654 | nucleobase-containing compound biosynthetic process | Biological process | 70 | 4175 | 262 | 18866 | 1.20731361704073 | 0.044329335409183 | 0.564838950637947 | 1.35330877964784 | 0.267175572519084 | KLF11//CTCF//TCFL5//PRDM5//NEDD4L//IFI16//RBPJ//MDM2//NFATC2//SIRT6//OVOL2//RBL1//BACH2//RPS14//SPI1//NR2C2//NRIP1//KLF7//GTF2IRD1//NCOR2//UGP2//PDE4D//DCTD//GPBP1L1//CNOT8//BRF1//ZNF483//DNM2//EFEMP1//SETD2//TERF2IP//CCAR1//GATAD1//ZNF236//TRRAP//ZNF646//DDX17//ZNF652//LCORL//MEIS1//THAP4//PLAGL1//RFX2//NPAS3//TADA2A//PHRF1//TAF15//TRIM24//FIP1L1//GTF3C5//FAF1//POLI//NAT10//ELOVL6//SCMH1//DYRK1A//CRLF3//RNF111//RNF4//FANK1//BMPR2//TMF1//PPT1//ICAM1//TFRC//PSMB5//MORC2//DDX11//CHCHD10//LPXN// |
| GO:2001234 | negative regulation of apoptotic signaling pathway | Biological process | 7 | 233 | 262 | 18866 | 2.16331946401075 | 0.0445553998030216 | 0.56620937108328 | 1.35109965498188 | 0.0267175572519084 | GSK3B//ICAM1//MDM2//GCLM//THBS1//EYA3//GNAI2// |
| GO:2000045 | regulation of G1/S transition of mitotic cell cycle | Biological process | 6 | 185 | 262 | 18866 | 2.33538271095523 | 0.0447439468847147 | 0.56620937108328 | 1.34926570922802 | 0.0229007633587786 | MDM2//PLAGL1//CNOT11//CNOT8//RPS27L//RBL1// |
| GO:0046854 | phosphatidylinositol phosphorylation | Biological process | 3 | 57 | 262 | 18866 | 3.78987545198875 | 0.0447577318408373 | 0.56620937108328 | 1.34913193005181 | 0.0114503816793893 | SMG1//PIP5K1C//PI4KA// |
| GO:0070936 | protein K48-linked ubiquitination | Biological process | 3 | 57 | 262 | 18866 | 3.78987545198875 | 0.0447577318408373 | 0.56620937108328 | 1.34913193005181 | 0.0114503816793893 | NEDD4L//RNF216//RNF4// |
| GO:0051960 | regulation of nervous system development | Biological process | 20 | 957 | 262 | 18866 | 1.50486172597254 | 0.0453919112491824 | 0.573204832751303 | 1.34302153068525 | 0.0763358778625954 | NF1//GSK3B//MOB2//ENC1//MDM2//PTPRG//DNM2//PARD3//DDX6//MEIS1//SPAG9//WDR1//NEDD4L//HECW2//BMPR2//HOOK3//TIAM2//CAPRIN1//ANKRD27//LDLR// |
| GO:0043488 | regulation of mRNA stability | Biological process | 6 | 186 | 262 | 18866 | 2.32282688992859 | 0.0457224033835081 | 0.576347224078899 | 1.33987094912623 | 0.0229007633587786 | TAF15//FTO//PUM1//IGF2BP3//ALKBH5//PSMB5// |
| GO:0007266 | Rho protein signal transduction | Biological process | 5 | 140 | 262 | 18866 | 2.57170119956379 | 0.0458923176534241 | 0.577457879350304 | 1.33826000901538 | 0.0190839694656489 | MYO9B//OPHN1//BCR//ARHGEF28//STARD13// |
| GO:0006351 | transcription, DNA-templated | Biological process | 62 | 3647 | 262 | 18866 | 1.22414951533939 | 0.0461896224649795 | 0.578081427597846 | 1.33545558748383 | 0.236641221374046 | KLF11//CTCF//TCFL5//PRDM5//NEDD4L//IFI16//RBPJ//MDM2//NFATC2//SIRT6//OVOL2//RBL1//BACH2//RPS14//SPI1//NR2C2//NRIP1//KLF7//GTF2IRD1//NCOR2//BRF1//ZNF483//DNM2//EFEMP1//SETD2//TERF2IP//CCAR1//GATAD1//GPBP1L1//ZNF236//TRRAP//CNOT8//ZNF646//DDX17//ZNF652//LCORL//MEIS1//THAP4//PLAGL1//RFX2//NPAS3//TADA2A//PHRF1//TAF15//TRIM24//FIP1L1//GTF3C5//FAF1//SCMH1//DYRK1A//CRLF3//RNF111//RNF4//FANK1//BMPR2//TMF1//ICAM1//TFRC//PSMB5//MORC2//DDX11//CHCHD10// |
| GO:0032774 | RNA biosynthetic process | Biological process | 63 | 3714 | 262 | 18866 | 1.22145420463935 | 0.0462244675899727 | 0.578081427597846 | 1.33512808231197 | 0.240458015267176 | KLF11//CTCF//TCFL5//PRDM5//NEDD4L//IFI16//RBPJ//MDM2//NFATC2//SIRT6//OVOL2//RBL1//BACH2//RPS14//SPI1//NR2C2//NRIP1//KLF7//GTF2IRD1//NCOR2//GPBP1L1//CNOT8//BRF1//ZNF483//DNM2//EFEMP1//SETD2//TERF2IP//CCAR1//GATAD1//ZNF236//TRRAP//ZNF646//DDX17//ZNF652//LCORL//MEIS1//THAP4//PLAGL1//RFX2//NPAS3//TADA2A//PHRF1//TAF15//TRIM24//FIP1L1//GTF3C5//FAF1//SCMH1//DYRK1A//CRLF3//RNF111//RNF4//FANK1//BMPR2//TMF1//ICAM1//TFRC//PSMB5//MORC2//DDX11//CHCHD10//LPXN// |
| GO:0006476 | protein deacetylation | Biological process | 4 | 97 | 262 | 18866 | 2.96938695207366 | 0.0462647992779826 | 0.578081427597846 | 1.33474931769956 | 0.0152671755725191 | PRDM5//SIRT6//DYRK1A//SIRT5// |
| GO:0006605 | protein targeting | Biological process | 11 | 444 | 262 | 18866 | 1.78397290420191 | 0.0465698015857669 | 0.578081427597846 | 1.33189561240848 | 0.0419847328244275 | PARD3//ARL6IP1//RPL13A//RPL18A//RPL37A//RPS14//CRAT//PITRM1//HUWE1//UBL5//GABARAP// |
| GO:0045851 | pH reduction | Biological process | 3 | 58 | 262 | 18866 | 3.72453277178205 | 0.0467313213641527 | 0.578081427597846 | 1.33039193879065 | 0.0114503816793893 | CLN6//PPT1//ATP6V0B// |
| GO:0000423 | mitophagy | Biological process | 2 | 25 | 262 | 18866 | 5.7606106870229 | 0.0467387144983499 | 0.578081427597846 | 1.33032323661539 | 0.00763358778625954 | HUWE1//AMBRA1// |
| GO:0039529 | RIG-I signaling pathway | Biological process | 2 | 25 | 262 | 18866 | 5.7606106870229 | 0.0467387144983499 | 0.578081427597846 | 1.33032323661539 | 0.00763358778625954 | PUM1//LSM14A// |
| GO:1904376 | negative regulation of protein localization to cell periphery | Biological process | 2 | 25 | 262 | 18866 | 5.7606106870229 | 0.0467387144983499 | 0.578081427597846 | 1.33032323661539 | 0.00763358778625954 | PKDCC//TMEM59// |
| GO:0048870 | cell motility | Biological process | 34 | 1823 | 262 | 18866 | 1.34298384091318 | 0.0467608011274076 | 0.578081427597846 | 1.33011805691499 | 0.129770992366412 | CORO1C//OVOL2//SDCCAG8//MIA3//BCR//ICAM1//OPHN1//CFAP44//BMPR2//THBS1//MCC//PTPRG//DOCK1//PLCG2//DDR1//NFATC2//TMF1//ATP8A1//GNAI2//CCAR1//SPAG9//NF1//PIP5K1C//PDE4D//SETD2//MYH9//STARD13//LPXN//SLC3A2//CD99L2//PDS5B//MDM2//WDR1//ACTB// |
| GO:0051674 | localization of cell | Biological process | 34 | 1823 | 262 | 18866 | 1.34298384091318 | 0.0467608011274076 | 0.578081427597846 | 1.33011805691499 | 0.129770992366412 | CORO1C//OVOL2//SDCCAG8//MIA3//BCR//ICAM1//OPHN1//CFAP44//BMPR2//THBS1//MCC//PTPRG//DOCK1//PLCG2//DDR1//NFATC2//TMF1//ATP8A1//GNAI2//CCAR1//SPAG9//NF1//PIP5K1C//PDE4D//SETD2//MYH9//STARD13//LPXN//ACTB//SLC3A2//CD99L2//PDS5B//MDM2//WDR1// |
| GO:0060560 | developmental growth involved in morphogenesis | Biological process | 7 | 236 | 262 | 18866 | 2.13581964031569 | 0.0471526573325857 | 0.581906657536228 | 1.32649382716083 | 0.0267175572519084 | SIRT6//DNM2//GSK3B//BMPR2//SLIT3//NEDD4L//DDR1// |
| GO:0048589 | developmental growth | Biological process | 15 | 670 | 262 | 18866 | 1.61211119972656 | 0.0477725455412972 | 0.588527746904044 | 1.32082161688946 | 0.0572519083969466 | SIRT6//EVC//DNM2//GSK3B//PDE4D//PKDCC//FTO//SH3PXD2B//LATS2//BMPR2//SLIT3//MEIS1//RBPJ//NEDD4L//DDR1// |
| GO:0048588 | developmental cell growth | Biological process | 7 | 237 | 262 | 18866 | 2.12680774309917 | 0.0480394755455323 | 0.590785118250719 | 1.31840174250403 | 0.0267175572519084 | SIRT6//DNM2//GSK3B//BMPR2//SLIT3//NEDD4L//DDR1// |
| GO:0016050 | vesicle organization | Biological process | 9 | 340 | 262 | 18866 | 1.90608441850022 | 0.048580548784348 | 0.596372309027611 | 1.3135375835476 | 0.0343511450381679 | RFX2//TMF1//HOOK3//DNM2//ANKRD27//GOLPH3L//TRAPPC4//MIA3//CORO1C// |
| GO:0010659 | cardiac muscle cell apoptotic process | Biological process | 3 | 59 | 262 | 18866 | 3.66140509768405 | 0.04874724781257 | 0.596372309027611 | 1.31204989880759 | 0.0114503816793893 | SIRT5//AMBRA1//GNB1// |
| GO:0043030 | regulation of macrophage activation | Biological process | 3 | 59 | 262 | 18866 | 3.66140509768405 | 0.04874724781257 | 0.596372309027611 | 1.31204989880759 | 0.0114503816793893 | THBS1//LDLR//GPR137B// |
| GO:1901990 | regulation of mitotic cell cycle phase transition | Biological process | 11 | 448 | 262 | 18866 | 1.76804457470011 | 0.0490800947889165 | 0.599405517499934 | 1.30909460718661 | 0.0419847328244275 | MDM2//PLAGL1//CNOT11//CNOT8//TAOK3//SDCCAG8//DYNC1H1//PSMB5//HECW2//RPS27L//RBL1// |
| GO:0019886 | antigen processing and presentation of exogenous peptide antigen via MHC class II | Biological process | 4 | 99 | 262 | 18866 | 2.90939933688025 | 0.0492186447167676 | 0.600059435329296 | 1.30787034918935 | 0.0152671755725191 | IFI30//DYNC1H1//DNM2//CANX// |
