# Supplementary material for: Differentially Expressed Circular RNAs and Their Therapeutic Mechanism in Non-segmental Vitiligo Patients Treated With Methylprednisolone
Source: Front Med (Lausanne). 2022 May 16;9:839066. doi: 10.3389/fmed.2022.839066 (PMC9149005; doi:10.3389/fmed.2022.839066)
Supplement: Supplementary file 1 [file Data_Sheet_1.ZIP › Additional files/GO Analysis Report/GO_GC_vs_control_down/CC_result(Human).html]

| GO.ID | Term | Ontology | Count | Pop.Hits | List.Total | Pop.Total | Fold.Enrichment | Pvalue | FDR | Enrichment.Score | Gene.Ratio | GENES |
| --- | --- | --- | --- | --- | --- | --- | --- | --- | --- | --- | --- | --- |
| GO:0005829 | cytosol | Cellular component | 127 | 5213 | 268 | 19559 | 1.777984000962 | 2.16235095816349e-13 | 1.8834076845604e-10 | 12.6650738169396 | 0.473880597014925 | HBG2//RPL13A//RPL18A//RPL37A//LARP4//RPS27L//RPS14//HUWE1//IFI30//DDX17//IGF2BP3//SDCCAG8//TUBGCP2//FAF1//GABARAP//OSBPL10//CCDC124//CRAT//TTLL11//DENND5B//DCTD//DDX6//DYNC1H1//DNM2//DOCK1//DOCK2//MORC2//HABP4//PHLPP2//PDS5B//SMG1//FLNB//FAM120A//ARL6IP1//TBC1D1//NEDD4L//AHCYL2//PIP5K1C//SIRT5//GAPDH//LSM14A//WSB1//TIAM2//LATS2//GDI2//SESN1//GCLM//GNAI2//GNB1//GSK3B//HK1//IFI16//KPNB1//KPNA5//KRT17//AFF3//CAPRIN1//MCC//MDH1//MDM2//MTR//MYH9//MYO9B//NF1//NFATC2//OPHN1//PCBP2//PCCB//CEP83//CRLF3//PDE3A//TRAPPC4//PDE4D//PHKG1//PI4KA//PLCG2//TERF2IP//RNF216//FBLIM1//RNF111//DPP8//ALKBH5//GOLPH3L//PPT1//CNOT11//AMBRA1//G2E3//ITFG2//PARD3//PSMB5//RALGAPA2//WDFY1//RAB2A//RANGAP1//ACTB//BACH2//PAPPA2//BCR//RPN1//ARHGEF28//SLC3A2//SRPK2//STAC//TMF1//TRIO//UGP2//DCAF17//MYO19//MOB2//NRIP1//ANKRD27//USP42//ZCCHC7//HOOK3//KLF11//TRIM24//SPAG9//STARD13//NEK9//FANK1//CNOT8//LPXN//AKAP7//GTF2IRD1//PUM1//KLHL21//WDR1// |
| GO:0005622 | intracellular | Cellular component | 251 | 15166 | 268 | 19559 | 1.20785422419749 | 5.4844955708862e-13 | 2.38849782112094e-10 | 12.2608633099042 | 0.936567164179104 | NRIP1//NCOR2//TRRAP//TADA2A//TMEM59//HUWE1//GABARAP//DNM2//ZDHHC17//PITPNB//GALNT1//RAB11FIP5//MIA3//TRAPPC4//GALNT7//GOLPH3L//RAB2A//TMF1//TERF2IP//RABL2B//HOOK3//RPN2//PSMB5//CTCF//PDS5B//RANGAP1//NAT10//RBL1//SRPK2//FANK1//TCFL5//DDX11//RBPJ//MEIS1//NASP//NFATC2//THAP4//RFX2//ACTB//BACH2//NPAS3//SPI1//NR2C2//KLF11//ENC1//KLF7//ZNF646//DDX6//MORC2//TUBGCP2//LATS2//CSPP1//LSM14A//CAPRIN1//CNOT8//PUM1//SESN1//FBLIM1//SPAG9//FLNB//MYH9//SH3PXD2B//LPXN//WDR1//DDX17//IGF2BP3//PRDM5//FAF1//AGAP1//CLK1//ZNF483//DYRK1A//EYA3//ZNF652//HABP4//SCMH1//PHLPP2//SMG1//FAM120A//TBC1D1//SIRT5//RPL13A//LCORL//GAPDH//GNAI1//SETD2//GSK3B//BRF1//IFI16//KPNB1//KPNA5//AFF3//CHCHD10//MCC//MDM2//NF1//PCBP2//RPS27L//CRLF3//PDE4D//SIRT6//RNF216//FAM193B//RNF111//ALKBH5//PPT1//CNOT11//G2E3//CCAR1//RALGAPA2//PRR12//STARD9//WDFY1//GATAD1//OVOL2//UBL5//GPBP1L1//RNF4//RPL37A//TRA2B//UGP2//VDAC3//ZNF236//FTO//TAF15//USP42//FAM120B//ADAM19//TRIM24//NEMF//NEK9//AKAP7//GTF2IRD1//PDE4DIP//NUP155//TMEM120B//RANBP17//RCL1//POLI//CYP24A1//DOCK1//NEDD4L//PIP5K1C//GNAI2//CDC40//PLAGL1//TEX10//SAGE1//ITFG2//RPS14//EFCAB6//SLC3A2//BMPR2//DCAF17//MOB2//FIP1L1//ATRIP//GTF3C5//CLN6//NOL10//ZCCHC7//TUBA1B//CDR2//SLC30A7//EVC//TBCEL//GDI2//GNB1//MYO9B//OPHN1//ACP6//TAOK3//PI4KA//CCSER2//ZCCHC2//DPP8//AMBRA1//CFAP44//PARD3//HECW2//STRN//MYO19//DYNLRB1//RPH3AL//SLC23A2//PITRM1//COX6B1//CRAT//MTO1//COQ2//HK1//PCCB//TTC19//BCKDHA//SLIT3//NDUFB2//IFI30//LDLR//SIAE//ANKRD27//SLC15A4//ABCD4//GPR137B//TFRC//ATP6V0B//ATP8A1//AHCYL2//RPN1//THBS1//ELOVL6//CANX//ARL6IP1//COL6A2//AP3S1//CEP83//PKDCC//STARD13//SDCCAG8//DYNC1H1//CCDC124//KLHL21//LARP4//OSBPL10//TTLL11//DENND5B//DCTD//DOCK2//WSB1//TIAM2//GCLM//HBG2//KRT17//MDH1//MTR//PDE3A//PHKG1//PLCG2//PAPPA2//BCR//RPL18A//ARHGEF28//STAC//TRIO//IFFO1//RCSD1//CORO1C//FRYL//PLEKHM2//RPPH1//PRSS57//NFASC// |
| GO:0043226 | organelle | Cellular component | 243 | 14482 | 268 | 19559 | 1.22458682626091 | 4.81142938879484e-12 | 1.39691833254677e-09 | 11.3177258834086 | 0.906716417910448 | NRIP1//NCOR2//TRRAP//TADA2A//TMEM59//HUWE1//GABARAP//DNM2//ZDHHC17//PITPNB//GALNT1//RAB11FIP5//MIA3//TRAPPC4//GALNT7//GOLPH3L//RAB2A//TMF1//TERF2IP//RABL2B//HOOK3//RPN2//CTCF//PDS5B//RANGAP1//NAT10//RBL1//SRPK2//FANK1//TCFL5//DDX11//RBPJ//MEIS1//NASP//NFATC2//THAP4//RFX2//ACTB//BACH2//NPAS3//SPI1//NR2C2//KLF11//ENC1//KLF7//ZNF646//DDX6//MORC2//TUBGCP2//LATS2//CSPP1//LSM14A//CAPRIN1//CNOT8//PUM1//SESN1//FBLIM1//SPAG9//FLNB//MYH9//SH3PXD2B//LPXN//WDR1//DDX17//IGF2BP3//PRDM5//FAF1//AGAP1//CLK1//ZNF483//DYRK1A//EYA3//ZNF652//HABP4//SCMH1//PHLPP2//SMG1//FAM120A//TBC1D1//SIRT5//RPL13A//LCORL//GAPDH//GNAI1//SETD2//GSK3B//BRF1//IFI16//KPNB1//KPNA5//AFF3//CHCHD10//MCC//MDM2//NF1//PCBP2//RPS27L//CRLF3//PDE4D//SIRT6//RNF216//FAM193B//RNF111//ALKBH5//PPT1//CNOT11//G2E3//CCAR1//PSMB5//RALGAPA2//PRR12//STARD9//WDFY1//GATAD1//OVOL2//UBL5//GPBP1L1//RNF4//RPL37A//TRA2B//UGP2//VDAC3//ZNF236//FTO//TAF15//USP42//FAM120B//ADAM19//TRIM24//NEMF//NEK9//AKAP7//GTF2IRD1//PDE4DIP//NUP155//TMEM120B//RANBP17//RCL1//POLI//CYP24A1//DOCK1//NEDD4L//PIP5K1C//GNAI2//CDC40//PLAGL1//TEX10//SAGE1//ITFG2//RPS14//EFCAB6//SLC3A2//BMPR2//DCAF17//MOB2//FIP1L1//ATRIP//GTF3C5//CLN6//NOL10//ZCCHC7//PITRM1//COX6B1//CRAT//MTO1//COQ2//HK1//PCCB//ACP6//TTC19//AMBRA1//BCKDHA//SLIT3//MYO19//NDUFB2//IFI30//LDLR//SIAE//ANKRD27//SLC15A4//GNB1//ABCD4//GPR137B//TFRC//ATP6V0B//ATP8A1//AHCYL2//RPN1//THBS1//ELOVL6//CANX//ARL6IP1//COL6A2//MBOAT2//NOMO1//ATP13A1//AP3S1//SLC30A7//GDI2//CEP83//PKDCC//STARD13//SDCCAG8//DYNC1H1//DYNLRB1//CCDC124//KLHL21//OSBPL10//DOCK2//TBCEL//CFAP44//PARD3//TUBA1B//TTLL11//IFFO1//KRT17//MYO9B//RCSD1//EVC//PLEKHM2//CORO1C//LARP4//BCR//RPL18A//STRN//OPHN1//CCSER2//RPH3AL//PI4KA//PRSS57//CCDC180//EFEMP1//TIAM2//ICAM1//MDH1//NID1//PLCG2//PTPRG//PAPPA2//DDR1//EMC3//HECW2//NFASC// |
| GO:0005737 | cytoplasm | Cellular component | 211 | 11620 | 268 | 19559 | 1.32522060523544 | 1.14266650329274e-11 | 2.48815631091994e-09 | 10.9420805035622 | 0.787313432835821 | TMEM59//HUWE1//GABARAP//DNM2//ZDHHC17//PITPNB//GALNT1//RAB11FIP5//MIA3//TRAPPC4//GALNT7//GOLPH3L//RAB2A//TMF1//RPN2//DDX6//LSM14A//CAPRIN1//CNOT8//PUM1//SPAG9//PITRM1//COX6B1//CRAT//CYP24A1//SIRT5//MTO1//COQ2//GSK3B//HK1//CHCHD10//PCCB//ACP6//TTC19//AMBRA1//BCKDHA//SLIT3//VDAC3//MYO19//NDUFB2//IFI30//LDLR//SIAE//PPT1//ANKRD27//SLC15A4//GNAI1//GNB1//ITFG2//ABCD4//SLC3A2//GPR137B//TFRC//CLN6//WDFY1//NEDD4L//ATP6V0B//ATP8A1//AHCYL2//RPN1//THBS1//ELOVL6//CANX//ARL6IP1//COL6A2//AP3S1//SLC30A7//HABP4//GDI2//CEP83//PLAGL1//ALKBH5//G2E3//TRRAP//ADAM19//PKDCC//PDE4DIP//HOOK3//MYH9//DDX17//IGF2BP3//SDCCAG8//TUBGCP2//FAF1//LARP4//OSBPL10//CCDC124//TTLL11//DENND5B//DCTD//DYNC1H1//DOCK1//DOCK2//MORC2//PHLPP2//PDS5B//SMG1//FLNB//FAM120A//TBC1D1//PIP5K1C//RPL13A//GAPDH//WSB1//TIAM2//LATS2//SESN1//GCLM//GNAI2//HBG2//IFI16//KPNB1//KPNA5//KRT17//AFF3//MCC//MDH1//MDM2//MTR//MYO9B//NF1//NFATC2//OPHN1//PCBP2//CRLF3//PDE3A//PDE4D//PHKG1//PI4KA//PLCG2//TERF2IP//RNF216//FBLIM1//RNF111//DPP8//CNOT11//PARD3//PSMB5//RALGAPA2//RANGAP1//ACTB//BACH2//PAPPA2//BCR//RPL18A//RPL37A//RPS14//ARHGEF28//SRPK2//STAC//TRIO//UGP2//DCAF17//MOB2//NRIP1//USP42//ZCCHC7//KLF11//TRIM24//STARD13//NEK9//FANK1//LPXN//AKAP7//GTF2IRD1//KLHL21//WDR1//TUBA1B//CORO1C//FRYL//PLEKHM2//RPS27L//RPH3AL//PRSS57//POLI//CCAR1//TRA2B//NFASC//CDR2//RABL2B//AGAP1//CLK1//DDX11//DYRK1A//EVC//EYA3//TBCEL//SH3PXD2B//RBPJ//NASP//TAOK3//SIRT6//CCSER2//FAM193B//ZCCHC2//TEX10//CFAP44//STARD9//HECW2//UBL5//RFX2//RNF4//RANBP17//BMPR2//STRN//FTO//CSPP1//TAF15//DYNLRB1//ENC1//SLC23A2// |
| GO:0043229 | intracellular organelle | Cellular component | 227 | 13340 | 268 | 19559 | 1.24188642618989 | 4.02613634311506e-10 | 7.01352950970643e-08 | 9.3951115220306 | 0.847014925373134 | NRIP1//NCOR2//TRRAP//TADA2A//TMEM59//HUWE1//GABARAP//DNM2//ZDHHC17//PITPNB//GALNT1//RAB11FIP5//MIA3//TRAPPC4//GALNT7//GOLPH3L//RAB2A//TMF1//TERF2IP//RABL2B//HOOK3//RPN2//CTCF//PDS5B//RANGAP1//NAT10//RBL1//SRPK2//FANK1//TCFL5//DDX11//RBPJ//MEIS1//NASP//NFATC2//THAP4//RFX2//ACTB//BACH2//NPAS3//SPI1//NR2C2//KLF11//ENC1//KLF7//ZNF646//DDX6//MORC2//TUBGCP2//LATS2//CSPP1//LSM14A//CAPRIN1//CNOT8//PUM1//SESN1//FBLIM1//SPAG9//FLNB//MYH9//SH3PXD2B//LPXN//WDR1//DDX17//IGF2BP3//PRDM5//FAF1//AGAP1//CLK1//ZNF483//DYRK1A//EYA3//ZNF652//HABP4//SCMH1//PHLPP2//SMG1//FAM120A//TBC1D1//SIRT5//RPL13A//LCORL//GAPDH//GNAI1//SETD2//GSK3B//BRF1//IFI16//KPNB1//KPNA5//AFF3//CHCHD10//MCC//MDM2//NF1//PCBP2//RPS27L//CRLF3//PDE4D//SIRT6//RNF216//FAM193B//RNF111//ALKBH5//PPT1//CNOT11//G2E3//CCAR1//PSMB5//RALGAPA2//PRR12//STARD9//WDFY1//GATAD1//OVOL2//UBL5//GPBP1L1//RNF4//RPL37A//TRA2B//UGP2//VDAC3//ZNF236//FTO//TAF15//USP42//FAM120B//ADAM19//TRIM24//NEMF//NEK9//AKAP7//GTF2IRD1//PDE4DIP//NUP155//TMEM120B//RANBP17//RCL1//POLI//CYP24A1//DOCK1//NEDD4L//PIP5K1C//GNAI2//CDC40//PLAGL1//TEX10//SAGE1//ITFG2//RPS14//EFCAB6//SLC3A2//BMPR2//DCAF17//MOB2//FIP1L1//ATRIP//GTF3C5//CLN6//NOL10//ZCCHC7//PITRM1//COX6B1//CRAT//MTO1//COQ2//HK1//PCCB//ACP6//TTC19//AMBRA1//BCKDHA//SLIT3//MYO19//NDUFB2//IFI30//LDLR//SIAE//ANKRD27//SLC15A4//GNB1//ABCD4//GPR137B//TFRC//ATP6V0B//ATP8A1//AHCYL2//RPN1//THBS1//ELOVL6//CANX//ARL6IP1//COL6A2//AP3S1//SLC30A7//GDI2//CEP83//PKDCC//STARD13//SDCCAG8//DYNC1H1//DYNLRB1//CCDC124//KLHL21//OSBPL10//DOCK2//TBCEL//CFAP44//PARD3//TUBA1B//TTLL11//IFFO1//KRT17//MYO9B//RCSD1//PLEKHM2//CORO1C//LARP4//OPHN1//CCSER2//RPL18A//RPH3AL//PI4KA//PRSS57//EVC//HECW2//NFASC// |
| GO:0043228 | non-membrane-bounded organelle | Cellular component | 118 | 5784 | 268 | 19559 | 1.48890015689189 | 3.15147325941914e-07 | 4.40646830881626e-05 | 6.50148637355692 | 0.440298507462687 | TERF2IP//RABL2B//HOOK3//CTCF//PDS5B//RANGAP1//NAT10//RBL1//SRPK2//FANK1//TCFL5//DDX11//RBPJ//MEIS1//NASP//NFATC2//THAP4//RFX2//ACTB//BACH2//NPAS3//SPI1//NR2C2//NRIP1//KLF11//ENC1//KLF7//NCOR2//ZNF646//DDX6//MORC2//TRRAP//TUBGCP2//LATS2//CSPP1//LSM14A//CAPRIN1//CNOT8//PUM1//SESN1//FBLIM1//FLNB//MYH9//SH3PXD2B//LPXN//WDR1//SETD2//TADA2A//TRIM24//RCL1//DDX17//PRDM5//HABP4//RPL13A//IFI16//MDM2//NF1//FAM193B//TEX10//CLN6//G2E3//RPS14//NOL10//DCAF17//MOB2//ZCCHC7//GAPDH//STARD13//SDCCAG8//DYNC1H1//DNM2//EYA3//GNAI1//GNAI2//GSK3B//TTC19//PSMB5//DYNLRB1//NEK9//PDE4DIP//CEP83//STARD9//CCDC124//KLHL21//CANX//OSBPL10//DOCK2//DYRK1A//TBCEL//CFAP44//PARD3//TUBA1B//GABARAP//TTLL11//IFFO1//KRT17//MYO9B//RCSD1//AMBRA1//SCMH1//LARP4//KPNB1//CORO1C//OPHN1//MYO19//CCSER2//RPL18A//RPL37A//RPS27L//RAB11FIP5//SPAG9//EVC//POLI//MCC//HECW2//BCR//SIRT6//RNF4// |
| GO:0043227 | membrane-bounded organelle | Cellular component | 219 | 13330 | 268 | 19559 | 1.19901831801234 | 3.54136373842868e-07 | 4.40646830881626e-05 | 6.45082946395651 | 0.817164179104478 | NRIP1//NCOR2//TRRAP//TADA2A//TMEM59//HUWE1//GABARAP//DNM2//ZDHHC17//PITPNB//GALNT1//RAB11FIP5//MIA3//TRAPPC4//GALNT7//GOLPH3L//RAB2A//TMF1//TERF2IP//RPN2//NAT10//CTCF//TCFL5//DDX11//RBPJ//MEIS1//NASP//NFATC2//THAP4//RFX2//ACTB//BACH2//NPAS3//SPI1//NR2C2//KLF11//ENC1//KLF7//ZNF646//SESN1//FBLIM1//SPAG9//DDX17//IGF2BP3//PRDM5//FAF1//AGAP1//CLK1//ZNF483//DDX6//DYRK1A//EYA3//ZNF652//MORC2//HABP4//SCMH1//PHLPP2//PDS5B//SMG1//FLNB//FAM120A//TBC1D1//SIRT5//RPL13A//LCORL//GAPDH//LATS2//GNAI1//SETD2//GSK3B//BRF1//IFI16//KPNB1//KPNA5//AFF3//CHCHD10//MCC//MDM2//MYH9//NF1//PCBP2//RPS27L//CRLF3//PDE4D//SIRT6//RNF216//FAM193B//RNF111//ALKBH5//PPT1//CNOT11//G2E3//CCAR1//PSMB5//RALGAPA2//PRR12//STARD9//WDFY1//GATAD1//OVOL2//UBL5//GPBP1L1//RNF4//RPL37A//TRA2B//SRPK2//UGP2//VDAC3//ZNF236//FTO//TAF15//USP42//FAM120B//ADAM19//TRIM24//NEMF//NEK9//FANK1//CNOT8//LPXN//AKAP7//GTF2IRD1//PDE4DIP//RANGAP1//NUP155//TMEM120B//RANBP17//RCL1//TUBGCP2//POLI//CYP24A1//DOCK1//NEDD4L//PIP5K1C//GNAI2//CDC40//PLAGL1//TEX10//SAGE1//ITFG2//RBL1//RPS14//EFCAB6//SLC3A2//BMPR2//DCAF17//MOB2//FIP1L1//ATRIP//GTF3C5//PUM1//CLN6//NOL10//ZCCHC7//PITRM1//COX6B1//CRAT//MTO1//COQ2//HK1//PCCB//ACP6//TTC19//AMBRA1//BCKDHA//SLIT3//MYO19//NDUFB2//IFI30//LDLR//SIAE//ANKRD27//SLC15A4//GNB1//ABCD4//GPR137B//TFRC//ATP6V0B//ATP8A1//AHCYL2//RPN1//THBS1//ELOVL6//CANX//ARL6IP1//COL6A2//MBOAT2//NOMO1//ATP13A1//AP3S1//SLC30A7//GDI2//CEP83//PKDCC//HOOK3//PLEKHM2//CORO1C//RPH3AL//PI4KA//STARD13//DYNC1H1//PRSS57//DOCK2//OSBPL10//EVC//CCDC180//EFEMP1//TIAM2//ICAM1//MDH1//NID1//PLCG2//PTPRG//PAPPA2//BCR//DDR1//WDR1//EMC3//NFASC// |
| GO:0043232 | intracellular non-membrane-bounded organelle | Cellular component | 117 | 5775 | 268 | 19559 | 1.47858305873231 | 5.51416278600276e-07 | 5.92290835606212e-05 | 6.25852041704432 | 0.436567164179104 | TERF2IP//RABL2B//HOOK3//CTCF//PDS5B//RANGAP1//NAT10//RBL1//SRPK2//FANK1//TCFL5//DDX11//RBPJ//MEIS1//NASP//NFATC2//THAP4//RFX2//ACTB//BACH2//NPAS3//SPI1//NR2C2//NRIP1//KLF11//ENC1//KLF7//NCOR2//ZNF646//DDX6//MORC2//TRRAP//TUBGCP2//LATS2//CSPP1//LSM14A//CAPRIN1//CNOT8//PUM1//SESN1//FBLIM1//FLNB//MYH9//SH3PXD2B//LPXN//WDR1//SETD2//TADA2A//TRIM24//RCL1//DDX17//PRDM5//HABP4//RPL13A//IFI16//MDM2//NF1//FAM193B//TEX10//CLN6//G2E3//RPS14//NOL10//DCAF17//MOB2//ZCCHC7//GAPDH//STARD13//SDCCAG8//DYNC1H1//DNM2//EYA3//GNAI1//GNAI2//GSK3B//TTC19//PSMB5//DYNLRB1//NEK9//PDE4DIP//CEP83//STARD9//CCDC124//KLHL21//CANX//OSBPL10//DOCK2//DYRK1A//TBCEL//CFAP44//PARD3//TUBA1B//GABARAP//TTLL11//IFFO1//KRT17//MYO9B//RCSD1//AMBRA1//SCMH1//LARP4//KPNB1//CORO1C//OPHN1//MYO19//CCSER2//RPL18A//RPL37A//RPS27L//RAB11FIP5//SPAG9//EVC//POLI//MCC//HECW2//SIRT6//RNF4// |
| GO:0043231 | intracellular membrane-bounded organelle | Cellular component | 196 | 11502 | 268 | 19559 | 1.24363965254583 | 6.12011196378405e-07 | 5.92290835606212e-05 | 6.21324063262424 | 0.73134328358209 | NRIP1//NCOR2//TRRAP//TADA2A//TMEM59//HUWE1//GABARAP//DNM2//ZDHHC17//PITPNB//GALNT1//RAB11FIP5//MIA3//TRAPPC4//GALNT7//GOLPH3L//RAB2A//TMF1//TERF2IP//RPN2//NAT10//CTCF//TCFL5//DDX11//RBPJ//MEIS1//NASP//NFATC2//THAP4//RFX2//ACTB//BACH2//NPAS3//SPI1//NR2C2//KLF11//ENC1//KLF7//ZNF646//SESN1//FBLIM1//DDX17//IGF2BP3//PRDM5//FAF1//AGAP1//CLK1//ZNF483//DDX6//DYRK1A//EYA3//ZNF652//MORC2//HABP4//SCMH1//PHLPP2//PDS5B//SMG1//FLNB//FAM120A//TBC1D1//SIRT5//RPL13A//LCORL//GAPDH//LATS2//GNAI1//SETD2//GSK3B//BRF1//IFI16//KPNB1//KPNA5//AFF3//CHCHD10//MCC//MDM2//MYH9//NF1//PCBP2//RPS27L//CRLF3//PDE4D//SIRT6//RNF216//FAM193B//RNF111//ALKBH5//PPT1//CNOT11//G2E3//CCAR1//PSMB5//RALGAPA2//PRR12//STARD9//WDFY1//GATAD1//OVOL2//UBL5//GPBP1L1//RNF4//RPL37A//TRA2B//SRPK2//UGP2//VDAC3//ZNF236//FTO//TAF15//USP42//FAM120B//ADAM19//TRIM24//NEMF//NEK9//FANK1//CNOT8//LPXN//AKAP7//GTF2IRD1//PDE4DIP//RANGAP1//NUP155//TMEM120B//RANBP17//RCL1//TUBGCP2//POLI//CYP24A1//DOCK1//NEDD4L//PIP5K1C//GNAI2//CDC40//PLAGL1//TEX10//SAGE1//ITFG2//RBL1//RPS14//EFCAB6//SLC3A2//BMPR2//DCAF17//MOB2//FIP1L1//ATRIP//GTF3C5//PUM1//CLN6//NOL10//ZCCHC7//PITRM1//COX6B1//CRAT//MTO1//COQ2//HK1//PCCB//ACP6//TTC19//AMBRA1//BCKDHA//SLIT3//MYO19//NDUFB2//IFI30//LDLR//SIAE//ANKRD27//SLC15A4//GNB1//ABCD4//GPR137B//SPAG9//ATP6V0B//ATP8A1//AHCYL2//RPN1//THBS1//ELOVL6//CANX//ARL6IP1//COL6A2//AP3S1//SLC30A7//GDI2//CEP83//PKDCC//HOOK3//STARD13//DYNC1H1//PRSS57//OSBPL10//TFRC// |
| GO:0005856 | cytoskeleton | Cellular component | 57 | 2268 | 268 | 19559 | 1.83418719892601 | 3.779301805831e-06 | 0.00032917718728788 | 5.42258842499929 | 0.212686567164179 | RABL2B//HOOK3//TUBGCP2//DDX11//LATS2//CSPP1//DDX6//FLNB//MYH9//FBLIM1//SH3PXD2B//LPXN//WDR1//SDCCAG8//DYNC1H1//DNM2//EYA3//GNAI1//GNAI2//GSK3B//TTC19//PSMB5//DYNLRB1//NEK9//PDE4DIP//CEP83//STARD9//CCDC124//KLHL21//TUBA1B//GABARAP//TTLL11//IFFO1//KRT17//MYO9B//ACTB//RCSD1//AMBRA1//CORO1C//OPHN1//MYO19//GAPDH//CCSER2//RAB11FIP5//SPAG9//EVC//FANK1//HECW2//RANGAP1//RNF4//OSBPL10//DOCK2//DYRK1A//TBCEL//CFAP44//PARD3//ENC1// |
| GO:0005925 | focal adhesion | Cellular component | 18 | 415 | 268 | 19559 | 3.1654558532638 | 1.83002226217635e-05 | 0.00144904490032327 | 4.73754362705562 | 0.0671641791044776 | DNM2//NFASC//FLNB//PIP5K1C//RPL13A//CORO1C//GDI2//ICAM1//MYH9//PCBP2//PI4KA//FBLIM1//ACTB//RPL37A//RPS14//CD99L2//KLF11//LPXN// |
| GO:0005654 | nucleoplasm | Cellular component | 83 | 3978 | 268 | 19559 | 1.522737931759 | 2.30906386749841e-05 | 0.00157935575416229 | 4.63656405455856 | 0.309701492537313 | NRIP1//NCOR2//TRRAP//TADA2A//FIP1L1//AFF3//HABP4//PRDM5//MDM2//PLAGL1//TERF2IP//SAGE1//RNF4//KLF11//RNF111//DDX17//POLI//DOCK1//DYRK1A//IFI16//CDC40//FAM193B//ALKBH5//SRPK2//FTO//LPXN//ACTB//TEX10//HUWE1//RCL1//CTCF//TUBGCP2//FAF1//CYP24A1//DDX11//EYA3//MORC2//SCMH1//PDS5B//SMG1//NEDD4L//PIP5K1C//SESN1//GNAI2//SETD2//GSK3B//BRF1//RBPJ//KPNB1//KPNA5//MCC//NASP//NFATC2//PCBP2//SIRT6//RNF216//NAT10//CCAR1//ITFG2//PSMB5//GATAD1//RANGAP1//RBL1//BACH2//RPS14//NPAS3//TRA2B//EFCAB6//SLC3A2//BMPR2//SPI1//NR2C2//DCAF17//TAF15//MOB2//ATRIP//USP42//ENC1//TRIM24//FANK1//GTF3C5//GTF2IRD1//PUM1// |
| GO:0030055 | cell-substrate junction | Cellular component | 18 | 423 | 268 | 19559 | 3.10558907589711 | 2.35724739427207e-05 | 0.00157935575416229 | 4.62759483566449 | 0.0671641791044776 | DNM2//NFASC//FLNB//PIP5K1C//RPL13A//CORO1C//GDI2//ICAM1//MYH9//PCBP2//PI4KA//FBLIM1//ACTB//RPL37A//RPS14//CD99L2//KLF11//LPXN// |
| GO:0005815 | microtubule organizing center | Cellular component | 26 | 784 | 268 | 19559 | 2.42029964971063 | 3.09652220619286e-05 | 0.00192647917256713 | 4.50912580117276 | 0.0970149253731343 | RABL2B//HOOK3//TUBGCP2//SDCCAG8//DDX11//DYNC1H1//DNM2//EYA3//GNAI1//GNAI2//GSK3B//TTC19//PSMB5//CSPP1//DYNLRB1//NEK9//PDE4DIP//CEP83//STARD9//RAB11FIP5//LATS2//SPAG9//TTLL11//EVC//FANK1//CCDC124// |
| GO:0015630 | microtubule cytoskeleton | Cellular component | 35 | 1258 | 268 | 19559 | 2.03048252378805 | 5.20024431210456e-05 | 0.00301960853056205 | 4.28397625234474 | 0.130597014925373 | RABL2B//HOOK3//TUBGCP2//DDX11//LATS2//CSPP1//SDCCAG8//DYNC1H1//DNM2//EYA3//GNAI1//GNAI2//GSK3B//TTC19//PSMB5//DYNLRB1//NEK9//PDE4DIP//CEP83//STARD9//CCDC124//MYH9//KLHL21//TUBA1B//GABARAP//TTLL11//RAB11FIP5//SPAG9//EVC//FANK1//HECW2//RANGAP1//RNF4//GAPDH//CCSER2// |
| GO:0005634 | nucleus | Cellular component | 142 | 8076 | 268 | 19559 | 1.28322817561524 | 6.65035001831741e-05 | 0.00353396695669602 | 4.17715549649788 | 0.529850746268657 | NRIP1//NCOR2//TRRAP//TADA2A//TERF2IP//NAT10//CTCF//TCFL5//DDX11//RBPJ//MEIS1//NASP//NFATC2//THAP4//RFX2//ACTB//BACH2//NPAS3//SPI1//NR2C2//KLF11//ENC1//KLF7//ZNF646//SESN1//FBLIM1//FAF1//KPNB1//RANGAP1//NUP155//TMEM120B//TRA2B//CCAR1//RANBP17//HUWE1//RCL1//DDX17//TUBGCP2//PRDM5//POLI//CYP24A1//DOCK1//DYRK1A//EYA3//MORC2//HABP4//SCMH1//PDS5B//SMG1//NEDD4L//PIP5K1C//GNAI2//SETD2//GSK3B//BRF1//IFI16//KPNA5//AFF3//MCC//MDM2//PCBP2//CDC40//SIRT6//PLAGL1//RNF216//FAM193B//RNF111//TEX10//ALKBH5//SAGE1//ITFG2//PSMB5//GATAD1//RBL1//RNF4//RPS14//EFCAB6//SLC3A2//BMPR2//SRPK2//FTO//DCAF17//TAF15//MOB2//FIP1L1//ATRIP//USP42//TRIM24//FANK1//GTF3C5//GTF2IRD1//PUM1//DDX6//RPL13A//NF1//CLN6//G2E3//NOL10//ZCCHC7//MYH9//LPXN//GAPDH//IGF2BP3//AGAP1//CLK1//ZNF483//DNM2//ZNF652//PHLPP2//FLNB//FAM120A//TBC1D1//SIRT5//LCORL//LATS2//GNAI1//CHCHD10//RPS27L//CRLF3//PDE4D//PPT1//CNOT11//RALGAPA2//PRR12//STARD9//WDFY1//OVOL2//RAB2A//UBL5//GPBP1L1//RPL37A//TMF1//UGP2//VDAC3//ZNF236//FAM120B//ADAM19//NEMF//NEK9//CNOT8//AKAP7//PDE4DIP// |
| GO:0010494 | cytoplasmic stress granule | Cellular component | 7 | 74 | 268 | 19559 | 6.90364058087939 | 6.89752448494056e-05 | 0.00353396695669602 | 4.16130674918068 | 0.0261194029850746 | LARP4//DDX6//HABP4//LSM14A//KPNB1//CAPRIN1//PUM1// |
| GO:0036464 | cytoplasmic ribonucleoprotein granule | Cellular component | 12 | 233 | 268 | 19559 | 3.75869579142912 | 9.4288994837909e-05 | 0.0045625396946566 | 4.02553899400797 | 0.0447761194029851 | DDX6//LSM14A//CAPRIN1//CNOT8//PUM1//LARP4//HABP4//KPNB1//POLI//MCC//PARD3//ACTB// |
| GO:0035770 | ribonucleoprotein granule | Cellular component | 12 | 243 | 268 | 19559 | 3.60401695227566 | 0.000140079616408142 | 0.00642154452060483 | 3.85362505618828 | 0.0447761194029851 | DDX6//LSM14A//CAPRIN1//CNOT8//PUM1//LARP4//HABP4//KPNB1//POLI//MCC//PARD3//ACTB// |
| GO:0005813 | centrosome | Cellular component | 20 | 583 | 268 | 19559 | 2.50364814008858 | 0.000167315339158876 | 0.00728658302036905 | 3.77646424190263 | 0.0746268656716418 | RABL2B//HOOK3//SDCCAG8//RAB11FIP5//LATS2//SPAG9//TUBGCP2//DDX11//DYNC1H1//DNM2//EYA3//GNAI1//GNAI2//GSK3B//TTC19//PSMB5//CSPP1//DYNLRB1//NEK9//PDE4DIP// |
| GO:0005773 | vacuole | Cellular component | 23 | 801 | 268 | 19559 | 2.09559412674455 | 0.000698840803318819 | 0.0289852542709853 | 3.15562174573423 | 0.0858208955223881 | GABARAP//RPN2//IFI30//LDLR//SIAE//PPT1//ANKRD27//TMEM59//SLC15A4//GNAI1//GNB1//ITFG2//ABCD4//RAB2A//SLC3A2//GPR137B//SPAG9//ATP6V0B//AMBRA1//ATP8A1//DYNC1H1//GDI2//PRSS57// |
| GO:0016604 | nuclear body | Cellular component | 23 | 810 | 268 | 19559 | 2.0723097475585 | 0.000812313201766075 | 0.031127181988742 | 3.09027648852662 | 0.0858208955223881 | HABP4//RNF111//RNF4//DDX17//POLI//DOCK1//DYRK1A//IFI16//CDC40//FAM193B//ALKBH5//SRPK2//FTO//NRIP1//LPXN//PRDM5//AFF3//MDM2//PLAGL1//TERF2IP//SAGE1//KLF11//NCOR2// |
| GO:0030054 | cell junction | Cellular component | 46 | 2093 | 268 | 19559 | 1.60398556667213 | 0.000841511630491722 | 0.031127181988742 | 3.07493987728356 | 0.171641791044776 | SDCCAG8//PARD3//ACTB//WDR1//PIP5K1C//MYH9//BMPR2//CD99L2//STRN//DNM2//NFASC//FLNB//RPL13A//CORO1C//GDI2//ICAM1//PCBP2//PI4KA//FBLIM1//RPL37A//RPS14//KLF11//LPXN//TRAPPC4//PPT1//PRR12//BCR//RPL18A//ATP8A1//ZDHHC17//OPHN1//CANX//GABARAP//TIAM2//GNAI2//GNB1//CAPRIN1//MDM2//VDAC3//RNF216//AKAP7//NF1//GSK3B//IFI30//SH3PXD2B//SLC3A2// |
| GO:0005774 | vacuolar membrane | Cellular component | 15 | 427 | 268 | 19559 | 2.56374742213989 | 0.000857695026096221 | 0.031127181988742 | 3.06666710845474 | 0.0559701492537313 | GABARAP//RPN2//SLC15A4//GNAI1//GNB1//ITFG2//ABCD4//RAB2A//SLC3A2//GPR137B//SPAG9//TMEM59//ATP8A1//LDLR//ATP6V0B// |
| GO:0032991 | protein-containing complex | Cellular component | 99 | 5501 | 268 | 19559 | 1.31342537449093 | 0.00100108019023651 | 0.03487763382784 | 2.99953113252834 | 0.369402985074627 | NRIP1//NCOR2//TRRAP//TADA2A//BRF1//GTF3C5//PSMB5//TERF2IP//TUBGCP2//RBPJ//THBS1//COL6A2//KPNB1//RANGAP1//RANBP17//EYA3//MEIS1//NFATC2//RBL1//SPI1//TRA2B//NAT10//NDUFB2//ARL6IP1//HBG2//GNAI1//GNAI2//GNB1//LARP4//FIP1L1//DYNC1H1//DYNLRB1//GABARAP//PDE4D//BCKDHA//PHKG1//AFF3//MYH9//RPN1//RPN2//RPL13A//DDX6//MYO9B//MYO19//CORO1C//GCLM//RPL18A//RPL37A//RPS27L//RPS14//TRAPPC4//CNOT11//CNOT8//AP3S1//RPPH1//GSK3B//HOOK3//FAF1//DDX11//ENC1//KLHL21//SMG1//SESN1//SAGE1//NOL10//DOCK1//ATP6V0B//LDLR//ACTB//ABCD4//BMPR2//DDR1//NUP155//COX6B1//VDAC3//CHCHD10//CDC40//TEX10//EMC3//DCAF17//STRN//GAPDH//CEP83//EVC//ITFG2//NEMF//LSM14A//SLC3A2//TFRC//DDX17//DYRK1A//DNM2//MDM2//NASP//RNF111//PARD3//BCR//CANX//AKAP7// |
| GO:0099080 | supramolecular complex | Cellular component | 32 | 1312 | 268 | 19559 | 1.7800327630142 | 0.00110061238662481 | 0.0368111137253576 | 2.95836560381586 | 0.119402985074627 | RANGAP1//DDX6//LSM14A//CAPRIN1//CNOT8//PUM1//KLHL21//TUBA1B//GABARAP//TTLL11//DYNC1H1//DNM2//CSPP1//DYNLRB1//HOOK3//TUBGCP2//IFFO1//KRT17//MYO9B//ACTB//RCSD1//LARP4//HABP4//KPNB1//PDE4DIP//CORO1C//FLNB//POLI//MCC//PARD3//MYH9//RNF4// |
| GO:0070161 | anchoring junction | Cellular component | 23 | 831 | 268 | 19559 | 2.01994090917255 | 0.00114110226243933 | 0.0368111137253576 | 2.94267543356337 | 0.0858208955223881 | SDCCAG8//PARD3//ACTB//WDR1//PIP5K1C//MYH9//BMPR2//CD99L2//STRN//DNM2//NFASC//FLNB//RPL13A//CORO1C//GDI2//ICAM1//PCBP2//PI4KA//FBLIM1//RPL37A//RPS14//KLF11//LPXN// |
| GO:0031974 | membrane-enclosed lumen | Cellular component | 114 | 6574 | 268 | 19559 | 1.26557242688293 | 0.00134017335629029 | 0.0389096997776281 | 2.87283902043318 | 0.425373134328358 | NRIP1//NCOR2//TRRAP//TADA2A//TERF2IP//NAT10//CTCF//TCFL5//DDX11//RBPJ//MEIS1//NASP//NFATC2//THAP4//RFX2//ACTB//BACH2//NPAS3//SPI1//NR2C2//KLF11//ENC1//KLF7//ZNF646//SESN1//FBLIM1//CCAR1//HUWE1//RCL1//DDX17//TUBGCP2//PRDM5//FAF1//POLI//CYP24A1//DOCK1//DYRK1A//EYA3//MORC2//HABP4//SCMH1//PDS5B//SMG1//NEDD4L//PIP5K1C//GNAI2//SETD2//GSK3B//BRF1//IFI16//KPNB1//KPNA5//AFF3//MCC//MDM2//PCBP2//CDC40//SIRT6//PLAGL1//RNF216//FAM193B//RNF111//TEX10//ALKBH5//SAGE1//ITFG2//PSMB5//GATAD1//RANGAP1//RBL1//RNF4//RPS14//TRA2B//EFCAB6//SLC3A2//BMPR2//SRPK2//FTO//DCAF17//TAF15//MOB2//FIP1L1//ATRIP//USP42//TRIM24//FANK1//GTF3C5//GTF2IRD1//PUM1//DDX6//RPL13A//NF1//CLN6//G2E3//NOL10//ZCCHC7//SIRT5//CHCHD10//PITRM1//PCCB//ACP6//BCKDHA//CRAT//COL6A2//MIA3//THBS1//CANX//LPXN//GDI2//DYNC1H1//PRSS57//DOCK2//IFI30//PPT1// |
| GO:0043233 | organelle lumen | Cellular component | 114 | 6574 | 268 | 19559 | 1.26557242688293 | 0.00134017335629029 | 0.0389096997776281 | 2.87283902043318 | 0.425373134328358 | NRIP1//NCOR2//TRRAP//TADA2A//TERF2IP//NAT10//CTCF//TCFL5//DDX11//RBPJ//MEIS1//NASP//NFATC2//THAP4//RFX2//ACTB//BACH2//NPAS3//SPI1//NR2C2//KLF11//ENC1//KLF7//ZNF646//SESN1//FBLIM1//CCAR1//HUWE1//RCL1//DDX17//TUBGCP2//PRDM5//FAF1//POLI//CYP24A1//DOCK1//DYRK1A//EYA3//MORC2//HABP4//SCMH1//PDS5B//SMG1//NEDD4L//PIP5K1C//GNAI2//SETD2//GSK3B//BRF1//IFI16//KPNB1//KPNA5//AFF3//MCC//MDM2//PCBP2//CDC40//SIRT6//PLAGL1//RNF216//FAM193B//RNF111//TEX10//ALKBH5//SAGE1//ITFG2//PSMB5//GATAD1//RANGAP1//RBL1//RNF4//RPS14//TRA2B//EFCAB6//SLC3A2//BMPR2//SRPK2//FTO//DCAF17//TAF15//MOB2//FIP1L1//ATRIP//USP42//TRIM24//FANK1//GTF3C5//GTF2IRD1//PUM1//DDX6//RPL13A//NF1//CLN6//G2E3//NOL10//ZCCHC7//SIRT5//CHCHD10//PITRM1//PCCB//ACP6//BCKDHA//CRAT//COL6A2//MIA3//THBS1//CANX//LPXN//GDI2//DYNC1H1//PRSS57//DOCK2//IFI30//PPT1// |
| GO:0070013 | intracellular organelle lumen | Cellular component | 114 | 6574 | 268 | 19559 | 1.26557242688293 | 0.00134017335629029 | 0.0389096997776281 | 2.87283902043318 | 0.425373134328358 | NRIP1//NCOR2//TRRAP//TADA2A//TERF2IP//NAT10//CTCF//TCFL5//DDX11//RBPJ//MEIS1//NASP//NFATC2//THAP4//RFX2//ACTB//BACH2//NPAS3//SPI1//NR2C2//KLF11//ENC1//KLF7//ZNF646//SESN1//FBLIM1//CCAR1//HUWE1//RCL1//DDX17//TUBGCP2//PRDM5//FAF1//POLI//CYP24A1//DOCK1//DYRK1A//EYA3//MORC2//HABP4//SCMH1//PDS5B//SMG1//NEDD4L//PIP5K1C//GNAI2//SETD2//GSK3B//BRF1//IFI16//KPNB1//KPNA5//AFF3//MCC//MDM2//PCBP2//CDC40//SIRT6//PLAGL1//RNF216//FAM193B//RNF111//TEX10//ALKBH5//SAGE1//ITFG2//PSMB5//GATAD1//RANGAP1//RBL1//RNF4//RPS14//TRA2B//EFCAB6//SLC3A2//BMPR2//SRPK2//FTO//DCAF17//TAF15//MOB2//FIP1L1//ATRIP//USP42//TRIM24//FANK1//GTF3C5//GTF2IRD1//PUM1//DDX6//RPL13A//NF1//CLN6//G2E3//NOL10//ZCCHC7//SIRT5//CHCHD10//PITRM1//PCCB//ACP6//BCKDHA//CRAT//COL6A2//MIA3//THBS1//CANX//LPXN//GDI2//DYNC1H1//PRSS57//DOCK2//IFI30//PPT1// |
| GO:0070062 | extracellular exosome | Cellular component | 46 | 2167 | 268 | 19559 | 1.54921171714111 | 0.00171807521826984 | 0.0465302987130735 | 2.76495782643933 | 0.171641791044776 | CCDC180//HUWE1//ATP8A1//COL6A2//DDX11//DYNC1H1//DNM2//DOCK2//EFEMP1//FLNB//NEDD4L//GAPDH//TIAM2//GDI2//GNAI1//GNAI2//GNB1//ICAM1//KPNB1//MDH1//MYH9//NID1//PCBP2//GALNT7//PI4KA//PLCG2//SIAE//PPT1//PSMB5//PTPRG//RAB2A//ACTB//PAPPA2//BCR//RPL37A//RPS14//SLC3A2//TFRC//THBS1//UGP2//VDAC3//DDR1//CANX//SPAG9//TMEM59//WDR1// |
| GO:0031981 | nuclear lumen | Cellular component | 97 | 5457 | 268 | 19559 | 1.29726778422347 | 0.00178484123288436 | 0.0465302987130735 | 2.7484004096632 | 0.361940298507463 | NRIP1//NCOR2//TRRAP//TADA2A//TERF2IP//NAT10//CTCF//TCFL5//DDX11//RBPJ//MEIS1//NASP//NFATC2//THAP4//RFX2//ACTB//BACH2//NPAS3//SPI1//NR2C2//KLF11//ENC1//KLF7//ZNF646//SESN1//FBLIM1//HUWE1//RCL1//DDX17//TUBGCP2//PRDM5//FAF1//POLI//CYP24A1//DOCK1//DYRK1A//EYA3//MORC2//HABP4//SCMH1//PDS5B//SMG1//NEDD4L//PIP5K1C//GNAI2//SETD2//GSK3B//BRF1//IFI16//KPNB1//KPNA5//AFF3//MCC//MDM2//PCBP2//CDC40//SIRT6//PLAGL1//RNF216//FAM193B//RNF111//TEX10//ALKBH5//SAGE1//CCAR1//ITFG2//PSMB5//GATAD1//RANGAP1//RBL1//RNF4//RPS14//TRA2B//EFCAB6//SLC3A2//BMPR2//SRPK2//FTO//DCAF17//TAF15//MOB2//FIP1L1//ATRIP//USP42//TRIM24//FANK1//GTF3C5//GTF2IRD1//PUM1//DDX6//RPL13A//NF1//CLN6//G2E3//NOL10//ZCCHC7//LPXN// |
| GO:0000323 | lytic vacuole | Cellular component | 20 | 706 | 268 | 19559 | 2.06746014967655 | 0.00181633772243915 | 0.0465302987130735 | 2.74080339735943 | 0.0746268656716418 | IFI30//GABARAP//LDLR//SIAE//PPT1//ANKRD27//TMEM59//SLC15A4//GNAI1//GNB1//ITFG2//ABCD4//RAB2A//SLC3A2//GPR137B//SPAG9//ATP8A1//DYNC1H1//GDI2//PRSS57// |
| GO:0005764 | lysosome | Cellular component | 20 | 706 | 268 | 19559 | 2.06746014967655 | 0.00181633772243915 | 0.0465302987130735 | 2.74080339735943 | 0.0746268656716418 | SLC15A4//GNAI1//GNB1//ITFG2//ABCD4//RAB2A//SLC3A2//GPR137B//SPAG9//TMEM59//ATP8A1//DYNC1H1//GDI2//PRSS57//LDLR//IFI30//PPT1//GABARAP//SIAE//ANKRD27// |
| GO:0042470 | melanosome | Cellular component | 6 | 105 | 268 | 19559 | 4.17036247334755 | 0.00324995666742599 | 0.0786308960368899 | 2.48812242955135 | 0.0223880597014925 | RAB2A//RPN1//SLC3A2//TFRC//CANX//ANKRD27// |
| GO:0048770 | pigment granule | Cellular component | 6 | 105 | 268 | 19559 | 4.17036247334755 | 0.00324995666742599 | 0.0786308960368899 | 2.48812242955135 | 0.0223880597014925 | RAB2A//RPN1//SLC3A2//TFRC//CANX//ANKRD27// |
| GO:0031090 | organelle membrane | Cellular component | 67 | 3571 | 268 | 19559 | 1.36929431531784 | 0.00344373889221799 | 0.0810674750032938 | 2.46296978461901 | 0.25 | HUWE1//GABARAP//DNM2//ZDHHC17//PITPNB//GALNT1//RAB11FIP5//MIA3//TRAPPC4//GALNT7//GOLPH3L//RAB2A//TMF1//TMEM59//RPN2//TMEM120B//TRA2B//CYP24A1//HK1//AMBRA1//VDAC3//MYO19//COX6B1//CRAT//SIRT5//COQ2//NDUFB2//TTC19//SLC15A4//GNAI1//GNB1//ITFG2//ABCD4//SLC3A2//GPR137B//SPAG9//ATP6V0B//ARL6IP1//MBOAT2//NOMO1//CLN6//ATP13A1//RPN1//ELOVL6//CANX//PLEKHM2//PIP5K1C//CORO1C//LDLR//TFRC//ATP8A1//RPH3AL//AP3S1//ANKRD27//PI4KA//MDM2//GAPDH//KPNB1//RANGAP1//NUP155//STARD13//FAF1//PHLPP2//EVC//CHCHD10//EMC3//NFASC// |
| GO:0016607 | nuclear speck | Cellular component | 13 | 401 | 268 | 19559 | 2.36597870994156 | 0.00372829209517101 | 0.0854563793393145 | 2.4284900699362 | 0.0485074626865672 | DDX17//POLI//DOCK1//DYRK1A//HABP4//IFI16//CDC40//FAM193B//ALKBH5//SRPK2//FTO//NRIP1//LPXN// |
| GO:0042995 | cell projection | Cellular component | 47 | 2321 | 268 | 19559 | 1.477864340512 | 0.00387231771131037 | 0.0864817622192649 | 2.41202901742554 | 0.175373134328358 | DDX6//MYH9//PIP5K1C//TTLL11//EVC//DYNLRB1//FANK1//GABARAP//AMBRA1//DNM2//CORO1C//TIAM2//CAPRIN1//MCC//ACTB//DYNC1H1//DYRK1A//GSK3B//NF1//PPT1//BCR//CANX//NFASC//GNAI2//GNB1//TRAPPC4//RANGAP1//BMPR2//STRN//CFAP44//PARD3//SDCCAG8//RABL2B//PHLPP2//FLNB//AHCYL2//PRR12//ANKRD27//OPHN1//MOB2//CEP83//AP3S1//ZDHHC17//SH3PXD2B//TRIO//LPXN//WDR1// |
| GO:0098805 | whole membrane | Cellular component | 37 | 1721 | 268 | 19559 | 1.569035271059 | 0.00408655091012855 | 0.0868459842550125 | 2.38864308628386 | 0.138059701492537 | GABARAP//RPN2//CYP24A1//RAB11FIP5//HK1//AMBRA1//VDAC3//MYO19//SLC15A4//GNAI1//GNB1//ITFG2//ABCD4//RAB2A//SLC3A2//GPR137B//SPAG9//TMEM59//ATP6V0B//BMPR2//PLEKHM2//PIP5K1C//CORO1C//LDLR//TFRC//ATP8A1//RPH3AL//ZDHHC17//PI4KA//DNM2//MDM2//TUBA1B//GNAI2//ICAM1//CLN6//PPT1//NFASC// |
| GO:0022626 | cytosolic ribosome | Cellular component | 6 | 110 | 268 | 19559 | 3.98080054274084 | 0.00408804288686052 | 0.0868459842550125 | 2.38848455675075 | 0.0223880597014925 | RPL13A//RPL18A//RPL37A//LARP4//RPS27L//RPS14// |
| GO:1903561 | extracellular vesicle | Cellular component | 46 | 2289 | 268 | 19559 | 1.46664123680418 | 0.00493406826636068 | 0.101566340862239 | 2.30679484603018 | 0.171641791044776 | CCDC180//HUWE1//ATP8A1//COL6A2//DDX11//DYNC1H1//DNM2//DOCK2//EFEMP1//FLNB//NEDD4L//GAPDH//TIAM2//GDI2//GNAI1//GNAI2//GNB1//ICAM1//KPNB1//MDH1//MYH9//NID1//PCBP2//GALNT7//PI4KA//PLCG2//SIAE//PPT1//PSMB5//PTPRG//RAB2A//ACTB//PAPPA2//BCR//RPL37A//RPS14//SLC3A2//TFRC//THBS1//UGP2//VDAC3//DDR1//CANX//SPAG9//TMEM59//WDR1// |
| GO:0043230 | extracellular organelle | Cellular component | 46 | 2291 | 268 | 19559 | 1.46536088653198 | 0.00501418215508184 | 0.101566340862239 | 2.29979989305233 | 0.171641791044776 | CCDC180//HUWE1//ATP8A1//COL6A2//DDX11//DYNC1H1//DNM2//DOCK2//EFEMP1//FLNB//NEDD4L//GAPDH//TIAM2//GDI2//GNAI1//GNAI2//GNB1//ICAM1//KPNB1//MDH1//MYH9//NID1//PCBP2//GALNT7//PI4KA//PLCG2//SIAE//PPT1//PSMB5//PTPRG//RAB2A//ACTB//PAPPA2//BCR//RPL37A//RPS14//SLC3A2//TFRC//THBS1//UGP2//VDAC3//DDR1//CANX//SPAG9//TMEM59//WDR1// |
| GO:0005765 | lysosomal membrane | Cellular component | 12 | 375 | 268 | 19559 | 2.33540298507463 | 0.00579407833716359 | 0.112147605148211 | 2.23701563733939 | 0.0447761194029851 | ATP8A1//LDLR//SLC15A4//GNAI1//GNB1//ITFG2//ABCD4//RAB2A//SLC3A2//GPR137B//SPAG9//TMEM59// |
| GO:0098852 | lytic vacuole membrane | Cellular component | 12 | 375 | 268 | 19559 | 2.33540298507463 | 0.00579407833716359 | 0.112147605148211 | 2.23701563733939 | 0.0447761194029851 | SLC15A4//GNAI1//GNB1//ITFG2//ABCD4//RAB2A//SLC3A2//GPR137B//SPAG9//TMEM59//ATP8A1//LDLR// |
| GO:0002102 | podosome | Cellular component | 3 | 29 | 268 | 19559 | 7.54979413278435 | 0.00714380808197543 | 0.134145165847526 | 2.14607022126097 | 0.0111940298507463 | SH3PXD2B//LPXN//WDR1// |
| GO:0000932 | P-body | Cellular component | 5 | 88 | 268 | 19559 | 4.14666723202171 | 0.00723860251990093 | 0.134145165847526 | 2.14034527033288 | 0.0186567164179104 | DDX6//LSM14A//CAPRIN1//CNOT8//PUM1// |
| GO:0036477 | somatodendritic compartment | Cellular component | 21 | 859 | 268 | 19559 | 1.78417719319584 | 0.00767317165282287 | 0.139150772097706 | 2.11502508629817 | 0.0783582089552239 | DYRK1A//NFASC//GNAI2//GNB1//GSK3B//CAPRIN1//NF1//TRAPPC4//PPT1//RANGAP1//BMPR2//STRN//CANX//FLNB//PARD3//ENC1//DNM2//OPHN1//BCR//TIAM2//LDLR// |
| GO:0098843 | postsynaptic endocytic zone | Cellular component | 2 | 10 | 268 | 19559 | 14.5962686567164 | 0.00782822942914764 | 0.139150772097706 | 2.10633645455815 | 0.00746268656716418 | DNM2//RNF216// |
| GO:0098588 | bounding membrane of organelle | Cellular component | 42 | 2121 | 268 | 19559 | 1.44517511452638 | 0.00927143822520846 | 0.161508453883131 | 2.03285289100945 | 0.156716417910448 | HUWE1//GABARAP//DNM2//ZDHHC17//PITPNB//GALNT1//RAB11FIP5//MIA3//TRAPPC4//GALNT7//GOLPH3L//RAB2A//TMF1//TMEM59//RPN2//CYP24A1//HK1//AMBRA1//VDAC3//MYO19//SLC15A4//GNAI1//GNB1//ITFG2//ABCD4//SLC3A2//GPR137B//SPAG9//ATP6V0B//ARL6IP1//PLEKHM2//PIP5K1C//CORO1C//LDLR//TFRC//ATP8A1//RPH3AL//PI4KA//MDM2//PHLPP2//EVC//NFASC// |
| GO:0005938 | cell cortex | Cellular component | 10 | 305 | 268 | 19559 | 2.39283092733056 | 0.00965296113340931 | 0.164857434258814 | 2.01533944244553 | 0.0373134328358209 | MYH9//ACTB//WDR1//GNAI1//DYNC1H1//FLNB//CORO1C//FRYL//MYO9B//PARD3// |
| GO:0034451 | centriolar satellite | Cellular component | 5 | 95 | 268 | 19559 | 3.84112333071485 | 0.00991495335238397 | 0.166075468652432 | 2.00370932471132 | 0.0186567164179104 | SDCCAG8//RAB11FIP5//LATS2//HOOK3//SPAG9// |
| GO:0030425 | dendrite | Cellular component | 16 | 613 | 268 | 19559 | 1.90489639891895 | 0.010713287391932 | 0.172306502566745 | 1.9700772446576 | 0.0597014925373134 | CANX//DNM2//OPHN1//BCR//STRN//DYRK1A//NFASC//GNAI2//GNB1//GSK3B//CAPRIN1//NF1//TRAPPC4//PPT1//RANGAP1//BMPR2// |
| GO:0097447 | dendritic tree | Cellular component | 16 | 615 | 268 | 19559 | 1.89870161388181 | 0.0110247099523686 | 0.172306502566745 | 1.95763282750573 | 0.0597014925373134 | DYRK1A//NFASC//GNAI2//GNB1//GSK3B//CAPRIN1//NF1//TRAPPC4//PPT1//RANGAP1//BMPR2//STRN//CANX//DNM2//OPHN1//BCR// |
| GO:0005834 | heterotrimeric G-protein complex | Cellular component | 3 | 34 | 268 | 19559 | 6.43953028972783 | 0.0111272314140186 | 0.172306502566745 | 1.95361287979695 | 0.0111940298507463 | GNAI1//GNAI2//GNB1// |
| GO:1905360 | GTPase complex | Cellular component | 3 | 34 | 268 | 19559 | 6.43953028972783 | 0.0111272314140186 | 0.172306502566745 | 1.95361287979695 | 0.0111940298507463 | GNAI1//GNAI2//GNB1// |
| GO:0031932 | TORC2 complex | Cellular component | 2 | 12 | 268 | 19559 | 12.1635572139303 | 0.0112760857018421 | 0.172306502566745 | 1.94784163201153 | 0.00746268656716418 | SMG1//SESN1// |
| GO:0045202 | synapse | Cellular component | 29 | 1357 | 268 | 19559 | 1.55966024703307 | 0.011634980159765 | 0.174725305502678 | 1.93423435294553 | 0.108208955223881 | TRAPPC4//PPT1//DNM2//PRR12//BCR//RPL18A//RPS14//BMPR2//STRN//ATP8A1//MYH9//ZDHHC17//OPHN1//CANX//ACTB//RNF216//AKAP7//PIP5K1C//NF1//GSK3B//GABARAP//CORO1C//TIAM2//GDI2//GNAI2//GNB1//CAPRIN1//MDM2//VDAC3// |
| GO:0030496 | midbody | Cellular component | 7 | 182 | 268 | 19559 | 2.80697474167623 | 0.0129206569745117 | 0.185534170690902 | 1.88871540327969 | 0.0261194029850746 | CCDC124//DDX11//DNM2//GNAI1//GNAI2//TTC19//NAT10// |
| GO:0001931 | uropod | Cellular component | 2 | 13 | 268 | 19559 | 11.2278989667049 | 0.0132067951582502 | 0.185534170690902 | 1.87920255815765 | 0.00746268656716418 | PIP5K1C//MYH9// |
| GO:0008250 | oligosaccharyltransferase complex | Cellular component | 2 | 13 | 268 | 19559 | 11.2278989667049 | 0.0132067951582502 | 0.185534170690902 | 1.87920255815765 | 0.00746268656716418 | RPN1//RPN2// |
| GO:0031254 | cell trailing edge | Cellular component | 2 | 13 | 268 | 19559 | 11.2278989667049 | 0.0132067951582502 | 0.185534170690902 | 1.87920255815765 | 0.00746268656716418 | PIP5K1C//MYH9// |
| GO:1990904 | ribonucleoprotein complex | Cellular component | 18 | 744 | 268 | 19559 | 1.76567766008666 | 0.0144430323115423 | 0.199680653069101 | 1.84034161715991 | 0.0671641791044776 | TRA2B//NAT10//LARP4//RPL13A//DDX6//RPL18A//RPL37A//RPS27L//RPS14//RPPH1//NOL10//CDC40//LSM14A//DDX17//DYRK1A//GAPDH//NFATC2//ACTB// |
| GO:0038201 | TOR complex | Cellular component | 2 | 15 | 268 | 19559 | 9.73084577114428 | 0.0174615345652367 | 0.237248319370281 | 1.75791759202056 | 0.00746268656716418 | SMG1//SESN1// |
| GO:0099512 | supramolecular fiber | Cellular component | 22 | 989 | 268 | 19559 | 1.62344747445784 | 0.0177050984604687 | 0.237248319370281 | 1.75190165372827 | 0.082089552238806 | DDX6//KLHL21//TUBA1B//GABARAP//TTLL11//DYNC1H1//DNM2//CSPP1//DYNLRB1//HOOK3//TUBGCP2//IFFO1//KRT17//MYO9B//ACTB//RCSD1//PDE4DIP//HABP4//CORO1C//FLNB//MYH9//RNF4// |
| GO:0120025 | plasma membrane bounded cell projection | Cellular component | 42 | 2221 | 268 | 19559 | 1.38010644660533 | 0.0191640752700591 | 0.249785905556344 | 1.7175121320475 | 0.156716417910448 | DDX6//MYH9//PIP5K1C//TTLL11//EVC//DYNLRB1//FANK1//GABARAP//AMBRA1//DNM2//CORO1C//TIAM2//CAPRIN1//MCC//ACTB//DYNC1H1//DYRK1A//GSK3B//NF1//PPT1//BCR//CANX//NFASC//GNAI2//GNB1//TRAPPC4//RANGAP1//BMPR2//STRN//CFAP44//PARD3//SDCCAG8//RABL2B//PHLPP2//FLNB//AHCYL2//PRR12//ANKRD27//OPHN1//MOB2//CEP83//AP3S1// |
| GO:0099081 | supramolecular polymer | Cellular component | 22 | 997 | 268 | 19559 | 1.61042081468285 | 0.0192143004274111 | 0.249785905556344 | 1.71637542311979 | 0.082089552238806 | DDX6//KLHL21//TUBA1B//GABARAP//TTLL11//DYNC1H1//DNM2//CSPP1//DYNLRB1//HOOK3//TUBGCP2//IFFO1//KRT17//MYO9B//ACTB//RCSD1//PDE4DIP//HABP4//CORO1C//FLNB//MYH9//RNF4// |
| GO:0030014 | CCR4-NOT complex | Cellular component | 2 | 16 | 268 | 19559 | 9.12266791044776 | 0.0197777205127899 | 0.253329331862353 | 1.7038237645975 | 0.00746268656716418 | CNOT8//CNOT11// |
| GO:0043005 | neuron projection | Cellular component | 28 | 1367 | 268 | 19559 | 1.49486292021968 | 0.0216847777920025 | 0.272521074771425 | 1.66384502378968 | 0.104477611940299 | DNM2//DYRK1A//GSK3B//NF1//PPT1//ACTB//BCR//CANX//NFASC//GNAI2//GNB1//CAPRIN1//TRAPPC4//RANGAP1//BMPR2//STRN//TIAM2//PARD3//PHLPP2//OPHN1//MOB2//SDCCAG8//AP3S1//DYNC1H1//FLNB//AHCYL2//PRR12//ANKRD27// |
| GO:0042622 | photoreceptor outer segment membrane | Cellular component | 2 | 17 | 268 | 19559 | 8.58604038630378 | 0.0222146915140886 | 0.272521074771425 | 1.65335971324279 | 0.00746268656716418 | PHLPP2//GNB1// |
| GO:0099092 | postsynaptic density, intracellular component | Cellular component | 2 | 17 | 268 | 19559 | 8.58604038630378 | 0.0222146915140886 | 0.272521074771425 | 1.65335971324279 | 0.00746268656716418 | DNM2//BCR// |
| GO:0015629 | actin cytoskeleton | Cellular component | 13 | 506 | 268 | 19559 | 1.87501474839243 | 0.0228052142955352 | 0.275879745158488 | 1.64196584243505 | 0.0485074626865672 | FLNB//MYH9//FBLIM1//SH3PXD2B//LPXN//WDR1//MYO9B//ACTB//RCSD1//MYO19//GABARAP//CORO1C//OPHN1// |
| GO:0048471 | perinuclear region of cytoplasm | Cellular component | 17 | 728 | 268 | 19559 | 1.70423466458914 | 0.0232853492224674 | 0.277829303736563 | 1.63291724434266 | 0.0634328358208955 | RANGAP1//FAF1//GABARAP//SLC30A7//DDX6//DNM2//GALNT1//GAPDH//MYO9B//PDE4D//AMBRA1//CCAR1//TRA2B//TFRC//MOB2//SPAG9//LPXN// |
| GO:0022627 | cytosolic small ribosomal subunit | Cellular component | 3 | 47 | 268 | 19559 | 4.65838361384566 | 0.0264902684420059 | 0.311797619094421 | 1.57691364071027 | 0.0111940298507463 | LARP4//RPS27L//RPS14// |
| GO:0005912 | adherens junction | Cellular component | 6 | 166 | 268 | 19559 | 2.63787987771983 | 0.0270586800652321 | 0.314241471157562 | 1.56769339230482 | 0.0223880597014925 | PIP5K1C//MYH9//PARD3//ACTB//BMPR2//CD99L2// |
| GO:0005791 | rough endoplasmic reticulum | Cellular component | 4 | 84 | 268 | 19559 | 3.47530206112296 | 0.0283351699797049 | 0.322266449269928 | 1.54767417772775 | 0.0149253731343284 | ARL6IP1//RPN1//RPN2//CANX// |
| GO:0031967 | organelle envelope | Cellular component | 25 | 1219 | 268 | 19559 | 1.49674617070513 | 0.0288596820241727 | 0.322266449269928 | 1.53970845827052 | 0.0932835820895522 | FAF1//KPNB1//TERF2IP//RANGAP1//NUP155//TMEM120B//TRA2B//CCAR1//RANBP17//CYP24A1//RAB11FIP5//HK1//AMBRA1//VDAC3//MYO19//COX6B1//CRAT//SIRT5//COQ2//NDUFB2//TTC19//CHCHD10//GAPDH//DNM2//STARD13// |
| GO:0031975 | envelope | Cellular component | 25 | 1219 | 268 | 19559 | 1.49674617070513 | 0.0288596820241727 | 0.322266449269928 | 1.53970845827052 | 0.0932835820895522 | FAF1//KPNB1//TERF2IP//RANGAP1//NUP155//TMEM120B//TRA2B//CCAR1//RANBP17//CYP24A1//RAB11FIP5//HK1//AMBRA1//VDAC3//MYO19//COX6B1//CRAT//SIRT5//COQ2//NDUFB2//TTC19//CHCHD10//GAPDH//DNM2//STARD13// |
| GO:0005643 | nuclear pore | Cellular component | 4 | 85 | 268 | 19559 | 3.43441615452151 | 0.029425111774264 | 0.323906479120753 | 1.53128187876987 | 0.0149253731343284 | NUP155//RANGAP1//KPNB1//RANBP17// |
| GO:0005794 | Golgi apparatus | Cellular component | 31 | 1599 | 268 | 19559 | 1.4148978372677 | 0.0314842258921226 | 0.323906479120753 | 1.50190698030102 | 0.115671641791045 | TMEM59//HUWE1//GABARAP//DNM2//ZDHHC17//PITPNB//GALNT1//RAB11FIP5//MIA3//TRAPPC4//GALNT7//GOLPH3L//RAB2A//TMF1//HOOK3//ATP8A1//AP3S1//SLC30A7//HABP4//NEDD4L//GDI2//LDLR//CEP83//PLAGL1//ALKBH5//PPT1//G2E3//TRRAP//ADAM19//PKDCC//PDE4DIP// |
| GO:0120111 | neuron projection cytoplasm | Cellular component | 4 | 87 | 268 | 19559 | 3.35546405901527 | 0.0316787418680254 | 0.323906479120753 | 1.49923207489265 | 0.0149253731343284 | CANX//AP3S1//DYNC1H1//RANGAP1// |
| GO:0099513 | polymeric cytoskeletal fiber | Cellular component | 17 | 757 | 268 | 19559 | 1.63894694296023 | 0.0321459757738156 | 0.323906479120753 | 1.49287338692685 | 0.0634328358208955 | DDX6//KLHL21//TUBA1B//GABARAP//TTLL11//DYNC1H1//DNM2//CSPP1//DYNLRB1//HOOK3//TUBGCP2//IFFO1//KRT17//MYO9B//ACTB//RCSD1//RNF4// |
| GO:0030424 | axon | Cellular component | 15 | 643 | 268 | 19559 | 1.70251967224531 | 0.03216815635551 | 0.323906479120753 | 1.49257382898217 | 0.0559701492537313 | DNM2//TIAM2//NFASC//PARD3//OPHN1//ACTB//AP3S1//DYNC1H1//RANGAP1//DYRK1A//GSK3B//NF1//PPT1//BCR//CANX// |
| GO:0009925 | basal plasma membrane | Cellular component | 3 | 51 | 268 | 19559 | 4.29302019315189 | 0.0327010674961766 | 0.323906479120753 | 1.48543806996565 | 0.0111940298507463 | SLC3A2//BMPR2//SLC23A2// |
| GO:0000242 | pericentriolar material | Cellular component | 2 | 21 | 268 | 19559 | 6.95060412224591 | 0.0330972177287566 | 0.323906479120753 | 1.48020851304709 | 0.00746268656716418 | RABL2B//HOOK3// |
| GO:0005868 | cytoplasmic dynein complex | Cellular component | 2 | 21 | 268 | 19559 | 6.95060412224591 | 0.0330972177287566 | 0.323906479120753 | 1.48020851304709 | 0.00746268656716418 | DYNC1H1//DYNLRB1// |
| GO:0035267 | NuA4 histone acetyltransferase complex | Cellular component | 2 | 21 | 268 | 19559 | 6.95060412224591 | 0.0330972177287566 | 0.323906479120753 | 1.48020851304709 | 0.00746268656716418 | ACTB//TRRAP// |
| GO:0043189 | H4/H2A histone acetyltransferase complex | Cellular component | 2 | 21 | 268 | 19559 | 6.95060412224591 | 0.0330972177287566 | 0.323906479120753 | 1.48020851304709 | 0.00746268656716418 | ACTB//TRRAP// |
| GO:0099091 | postsynaptic specialization, intracellular component | Cellular component | 2 | 21 | 268 | 19559 | 6.95060412224591 | 0.0330972177287566 | 0.323906479120753 | 1.48020851304709 | 0.00746268656716418 | DNM2//BCR// |
| GO:0005874 | microtubule | Cellular component | 11 | 431 | 268 | 19559 | 1.86263289122831 | 0.0360274451876421 | 0.34866560842707 | 1.44336653388767 | 0.041044776119403 | KLHL21//TUBA1B//TUBGCP2//DYNC1H1//RNF4//GABARAP//TTLL11//DNM2//CSPP1//DYNLRB1//HOOK3// |
| GO:0045121 | membrane raft | Cellular component | 9 | 329 | 268 | 19559 | 1.99645012021957 | 0.0381551605833311 | 0.365199394154741 | 1.41844671429384 | 0.0335820895522388 | BMPR2//CORO1C//TUBA1B//GNAI1//GNAI2//HK1//ICAM1//CLN6//PPT1// |
| GO:0098857 | membrane microdomain | Cellular component | 9 | 330 | 268 | 19559 | 1.99040027137042 | 0.0387760323071944 | 0.367107871082243 | 1.41143663144214 | 0.0335820895522388 | BMPR2//CORO1C//TUBA1B//GNAI1//GNAI2//HK1//ICAM1//CLN6//PPT1// |
| GO:0031982 | vesicle | Cellular component | 68 | 4073 | 268 | 19559 | 1.21844619280225 | 0.0406102273928953 | 0.380338796335611 | 1.39136457871476 | 0.253731343283582 | SPAG9//DNM2//TFRC//RAB11FIP5//LDLR//CLN6//WDFY1//ANKRD27//TMEM59//NEDD4L//TRAPPC4//PPT1//PLEKHM2//PIP5K1C//CORO1C//ATP6V0B//AP3S1//THBS1//ATP8A1//RPH3AL//ZDHHC17//PI4KA//MDM2//GABARAP//SLC30A7//HUWE1//GDI2//DYNC1H1//PRSS57//SLC15A4//DOCK2//KPNB1//RAB2A//RPN1//SLC3A2//CANX//FLNB//AMBRA1//CCDC180//COL6A2//DDX11//EFEMP1//GAPDH//TIAM2//GNAI1//GNAI2//GNB1//ICAM1//MDH1//MYH9//NID1//PCBP2//GALNT7//PLCG2//SIAE//PSMB5//PTPRG//ACTB//PAPPA2//BCR//RPL37A//RPS14//UGP2//VDAC3//DDR1//WDR1//NFASC//MYO19// |
| GO:0016459 | myosin complex | Cellular component | 3 | 57 | 268 | 19559 | 3.84112333071485 | 0.043307131215642 | 0.39705801356657 | 1.36344058418707 | 0.0111940298507463 | MYH9//MYO9B//MYO19// |
| GO:1904115 | axon cytoplasm | Cellular component | 3 | 57 | 268 | 19559 | 3.84112333071485 | 0.043307131215642 | 0.39705801356657 | 1.36344058418707 | 0.0111940298507463 | AP3S1//DYNC1H1//RANGAP1// |
| GO:0045335 | phagocytic vesicle | Cellular component | 5 | 140 | 268 | 19559 | 2.60647654584222 | 0.0437806082400799 | 0.397217810178225 | 1.35871820911173 | 0.0186567164179104 | DNM2//ATP6V0B//FLNB//RAB11FIP5//AMBRA1// |
| GO:0044391 | ribosomal subunit | Cellular component | 6 | 187 | 268 | 19559 | 2.34164737808285 | 0.0442995208223098 | 0.397782295218885 | 1.35360097141468 | 0.0223880597014925 | RPL13A//RPL18A//RPL37A//LARP4//RPS27L//RPS14// |
| GO:0022625 | cytosolic large ribosomal subunit | Cellular component | 3 | 58 | 268 | 19559 | 3.77489706639218 | 0.0452224566019843 | 0.401926119391105 | 1.34464584933901 | 0.0111940298507463 | RPL13A//RPL18A//RPL37A// |
| GO:0031234 | extrinsic component of cytoplasmic side of plasma membrane | Cellular component | 4 | 99 | 268 | 19559 | 2.94874114277099 | 0.047283144221203 | 0.41355733356036 | 1.32529365157444 | 0.0149253731343284 | GNAI1//GNAI2//GNB1//STAC// |
| GO:0098589 | membrane region | Cellular component | 9 | 343 | 268 | 19559 | 1.91496236021061 | 0.0474807501217405 | 0.41355733356036 | 1.32348242848831 | 0.0335820895522388 | BMPR2//CORO1C//TUBA1B//GNAI1//GNAI2//HK1//ICAM1//CLN6//PPT1// |
| GO:0005741 | mitochondrial outer membrane | Cellular component | 6 | 192 | 268 | 19559 | 2.28066697761194 | 0.0492247816413764 | 0.424502819897414 | 1.30781620169377 | 0.0223880597014925 | CYP24A1//RAB11FIP5//HK1//AMBRA1//VDAC3//MYO19// |
