# Supplementary material for: Differentially Expressed Circular RNAs and Their Therapeutic Mechanism in Non-segmental Vitiligo Patients Treated With Methylprednisolone
Source: Front Med (Lausanne). 2022 May 16;9:839066. doi: 10.3389/fmed.2022.839066 (PMC9149005; doi:10.3389/fmed.2022.839066)
Supplement: Supplementary file 1 [file Data_Sheet_1.ZIP › Additional files/GO Analysis Report/GO_GC_vs_control_down/MF_result(Human).html]

| GO.ID | Term | Ontology | Count | Pop.Hits | List.Total | Pop.Total | Fold.Enrichment | Pvalue | FDR | Enrichment.Score | Gene.Ratio | GENES |
| --- | --- | --- | --- | --- | --- | --- | --- | --- | --- | --- | --- | --- |
| GO:1901363 | heterocyclic compound binding | Molecular function | 131 | 6213 | 268 | 18352 | 1.44383826882007 | 2.27220481202019e-07 | 0.00018543648806086 | 6.64355252475625 | 0.488805970149254 | NAT10//NEMF//VDAC3//AKAP7//CTCF//PRDM5//KLF11//PLAGL1//RBL1//NPAS3//NR2C2//NCOR2//TCFL5//ZNF483//ZNF652//IFI16//RBPJ//MEIS1//NFATC2//CCAR1//RFX2//ACTB//BACH2//SPI1//NRIP1//KLF7//GTF2IRD1//ZNF646//BRF1//ZCCHC2//HUWE1//DDX11//PDS5B//LCORL//THAP4//CRLF3//TERF2IP//GPBP1L1//RNF4//TADA2A//TMF1//ZNF236//TAF15//ZMYM4//GTF3C5//POLI//LSM14A//AFF3//PCBP2//DDX17//IGF2BP3//LARP4//CCDC124//DDX6//DYNC1H1//HABP4//SMG1//GPATCH8//FLNB//FAM120A//R3HDM1//RPL13A//MTO1//GDI2//KPNB1//CAPRIN1//MYH9//RPS27L//CDC40//ALKBH5//RANGAP1//RPL18A//RPL37A//RPN1//RPS14//ARHGEF28//TRA2B//SLC3A2//SRPK2//TFRC//NOL10//FIP1L1//CANX//ZCCHC7//CNOT8//PUM1//TUBA1B//ATP8A1//CLK1//TTLL11//DYRK1A//MORC2//PIP5K1C//LATS2//GSK3B//HK1//MYO9B//PCCB//TAOK3//PHKG1//PI4KA//ATP13A1//STARD9//ABCD4//BCR//BMPR2//TRIO//DDR1//MYO19//PKDCC//NEK9//RABL2B//AGAP1//DNM2//GNAI1//GNAI2//RAB2A//RANBP17//MDM2//CYP24A1//HBG2//PDE4D//MTR//UGP2//TRIM24//GATAD1//OVOL2//GAPDH//MDH1//SIRT5//SIRT6// |
| GO:0097159 | organic cyclic compound binding | Molecular function | 132 | 6299 | 268 | 18352 | 1.4349967893506 | 2.8463006609495e-07 | 0.00018543648806086 | 6.54571922635484 | 0.492537313432836 | NAT10//NEMF//VDAC3//AKAP7//CTCF//PRDM5//KLF11//PLAGL1//RBL1//NPAS3//NR2C2//NCOR2//TCFL5//ZNF483//ZNF652//IFI16//RBPJ//MEIS1//NFATC2//CCAR1//RFX2//ACTB//BACH2//SPI1//NRIP1//KLF7//GTF2IRD1//ZNF646//BRF1//ZCCHC2//HUWE1//DDX11//PDS5B//LCORL//THAP4//CRLF3//TERF2IP//GPBP1L1//RNF4//TADA2A//TMF1//ZNF236//TAF15//ZMYM4//GTF3C5//POLI//LSM14A//AFF3//PCBP2//DDX17//IGF2BP3//LARP4//CCDC124//DDX6//DYNC1H1//HABP4//SMG1//GPATCH8//FLNB//FAM120A//R3HDM1//RPL13A//MTO1//GDI2//KPNB1//CAPRIN1//MYH9//RPS27L//CDC40//ALKBH5//RANGAP1//RPL18A//RPL37A//RPN1//RPS14//ARHGEF28//TRA2B//SLC3A2//SRPK2//TFRC//NOL10//FIP1L1//CANX//ZCCHC7//CNOT8//PUM1//TUBA1B//ATP8A1//CLK1//TTLL11//DYRK1A//MORC2//PIP5K1C//LATS2//GSK3B//HK1//MYO9B//PCCB//TAOK3//PHKG1//PI4KA//ATP13A1//STARD9//ABCD4//BCR//BMPR2//TRIO//DDR1//MYO19//PKDCC//NEK9//RABL2B//AGAP1//DNM2//GNAI1//GNAI2//RAB2A//RANBP17//MDM2//OSBPL10//CYP24A1//HBG2//PDE4D//MTR//UGP2//TRIM24//GATAD1//OVOL2//GAPDH//MDH1//SIRT5//SIRT6// |
| GO:0005096 | GTPase activator activity | Molecular function | 14 | 275 | 268 | 18352 | 3.48613297150611 | 5.58399814790584e-05 | 0.0209756794428301 | 4.25305473443117 | 0.0522388059701493 | AGAP1//DOCK1//DOCK2//TBC1D1//TIAM2//GDI2//MYO9B//NF1//OPHN1//RALGAPA2//RANGAP1//BCR//ANKRD27//STARD13// |
| GO:0043167 | ion binding | Molecular function | 122 | 6281 | 268 | 18352 | 1.33008575970648 | 7.54326516488775e-05 | 0.0209756794428301 | 4.12244062546403 | 0.455223880597015 | ATP8A1//MORC2//GNAI1//SRPK2//OSBPL10//THBS1//CYP24A1//EFEMP1//LDLR//NID1//SUSD1//EFCAB6//SLIT3//CANX//DDX17//CLK1//TTLL11//DDX6//DDX11//DYNC1H1//DYRK1A//SMG1//PIP5K1C//LATS2//GSK3B//HK1//MYH9//MYO9B//PCCB//TAOK3//PHKG1//PI4KA//NAT10//ATP13A1//STARD9//ABCD4//ACTB//BCR//BMPR2//TRIO//DDR1//MYO19//PKDCC//NEK9//TUBA1B//RABL2B//AGAP1//DNM2//GNAI2//RAB2A//RANBP17//OPHN1//WDFY1//PARD3//FTO//PRSS57//EVA1C//PITRM1//CTCF//DCTD//SIRT5//KPNB1//MDM2//MTR//SIRT6//ZCCHC2//GATAD1//RNF4//PAPPA2//NR2C2//ZCCHC7//KLF7//TRIM24//ZMYM4//NF1//SH3PXD2B//GALNT1//PDE4D//PITPNB//CLN6//PPT1//PRDM5//POLI//SMYD4//ZNF483//EYA3//LNX2//ZNF652//PHLPP2//GPATCH8//SETD2//BRF1//HBG2//RPS27L//THAP4//PDE3A//GALNT7//PLAGL1//RNF216//FBLIM1//RNF111//ALKBH5//G2E3//PHRF1//OVOL2//BCKDHA//RPL37A//ARHGEF28//STAC//UGP2//ZNF236//TAF15//MOB2//KLF11//ADAM19//CNOT8//LPXN//RPH3AL//ZNF646//MTO1//GOLPH3L//SESN1// |
| GO:0005515 | protein binding | Molecular function | 226 | 13697 | 268 | 18352 | 1.12987809728462 | 8.04899441397933e-05 | 0.0209756794428301 | 4.09425837400966 | 0.843283582089552 | ANKRD27//GSK3B//SPI1//RBL1//RBPJ//GNAI1//GNAI2//PLCG2//THBS1//LDLR//MDM2//TRIM24//FLNB//MYH9//MYO9B//OPHN1//MYO19//ENC1//SPAG9//DOCK1//TIAM2//BCR//ARHGEF28//TRIO//NCOR2//EFEMP1//ICAM1//PHKG1//STRN//GABARAP//DNM2//GAPDH//CCSER2//STARD9//HOOK3//RPH3AL//BRF1//IFI16//NFATC2//RNF4//KPNB1//RANGAP1//RANBP17//TTLL11//DENND5B//ADAM19//TBC1D1//RAB11FIP5//GDI2//BMPR2//PLEKHM2//ACTB//PCBP2//PDE4D//PDE4DIP//FAF1//TFRC//NEK9//AKAP7//TERF2IP//PARD3//DDX6//NFASC//PHRF1//TRA2B//LNX2//NRIP1//GNB1//DOCK2//FBLIM1//TUBA1B//AMBRA1//HBG2//HABP4//RNF111//CANX//GCLM//SH3PXD2B//SCMH1//NASP//DCTD//DYRK1A//ARL6IP1//ZDHHC17//HK1//THAP4//CRLF3//PTPRG//UGP2//MORC2//CLN6//TBCEL//SETD2//TUBGCP2//NID1//NEDD4L//STAC//PI4KA//SLC3A2//DYNC1H1//DYNLRB1//RALGAPA2//NPAS3//TCFL5//CORO1C//SLIT3//TMF1//RCSD1//WDR1//NAT10//PRDM5//ATRIP//SRPK2//KLHL21//HUWE1//RCL1//CDR2//ATP8A1//IFI30//DDX17//IGF2BP3//CTCF//SDCCAG8//RABL2B//POLI//LARP4//AGAP1//AP3S1//CLK1//SLC15A4//COL6A2//TMEM120B//DDX11//EYA3//ZNF652//PHLPP2//PDS5B//SMG1//GPATCH8//AHCYL2//PIP5K1C//NOMO1//LCORL//NALCN//LSM14A//WSB1//LATS2//SESN1//FRYL//MIA3//KPNA5//KRT17//CHCHD10//CAPRIN1//MCC//MDH1//MEIS1//MTR//NF1//PCCB//RPS27L//CEP83//TAOK3//CDC40//PDE3A//TRAPPC4//SIRT6//PLAGL1//ATP6V0B//RNF216//FAM193B//TEX10//TTC19//GOLPH3L//PPT1//CNOT11//G2E3//CCAR1//CFAP44//EMC3//PSMB5//FAM219B//HECW2//WDFY1//ABCD4//OVOL2//RAB2A//UBL5//BCKDHA//RFX2//GPBP1L1//BACH2//RPL18A//RPL37A//RPN1//RPN2//RPS14//LMBR1//TADA2A//GPR137B//NR2C2//UMAD1//ZNF236//DDR1//ELOVL6//DCAF17//TAF15//MOB2//FIP1L1//TRRAP//CD99L2//USP42//ZCCHC7//FAM120B//KLF11//STARD13//ZMYM4//FANK1//GTF3C5//CNOT8//LPXN//TMEM59//GTF2IRD1//NUP155//PUM1//ZNF646// |
| GO:0017016 | Ras GTPase binding | Molecular function | 17 | 415 | 268 | 18352 | 2.80510699514476 | 0.000136504325536202 | 0.0256394693825188 | 3.86485358652257 | 0.0634328358208955 | DOCK1//BCR//ARHGEF28//TRIO//KPNB1//RANGAP1//RANBP17//MYO9B//DENND5B//TBC1D1//RAB11FIP5//GDI2//ANKRD27//RPH3AL//DOCK2//TIAM2//CORO1C// |
| GO:0003676 | nucleic acid binding | Molecular function | 89 | 4303 | 268 | 18352 | 1.41633917329458 | 0.000162295955286921 | 0.0256394693825188 | 3.78969230345448 | 0.332089552238806 | NAT10//NEMF//CTCF//PRDM5//KLF11//PLAGL1//RBL1//NPAS3//NR2C2//NCOR2//TCFL5//ZNF483//ZNF652//IFI16//RBPJ//MEIS1//NFATC2//CCAR1//RFX2//ACTB//BACH2//SPI1//NRIP1//KLF7//GTF2IRD1//ZNF646//BRF1//HUWE1//DDX11//PDS5B//LCORL//THAP4//CRLF3//TERF2IP//GPBP1L1//RNF4//TADA2A//TMF1//ZNF236//TAF15//ZMYM4//GTF3C5//POLI//LSM14A//AFF3//PCBP2//DDX17//IGF2BP3//LARP4//CCDC124//DDX6//DYNC1H1//HABP4//SMG1//GPATCH8//FLNB//FAM120A//R3HDM1//RPL13A//MTO1//GDI2//KPNB1//CAPRIN1//MYH9//RPS27L//CDC40//ALKBH5//RANGAP1//RPL18A//RPL37A//RPN1//RPS14//ARHGEF28//TRA2B//SLC3A2//SRPK2//TFRC//NOL10//FIP1L1//CANX//ZCCHC7//CNOT8//PUM1//TUBA1B//MDM2//TRIM24//GATAD1//OVOL2//ZCCHC2// |
| GO:0003723 | RNA binding | Molecular function | 49 | 1988 | 268 | 18352 | 1.68782846331722 | 0.000168820781038453 | 0.0256394693825188 | 3.77257409482628 | 0.182835820895522 | NAT10//NEMF//TUBA1B//LSM14A//SLC3A2//TFRC//DDX11//IGF2BP3//DDX6//RPL13A//PCBP2//TRA2B//PUM1//TAF15//MDM2//LARP4//RPS14//HUWE1//DDX17//CCDC124//DYNC1H1//HABP4//SMG1//GPATCH8//FLNB//FAM120A//R3HDM1//MTO1//GDI2//IFI16//KPNB1//CAPRIN1//MYH9//RPS27L//CDC40//ALKBH5//CCAR1//RANGAP1//RPL18A//RPL37A//RPN1//ARHGEF28//SPI1//SRPK2//NOL10//FIP1L1//CANX//ZCCHC7//CNOT8// |
| GO:0030695 | GTPase regulator activity | Molecular function | 14 | 307 | 268 | 18352 | 3.12275754776606 | 0.000178665394195654 | 0.0256394693825188 | 3.74795955810025 | 0.0522388059701493 | GDI2//AGAP1//DOCK1//DOCK2//TBC1D1//TIAM2//MYO9B//NF1//OPHN1//RALGAPA2//RANGAP1//BCR//ANKRD27//STARD13// |
| GO:0031267 | small GTPase binding | Molecular function | 17 | 428 | 268 | 18352 | 2.71990514716139 | 0.000196772596949492 | 0.0256394693825188 | 3.70603538264383 | 0.0634328358208955 | DOCK1//BCR//ARHGEF28//TRIO//KPNB1//RANGAP1//RANBP17//MYO9B//DENND5B//TBC1D1//RAB11FIP5//GDI2//ANKRD27//RPH3AL//DOCK2//TIAM2//CORO1C// |
| GO:0005089 | Rho guanyl-nucleotide exchange factor activity | Molecular function | 6 | 60 | 268 | 18352 | 6.84776119402985 | 0.000237126479609223 | 0.0280887093573471 | 3.62501994620717 | 0.0223880597014925 | DOCK2//TIAM2//DOCK1//BCR//ARHGEF28//TRIO// |
| GO:0005488 | binding | Molecular function | 256 | 16428 | 268 | 18352 | 1.06709694769396 | 0.000284922917368451 | 0.0309378801109243 | 3.54527261749842 | 0.955223880597015 | NAT10//NEMF//ANKRD27//VDAC3//AKAP7//ATP8A1//MORC2//GNAI1//SRPK2//CTCF//PRDM5//KLF11//PLAGL1//RBL1//NPAS3//NR2C2//NCOR2//TCFL5//ZNF483//ZNF652//IFI16//RBPJ//MEIS1//NFATC2//CCAR1//RFX2//ACTB//BACH2//SPI1//NRIP1//KLF7//GTF2IRD1//ZNF646//BRF1//GSK3B//LDLR//ICAM1//TFRC//GNAI2//PLCG2//OSBPL10//THBS1//MDM2//TRIM24//ZCCHC2//HUWE1//DDX11//PDS5B//LCORL//THAP4//CRLF3//TERF2IP//GPBP1L1//RNF4//TADA2A//TMF1//ZNF236//TAF15//ZMYM4//GTF3C5//SCMH1//SIRT6//OVOL2//POLI//LSM14A//AFF3//PCBP2//DDX17//IGF2BP3//LARP4//CCDC124//DDX6//DYNC1H1//HABP4//SMG1//GPATCH8//FLNB//FAM120A//R3HDM1//RPL13A//MTO1//GDI2//KPNB1//CAPRIN1//MYH9//RPS27L//CDC40//ALKBH5//RANGAP1//RPL18A//RPL37A//RPN1//RPS14//ARHGEF28//TRA2B//SLC3A2//NOL10//FIP1L1//CANX//ZCCHC7//CNOT8//PUM1//TUBA1B//MYO9B//OPHN1//MYO19//ENC1//SPAG9//DOCK1//TIAM2//BCR//TRIO//EFEMP1//CYP24A1//NID1//SUSD1//EFCAB6//SLIT3//RCL1//CDR2//IFI30//SDCCAG8//TUBGCP2//FAF1//RABL2B//GABARAP//AGAP1//AP3S1//CLK1//SLC15A4//COL6A2//TMEM120B//DCTD//DNM2//DOCK2//DYRK1A//EYA3//TBCEL//LNX2//PHLPP2//NFASC//ARL6IP1//PLEKHM2//TBC1D1//NEDD4L//AHCYL2//ZDHHC17//PIP5K1C//NOMO1//CORO1C//NALCN//GAPDH//RAB11FIP5//WSB1//LATS2//SESN1//GCLM//GNB1//FRYL//SH3PXD2B//SETD2//HBG2//HK1//MIA3//KPNA5//KRT17//CHCHD10//MCC//MDH1//MTR//NASP//NF1//PCCB//CEP83//TAOK3//PDE3A//TRAPPC4//PDE4D//PI4KA//ATP6V0B//RNF216//FAM193B//FBLIM1//RNF111//TEX10//TTC19//CLN6//GOLPH3L//PPT1//CNOT11//AMBRA1//G2E3//CFAP44//EMC3//PARD3//PSMB5//FAM219B//HECW2//WDFY1//PTPRG//ABCD4//RAB2A//UBL5//BCKDHA//RPN2//LMBR1//BMPR2//STAC//STRN//GPR137B//UMAD1//UGP2//DDR1//ELOVL6//DCAF17//MOB2//TRRAP//DYNLRB1//CD99L2//ATRIP//USP42//HOOK3//FAM120B//ADAM19//STARD13//NEK9//FANK1//LPXN//RPH3AL//TMEM59//NUP155//PDE4DIP//KLHL21//PHKG1//TTLL11//ATP13A1//STARD9//PKDCC//RANBP17//CCSER2//FTO//PRSS57//EVA1C//PITRM1//SIRT5//GATAD1//PAPPA2//DENND5B//PHRF1//GALNT1//GALNT7//PITPNB//SMYD4//RALGAPA2//RCSD1//WDR1// |
| GO:0000166 | nucleotide binding | Molecular function | 50 | 2130 | 268 | 18352 | 1.60745567934973 | 0.000456438067455767 | 0.0404742993642677 | 3.34061814208786 | 0.186567164179104 | ATP8A1//DDX17//CLK1//TTLL11//DDX6//DDX11//DYNC1H1//DYRK1A//MORC2//SMG1//PIP5K1C//LATS2//GSK3B//HK1//MYH9//MYO9B//PCCB//TAOK3//PHKG1//PI4KA//NAT10//ATP13A1//STARD9//ABCD4//ACTB//BCR//BMPR2//SRPK2//TRIO//DDR1//MYO19//PKDCC//NEK9//TUBA1B//RABL2B//AGAP1//DNM2//GNAI1//GNAI2//RAB2A//RANBP17//PDE4D//UGP2//MTO1//GAPDH//MDH1//SIRT5//SIRT6//VDAC3//AKAP7// |
| GO:1901265 | nucleoside phosphate binding | Molecular function | 50 | 2131 | 268 | 18352 | 1.60670135946266 | 0.000461404371886281 | 0.0404742993642677 | 3.33591829478005 | 0.186567164179104 | VDAC3//AKAP7//ATP8A1//DDX17//CLK1//TTLL11//DDX6//DDX11//DYNC1H1//DYRK1A//MORC2//SMG1//PIP5K1C//LATS2//GSK3B//HK1//MYH9//MYO9B//PCCB//TAOK3//PHKG1//PI4KA//NAT10//ATP13A1//STARD9//ABCD4//ACTB//BCR//BMPR2//SRPK2//TRIO//DDR1//MYO19//PKDCC//NEK9//TUBA1B//RABL2B//AGAP1//DNM2//GNAI1//GNAI2//RAB2A//RANBP17//PDE4D//UGP2//MTO1//GAPDH//MDH1//SIRT5//SIRT6// |
| GO:0051020 | GTPase binding | Molecular function | 19 | 548 | 268 | 18352 | 2.37422377165269 | 0.000465935909795868 | 0.0404742993642677 | 3.33167381707871 | 0.0708955223880597 | DOCK1//TIAM2//BCR//ARHGEF28//TRIO//ANKRD27//KPNB1//RANGAP1//RANBP17//MYO9B//DENND5B//TBC1D1//RAB11FIP5//GDI2//RPH3AL//DOCK2//CORO1C//GNB1//AMBRA1// |
| GO:0008092 | cytoskeletal protein binding | Molecular function | 28 | 980 | 268 | 18352 | 1.95650319829424 | 0.000547841885900477 | 0.0446148735830201 | 3.26134476631348 | 0.104477611940299 | FLNB//MYH9//MYO9B//OPHN1//MYO19//ENC1//GABARAP//DNM2//GAPDH//CCSER2//STARD9//HOOK3//TTLL11//PLEKHM2//ACTB//SPAG9//GNB1//FBLIM1//GSK3B//TBCEL//SETD2//TUBGCP2//RAB11FIP5//DYRK1A//CORO1C//RCSD1//WDR1//RPH3AL// |
| GO:0060589 | nucleoside-triphosphatase regulator activity | Molecular function | 14 | 348 | 268 | 18352 | 2.75484645736833 | 0.000631012196329663 | 0.0483652289304442 | 3.19996224654432 | 0.0522388059701493 | GDI2//AGAP1//DOCK1//DOCK2//TBC1D1//TIAM2//MYO9B//NF1//OPHN1//RALGAPA2//RANGAP1//BCR//ANKRD27//STARD13// |
| GO:0043168 | anion binding | Molecular function | 61 | 2829 | 268 | 18352 | 1.47654094321605 | 0.000911912447683063 | 0.066012328851724 | 3.04004685609414 | 0.227611940298507 | OSBPL10//THBS1//ATP8A1//DDX17//CLK1//TTLL11//DDX6//DDX11//DYNC1H1//DYRK1A//MORC2//SMG1//PIP5K1C//LATS2//GSK3B//HK1//MYH9//MYO9B//PCCB//TAOK3//PHKG1//PI4KA//NAT10//ATP13A1//STARD9//ABCD4//ACTB//BCR//BMPR2//SRPK2//TRIO//DDR1//MYO19//PKDCC//NEK9//TUBA1B//RABL2B//AGAP1//DNM2//GNAI1//GNAI2//RAB2A//RANBP17//OPHN1//WDFY1//PARD3//PRSS57//EVA1C//SLIT3//NF1//SH3PXD2B//PDE4D//PITPNB//ZCCHC2//CLN6//PPT1//MTO1//GOLPH3L//SIRT5//SIRT6//SESN1// |
| GO:0043169 | cation binding | Molecular function | 85 | 4314 | 268 | 18352 | 1.34923435672818 | 0.00122986883493397 | 0.080155685927021 | 2.91014120344019 | 0.317164179104478 | ATP8A1//MORC2//GNAI1//SRPK2//CYP24A1//EFEMP1//LDLR//NID1//SUSD1//EFCAB6//SLIT3//THBS1//CANX//FTO//PITRM1//CTCF//DCTD//SIRT5//KPNB1//MDM2//MTR//SIRT6//ZCCHC2//WDFY1//GATAD1//RNF4//PAPPA2//NR2C2//ZCCHC7//KLF7//TRIM24//ZMYM4//GALNT1//PITPNB//NF1//PRDM5//POLI//SMYD4//AGAP1//ZNF483//DDX11//EYA3//LNX2//ZNF652//PHLPP2//SMG1//GPATCH8//LATS2//GNAI2//SETD2//BRF1//HBG2//MYO9B//RPS27L//THAP4//PDE3A//PDE4D//GALNT7//PLAGL1//RNF216//FBLIM1//RNF111//ALKBH5//G2E3//ATP13A1//PHRF1//OVOL2//BCKDHA//RPL37A//ARHGEF28//BMPR2//STAC//UGP2//ZNF236//DDR1//TAF15//MOB2//KLF11//ADAM19//NEK9//CNOT8//LPXN//RPH3AL//ZNF646//SESN1// |
| GO:0032183 | SUMO binding | Molecular function | 3 | 15 | 268 | 18352 | 13.6955223880597 | 0.00123032518690746 | 0.080155685927021 | 2.90998008513456 | 0.0111940298507463 | RNF111//RNF4//HABP4// |
| GO:0097367 | carbohydrate derivative binding | Molecular function | 50 | 2240 | 268 | 18352 | 1.52851812366738 | 0.00140227184246836 | 0.0869618054212138 | 2.85316778648364 | 0.186567164179104 | ATP8A1//DDX17//CLK1//TTLL11//DDX6//DDX11//DYNC1H1//DYRK1A//MORC2//SMG1//PIP5K1C//LATS2//GSK3B//HK1//MYH9//MYO9B//PCCB//TAOK3//PHKG1//PI4KA//NAT10//ATP13A1//STARD9//ABCD4//ACTB//BCR//BMPR2//SRPK2//TRIO//DDR1//MYO19//PKDCC//NEK9//TUBA1B//RABL2B//AGAP1//DNM2//GNAI1//GNAI2//RAB2A//RANBP17//PRSS57//EVA1C//SLIT3//THBS1//PDE4D//UGP2//CLN6//PPT1//NID1// |
| GO:0005088 | Ras guanyl-nucleotide exchange factor activity | Molecular function | 7 | 115 | 268 | 18352 | 4.16820246593121 | 0.00150232659388417 | 0.0869618054212138 | 2.82323564489285 | 0.0261194029850746 | DOCK1//BCR//ARHGEF28//TRIO//DENND5B//DOCK2//TIAM2// |
| GO:0003824 | catalytic activity | Molecular function | 106 | 5673 | 268 | 18352 | 1.27950411875051 | 0.00153501268203217 | 0.0869618054212138 | 2.8138880320996 | 0.395522388059701 | MYH9//MYO9B//MYO19//CNOT8//SIAE//COQ2//DDX11//DDX17//DDX6//STARD9//DYNLRB1//BCKDHA//MBOAT2//POLI//TUBA1B//RABL2B//AGAP1//DNM2//GNAI1//GNAI2//GNB1//RAB2A//SIRT6//UGP2//ACP6//CRAT//PDE3A//PDE4D//COX6B1//DCTD//PSMB5//TMEM59//USP42//PITRM1//ADAM19//PRSS57//HK1//GCLM//GAPDH//TADA2A//PI4KA//PLCG2//MDH1//RCL1//RPPH1//RPN1//RPN2//HBG2//GALNT1//GALNT7//PCCB//DYRK1A//SMG1//GSK3B//TRIM24//PKDCC//CLK1//LATS2//TAOK3//BCR//BMPR2//SRPK2//TRIO//NEK9//PHKG1//DDR1//PHLPP2//EYA3//PTPRG//HUWE1//NEDD4L//MDM2//G2E3//RNF4//KLHL21//EFEMP1//NAT10//NDUFB2//SMYD4//CFAP44//DPP8//PAPPA2//CYP24A1//PPT1//DYNC1H1//MTR//ELOVL6//SETD2//PIP5K1C//ZDHHC17//IFI30//SESN1//ALKBH5//FTO//ATP8A1//MORC2//ATP13A1//ABCD4//SIRT5//PRDM5//RNF216//RNF111//HECW2//UBE3B//TTLL11//SLC3A2// |
| GO:0061629 | RNA polymerase II-specific DNA-binding transcription factor binding | Molecular function | 11 | 267 | 268 | 18352 | 2.82117502375762 | 0.00196397472116606 | 0.105848491791962 | 2.70686410643132 | 0.041044776119403 | RBL1//RBPJ//TRIM24//STRN//NRIP1//ACTB//NCOR2//TMF1//FAF1//GSK3B//SPI1// |
| GO:0008047 | enzyme activator activity | Molecular function | 17 | 528 | 268 | 18352 | 2.20477159656264 | 0.00203086131603917 | 0.105848491791962 | 2.69231973279183 | 0.0634328358208955 | AGAP1//DOCK1//DOCK2//TBC1D1//TIAM2//GDI2//MYO9B//NF1//OPHN1//RALGAPA2//RANGAP1//BCR//ANKRD27//STARD13//RPS27L//SH3PXD2B//PITRM1// |
| GO:0036094 | small molecule binding | Molecular function | 54 | 2523 | 268 | 18352 | 1.46563259800877 | 0.00225608306362007 | 0.113064470457575 | 2.64664491470299 | 0.201492537313433 | VDAC3//AKAP7//ATP8A1//DDX17//CLK1//TTLL11//DDX6//DDX11//DYNC1H1//DYRK1A//MORC2//SMG1//PIP5K1C//LATS2//GSK3B//HK1//MYH9//MYO9B//PCCB//TAOK3//PHKG1//PI4KA//NAT10//ATP13A1//STARD9//ABCD4//ACTB//BCR//BMPR2//SRPK2//TRIO//DDR1//MYO19//PKDCC//NEK9//TUBA1B//RABL2B//AGAP1//DNM2//GNAI1//GNAI2//RAB2A//RANBP17//UGP2//OSBPL10//HBG2//PDE4D//MTR//MTO1//GAPDH//MDH1//SIRT5//SIRT6//SESN1// |
| GO:0032553 | ribonucleotide binding | Molecular function | 43 | 1899 | 268 | 18352 | 1.55057257158127 | 0.00241576132891826 | 0.116582852280759 | 2.61694597512291 | 0.16044776119403 | ATP8A1//DDX17//CLK1//TTLL11//DDX6//DDX11//DYNC1H1//DYRK1A//MORC2//SMG1//PIP5K1C//LATS2//GSK3B//HK1//MYH9//MYO9B//PCCB//TAOK3//PHKG1//PI4KA//NAT10//ATP13A1//STARD9//ABCD4//ACTB//BCR//BMPR2//SRPK2//TRIO//DDR1//MYO19//PKDCC//NEK9//TUBA1B//RABL2B//AGAP1//DNM2//GNAI1//GNAI2//RAB2A//RANBP17//PDE4D//UGP2// |
| GO:0046872 | metal ion binding | Molecular function | 82 | 4229 | 268 | 18352 | 1.32777587588188 | 0.00251487846220569 | 0.1170316655805 | 2.59948299846842 | 0.305970149253731 | ATP8A1//MORC2//GNAI1//SRPK2//CYP24A1//EFEMP1//LDLR//NID1//SUSD1//EFCAB6//SLIT3//THBS1//CANX//FTO//PITRM1//CTCF//DCTD//SIRT5//KPNB1//MDM2//MTR//SIRT6//ZCCHC2//WDFY1//GATAD1//RNF4//PAPPA2//NR2C2//ZCCHC7//KLF7//TRIM24//ZMYM4//GALNT1//PRDM5//POLI//SMYD4//AGAP1//ZNF483//DDX11//EYA3//LNX2//ZNF652//PHLPP2//SMG1//GPATCH8//LATS2//GNAI2//SETD2//BRF1//HBG2//MYO9B//RPS27L//THAP4//PDE3A//PDE4D//GALNT7//PLAGL1//RNF216//FBLIM1//RNF111//ALKBH5//G2E3//ATP13A1//PHRF1//OVOL2//BCKDHA//RPL37A//ARHGEF28//BMPR2//STAC//UGP2//ZNF236//DDR1//TAF15//MOB2//KLF11//ADAM19//NEK9//CNOT8//LPXN//RPH3AL//ZNF646// |
| GO:0017048 | Rho GTPase binding | Molecular function | 8 | 162 | 268 | 18352 | 3.38161046618758 | 0.00266303018761374 | 0.1196527011883 | 2.57462391045187 | 0.0298507462686567 | DOCK1//BCR//ARHGEF28//TRIO//DOCK2//TIAM2//CORO1C//MYO9B// |
| GO:0140096 | catalytic activity, acting on a protein | Molecular function | 48 | 2198 | 268 | 18352 | 1.49541645729496 | 0.00276091292560226 | 0.119915651401991 | 2.55894729003831 | 0.17910447761194 | PSMB5//TMEM59//USP42//PITRM1//ADAM19//PRSS57//SIRT6//GALNT1//GALNT7//DYRK1A//SMG1//GSK3B//TRIM24//PKDCC//CLK1//LATS2//TAOK3//BCR//BMPR2//SRPK2//TRIO//NEK9//PHKG1//DDR1//PHLPP2//EYA3//PTPRG//HUWE1//NEDD4L//MDM2//G2E3//RNF4//KLHL21//EFEMP1//CFAP44//DPP8//PAPPA2//PPT1//SETD2//ZDHHC17//SIRT5//GAPDH//PRDM5//RNF216//RNF111//HECW2//UBE3B//TTLL11// |
| GO:0016740 | transferase activity | Molecular function | 49 | 2261 | 268 | 18352 | 1.48403493369068 | 0.00289589000150914 | 0.121720795869884 | 2.53821793855392 | 0.182835820895522 | COQ2//MBOAT2//POLI//SIRT6//UGP2//CRAT//HK1//TADA2A//PI4KA//RPN1//RPN2//GALNT1//GALNT7//DYRK1A//SMG1//GSK3B//TRIM24//PKDCC//CLK1//LATS2//TAOK3//BCR//BMPR2//SRPK2//TRIO//NEK9//PHKG1//DDR1//HUWE1//NEDD4L//MDM2//G2E3//RNF4//KLHL21//EFEMP1//NAT10//SMYD4//MTR//ELOVL6//SETD2//PIP5K1C//ZDHHC17//GAPDH//PRDM5//RNF216//RNF111//HECW2//UBE3B//FTO// |
| GO:0035639 | purine ribonucleoside triphosphate binding | Molecular function | 41 | 1816 | 268 | 18352 | 1.54602537970938 | 0.00323451139980591 | 0.131705261060847 | 2.49019131388071 | 0.152985074626866 | ATP8A1//DDX17//CLK1//TTLL11//DDX6//DDX11//DYNC1H1//DYRK1A//MORC2//SMG1//PIP5K1C//LATS2//GSK3B//HK1//MYH9//MYO9B//PCCB//TAOK3//PHKG1//PI4KA//NAT10//ATP13A1//STARD9//ABCD4//ACTB//BCR//BMPR2//SRPK2//TRIO//DDR1//MYO19//PKDCC//NEK9//TUBA1B//RABL2B//AGAP1//DNM2//GNAI1//GNAI2//RAB2A//RANBP17// |
| GO:0032555 | purine ribonucleotide binding | Molecular function | 42 | 1882 | 268 | 18352 | 1.52819325265278 | 0.00354340114801259 | 0.139910657450315 | 2.45057967845813 | 0.156716417910448 | ATP8A1//DDX17//CLK1//TTLL11//DDX6//DDX11//DYNC1H1//DYRK1A//MORC2//SMG1//PIP5K1C//LATS2//GSK3B//HK1//MYH9//MYO9B//PCCB//TAOK3//PHKG1//PI4KA//NAT10//ATP13A1//STARD9//ABCD4//ACTB//BCR//BMPR2//SRPK2//TRIO//DDR1//MYO19//PKDCC//NEK9//TUBA1B//RABL2B//AGAP1//DNM2//GNAI1//GNAI2//RAB2A//RANBP17//PDE4D// |
| GO:0050321 | tau-protein kinase activity | Molecular function | 3 | 22 | 268 | 18352 | 9.33785617367707 | 0.00386162756306491 | 0.147991197490399 | 2.41322961432292 | 0.0111940298507463 | DYRK1A//GSK3B//PHKG1// |
| GO:0017076 | purine nucleotide binding | Molecular function | 42 | 1896 | 268 | 18352 | 1.51690912525978 | 0.00404330294553273 | 0.150526392515118 | 2.39326371781938 | 0.156716417910448 | ATP8A1//DDX17//CLK1//TTLL11//DDX6//DDX11//DYNC1H1//DYRK1A//MORC2//SMG1//PIP5K1C//LATS2//GSK3B//HK1//MYH9//MYO9B//PCCB//TAOK3//PHKG1//PI4KA//NAT10//ATP13A1//STARD9//ABCD4//ACTB//BCR//BMPR2//SRPK2//TRIO//DDR1//MYO19//PKDCC//NEK9//TUBA1B//RABL2B//AGAP1//DNM2//GNAI1//GNAI2//RAB2A//RANBP17//PDE4D// |
| GO:0016773 | phosphotransferase activity, alcohol group as acceptor | Molecular function | 19 | 669 | 268 | 18352 | 1.94480512237021 | 0.00453868517431686 | 0.164275188392635 | 2.34306994102866 | 0.0708955223880597 | HK1//PI4KA//DYRK1A//SMG1//GSK3B//TRIM24//PKDCC//CLK1//LATS2//TAOK3//BCR//BMPR2//SRPK2//TRIO//NEK9//PHKG1//DDR1//EFEMP1//PIP5K1C// |
| GO:0140297 | DNA-binding transcription factor binding | Molecular function | 12 | 347 | 268 | 18352 | 2.36810185384318 | 0.00518107864030081 | 0.182457985630053 | 2.28557981578285 | 0.0447761194029851 | RBL1//RBPJ//TRIM24//STRN//NRIP1//ACTB//NCOR2//TMF1//FAF1//GSK3B//SPI1//PRDM5// |
| GO:0017111 | nucleoside-triphosphatase activity | Molecular function | 21 | 802 | 268 | 18352 | 1.79305467674098 | 0.00724664666902055 | 0.248483700256152 | 2.1398629134348 | 0.0783582089552239 | MYH9//MYO9B//MYO19//DDX11//DDX17//DDX6//STARD9//DYNLRB1//TUBA1B//RABL2B//AGAP1//DNM2//GNAI1//GNAI2//GNB1//RAB2A//DYNC1H1//ATP8A1//MORC2//ATP13A1//ABCD4// |
| GO:0051287 | NAD binding | Molecular function | 4 | 55 | 268 | 18352 | 4.98018995929444 | 0.00846354990931959 | 0.276659023150773 | 2.07244744042897 | 0.0149253731343284 | SIRT5//SIRT6//GAPDH//MDH1// |
| GO:0000146 | microfilament motor activity | Molecular function | 3 | 29 | 268 | 18352 | 7.08389089037571 | 0.00850016599435464 | 0.276659023150773 | 2.0705725931412 | 0.0111940298507463 | MYO19//MYH9//MYO9B// |
| GO:0015173 | aromatic amino acid transmembrane transporter activity | Molecular function | 2 | 10 | 268 | 18352 | 13.6955223880597 | 0.00884945841645505 | 0.276659023150773 | 2.05308330714875 | 0.00746268656716418 | SLC15A4//SLC3A2// |
| GO:0004672 | protein kinase activity | Molecular function | 16 | 564 | 268 | 18352 | 1.94262728908648 | 0.00897034879462016 | 0.276659023150773 | 2.04719066992873 | 0.0597014925373134 | CLK1//DYRK1A//SMG1//LATS2//GSK3B//TAOK3//BCR//BMPR2//SRPK2//TRIO//NEK9//PHKG1//DDR1//PKDCC//EFEMP1//TRIM24// |
| GO:0005524 | ATP binding | Molecular function | 33 | 1475 | 268 | 18352 | 1.53204148747786 | 0.00933014549309347 | 0.276659023150773 | 2.03011158386819 | 0.123134328358209 | ATP8A1//DDX17//CLK1//TTLL11//DDX6//DDX11//DYNC1H1//DYRK1A//MORC2//SMG1//PIP5K1C//LATS2//GSK3B//HK1//MYH9//MYO9B//PCCB//TAOK3//PHKG1//PI4KA//NAT10//ATP13A1//STARD9//ABCD4//ACTB//BCR//BMPR2//SRPK2//TRIO//DDR1//MYO19//PKDCC//NEK9// |
| GO:0045505 | dynein intermediate chain binding | Molecular function | 3 | 30 | 268 | 18352 | 6.84776119402985 | 0.0093442181853766 | 0.276659023150773 | 2.02945702943557 | 0.0111940298507463 | DYNC1H1//DYNLRB1//HOOK3// |
| GO:0032559 | adenyl ribonucleotide binding | Molecular function | 34 | 1534 | 268 | 18352 | 1.51775671836385 | 0.00955460939507658 | 0.276659023150773 | 2.01978706277227 | 0.126865671641791 | ATP8A1//DDX17//CLK1//TTLL11//DDX6//DDX11//DYNC1H1//DYRK1A//MORC2//SMG1//PIP5K1C//LATS2//GSK3B//HK1//MYH9//MYO9B//PCCB//TAOK3//PHKG1//PI4KA//NAT10//ATP13A1//STARD9//ABCD4//ACTB//BCR//BMPR2//SRPK2//TRIO//DDR1//MYO19//PKDCC//NEK9//PDE4D// |
| GO:0140142 | nucleocytoplasmic carrier activity | Molecular function | 3 | 31 | 268 | 18352 | 6.62686567164179 | 0.0102354798855935 | 0.289931093281051 | 1.98989179083445 | 0.0111940298507463 | RANBP17//KPNB1//KPNA5// |
| GO:0030554 | adenyl nucleotide binding | Molecular function | 34 | 1546 | 268 | 18352 | 1.50597594176594 | 0.01067464016995 | 0.290799006255205 | 1.97164675563212 | 0.126865671641791 | ATP8A1//DDX17//CLK1//TTLL11//DDX6//DDX11//DYNC1H1//DYRK1A//MORC2//SMG1//PIP5K1C//LATS2//GSK3B//HK1//MYH9//MYO9B//PCCB//TAOK3//PHKG1//PI4KA//NAT10//ATP13A1//STARD9//ABCD4//ACTB//BCR//BMPR2//SRPK2//TRIO//DDR1//MYO19//PKDCC//NEK9//PDE4D// |
| GO:0005536 | glucose binding | Molecular function | 2 | 11 | 268 | 18352 | 12.4504748982361 | 0.01071247298561 | 0.290799006255205 | 1.97011026025804 | 0.00746268656716418 | HK1//UGP2// |
| GO:0016301 | kinase activity | Molecular function | 19 | 733 | 268 | 18352 | 1.77499949094907 | 0.0114992761245988 | 0.305786873272495 | 1.93932949747135 | 0.0708955223880597 | HK1//PI4KA//DYRK1A//SMG1//GSK3B//TRIM24//PKDCC//CLK1//LATS2//TAOK3//BCR//BMPR2//SRPK2//TRIO//NEK9//PHKG1//DDR1//EFEMP1//PIP5K1C// |
| GO:0032182 | ubiquitin-like protein binding | Molecular function | 5 | 94 | 268 | 18352 | 3.64242616703715 | 0.0122435466145754 | 0.319036235570356 | 1.91209276094613 | 0.0186567164179104 | HABP4//RNF111//RNF4//FAF1//MDM2// |
| GO:0004115 | 3',5'-cyclic-AMP phosphodiesterase activity | Molecular function | 2 | 12 | 268 | 18352 | 11.4129353233831 | 0.0127320677280572 | 0.319036235570356 | 1.89510105982143 | 0.00746268656716418 | PDE3A//PDE4D// |
| GO:0034452 | dynactin binding | Molecular function | 2 | 12 | 268 | 18352 | 11.4129353233831 | 0.0127320677280572 | 0.319036235570356 | 1.89510105982143 | 0.00746268656716418 | GSK3B//HOOK3// |
| GO:0003774 | motor activity | Molecular function | 6 | 132 | 268 | 18352 | 3.11261872455902 | 0.0130266675040006 | 0.320259391654958 | 1.88516667175715 | 0.0223880597014925 | MYH9//MYO9B//MYO19//STARD9//DYNLRB1//DYNC1H1// |
| GO:0005085 | guanyl-nucleotide exchange factor activity | Molecular function | 8 | 215 | 268 | 18352 | 2.54800416522041 | 0.0139249552660981 | 0.332320783593362 | 1.85620619132249 | 0.0298507462686567 | DOCK1//BCR//ARHGEF28//TRIO//DENND5B//DOCK2//TIAM2//ANKRD27// |
| GO:0016462 | pyrophosphatase activity | Molecular function | 21 | 854 | 268 | 18352 | 1.68387570344996 | 0.0140287875942602 | 0.332320783593362 | 1.85297986025279 | 0.0783582089552239 | MYH9//MYO9B//MYO19//DDX11//DDX17//DDX6//STARD9//DYNLRB1//TUBA1B//RABL2B//AGAP1//DNM2//GNAI1//GNAI2//GNB1//RAB2A//DYNC1H1//ATP8A1//MORC2//ATP13A1//ABCD4// |
| GO:0016817 | hydrolase activity, acting on acid anhydrides | Molecular function | 21 | 857 | 268 | 18352 | 1.67798115606332 | 0.0145374402646367 | 0.332320783593362 | 1.83751205680636 | 0.0783582089552239 | MYH9//MYO9B//MYO19//DDX11//DDX17//DDX6//STARD9//DYNLRB1//TUBA1B//RABL2B//AGAP1//DNM2//GNAI1//GNAI2//GNB1//RAB2A//DYNC1H1//ATP8A1//MORC2//ATP13A1//ABCD4// |
| GO:0016818 | hydrolase activity, acting on acid anhydrides, in phosphorus-containing anhydrides | Molecular function | 21 | 857 | 268 | 18352 | 1.67798115606332 | 0.0145374402646367 | 0.332320783593362 | 1.83751205680636 | 0.0783582089552239 | MYH9//MYO9B//MYO19//DDX11//DDX17//DDX6//STARD9//DYNLRB1//TUBA1B//RABL2B//AGAP1//DNM2//GNAI1//GNAI2//GNB1//RAB2A//DYNC1H1//ATP8A1//MORC2//ATP13A1//ABCD4// |
| GO:0016922 | nuclear receptor binding | Molecular function | 5 | 101 | 268 | 18352 | 3.38998078912369 | 0.0162856503747445 | 0.36270967226065 | 1.78819489300806 | 0.0186567164179104 | STRN//NRIP1//NCOR2//TMF1//TRIM24// |
| GO:0005543 | phospholipid binding | Molecular function | 13 | 454 | 268 | 18352 | 1.96081267670458 | 0.0164235384983717 | 0.36270967226065 | 1.78453326714614 | 0.0485074626865672 | OSBPL10//THBS1//WDFY1//PARD3//NF1//SH3PXD2B//PITPNB//ZCCHC2//CLN6//PPT1//GOLPH3L//AGAP1//OPHN1// |
| GO:0050998 | nitric-oxide synthase binding | Molecular function | 2 | 14 | 268 | 18352 | 9.78251599147122 | 0.017221366040834 | 0.367859671331257 | 1.76393240221651 | 0.00746268656716418 | DNM2//ACTB// |
| GO:0070403 | NAD+ binding | Molecular function | 2 | 14 | 268 | 18352 | 9.78251599147122 | 0.017221366040834 | 0.367859671331257 | 1.76393240221651 | 0.00746268656716418 | SIRT5//SIRT6// |
| GO:0015631 | tubulin binding | Molecular function | 11 | 365 | 268 | 18352 | 2.0637088529953 | 0.0187819979553051 | 0.394724892512299 | 1.72625821106825 | 0.041044776119403 | GABARAP//DNM2//GAPDH//CCSER2//STARD9//HOOK3//TBCEL//SETD2//TUBGCP2//RAB11FIP5//TTLL11// |
| GO:0005518 | collagen binding | Molecular function | 4 | 70 | 268 | 18352 | 3.91300639658849 | 0.0192190948080654 | 0.397499691030305 | 1.71626707083604 | 0.0149253731343284 | THBS1//COL6A2//NID1//DDR1// |
| GO:0035259 | glucocorticoid receptor binding | Molecular function | 2 | 15 | 268 | 18352 | 9.13034825870647 | 0.0196815261722073 | 0.400703571912283 | 1.70594122793368 | 0.00746268656716418 | NRIP1//NCOR2// |
| GO:0016772 | transferase activity, transferring phosphorus-containing groups | Molecular function | 21 | 885 | 268 | 18352 | 1.62489248671895 | 0.0200233652672208 | 0.401391460664442 | 1.69846293014537 | 0.0783582089552239 | POLI//UGP2//HK1//PI4KA//DYRK1A//SMG1//GSK3B//TRIM24//PKDCC//CLK1//LATS2//TAOK3//BCR//BMPR2//SRPK2//TRIO//NEK9//PHKG1//DDR1//EFEMP1//PIP5K1C// |
| GO:0019899 | enzyme binding | Molecular function | 45 | 2277 | 268 | 18352 | 1.35331248893871 | 0.0210082988571454 | 0.414754748649401 | 1.67760911308028 | 0.167910447761194 | GSK3B//LDLR//SPAG9//DOCK1//TIAM2//BCR//ARHGEF28//TRIO//ANKRD27//KPNB1//RANGAP1//RANBP17//MYO9B//DENND5B//TBC1D1//RAB11FIP5//GDI2//RPH3AL//FAF1//DNM2//ACTB//TFRC//NEK9//AKAP7//NFATC2//TERF2IP//PARD3//DOCK2//TUBA1B//GABARAP//MDM2//PCBP2//AMBRA1//GCLM//NRIP1//NCOR2//CORO1C//GNB1//PDE4D//STRN//PHRF1//NAT10//BMPR2//PHKG1//PDE4DIP// |
| GO:0008536 | Ran GTPase binding | Molecular function | 3 | 41 | 268 | 18352 | 5.01055697124135 | 0.0218245932114802 | 0.424439476933712 | 1.66105384236035 | 0.0111940298507463 | KPNB1//RANGAP1//RANBP17// |
| GO:0034979 | NAD-dependent protein deacetylase activity | Molecular function | 2 | 16 | 268 | 18352 | 8.55970149253731 | 0.0222791753670854 | 0.42690831622518 | 1.65210088801143 | 0.00746268656716418 | SIRT6//SIRT5// |
| GO:0019894 | kinesin binding | Molecular function | 3 | 43 | 268 | 18352 | 4.77750780978827 | 0.0247368471018293 | 0.461486495189205 | 1.60665565529448 | 0.0111940298507463 | PLEKHM2//ACTB//SPAG9// |
| GO:0030169 | low-density lipoprotein particle binding | Molecular function | 2 | 17 | 268 | 18352 | 8.05618964003512 | 0.0250098048581527 | 0.461486495189205 | 1.60188969688837 | 0.00746268656716418 | LDLR//THBS1// |
| GO:0003725 | double-stranded RNA binding | Molecular function | 4 | 76 | 268 | 18352 | 3.60408483896308 | 0.025146232661883 | 0.461486495189205 | 1.599527070517 | 0.0149253731343284 | TUBA1B//LSM14A//SLC3A2//TFRC// |
| GO:0001227 | DNA-binding transcription repressor activity, RNA polymerase II-specific | Molecular function | 10 | 335 | 268 | 18352 | 2.04410781911339 | 0.0258672350948982 | 0.465459756962413 | 1.58724998980689 | 0.0373134328358209 | CTCF//TCFL5//PRDM5//IFI16//NFATC2//PLAGL1//OVOL2//BACH2//SPI1//GTF2IRD1// |
| GO:0001217 | DNA-binding transcription repressor activity | Molecular function | 10 | 336 | 268 | 18352 | 2.03802416488984 | 0.0263313482037459 | 0.465459756962413 | 1.57952690380858 | 0.0373134328358209 | CTCF//TCFL5//PRDM5//IFI16//NFATC2//PLAGL1//OVOL2//BACH2//SPI1//GTF2IRD1// |
| GO:0004674 | protein serine/threonine kinase activity | Molecular function | 12 | 435 | 268 | 18352 | 1.88903757076686 | 0.0266520781058981 | 0.465459756962413 | 1.57426892266974 | 0.0447761194029851 | PHKG1//SRPK2//BMPR2//CLK1//DYRK1A//SMG1//LATS2//GSK3B//TAOK3//BCR//TRIO//NEK9// |
| GO:0008134 | transcription factor binding | Molecular function | 16 | 642 | 268 | 18352 | 1.70660715116009 | 0.026898115086437 | 0.465459756962413 | 1.57027815257161 | 0.0597014925373134 | GSK3B//SPI1//RBL1//RBPJ//TRIM24//BRF1//STRN//NRIP1//ACTB//NCOR2//TMF1//FAF1//PRDM5//IFI16//NFATC2//RNF4// |
| GO:0003677 | DNA binding | Molecular function | 48 | 2498 | 268 | 18352 | 1.31582280749973 | 0.0271488423093963 | 0.465459756962413 | 1.56624868502028 | 0.17910447761194 | CTCF//PRDM5//KLF11//PLAGL1//RBL1//NPAS3//NR2C2//NCOR2//TCFL5//ZNF483//ZNF652//IFI16//RBPJ//MEIS1//NFATC2//CCAR1//RFX2//ACTB//BACH2//SPI1//NRIP1//KLF7//GTF2IRD1//ZNF646//BRF1//POLI//DDX11//LSM14A//AFF3//PCBP2//TRIM24//SMG1//TERF2IP//GATAD1//OVOL2//HUWE1//PDS5B//LCORL//THAP4//CRLF3//GPBP1L1//RNF4//TADA2A//TMF1//ZNF236//TAF15//ZMYM4//GTF3C5// |
| GO:0048156 | tau protein binding | Molecular function | 3 | 45 | 268 | 18352 | 4.56517412935323 | 0.0278481221467735 | 0.465555341280994 | 1.55520408482742 | 0.0111940298507463 | DYRK1A//GSK3B//ACTB// |
| GO:0046965 | retinoid X receptor binding | Molecular function | 2 | 18 | 268 | 18352 | 7.60862354892206 | 0.0278690073828991 | 0.465555341280994 | 1.55487849938166 | 0.00746268656716418 | NRIP1//NCOR2// |
| GO:0003682 | chromatin binding | Molecular function | 15 | 596 | 268 | 18352 | 1.72342983071221 | 0.0292803024577611 | 0.48012568462199 | 1.53342444144362 | 0.0559701492537313 | RNF4//ACTB//CTCF//RBL1//DDX11//MORC2//SCMH1//RBPJ//MEIS1//NFATC2//SIRT6//OVOL2//TADA2A//TRIM24//NCOR2// |
| GO:0004715 | non-membrane spanning protein tyrosine kinase activity | Molecular function | 3 | 46 | 268 | 18352 | 4.46593121349773 | 0.029478169431895 | 0.48012568462199 | 1.53049948927672 | 0.0111940298507463 | CLK1//DYRK1A//PKDCC// |
| GO:0035258 | steroid hormone receptor binding | Molecular function | 4 | 81 | 268 | 18352 | 3.38161046618758 | 0.0308401258419805 | 0.484346688963476 | 1.51088385849507 | 0.0149253731343284 | STRN//NRIP1//NCOR2//TMF1// |
| GO:0004653 | polypeptide N-acetylgalactosaminyltransferase activity | Molecular function | 2 | 19 | 268 | 18352 | 7.20816967792616 | 0.0308524751987479 | 0.484346688963476 | 1.51070998812876 | 0.00746268656716418 | GALNT1//GALNT7// |
| GO:0030676 | Rac guanyl-nucleotide exchange factor activity | Molecular function | 2 | 19 | 268 | 18352 | 7.20816967792616 | 0.0308524751987479 | 0.484346688963476 | 1.51070998812876 | 0.00746268656716418 | DOCK2//TIAM2// |
| GO:0003712 | transcription coregulator activity | Molecular function | 13 | 498 | 268 | 18352 | 1.78756818318048 | 0.0317997462734137 | 0.490187591305576 | 1.4975763451884 | 0.0485074626865672 | DDX17//DYRK1A//CCAR1//TADA2A//NRIP1//TRIM24//SIRT6//NCOR2//RNF4//TMF1//TAF15//TRRAP//LPXN// |
| GO:0008270 | zinc ion binding | Molecular function | 19 | 819 | 268 | 18352 | 1.58861370801669 | 0.0319769341987521 | 0.490187591305576 | 1.49516317674956 | 0.0708955223880597 | PITRM1//CTCF//DCTD//MORC2//SIRT5//KPNB1//MDM2//MTR//SIRT6//ZCCHC2//WDFY1//GATAD1//RNF4//PAPPA2//NR2C2//ZCCHC7//KLF7//TRIM24//ZMYM4// |
| GO:0031683 | G-protein beta/gamma-subunit complex binding | Molecular function | 2 | 20 | 268 | 18352 | 6.84776119402985 | 0.0339559981341646 | 0.508559374354212 | 1.46908349895401 | 0.00746268656716418 | GNAI1//GNAI2// |
| GO:0061608 | nuclear import signal receptor activity | Molecular function | 2 | 20 | 268 | 18352 | 6.84776119402985 | 0.0339559981341646 | 0.508559374354212 | 1.46908349895401 | 0.00746268656716418 | KPNB1//KPNA5// |
| GO:0016881 | acid-amino acid ligase activity | Molecular function | 2 | 21 | 268 | 18352 | 6.52167732764748 | 0.0371754616751247 | 0.550450301848721 | 1.42974362935035 | 0.00746268656716418 | GCLM//TTLL11// |
| GO:0017137 | Rab GTPase binding | Molecular function | 6 | 172 | 268 | 18352 | 2.38875390489413 | 0.0407970915774527 | 0.597287756465403 | 1.38937079663771 | 0.0223880597014925 | DENND5B//TBC1D1//RAB11FIP5//GDI2//ANKRD27//RPH3AL// |
| GO:0008081 | phosphoric diester hydrolase activity | Molecular function | 4 | 89 | 268 | 18352 | 3.07764548046285 | 0.0414090371511759 | 0.599510837866469 | 1.38290486765731 | 0.0149253731343284 | PDE3A//PDE4D//PLCG2//GNB1// |
| GO:0003713 | transcription coactivator activity | Molecular function | 8 | 267 | 268 | 18352 | 2.0517636536419 | 0.0428665990382333 | 0.61379157360716 | 1.36788097129503 | 0.0298507462686567 | CCAR1//RNF4//TMF1//DDX17//DYRK1A//TADA2A//NRIP1//TRIM24// |
| GO:0004114 | 3',5'-cyclic-nucleotide phosphodiesterase activity | Molecular function | 2 | 23 | 268 | 18352 | 5.9545749513303 | 0.0439462195665752 | 0.61379157360716 | 1.35707847876977 | 0.00746268656716418 | PDE3A//PDE4D// |
| GO:0019829 | ATPase-coupled cation transmembrane transporter activity | Molecular function | 3 | 54 | 268 | 18352 | 3.80431177446103 | 0.0442796683952978 | 0.61379157360716 | 1.35379564015627 | 0.0111940298507463 | ATP13A1//ATP6V0B//ATP8A1// |
| GO:0030374 | nuclear receptor transcription coactivator activity | Molecular function | 3 | 54 | 268 | 18352 | 3.80431177446103 | 0.0442796683952978 | 0.61379157360716 | 1.35379564015627 | 0.0111940298507463 | CCAR1//RNF4//TMF1// |
| GO:0016887 | ATPase activity | Molecular function | 11 | 423 | 268 | 18352 | 1.78074168166261 | 0.0470522916018745 | 0.644574370919511 | 1.32741922014718 | 0.041044776119403 | DDX11//DDX17//DDX6//DYNC1H1//MYH9//MYO19//ATP8A1//MORC2//MYO9B//ATP13A1//ABCD4// |
| GO:0030898 | actin-dependent ATPase activity | Molecular function | 2 | 24 | 268 | 18352 | 5.70646766169154 | 0.0474897464376616 | 0.644574370919511 | 1.32340014924338 | 0.00746268656716418 | MYH9//MYO19// |
| GO:0004713 | protein tyrosine kinase activity | Molecular function | 5 | 135 | 268 | 18352 | 2.53620784964069 | 0.0481558679126529 | 0.64687727721842 | 1.3173507854488 | 0.0186567164179104 | DDR1//CLK1//DYRK1A//PKDCC//EFEMP1// |
