# Supplementary material for: Differentially Expressed Circular RNAs and Their Therapeutic Mechanism in Non-segmental Vitiligo Patients Treated With Methylprednisolone
Source: Front Med (Lausanne). 2022 May 16;9:839066. doi: 10.3389/fmed.2022.839066 (PMC9149005; doi:10.3389/fmed.2022.839066)
Supplement: Supplementary file 1 [file Data_Sheet_1.ZIP › Additional files/GO Analysis Report/GO_GC_vs_control_up/BP_result(Human).html]

| GO.ID | Term | Ontology | Count | Pop.Hits | List.Total | Pop.Total | Fold.Enrichment | Pvalue | FDR | Enrichment.Score | Gene.Ratio | GENES |
| --- | --- | --- | --- | --- | --- | --- | --- | --- | --- | --- | --- | --- |
| GO:0071702 | organic substance transport | Biological process | 13 | 2800 | 36 | 18866 | 2.43311507936508 | 0.00132816433839672 | 1 | 2.87674818487525 | 0.361111111111111 | SLC38A2//RPL36//RPL7A//M6PR//SEC24A//ARL1//SLC37A3//MYH9//SLC10A7//TM7SF3//SOAT1//VAMP3//ATP8A2// |
| GO:0097502 | mannosylation | Biological process | 2 | 37 | 36 | 18866 | 28.3273273273273 | 0.00226082696142276 | 1 | 2.64573267629908 | 0.0555555555555556 | DPY19L1//PIGB// |
| GO:0006509 | membrane protein ectodomain proteolysis | Biological process | 2 | 42 | 36 | 18866 | 24.9550264550265 | 0.00290533538522023 | 1 | 2.53680372642579 | 0.0555555555555556 | MYH9//PSEN2// |
| GO:0003009 | skeletal muscle contraction | Biological process | 2 | 43 | 36 | 18866 | 24.374677002584 | 0.00304341414657721 | 1 | 2.51663894500139 | 0.0555555555555556 | ATP8A2//MYH14// |
| GO:0050856 | regulation of T cell receptor signaling pathway | Biological process | 2 | 43 | 36 | 18866 | 24.374677002584 | 0.00304341414657721 | 1 | 2.51663894500139 | 0.0555555555555556 | DUSP3//CD226// |
| GO:0050879 | multicellular organismal movement | Biological process | 2 | 55 | 36 | 18866 | 19.0565656565657 | 0.00493362989858389 | 1 | 2.30683343267996 | 0.0555555555555556 | MYH14//ATP8A2// |
| GO:0050881 | musculoskeletal movement | Biological process | 2 | 55 | 36 | 18866 | 19.0565656565657 | 0.00493362989858389 | 1 | 2.30683343267996 | 0.0555555555555556 | MYH14//ATP8A2// |
| GO:0048193 | Golgi vesicle transport | Biological process | 4 | 374 | 36 | 18866 | 5.60487225193108 | 0.00542577191647478 | 1 | 2.26553846665595 | 0.111111111111111 | SEC24A//VAMP3//ARL1//SLC10A7// |
| GO:0019538 | protein metabolic process | Biological process | 19 | 5894 | 36 | 18866 | 1.68935452248991 | 0.00579854699162995 | 1 | 2.2366808189497 | 0.527777777777778 | DUSP3//ANKIB1//RPL36//RPL7A//TRIO//STT3A//PIGB//MYH9//PSEN2//ANAPC7//MYCBP2//FBXO24//STAMBPL1//DPY19L1//TPX2//SOAT1//RCAN1//SEC24A//GRN// |
| GO:0033619 | membrane protein proteolysis | Biological process | 2 | 61 | 36 | 18866 | 17.1821493624772 | 0.00603639729837775 | 1 | 2.21922218393707 | 0.0555555555555556 | MYH9//PSEN2// |
| GO:0000956 | nuclear-transcribed mRNA catabolic process | Biological process | 3 | 210 | 36 | 18866 | 7.48650793650794 | 0.00740810976176161 | 1 | 2.13029259158338 | 0.0833333333333333 | RPL36//RPL7A//XRN2// |
| GO:0050854 | regulation of antigen receptor-mediated signaling pathway | Biological process | 2 | 69 | 36 | 18866 | 15.1900161030596 | 0.00766490706533609 | 1 | 2.11549310646602 | 0.0555555555555556 | DUSP3//CD226// |
| GO:0042982 | amyloid precursor protein metabolic process | Biological process | 2 | 71 | 36 | 18866 | 14.7621283255086 | 0.00809969969538731 | 1 | 2.09153108273294 | 0.0555555555555556 | SOAT1//PSEN2// |
| GO:1901137 | carbohydrate derivative biosynthetic process | Biological process | 5 | 674 | 36 | 18866 | 3.88765248928454 | 0.00863907163812225 | 1 | 2.06353292466267 | 0.138888888888889 | STT3A//PIGB//DPY19L1//SLC10A7//SOAT1// |
| GO:0015031 | protein transport | Biological process | 9 | 1985 | 36 | 18866 | 2.37607052896725 | 0.0105250021324709 | 1 | 1.97777780749987 | 0.25 | RPL36//RPL7A//M6PR//SEC24A//ARL1//TM7SF3//SLC10A7//VAMP3//MYH9// |
| GO:0071705 | nitrogen compound transport | Biological process | 10 | 2370 | 36 | 18866 | 2.21120487576184 | 0.0111246889956265 | 1 | 1.95371212141411 | 0.277777777777778 | SLC38A2//RPL36//RPL7A//M6PR//SEC24A//ARL1//MYH9//TM7SF3//SLC10A7//VAMP3// |
| GO:0033036 | macromolecule localization | Biological process | 12 | 3173 | 36 | 18866 | 1.98193087509192 | 0.011865769576192 | 1 | 1.92570408957011 | 0.333333333333333 | RPL36//RPL7A//M6PR//SEC24A//ARL1//SOAT1//MYH9//SLC10A7//TM7SF3//MYCBP2//VAMP3//ATP8A2// |
| GO:0015833 | peptide transport | Biological process | 9 | 2026 | 36 | 18866 | 2.32798617966436 | 0.0119635188709633 | 1 | 1.92214106102749 | 0.25 | RPL36//RPL7A//M6PR//SEC24A//ARL1//MYH9//TM7SF3//SLC10A7//VAMP3// |
| GO:0044267 | cellular protein metabolic process | Biological process | 17 | 5350 | 36 | 18866 | 1.66522326064382 | 0.0123772361676562 | 1 | 1.90737632229014 | 0.472222222222222 | DUSP3//ANKIB1//RPL36//RPL7A//TRIO//STT3A//PIGB//ANAPC7//PSEN2//MYCBP2//FBXO24//STAMBPL1//DPY19L1//TPX2//RCAN1//MYH9//GRN// |
| GO:0000902 | cell morphogenesis | Biological process | 6 | 1041 | 36 | 18866 | 3.02049311559398 | 0.0130780626593773 | 1 | 1.8834565861844 | 0.166666666666667 | ATP8A2//TRIO//MYH9//MYH14//MYCBP2//VAMP3// |
| GO:0042886 | amide transport | Biological process | 9 | 2061 | 36 | 18866 | 2.28845220766618 | 0.013306372531114 | 1 | 1.8759403220186 | 0.25 | RPL36//RPL7A//M6PR//SEC24A//ARL1//MYH9//TM7SF3//SLC10A7//VAMP3// |
| GO:0042147 | retrograde transport, endosome to Golgi | Biological process | 2 | 92 | 36 | 18866 | 11.3925120772947 | 0.0133068262897723 | 1 | 1.87592551245867 | 0.0555555555555556 | ARL1//VAMP3// |
| GO:0000904 | cell morphogenesis involved in differentiation | Biological process | 5 | 753 | 36 | 18866 | 3.47978456544194 | 0.0135145414001851 | 1 | 1.86919868697955 | 0.138888888888889 | ATP8A2//TRIO//MYCBP2//MYH9//VAMP3// |
| GO:0070085 | glycosylation | Biological process | 3 | 265 | 36 | 18866 | 5.93270440251572 | 0.0139024066406899 | 1 | 1.85691001267396 | 0.0833333333333333 | STT3A//DPY19L1//PIGB// |
| GO:0045184 | establishment of protein localization | Biological process | 9 | 2080 | 36 | 18866 | 2.26754807692308 | 0.0140817335709577 | 1 | 1.85134387687315 | 0.25 | RPL36//RPL7A//M6PR//SEC24A//ARL1//MYH9//TM7SF3//SLC10A7//VAMP3// |
| GO:1901564 | organonitrogen compound metabolic process | Biological process | 20 | 6846 | 36 | 18866 | 1.53098321809978 | 0.0141524944478572 | 1 | 1.84916700682072 | 0.555555555555556 | DUSP3//ANKIB1//RPL36//RPL7A//TRIO//STT3A//PIGB//MYH9//PSEN2//ANAPC7//MYCBP2//FBXO24//STAMBPL1//DPY19L1//SLC10A7//TPX2//SOAT1//RCAN1//SEC24A//GRN// |
| GO:0044260 | cellular macromolecule metabolic process | Biological process | 23 | 8408 | 36 | 18866 | 1.43354873665292 | 0.0152740870297424 | 1 | 1.81604473917344 | 0.638888888888889 | RPL36//RPL7A//DUSP3//ANKIB1//XRN2//VAMP3//ANXA4//TRIO//STT3A//PIGB//ANAPC7//PSEN2//MYCBP2//FBXO24//STAMBPL1//DPY19L1//TPX2//SOAT1//RCAN1//SEC24A//L3MBTL4//MYH9//GRN// |
| GO:0002474 | antigen processing and presentation of peptide antigen via MHC class I | Biological process | 2 | 99 | 36 | 18866 | 10.5869809203143 | 0.0152929070109508 | 1 | 1.81550995220614 | 0.0555555555555556 | VAMP3//SEC24A// |
| GO:0042632 | cholesterol homeostasis | Biological process | 2 | 101 | 36 | 18866 | 10.3773377337734 | 0.0158824470370447 | 1 | 1.79908258422551 | 0.0555555555555556 | SEC24A//SOAT1// |
| GO:0055092 | sterol homeostasis | Biological process | 2 | 102 | 36 | 18866 | 10.2755991285403 | 0.0161808492280823 | 1 | 1.79099868881822 | 0.0555555555555556 | SEC24A//SOAT1// |
| GO:0006614 | SRP-dependent cotranslational protein targeting to membrane | Biological process | 2 | 105 | 36 | 18866 | 9.98201058201058 | 0.0170904674279407 | 1 | 1.76724605906983 | 0.0555555555555556 | RPL36//RPL7A// |
| GO:0032940 | secretion by cell | Biological process | 7 | 1436 | 36 | 18866 | 2.55458836273599 | 0.0172668563294104 | 1 | 1.76278672456725 | 0.194444444444444 | VAMP3//SLC38A2//TM7SF3//M6PR//GRN//MYH9//SEC24A// |
| GO:0050905 | neuromuscular process | Biological process | 2 | 107 | 36 | 18866 | 9.79543094496365 | 0.0177087922108983 | 1 | 1.75181105789684 | 0.0555555555555556 | ATP8A2//MYCBP2// |
| GO:0061564 | axon development | Biological process | 4 | 530 | 36 | 18866 | 3.95513626834382 | 0.017819333448796 | 1 | 1.74910854524437 | 0.111111111111111 | ATP8A2//TRIO//MYCBP2//GRN// |
| GO:0006613 | cotranslational protein targeting to membrane | Biological process | 2 | 109 | 36 | 18866 | 9.61569826707441 | 0.0183365570377109 | 1 | 1.73668220628287 | 0.0555555555555556 | RPL36//RPL7A// |
| GO:0007041 | lysosomal transport | Biological process | 2 | 110 | 36 | 18866 | 9.52828282828283 | 0.018653955471864 | 1 | 1.7292290642595 | 0.0555555555555556 | M6PR//GRN// |
| GO:0007220 | Notch receptor processing | Biological process | 1 | 10 | 36 | 18866 | 52.4055555555556 | 0.0189233984814678 | 1 | 1.72300086533343 | 0.0277777777777778 | PSEN2// |
| GO:0030202 | heparin metabolic process | Biological process | 1 | 10 | 36 | 18866 | 52.4055555555556 | 0.0189233984814678 | 1 | 1.72300086533343 | 0.0277777777777778 | SLC10A7// |
| GO:0045713 | low-density lipoprotein particle receptor biosynthetic process | Biological process | 1 | 10 | 36 | 18866 | 52.4055555555556 | 0.0189233984814678 | 1 | 1.72300086533343 | 0.0277777777777778 | SEC24A// |
| GO:1900426 | positive regulation of defense response to bacterium | Biological process | 1 | 10 | 36 | 18866 | 52.4055555555556 | 0.0189233984814678 | 1 | 1.72300086533343 | 0.0277777777777778 | GRN// |
| GO:1901566 | organonitrogen compound biosynthetic process | Biological process | 8 | 1829 | 36 | 18866 | 2.29220581981654 | 0.0197591611576159 | 1 | 1.70423149660828 | 0.222222222222222 | RPL36//RPL7A//STT3A//PIGB//DPY19L1//SLC10A7//SOAT1//SEC24A// |
| GO:0140352 | export from cell | Biological process | 7 | 1489 | 36 | 18866 | 2.46365942840087 | 0.0206891537319149 | 1 | 1.68425727333021 | 0.194444444444444 | VAMP3//SLC38A2//TM7SF3//M6PR//GRN//MYH9//SEC24A// |
| GO:0048680 | positive regulation of axon regeneration | Biological process | 1 | 11 | 36 | 18866 | 47.6414141414141 | 0.0207964764224238 | 1 | 1.68201024195688 | 0.0277777777777778 | GRN// |
| GO:0060368 | regulation of Fc receptor mediated stimulatory signaling pathway | Biological process | 1 | 11 | 36 | 18866 | 47.6414141414141 | 0.0207964764224238 | 1 | 1.68201024195688 | 0.0277777777777778 | CD226// |
| GO:1905245 | regulation of aspartic-type peptidase activity | Biological process | 1 | 11 | 36 | 18866 | 47.6414141414141 | 0.0207964764224238 | 1 | 1.68201024195688 | 0.0277777777777778 | GRN// |
| GO:2001138 | regulation of phospholipid transport | Biological process | 1 | 11 | 36 | 18866 | 47.6414141414141 | 0.0207964764224238 | 1 | 1.68201024195688 | 0.0277777777777778 | ATP8A2// |
| GO:2001140 | positive regulation of phospholipid transport | Biological process | 1 | 11 | 36 | 18866 | 47.6414141414141 | 0.0207964764224238 | 1 | 1.68201024195688 | 0.0277777777777778 | ATP8A2// |
| GO:0000184 | nuclear-transcribed mRNA catabolic process, nonsense-mediated decay | Biological process | 2 | 120 | 36 | 18866 | 8.73425925925926 | 0.0219545963442981 | 1 | 1.65847454339473 | 0.0555555555555556 | RPL36//RPL7A// |
| GO:0045047 | protein targeting to ER | Biological process | 2 | 120 | 36 | 18866 | 8.73425925925926 | 0.0219545963442981 | 1 | 1.65847454339473 | 0.0555555555555556 | RPL36//RPL7A// |
| GO:0001768 | establishment of T cell polarity | Biological process | 1 | 12 | 36 | 18866 | 43.6712962962963 | 0.0226660774220941 | 1 | 1.64462363211604 | 0.0277777777777778 | MYH9// |
| GO:0001778 | plasma membrane repair | Biological process | 1 | 12 | 36 | 18866 | 43.6712962962963 | 0.0226660774220941 | 1 | 1.64462363211604 | 0.0277777777777778 | MYH9// |
| GO:0036444 | calcium import into the mitochondrion | Biological process | 1 | 12 | 36 | 18866 | 43.6712962962963 | 0.0226660774220941 | 1 | 1.64462363211604 | 0.0277777777777778 | PSEN2// |
| GO:0044090 | positive regulation of vacuole organization | Biological process | 1 | 12 | 36 | 18866 | 43.6712962962963 | 0.0226660774220941 | 1 | 1.64462363211604 | 0.0277777777777778 | GRN// |
| GO:0070572 | positive regulation of neuron projection regeneration | Biological process | 1 | 12 | 36 | 18866 | 43.6712962962963 | 0.0226660774220941 | 1 | 1.64462363211604 | 0.0277777777777778 | GRN// |
| GO:1903332 | regulation of protein folding | Biological process | 1 | 12 | 36 | 18866 | 43.6712962962963 | 0.0226660774220941 | 1 | 1.64462363211604 | 0.0277777777777778 | GRN// |
| GO:0006810 | transport | Biological process | 16 | 5224 | 36 | 18866 | 1.60507061425898 | 0.022938697461102 | 1 | 1.63943124648342 | 0.444444444444444 | SLC38A2//RPL36//RPL7A//M6PR//SLC10A7//PSEN2//SEC24A//ARL1//VAMP3//MYH9//GRN//SLC37A3//TM7SF3//SOAT1//UNC79//ATP8A2// |
| GO:0072599 | establishment of protein localization to endoplasmic reticulum | Biological process | 2 | 124 | 36 | 18866 | 8.45250896057348 | 0.023338017759922 | 1 | 1.63193603400129 | 0.0555555555555556 | RPL36//RPL7A// |
| GO:0000212 | meiotic spindle organization | Biological process | 1 | 13 | 36 | 18866 | 40.3119658119658 | 0.024532207750556 | 1 | 1.61026336615404 | 0.0277777777777778 | MYH9// |
| GO:0001767 | establishment of lymphocyte polarity | Biological process | 1 | 13 | 36 | 18866 | 40.3119658119658 | 0.024532207750556 | 1 | 1.61026336615404 | 0.0277777777777778 | MYH9// |
| GO:0002679 | respiratory burst involved in defense response | Biological process | 1 | 13 | 36 | 18866 | 40.3119658119658 | 0.024532207750556 | 1 | 1.61026336615404 | 0.0277777777777778 | GRN// |
| GO:0002836 | positive regulation of response to tumor cell | Biological process | 1 | 13 | 36 | 18866 | 40.3119658119658 | 0.024532207750556 | 1 | 1.61026336615404 | 0.0277777777777778 | CD226// |
| GO:0002839 | positive regulation of immune response to tumor cell | Biological process | 1 | 13 | 36 | 18866 | 40.3119658119658 | 0.024532207750556 | 1 | 1.61026336615404 | 0.0277777777777778 | CD226// |
| GO:0015697 | quaternary ammonium group transport | Biological process | 1 | 13 | 36 | 18866 | 40.3119658119658 | 0.024532207750556 | 1 | 1.61026336615404 | 0.0277777777777778 | SLC38A2// |
| GO:0034379 | very-low-density lipoprotein particle assembly | Biological process | 1 | 13 | 36 | 18866 | 40.3119658119658 | 0.024532207750556 | 1 | 1.61026336615404 | 0.0277777777777778 | SOAT1// |
| GO:0045842 | positive regulation of mitotic metaphase/anaphase transition | Biological process | 1 | 13 | 36 | 18866 | 40.3119658119658 | 0.024532207750556 | 1 | 1.61026336615404 | 0.0277777777777778 | ANAPC7// |
| GO:1901970 | positive regulation of mitotic sister chromatid separation | Biological process | 1 | 13 | 36 | 18866 | 40.3119658119658 | 0.024532207750556 | 1 | 1.61026336615404 | 0.0277777777777778 | ANAPC7// |
| GO:1902563 | regulation of neutrophil activation | Biological process | 1 | 13 | 36 | 18866 | 40.3119658119658 | 0.024532207750556 | 1 | 1.61026336615404 | 0.0277777777777778 | GRN// |
| GO:0001921 | positive regulation of receptor recycling | Biological process | 1 | 14 | 36 | 18866 | 37.4325396825397 | 0.0263948736669072 | 1 | 1.57848041231036 | 0.0277777777777778 | VAMP3// |
| GO:0035751 | regulation of lysosomal lumen pH | Biological process | 1 | 14 | 36 | 18866 | 37.4325396825397 | 0.0263948736669072 | 1 | 1.57848041231036 | 0.0277777777777778 | GRN// |
| GO:1902101 | positive regulation of metaphase/anaphase transition of cell cycle | Biological process | 1 | 14 | 36 | 18866 | 37.4325396825397 | 0.0263948736669072 | 1 | 1.57848041231036 | 0.0277777777777778 | ANAPC7// |
| GO:0009101 | glycoprotein biosynthetic process | Biological process | 3 | 342 | 36 | 18866 | 4.59697855750487 | 0.0271216381890402 | 1 | 1.56668408195197 | 0.0833333333333333 | STT3A//DPY19L1//SOAT1// |
| GO:0090150 | establishment of protein localization to membrane | Biological process | 3 | 342 | 36 | 18866 | 4.59697855750487 | 0.0271216381890402 | 1 | 1.56668408195197 | 0.0833333333333333 | RPL36//RPL7A//VAMP3// |
| GO:0046903 | secretion | Biological process | 7 | 1575 | 36 | 18866 | 2.32913580246914 | 0.0272277979564642 | 1 | 1.56498748073228 | 0.194444444444444 | VAMP3//SLC38A2//TM7SF3//M6PR//GRN//MYH9//SEC24A// |
| GO:0002281 | macrophage activation involved in immune response | Biological process | 1 | 15 | 36 | 18866 | 34.937037037037 | 0.0282540814192942 | 1 | 1.54891880767299 | 0.0277777777777778 | GRN// |
| GO:0009404 | toxin metabolic process | Biological process | 1 | 15 | 36 | 18866 | 34.937037037037 | 0.0282540814192942 | 1 | 1.54891880767299 | 0.0277777777777778 | ARL1// |
| GO:0032328 | alanine transport | Biological process | 1 | 15 | 36 | 18866 | 34.937037037037 | 0.0282540814192942 | 1 | 1.54891880767299 | 0.0277777777777778 | SLC38A2// |
| GO:0050862 | positive regulation of T cell receptor signaling pathway | Biological process | 1 | 15 | 36 | 18866 | 34.937037037037 | 0.0282540814192942 | 1 | 1.54891880767299 | 0.0277777777777778 | CD226// |
| GO:0070885 | negative regulation of calcineurin-NFAT signaling cascade | Biological process | 1 | 15 | 36 | 18866 | 34.937037037037 | 0.0282540814192942 | 1 | 1.54891880767299 | 0.0277777777777778 | RCAN1// |
| GO:0090110 | COPII-coated vesicle cargo loading | Biological process | 1 | 15 | 36 | 18866 | 34.937037037037 | 0.0282540814192942 | 1 | 1.54891880767299 | 0.0277777777777778 | SEC24A// |
| GO:0090594 | inflammatory response to wounding | Biological process | 1 | 15 | 36 | 18866 | 34.937037037037 | 0.0282540814192942 | 1 | 1.54891880767299 | 0.0277777777777778 | GRN// |
| GO:0106057 | negative regulation of calcineurin-mediated signaling | Biological process | 1 | 15 | 36 | 18866 | 34.937037037037 | 0.0282540814192942 | 1 | 1.54891880767299 | 0.0277777777777778 | RCAN1// |
| GO:1905820 | positive regulation of chromosome separation | Biological process | 1 | 15 | 36 | 18866 | 34.937037037037 | 0.0282540814192942 | 1 | 1.54891880767299 | 0.0277777777777778 | ANAPC7// |
| GO:0051234 | establishment of localization | Biological process | 16 | 5352 | 36 | 18866 | 1.56668327520345 | 0.0286398129813935 | 1 | 1.54302982230741 | 0.444444444444444 | SLC38A2//RPL36//RPL7A//M6PR//SLC10A7//PSEN2//SEC24A//ARL1//VAMP3//MYH9//GRN//SLC37A3//TM7SF3//SOAT1//UNC79//ATP8A2// |
| GO:0002834 | regulation of response to tumor cell | Biological process | 1 | 16 | 36 | 18866 | 32.7534722222222 | 0.0301098372449218 | 1 | 1.52129159200711 | 0.0277777777777778 | CD226// |
| GO:0002837 | regulation of immune response to tumor cell | Biological process | 1 | 16 | 36 | 18866 | 32.7534722222222 | 0.0301098372449218 | 1 | 1.52129159200711 | 0.0277777777777778 | CD226// |
| GO:0050884 | neuromuscular process controlling posture | Biological process | 1 | 16 | 36 | 18866 | 32.7534722222222 | 0.0301098372449218 | 1 | 1.52129159200711 | 0.0277777777777778 | ATP8A2// |
| GO:0051014 | actin filament severing | Biological process | 1 | 16 | 36 | 18866 | 32.7534722222222 | 0.0301098372449218 | 1 | 1.52129159200711 | 0.0277777777777778 | MYH9// |
| GO:1903729 | regulation of plasma membrane organization | Biological process | 1 | 16 | 36 | 18866 | 32.7534722222222 | 0.0301098372449218 | 1 | 1.52129159200711 | 0.0277777777777778 | MYH9// |
| GO:0008104 | protein localization | Biological process | 10 | 2788 | 36 | 18866 | 1.87968276741591 | 0.0319288972421775 | 1 | 1.49581608083129 | 0.277777777777778 | RPL36//RPL7A//M6PR//SEC24A//ARL1//MYH9//TM7SF3//MYCBP2//SLC10A7//VAMP3// |
| GO:0016254 | preassembly of GPI anchor in ER membrane | Biological process | 1 | 17 | 36 | 18866 | 30.8267973856209 | 0.0319621473700773 | 1 | 1.49536405039539 | 0.0277777777777778 | PIGB// |
| GO:0060263 | regulation of respiratory burst | Biological process | 1 | 17 | 36 | 18866 | 30.8267973856209 | 0.0319621473700773 | 1 | 1.49536405039539 | 0.0277777777777778 | GRN// |
| GO:0061024 | membrane organization | Biological process | 5 | 944 | 36 | 18866 | 2.77571798493409 | 0.0323084335815654 | 1 | 1.49068409748757 | 0.138888888888889 | MYH9//VAMP3//ATP8A2//SEC24A//M6PR// |
| GO:0045862 | positive regulation of proteolysis | Biological process | 3 | 370 | 36 | 18866 | 4.2490990990991 | 0.0331483950632714 | 1 | 1.4795374938667 | 0.0833333333333333 | ANKIB1//MYH9//GRN// |
| GO:0007034 | vacuolar transport | Biological process | 2 | 150 | 36 | 18866 | 6.98740740740741 | 0.033162580978934 | 1 | 1.47935167651474 | 0.0555555555555556 | M6PR//GRN// |
| GO:0035264 | multicellular organism growth | Biological process | 2 | 150 | 36 | 18866 | 6.98740740740741 | 0.033162580978934 | 1 | 1.47935167651474 | 0.0555555555555556 | ATP8A2//UNC79// |
| GO:0060041 | retina development in camera-type eye | Biological process | 2 | 150 | 36 | 18866 | 6.98740740740741 | 0.033162580978934 | 1 | 1.47935167651474 | 0.0555555555555556 | ATP8A2//XRN2// |
| GO:0031293 | membrane protein intracellular domain proteolysis | Biological process | 1 | 18 | 36 | 18866 | 29.1141975308642 | 0.0338110180101473 | 1 | 1.47094175293924 | 0.0277777777777778 | PSEN2// |
| GO:0034433 | steroid esterification | Biological process | 1 | 18 | 36 | 18866 | 29.1141975308642 | 0.0338110180101473 | 1 | 1.47094175293924 | 0.0277777777777778 | SOAT1// |
| GO:0034434 | sterol esterification | Biological process | 1 | 18 | 36 | 18866 | 29.1141975308642 | 0.0338110180101473 | 1 | 1.47094175293924 | 0.0277777777777778 | SOAT1// |
| GO:0034435 | cholesterol esterification | Biological process | 1 | 18 | 36 | 18866 | 29.1141975308642 | 0.0338110180101473 | 1 | 1.47094175293924 | 0.0277777777777778 | SOAT1// |
| GO:0042983 | amyloid precursor protein biosynthetic process | Biological process | 1 | 18 | 36 | 18866 | 29.1141975308642 | 0.0338110180101473 | 1 | 1.47094175293924 | 0.0277777777777778 | SOAT1// |
| GO:0042984 | regulation of amyloid precursor protein biosynthetic process | Biological process | 1 | 18 | 36 | 18866 | 29.1141975308642 | 0.0338110180101473 | 1 | 1.47094175293924 | 0.0277777777777778 | SOAT1// |
| GO:0043031 | negative regulation of macrophage activation | Biological process | 1 | 18 | 36 | 18866 | 29.1141975308642 | 0.0338110180101473 | 1 | 1.47094175293924 | 0.0277777777777778 | GRN// |
| GO:0070972 | protein localization to endoplasmic reticulum | Biological process | 2 | 152 | 36 | 18866 | 6.89546783625731 | 0.0339754245010607 | 1 | 1.46883510833155 | 0.0555555555555556 | RPL36//RPL7A// |
| GO:0051640 | organelle localization | Biological process | 4 | 649 | 36 | 18866 | 3.22992638246876 | 0.0342236520485411 | 1 | 1.46567364821688 | 0.111111111111111 | VAMP3//MYH9//SEC24A//PSEN2// |
| GO:0030048 | actin filament-based movement | Biological process | 2 | 153 | 36 | 18866 | 6.85039941902687 | 0.03438477993988 | 1 | 1.46363375076653 | 0.0555555555555556 | MYH9//MYH14// |
| GO:0006402 | mRNA catabolic process | Biological process | 3 | 376 | 36 | 18866 | 4.18129432624113 | 0.0345241496237723 | 1 | 1.46187700987465 | 0.0833333333333333 | RPL36//RPL7A//XRN2// |
| GO:0008360 | regulation of cell shape | Biological process | 2 | 156 | 36 | 18866 | 6.71866096866097 | 0.0356244792649587 | 1 | 1.44825147516611 | 0.0555555555555556 | MYH9//MYH14// |
| GO:0050908 | detection of light stimulus involved in visual perception | Biological process | 1 | 19 | 36 | 18866 | 27.5818713450292 | 0.0356564553696355 | 1 | 1.44786183249892 | 0.0277777777777778 | ATP8A2// |
| GO:0050962 | detection of light stimulus involved in sensory perception | Biological process | 1 | 19 | 36 | 18866 | 27.5818713450292 | 0.0356564553696355 | 1 | 1.44786183249892 | 0.0277777777777778 | ATP8A2// |
| GO:0071625 | vocalization behavior | Biological process | 1 | 19 | 36 | 18866 | 27.5818713450292 | 0.0356564553696355 | 1 | 1.44786183249892 | 0.0277777777777778 | MYH14// |
| GO:0150079 | negative regulation of neuroinflammatory response | Biological process | 1 | 19 | 36 | 18866 | 27.5818713450292 | 0.0356564553696355 | 1 | 1.44786183249892 | 0.0277777777777778 | GRN// |
| GO:0150146 | cell junction disassembly | Biological process | 1 | 19 | 36 | 18866 | 27.5818713450292 | 0.0356564553696355 | 1 | 1.44786183249892 | 0.0277777777777778 | DUSP3// |
| GO:1900424 | regulation of defense response to bacterium | Biological process | 1 | 19 | 36 | 18866 | 27.5818713450292 | 0.0356564553696355 | 1 | 1.44786183249892 | 0.0277777777777778 | GRN// |
| GO:0002418 | immune response to tumor cell | Biological process | 1 | 20 | 36 | 18866 | 26.2027777777778 | 0.0374984656421824 | 1 | 1.42598650231938 | 0.0277777777777778 | CD226// |
| GO:0010878 | cholesterol storage | Biological process | 1 | 20 | 36 | 18866 | 26.2027777777778 | 0.0374984656421824 | 1 | 1.42598650231938 | 0.0277777777777778 | SOAT1// |
| GO:0035493 | SNARE complex assembly | Biological process | 1 | 20 | 36 | 18866 | 26.2027777777778 | 0.0374984656421824 | 1 | 1.42598650231938 | 0.0277777777777778 | VAMP3// |
| GO:0071243 | cellular response to arsenic-containing substance | Biological process | 1 | 20 | 36 | 18866 | 26.2027777777778 | 0.0374984656421824 | 1 | 1.42598650231938 | 0.0277777777777778 | SLC38A2// |
| GO:0006869 | lipid transport | Biological process | 3 | 393 | 36 | 18866 | 4.00042408821035 | 0.0385825177429129 | 1 | 1.41360943542266 | 0.0833333333333333 | SLC10A7//SOAT1//ATP8A2// |
| GO:0055088 | lipid homeostasis | Biological process | 2 | 163 | 36 | 18866 | 6.43012951601909 | 0.0385838924149497 | 1 | 1.41359396204561 | 0.0555555555555556 | SEC24A//SOAT1// |
| GO:0043170 | macromolecule metabolic process | Biological process | 26 | 10650 | 36 | 18866 | 1.27938445487741 | 0.0386573092177397 | 1 | 1.41276837877506 | 0.722222222222222 | RPL36//RPL7A//DUSP3//ANKIB1//XRN2//CD226//VAMP3//ANXA4//TRIO//STT3A//PIGB//MYH9//PSEN2//ANAPC7//SLC38A2//MYCBP2//FBXO24//STAMBPL1//DPY19L1//SLC10A7//TPX2//SOAT1//RCAN1//SEC24A//L3MBTL4//GRN// |
| GO:0030220 | platelet formation | Biological process | 1 | 21 | 36 | 18866 | 24.9550264550265 | 0.0393370550105832 | 1 | 1.40519815688562 | 0.0277777777777778 | MYH9// |
| GO:1903978 | regulation of microglial cell activation | Biological process | 1 | 21 | 36 | 18866 | 24.9550264550265 | 0.0393370550105832 | 1 | 1.40519815688562 | 0.0277777777777778 | GRN// |
| GO:0016482 | cytosolic transport | Biological process | 2 | 165 | 36 | 18866 | 6.35218855218855 | 0.0394463117728627 | 1 | 1.40399359705721 | 0.0555555555555556 | ARL1//VAMP3// |
| GO:0045931 | positive regulation of mitotic cell cycle | Biological process | 2 | 168 | 36 | 18866 | 6.23875661375661 | 0.040753754362873 | 1 | 1.38983237651975 | 0.0555555555555556 | ANAPC7//DUSP3// |
| GO:0006622 | protein targeting to lysosome | Biological process | 1 | 22 | 36 | 18866 | 23.8207070707071 | 0.0411722296468059 | 1 | 1.38539561352026 | 0.0277777777777778 | M6PR// |
| GO:0010560 | positive regulation of glycoprotein biosynthetic process | Biological process | 1 | 22 | 36 | 18866 | 23.8207070707071 | 0.0411722296468059 | 1 | 1.38539561352026 | 0.0277777777777778 | SOAT1// |
| GO:0033005 | positive regulation of mast cell activation | Biological process | 1 | 22 | 36 | 18866 | 23.8207070707071 | 0.0411722296468059 | 1 | 1.38539561352026 | 0.0277777777777778 | CD226// |
| GO:0036344 | platelet morphogenesis | Biological process | 1 | 22 | 36 | 18866 | 23.8207070707071 | 0.0411722296468059 | 1 | 1.38539561352026 | 0.0277777777777778 | MYH9// |
| GO:0062033 | positive regulation of mitotic sister chromatid segregation | Biological process | 1 | 22 | 36 | 18866 | 23.8207070707071 | 0.0411722296468059 | 1 | 1.38539561352026 | 0.0277777777777778 | ANAPC7// |
| GO:0140056 | organelle localization by membrane tethering | Biological process | 2 | 171 | 36 | 18866 | 6.12930474333983 | 0.0420775776353707 | 1 | 1.37594927003951 | 0.0555555555555556 | VAMP3//PSEN2// |
| GO:0050714 | positive regulation of protein secretion | Biological process | 2 | 172 | 36 | 18866 | 6.09366925064599 | 0.0425224544214236 | 1 | 1.3713816756888 | 0.0555555555555556 | TM7SF3//SEC24A// |
| GO:0021543 | pallium development | Biological process | 2 | 173 | 36 | 18866 | 6.05844572896596 | 0.0429691189678781 | 1 | 1.36684355093121 | 0.0555555555555556 | XRN2//SLC38A2// |
| GO:0001919 | regulation of receptor recycling | Biological process | 1 | 23 | 36 | 18866 | 22.7850241545894 | 0.0430039957120102 | 1 | 1.36649119011677 | 0.0277777777777778 | VAMP3// |
| GO:0007035 | vacuolar acidification | Biological process | 1 | 23 | 36 | 18866 | 22.7850241545894 | 0.0430039957120102 | 1 | 1.36649119011677 | 0.0277777777777778 | GRN// |
| GO:0032799 | low-density lipoprotein receptor particle metabolic process | Biological process | 1 | 23 | 36 | 18866 | 22.7850241545894 | 0.0430039957120102 | 1 | 1.36649119011677 | 0.0277777777777778 | SEC24A// |
| GO:0050860 | negative regulation of T cell receptor signaling pathway | Biological process | 1 | 23 | 36 | 18866 | 22.7850241545894 | 0.0430039957120102 | 1 | 1.36649119011677 | 0.0277777777777778 | DUSP3// |
| GO:0070584 | mitochondrion morphogenesis | Biological process | 1 | 23 | 36 | 18866 | 22.7850241545894 | 0.0430039957120102 | 1 | 1.36649119011677 | 0.0277777777777778 | MYH14// |
| GO:0006401 | RNA catabolic process | Biological process | 3 | 415 | 36 | 18866 | 3.78835341365462 | 0.0441833333734431 | 1 | 1.3547415222563 | 0.0833333333333333 | RPL36//RPL7A//XRN2// |
| GO:0009100 | glycoprotein metabolic process | Biological process | 3 | 415 | 36 | 18866 | 3.78835341365462 | 0.0441833333734431 | 1 | 1.3547415222563 | 0.0833333333333333 | STT3A//DPY19L1//SOAT1// |
| GO:0032990 | cell part morphogenesis | Biological process | 4 | 705 | 36 | 18866 | 2.97336485421592 | 0.0442465302845387 | 1 | 1.35412078004774 | 0.111111111111111 | ATP8A2//TRIO//MYCBP2//MYH14// |
| GO:0002478 | antigen processing and presentation of exogenous peptide antigen | Biological process | 2 | 177 | 36 | 18866 | 5.92153170119272 | 0.0447734947288378 | 1 | 1.3489790060808 | 0.0555555555555556 | VAMP3//SEC24A// |
| GO:0010869 | regulation of receptor biosynthetic process | Biological process | 1 | 24 | 36 | 18866 | 21.8356481481481 | 0.0448323593565668 | 1 | 1.34840840526592 | 0.0277777777777778 | SEC24A// |
| GO:0035268 | protein mannosylation | Biological process | 1 | 24 | 36 | 18866 | 21.8356481481481 | 0.0448323593565668 | 1 | 1.34840840526592 | 0.0277777777777778 | DPY19L1// |
| GO:0045954 | positive regulation of natural killer cell mediated cytotoxicity | Biological process | 1 | 24 | 36 | 18866 | 21.8356481481481 | 0.0448323593565668 | 1 | 1.34840840526592 | 0.0277777777777778 | CD226// |
| GO:0006941 | striated muscle contraction | Biological process | 2 | 178 | 36 | 18866 | 5.88826466916355 | 0.045228978252589 | 1 | 1.34458322309367 | 0.0555555555555556 | MYH14//ATP8A2// |
| GO:0019083 | viral transcription | Biological process | 2 | 178 | 36 | 18866 | 5.88826466916355 | 0.045228978252589 | 1 | 1.34458322309367 | 0.0555555555555556 | RPL36//RPL7A// |
| GO:0022406 | membrane docking | Biological process | 2 | 180 | 36 | 18866 | 5.82283950617284 | 0.0461451573796984 | 1 | 1.33587386849777 | 0.0555555555555556 | VAMP3//PSEN2// |
| GO:0007417 | central nervous system development | Biological process | 5 | 1042 | 36 | 18866 | 2.51466197483472 | 0.0463525059284492 | 1 | 1.33392678187593 | 0.138888888888889 | GRN//XRN2//MYCBP2//SLC38A2//TRIO// |
| GO:0006851 | mitochondrial calcium ion transmembrane transport | Biological process | 1 | 25 | 36 | 18866 | 20.9622222222222 | 0.0466573267200719 | 1 | 1.33108014818223 | 0.0277777777777778 | PSEN2// |
| GO:0010842 | retina layer formation | Biological process | 1 | 25 | 36 | 18866 | 20.9622222222222 | 0.0466573267200719 | 1 | 1.33108014818223 | 0.0277777777777778 | ATP8A2// |
| GO:0010954 | positive regulation of protein processing | Biological process | 1 | 25 | 36 | 18866 | 20.9622222222222 | 0.0466573267200719 | 1 | 1.33108014818223 | 0.0277777777777778 | MYH9// |
| GO:0050857 | positive regulation of antigen receptor-mediated signaling pathway | Biological process | 1 | 25 | 36 | 18866 | 20.9622222222222 | 0.0466573267200719 | 1 | 1.33108014818223 | 0.0277777777777778 | CD226// |
| GO:0007051 | spindle organization | Biological process | 2 | 183 | 36 | 18866 | 5.72738312082574 | 0.0475323383470725 | 1 | 1.32301082012034 | 0.0555555555555556 | MYH9//TPX2// |
| GO:1903034 | regulation of response to wounding | Biological process | 2 | 183 | 36 | 18866 | 5.72738312082574 | 0.0475323383470725 | 1 | 1.32301082012034 | 0.0555555555555556 | GRN//MYH9// |
| GO:0002695 | negative regulation of leukocyte activation | Biological process | 2 | 184 | 36 | 18866 | 5.69625603864734 | 0.0479981439859754 | 1 | 1.31877555579594 | 0.0555555555555556 | DUSP3//GRN// |
| GO:0019884 | antigen processing and presentation of exogenous antigen | Biological process | 2 | 185 | 36 | 18866 | 5.66546546546546 | 0.0484656426431042 | 1 | 1.31456602424508 | 0.0555555555555556 | VAMP3//SEC24A// |
| GO:0000188 | inactivation of MAPK activity | Biological process | 1 | 26 | 36 | 18866 | 20.1559829059829 | 0.0484789039313706 | 1 | 1.31444720777615 | 0.0277777777777778 | DUSP3// |
| GO:0002347 | response to tumor cell | Biological process | 1 | 26 | 36 | 18866 | 20.1559829059829 | 0.0484789039313706 | 1 | 1.31444720777615 | 0.0277777777777778 | CD226// |
| GO:0021952 | central nervous system projection neuron axonogenesis | Biological process | 1 | 26 | 36 | 18866 | 20.1559829059829 | 0.0484789039313706 | 1 | 1.31444720777615 | 0.0277777777777778 | MYCBP2// |
| GO:1903020 | positive regulation of glycoprotein metabolic process | Biological process | 1 | 26 | 36 | 18866 | 20.1559829059829 | 0.0484789039313706 | 1 | 1.31444720777615 | 0.0277777777777778 | SOAT1// |
