# Supplementary material for: Differentially Expressed Circular RNAs and Their Therapeutic Mechanism in Non-segmental Vitiligo Patients Treated With Methylprednisolone
Source: Front Med (Lausanne). 2022 May 16;9:839066. doi: 10.3389/fmed.2022.839066 (PMC9149005; doi:10.3389/fmed.2022.839066)
Supplement: Supplementary file 1 [file Data_Sheet_1.ZIP › Additional files/GO Analysis Report/GO_GC_vs_control_up/CC_result(Human).html]

| GO.ID | Term | Ontology | Count | Pop.Hits | List.Total | Pop.Total | Fold.Enrichment | Pvalue | FDR | Enrichment.Score | Gene.Ratio | GENES |
| --- | --- | --- | --- | --- | --- | --- | --- | --- | --- | --- | --- | --- |
| GO:0016020 | membrane | Cellular component | 33 | 9562 | 43 | 19559 | 1.56979662715302 | 0.000174379878083584 | 0.0841532117745142 | 3.75850363030619 | 0.767441860465116 | SEC24A//PSEN2//SLC10A7//DUSP3//MYH9//DPY19L1//GRN//M6PR//STT3A//TXNDC11//SOAT1//PIGB//CD226//ANXA4//ATP8A2//TM7SF3//SLC38A2//ENOX1//UNC79//VAMP3//TMEM71//FOCAD//TMEM181//TMEM164//RNFT2//SLC37A3//ARL1//XRN2//MYCBP2//RPL36//STAMBPL1//RPL7A//MYH14// |
| GO:0005802 | trans-Golgi network | Cellular component | 5 | 245 | 43 | 19559 | 9.2828666350261 | 0.000193233551721043 | 0.0841532117745142 | 3.71391746351989 | 0.116279069767442 | ARL1//M6PR//VAMP3//GRN//SLC10A7// |
| GO:0016460 | myosin II complex | Cellular component | 2 | 20 | 43 | 19559 | 45.4860465116279 | 0.000874741464236616 | 0.177574678927049 | 3.05812028668789 | 0.0465116279069767 | MYH9//MYH14// |
| GO:0032982 | myosin filament | Cellular component | 2 | 22 | 43 | 19559 | 41.3509513742072 | 0.00106053833207053 | 0.177574678927049 | 2.97447362973501 | 0.0465116279069767 | MYH9//MYH14// |
| GO:0032588 | trans-Golgi network membrane | Cellular component | 3 | 94 | 43 | 19559 | 14.5168233547749 | 0.00115418231210155 | 0.177574678927049 | 2.93772558555981 | 0.0697674418604651 | ARL1//M6PR//VAMP3// |
| GO:0098791 | Golgi apparatus subcompartment | Cellular component | 5 | 368 | 43 | 19559 | 6.18016936299292 | 0.00122759833470497 | 0.177574678927049 | 2.91094370938076 | 0.116279069767442 | SLC10A7//GRN//ARL1//M6PR//VAMP3// |
| GO:0031984 | organelle subcompartment | Cellular component | 5 | 392 | 43 | 19559 | 5.80179164689132 | 0.00162228110979992 | 0.177574678927049 | 2.78987388880743 | 0.116279069767442 | SLC10A7//GRN//ARL1//M6PR//VAMP3// |
| GO:0005903 | brush border | Cellular component | 3 | 106 | 43 | 19559 | 12.8734093900834 | 0.00163099590288908 | 0.177574678927049 | 2.7875471299192 | 0.0697674418604651 | MYH9//SLC38A2//MYH14// |
| GO:0042788 | polysomal ribosome | Cellular component | 2 | 32 | 43 | 19559 | 28.4287790697674 | 0.0022456522319367 | 0.214425897722661 | 2.64865749894056 | 0.0465116279069767 | RPL36//RPL7A// |
| GO:0005789 | endoplasmic reticulum membrane | Cellular component | 8 | 1102 | 43 | 19559 | 3.30207234204195 | 0.00246183579474926 | 0.214425897722661 | 2.60874091802231 | 0.186046511627907 | STT3A//SLC37A3//SEC24A//TXNDC11//PSEN2//SOAT1//SLC10A7//PIGB// |
| GO:0042175 | nuclear outer membrane-endoplasmic reticulum membrane network | Cellular component | 8 | 1124 | 43 | 19559 | 3.23744103285608 | 0.00278336337300245 | 0.220391772535012 | 2.55543009205326 | 0.186046511627907 | SEC24A//STT3A//TXNDC11//PSEN2//SOAT1//SLC10A7//PIGB//SLC37A3// |
| GO:0001772 | immunological synapse | Cellular component | 2 | 39 | 43 | 19559 | 23.3261776982707 | 0.00332235898250412 | 0.241147889480091 | 2.47855344361925 | 0.0465116279069767 | DUSP3//MYH9// |
| GO:0098862 | cluster of actin-based cell projections | Cellular component | 3 | 159 | 43 | 19559 | 8.58227292672225 | 0.00512669269781661 | 0.340276192444321 | 2.29016271408727 | 0.0697674418604651 | MYH9//SLC38A2//MYH14// |
| GO:0016021 | integral component of membrane | Cellular component | 20 | 5345 | 43 | 19559 | 1.70200361128636 | 0.00546942215180309 | 0.340276192444321 | 2.26205855475243 | 0.465116279069767 | CD226//M6PR//SLC38A2//PSEN2//SLC37A3//VAMP3//TMEM71//DPY19L1//STT3A//TXNDC11//ATP8A2//TM7SF3//FOCAD//UNC79//TMEM181//SOAT1//SLC10A7//TMEM164//RNFT2//PIGB// |
| GO:0000139 | Golgi membrane | Cellular component | 6 | 766 | 43 | 19559 | 3.56287570587164 | 0.00626606653083724 | 0.344296875503266 | 2.20300499824747 | 0.13953488372093 | ARL1//M6PR//VAMP3//SEC24A//PSEN2//SLC10A7// |
| GO:0016459 | myosin complex | Cellular component | 2 | 57 | 43 | 19559 | 15.9600163198694 | 0.00697905839091278 | 0.344296875503266 | 2.15620316809548 | 0.0465116279069767 | MYH9//MYH14// |
| GO:0022625 | cytosolic large ribosomal subunit | Cellular component | 2 | 58 | 43 | 19559 | 15.6848436246993 | 0.00721828509563191 | 0.344296875503266 | 2.14156596890791 | 0.0465116279069767 | RPL36//RPL7A// |
| GO:0030662 | coated vesicle membrane | Cellular component | 3 | 183 | 43 | 19559 | 7.45672893633244 | 0.00755543752288232 | 0.344296875503266 | 2.12174038132208 | 0.0697674418604651 | SEC24A//M6PR//VAMP3// |
| GO:0005637 | nuclear inner membrane | Cellular component | 2 | 60 | 43 | 19559 | 15.162015503876 | 0.00770777744149909 | 0.344296875503266 | 2.1130708338874 | 0.0465116279069767 | DPY19L1//PSEN2// |
| GO:0031224 | intrinsic component of membrane | Cellular component | 20 | 5511 | 43 | 19559 | 1.65073658180468 | 0.00790578359364561 | 0.344296875503266 | 2.10205507784681 | 0.465116279069767 | CD226//M6PR//SLC38A2//PSEN2//TMEM71//DPY19L1//STT3A//TXNDC11//ATP8A2//TM7SF3//FOCAD//UNC79//TMEM181//SOAT1//SLC10A7//TMEM164//RNFT2//VAMP3//PIGB//SLC37A3// |
| GO:0001725 | stress fiber | Cellular component | 2 | 68 | 43 | 19559 | 13.3782489740082 | 0.0098105180590898 | 0.382080370146759 | 2.00830805844437 | 0.0465116279069767 | MYH9//MYH14// |
| GO:0097517 | contractile actin filament bundle | Cellular component | 2 | 68 | 43 | 19559 | 13.3782489740082 | 0.0098105180590898 | 0.382080370146759 | 2.00830805844437 | 0.0465116279069767 | MYH9//MYH14// |
| GO:0005844 | polysome | Cellular component | 2 | 69 | 43 | 19559 | 13.1843613077182 | 0.0100893783161601 | 0.382080370146759 | 1.99613559314656 | 0.0465116279069767 | RPL36//RPL7A// |
| GO:0005783 | endoplasmic reticulum | Cellular component | 8 | 1430 | 43 | 19559 | 2.54467393072044 | 0.0116458499024262 | 0.384095400078345 | 1.93382881159774 | 0.186046511627907 | STT3A//SEC24A//GRN//PSEN2//PHTF2//SOAT1//SLC10A7//PIGB// |
| GO:0031594 | neuromuscular junction | Cellular component | 2 | 76 | 43 | 19559 | 11.9700122399021 | 0.0121386646902183 | 0.384095400078345 | 1.91582908505363 | 0.0465116279069767 | MYH9//PSEN2// |
| GO:0032432 | actin filament bundle | Cellular component | 2 | 76 | 43 | 19559 | 11.9700122399021 | 0.0121386646902183 | 0.384095400078345 | 1.91582908505363 | 0.0465116279069767 | MYH9//MYH14// |
| GO:0030133 | transport vesicle | Cellular component | 4 | 410 | 43 | 19559 | 4.43766307430516 | 0.0123296136217737 | 0.384095400078345 | 1.9090505328577 | 0.0930232558139535 | PSEN2//SEC24A//VAMP3//M6PR// |
| GO:0030424 | axon | Cellular component | 5 | 643 | 43 | 19559 | 3.53701761365691 | 0.0129784066062195 | 0.384095400078345 | 1.88677862376093 | 0.116279069767442 | PSEN2//MYH14//TPX2//MYCBP2//SLC38A2// |
| GO:0042641 | actomyosin | Cellular component | 2 | 79 | 43 | 19559 | 11.5154548130704 | 0.0130681652141594 | 0.384095400078345 | 1.88378538359787 | 0.0465116279069767 | MYH9//MYH14// |
| GO:0012505 | endomembrane system | Cellular component | 17 | 4576 | 43 | 19559 | 1.68982253211904 | 0.0132294626892656 | 0.384095400078345 | 1.87845779419691 | 0.395348837209302 | SEC24A//PSEN2//SLC10A7//DPY19L1//GRN//M6PR//ATP8A2//STAMBPL1//PHTF2//SOAT1//PIGB//STT3A//TXNDC11//ARL1//VAMP3//SLC37A3//ANXA4// |
| GO:0031090 | organelle membrane | Cellular component | 14 | 3571 | 43 | 19559 | 1.78326701529765 | 0.0173748836870319 | 0.488178183593703 | 1.76007809403277 | 0.325581395348837 | SEC24A//PSEN2//SLC10A7//DPY19L1//GRN//M6PR//STT3A//TXNDC11//SOAT1//PIGB//ANXA4//SLC37A3//VAMP3//ARL1// |
| GO:0005768 | endosome | Cellular component | 6 | 976 | 43 | 19559 | 2.79627335112467 | 0.0191275874573579 | 0.52062902110496 | 1.71833980362822 | 0.13953488372093 | PSEN2//GRN//M6PR//VAMP3//ATP8A2//STAMBPL1// |
| GO:0005794 | Golgi apparatus | Cellular component | 8 | 1599 | 43 | 19559 | 2.27572465348983 | 0.0216180443264363 | 0.536142042764332 | 1.66518359699943 | 0.186046511627907 | SEC24A//PSEN2//SLC10A7//GRN//ARL1//M6PR//VAMP3//ATP8A2// |
| GO:0005797 | Golgi medial cisterna | Cellular component | 1 | 10 | 43 | 19559 | 45.4860465116279 | 0.0217734963220328 | 0.536142042764332 | 1.66207182766784 | 0.0232558139534884 | SLC10A7// |
| GO:0070938 | contractile ring | Cellular component | 1 | 10 | 43 | 19559 | 45.4860465116279 | 0.0217734963220328 | 0.536142042764332 | 1.66207182766784 | 0.0232558139534884 | MYH9// |
| GO:0035253 | ciliary rootlet | Cellular component | 1 | 11 | 43 | 19559 | 41.3509513742072 | 0.0239252043203014 | 0.536142042764332 | 1.62114434467753 | 0.0232558139534884 | PSEN2// |
| GO:0022626 | cytosolic ribosome | Cellular component | 2 | 110 | 43 | 19559 | 8.27019027484144 | 0.0243627087662299 | 0.536142042764332 | 1.61327442635013 | 0.0465116279069767 | RPL36//RPL7A// |
| GO:0000151 | ubiquitin ligase complex | Cellular component | 3 | 287 | 43 | 19559 | 4.7546390081841 | 0.0250553013473874 | 0.536142042764332 | 1.6011003695042 | 0.0697674418604651 | ANAPC7//FBXO24//ANKIB1// |
| GO:0015934 | large ribosomal subunit | Cellular component | 2 | 116 | 43 | 19559 | 7.84242181234964 | 0.0268828036508851 | 0.536142042764332 | 1.57052543998528 | 0.0465116279069767 | RPL36//RPL7A// |
| GO:0030135 | coated vesicle | Cellular component | 3 | 298 | 43 | 19559 | 4.57913219915717 | 0.0275984113053724 | 0.536142042764332 | 1.55911591725248 | 0.0697674418604651 | SEC24A//VAMP3//M6PR// |
| GO:0030665 | clathrin-coated vesicle membrane | Cellular component | 2 | 118 | 43 | 19559 | 7.70949940875049 | 0.0277454273933271 | 0.536142042764332 | 1.55680858088283 | 0.0465116279069767 | M6PR//VAMP3// |
| GO:0001931 | uropod | Cellular component | 1 | 13 | 43 | 19559 | 34.9892665474061 | 0.0282147608093546 | 0.536142042764332 | 1.54952362710997 | 0.0232558139534884 | MYH9// |
| GO:0008250 | oligosaccharyltransferase complex | Cellular component | 1 | 13 | 43 | 19559 | 34.9892665474061 | 0.0282147608093546 | 0.536142042764332 | 1.54952362710997 | 0.0232558139534884 | STT3A// |
| GO:0031254 | cell trailing edge | Cellular component | 1 | 13 | 43 | 19559 | 34.9892665474061 | 0.0282147608093546 | 0.536142042764332 | 1.54952362710997 | 0.0232558139534884 | MYH9// |
| GO:0043005 | neuron projection | Cellular component | 7 | 1367 | 43 | 19559 | 2.32920501522601 | 0.0282571469921409 | 0.536142042764332 | 1.54887168920079 | 0.162790697674419 | ATP8A2//MYCBP2//SLC38A2//PSEN2//MYH14//TPX2//VAMP3// |
| GO:0031965 | nuclear membrane | Cellular component | 3 | 301 | 43 | 19559 | 4.53349300780345 | 0.0283151939921461 | 0.536142042764332 | 1.54798045860288 | 0.0697674418604651 | DPY19L1//PSEN2//ANXA4// |
| GO:0030127 | COPII vesicle coat | Cellular component | 1 | 15 | 43 | 19559 | 30.3240310077519 | 0.0324859025020816 | 0.602025980410916 | 1.48830506344596 | 0.0232558139534884 | SEC24A// |
| GO:0005680 | anaphase-promoting complex | Cellular component | 1 | 22 | 43 | 19559 | 20.6754756871036 | 0.0472909933361243 | 0.838585376883073 | 1.32522156363594 | 0.0232558139534884 | ANAPC7// |
| GO:0009986 | cell surface | Cellular component | 5 | 912 | 43 | 19559 | 2.4937525499796 | 0.0485954448603214 | 0.838585376883073 | 1.31340443783115 | 0.116279069767442 | CD226//ENOX1//ANXA4//PSEN2//VAMP3// |
| GO:0005765 | lysosomal membrane | Cellular component | 3 | 375 | 43 | 19559 | 3.63888372093023 | 0.049102014031041 | 0.838585376883073 | 1.30890069393347 | 0.0697674418604651 | GRN//M6PR//PSEN2// |
| GO:0098852 | lytic vacuole membrane | Cellular component | 3 | 375 | 43 | 19559 | 3.63888372093023 | 0.049102014031041 | 0.838585376883073 | 1.30890069393347 | 0.0697674418604651 | GRN//M6PR//PSEN2// |
