# Supplementary material for: Differentially Expressed Circular RNAs and Their Therapeutic Mechanism in Non-segmental Vitiligo Patients Treated With Methylprednisolone
Source: Front Med (Lausanne). 2022 May 16;9:839066. doi: 10.3389/fmed.2022.839066 (PMC9149005; doi:10.3389/fmed.2022.839066)
Supplement: Supplementary file 1 [file Data_Sheet_1.ZIP › Additional files/GO Analysis Report/GO_GC_vs_control_up/MF_result(Human).html]

| GO.ID | Term | Ontology | Count | Pop.Hits | List.Total | Pop.Total | Fold.Enrichment | Pvalue | FDR | Enrichment.Score | Gene.Ratio | GENES |
| --- | --- | --- | --- | --- | --- | --- | --- | --- | --- | --- | --- | --- |
| GO:0030898 | actin-dependent ATPase activity | Molecular function | 2 | 24 | 40 | 18352 | 38.2333333333333 | 0.00124025002543734 | 0.786971497956073 | 2.90649075538446 | 0.05 | MYH9//MYH14// |
| GO:0000030 | mannosyltransferase activity | Molecular function | 2 | 28 | 40 | 18352 | 32.7714285714286 | 0.00168927174827771 | 0.786971497956073 | 2.77230048110732 | 0.05 | PIGB//DPY19L1// |
| GO:0000146 | microfilament motor activity | Molecular function | 2 | 29 | 40 | 18352 | 31.6413793103448 | 0.00181190674893954 | 0.786971497956073 | 2.74186415735629 | 0.05 | MYH9//MYH14// |
| GO:0005089 | Rho guanyl-nucleotide exchange factor activity | Molecular function | 2 | 60 | 40 | 18352 | 15.2933333333333 | 0.00756986264528685 | 1 | 2.1209120006763 | 0.05 | PLEKHG1//TRIO// |
| GO:0016758 | transferase activity, transferring hexosyl groups | Molecular function | 3 | 201 | 40 | 18352 | 6.84776119402985 | 0.00949493805450252 | 1 | 2.02250786427878 | 0.075 | PIGB//DPY19L1//STT3A// |
| GO:0005085 | guanyl-nucleotide exchange factor activity | Molecular function | 3 | 215 | 40 | 18352 | 6.40186046511628 | 0.011390975726044 | 1 | 1.94343907361296 | 0.075 | PLEKHG1//TRIO//MYCBP2// |
| GO:0016757 | transferase activity, transferring glycosyl groups | Molecular function | 3 | 275 | 40 | 18352 | 5.00509090909091 | 0.0218648015135204 | 1 | 1.66025446078307 | 0.075 | PIGB//DPY19L1//STT3A// |
| GO:0015643 | toxic substance binding | Molecular function | 1 | 11 | 40 | 18352 | 41.7090909090909 | 0.0237223973040468 | 1 | 1.62484142478036 | 0.025 | TMEM181// |
| GO:0140326 | ATPase-coupled intramembrane lipid transporter activity | Molecular function | 1 | 11 | 40 | 18352 | 41.7090909090909 | 0.0237223973040468 | 1 | 1.62484142478036 | 0.025 | ATP8A2// |
| GO:0061578 | Lys63-specific deubiquitinase activity | Molecular function | 1 | 12 | 40 | 18352 | 38.2333333333333 | 0.0258515671479943 | 1 | 1.58751312440383 | 0.025 | STAMBPL1// |
| GO:0000149 | SNARE binding | Molecular function | 2 | 115 | 40 | 18352 | 7.97913043478261 | 0.0260056756281669 | 1 | 1.58493185876002 | 0.05 | VAMP3//SEC24A// |
| GO:0005088 | Ras guanyl-nucleotide exchange factor activity | Molecular function | 2 | 115 | 40 | 18352 | 7.97913043478261 | 0.0260056756281669 | 1 | 1.58493185876002 | 0.05 | PLEKHG1//TRIO// |
| GO:0004859 | phospholipase inhibitor activity | Molecular function | 1 | 13 | 40 | 18352 | 35.2923076923077 | 0.027976209313429 | 1 | 1.55321113136627 | 0.025 | ANXA4// |
| GO:0033549 | MAP kinase phosphatase activity | Molecular function | 1 | 14 | 40 | 18352 | 32.7714285714286 | 0.0300963331820949 | 1 | 1.52148641390081 | 0.025 | DUSP3// |
| GO:0005283 | amino acid:sodium symporter activity | Molecular function | 1 | 15 | 40 | 18352 | 30.5866666666667 | 0.0322119481168055 | 1 | 1.49198300908921 | 0.025 | SLC38A2// |
| GO:0003774 | motor activity | Molecular function | 2 | 132 | 40 | 18352 | 6.95151515151515 | 0.0335181216049319 | 1 | 1.47472032773481 | 0.05 | MYH9//MYH14// |
| GO:0005319 | lipid transporter activity | Molecular function | 2 | 134 | 40 | 18352 | 6.84776119402985 | 0.0344518368221716 | 1 | 1.46278761844525 | 0.05 | SLC10A7//ATP8A2// |
| GO:0070122 | isopeptidase activity | Molecular function | 1 | 17 | 40 | 18352 | 26.9882352941176 | 0.0364296885411907 | 1 | 1.43854454178088 | 0.025 | STAMBPL1// |
| GO:0055102 | lipase inhibitor activity | Molecular function | 1 | 18 | 40 | 18352 | 25.4888888888889 | 0.0385318326621799 | 1 | 1.41418033445588 | 0.025 | ANXA4// |
| GO:0015293 | symporter activity | Molecular function | 2 | 143 | 40 | 18352 | 6.41678321678322 | 0.0387776323150656 | 1 | 1.4114187116032 | 0.05 | SLC38A2//SLC10A7// |
| GO:0005178 | integrin binding | Molecular function | 2 | 144 | 40 | 18352 | 6.37222222222222 | 0.039270558919222 | 1 | 1.40593291757841 | 0.05 | CD226//MYH9// |
| GO:0005416 | amino acid:cation symporter activity | Molecular function | 1 | 19 | 40 | 18352 | 24.1473684210526 | 0.0406295051119191 | 1 | 1.39115846756952 | 0.025 | SLC38A2// |
| GO:0008409 | 5'-3' exonuclease activity | Molecular function | 1 | 19 | 40 | 18352 | 24.1473684210526 | 0.0406295051119191 | 1 | 1.39115846756952 | 0.025 | XRN2// |
| GO:0015125 | bile acid transmembrane transporter activity | Molecular function | 1 | 19 | 40 | 18352 | 24.1473684210526 | 0.0406295051119191 | 1 | 1.39115846756952 | 0.025 | SLC10A7// |
| GO:0043495 | protein-membrane adaptor activity | Molecular function | 1 | 21 | 40 | 18352 | 21.8476190476191 | 0.0448114720538296 | 1 | 1.34861078930823 | 0.025 | MYH9// |
| GO:0140303 | intramembrane lipid transporter activity | Molecular function | 1 | 21 | 40 | 18352 | 21.8476190476191 | 0.0448114720538296 | 1 | 1.34861078930823 | 0.025 | ATP8A2// |
| GO:0000062 | fatty-acyl-CoA binding | Molecular function | 1 | 22 | 40 | 18352 | 20.8545454545455 | 0.0468957850273636 | 1 | 1.32886618972882 | 0.025 | SOAT1// |
| GO:0017048 | Rho GTPase binding | Molecular function | 2 | 162 | 40 | 18352 | 5.6641975308642 | 0.048543802224839 | 1 | 1.31386621027446 | 0.05 | PLEKHG1//TRIO// |
| GO:0046943 | carboxylic acid transmembrane transporter activity | Molecular function | 2 | 162 | 40 | 18352 | 5.6641975308642 | 0.048543802224839 | 1 | 1.31386621027446 | 0.05 | SLC38A2//SLC10A7// |
| GO:0005342 | organic acid transmembrane transporter activity | Molecular function | 2 | 163 | 40 | 18352 | 5.62944785276074 | 0.0490804800721116 | 1 | 1.30909119794892 | 0.05 | SLC38A2//SLC10A7// |
