# Supplementary material for: Differentially Expressed Circular RNAs and Their Therapeutic Mechanism in Non-segmental Vitiligo Patients Treated With Methylprednisolone
Source: Front Med (Lausanne). 2022 May 16;9:839066. doi: 10.3389/fmed.2022.839066 (PMC9149005; doi:10.3389/fmed.2022.839066)
Supplement: Supplementary file 1 [file Data_Sheet_1.ZIP › Additional files/Pathway Analysis Report/Pathway_GC_vs_control_down/hsa_pathwayResult.html]

| PathwayID | Definition | OriginalWebSite | Fisher-Pvalue | SelectionCounts | SelectionSize | Count | Size | FDR | Enrichment\_Score | GeneRatio | Genes |
| --- | --- | --- | --- | --- | --- | --- | --- | --- | --- | --- | --- |
| hsa04216 | Ferroptosis - Homo sapiens (human) | http://www.genome.jp/kegg-bin/show\_pathway?hsa04216+2730+5094+6520+7037+7419 | 2.903931e-04 | 5 | 117 | 41 | 8070 | 9.786249e-02 | 3.537014 | 0.042735 | GCLM//PCBP2//SLC3A2//TFRC//VDAC3 |
| hsa04360 | Axon guidance - Homo sapiens (human) | http://www.genome.jp/kegg-bin/show\_pathway?hsa04360+659+2770+2771+2932+4773+56288+5336+6586 | 4.679050e-03 | 8 | 117 | 181 | 8070 | 4.352881e-01 | 2.329842 | 0.068376 | BMPR2//GNAI1//GNAI2//GSK3B//NFATC2//PARD3//PLCG2//SLIT3 |
| hsa00270 | Cysteine and methionine metabolism - Homo sapiens (human) | http://www.genome.jp/kegg-bin/show\_pathway?hsa00270+23382+2730+4190+4548 | 5.783516e-03 | 4 | 117 | 50 | 8070 | 4.352881e-01 | 2.237808 | 0.034188 | AHCYL2//GCLM//MDH1//MTR |
| hsa04670 | Leukocyte transendothelial migration - Homo sapiens (human) | http://www.genome.jp/kegg-bin/show\_pathway?hsa04670+60+83692+2770+2771+3383+5336 | 6.115113e-03 | 6 | 117 | 114 | 8070 | 4.352881e-01 | 2.213596 | 0.051282 | ACTB//CD99L2//GNAI1//GNAI2//ICAM1//PLCG2 |
| hsa04145 | Phagosome - Homo sapiens (human) | http://www.genome.jp/kegg-bin/show\_pathway?hsa04145+60+533+821+1778+7037+7057+10376 | 6.458280e-03 | 7 | 117 | 152 | 8070 | 4.352881e-01 | 2.189883 | 0.059829 | ACTB//ATP6V0B//CANX//DYNC1H1//TFRC//THBS1//TUBA1B |
| hsa05032 | Morphine addiction - Homo sapiens (human) | http://www.genome.jp/kegg-bin/show\_pathway?hsa05032+2770+2771+2782+5139+5144 | 1.016256e-02 | 5 | 117 | 91 | 8070 | 5.186277e-01 | 1.992997 | 0.042735 | GNAI1//GNAI2//GNB1//PDE3A//PDE4D |
| hsa04144 | Endocytosis - Homo sapiens (human) | http://www.genome.jp/kegg-bin/show\_pathway?hsa04144+116987+1785+3949+4193+23327+56288+23396+26056+7037 | 1.077268e-02 | 9 | 117 | 252 | 8070 | 5.186277e-01 | 1.967676 | 0.076923 | AGAP1//DNM2//LDLR//MDM2//NEDD4L//PARD3//PIP5K1C//RAB11FIP5//TFRC |
| hsa05135 | Yersinia infection - Homo sapiens (human) | http://www.genome.jp/kegg-bin/show\_pathway?hsa05135+60+64283+1793+2932+4773+23396 | 1.442942e-02 | 6 | 117 | 137 | 8070 | 6.078394e-01 | 1.840751 | 0.051282 | ACTB//ARHGEF28//DOCK1//GSK3B//NFATC2//PIP5K1C |
| hsa04062 | Chemokine signaling pathway - Homo sapiens (human) | http://www.genome.jp/kegg-bin/show\_pathway?hsa04062+1794+2770+2771+2782+2932+56288+5336 | 2.132842e-02 | 7 | 117 | 192 | 8070 | 7.671679e-01 | 1.671041 | 0.059829 | DOCK2//GNAI1//GNAI2//GNB1//GSK3B//PARD3//PLCG2 |
| hsa04510 | Focal adhesion - Homo sapiens (human) | http://www.genome.jp/kegg-bin/show\_pathway?hsa04510+60+1292+1793+2317+2932+23396+7057 | 2.658722e-02 | 7 | 117 | 201 | 8070 | 7.671679e-01 | 1.575327 | 0.059829 | ACTB//COL6A2//DOCK1//FLNB//GSK3B//PIP5K1C//THBS1 |
| hsa05132 | Salmonella infection - Homo sapiens (human) | http://www.genome.jp/kegg-bin/show\_pathway?hsa05132+60+1785+1778+83658+2317+2597+23207+10376 | 2.795477e-02 | 8 | 117 | 249 | 8070 | 7.671679e-01 | 1.553544 | 0.068376 | ACTB//DNM2//DYNC1H1//DYNLRB1//FLNB//GAPDH//PLEKHM2//TUBA1B |
| hsa04022 | cGMP-PKG signaling pathway - Homo sapiens (human) | http://www.genome.jp/kegg-bin/show\_pathway?hsa04022+2770+2771+9569+4773+5139+7419 | 3.418004e-02 | 6 | 117 | 167 | 8070 | 7.671679e-01 | 1.466227 | 0.051282 | GNAI1//GNAI2//GTF2IRD1//NFATC2//PDE3A//VDAC3 |
| hsa05110 | Vibrio cholerae infection - Homo sapiens (human) | http://www.genome.jp/kegg-bin/show\_pathway?hsa05110+60+533+5336 | 3.555433e-02 | 3 | 117 | 50 | 8070 | 7.671679e-01 | 1.449108 | 0.025641 | ACTB//ATP6V0B//PLCG2 |
| hsa04727 | GABAergic synapse - Homo sapiens (human) | http://www.genome.jp/kegg-bin/show\_pathway?hsa04727+11337+2770+2771+2782 | 3.998286e-02 | 4 | 117 | 89 | 8070 | 7.671679e-01 | 1.398126 | 0.034188 | GABARAP//GNAI1//GNAI2//GNB1 |
