# Supplementary material for: Differentially Expressed Circular RNAs and Their Therapeutic Mechanism in Non-segmental Vitiligo Patients Treated With Methylprednisolone
Source: Front Med (Lausanne). 2022 May 16;9:839066. doi: 10.3389/fmed.2022.839066 (PMC9149005; doi:10.3389/fmed.2022.839066)
Supplement: Supplementary file 1 [file Data_Sheet_1.ZIP › Additional files/Pathway Analysis Report/Pathway_GC_vs_control_up/hsa_pathwayResult.html]

| PathwayID | Definition | OriginalWebSite | Fisher-Pvalue | SelectionCounts | SelectionSize | Count | Size | FDR | Enrichment\_Score | GeneRatio | Genes |
| --- | --- | --- | --- | --- | --- | --- | --- | --- | --- | --- | --- |
| hsa05130 | Pathogenic Escherichia coli infection - Homo sapiens (human) | http://www.genome.jp/kegg-bin/show\_pathway?hsa05130+79784+4627+10802 | 7.567897e-03 | 3 | 17 | 197 | 8070 | 1.000000e+00 | 2.121025 | 0.176471 | MYH14//MYH9//SEC24A |
| hsa04270 | Vascular smooth muscle contraction - Homo sapiens (human) | http://www.genome.jp/kegg-bin/show\_pathway?hsa04270+79784+4627 | 3.205973e-02 | 2 | 17 | 135 | 8070 | 1.000000e+00 | 1.494040 | 0.117647 | MYH14//MYH9 |
| hsa04145 | Phagosome - Homo sapiens (human) | http://www.genome.jp/kegg-bin/show\_pathway?hsa04145+4074+9341 | 3.983582e-02 | 2 | 17 | 152 | 8070 | 1.000000e+00 | 1.399726 | 0.117647 | M6PR//VAMP3 |
| hsa03010 | Ribosome - Homo sapiens (human) | http://www.genome.jp/kegg-bin/show\_pathway?hsa03010+25873+6130 | 4.273781e-02 | 2 | 17 | 158 | 8070 | 1.000000e+00 | 1.369188 | 0.117647 | RPL36//RPL7A |
| hsa04530 | Tight junction - Homo sapiens (human) | http://www.genome.jp/kegg-bin/show\_pathway?hsa04530+79784+4627 | 4.826063e-02 | 2 | 17 | 169 | 8070 | 1.000000e+00 | 1.316407 | 0.117647 | MYH14//MYH9 |
| hsa04141 | Protein processing in endoplasmic reticulum - Homo sapiens (human) | http://www.genome.jp/kegg-bin/show\_pathway?hsa04141+10802+3703 | 4.929210e-02 | 2 | 17 | 171 | 8070 | 1.000000e+00 | 1.307223 | 0.117647 | SEC24A//STT3A |
