# Supplementary figures and images for: Differentially Expressed Circular RNAs and Their Therapeutic Mechanism in Non-segmental Vitiligo Patients Treated With Methylprednisolone (part 2 of 2)
Source: Front Med (Lausanne). 2022 May 16;9:839066. doi: 10.3389/fmed.2022.839066 (PMC9149005; doi:10.3389/fmed.2022.839066)

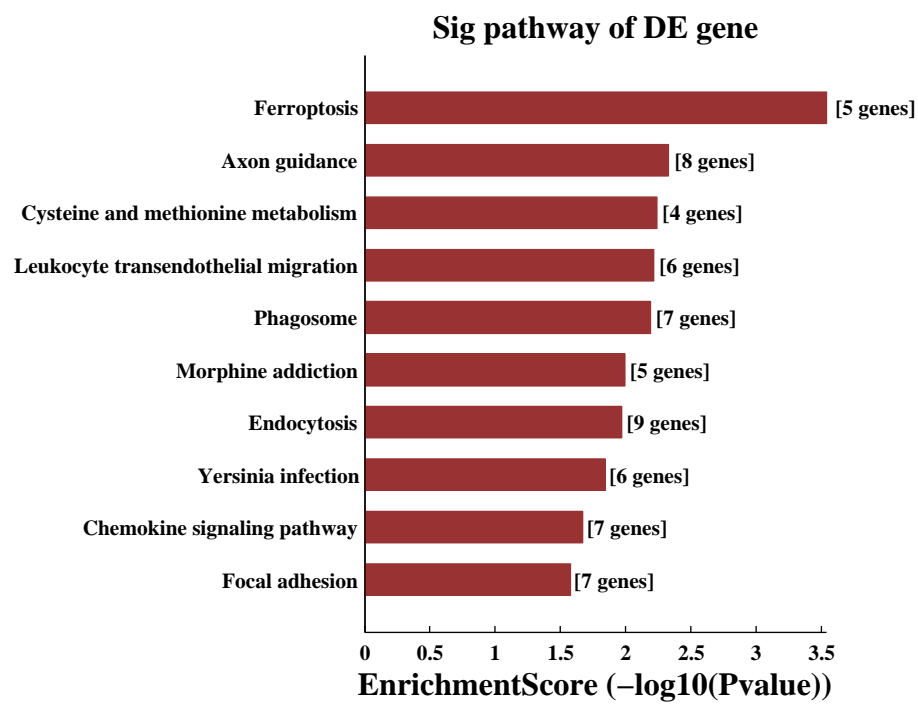

Supplement: Supplementary file 1 [file Data_Sheet_1.ZIP › Additional files/Pathway Analysis Report/Pathway_GC_vs_control_down/hsa_EnrichmentScore.pdf]

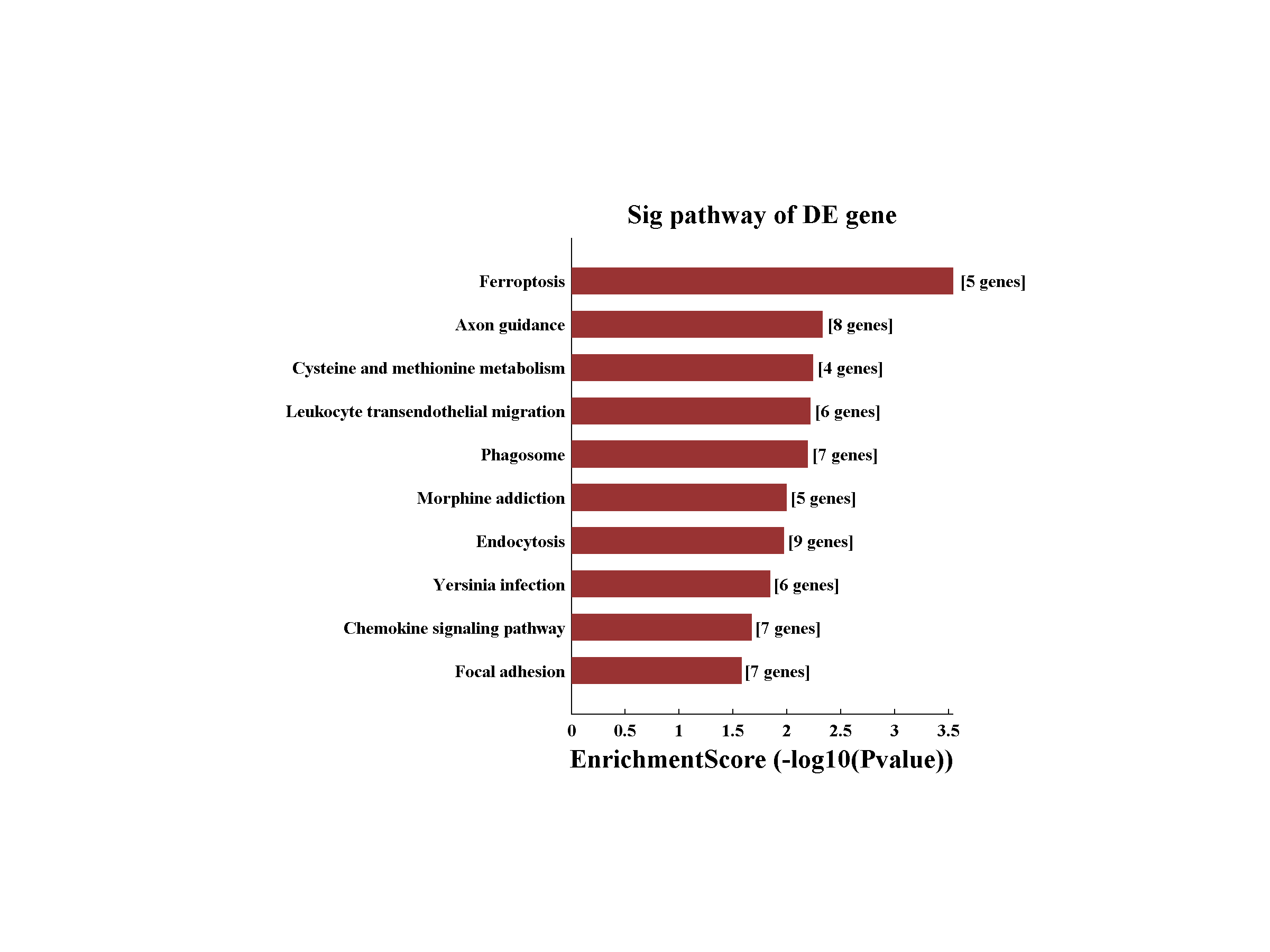

Supplement: Supplementary file 1 [file Data_Sheet_1.ZIP › Additional files/Pathway Analysis Report/Pathway_GC_vs_control_down/hsa_EnrichmentScore.png]

# Sig pathway of DE gene

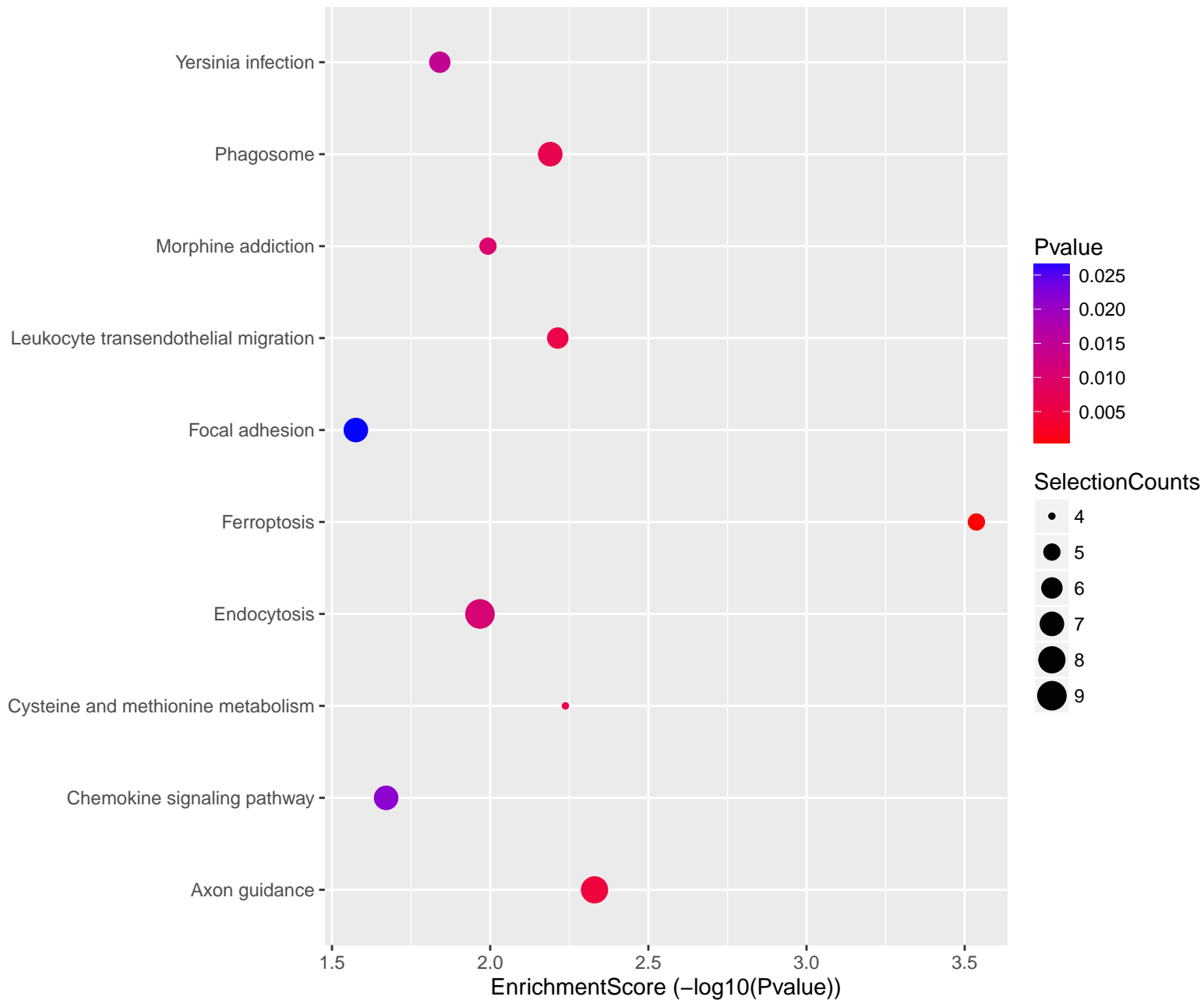

Supplement: Supplementary file 1 [file Data_Sheet_1.ZIP › Additional files/Pathway Analysis Report/Pathway_GC_vs_control_down/hsa_EnrichmentScoreDotPlot.pdf]

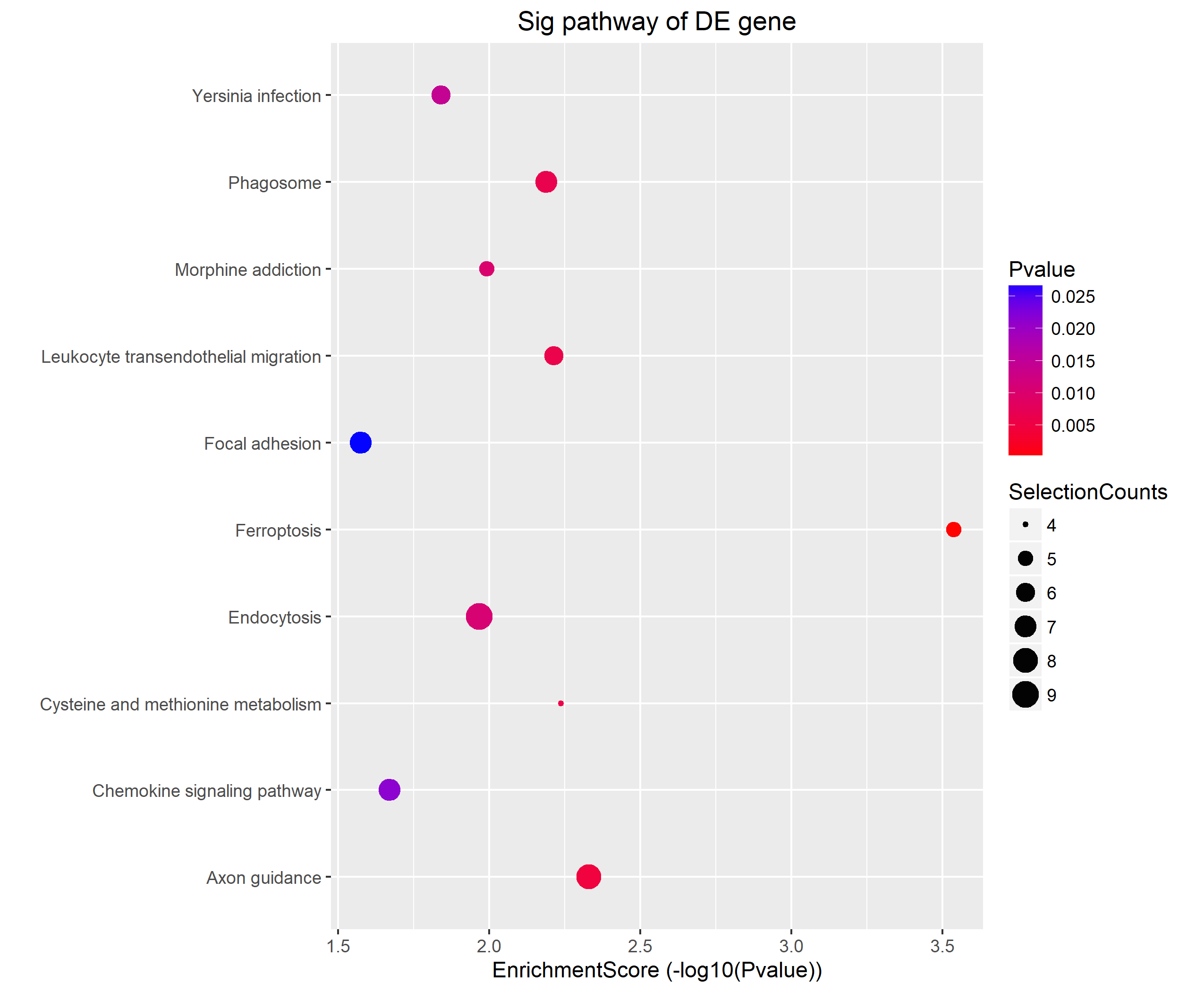

Supplement: Supplementary file 1 [file Data_Sheet_1.ZIP › Additional files/Pathway Analysis Report/Pathway_GC_vs_control_down/hsa_EnrichmentScoreDotPlot.png]

# Sig pathway of DE gene

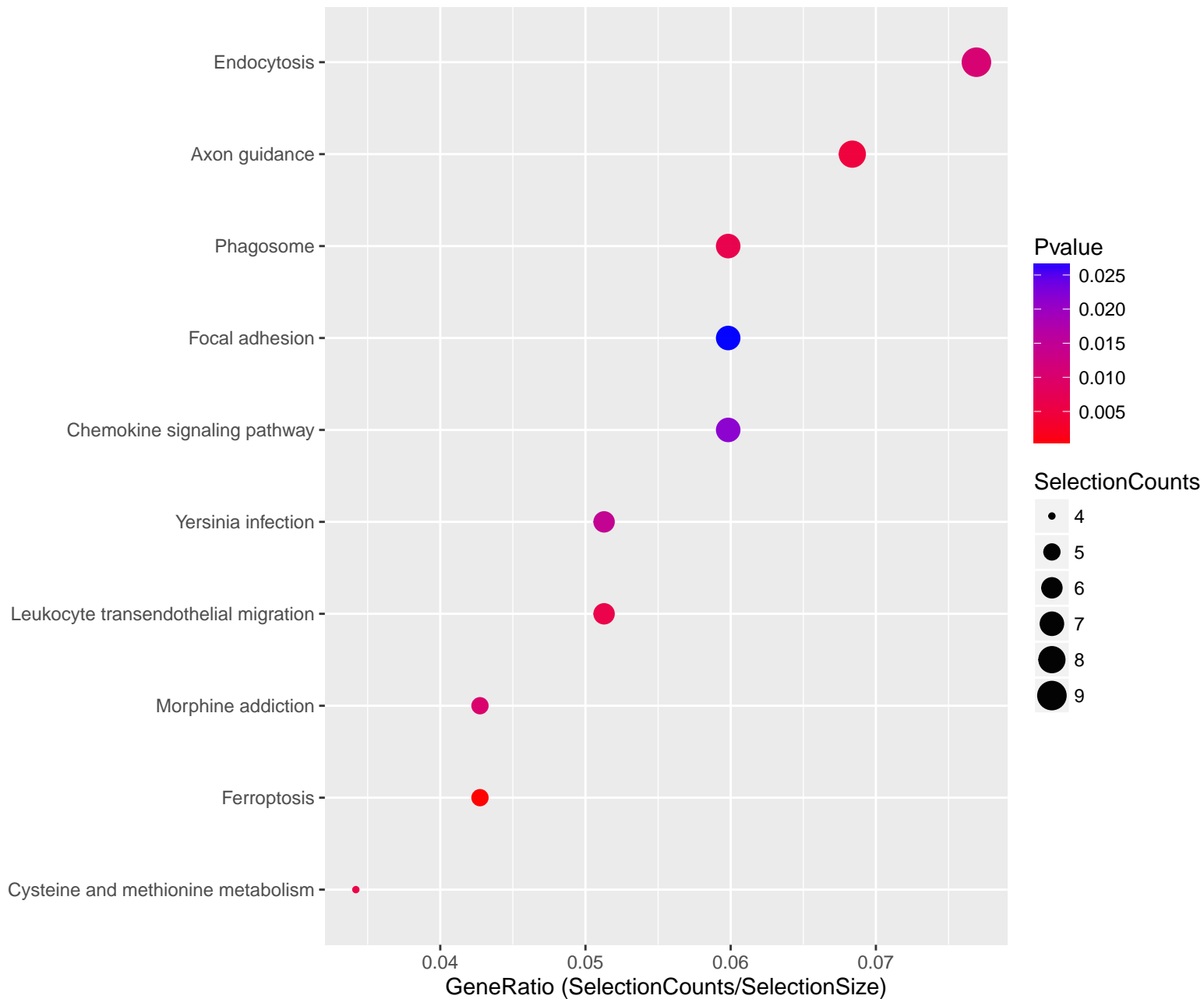

Supplement: Supplementary file 1 [file Data_Sheet_1.ZIP › Additional files/Pathway Analysis Report/Pathway_GC_vs_control_down/hsa_GeneRatioDotPlot.pdf]

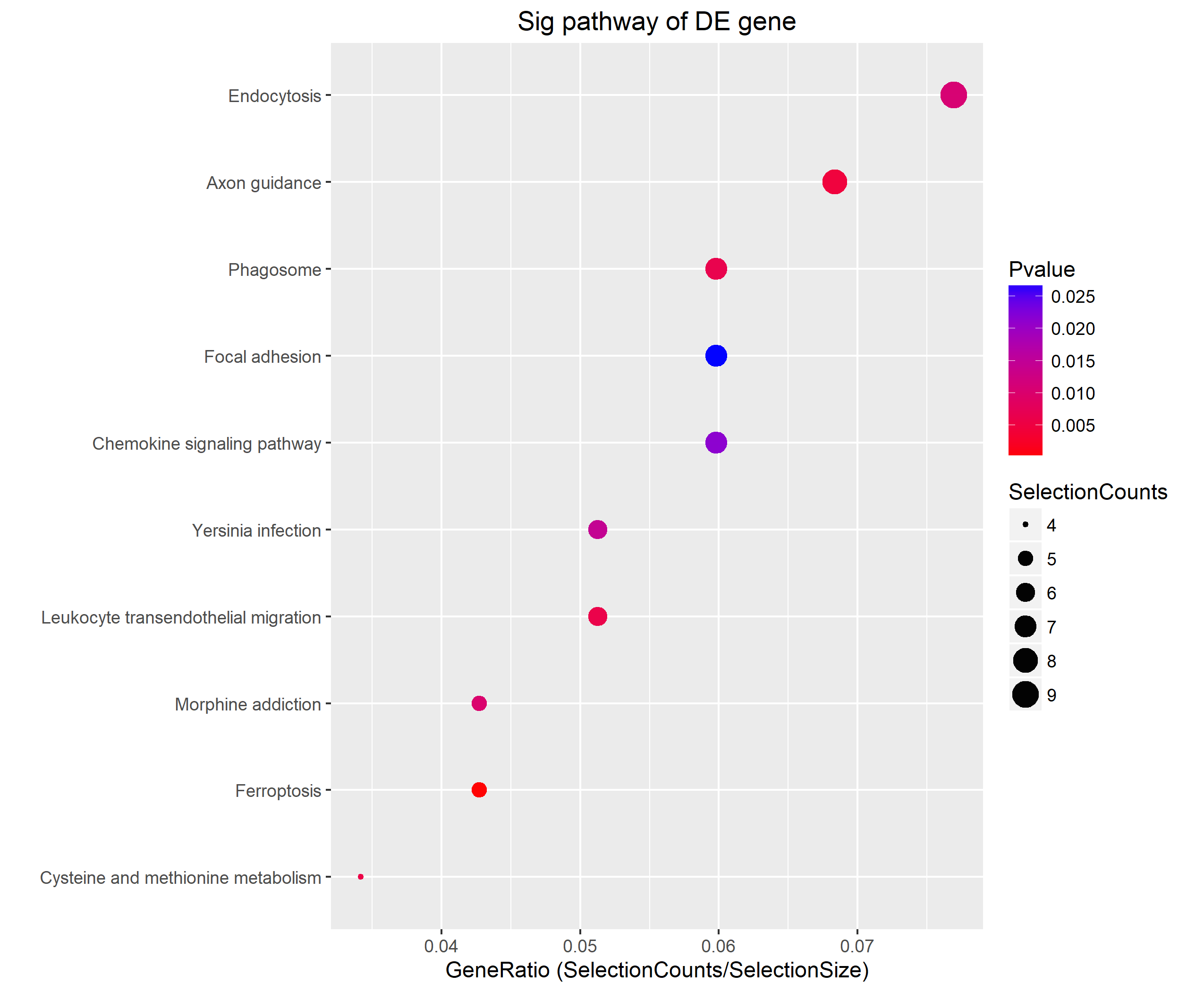

Supplement: Supplementary file 1 [file Data_Sheet_1.ZIP › Additional files/Pathway Analysis Report/Pathway_GC_vs_control_down/hsa_GeneRatioDotPlot.png]

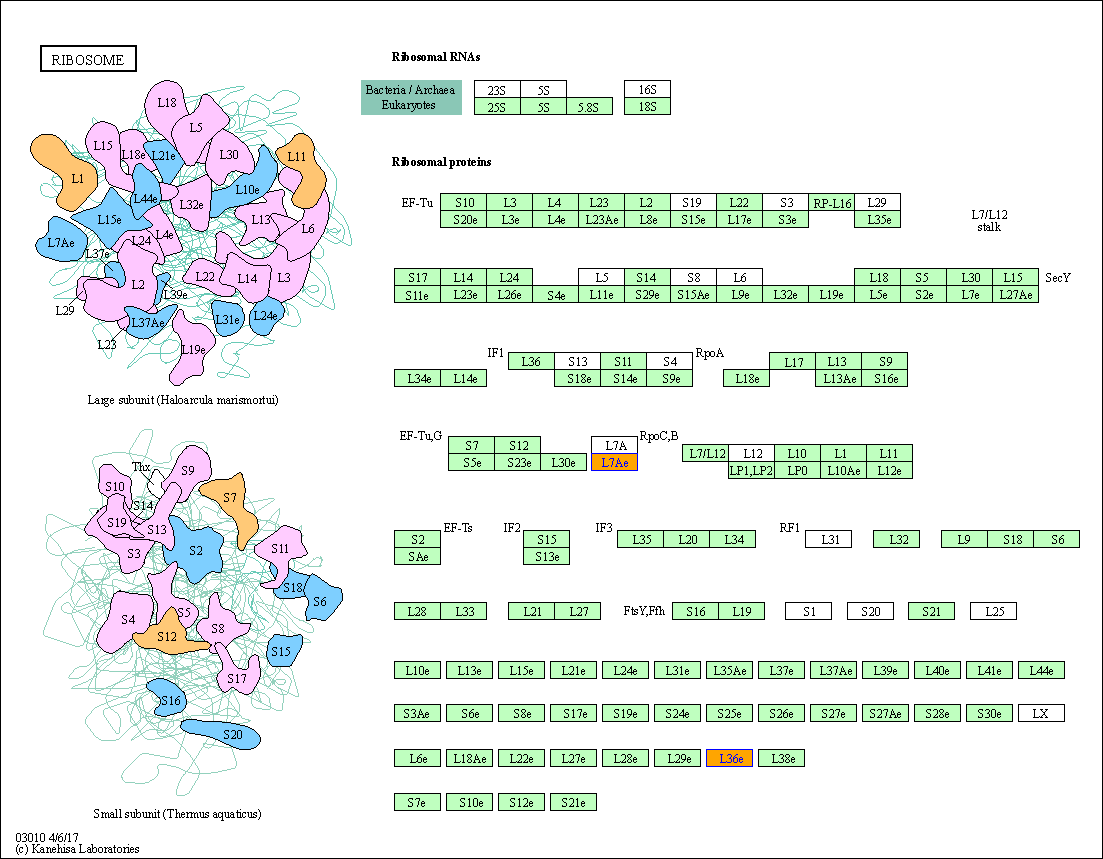

Supplement: Supplementary file 1 [file Data_Sheet_1.ZIP › Additional files/Pathway Analysis Report/Pathway_GC_vs_control_up/hsa03010.png]

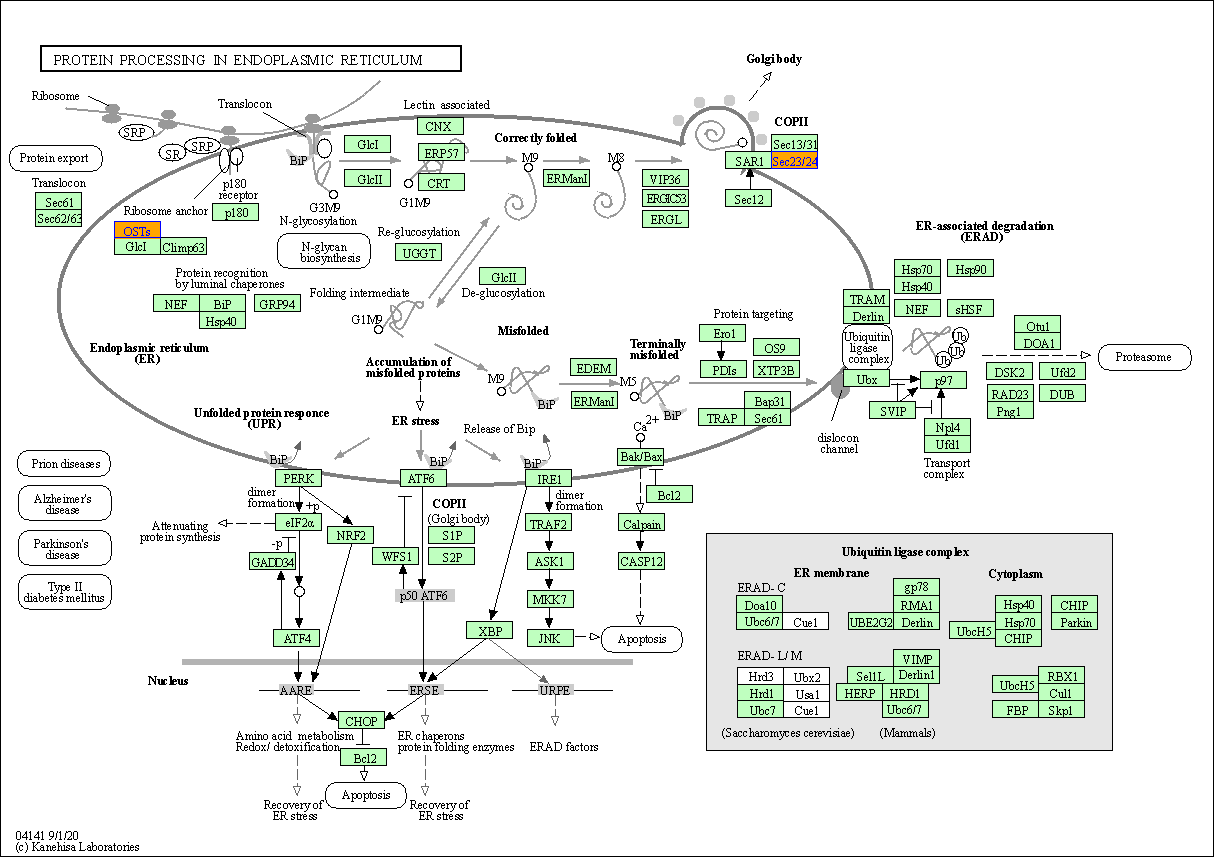

Supplement: Supplementary file 1 [file Data_Sheet_1.ZIP › Additional files/Pathway Analysis Report/Pathway_GC_vs_control_up/hsa04141.png]

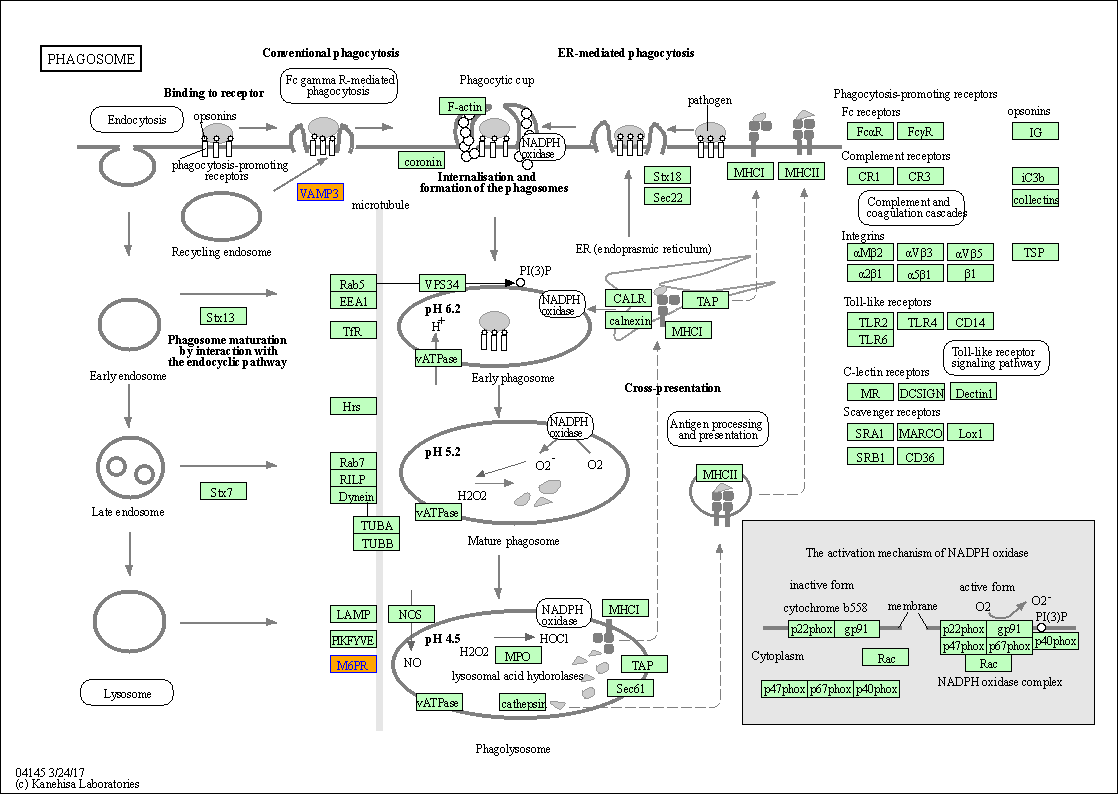

Supplement: Supplementary file 1 [file Data_Sheet_1.ZIP › Additional files/Pathway Analysis Report/Pathway_GC_vs_control_up/hsa04145.png]

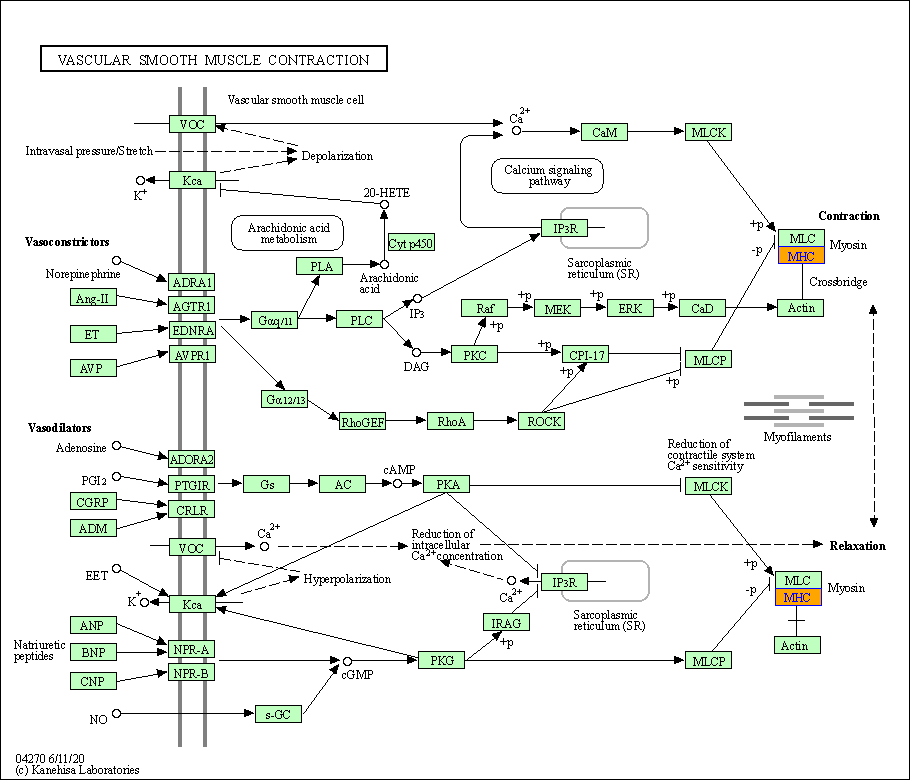

Supplement: Supplementary file 1 [file Data_Sheet_1.ZIP › Additional files/Pathway Analysis Report/Pathway_GC_vs_control_up/hsa04270.png]

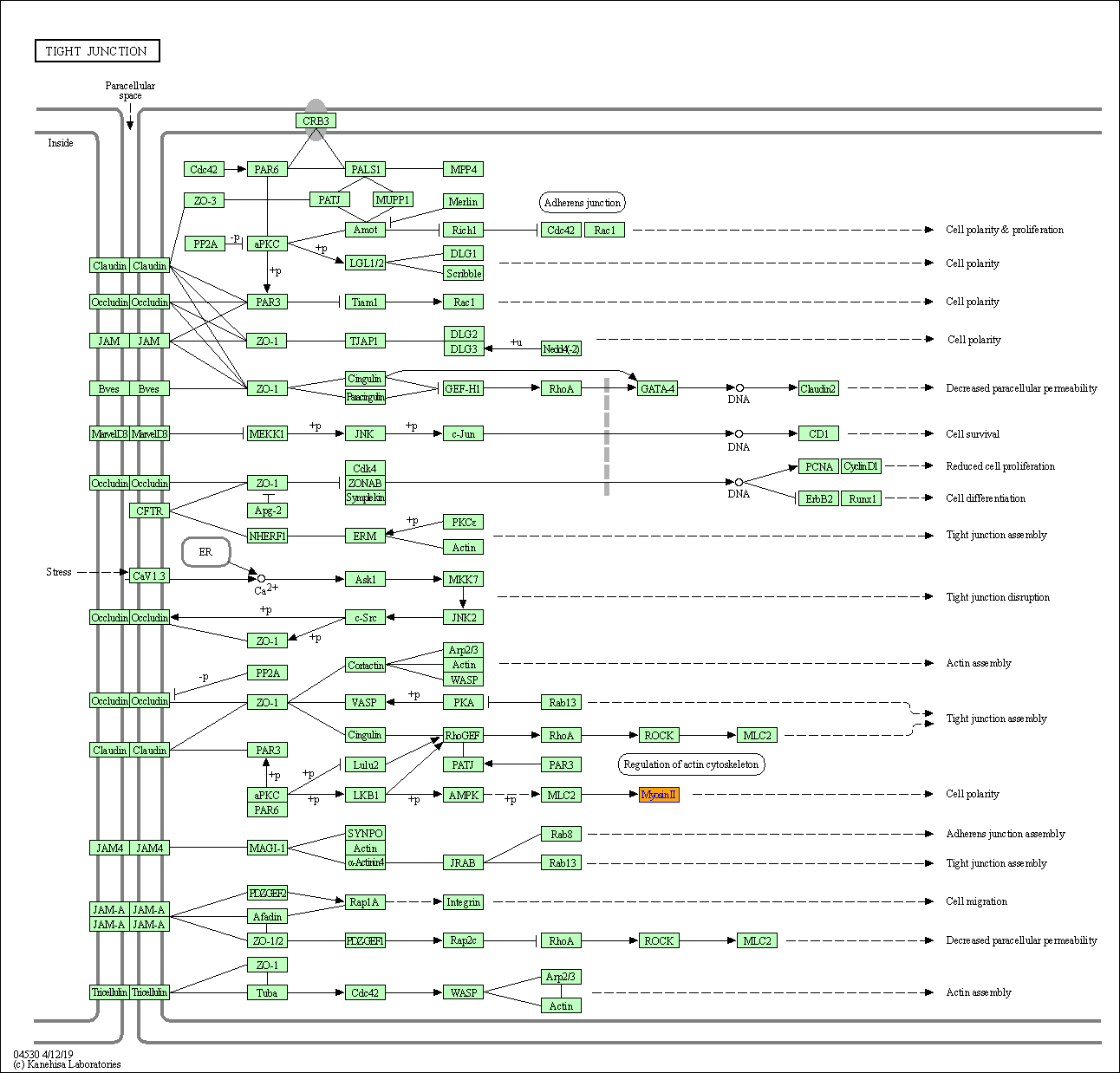

Supplement: Supplementary file 1 [file Data_Sheet_1.ZIP › Additional files/Pathway Analysis Report/Pathway_GC_vs_control_up/hsa04530.png]

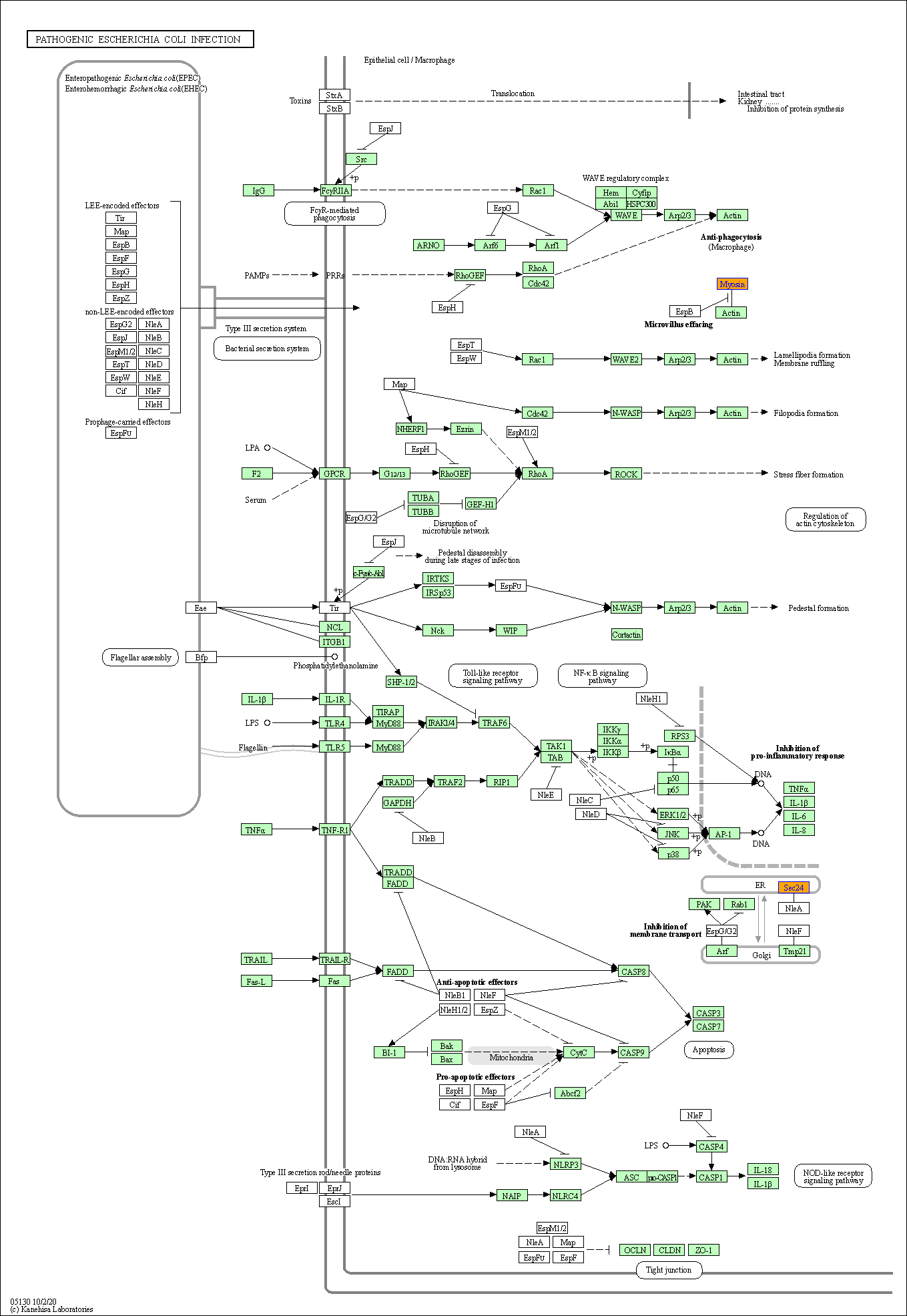

Supplement: Supplementary file 1 [file Data_Sheet_1.ZIP › Additional files/Pathway Analysis Report/Pathway_GC_vs_control_up/hsa05130.png]

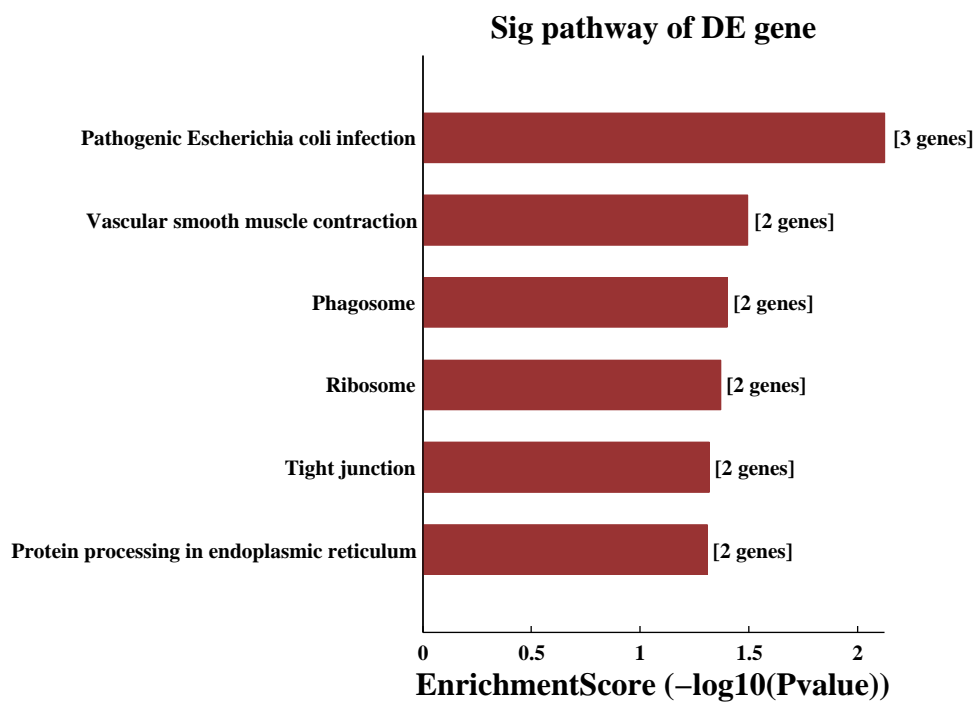

Supplement: Supplementary file 1 [file Data_Sheet_1.ZIP › Additional files/Pathway Analysis Report/Pathway_GC_vs_control_up/hsa_EnrichmentScore.pdf]

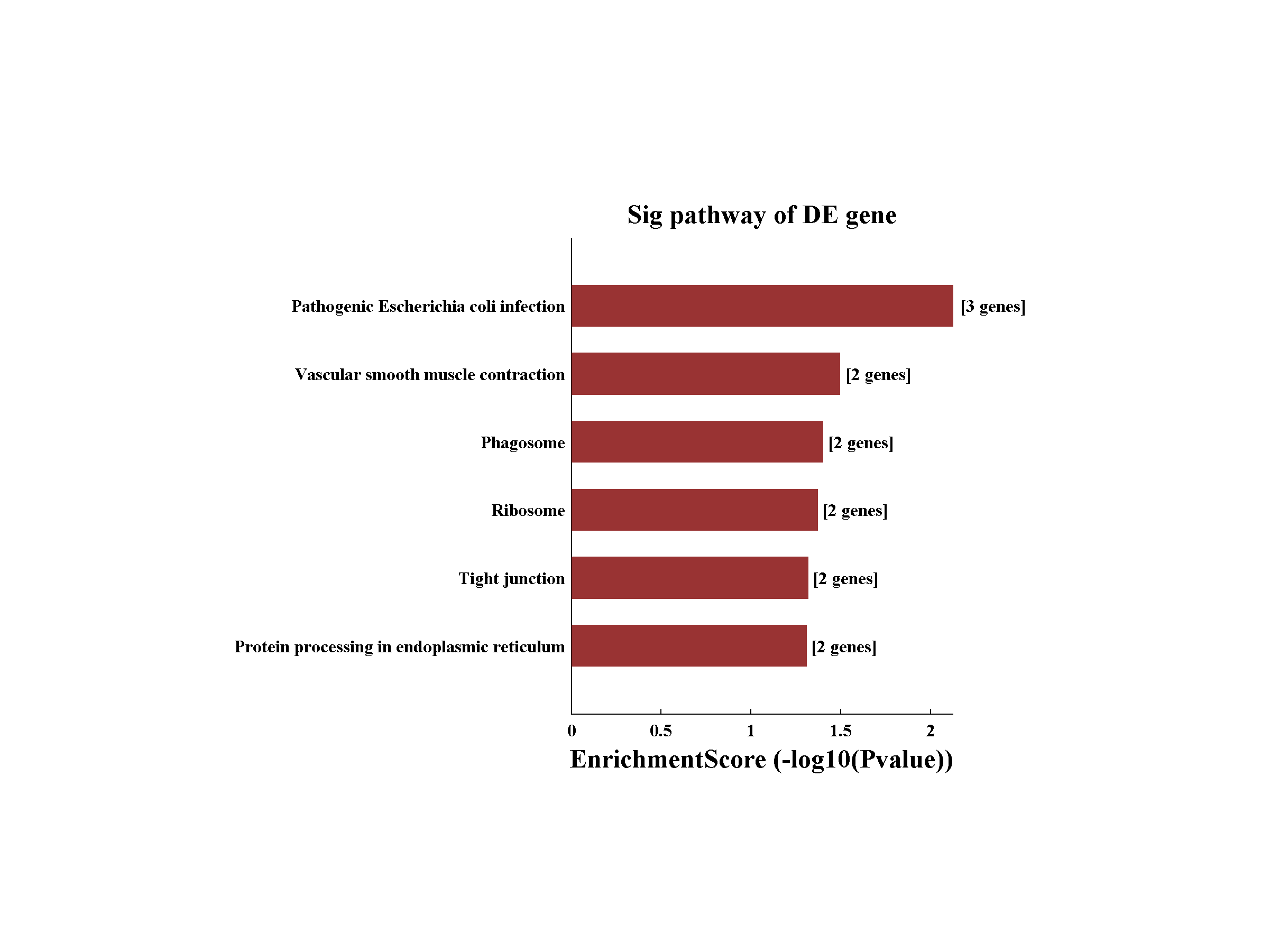

Supplement: Supplementary file 1 [file Data_Sheet_1.ZIP › Additional files/Pathway Analysis Report/Pathway_GC_vs_control_up/hsa_EnrichmentScore.png]

# Sig pathway of DE gene

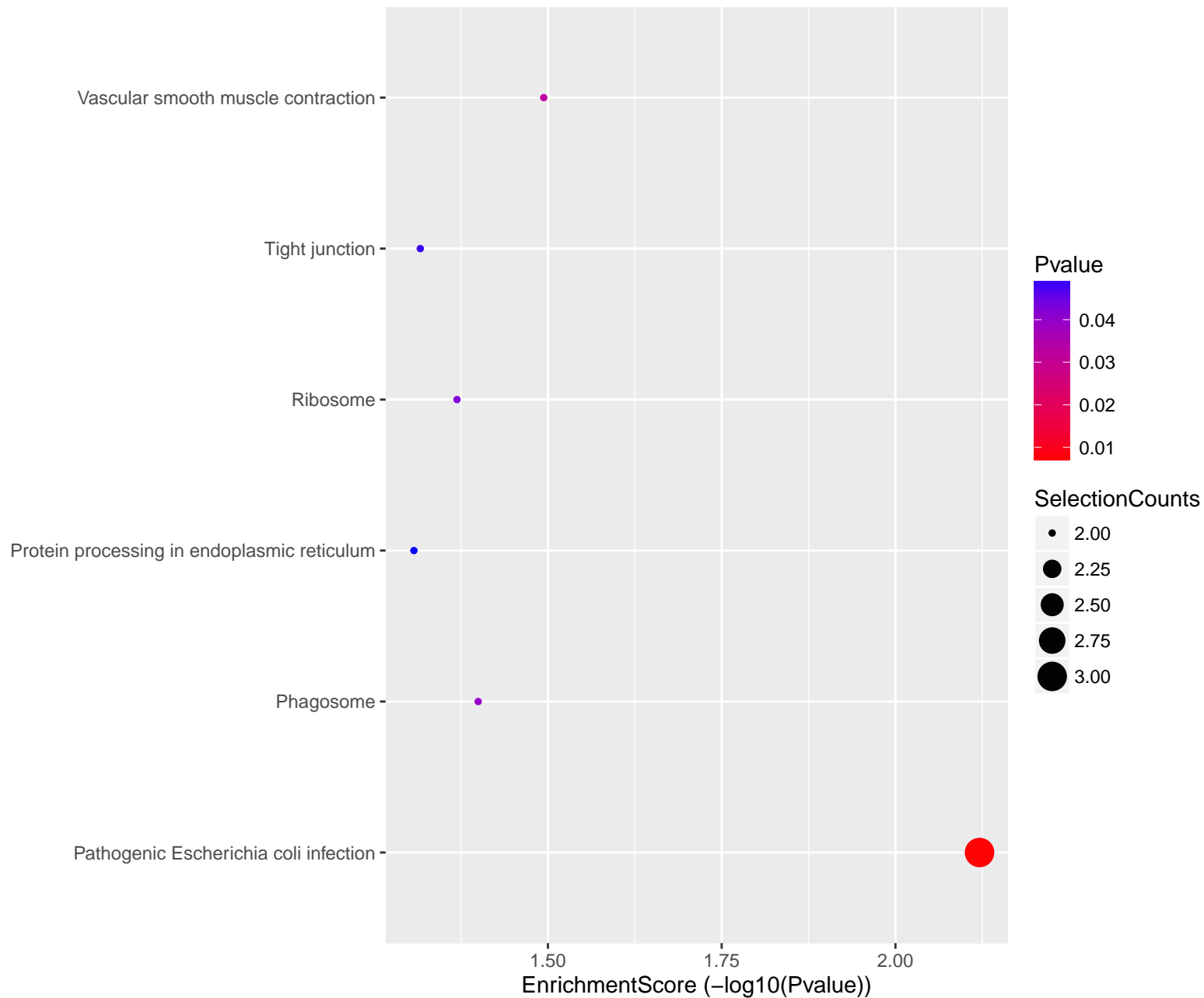

Supplement: Supplementary file 1 [file Data_Sheet_1.ZIP › Additional files/Pathway Analysis Report/Pathway_GC_vs_control_up/hsa_EnrichmentScoreDotPlot.pdf]

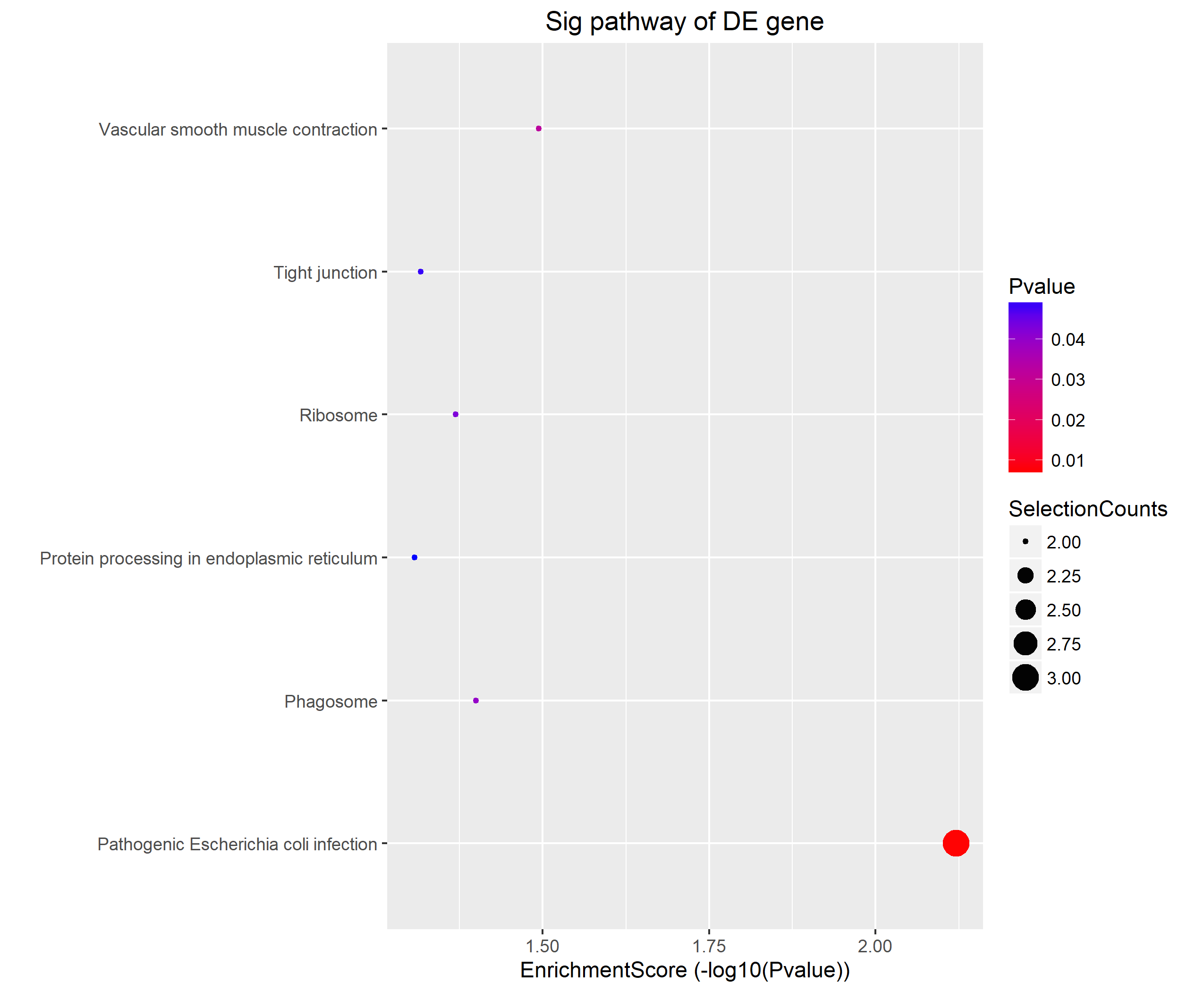

Supplement: Supplementary file 1 [file Data_Sheet_1.ZIP › Additional files/Pathway Analysis Report/Pathway_GC_vs_control_up/hsa_EnrichmentScoreDotPlot.png]

# Sig pathway of DE gene

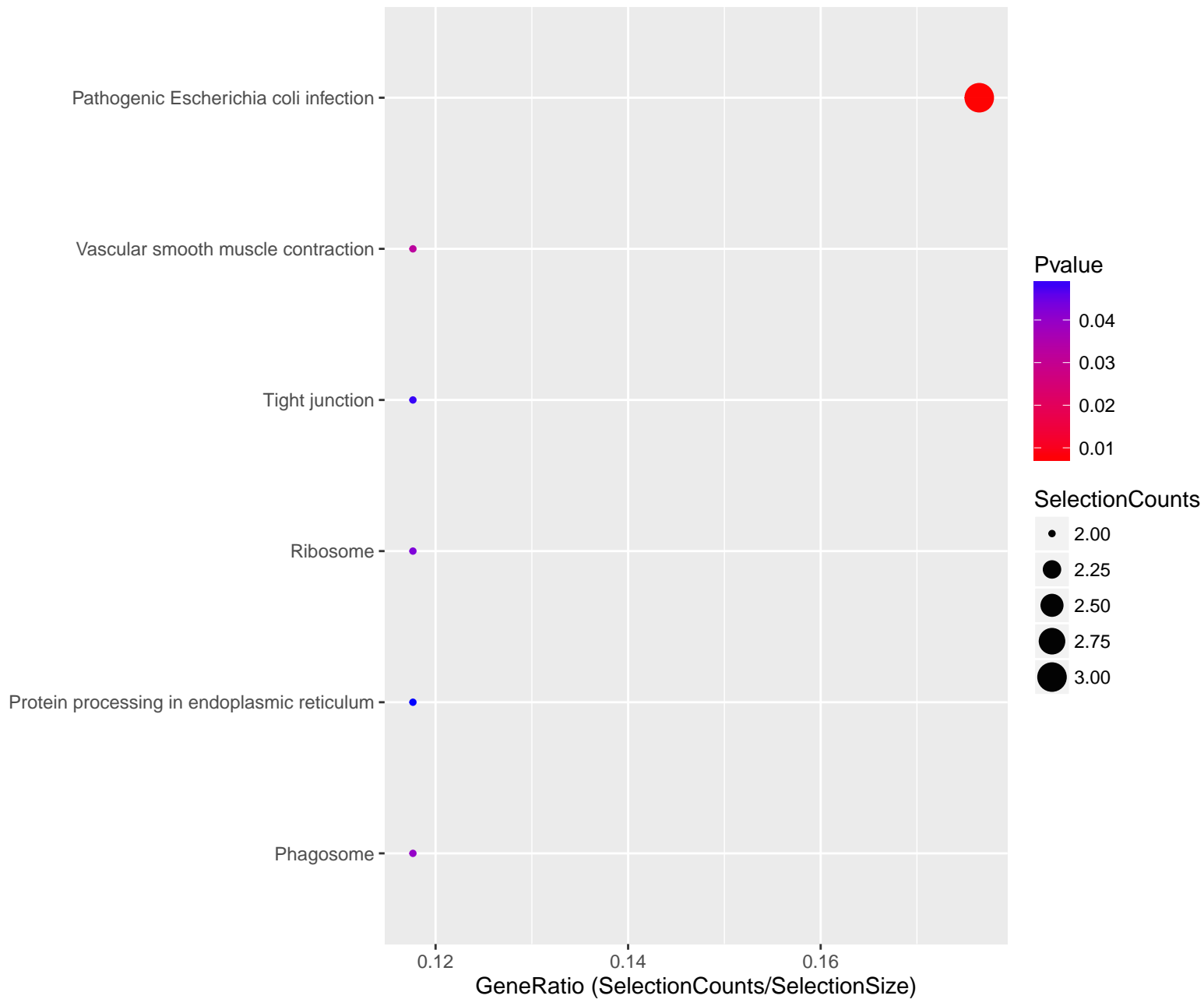

Supplement: Supplementary file 1 [file Data_Sheet_1.ZIP › Additional files/Pathway Analysis Report/Pathway_GC_vs_control_up/hsa_GeneRatioDotPlot.pdf]

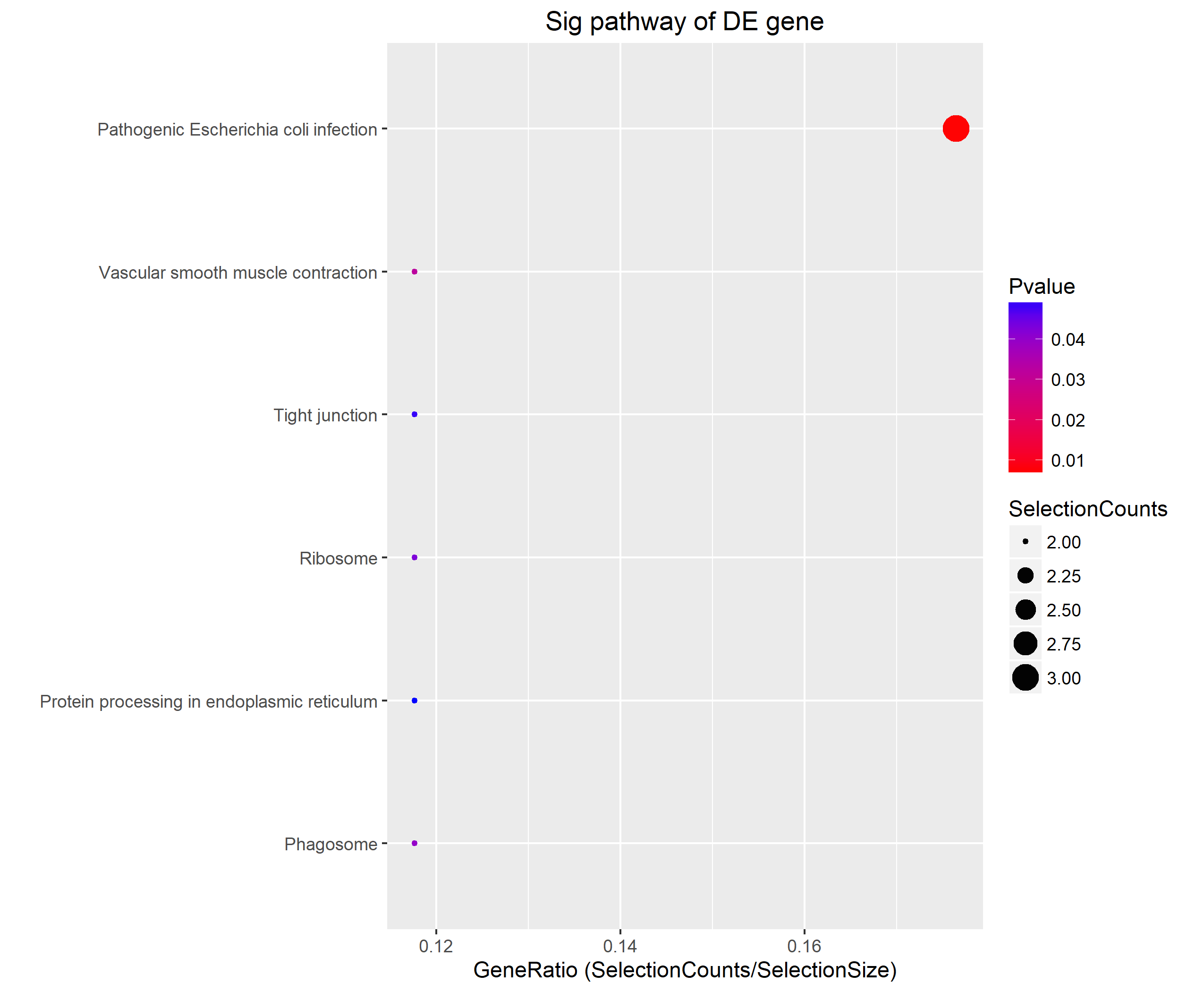

Supplement: Supplementary file 1 [file Data_Sheet_1.ZIP › Additional files/Pathway Analysis Report/Pathway_GC_vs_control_up/hsa_GeneRatioDotPlot.png]
