# Supplementary figures and images for: Differentially Expressed Circular RNAs and Their Therapeutic Mechanism in Non-segmental Vitiligo Patients Treated With Methylprednisolone (part 1 of 2)
Source: Front Med (Lausanne). 2022 May 16;9:839066. doi: 10.3389/fmed.2022.839066 (PMC9149005; doi:10.3389/fmed.2022.839066)

# GO Biological Process Classification

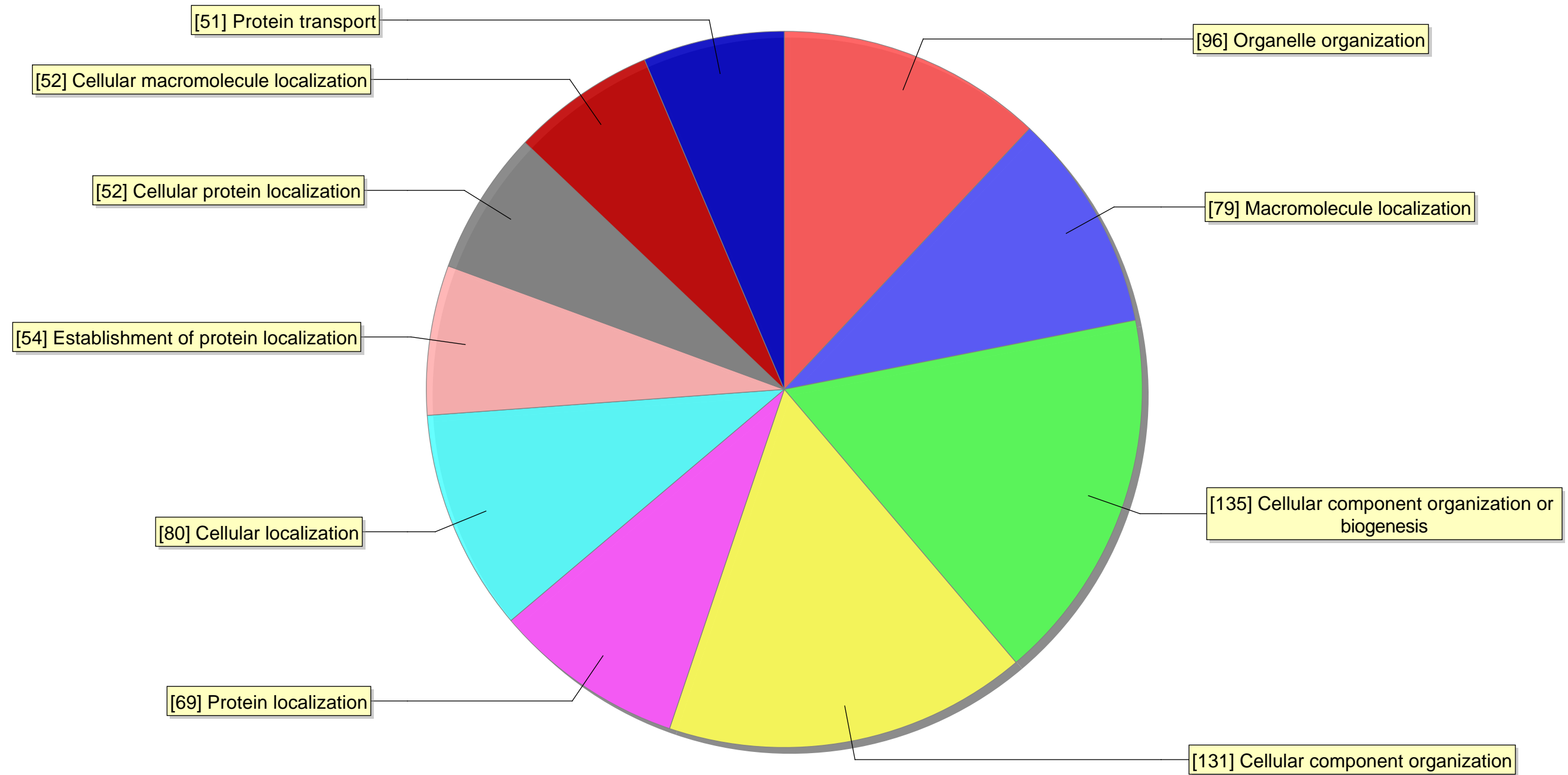

Supplement: Supplementary file 1 [file Data_Sheet_1.ZIP › Additional files/GO Analysis Report/GO_GC_vs_control_down/BP_Count.pdf]

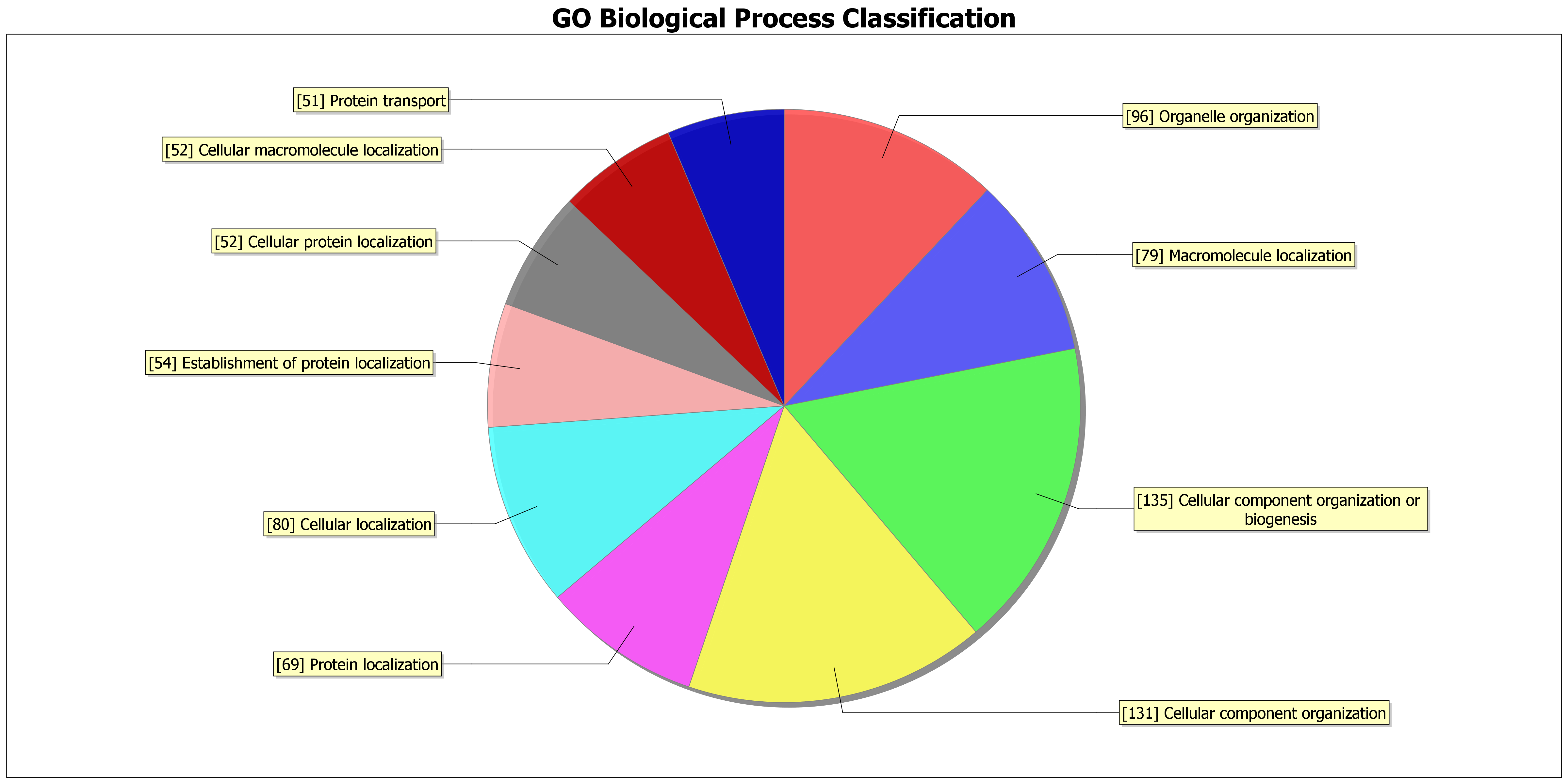

Supplement: Supplementary file 1 [file Data_Sheet_1.ZIP › Additional files/GO Analysis Report/GO_GC_vs_control_down/BP_Count.png]

## Sig GO terms of DE gene-BP

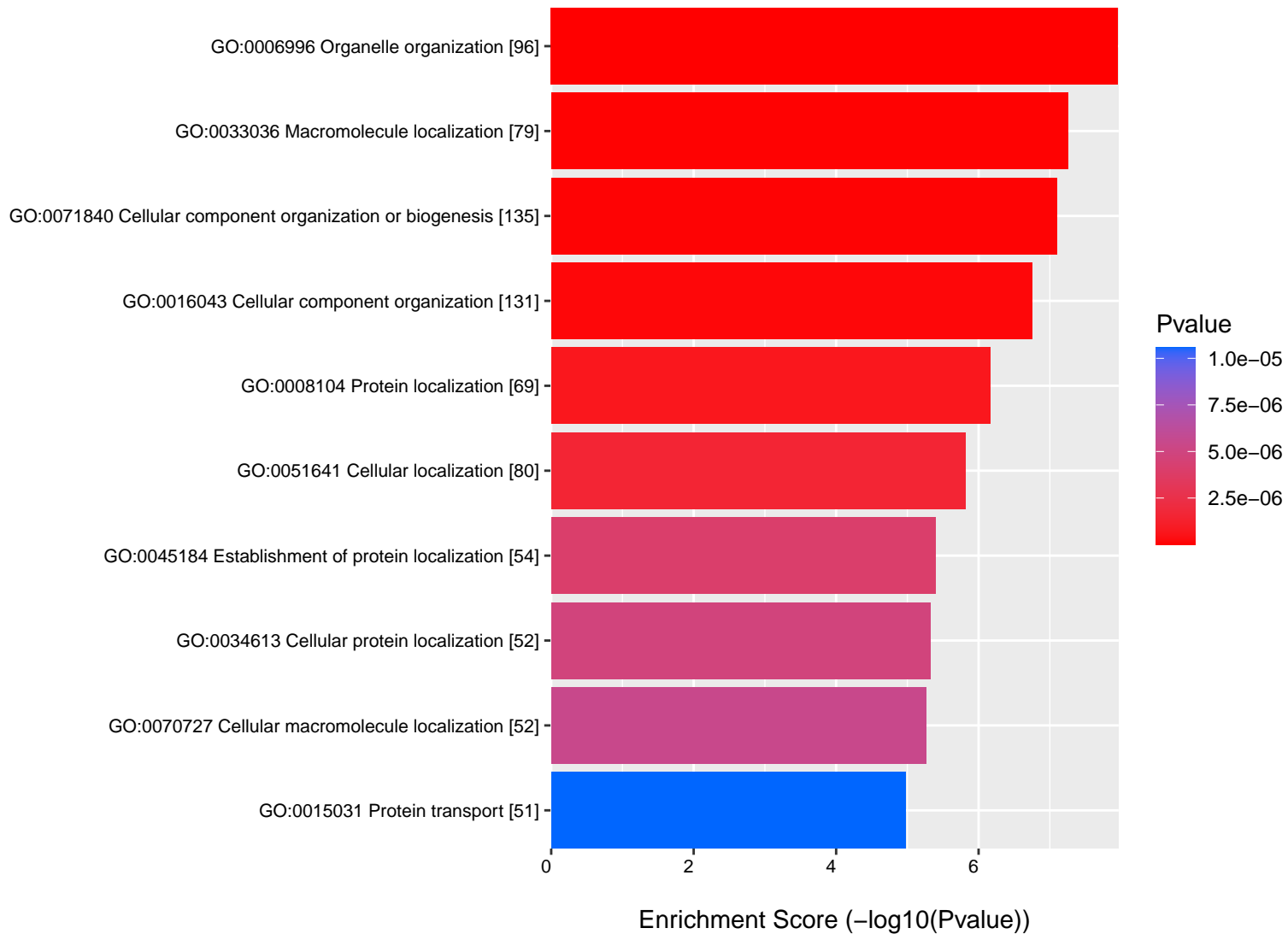

Supplement: Supplementary file 1 [file Data_Sheet_1.ZIP › Additional files/GO Analysis Report/GO_GC_vs_control_down/BP_EnrichmentScore.pdf]

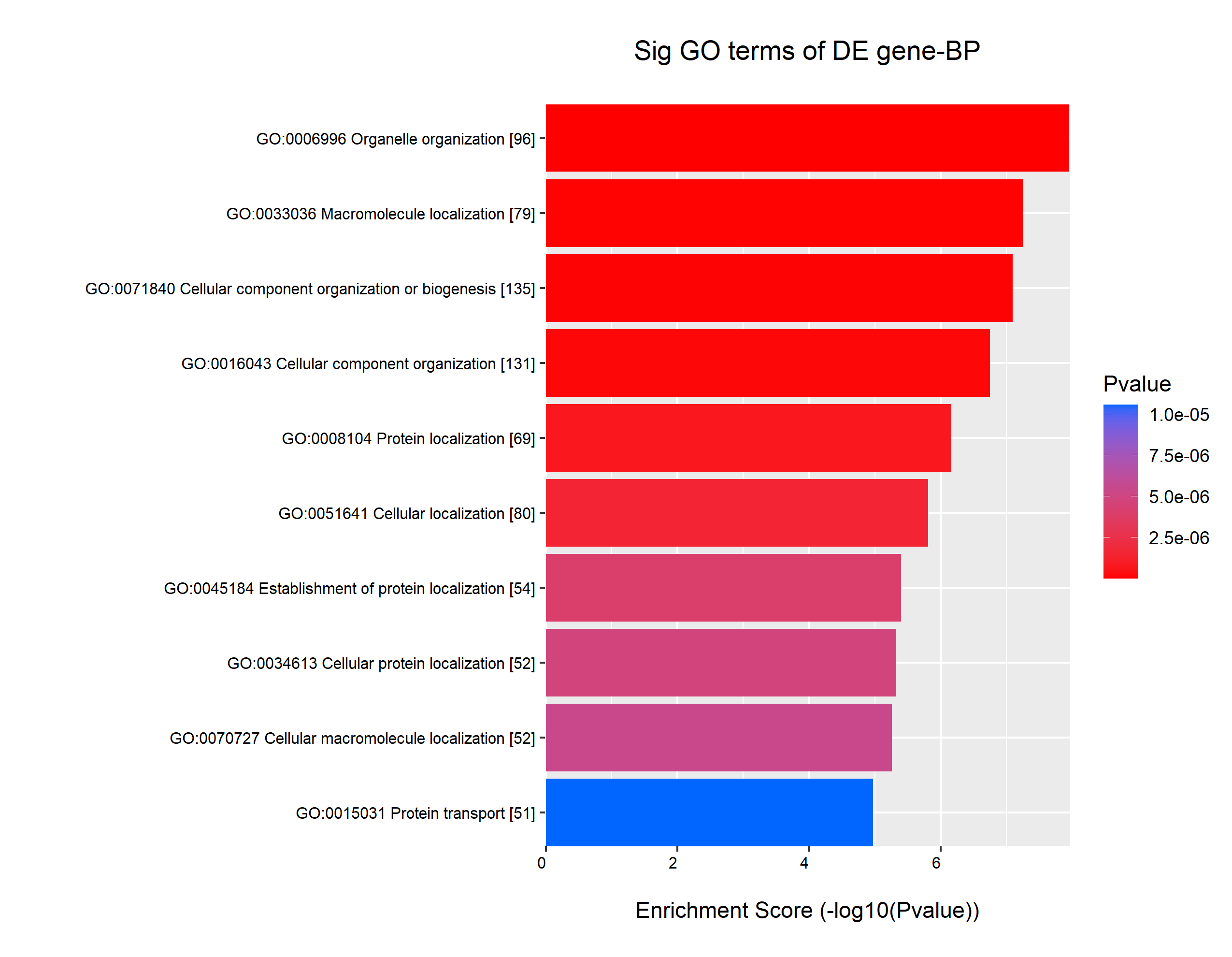

Supplement: Supplementary file 1 [file Data_Sheet_1.ZIP › Additional files/GO Analysis Report/GO_GC_vs_control_down/BP_EnrichmentScore.png]

# Sig GO terms of DE gene-BP

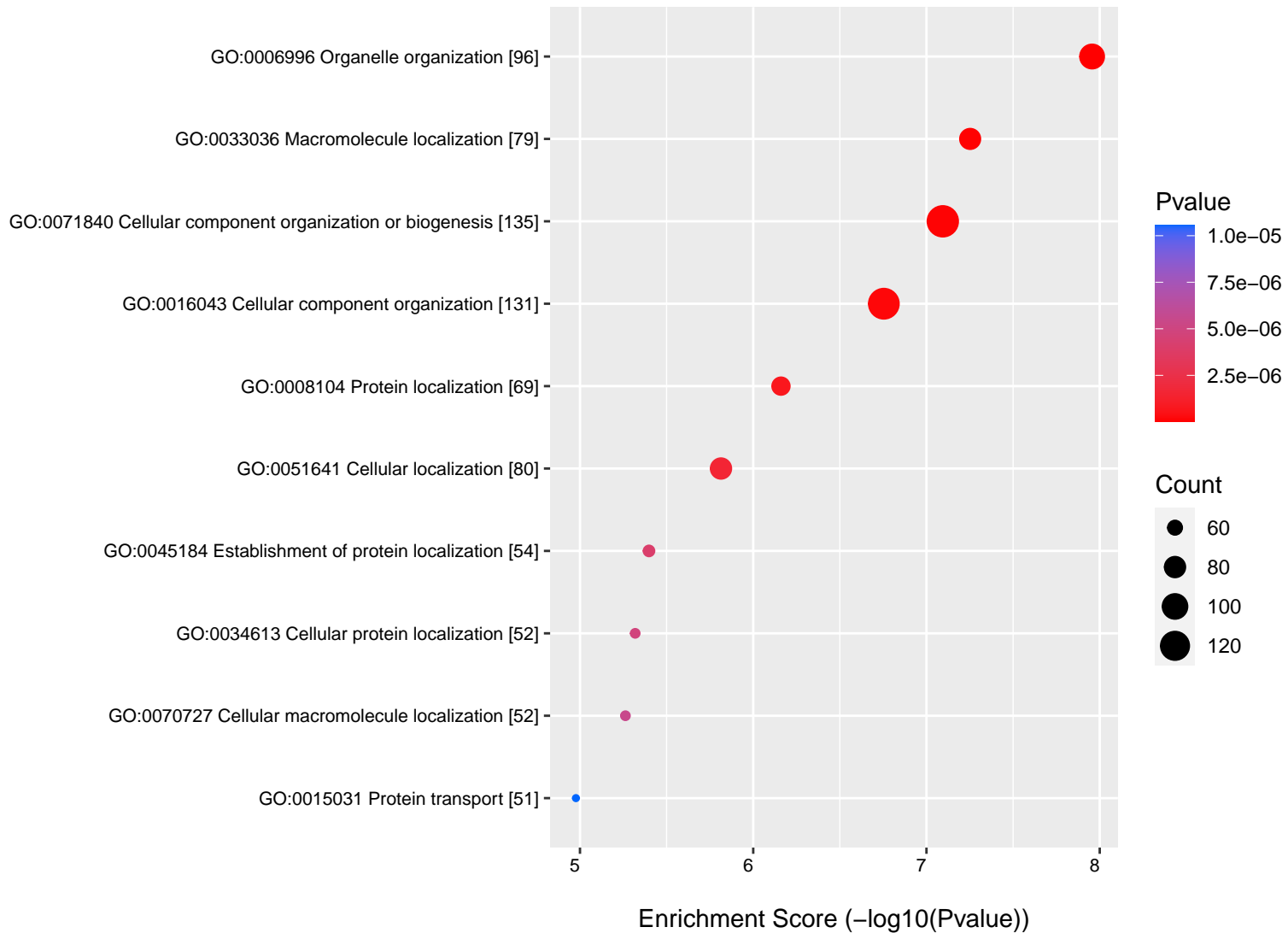

Supplement: Supplementary file 1 [file Data_Sheet_1.ZIP › Additional files/GO Analysis Report/GO_GC_vs_control_down/BP_EnrichmentScoreDotPlot.pdf]

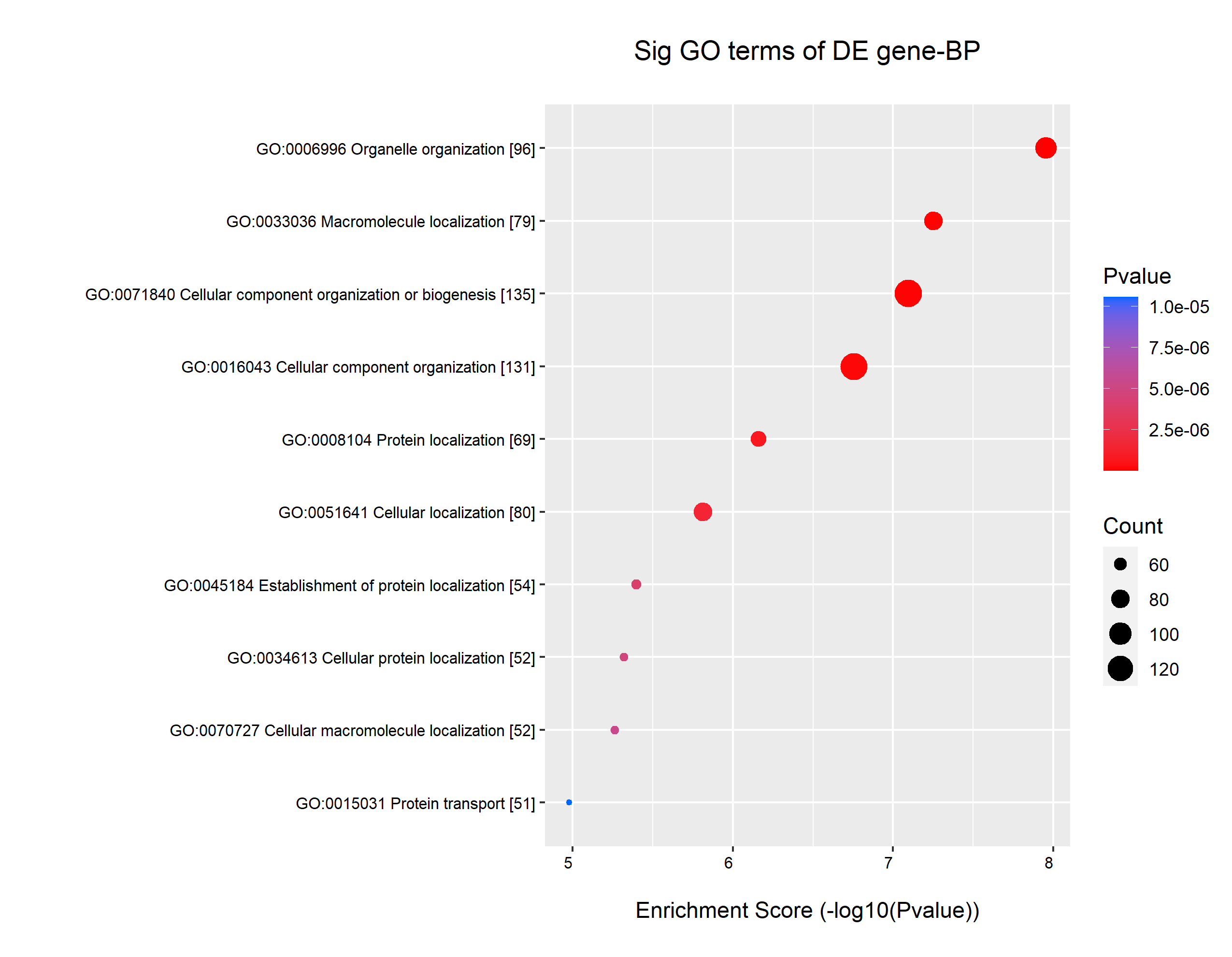

Supplement: Supplementary file 1 [file Data_Sheet_1.ZIP › Additional files/GO Analysis Report/GO_GC_vs_control_down/BP_EnrichmentScoreDotPlot.png]

## Sig GO terms of DE gene-BP

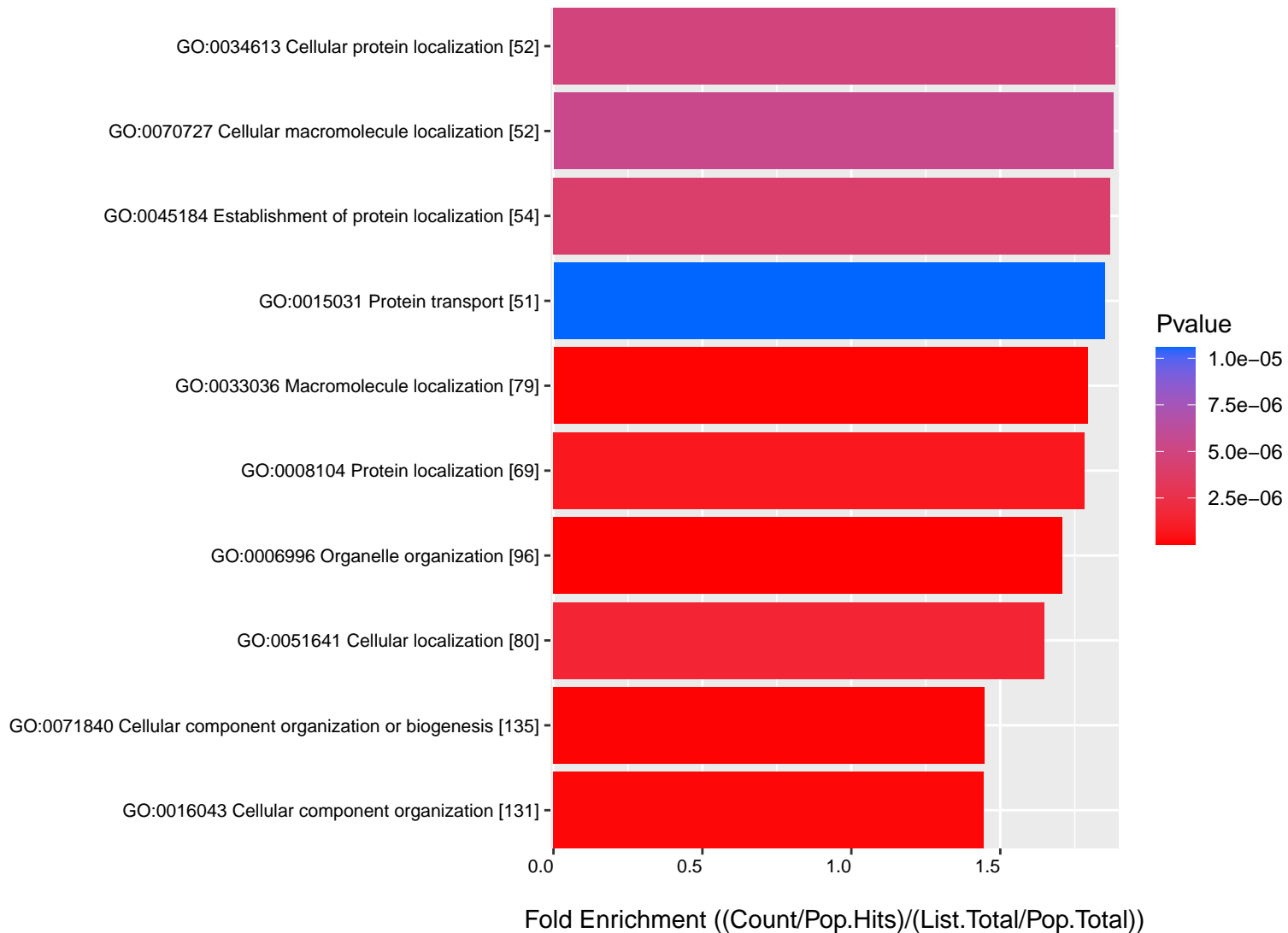

Supplement: Supplementary file 1 [file Data_Sheet_1.ZIP › Additional files/GO Analysis Report/GO_GC_vs_control_down/BP_FoldEnrichment.pdf]

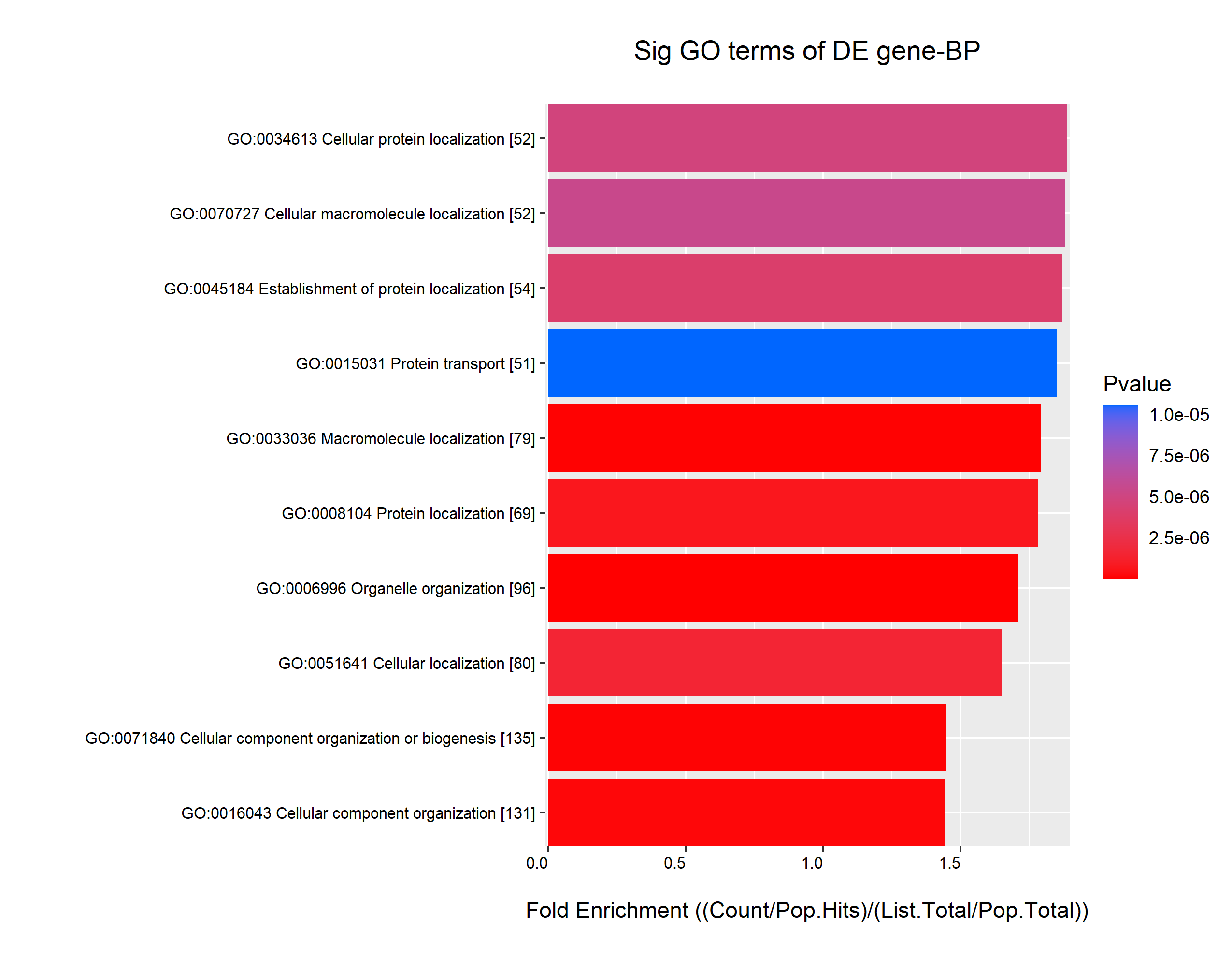

Supplement: Supplementary file 1 [file Data_Sheet_1.ZIP › Additional files/GO Analysis Report/GO_GC_vs_control_down/BP_FoldEnrichment.png]

## Sig GO terms of DE gene-BP

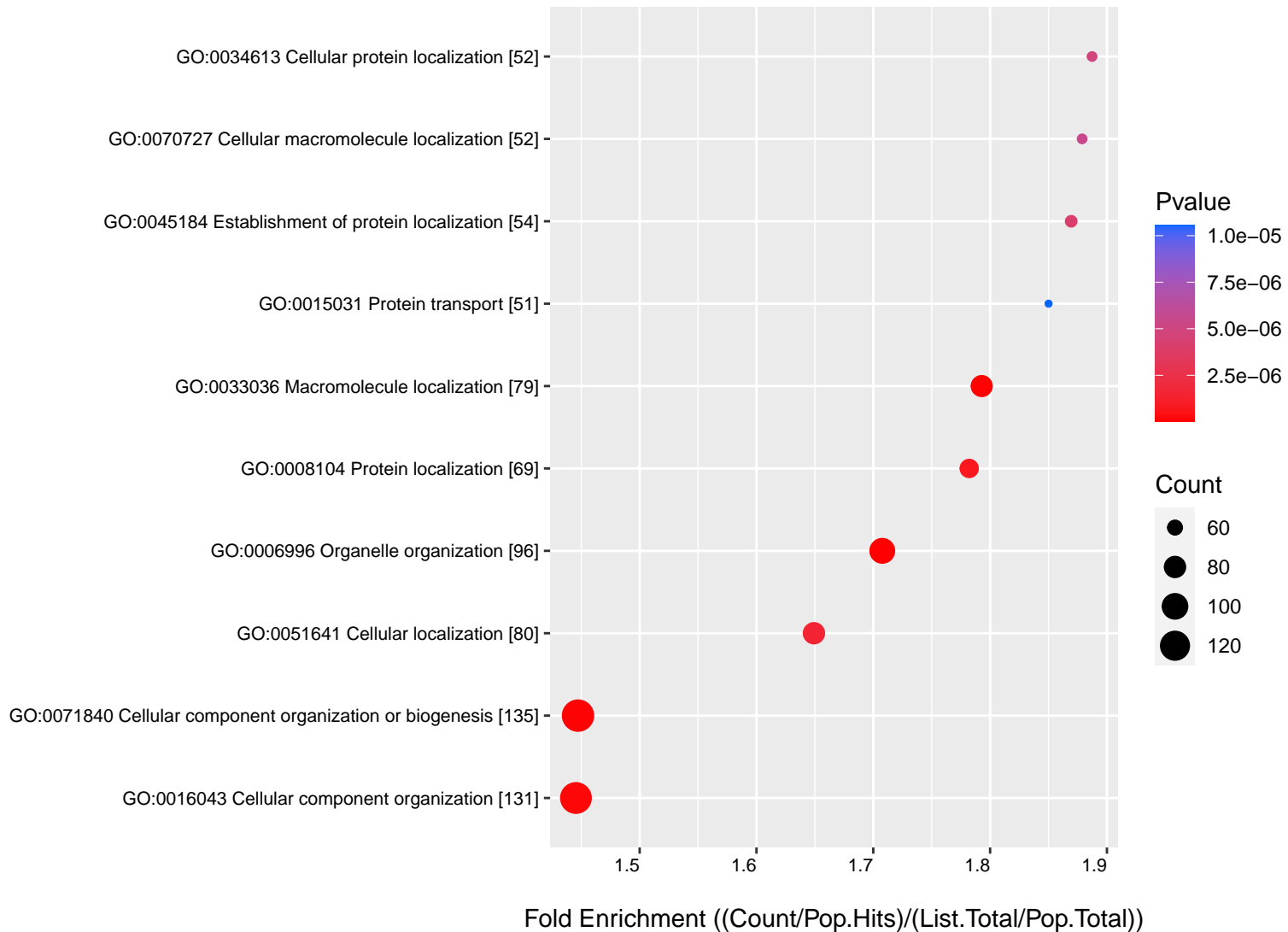

Supplement: Supplementary file 1 [file Data_Sheet_1.ZIP › Additional files/GO Analysis Report/GO_GC_vs_control_down/BP_FoldEnrichmentDotPlot.pdf]

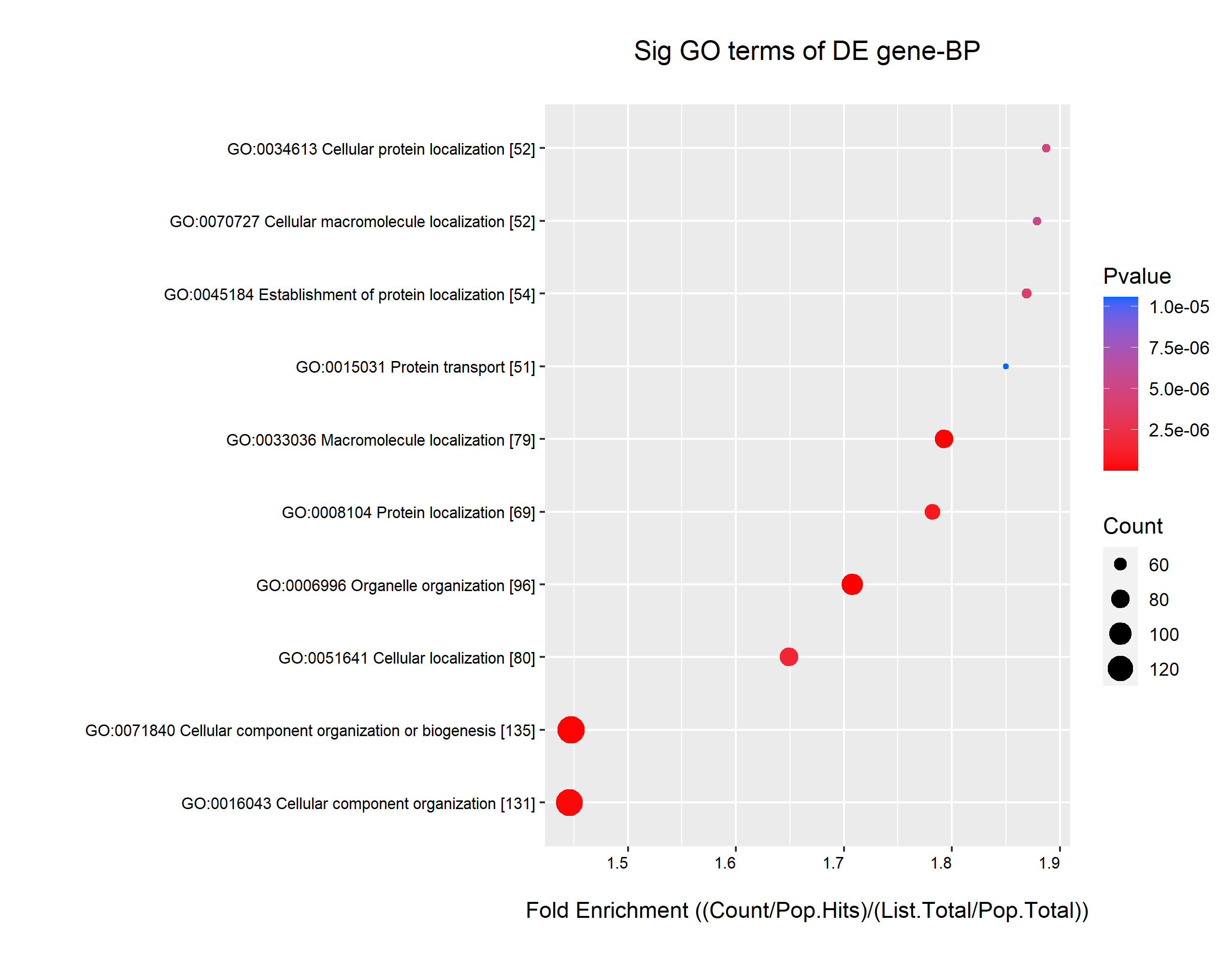

Supplement: Supplementary file 1 [file Data_Sheet_1.ZIP › Additional files/GO Analysis Report/GO_GC_vs_control_down/BP_FoldEnrichmentDotPlot.png]

## Sig GO terms of DE gene-BP

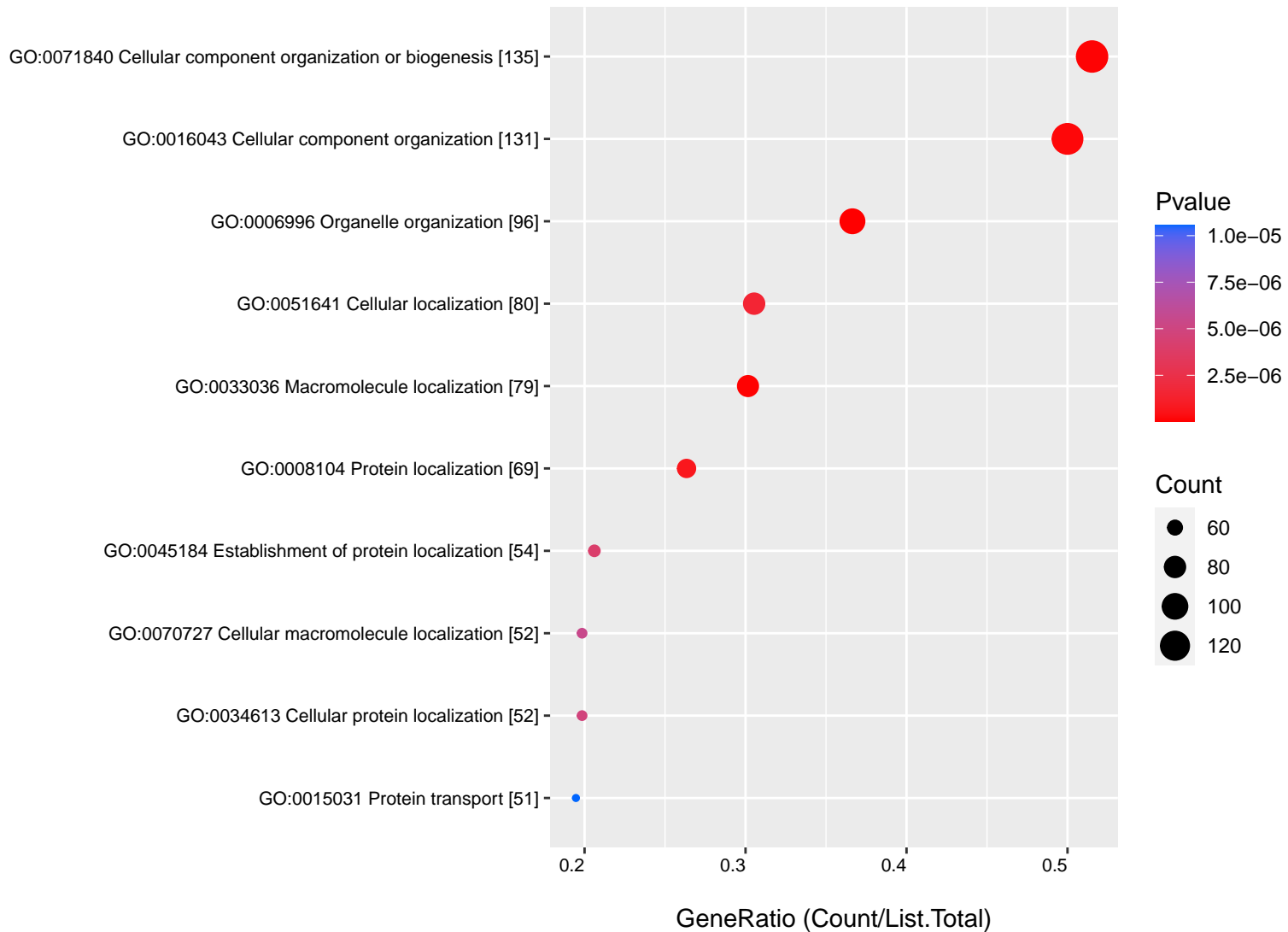

Supplement: Supplementary file 1 [file Data_Sheet_1.ZIP › Additional files/GO Analysis Report/GO_GC_vs_control_down/BP_GeneRatioDotPlot.pdf]

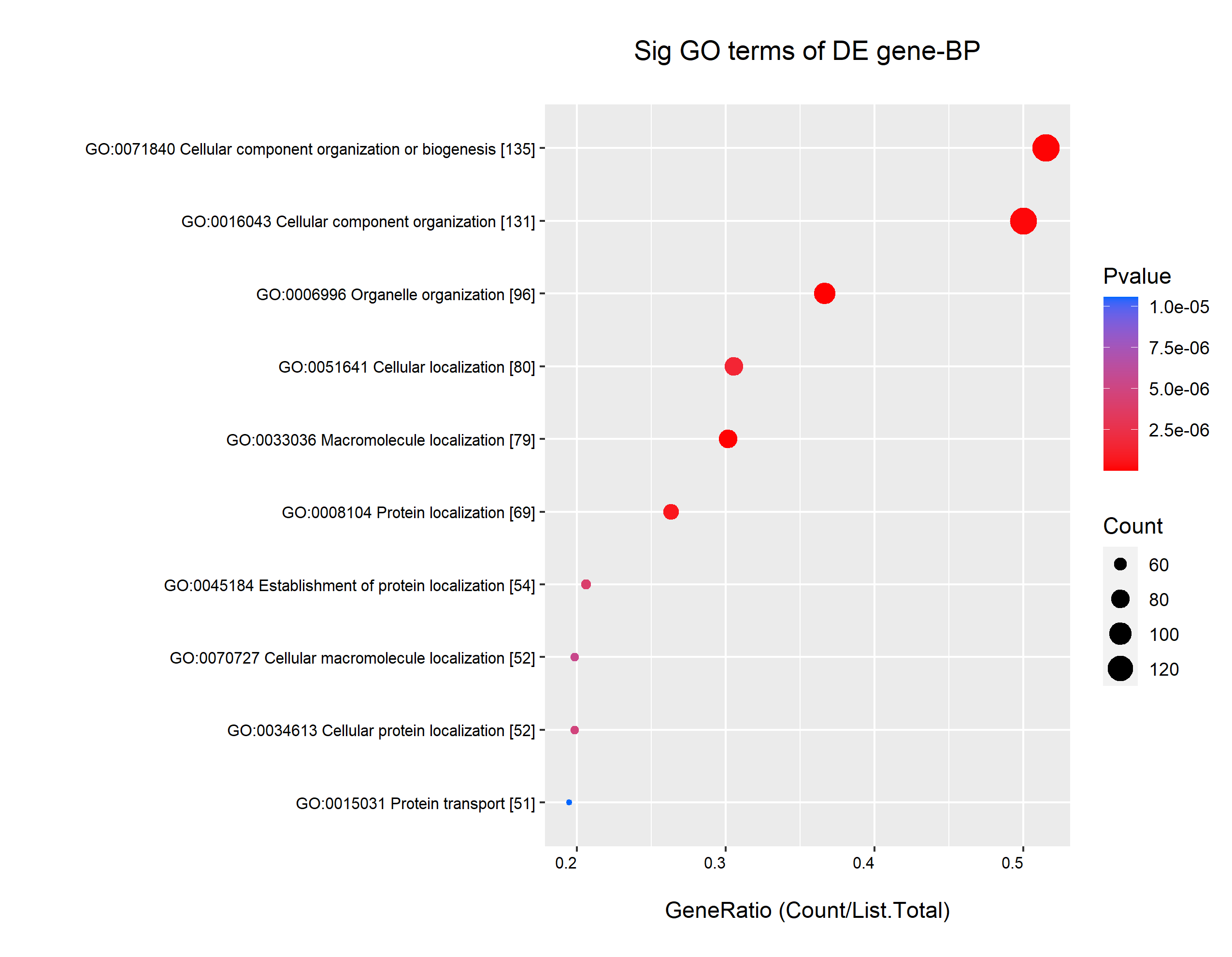

Supplement: Supplementary file 1 [file Data_Sheet_1.ZIP › Additional files/GO Analysis Report/GO_GC_vs_control_down/BP_GeneRatioDotPlot.png]

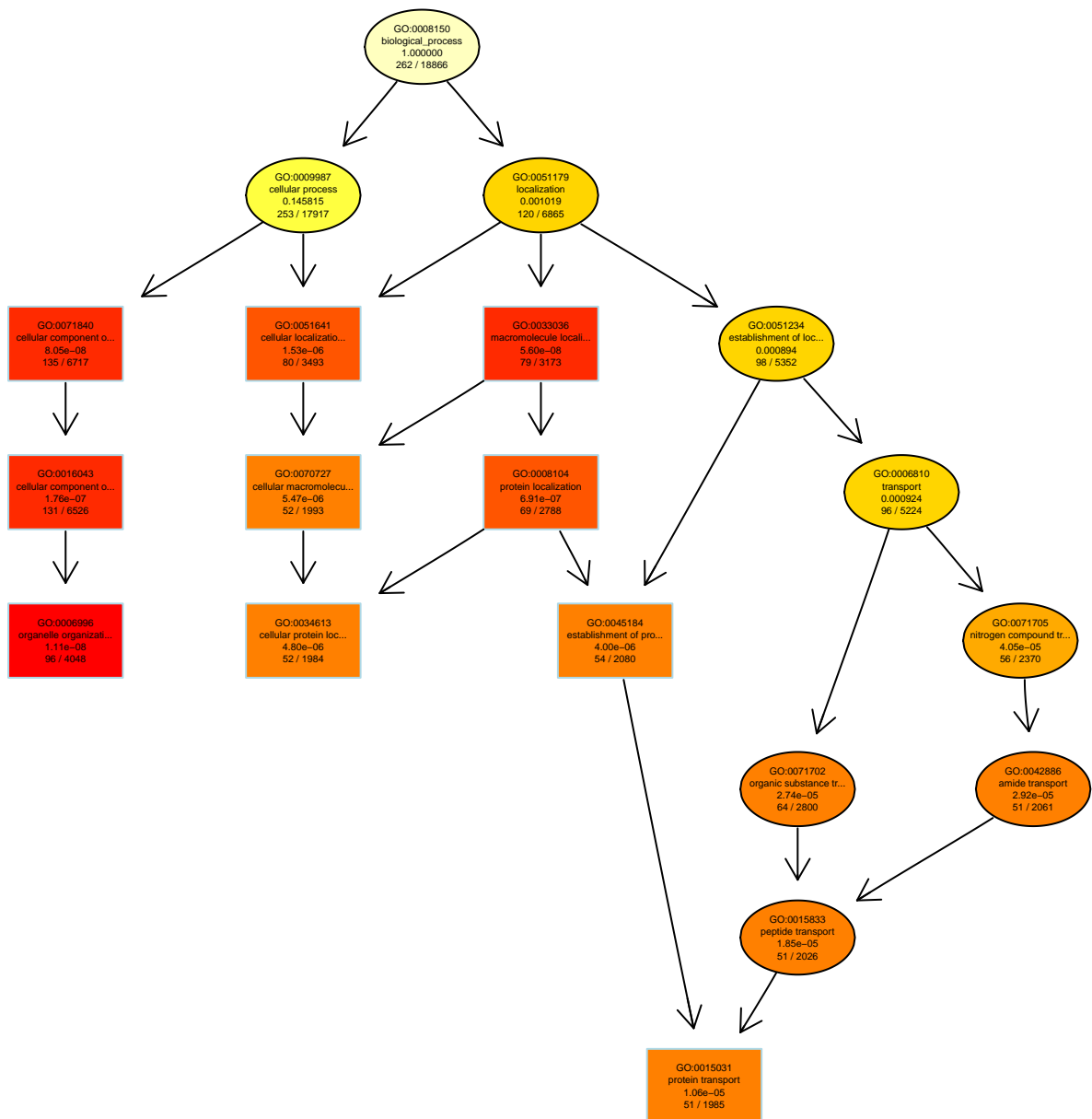

Supplement: Supplementary file 1 [file Data_Sheet_1.ZIP › Additional files/GO Analysis Report/GO_GC_vs_control_down/BP_Pvalue_tree.pdf]

# GO Cellular Component Classification

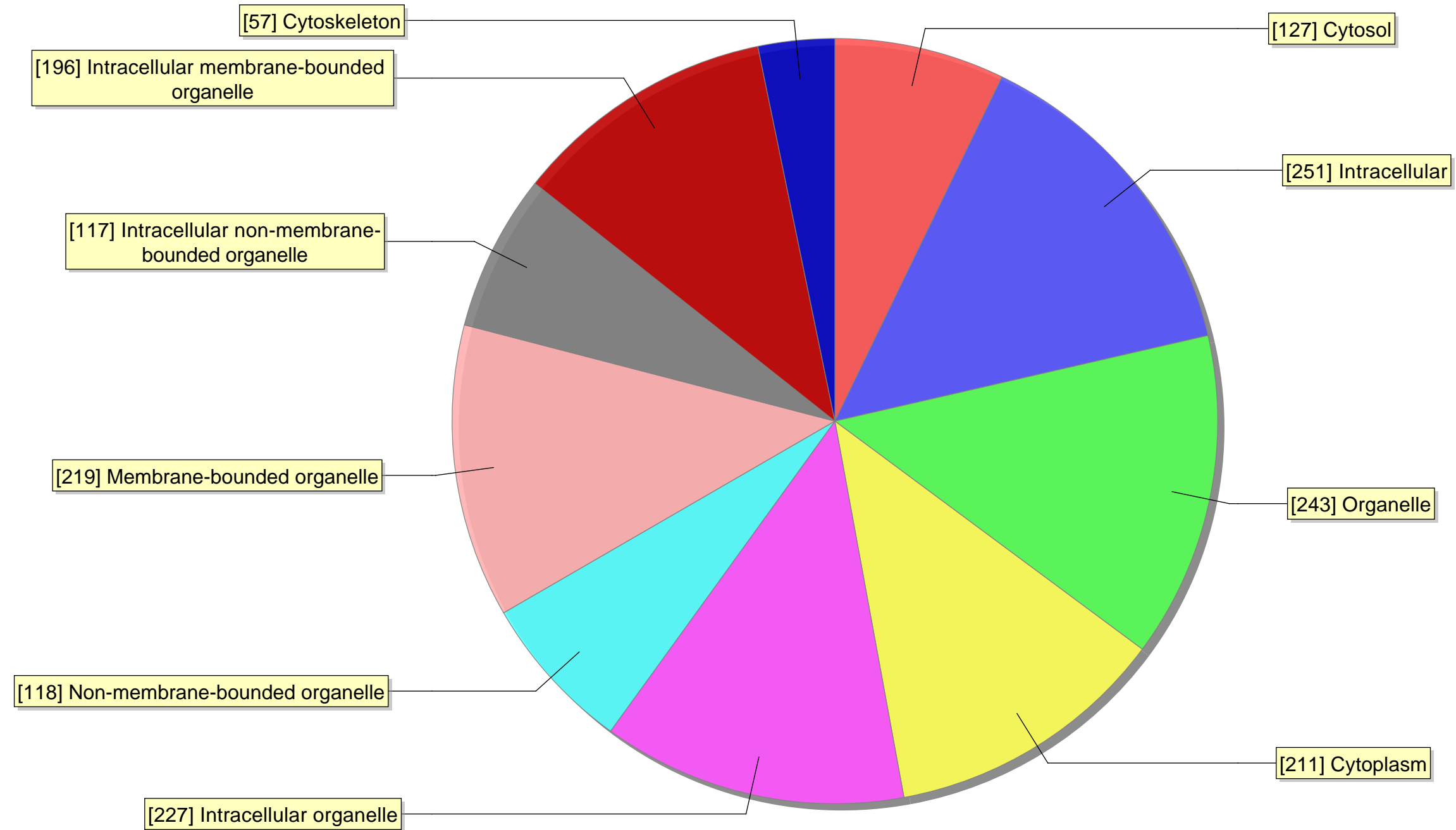

Supplement: Supplementary file 1 [file Data_Sheet_1.ZIP › Additional files/GO Analysis Report/GO_GC_vs_control_down/CC_Count.pdf]

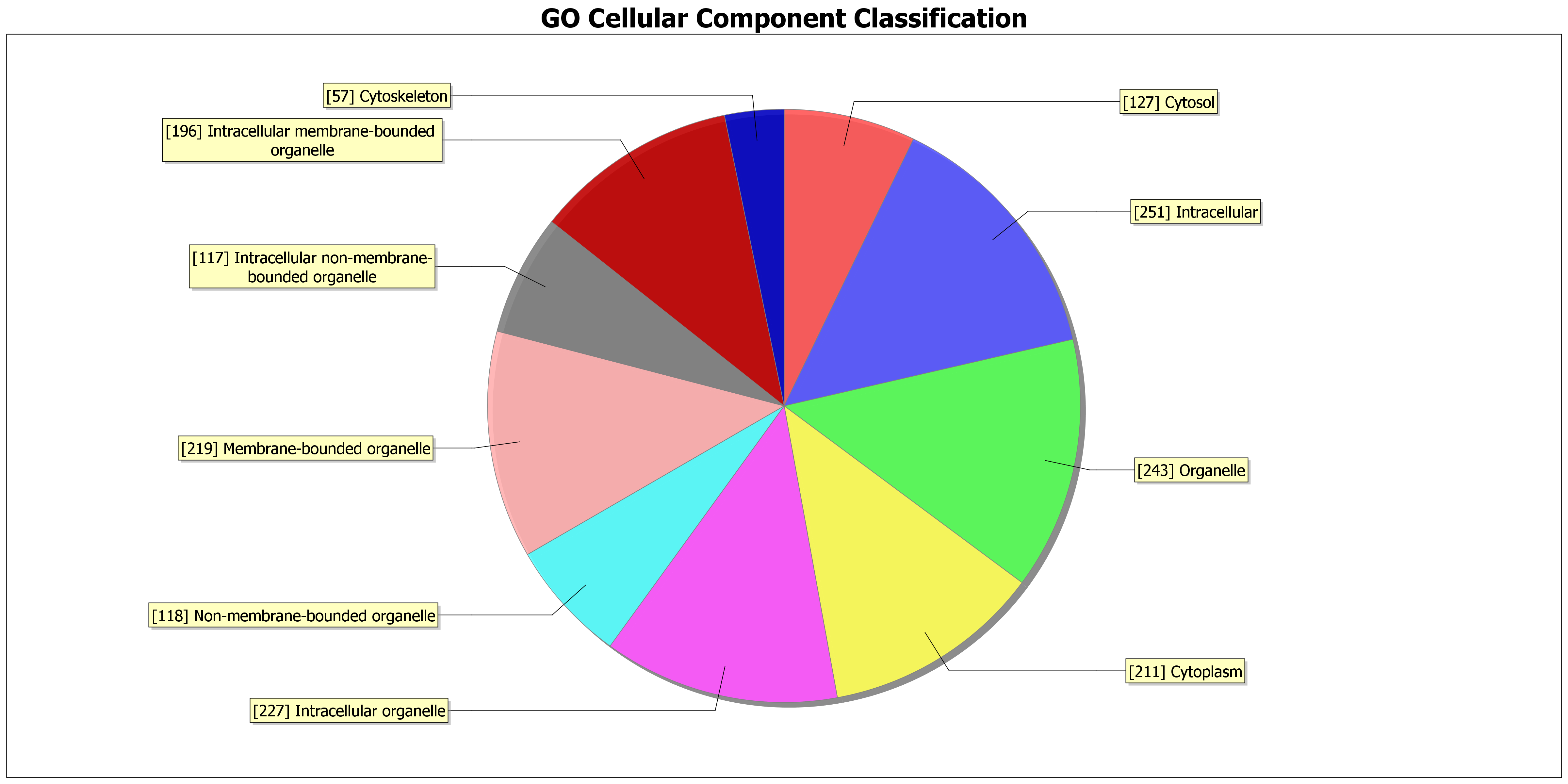

Supplement: Supplementary file 1 [file Data_Sheet_1.ZIP › Additional files/GO Analysis Report/GO_GC_vs_control_down/CC_Count.png]

## Sig GO terms of DE gene-CC

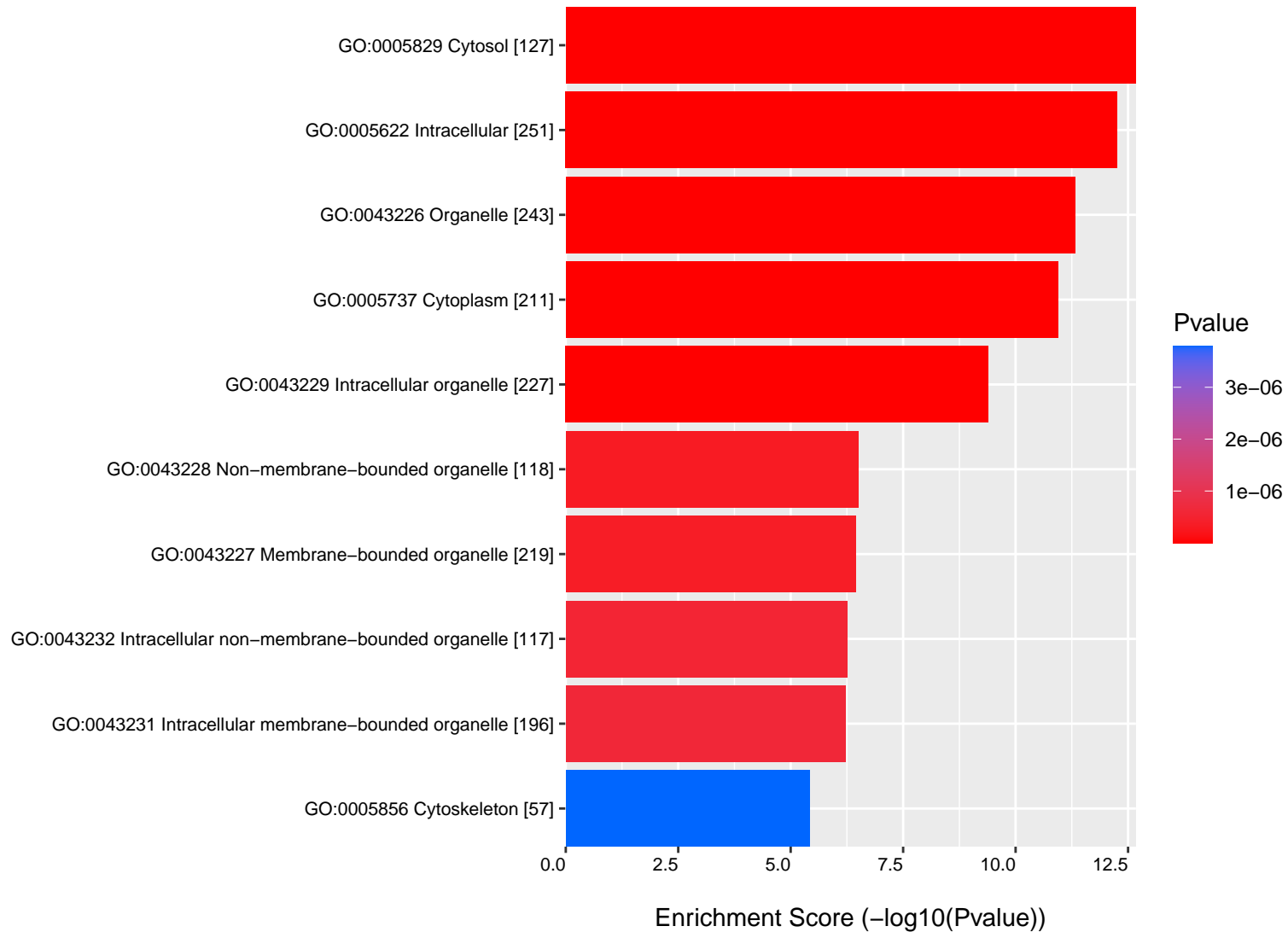

Supplement: Supplementary file 1 [file Data_Sheet_1.ZIP › Additional files/GO Analysis Report/GO_GC_vs_control_down/CC_EnrichmentScore.pdf]

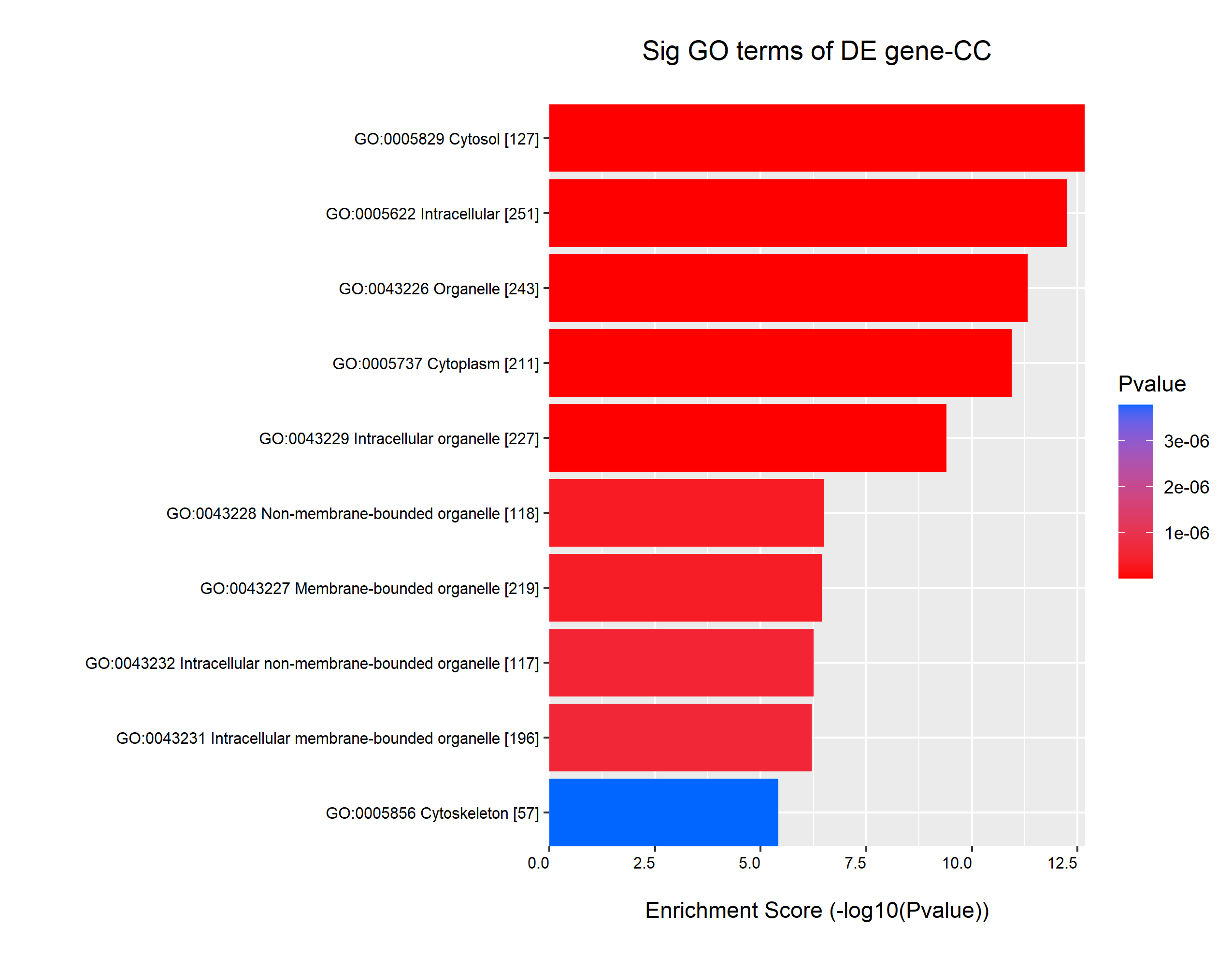

Supplement: Supplementary file 1 [file Data_Sheet_1.ZIP › Additional files/GO Analysis Report/GO_GC_vs_control_down/CC_EnrichmentScore.png]

## Sig GO terms of DE gene-CC

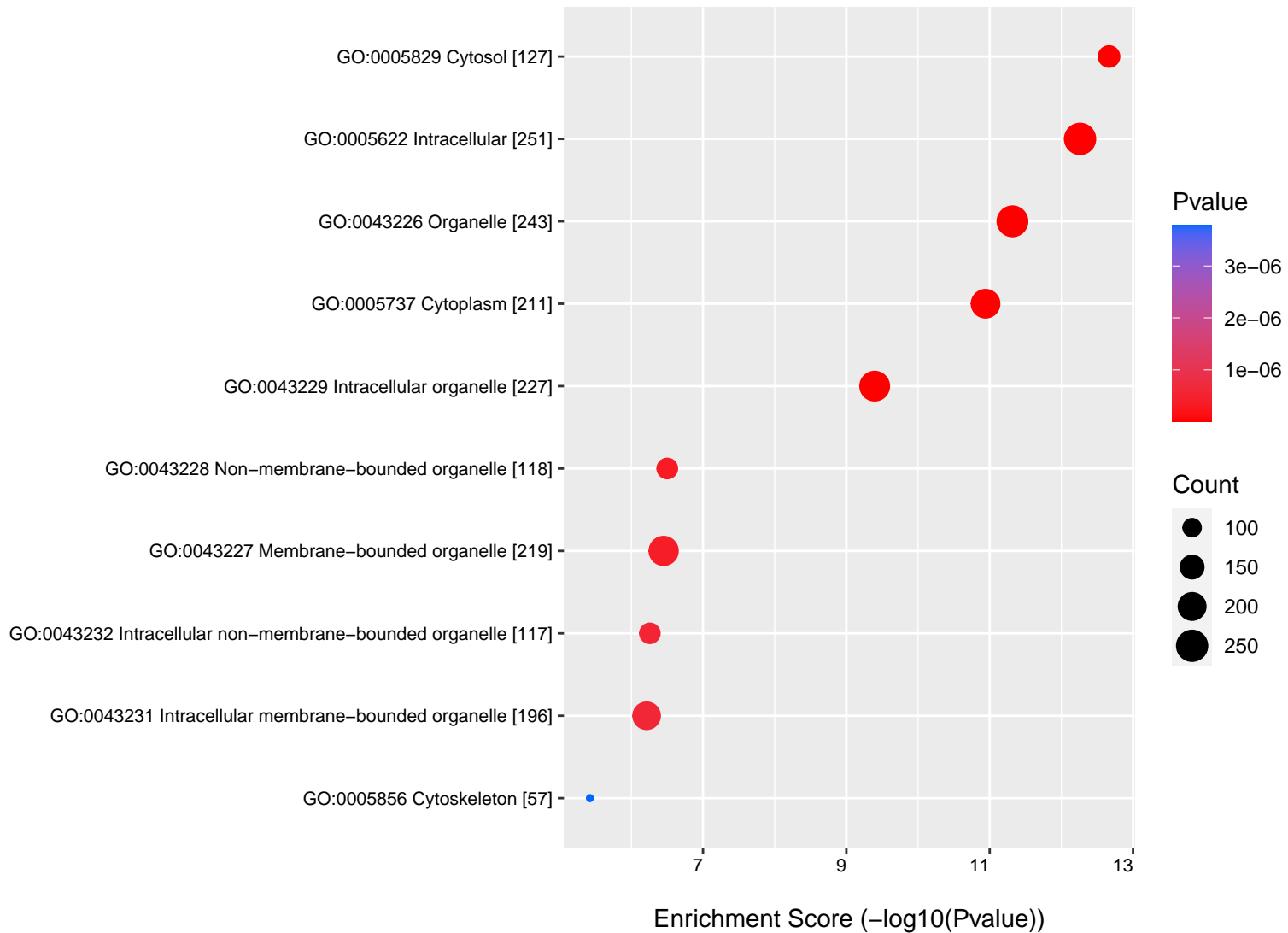

Supplement: Supplementary file 1 [file Data_Sheet_1.ZIP › Additional files/GO Analysis Report/GO_GC_vs_control_down/CC_EnrichmentScoreDotPlot.pdf]

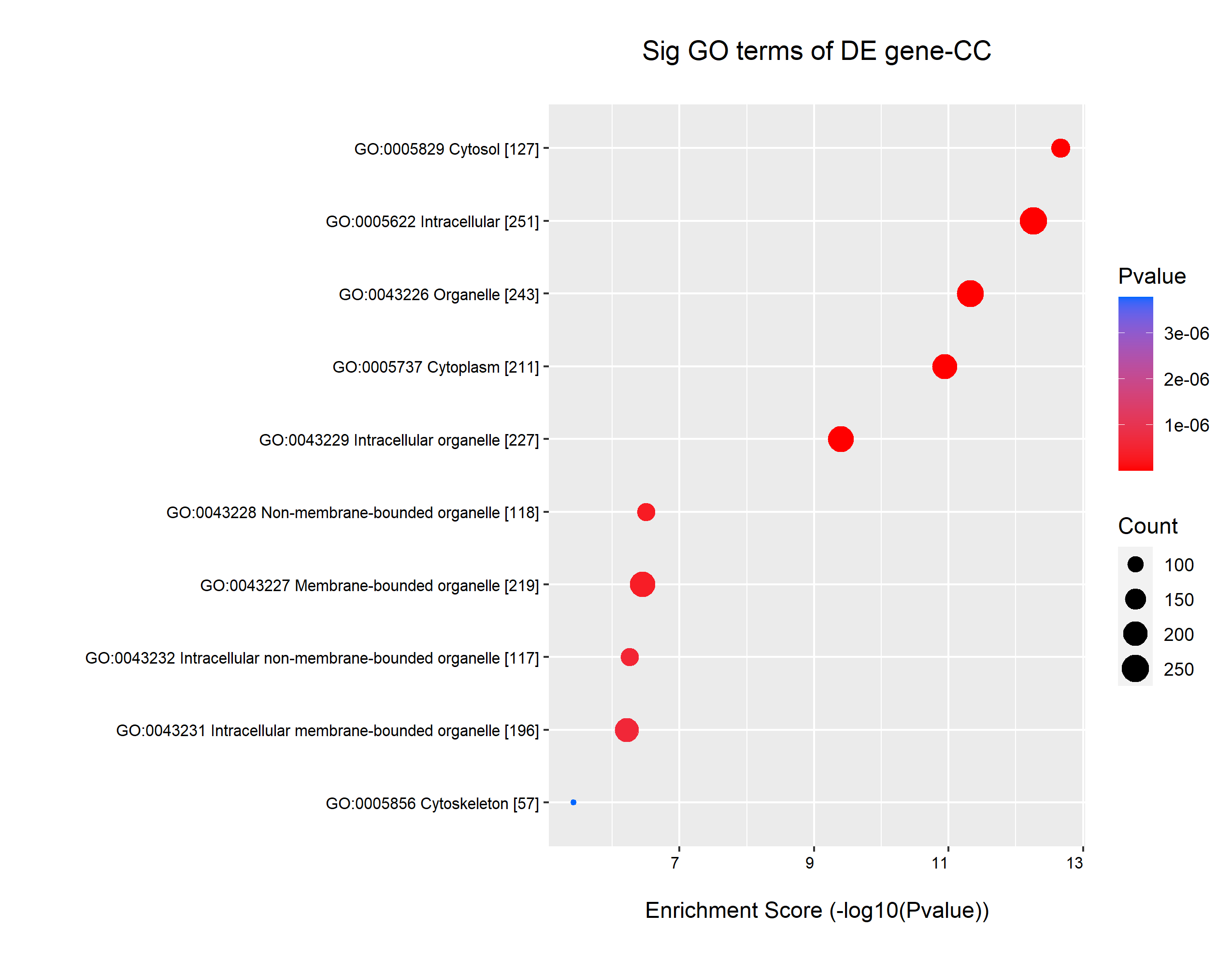

Supplement: Supplementary file 1 [file Data_Sheet_1.ZIP › Additional files/GO Analysis Report/GO_GC_vs_control_down/CC_EnrichmentScoreDotPlot.png]

## Sig GO terms of DE gene-CC

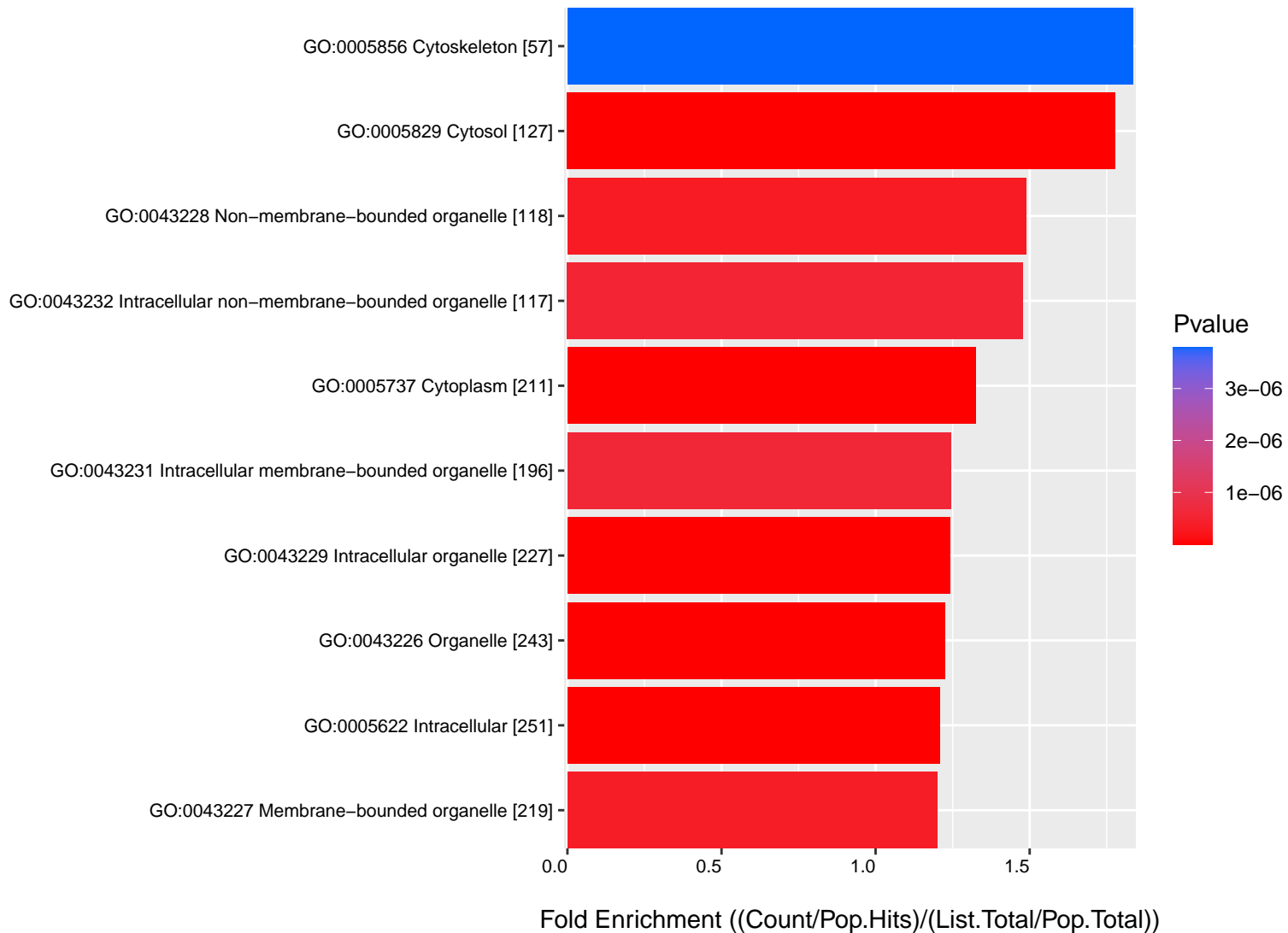

Supplement: Supplementary file 1 [file Data_Sheet_1.ZIP › Additional files/GO Analysis Report/GO_GC_vs_control_down/CC_FoldEnrichment.pdf]

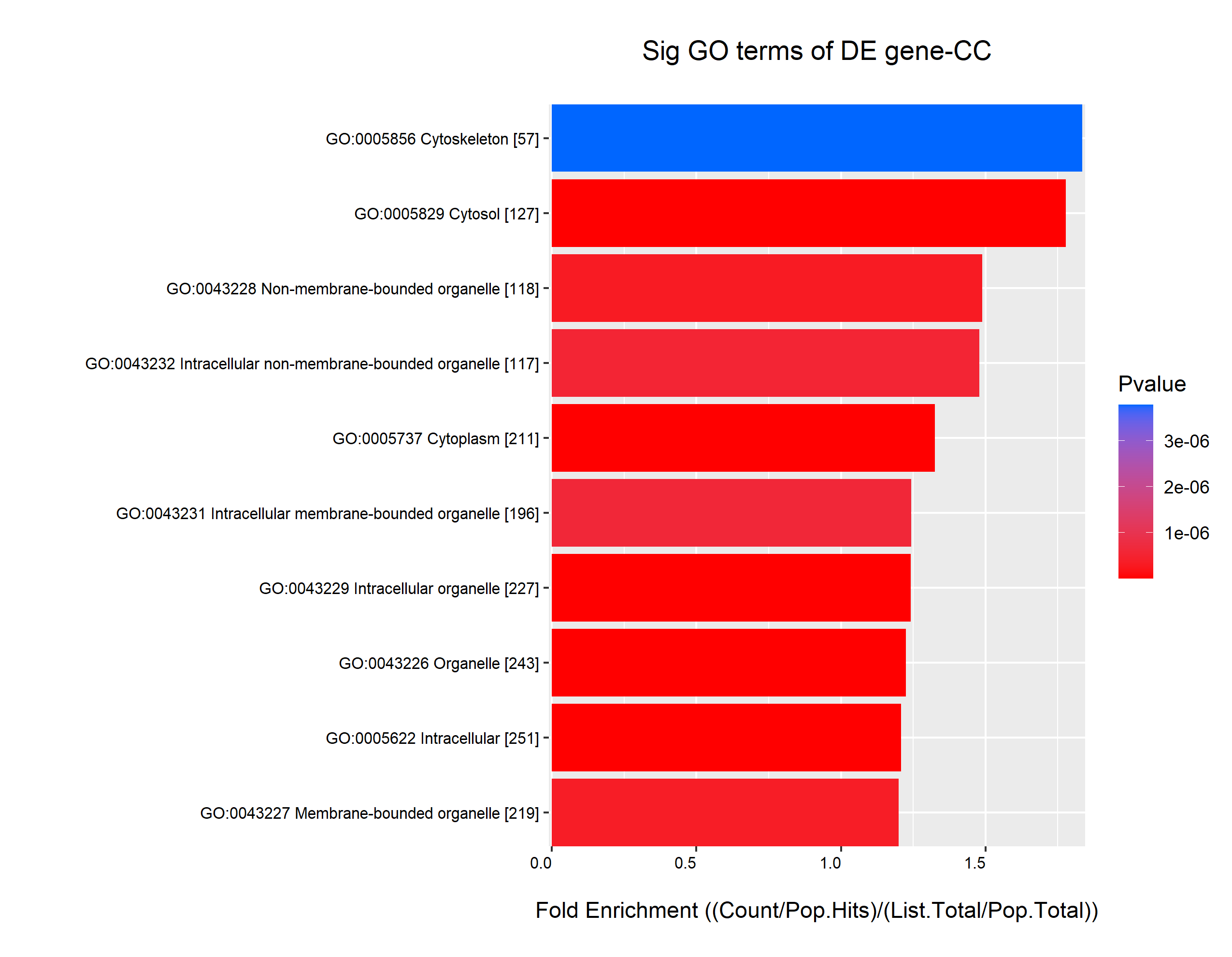

Supplement: Supplementary file 1 [file Data_Sheet_1.ZIP › Additional files/GO Analysis Report/GO_GC_vs_control_down/CC_FoldEnrichment.png]

# Sig GO terms of DE gene-CC

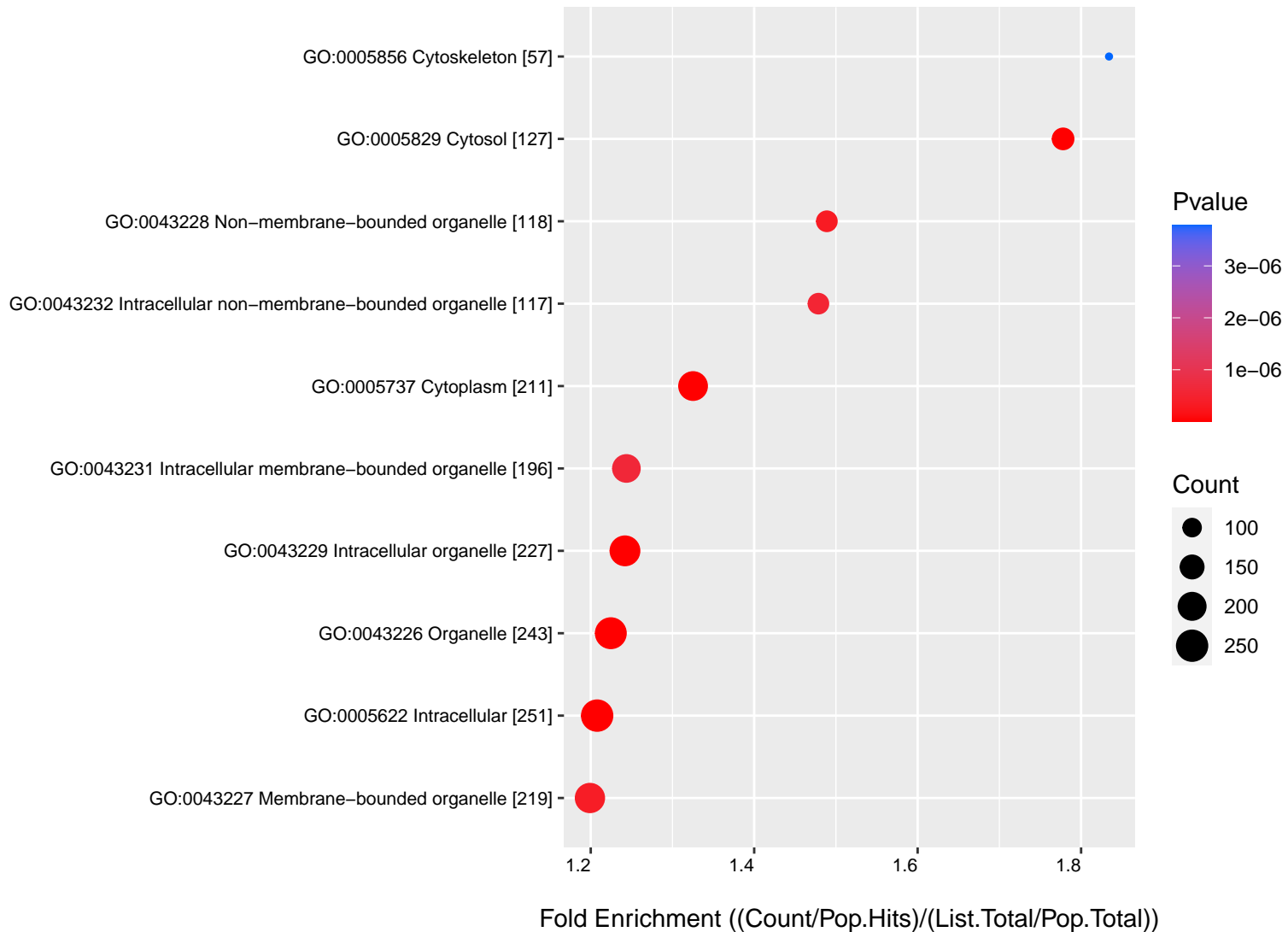

Supplement: Supplementary file 1 [file Data_Sheet_1.ZIP › Additional files/GO Analysis Report/GO_GC_vs_control_down/CC_FoldEnrichmentDotPlot.pdf]

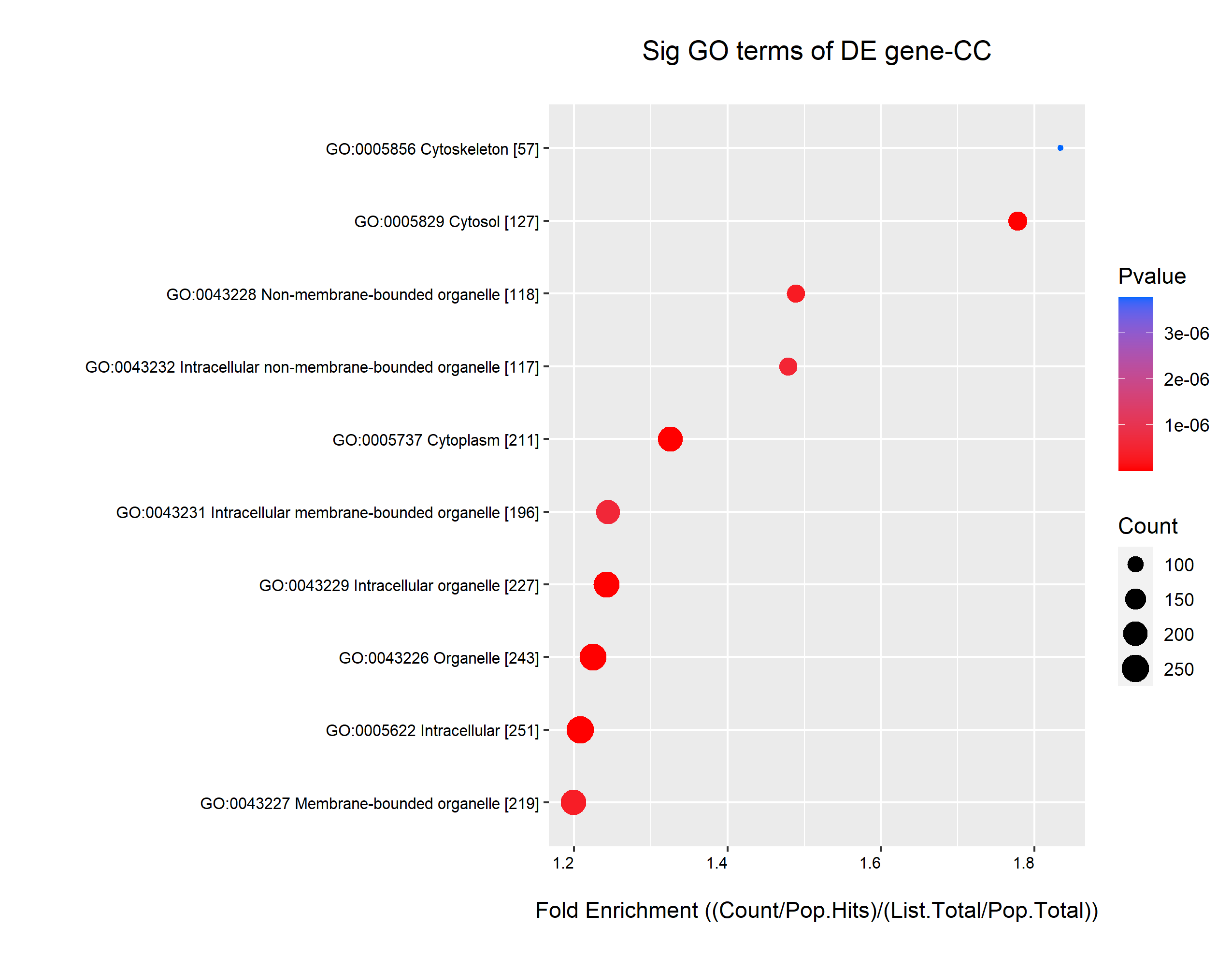

Supplement: Supplementary file 1 [file Data_Sheet_1.ZIP › Additional files/GO Analysis Report/GO_GC_vs_control_down/CC_FoldEnrichmentDotPlot.png]

## Sig GO terms of DE gene-CC

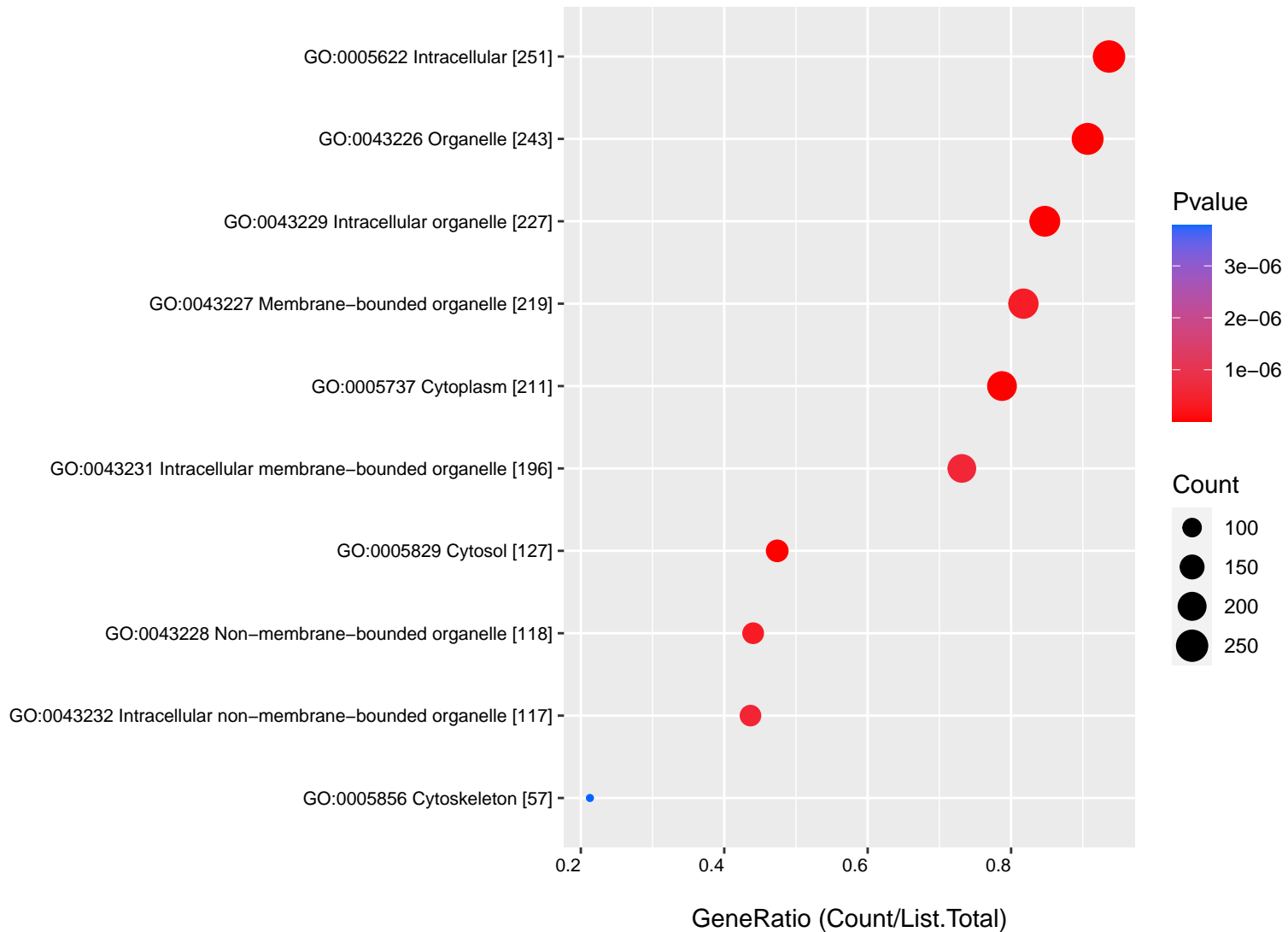

Supplement: Supplementary file 1 [file Data_Sheet_1.ZIP › Additional files/GO Analysis Report/GO_GC_vs_control_down/CC_GeneRatioDotPlot.pdf]

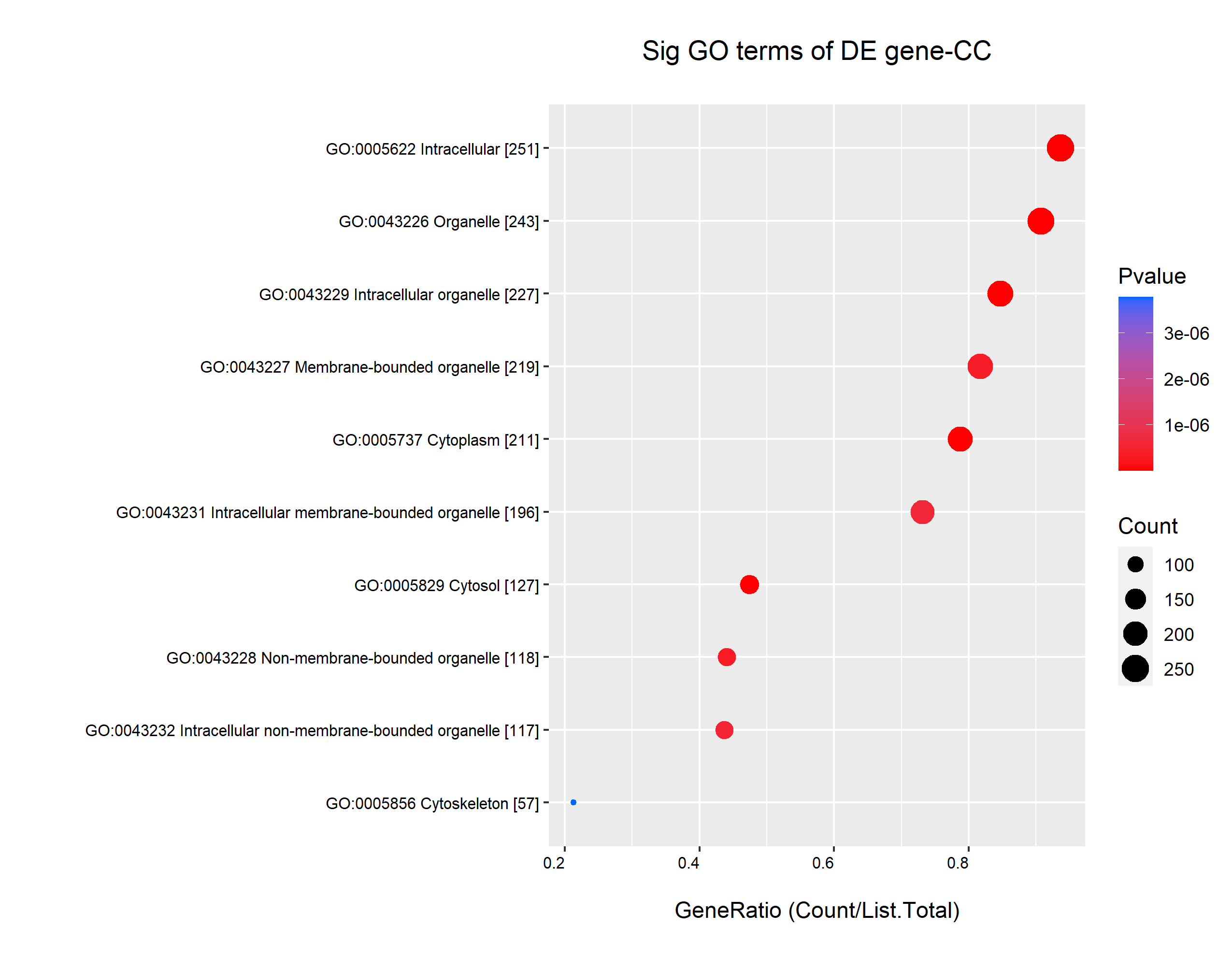

Supplement: Supplementary file 1 [file Data_Sheet_1.ZIP › Additional files/GO Analysis Report/GO_GC_vs_control_down/CC_GeneRatioDotPlot.png]

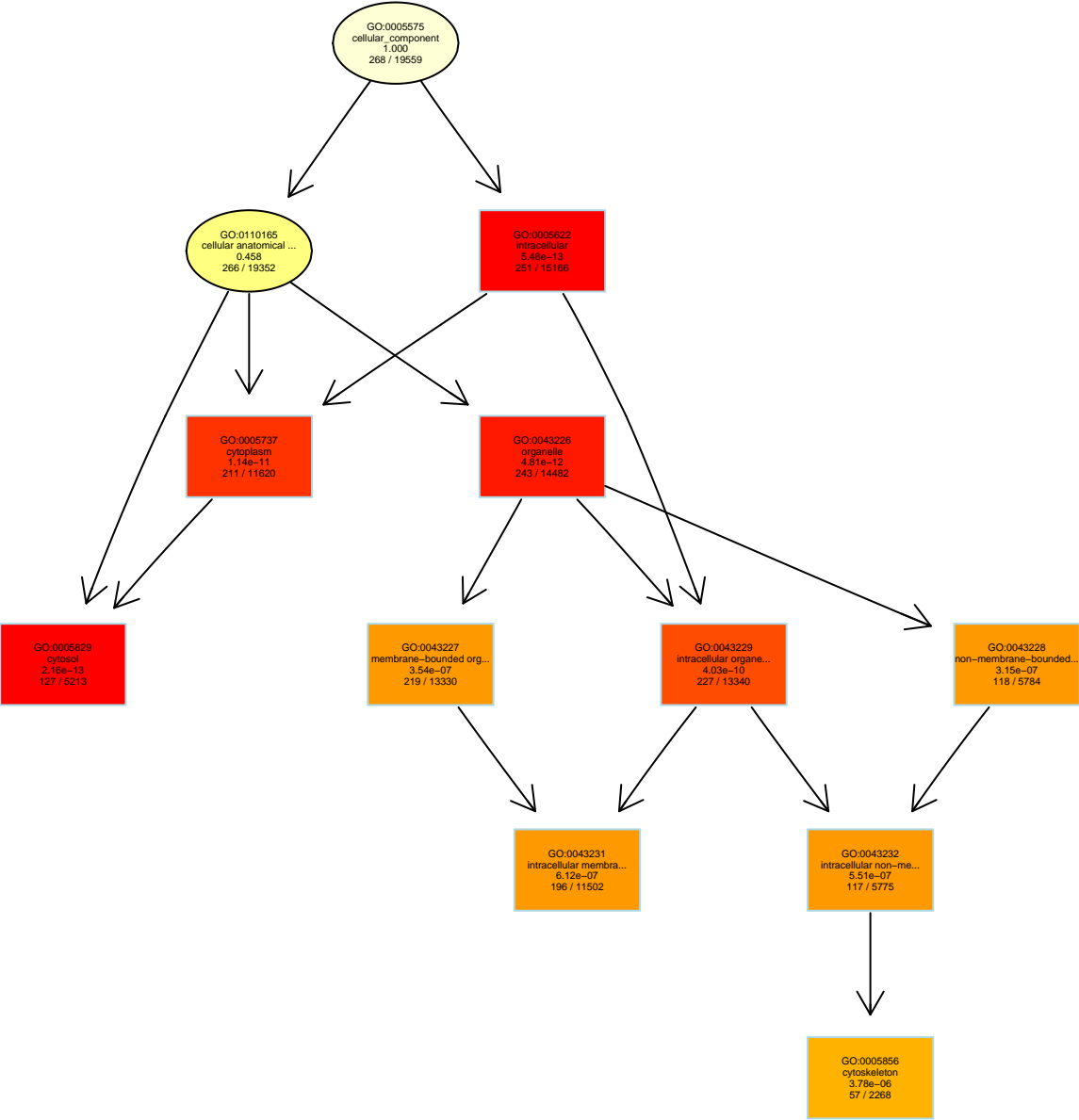

Supplement: Supplementary file 1 [file Data_Sheet_1.ZIP › Additional files/GO Analysis Report/GO_GC_vs_control_down/CC_Pvalue_tree.pdf]

Sig GO terms of DE gene

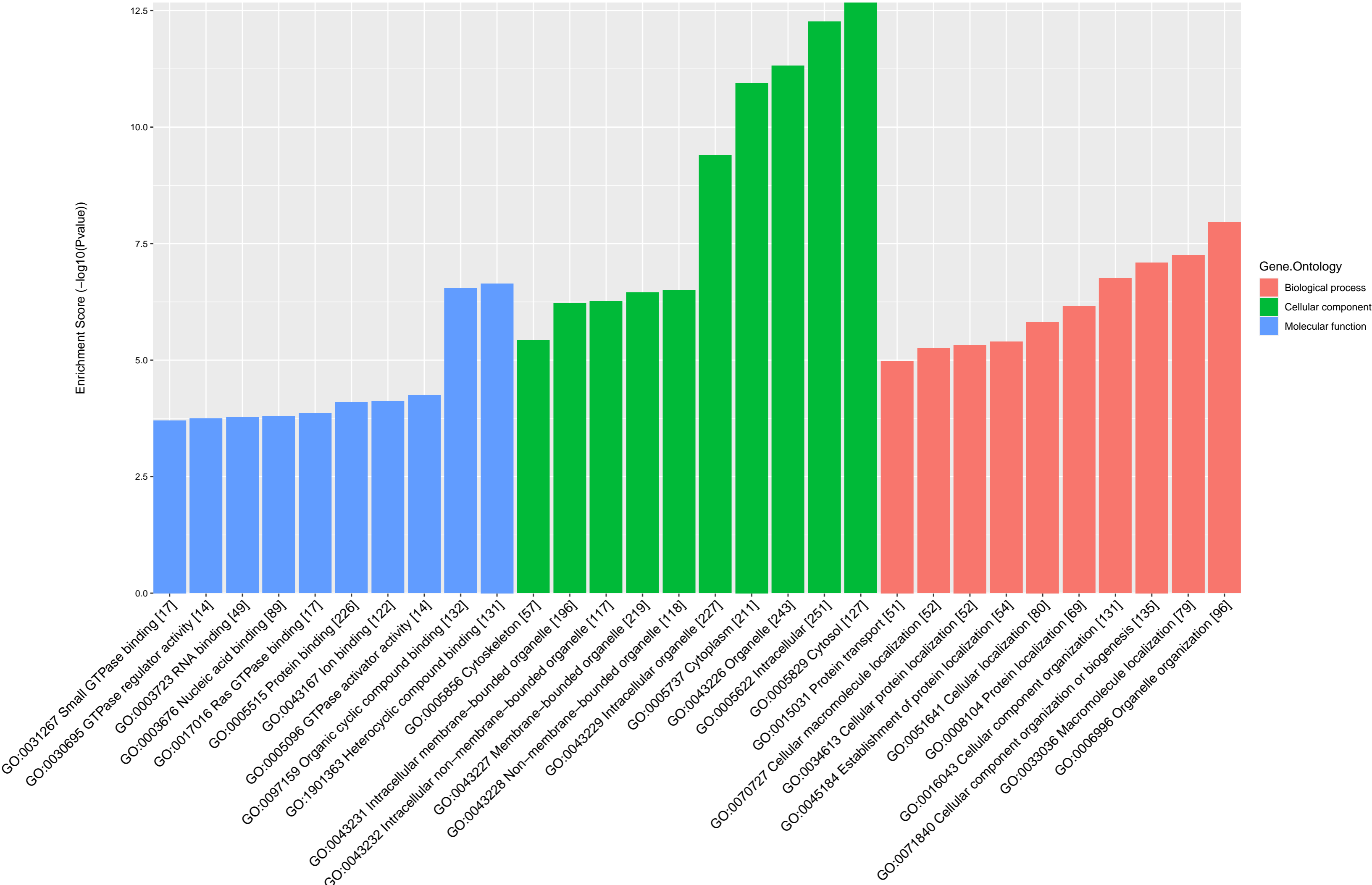

Supplement: Supplementary file 1 [file Data_Sheet_1.ZIP › Additional files/GO Analysis Report/GO_GC_vs_control_down/GeneOntology_EnrichmentScore.pdf]

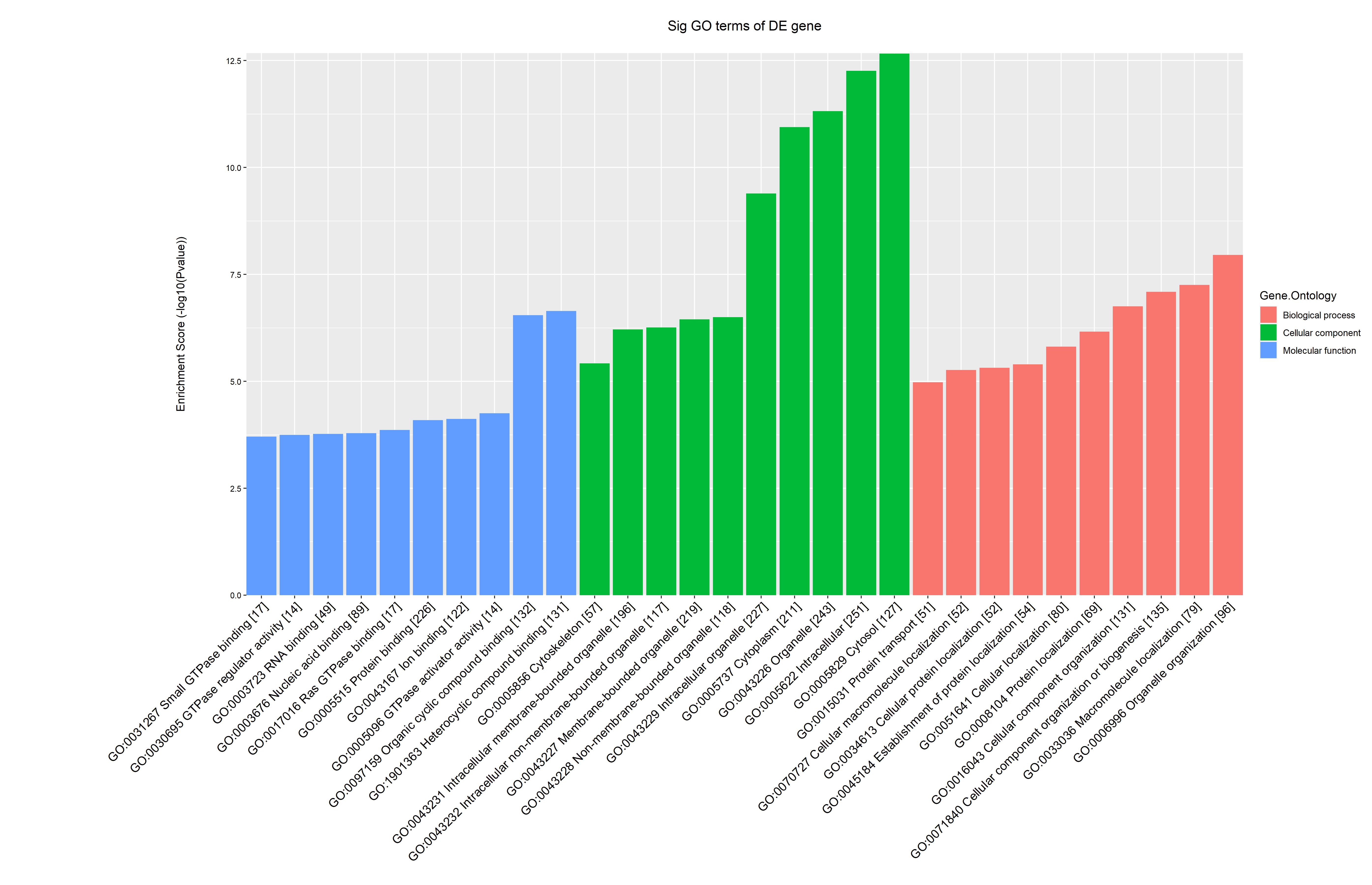

Supplement: Supplementary file 1 [file Data_Sheet_1.ZIP › Additional files/GO Analysis Report/GO_GC_vs_control_down/GeneOntology_EnrichmentScore.png]

Sig GO terms of DE gene

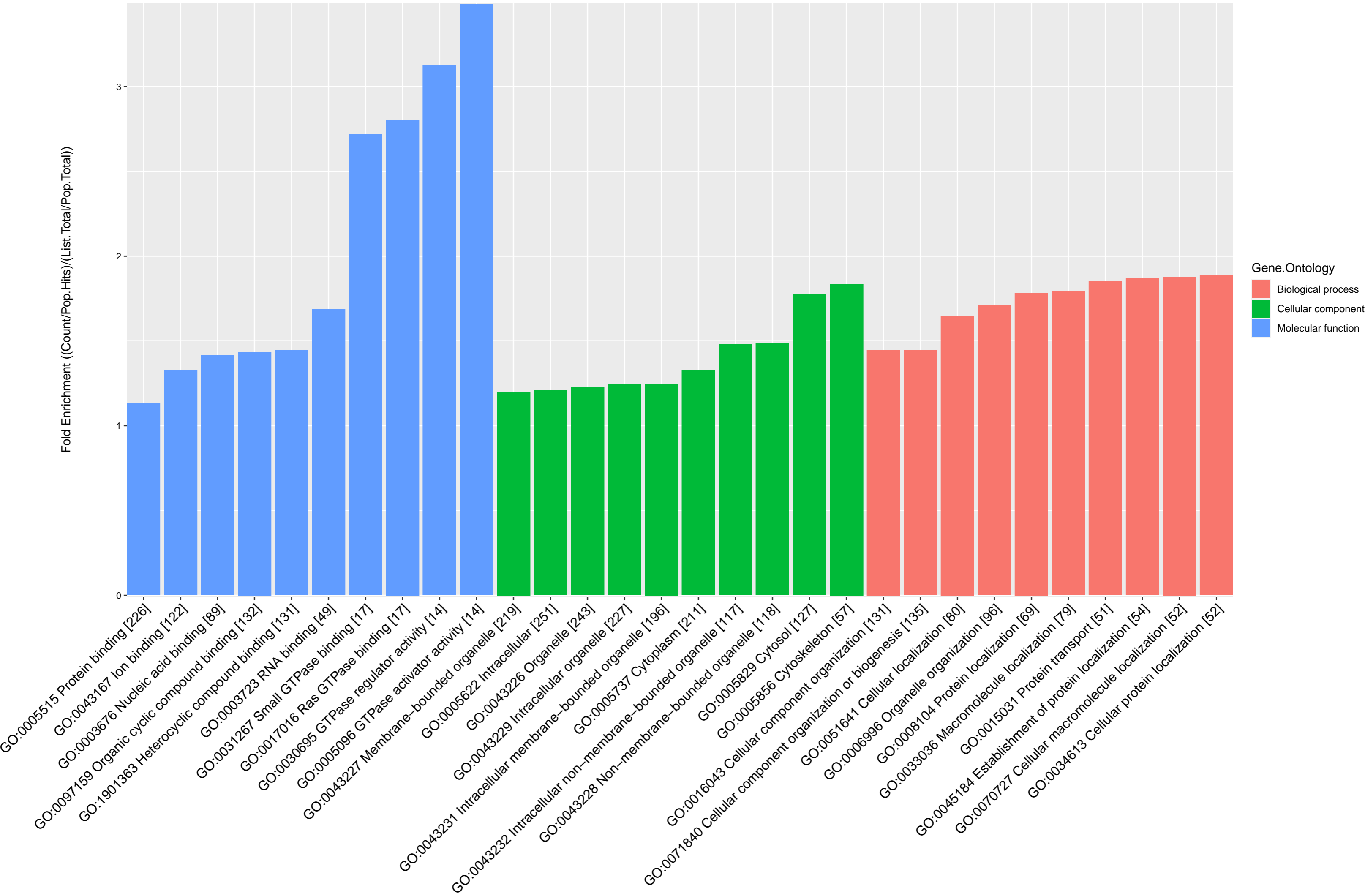

Supplement: Supplementary file 1 [file Data_Sheet_1.ZIP › Additional files/GO Analysis Report/GO_GC_vs_control_down/GeneOntology_FoldEnrichment.pdf]

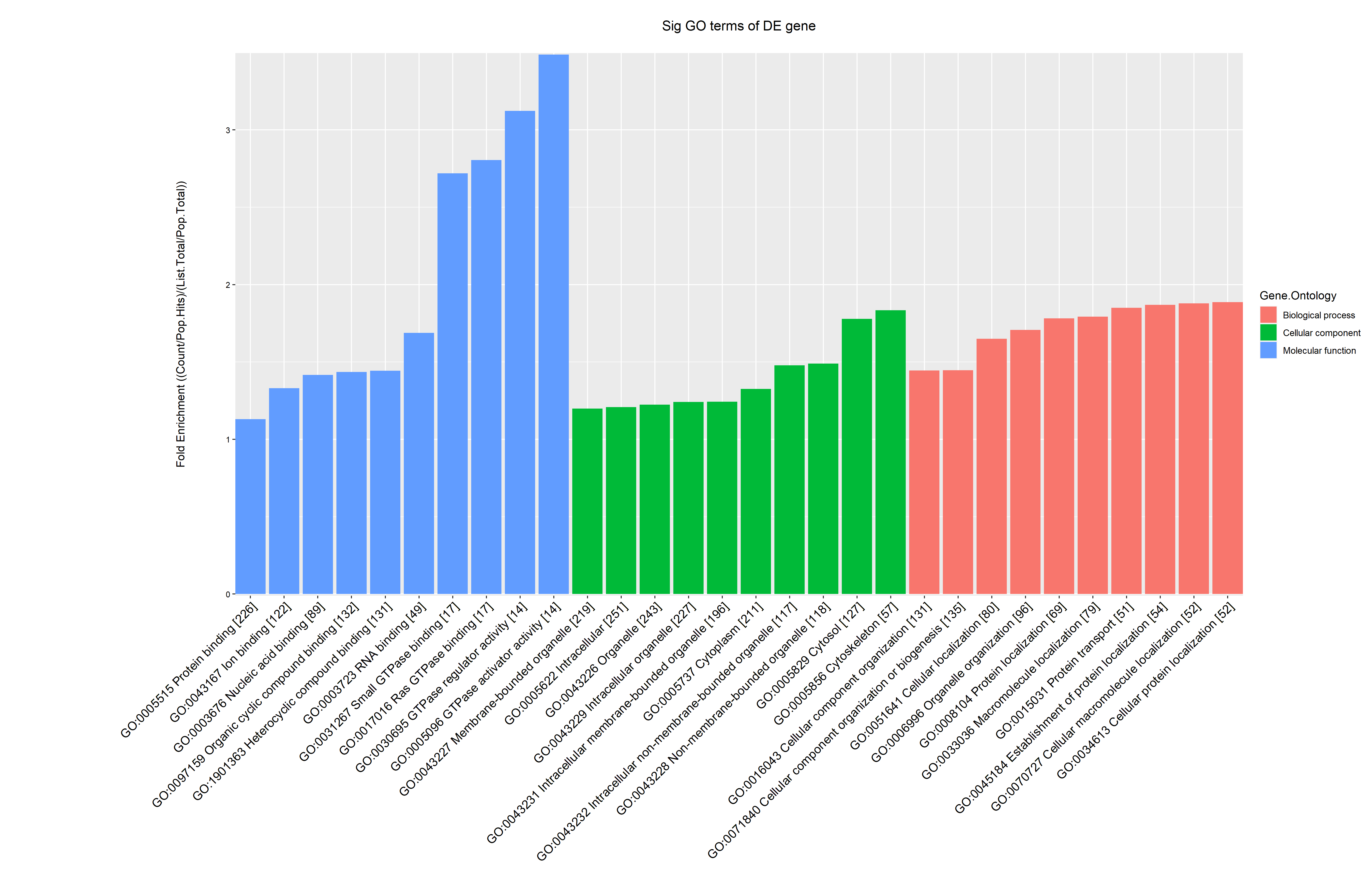

Supplement: Supplementary file 1 [file Data_Sheet_1.ZIP › Additional files/GO Analysis Report/GO_GC_vs_control_down/GeneOntology_FoldEnrichment.png]

# GO Molecular Function Classification

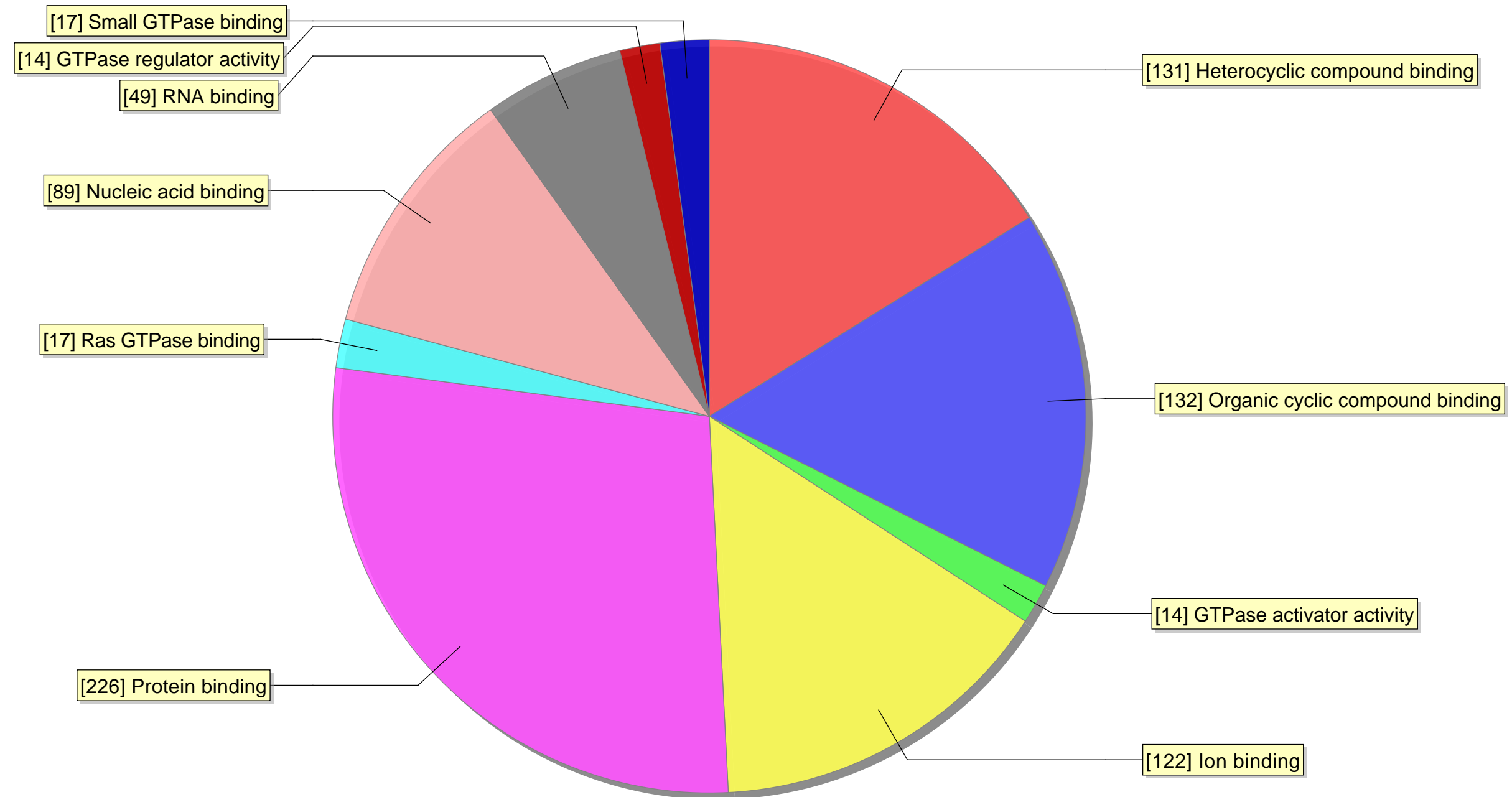

Supplement: Supplementary file 1 [file Data_Sheet_1.ZIP › Additional files/GO Analysis Report/GO_GC_vs_control_down/MF_Count.pdf]

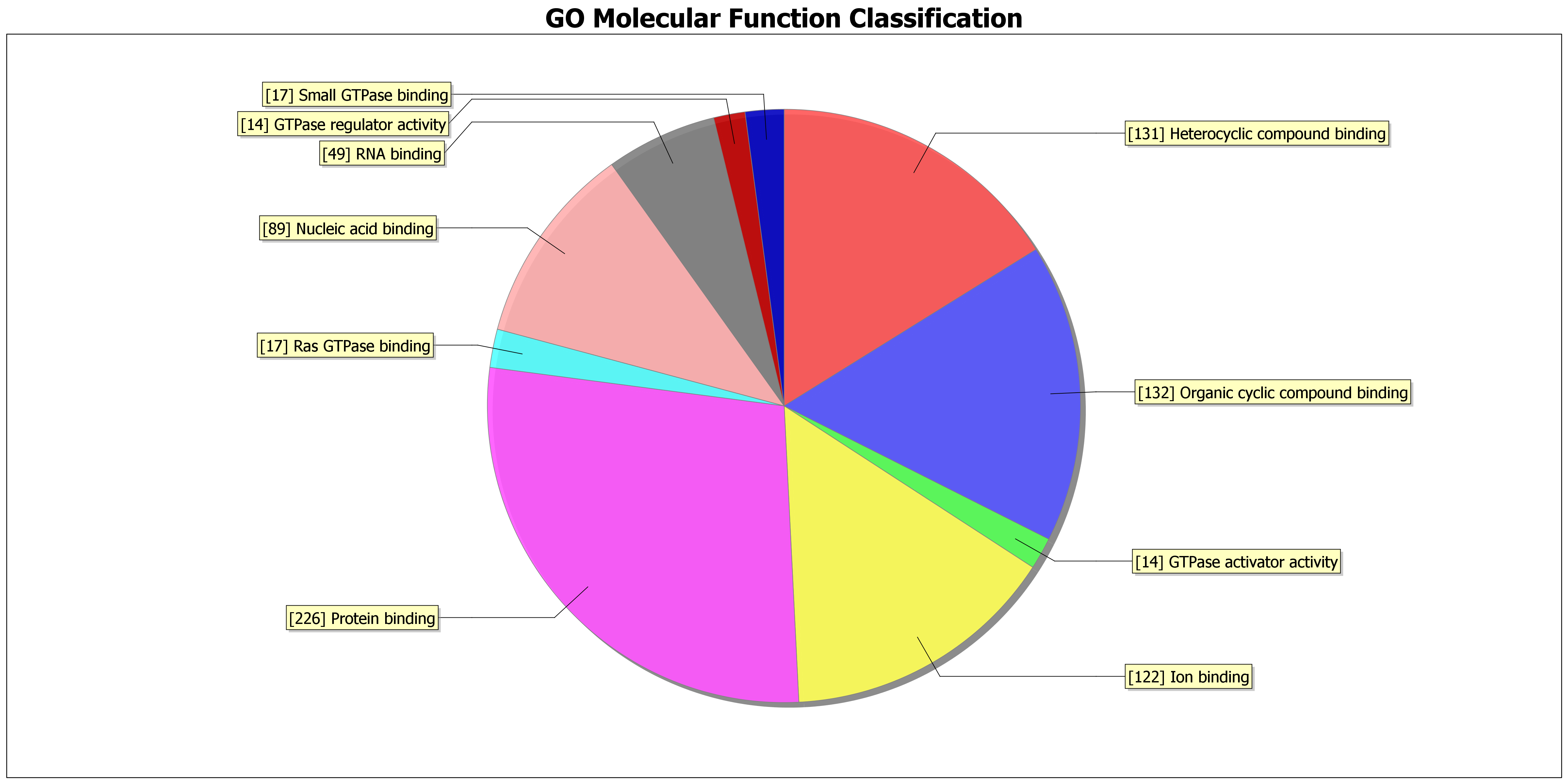

Supplement: Supplementary file 1 [file Data_Sheet_1.ZIP › Additional files/GO Analysis Report/GO_GC_vs_control_down/MF_Count.png]

## Sig GO terms of DE gene-MF

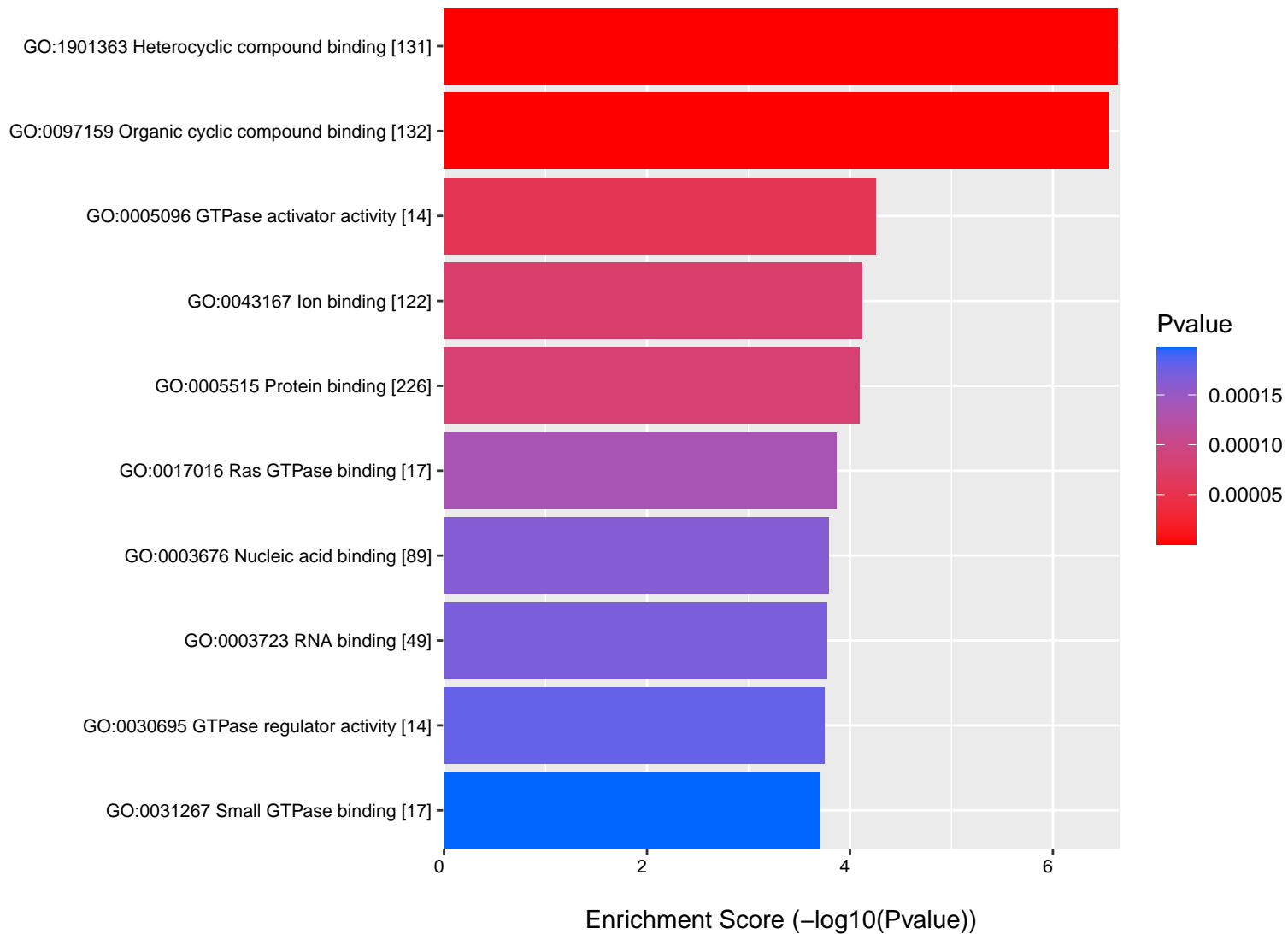

Supplement: Supplementary file 1 [file Data_Sheet_1.ZIP › Additional files/GO Analysis Report/GO_GC_vs_control_down/MF_EnrichmentScore.pdf]

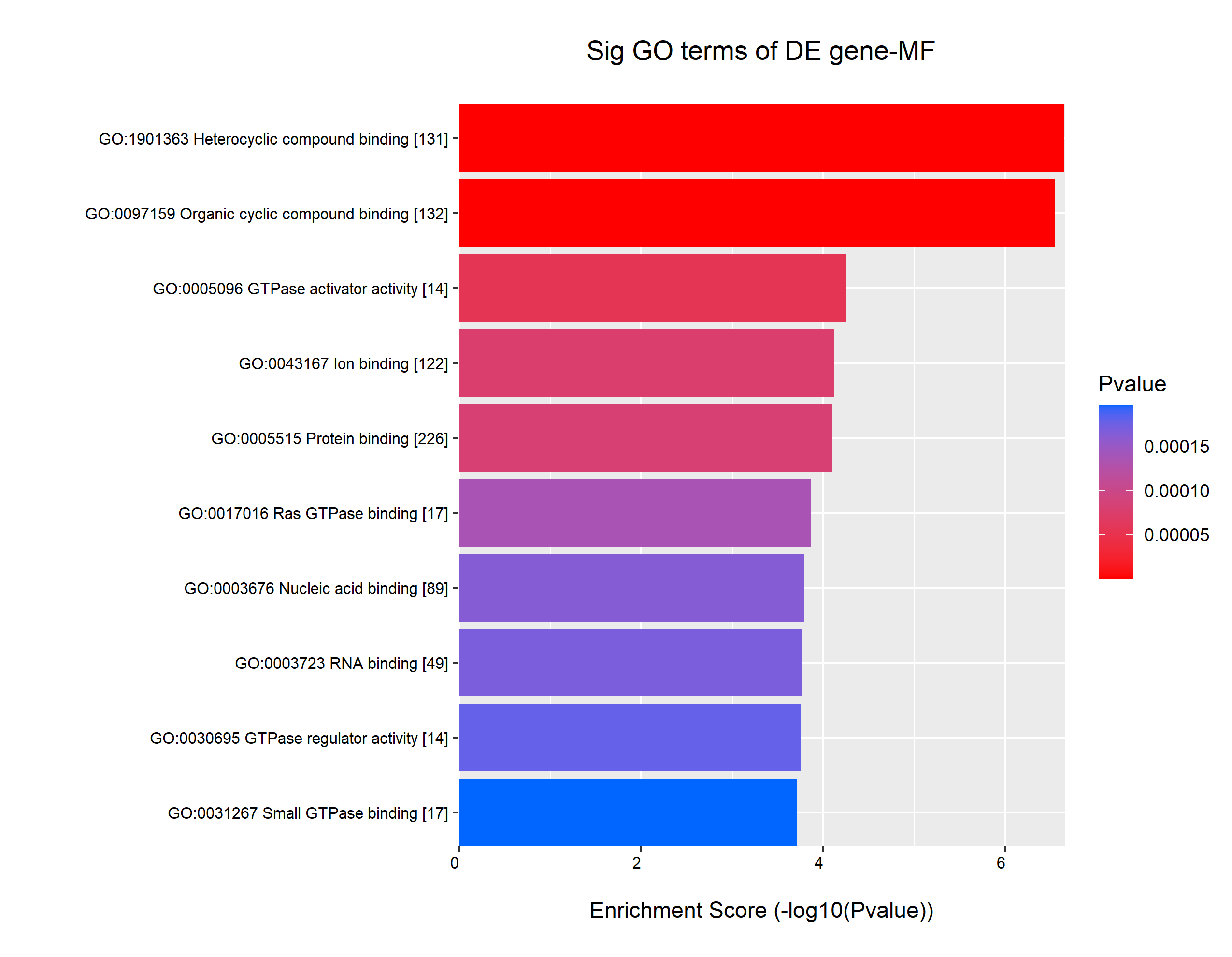

Supplement: Supplementary file 1 [file Data_Sheet_1.ZIP › Additional files/GO Analysis Report/GO_GC_vs_control_down/MF_EnrichmentScore.png]

## Sig GO terms of DE gene–MF

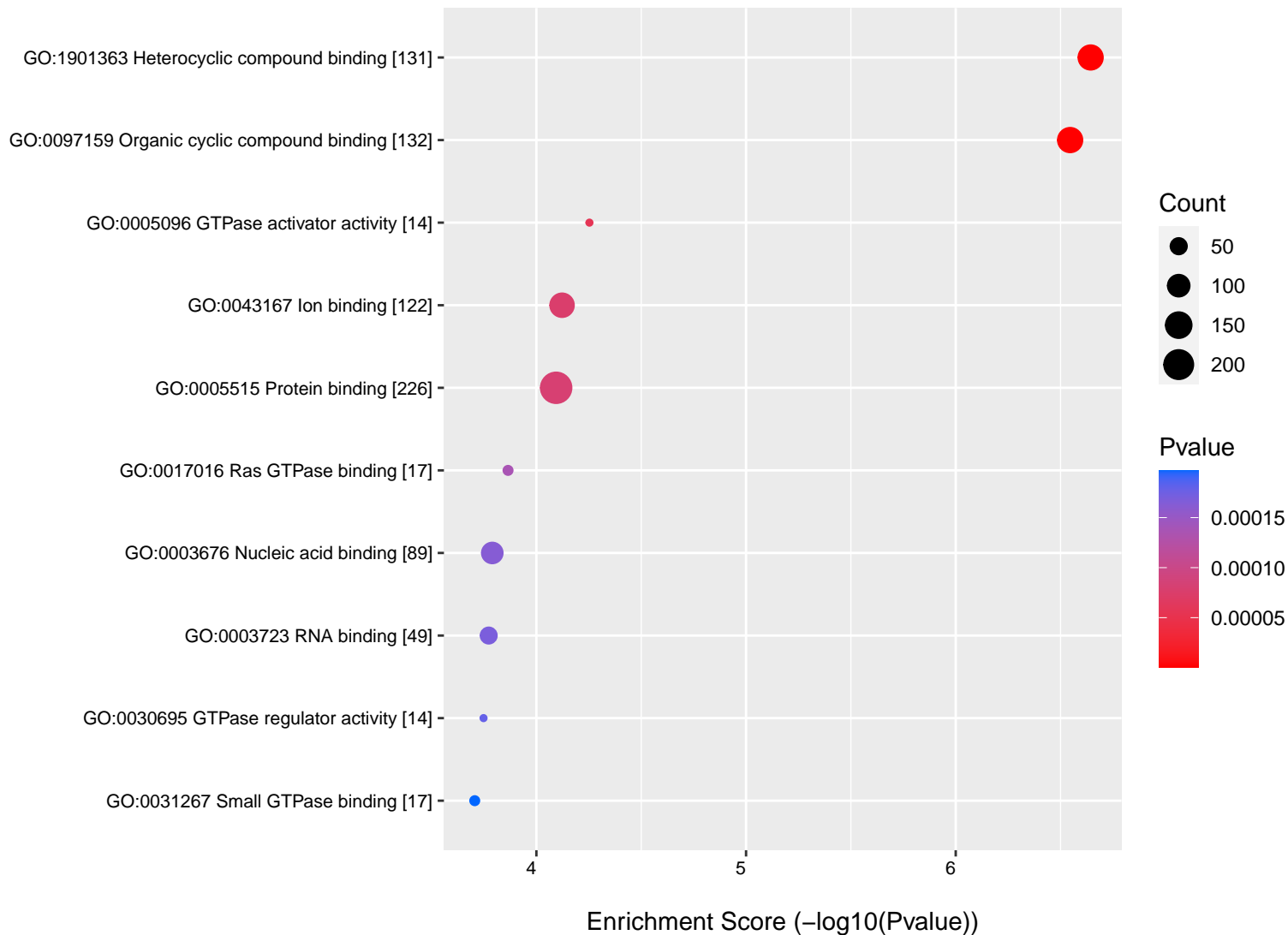

Supplement: Supplementary file 1 [file Data_Sheet_1.ZIP › Additional files/GO Analysis Report/GO_GC_vs_control_down/MF_EnrichmentScoreDotPlot.pdf]

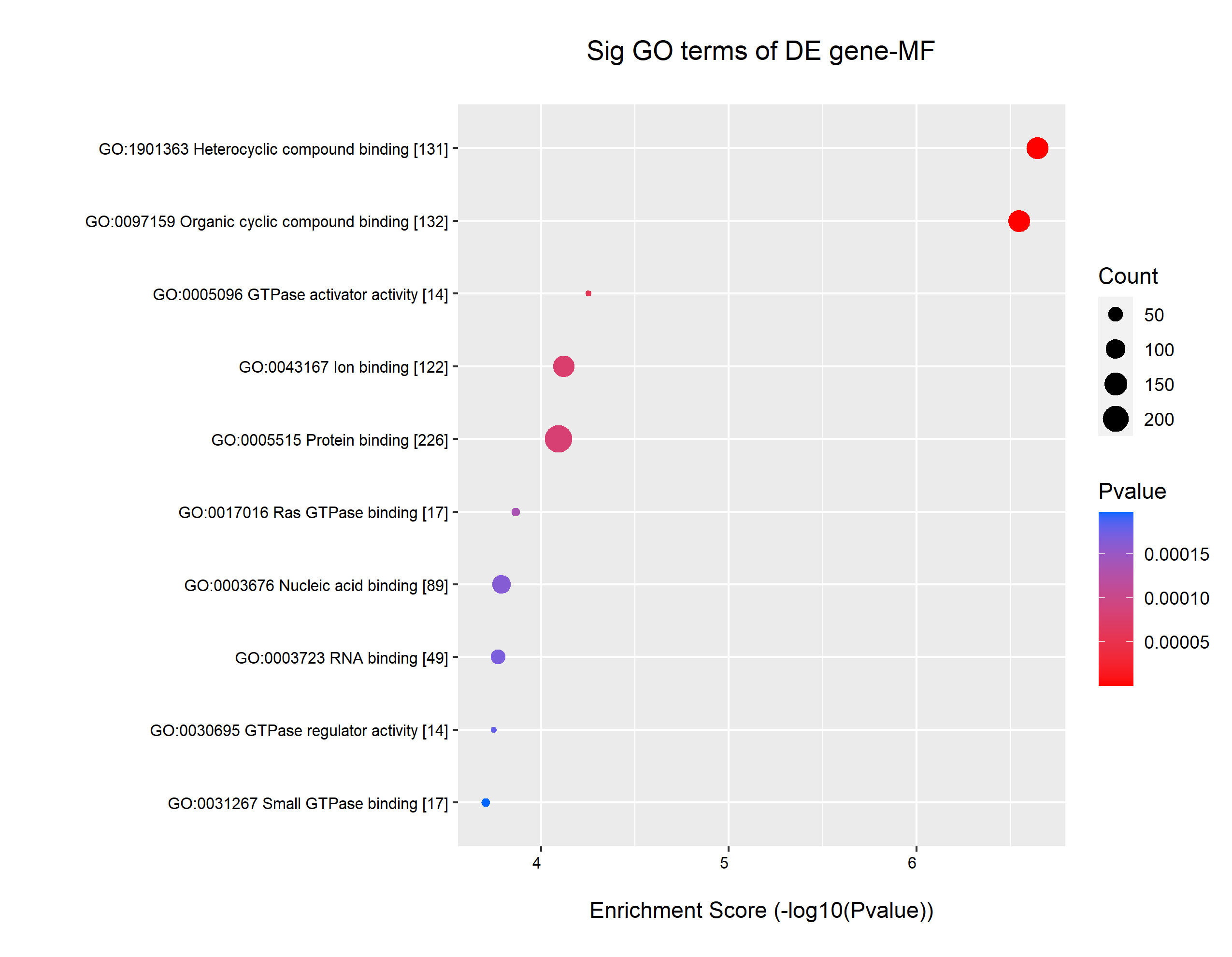

Supplement: Supplementary file 1 [file Data_Sheet_1.ZIP › Additional files/GO Analysis Report/GO_GC_vs_control_down/MF_EnrichmentScoreDotPlot.png]

## Sig GO terms of DE gene–MF

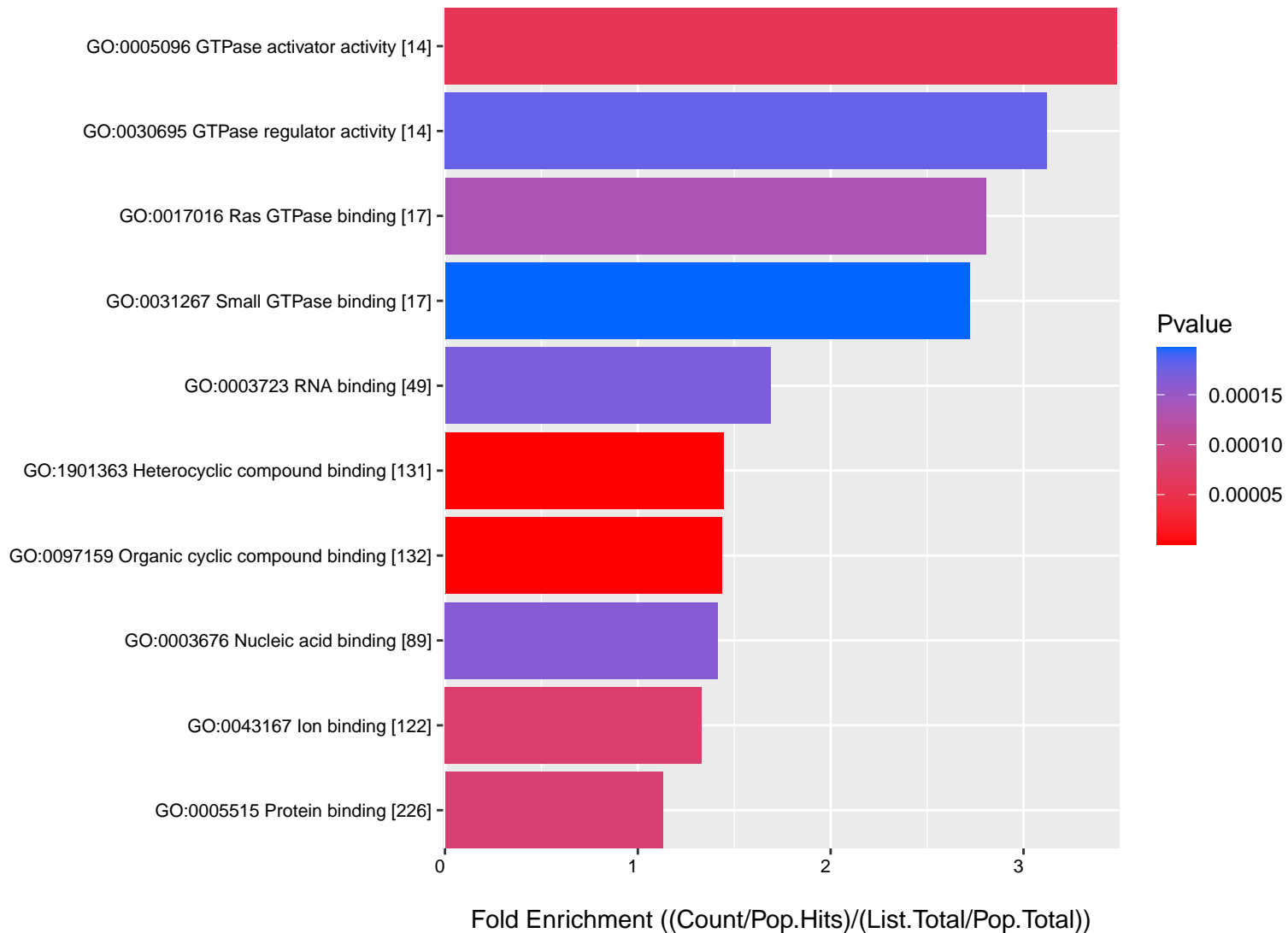

Supplement: Supplementary file 1 [file Data_Sheet_1.ZIP › Additional files/GO Analysis Report/GO_GC_vs_control_down/MF_FoldEnrichment.pdf]

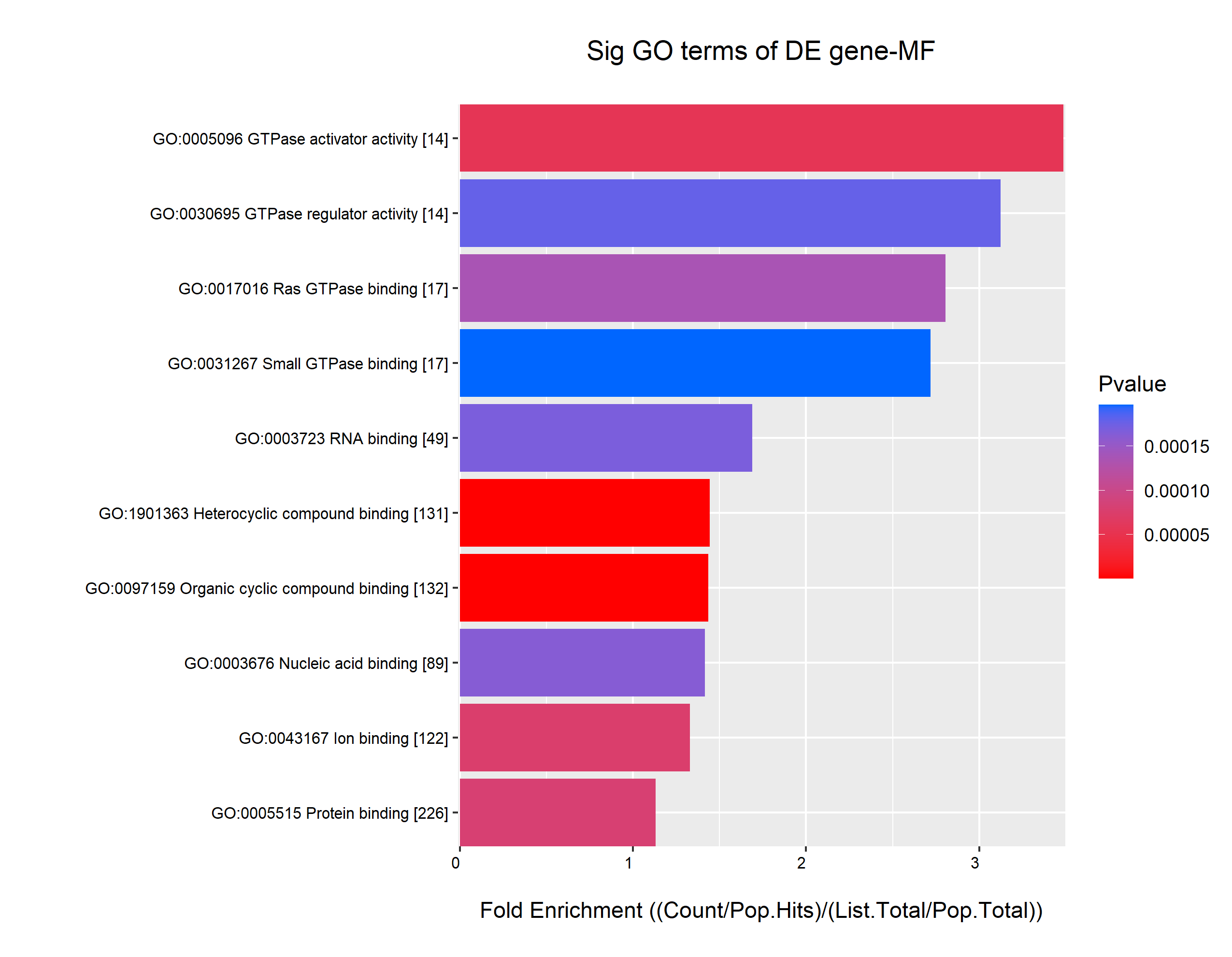

Supplement: Supplementary file 1 [file Data_Sheet_1.ZIP › Additional files/GO Analysis Report/GO_GC_vs_control_down/MF_FoldEnrichment.png]

## Sig GO terms of DE gene-MF

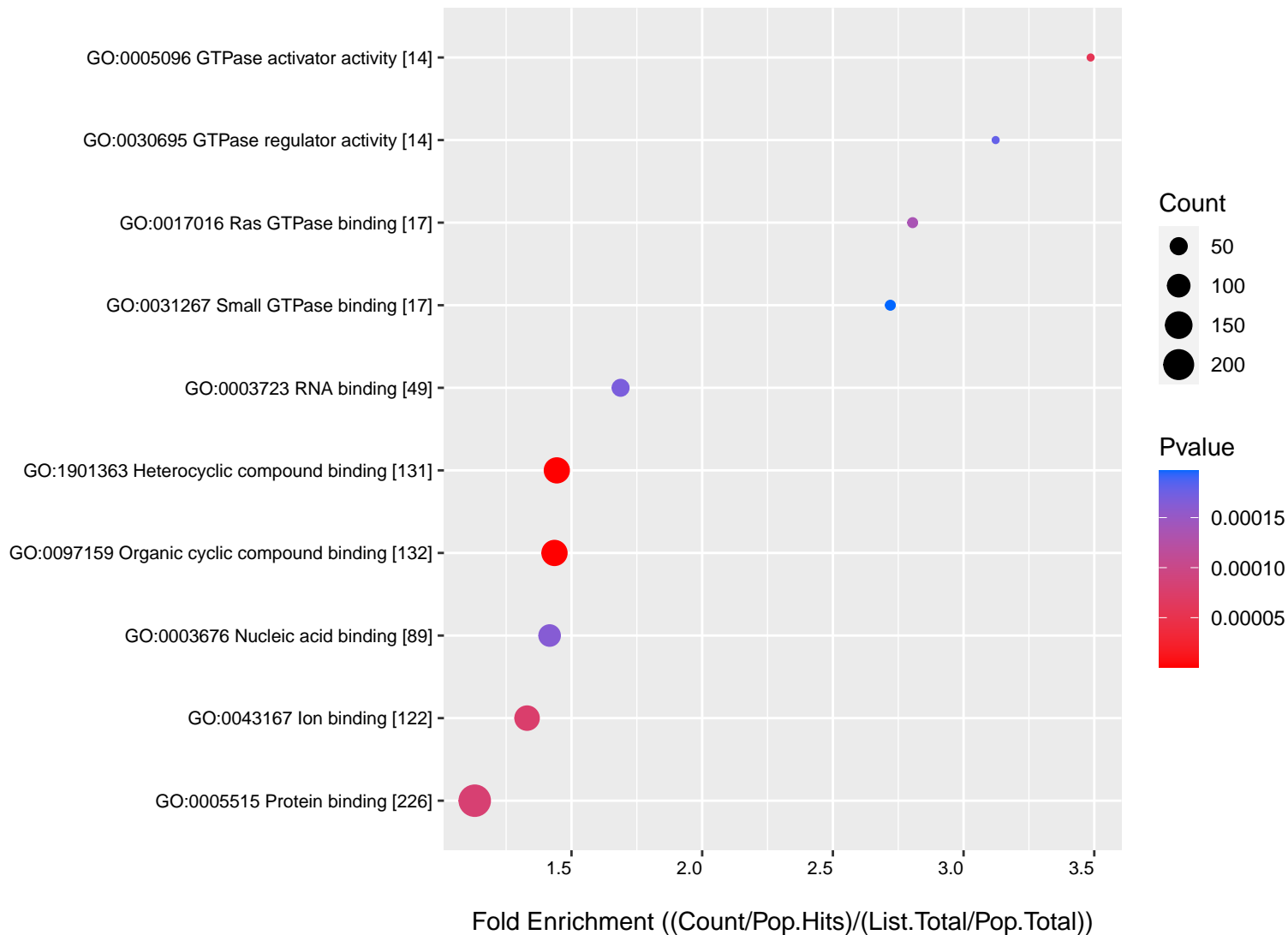

Supplement: Supplementary file 1 [file Data_Sheet_1.ZIP › Additional files/GO Analysis Report/GO_GC_vs_control_down/MF_FoldEnrichmentDotPlot.pdf]

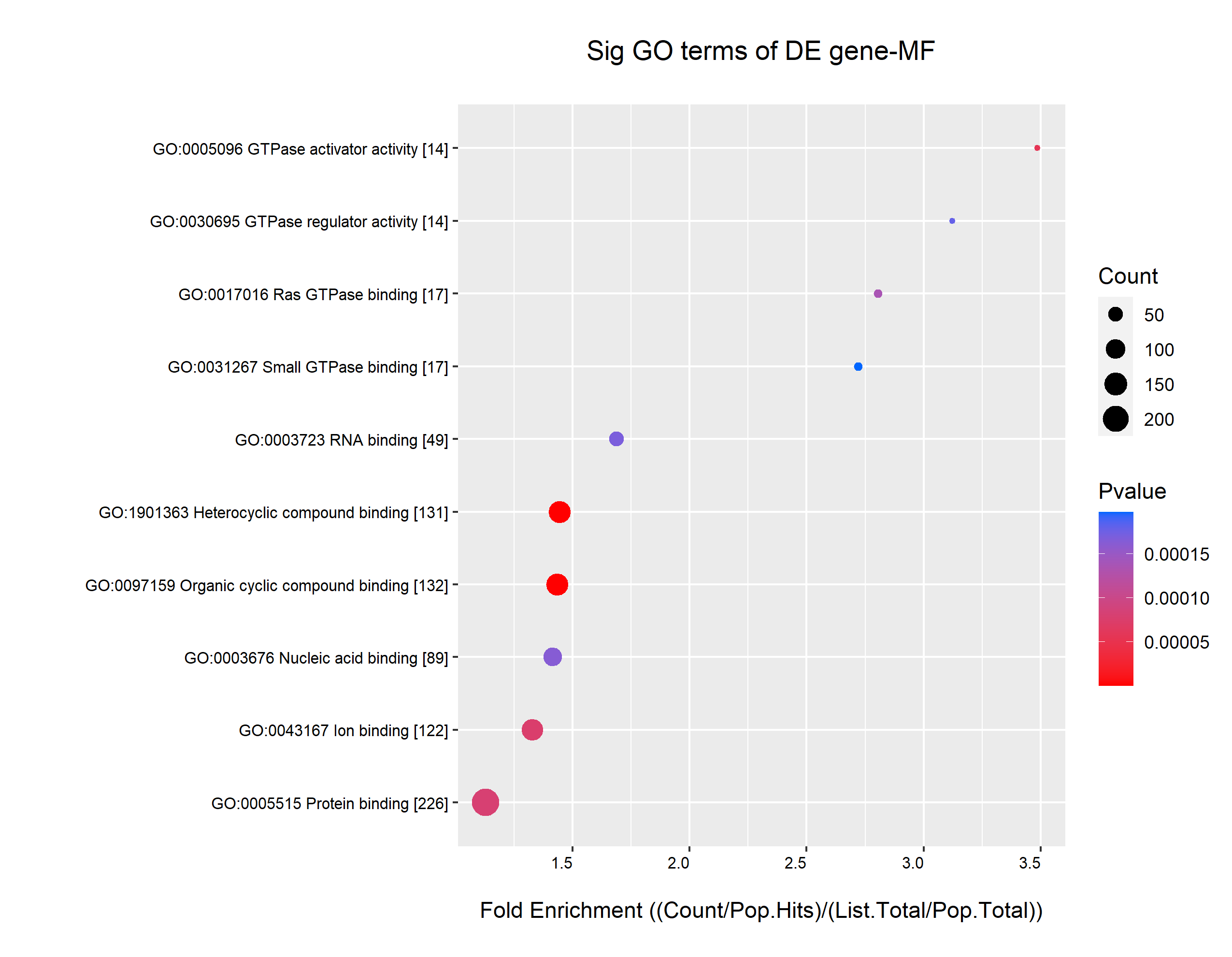

Supplement: Supplementary file 1 [file Data_Sheet_1.ZIP › Additional files/GO Analysis Report/GO_GC_vs_control_down/MF_FoldEnrichmentDotPlot.png]

## Sig GO terms of DE gene-MF

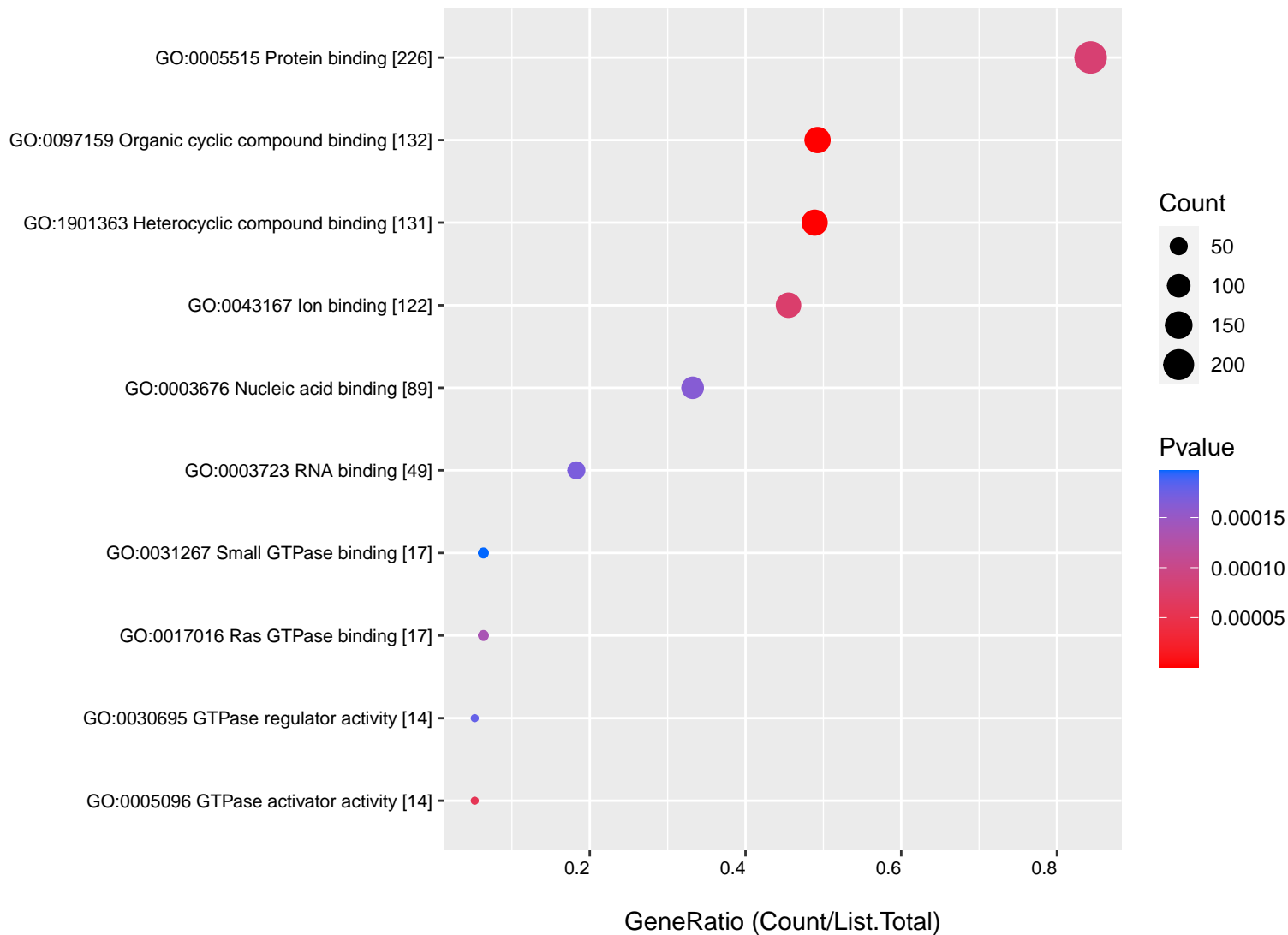

Supplement: Supplementary file 1 [file Data_Sheet_1.ZIP › Additional files/GO Analysis Report/GO_GC_vs_control_down/MF_GeneRatioDotPlot.pdf]

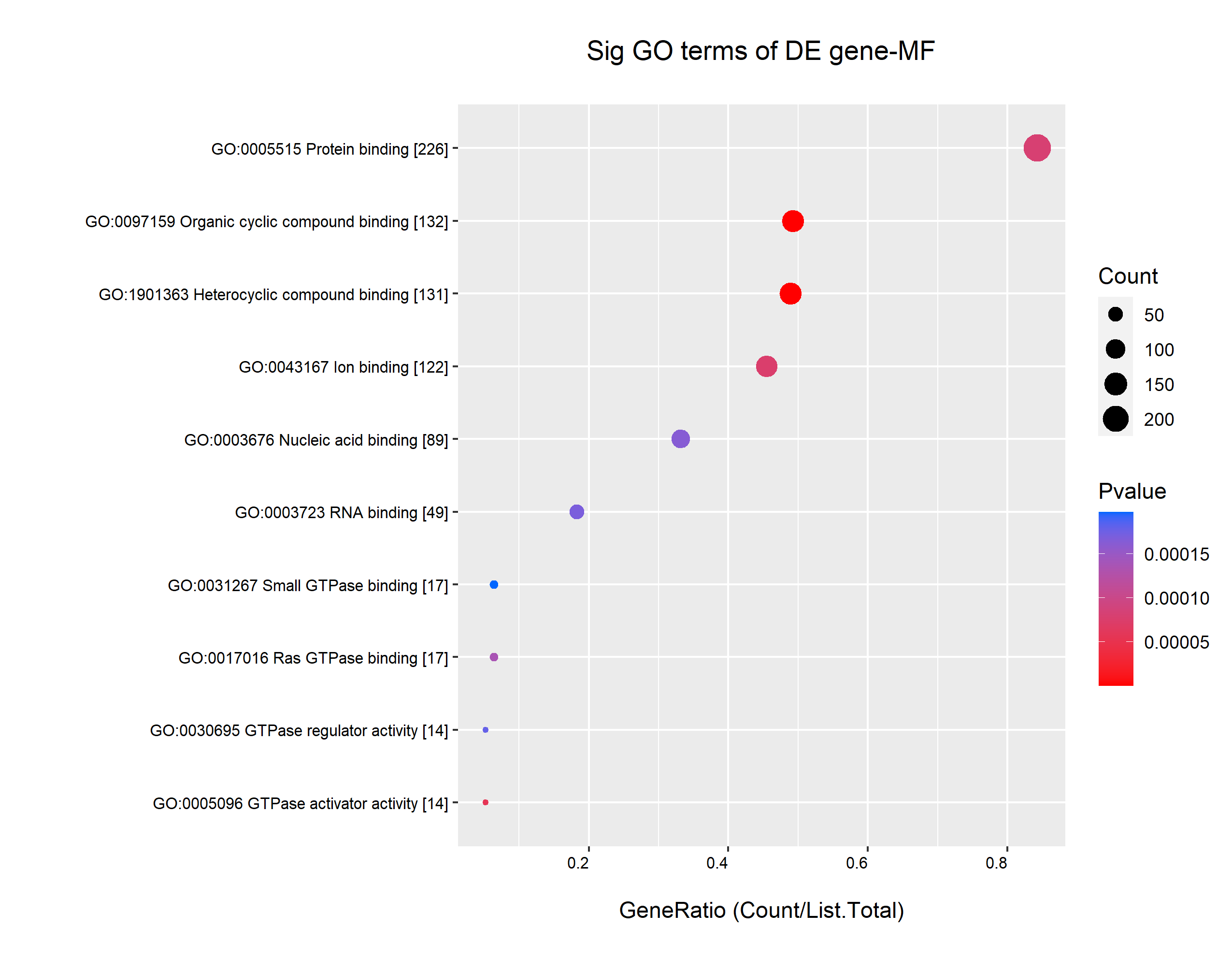

Supplement: Supplementary file 1 [file Data_Sheet_1.ZIP › Additional files/GO Analysis Report/GO_GC_vs_control_down/MF_GeneRatioDotPlot.png]

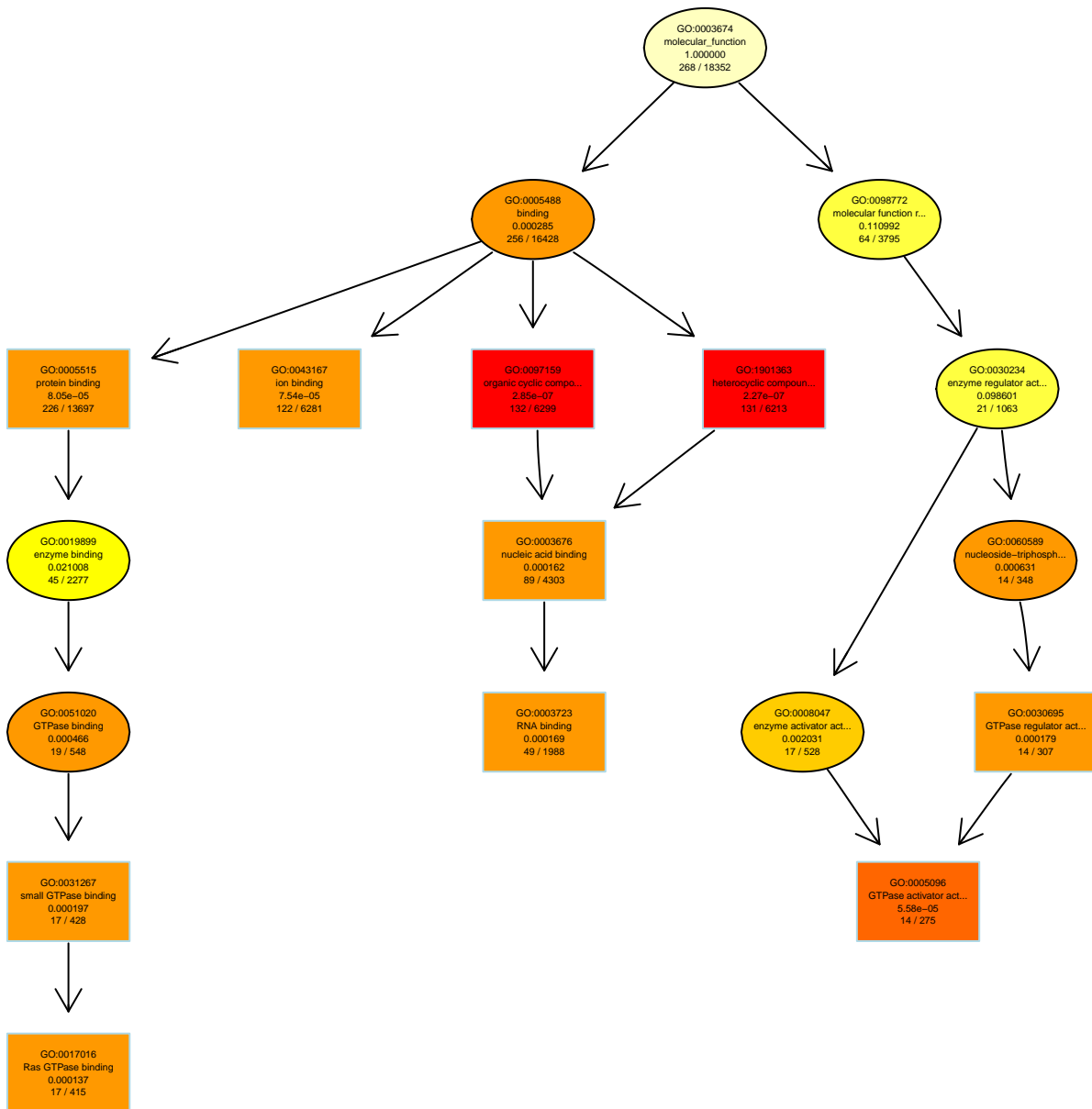

Supplement: Supplementary file 1 [file Data_Sheet_1.ZIP › Additional files/GO Analysis Report/GO_GC_vs_control_down/MF_Pvalue_tree.pdf]

# GO Biological Process Classification

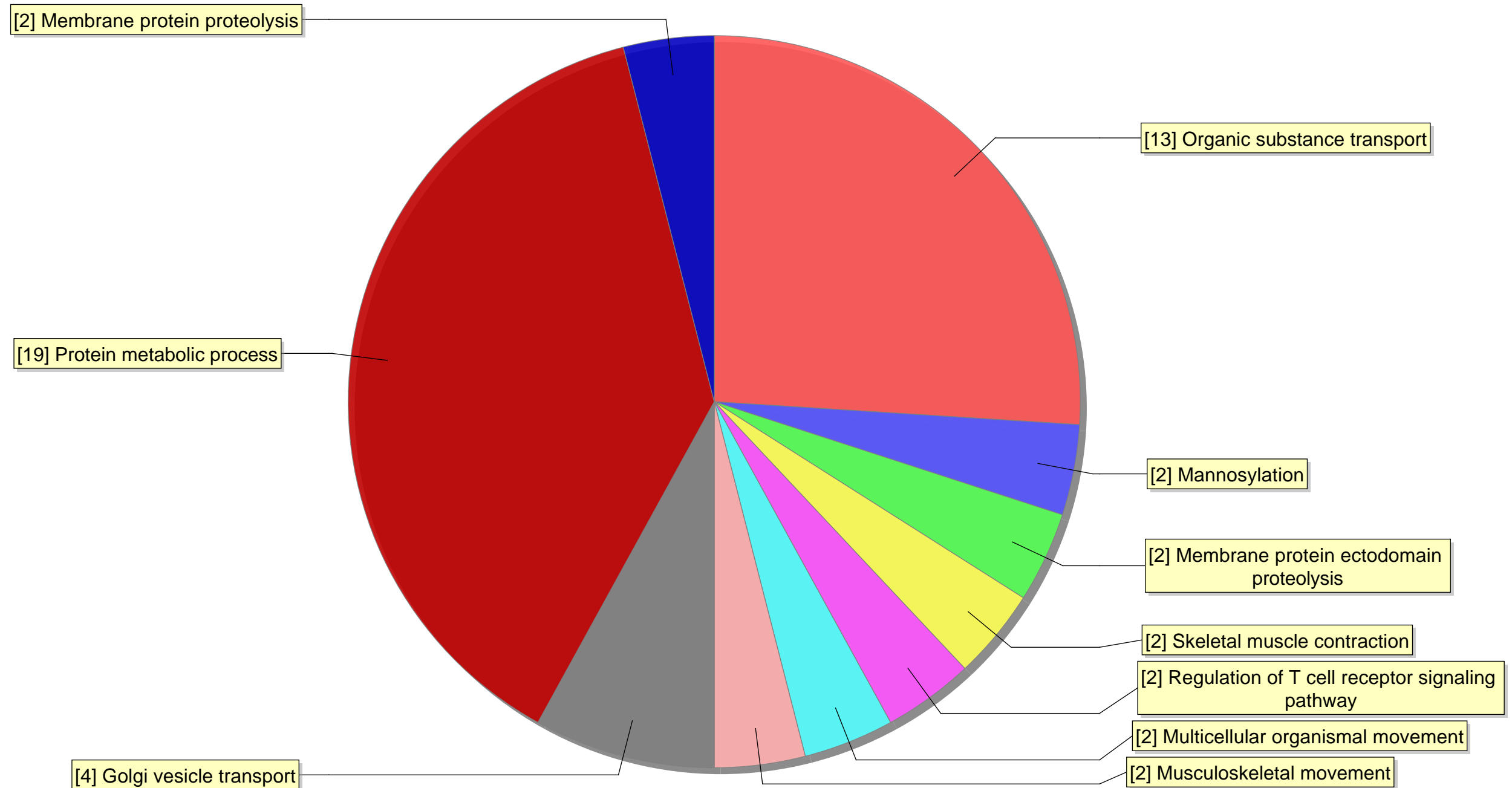

Supplement: Supplementary file 1 [file Data_Sheet_1.ZIP › Additional files/GO Analysis Report/GO_GC_vs_control_up/BP_Count.pdf]

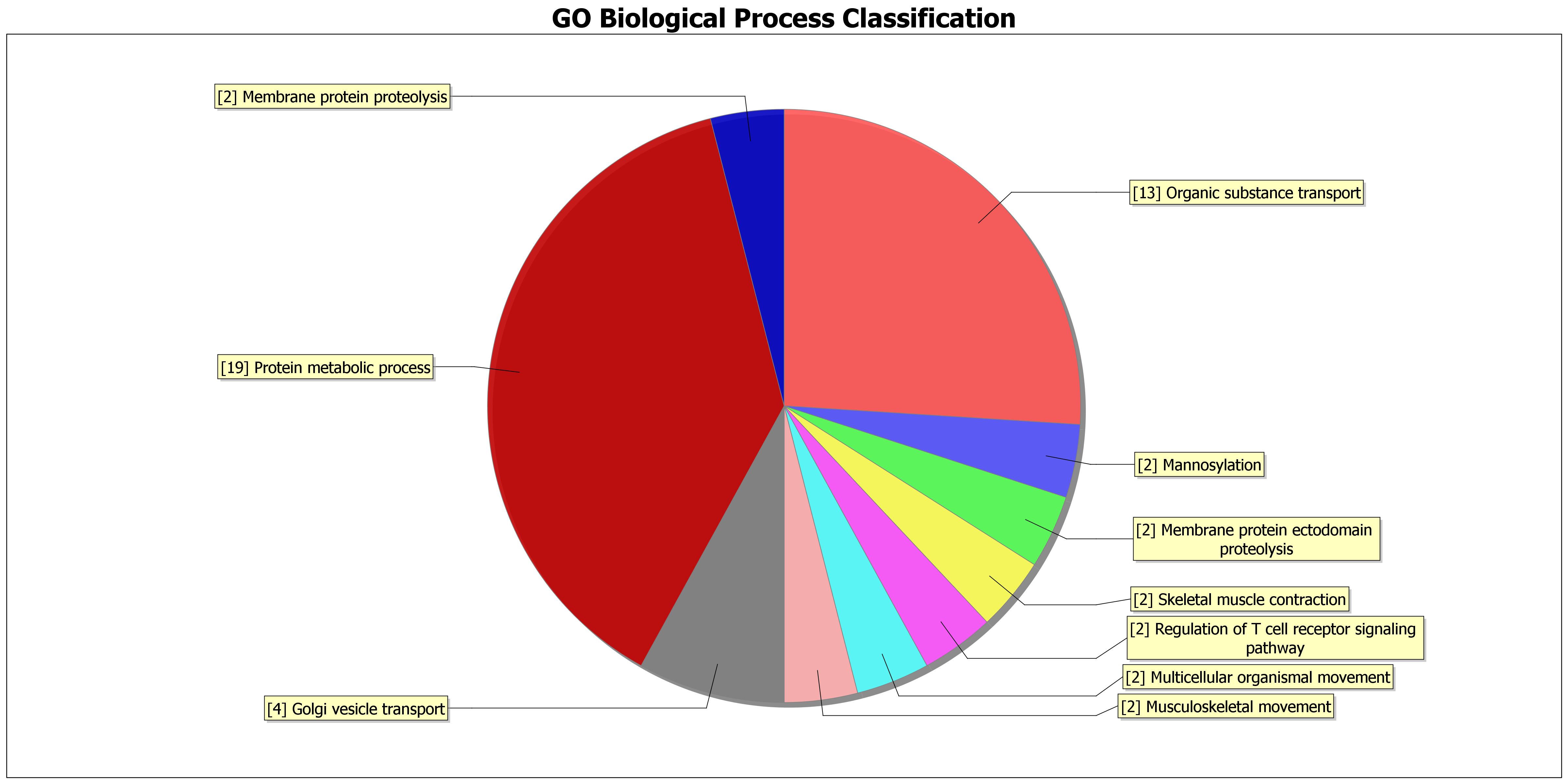

Supplement: Supplementary file 1 [file Data_Sheet_1.ZIP › Additional files/GO Analysis Report/GO_GC_vs_control_up/BP_Count.png]

## Sig GO terms of DE gene-BP

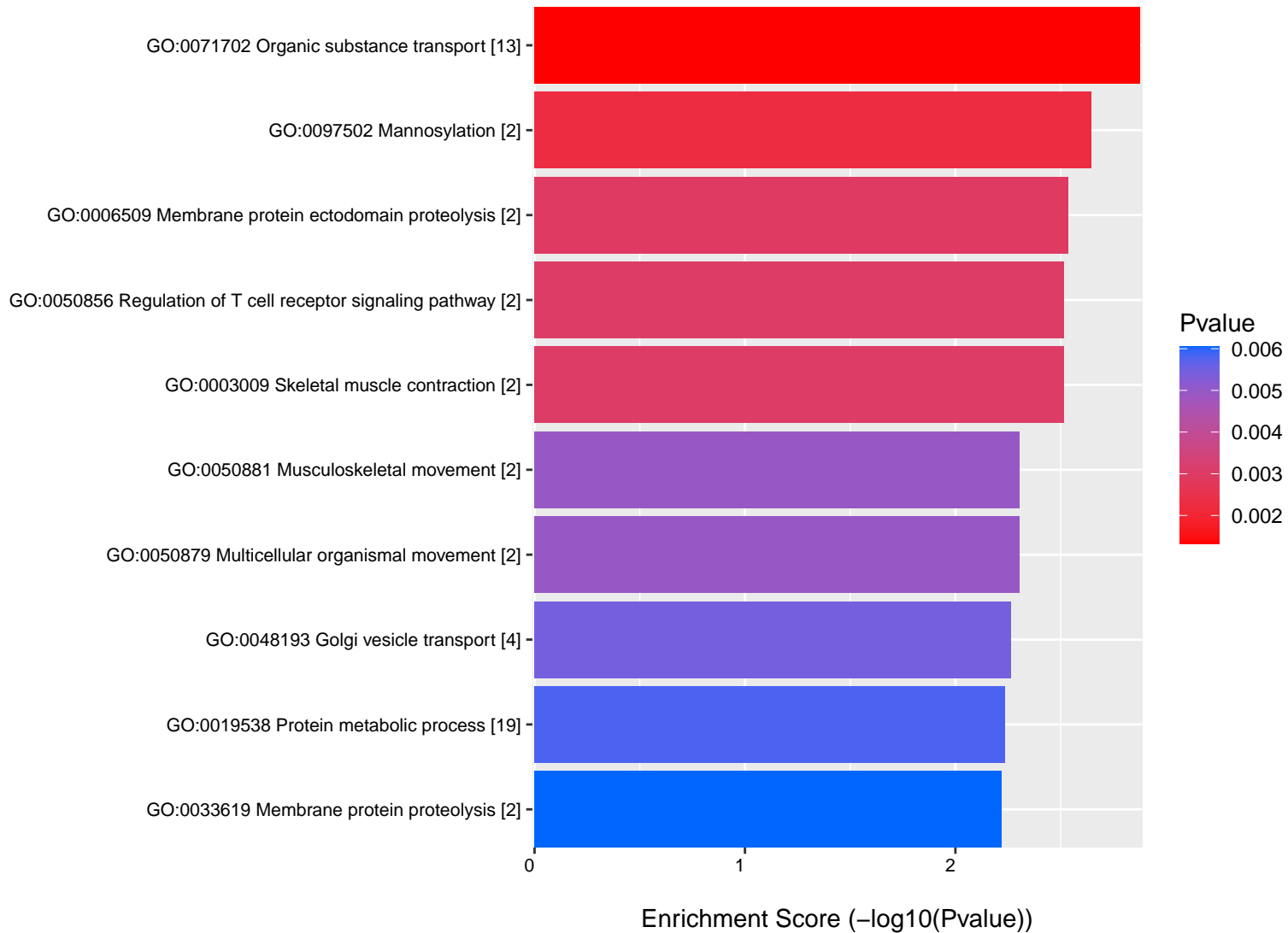

Supplement: Supplementary file 1 [file Data_Sheet_1.ZIP › Additional files/GO Analysis Report/GO_GC_vs_control_up/BP_EnrichmentScore.pdf]

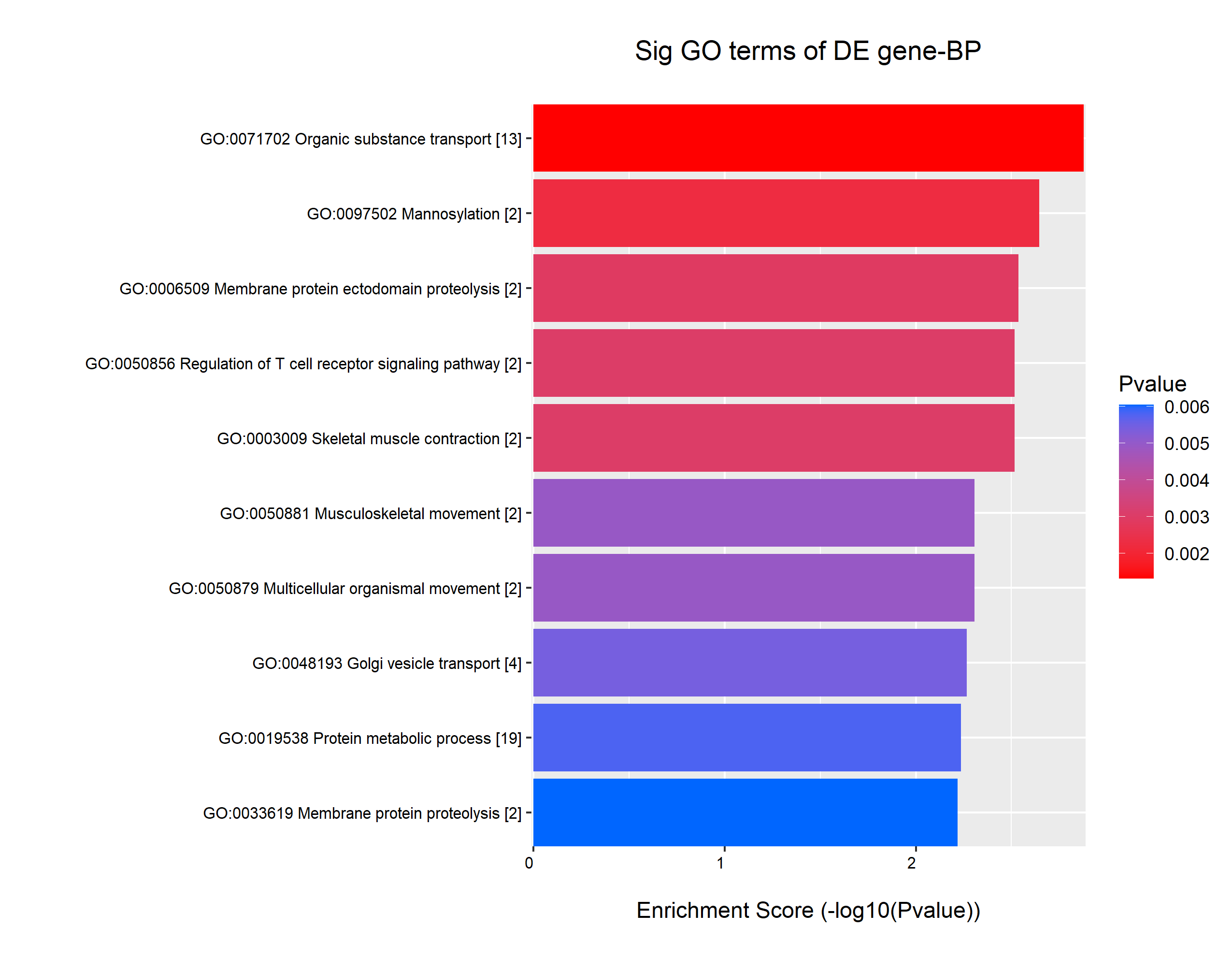

Supplement: Supplementary file 1 [file Data_Sheet_1.ZIP › Additional files/GO Analysis Report/GO_GC_vs_control_up/BP_EnrichmentScore.png]

## Sig GO terms of DE gene-BP

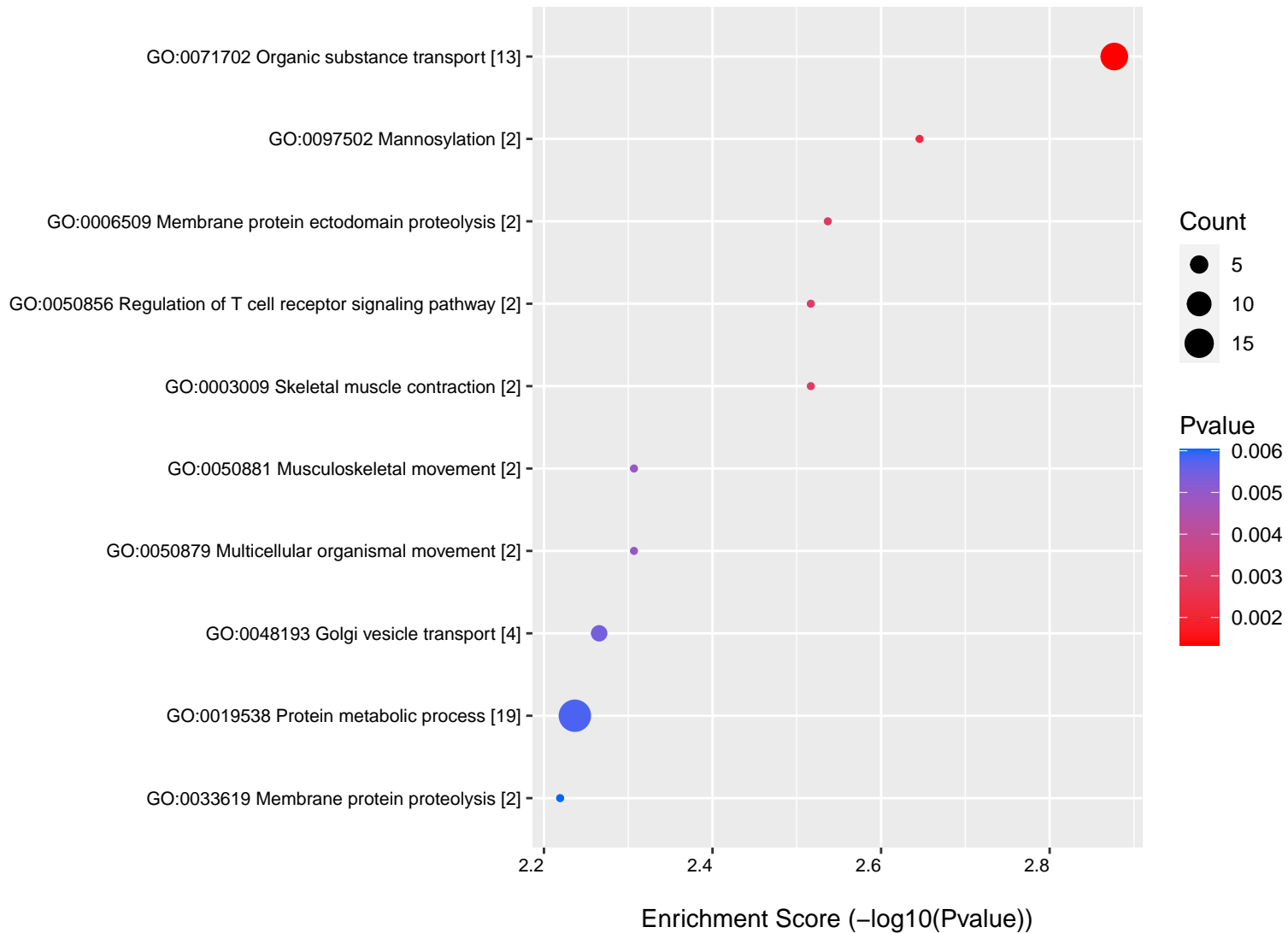

Supplement: Supplementary file 1 [file Data_Sheet_1.ZIP › Additional files/GO Analysis Report/GO_GC_vs_control_up/BP_EnrichmentScoreDotPlot.pdf]

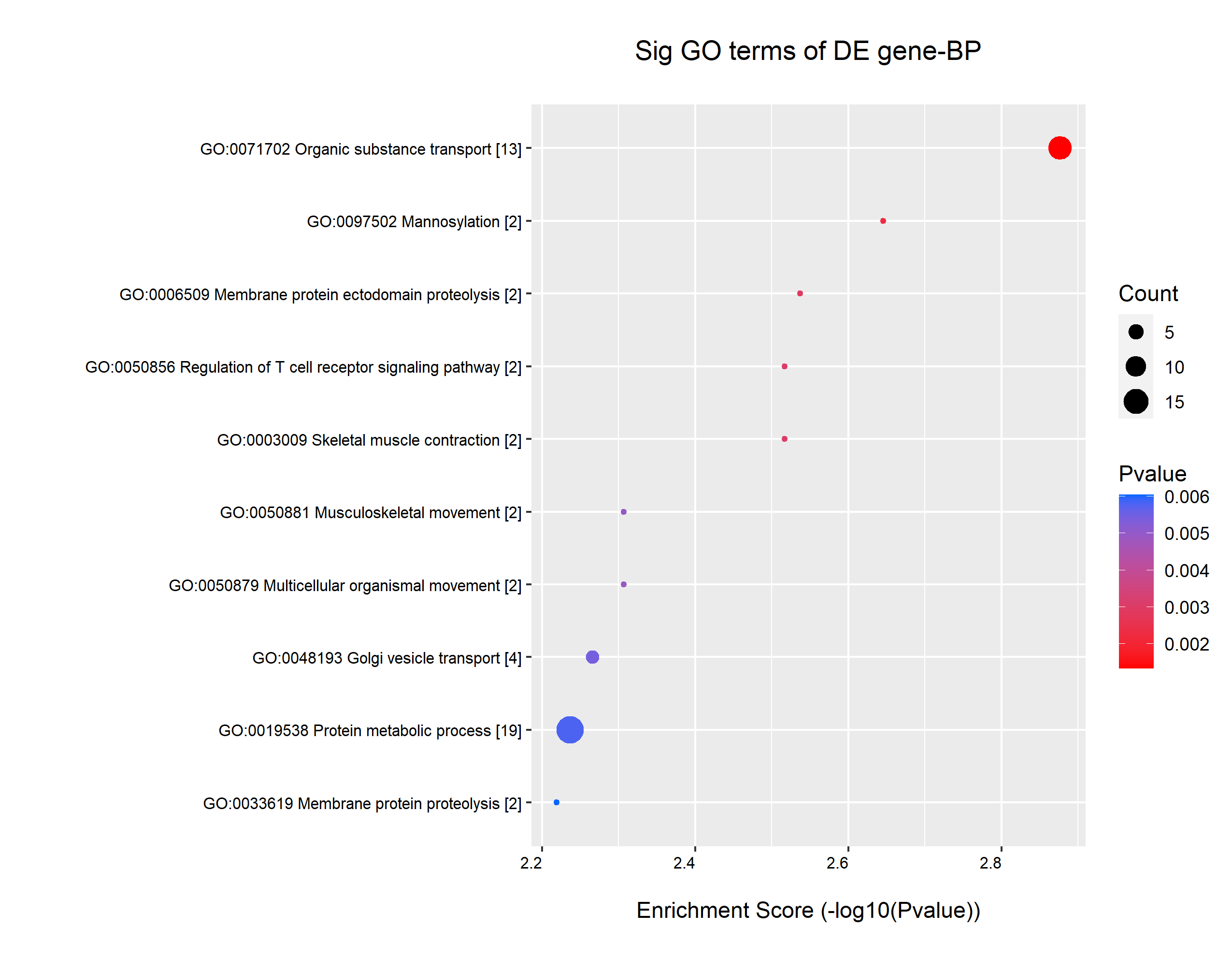

Supplement: Supplementary file 1 [file Data_Sheet_1.ZIP › Additional files/GO Analysis Report/GO_GC_vs_control_up/BP_EnrichmentScoreDotPlot.png]

## Sig GO terms of DE gene-BP

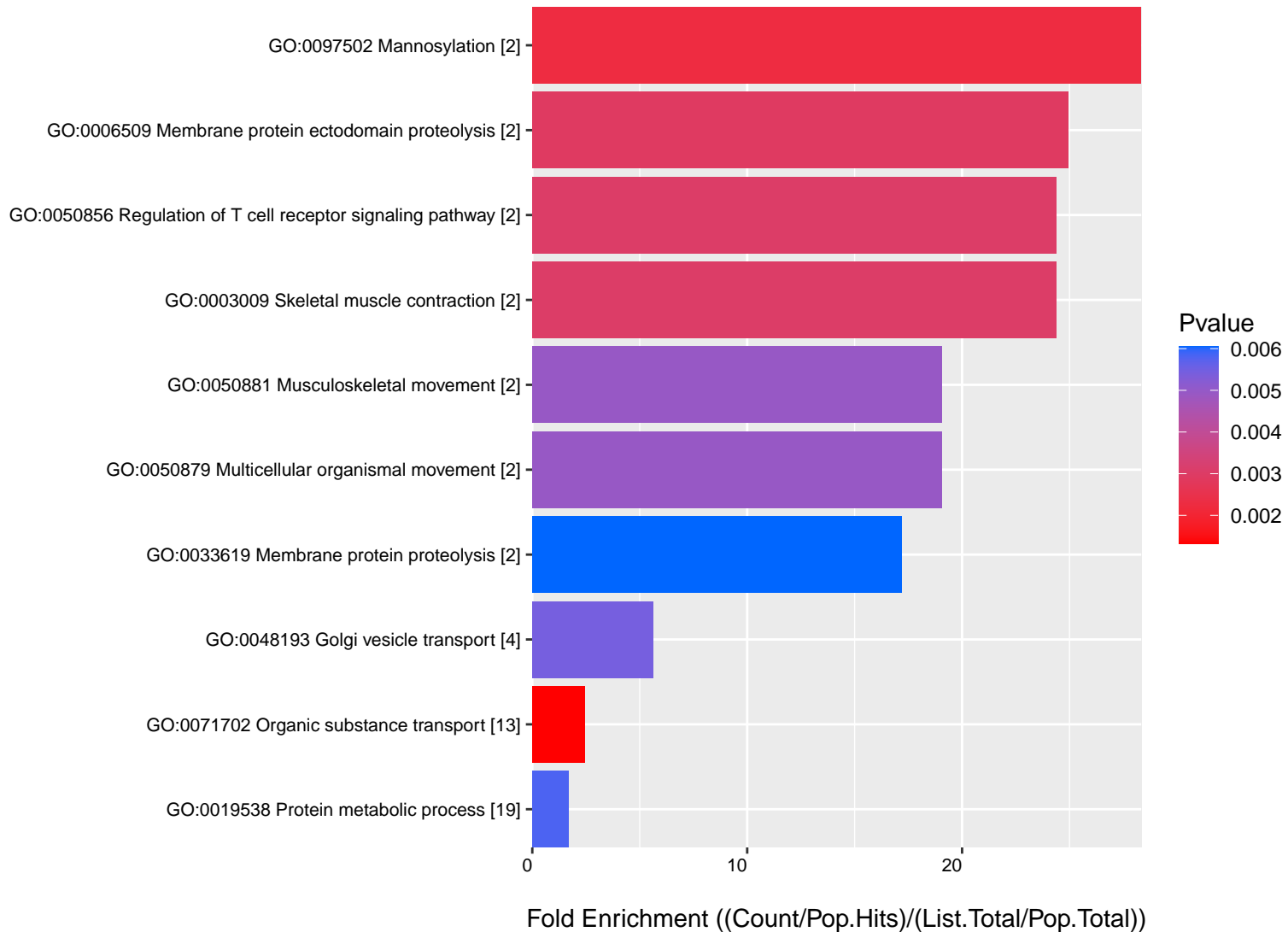

Supplement: Supplementary file 1 [file Data_Sheet_1.ZIP › Additional files/GO Analysis Report/GO_GC_vs_control_up/BP_FoldEnrichment.pdf]

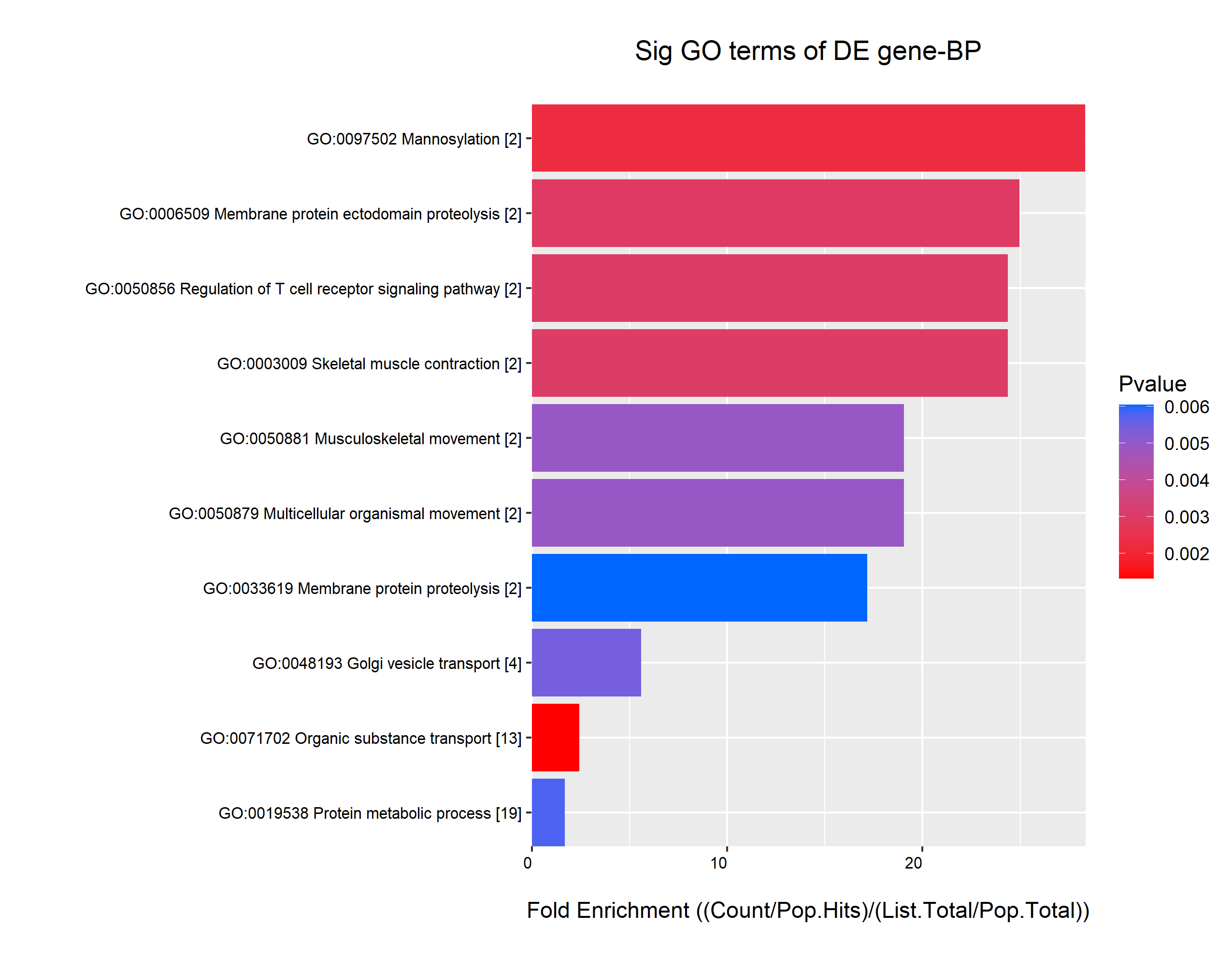

Supplement: Supplementary file 1 [file Data_Sheet_1.ZIP › Additional files/GO Analysis Report/GO_GC_vs_control_up/BP_FoldEnrichment.png]

# Sig GO terms of DE gene-BP

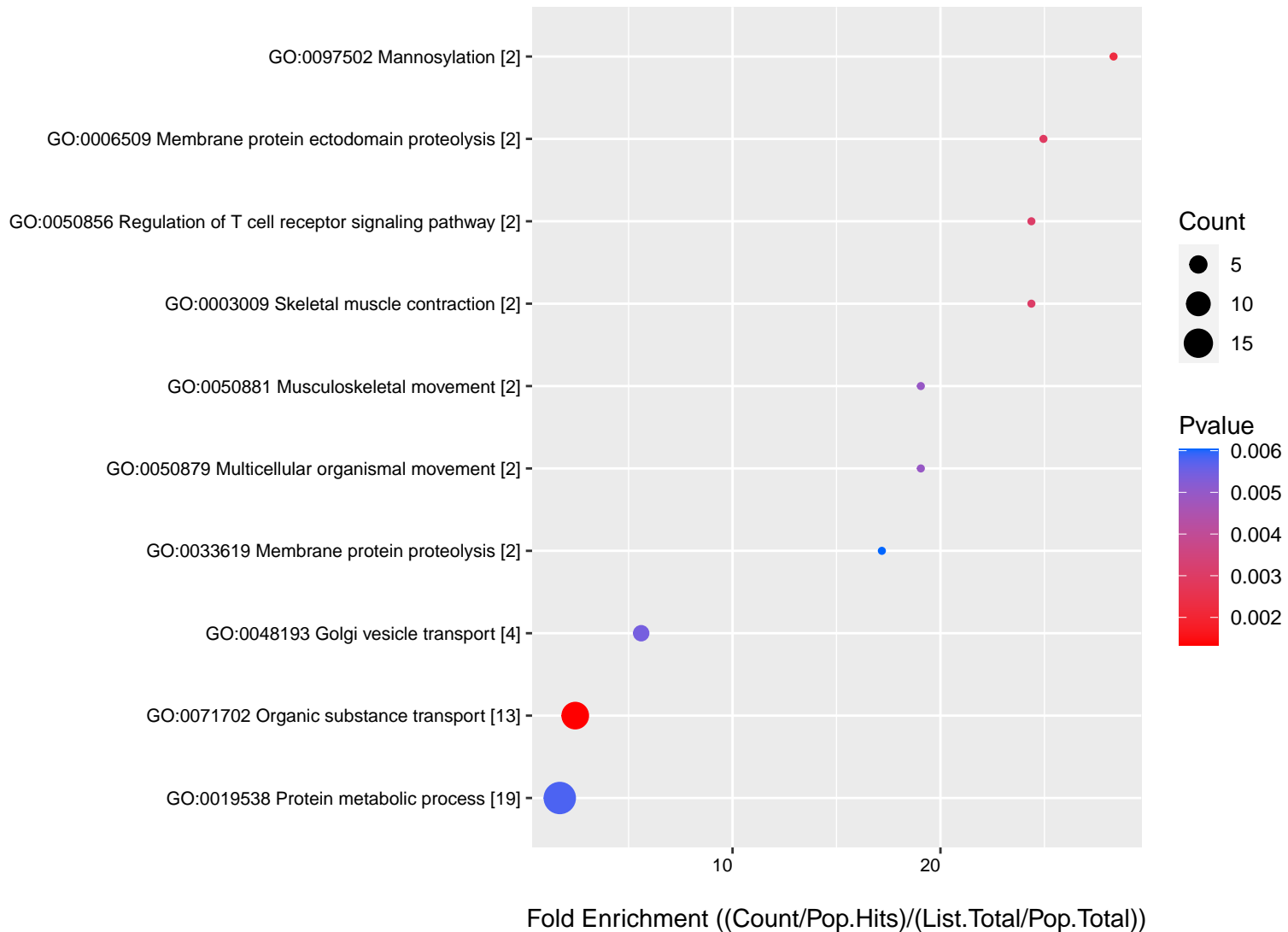

Supplement: Supplementary file 1 [file Data_Sheet_1.ZIP › Additional files/GO Analysis Report/GO_GC_vs_control_up/BP_FoldEnrichmentDotPlot.pdf]

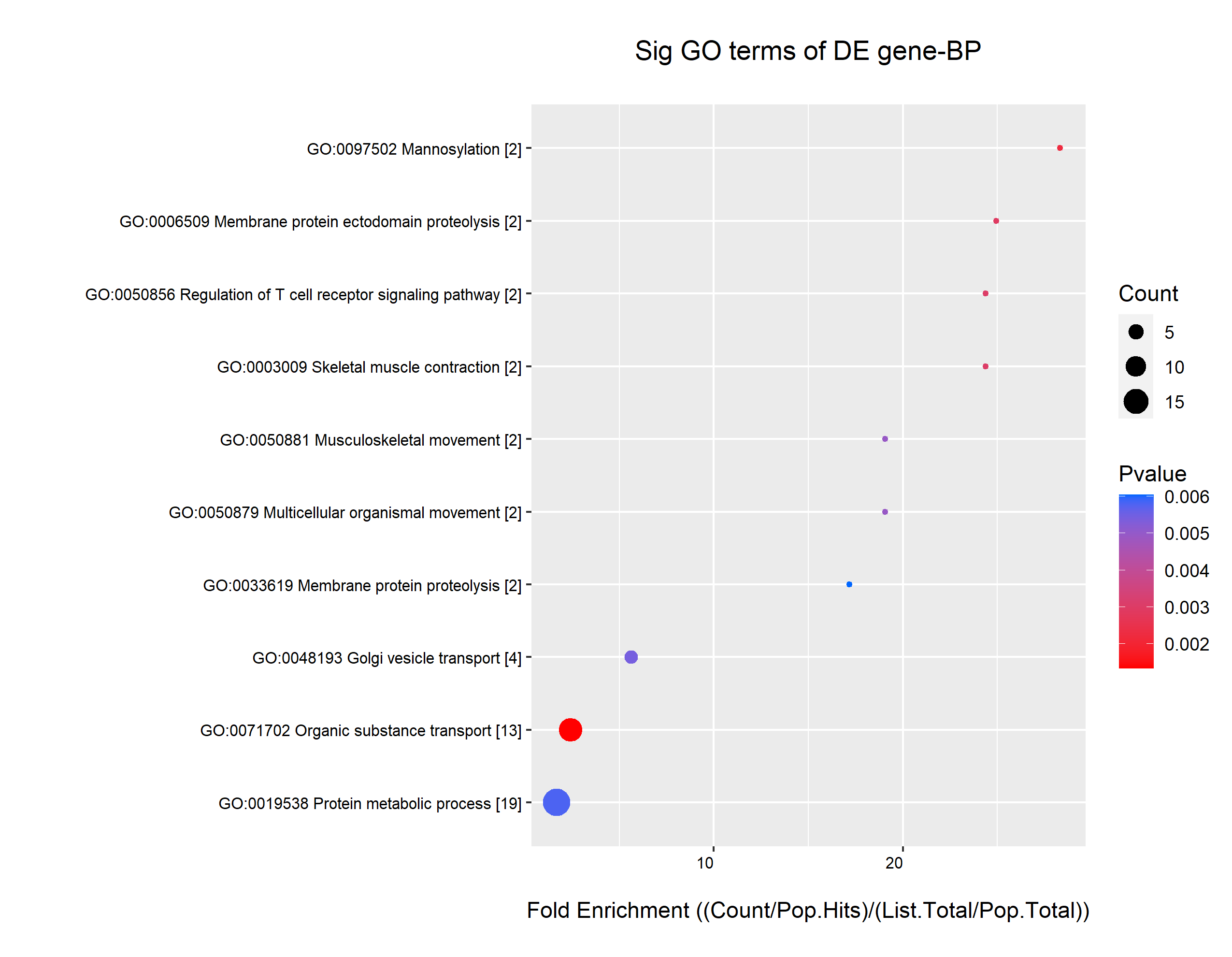

Supplement: Supplementary file 1 [file Data_Sheet_1.ZIP › Additional files/GO Analysis Report/GO_GC_vs_control_up/BP_FoldEnrichmentDotPlot.png]

# Sig GO terms of DE gene-BP

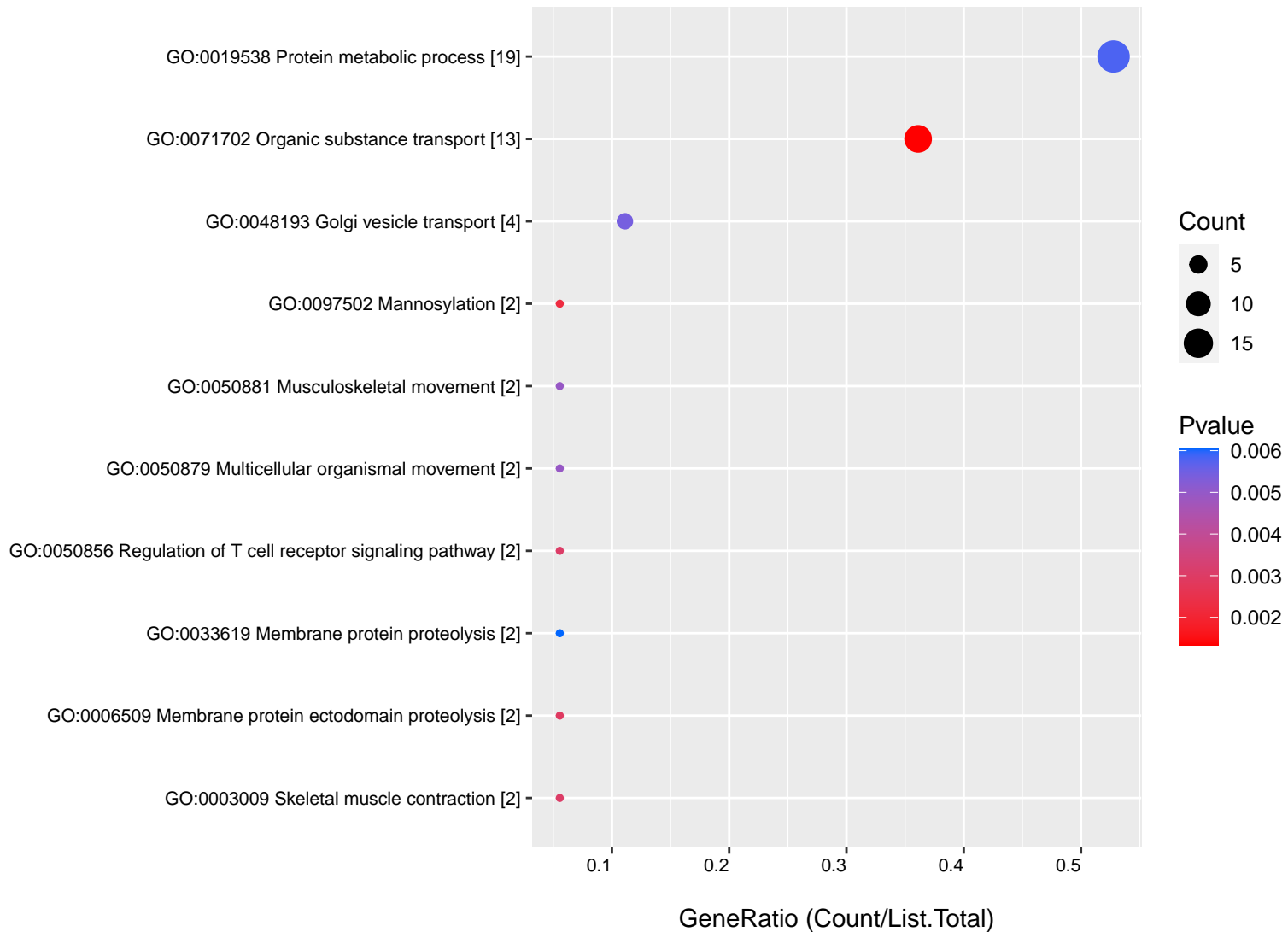

Supplement: Supplementary file 1 [file Data_Sheet_1.ZIP › Additional files/GO Analysis Report/GO_GC_vs_control_up/BP_GeneRatioDotPlot.pdf]

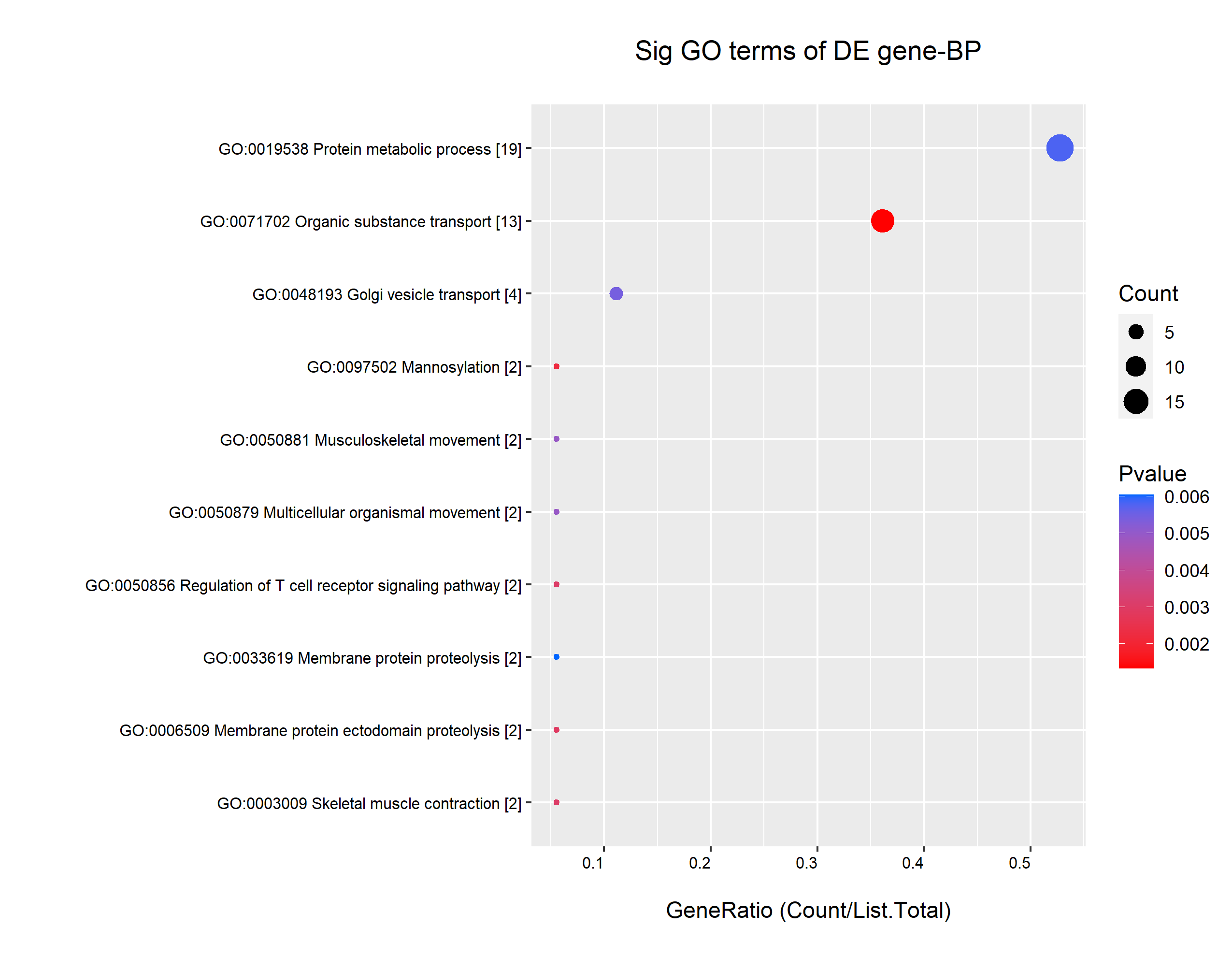

Supplement: Supplementary file 1 [file Data_Sheet_1.ZIP › Additional files/GO Analysis Report/GO_GC_vs_control_up/BP_GeneRatioDotPlot.png]

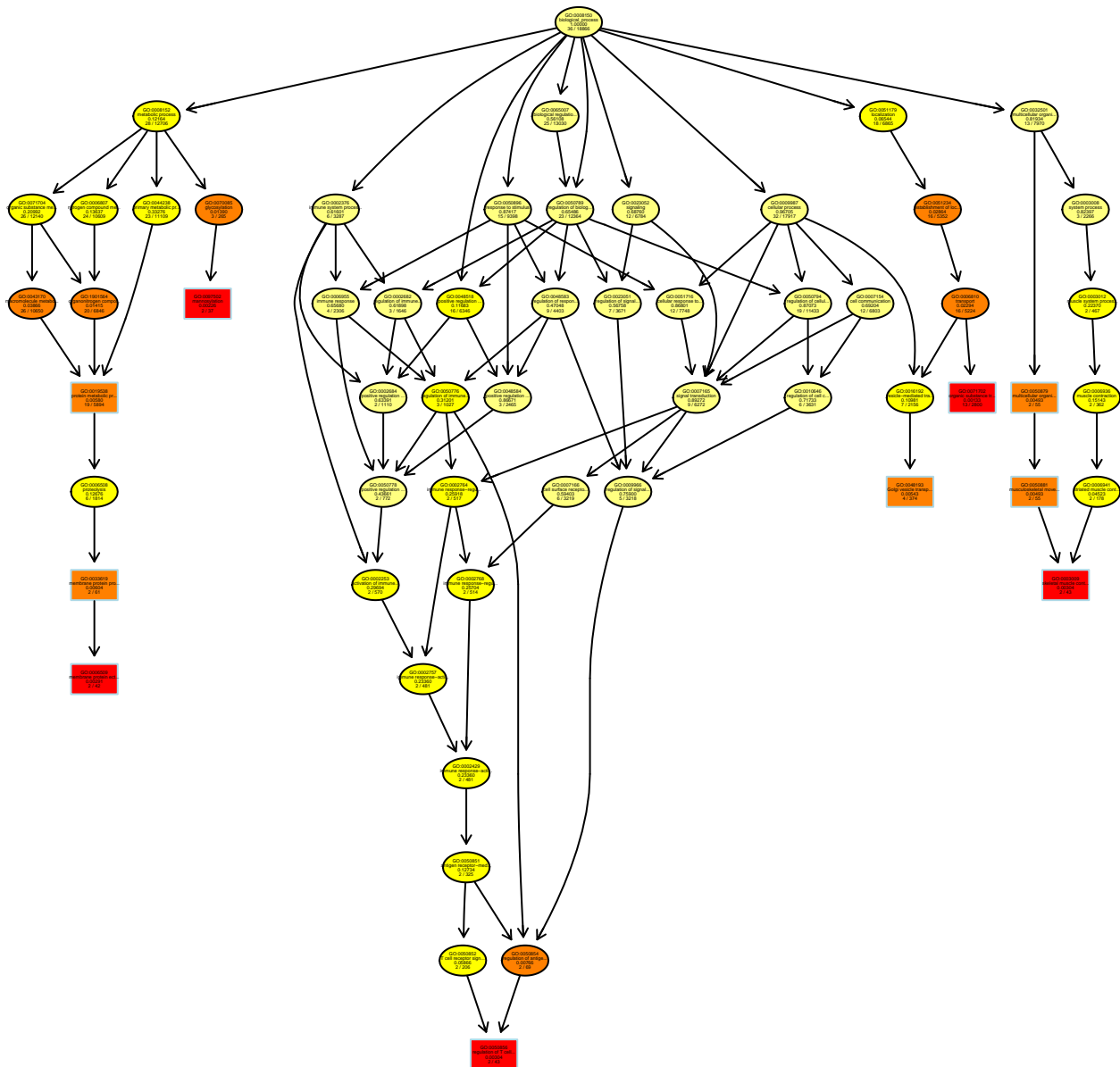

Supplement: Supplementary file 1 [file Data_Sheet_1.ZIP › Additional files/GO Analysis Report/GO_GC_vs_control_up/BP_Pvalue_tree.pdf]

# GO Cellular Component Classification

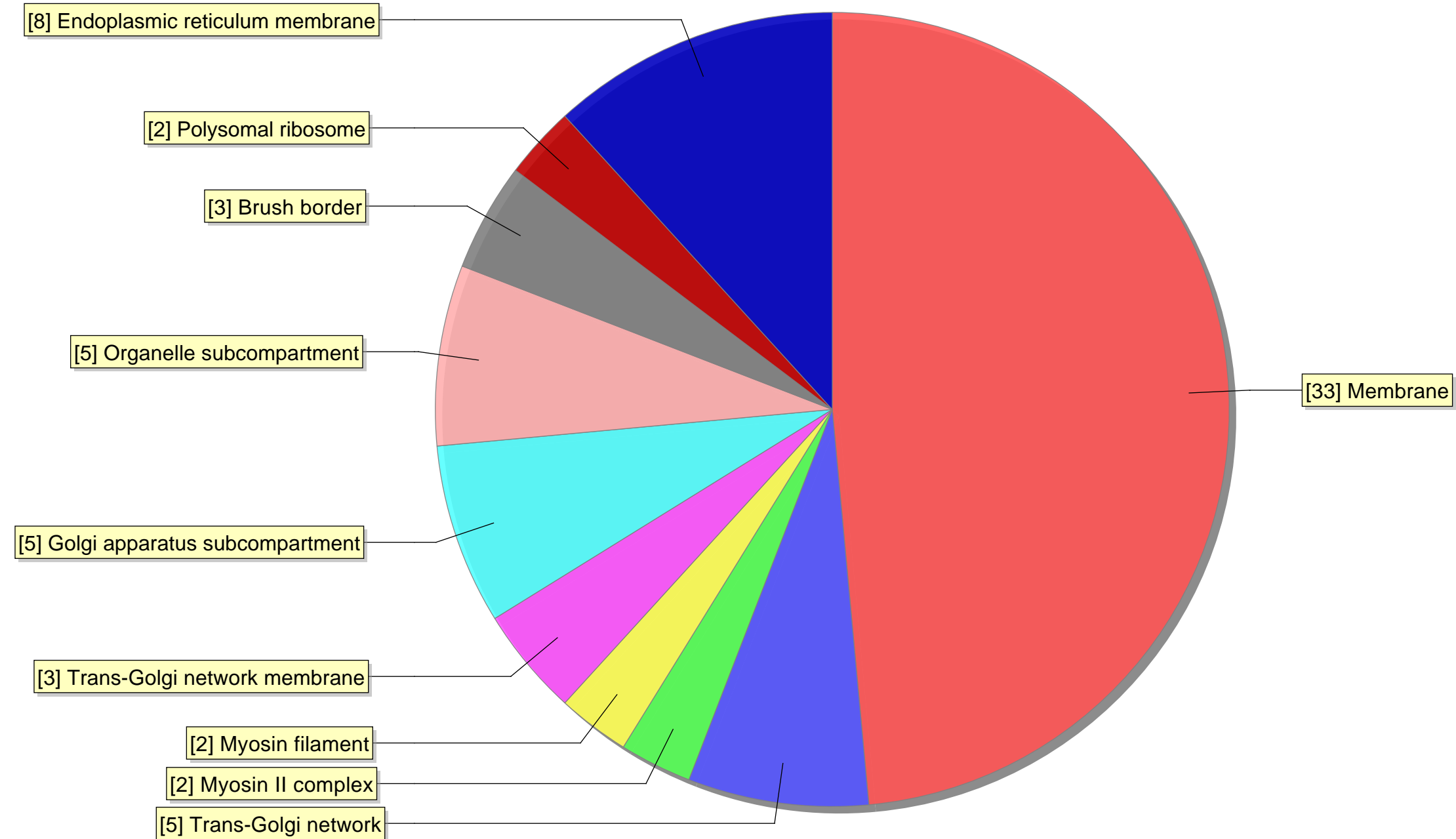

Supplement: Supplementary file 1 [file Data_Sheet_1.ZIP › Additional files/GO Analysis Report/GO_GC_vs_control_up/CC_Count.pdf]

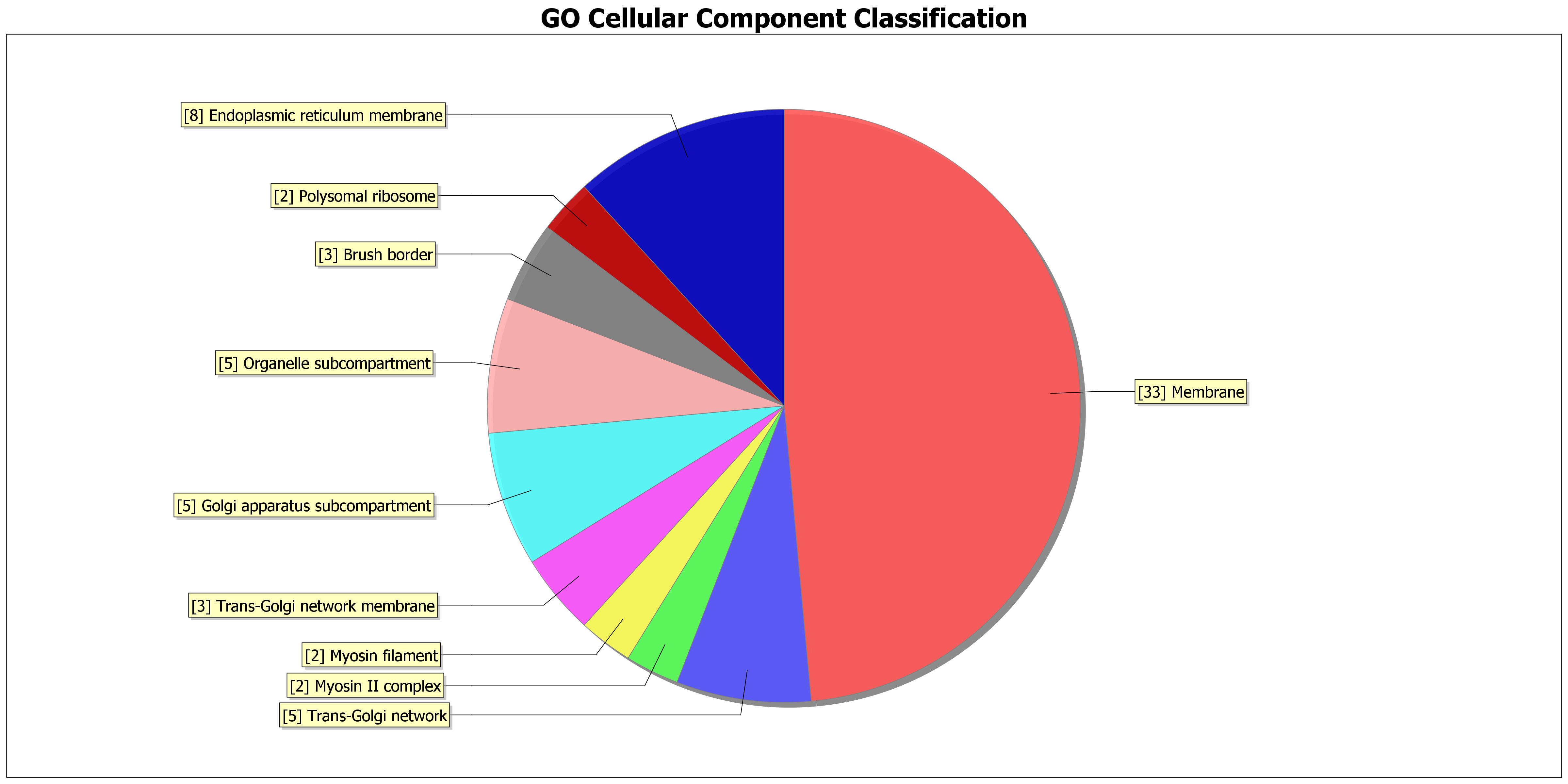

Supplement: Supplementary file 1 [file Data_Sheet_1.ZIP › Additional files/GO Analysis Report/GO_GC_vs_control_up/CC_Count.png]

## Sig GO terms of DE gene-CC

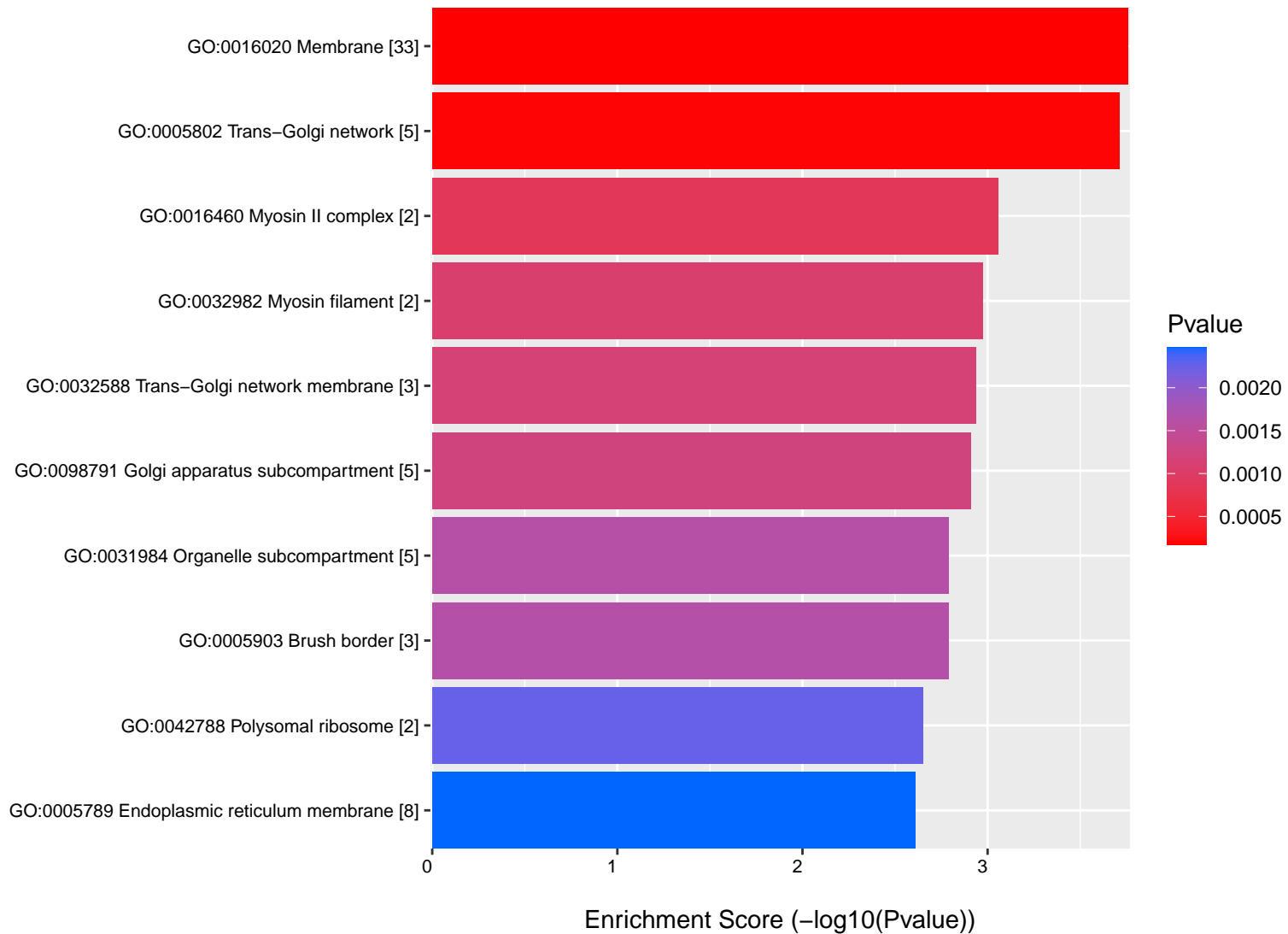

Supplement: Supplementary file 1 [file Data_Sheet_1.ZIP › Additional files/GO Analysis Report/GO_GC_vs_control_up/CC_EnrichmentScore.pdf]

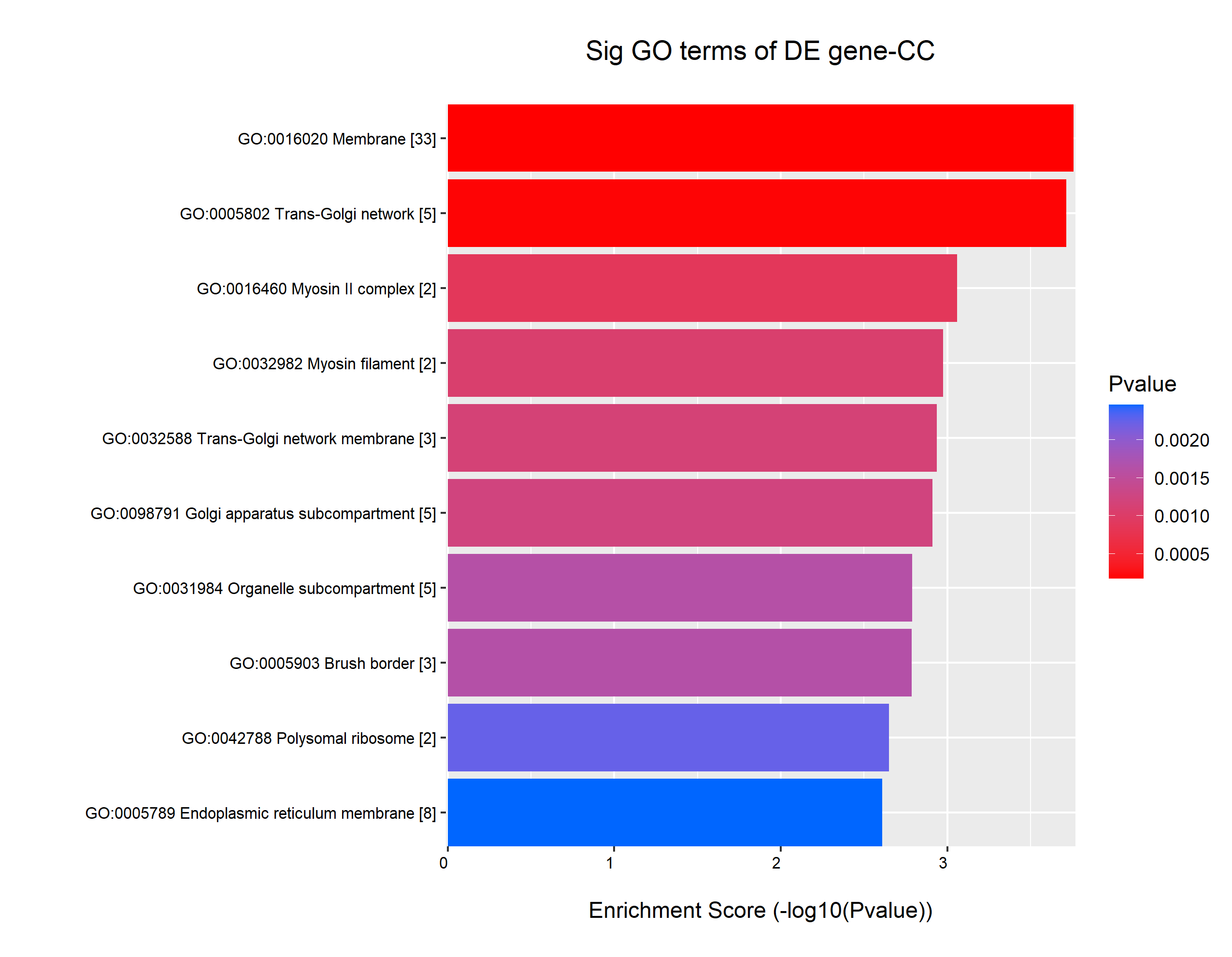

Supplement: Supplementary file 1 [file Data_Sheet_1.ZIP › Additional files/GO Analysis Report/GO_GC_vs_control_up/CC_EnrichmentScore.png]

## Sig GO terms of DE gene-CC

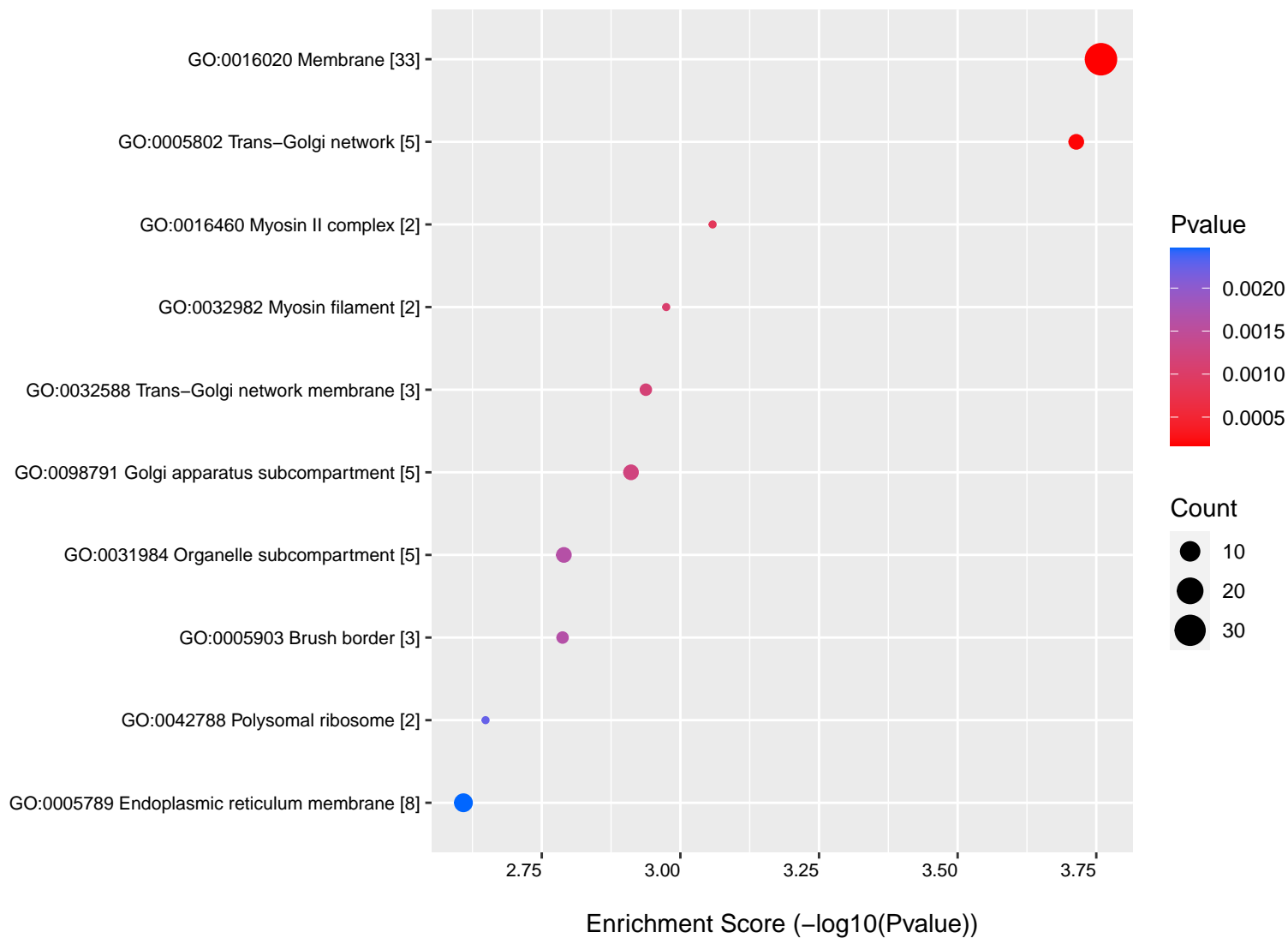

Supplement: Supplementary file 1 [file Data_Sheet_1.ZIP › Additional files/GO Analysis Report/GO_GC_vs_control_up/CC_EnrichmentScoreDotPlot.pdf]

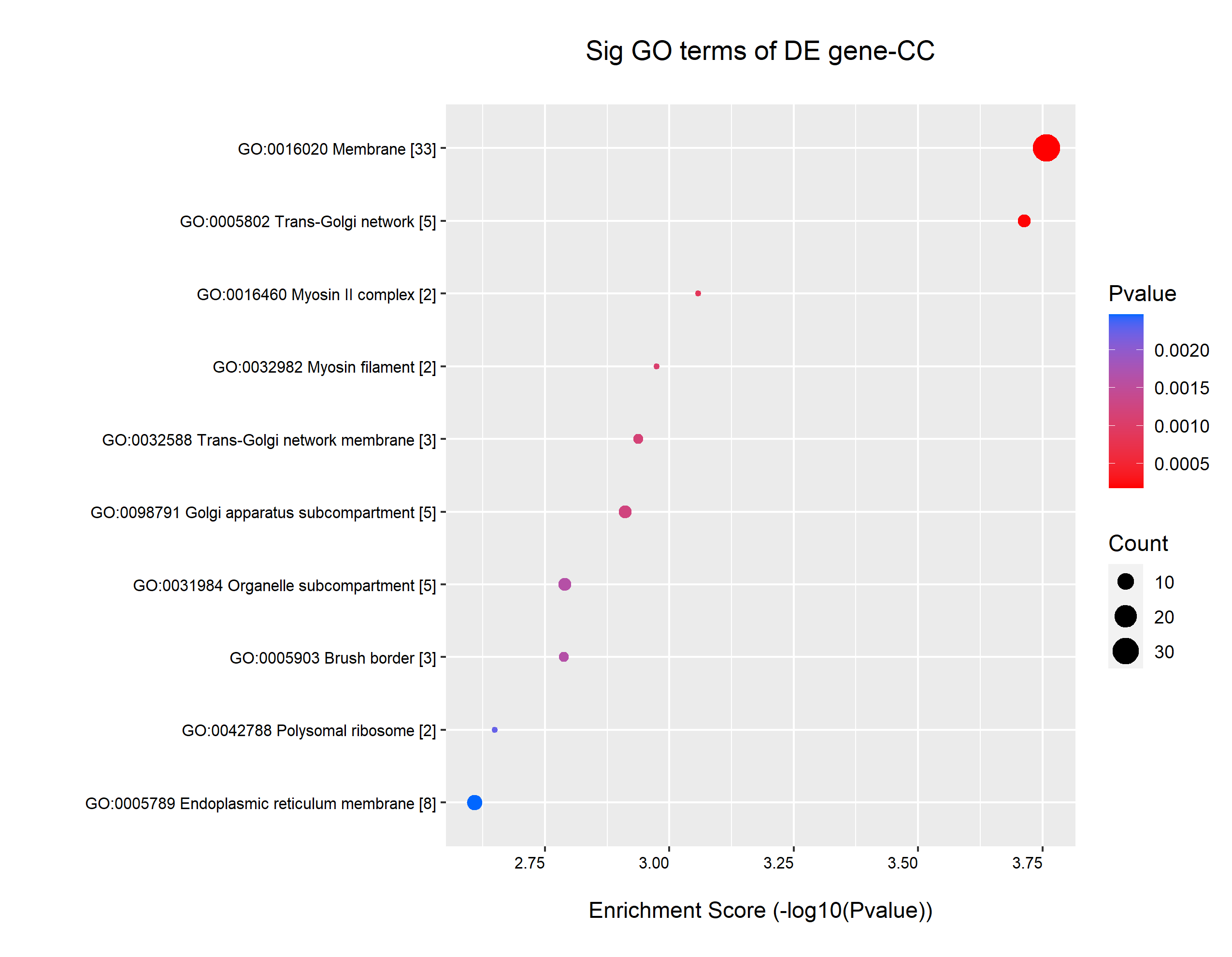

Supplement: Supplementary file 1 [file Data_Sheet_1.ZIP › Additional files/GO Analysis Report/GO_GC_vs_control_up/CC_EnrichmentScoreDotPlot.png]

## Sig GO terms of DE gene-CC

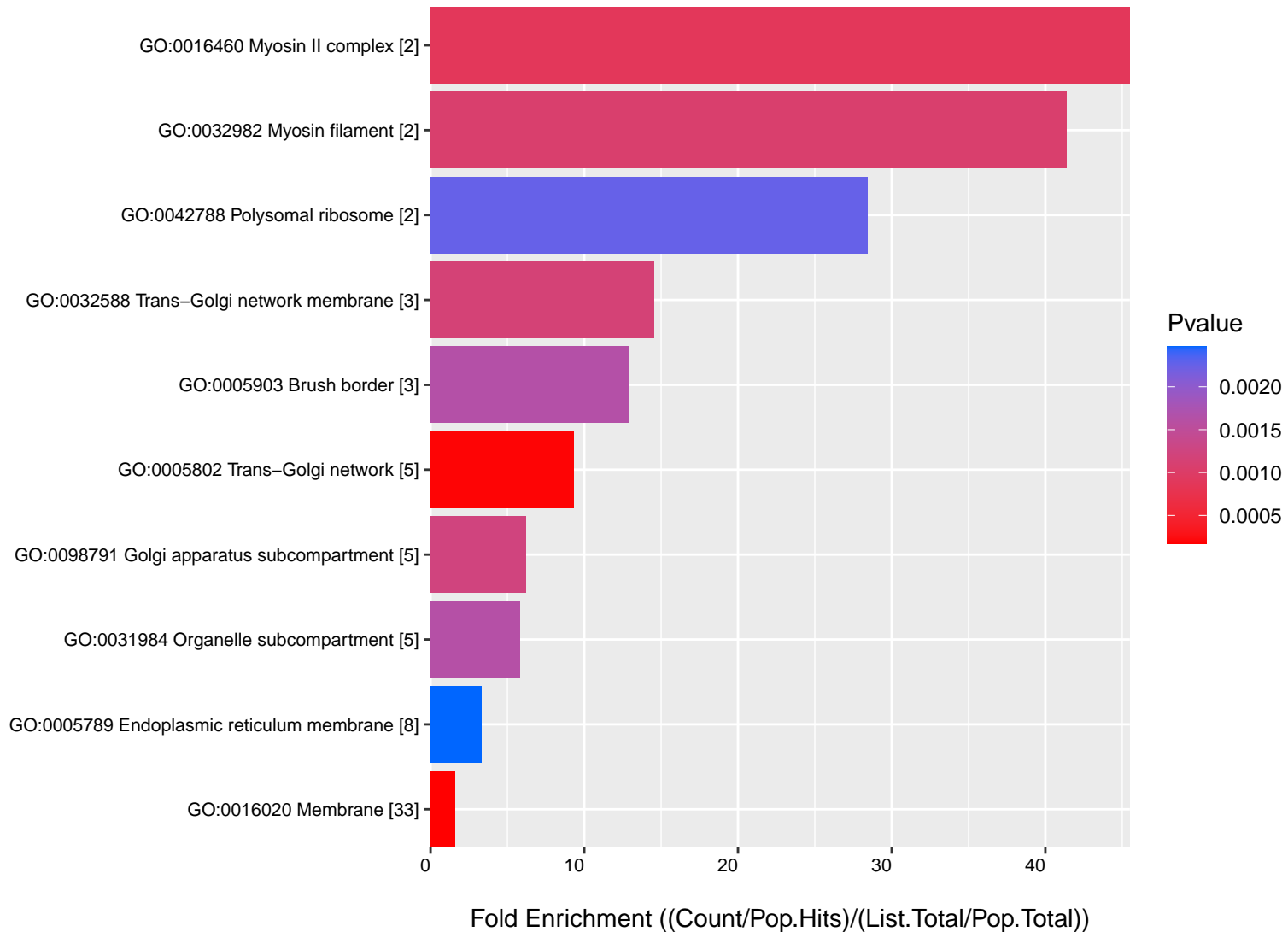

Supplement: Supplementary file 1 [file Data_Sheet_1.ZIP › Additional files/GO Analysis Report/GO_GC_vs_control_up/CC_FoldEnrichment.pdf]

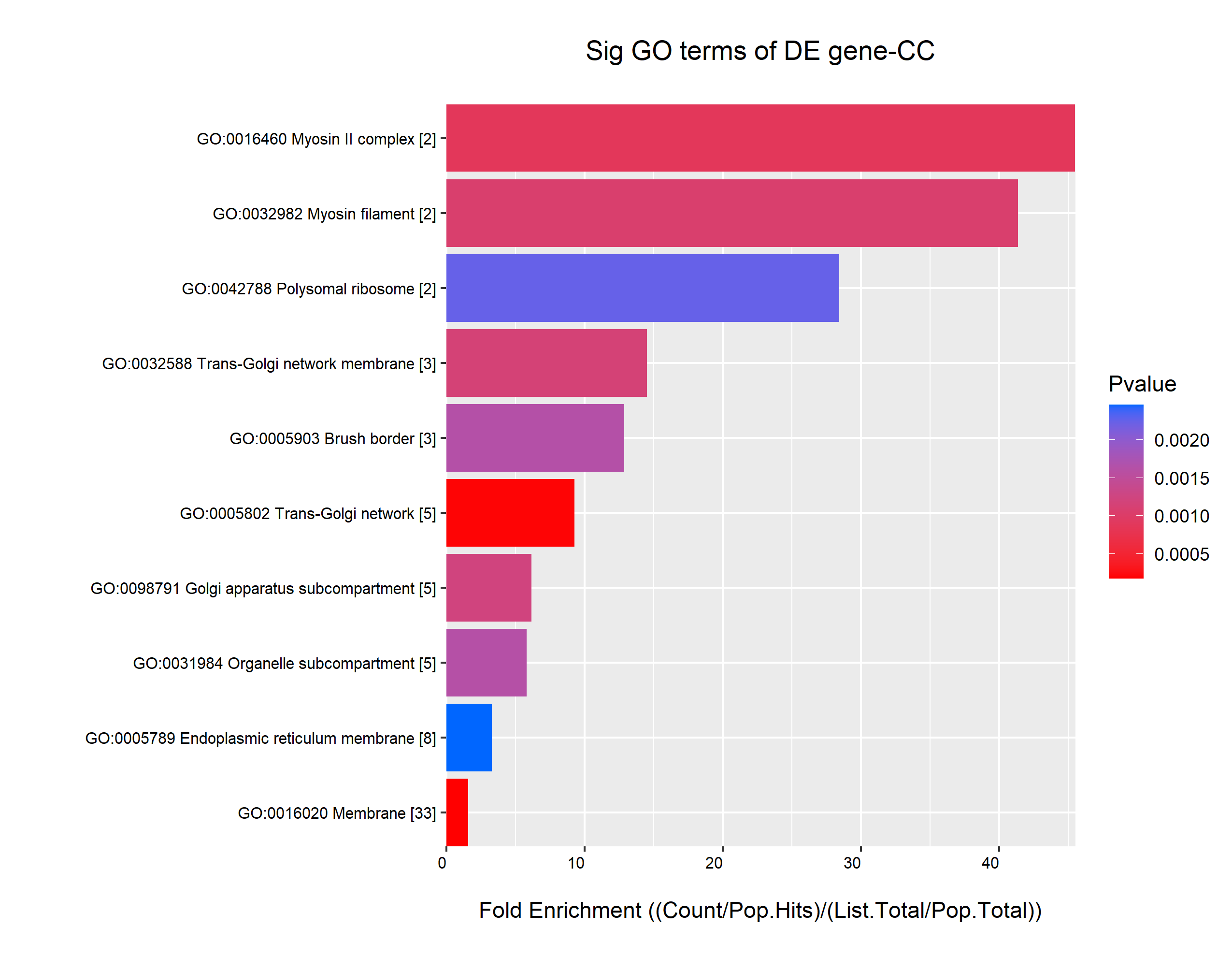

Supplement: Supplementary file 1 [file Data_Sheet_1.ZIP › Additional files/GO Analysis Report/GO_GC_vs_control_up/CC_FoldEnrichment.png]

## Sig GO terms of DE gene-CC

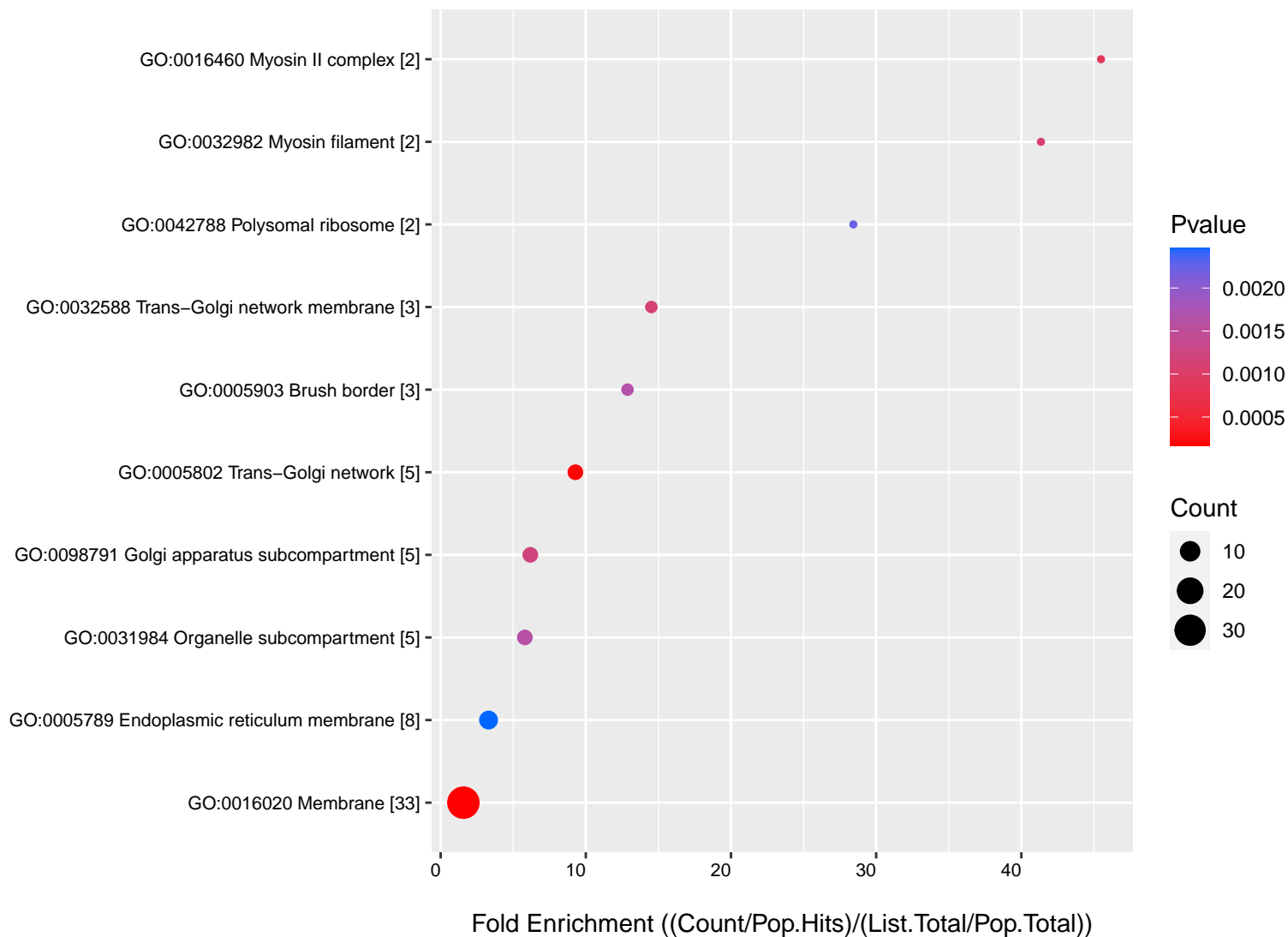

Supplement: Supplementary file 1 [file Data_Sheet_1.ZIP › Additional files/GO Analysis Report/GO_GC_vs_control_up/CC_FoldEnrichmentDotPlot.pdf]

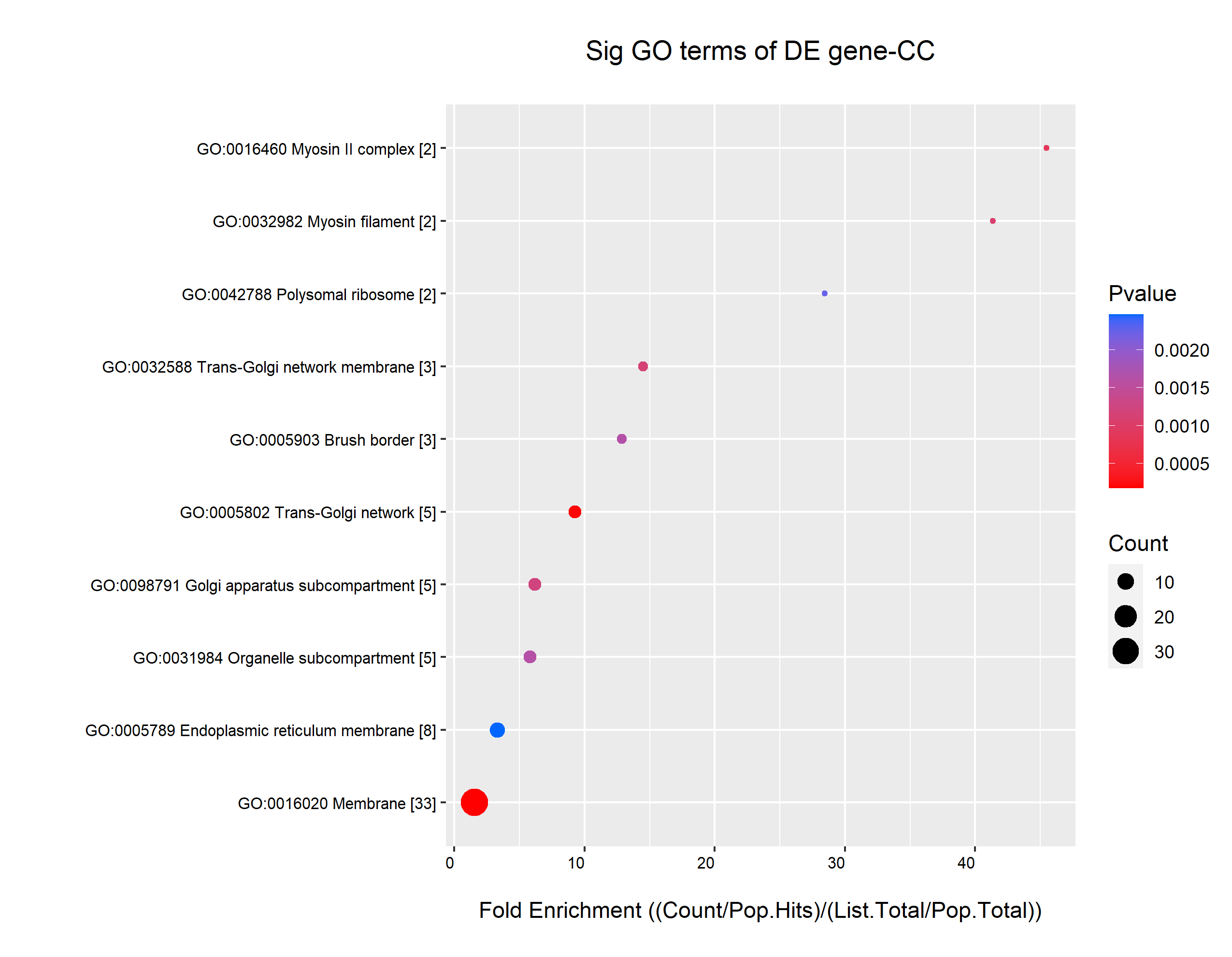

Supplement: Supplementary file 1 [file Data_Sheet_1.ZIP › Additional files/GO Analysis Report/GO_GC_vs_control_up/CC_FoldEnrichmentDotPlot.png]

## Sig GO terms of DE gene-CC

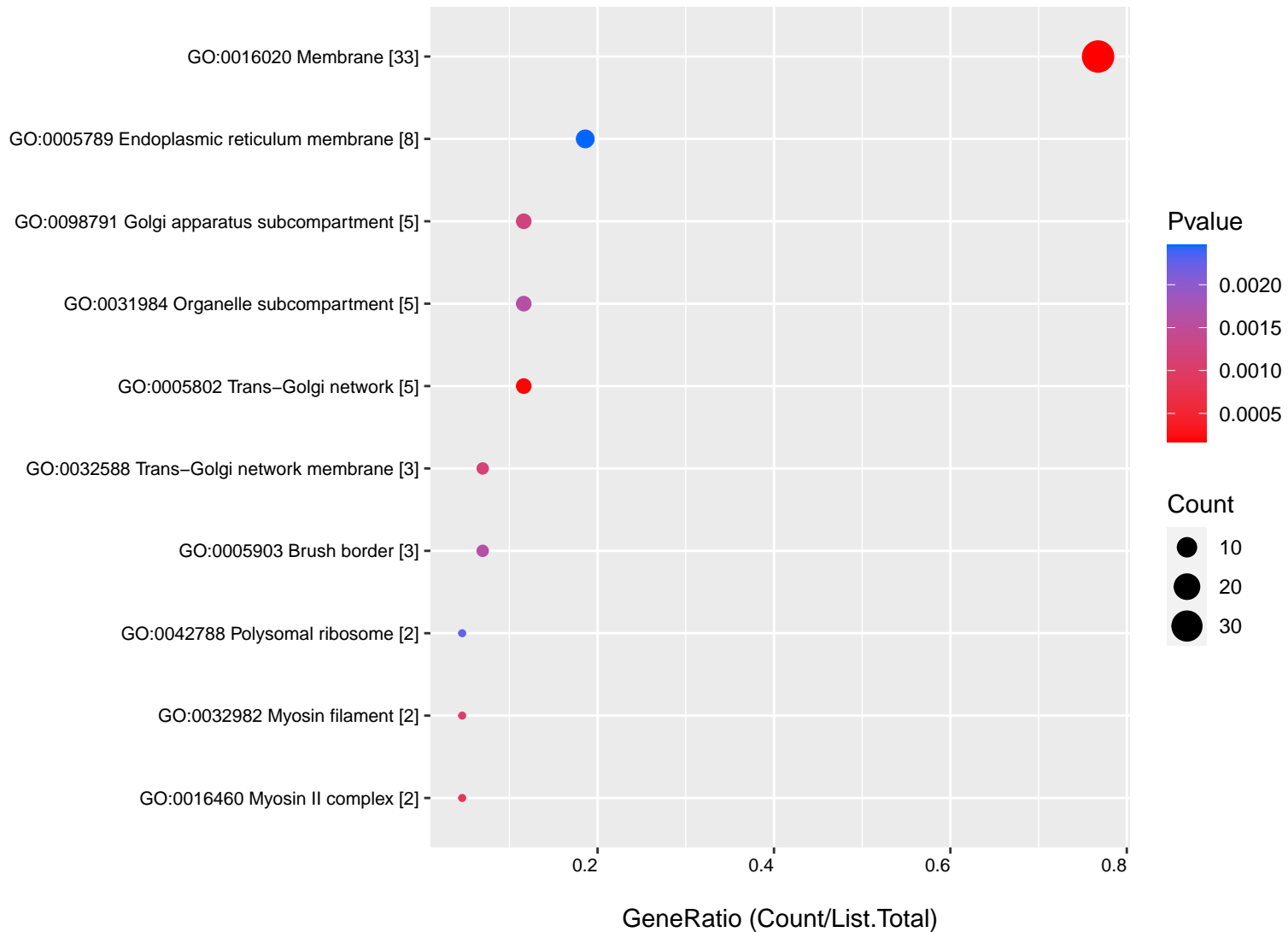

Supplement: Supplementary file 1 [file Data_Sheet_1.ZIP › Additional files/GO Analysis Report/GO_GC_vs_control_up/CC_GeneRatioDotPlot.pdf]

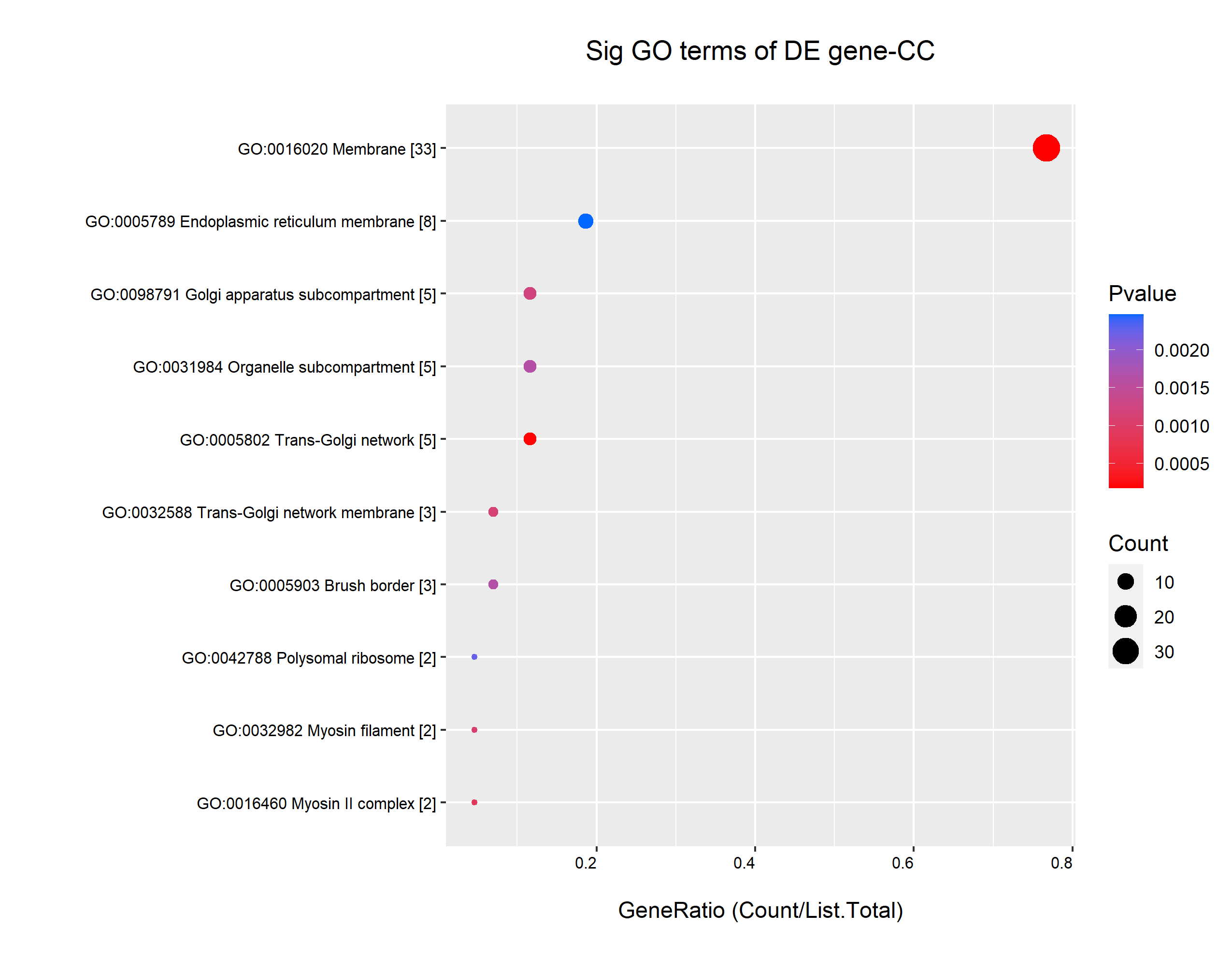

Supplement: Supplementary file 1 [file Data_Sheet_1.ZIP › Additional files/GO Analysis Report/GO_GC_vs_control_up/CC_GeneRatioDotPlot.png]

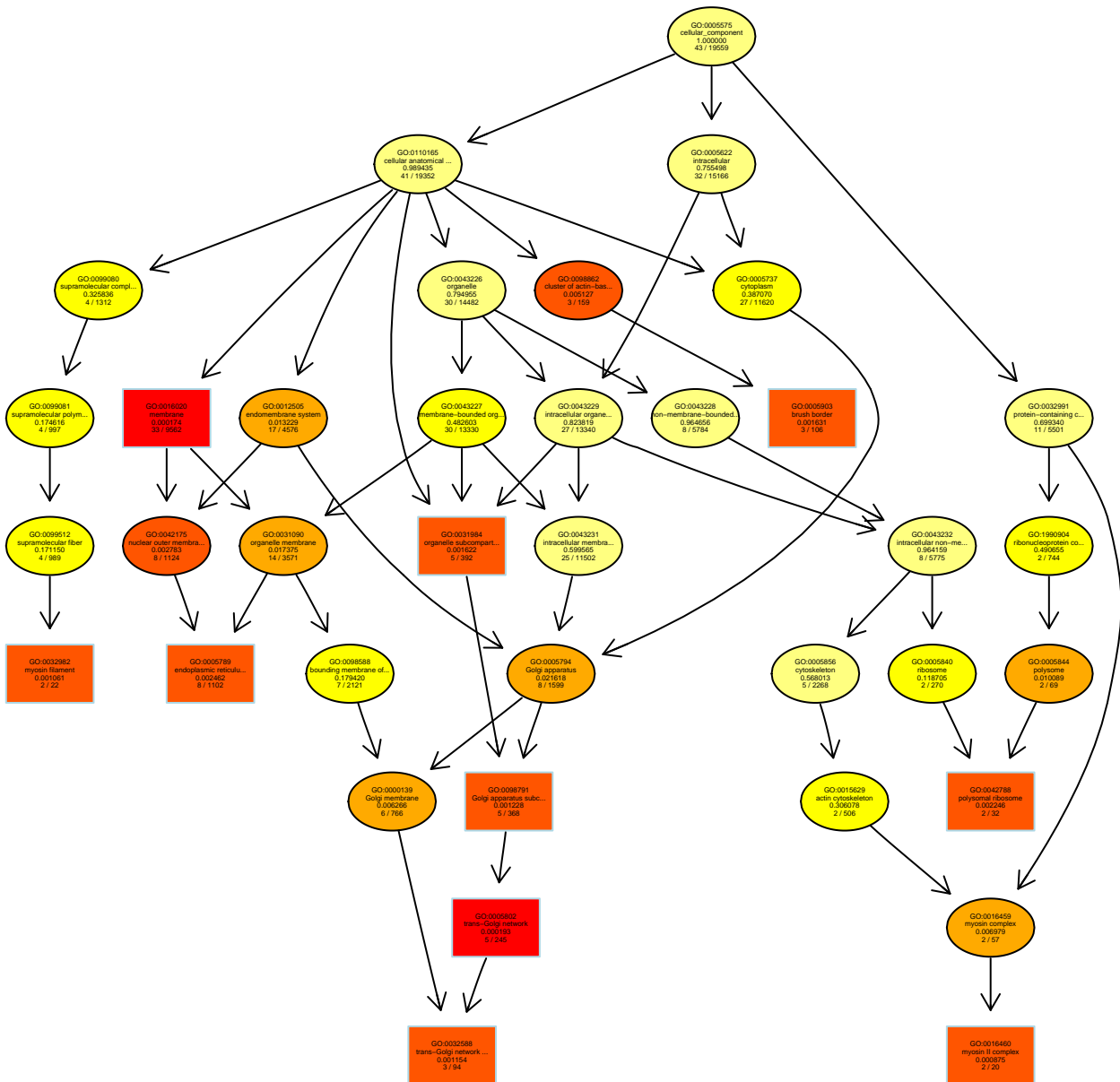

Supplement: Supplementary file 1 [file Data_Sheet_1.ZIP › Additional files/GO Analysis Report/GO_GC_vs_control_up/CC_Pvalue_tree.pdf]

Sig GO terms of DE gene

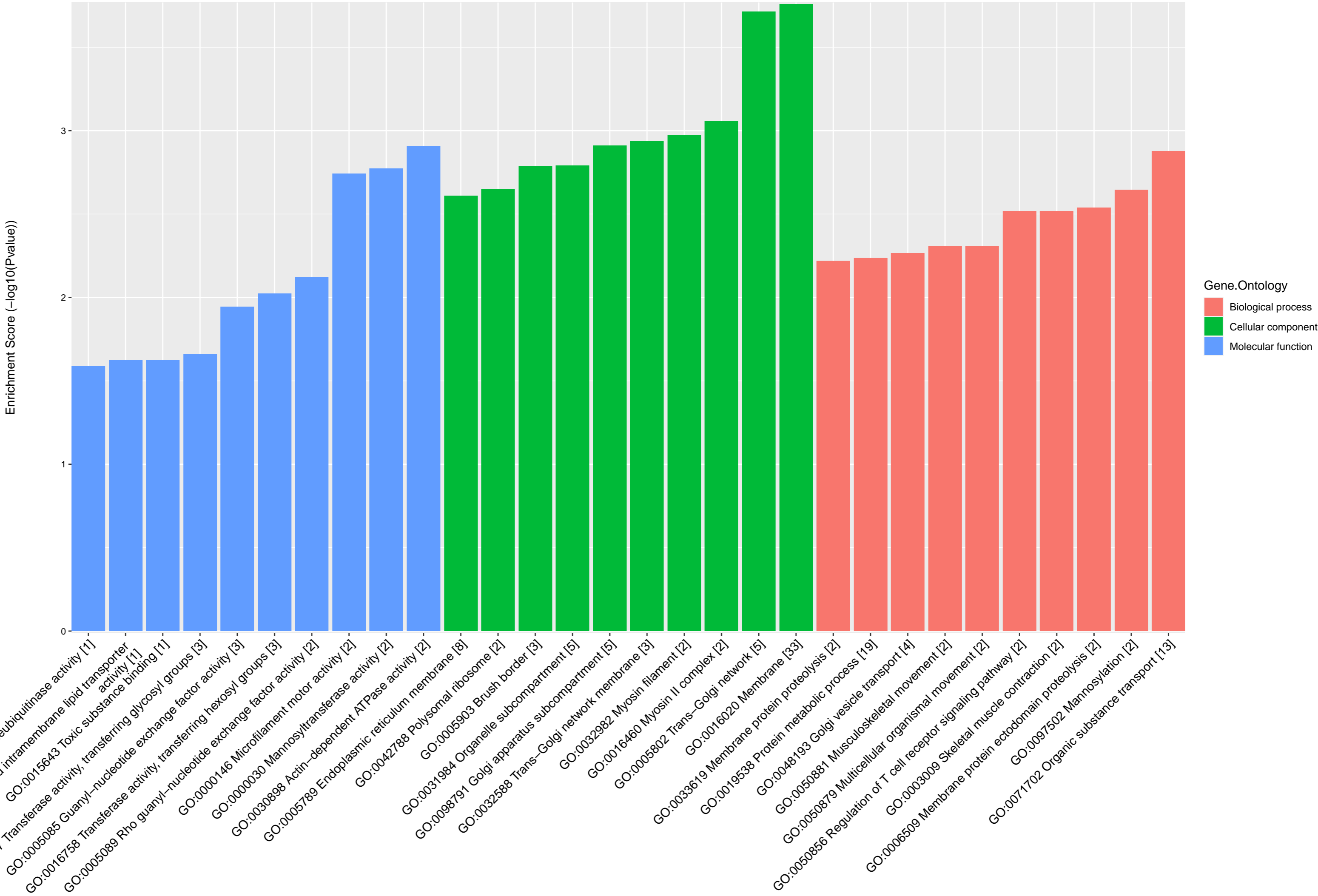

Supplement: Supplementary file 1 [file Data_Sheet_1.ZIP › Additional files/GO Analysis Report/GO_GC_vs_control_up/GeneOntology_EnrichmentScore.pdf]

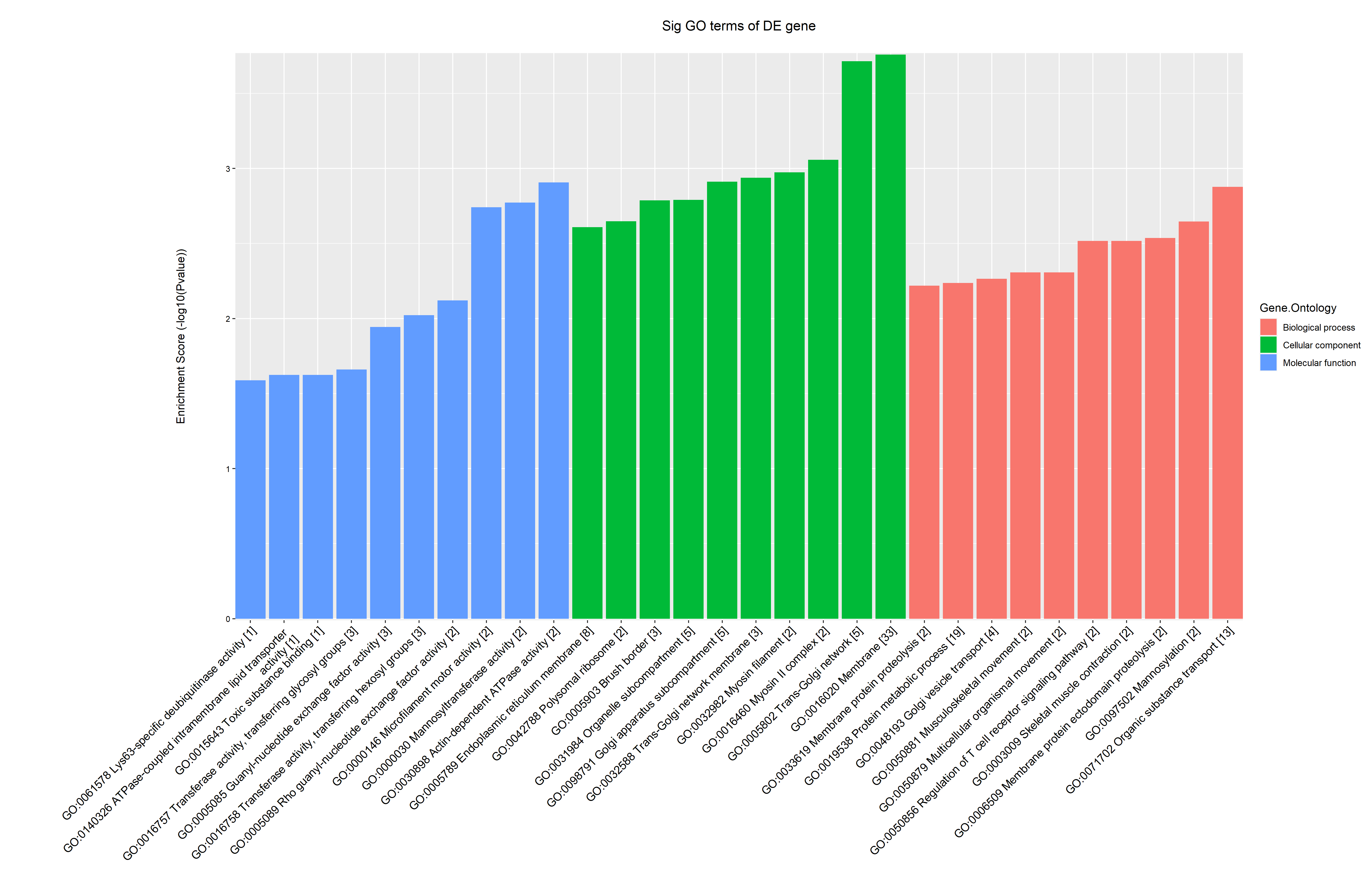

Supplement: Supplementary file 1 [file Data_Sheet_1.ZIP › Additional files/GO Analysis Report/GO_GC_vs_control_up/GeneOntology_EnrichmentScore.png]

Sig GO terms of DE gene

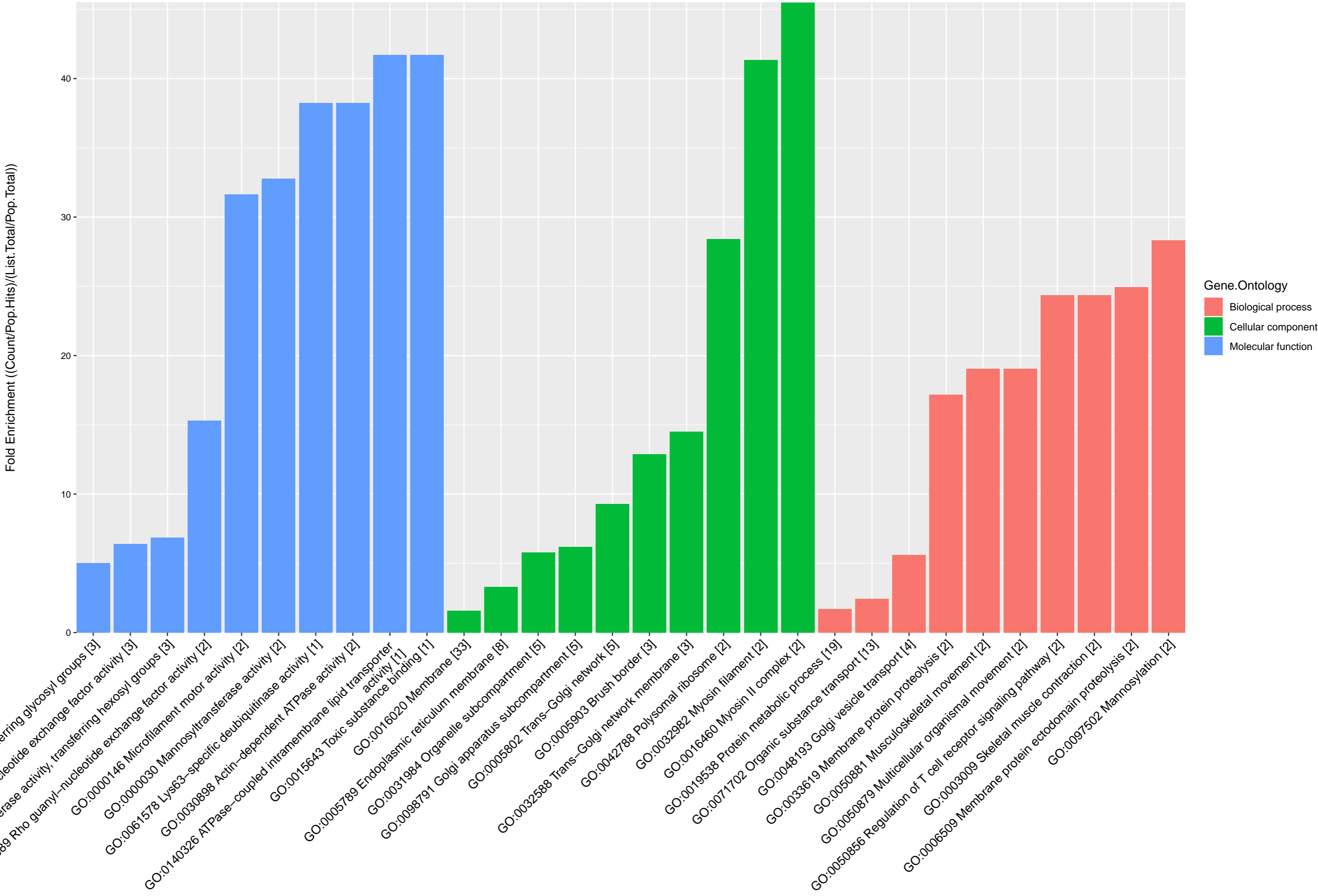

Supplement: Supplementary file 1 [file Data_Sheet_1.ZIP › Additional files/GO Analysis Report/GO_GC_vs_control_up/GeneOntology_FoldEnrichment.pdf]

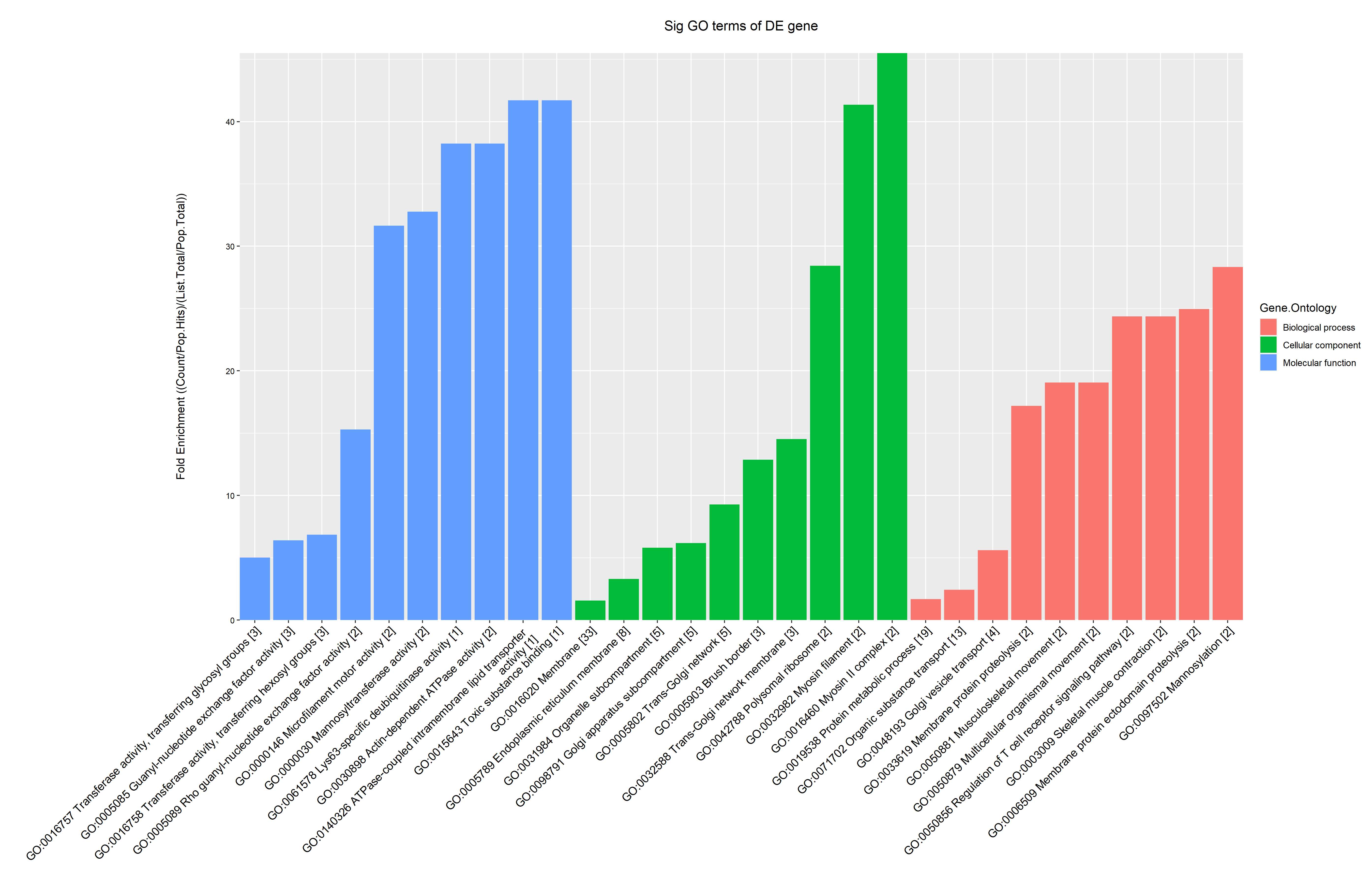

Supplement: Supplementary file 1 [file Data_Sheet_1.ZIP › Additional files/GO Analysis Report/GO_GC_vs_control_up/GeneOntology_FoldEnrichment.png]

# GO Molecular Function Classification

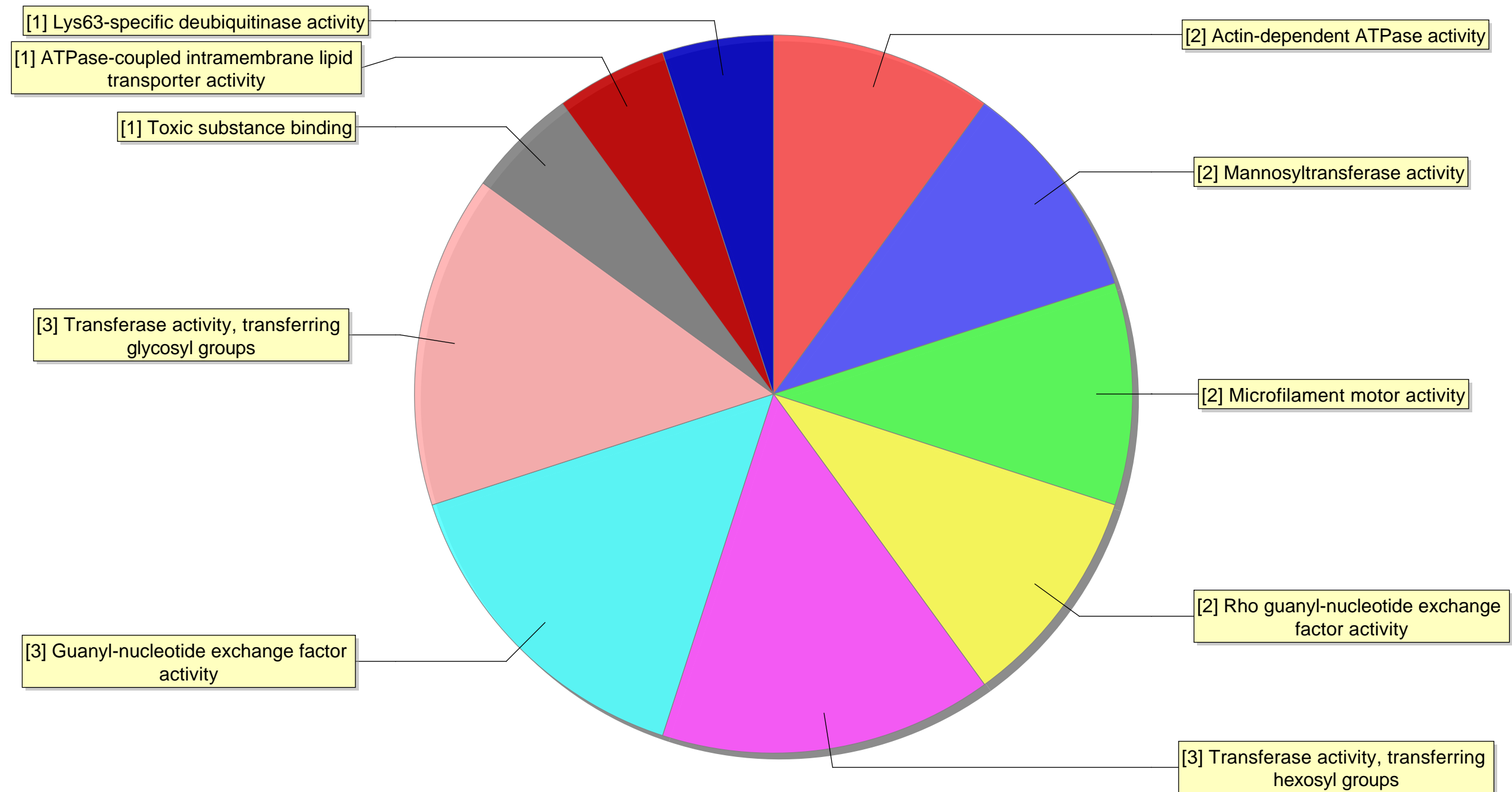

Supplement: Supplementary file 1 [file Data_Sheet_1.ZIP › Additional files/GO Analysis Report/GO_GC_vs_control_up/MF_Count.pdf]

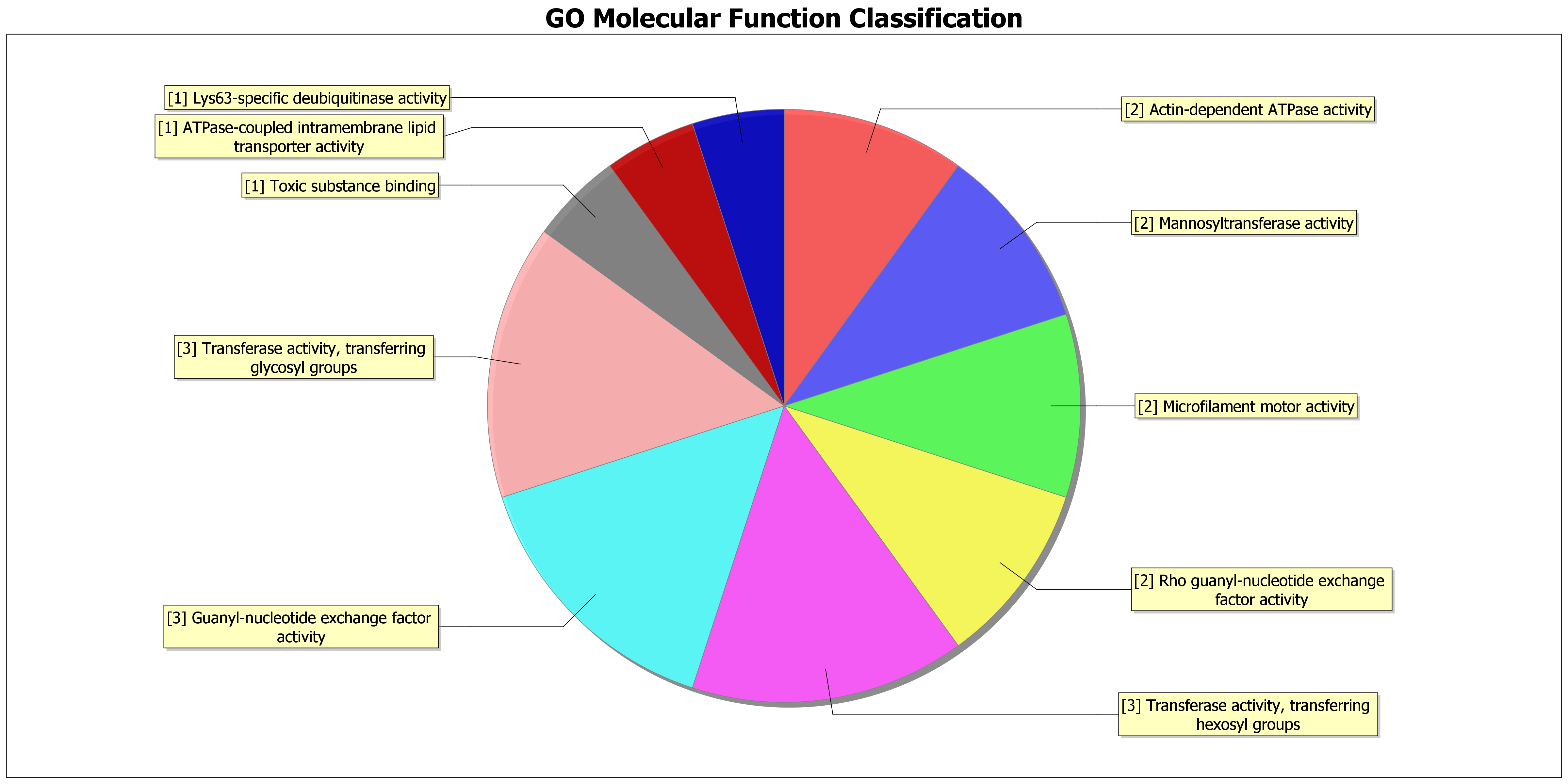

Supplement: Supplementary file 1 [file Data_Sheet_1.ZIP › Additional files/GO Analysis Report/GO_GC_vs_control_up/MF_Count.png]

## Sig GO terms of DE gene-MF

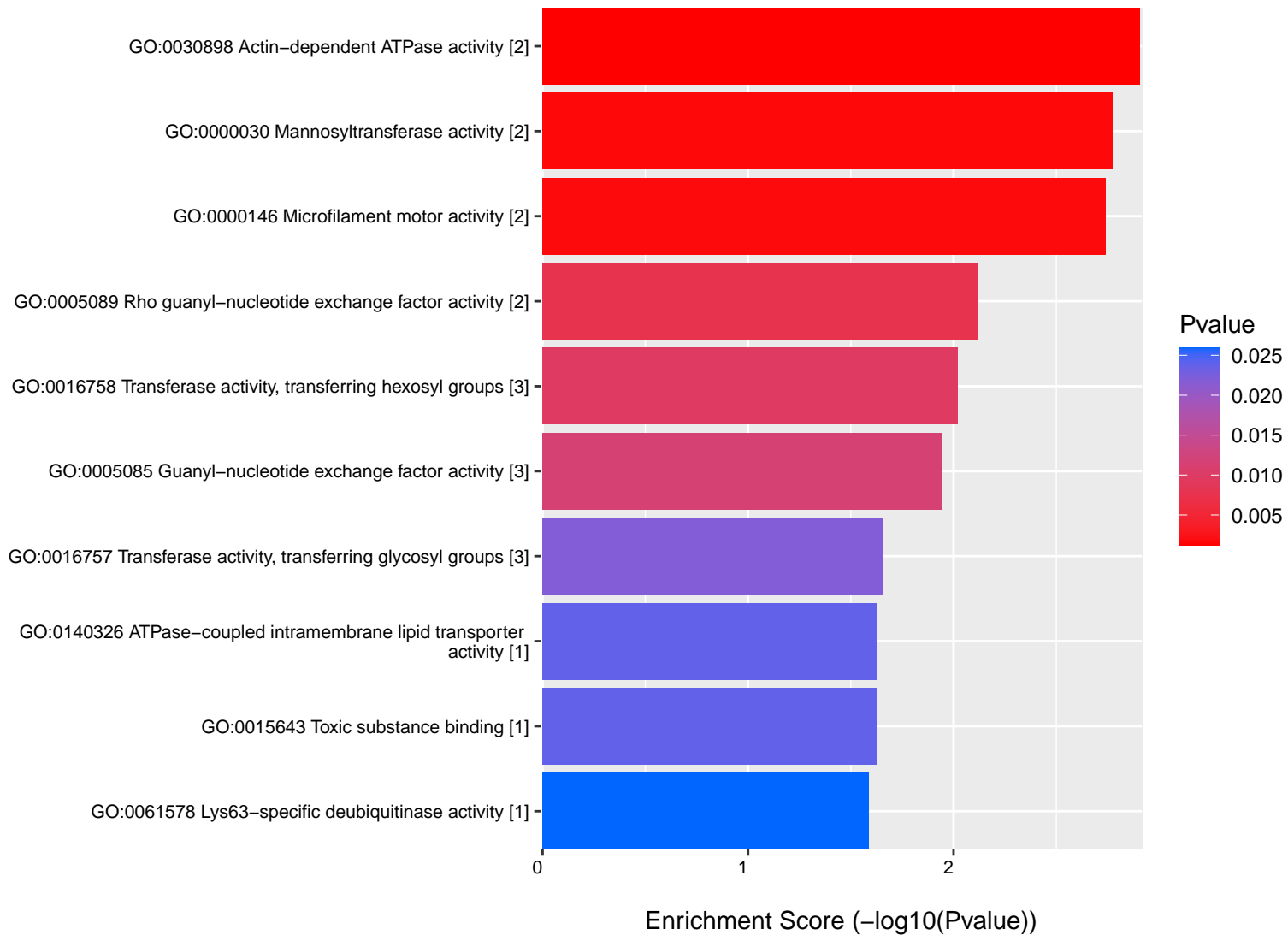

Supplement: Supplementary file 1 [file Data_Sheet_1.ZIP › Additional files/GO Analysis Report/GO_GC_vs_control_up/MF_EnrichmentScore.pdf]

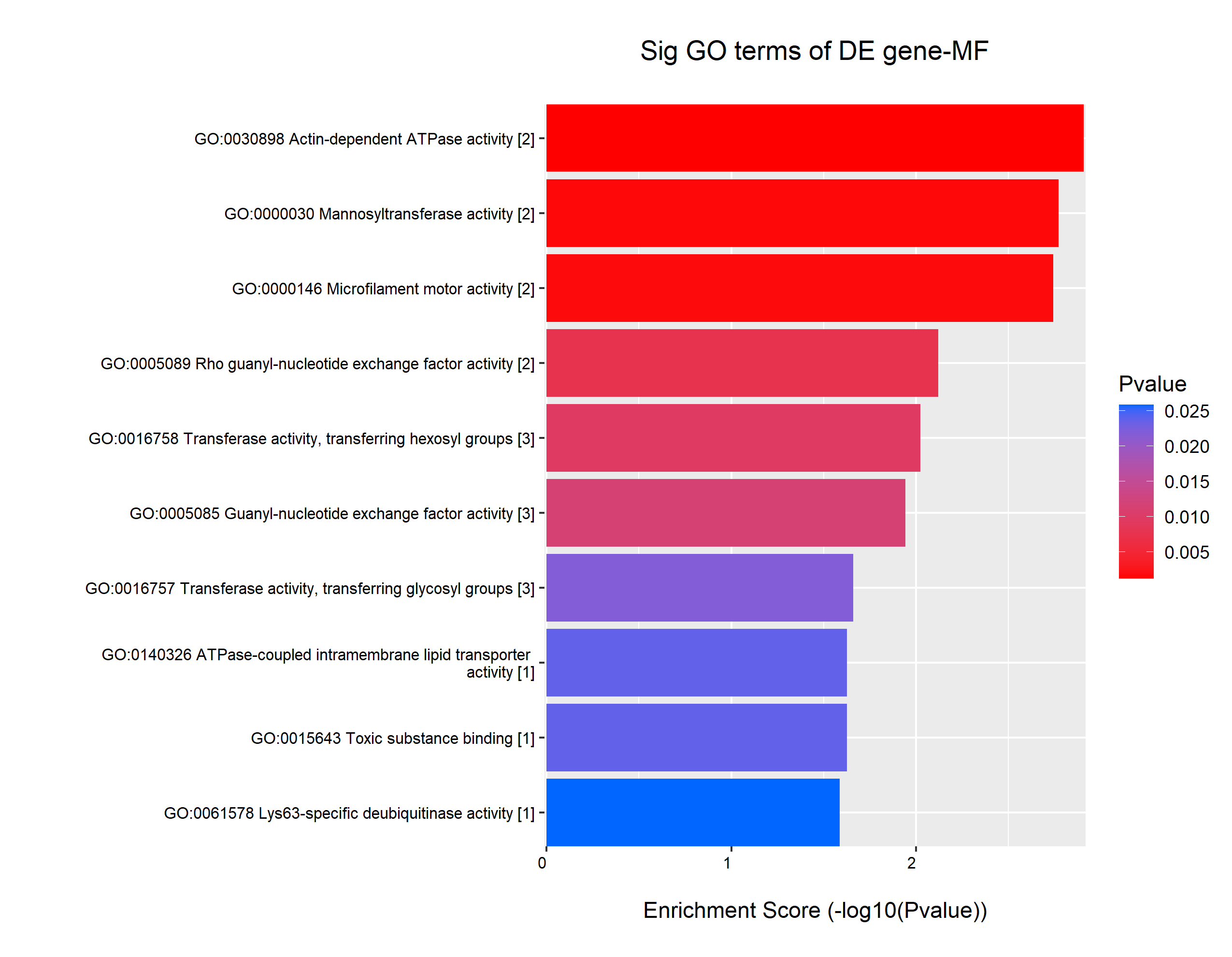

Supplement: Supplementary file 1 [file Data_Sheet_1.ZIP › Additional files/GO Analysis Report/GO_GC_vs_control_up/MF_EnrichmentScore.png]

# Sig GO terms of DE gene-MF

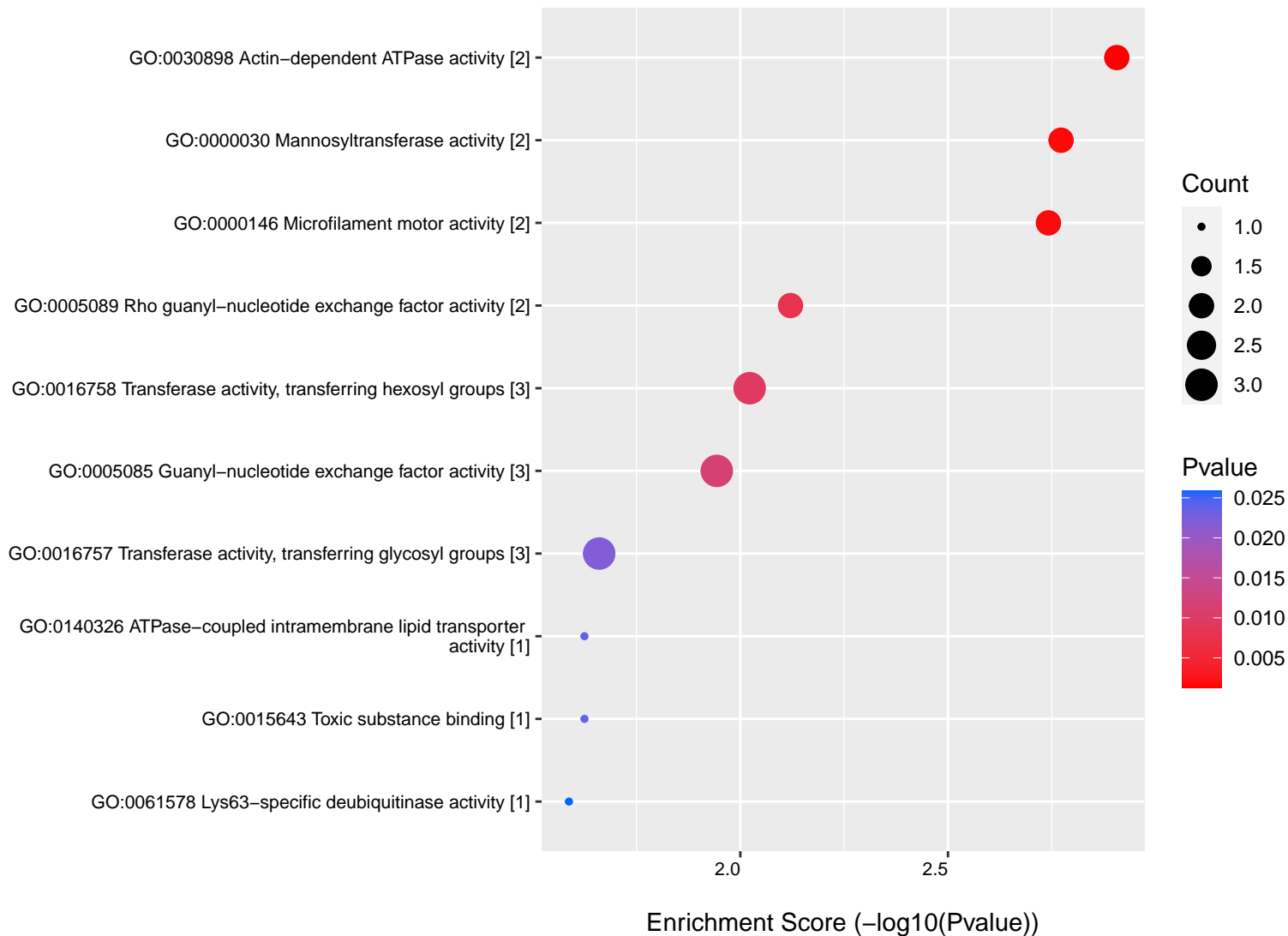

Supplement: Supplementary file 1 [file Data_Sheet_1.ZIP › Additional files/GO Analysis Report/GO_GC_vs_control_up/MF_EnrichmentScoreDotPlot.pdf]

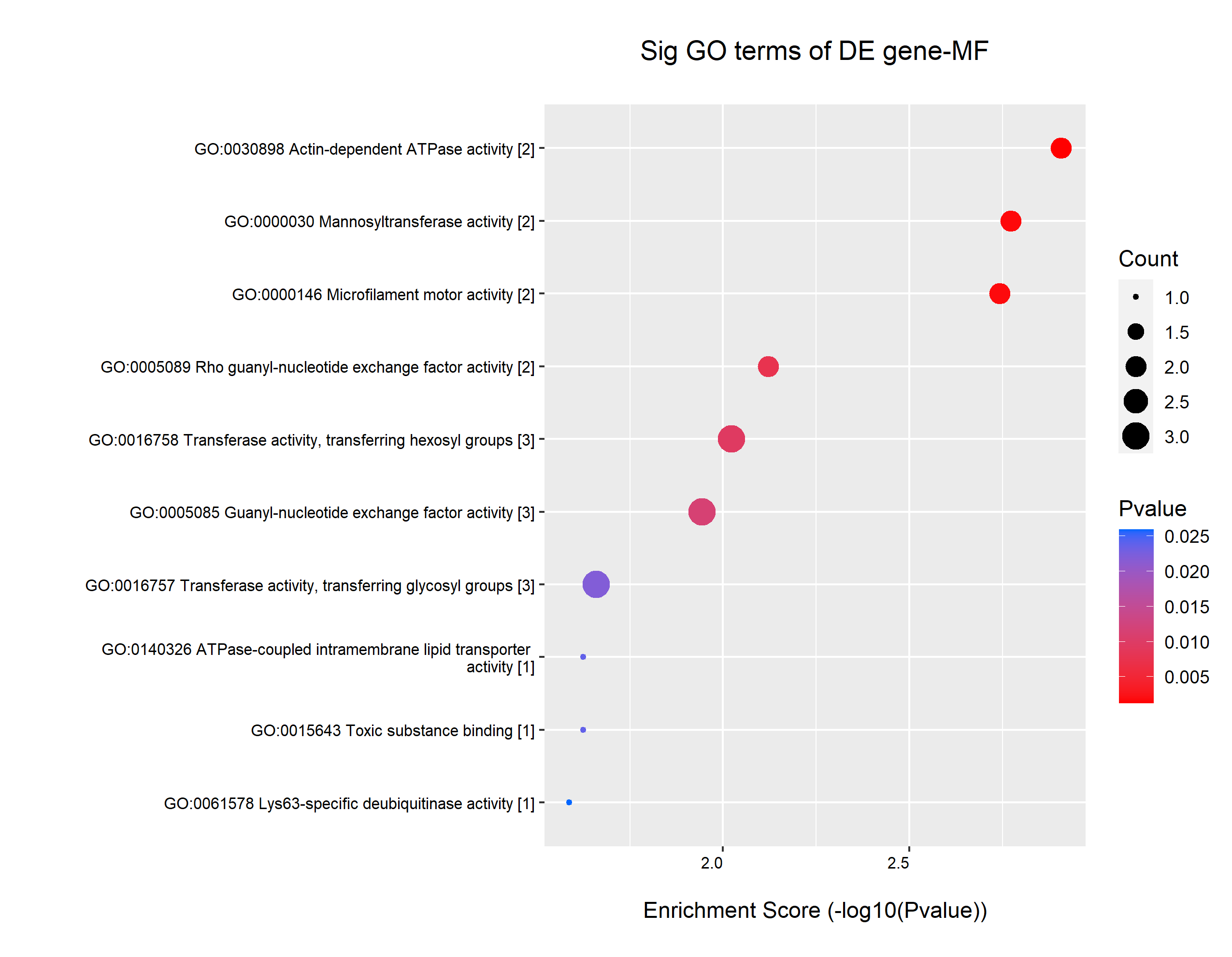

Supplement: Supplementary file 1 [file Data_Sheet_1.ZIP › Additional files/GO Analysis Report/GO_GC_vs_control_up/MF_EnrichmentScoreDotPlot.png]

# Sig GO terms of DE gene–MF

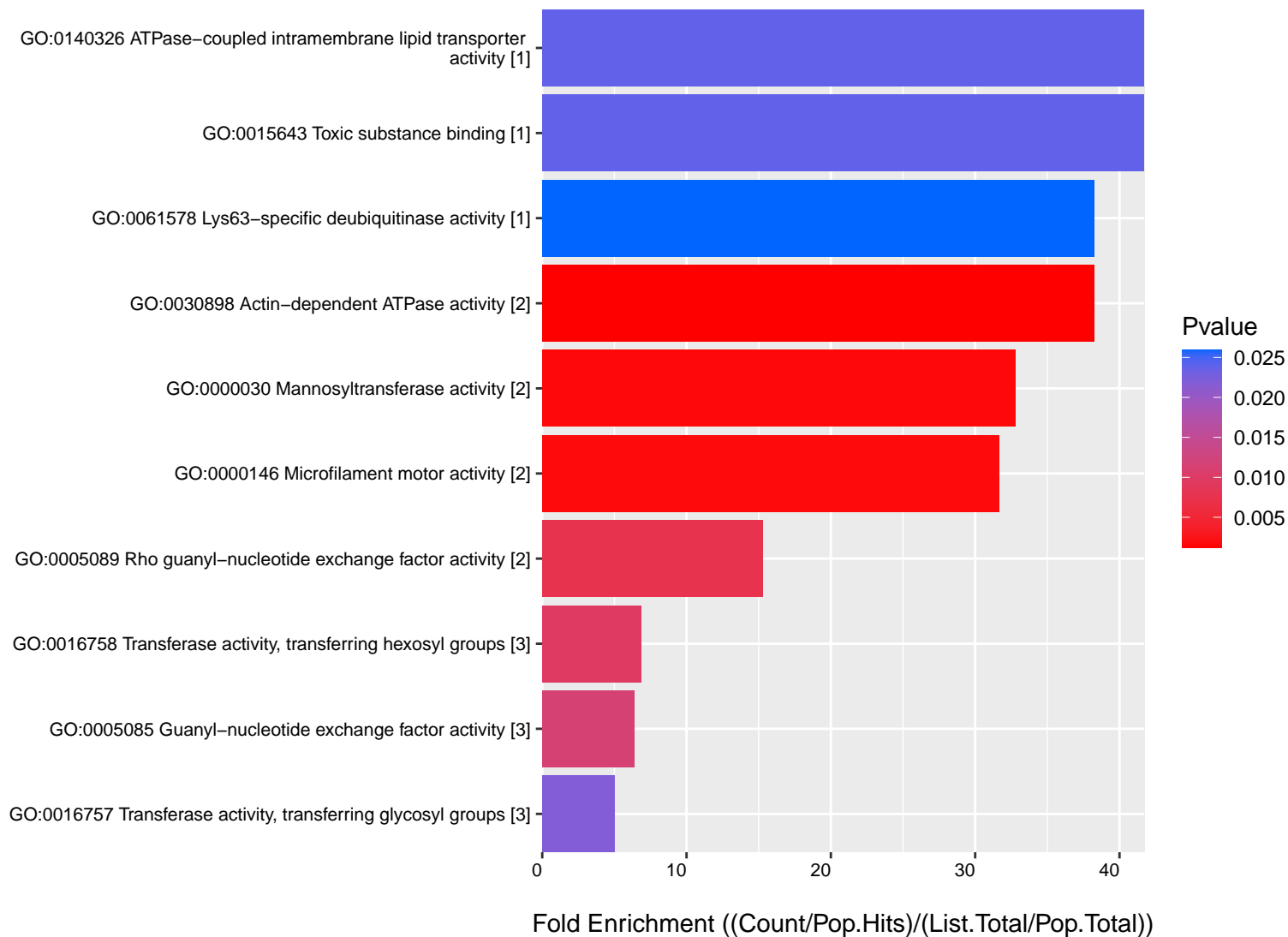

Supplement: Supplementary file 1 [file Data_Sheet_1.ZIP › Additional files/GO Analysis Report/GO_GC_vs_control_up/MF_FoldEnrichment.pdf]

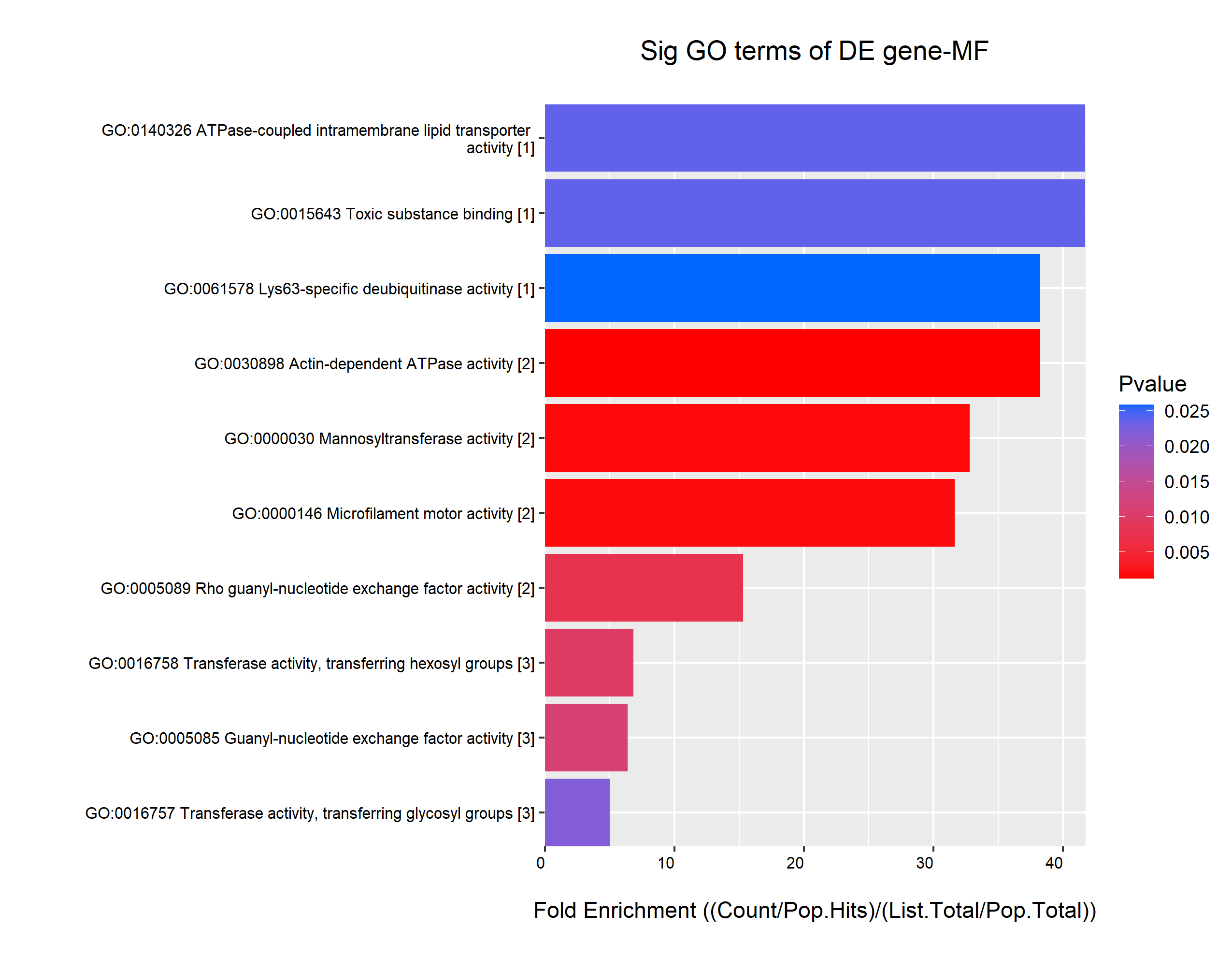

Supplement: Supplementary file 1 [file Data_Sheet_1.ZIP › Additional files/GO Analysis Report/GO_GC_vs_control_up/MF_FoldEnrichment.png]

# Sig GO terms of DE gene–MF

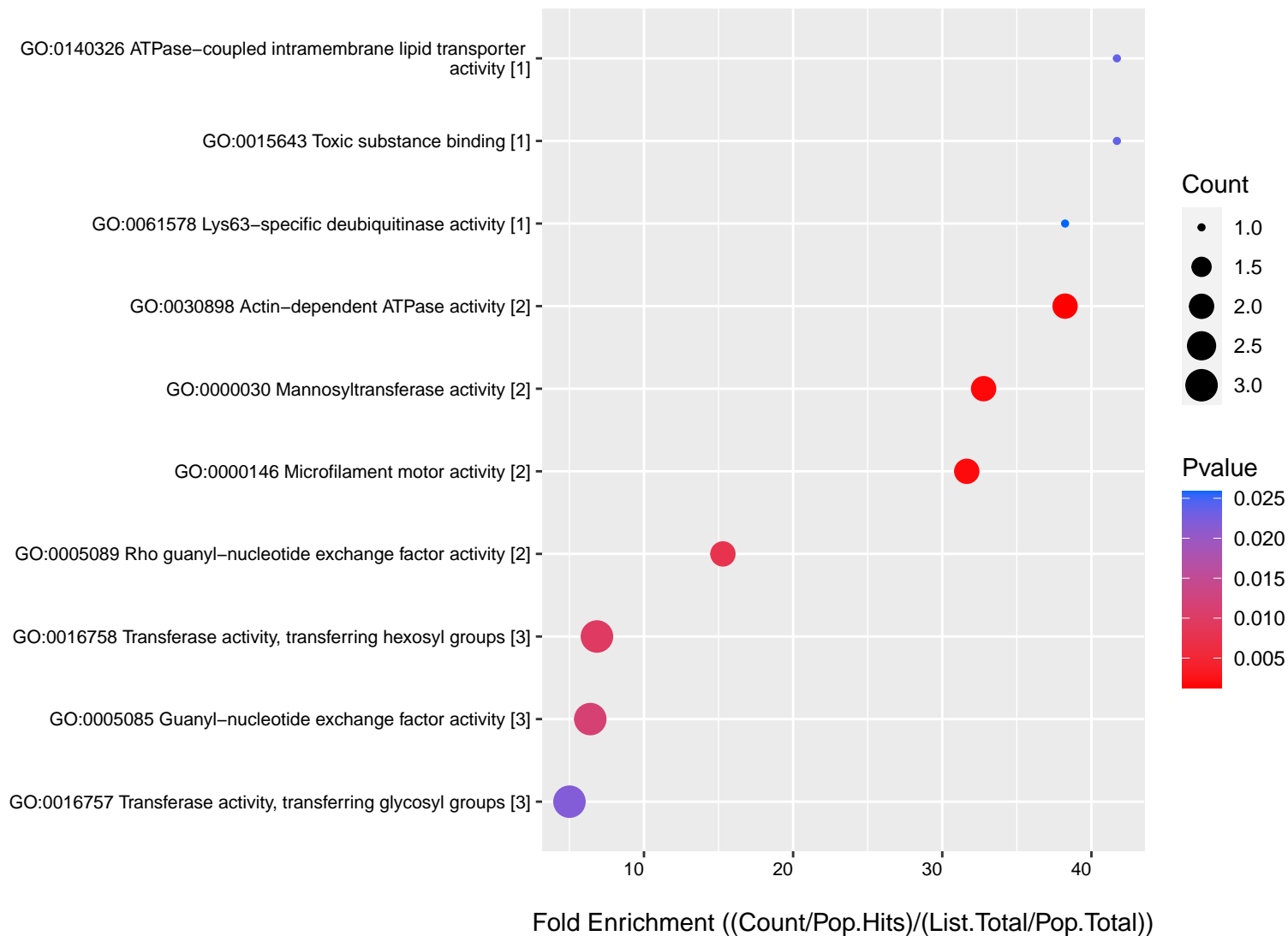

Supplement: Supplementary file 1 [file Data_Sheet_1.ZIP › Additional files/GO Analysis Report/GO_GC_vs_control_up/MF_FoldEnrichmentDotPlot.pdf]

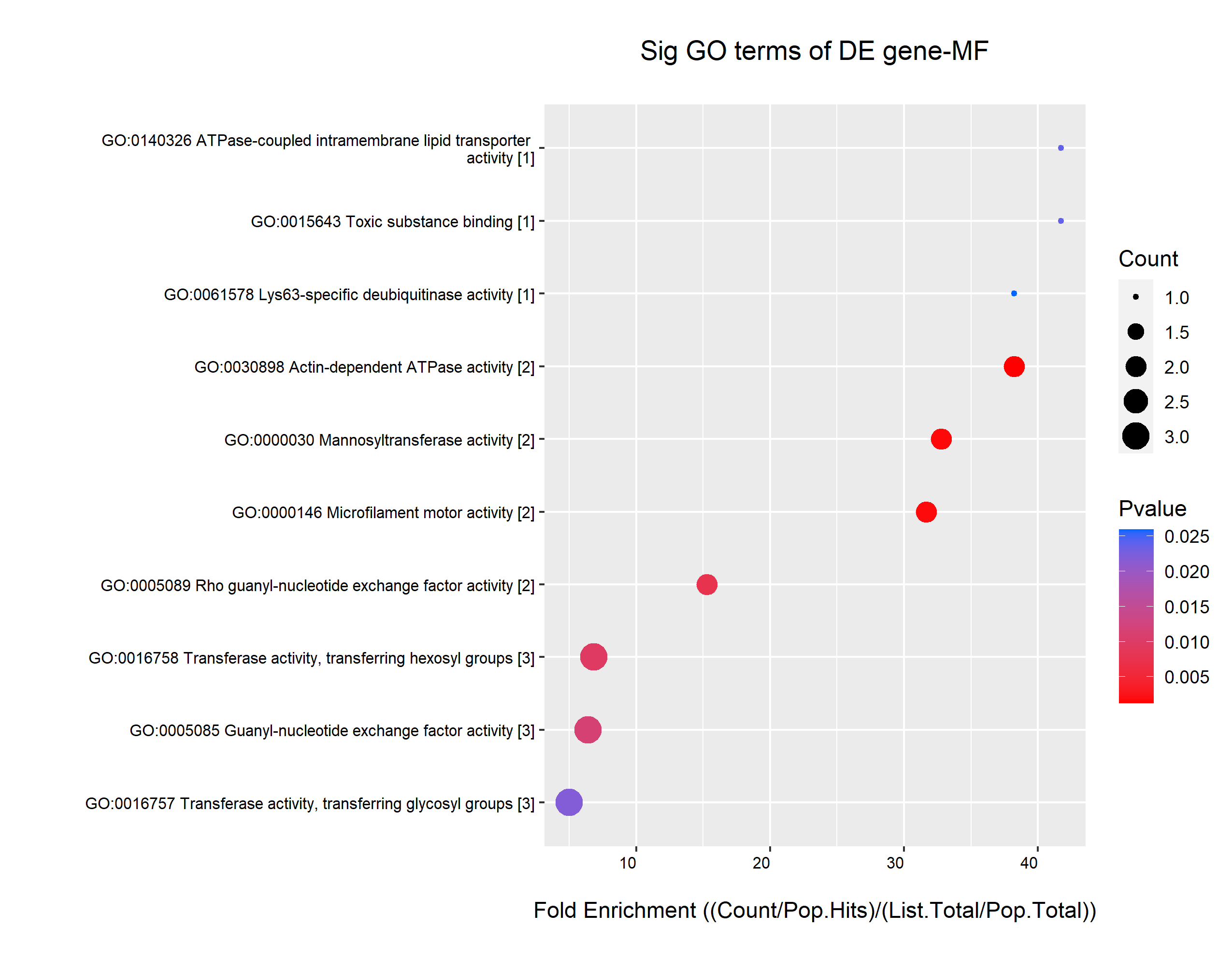

Supplement: Supplementary file 1 [file Data_Sheet_1.ZIP › Additional files/GO Analysis Report/GO_GC_vs_control_up/MF_FoldEnrichmentDotPlot.png]

# Sig GO terms of DE gene-MF

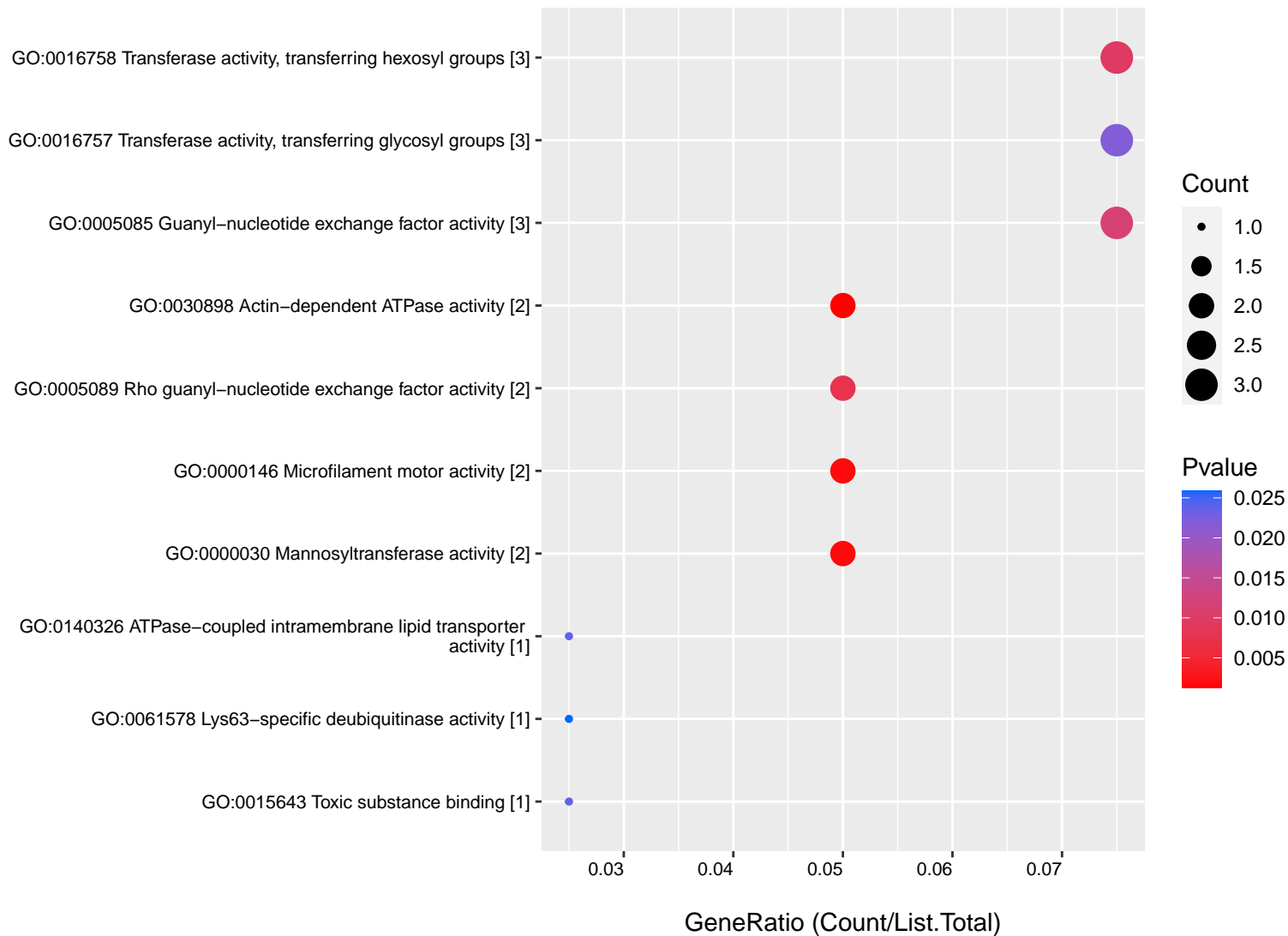

Supplement: Supplementary file 1 [file Data_Sheet_1.ZIP › Additional files/GO Analysis Report/GO_GC_vs_control_up/MF_GeneRatioDotPlot.pdf]

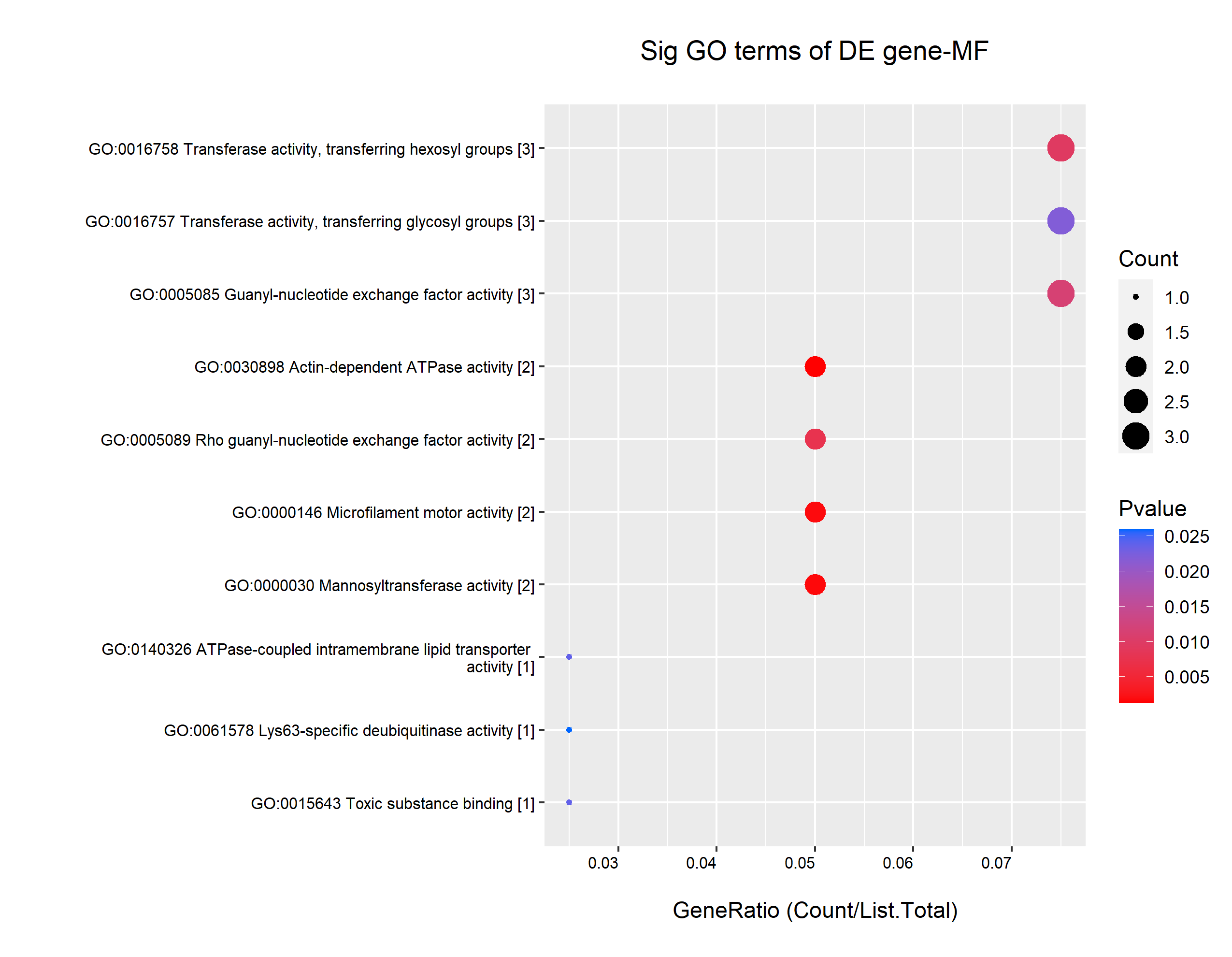

Supplement: Supplementary file 1 [file Data_Sheet_1.ZIP › Additional files/GO Analysis Report/GO_GC_vs_control_up/MF_GeneRatioDotPlot.png]

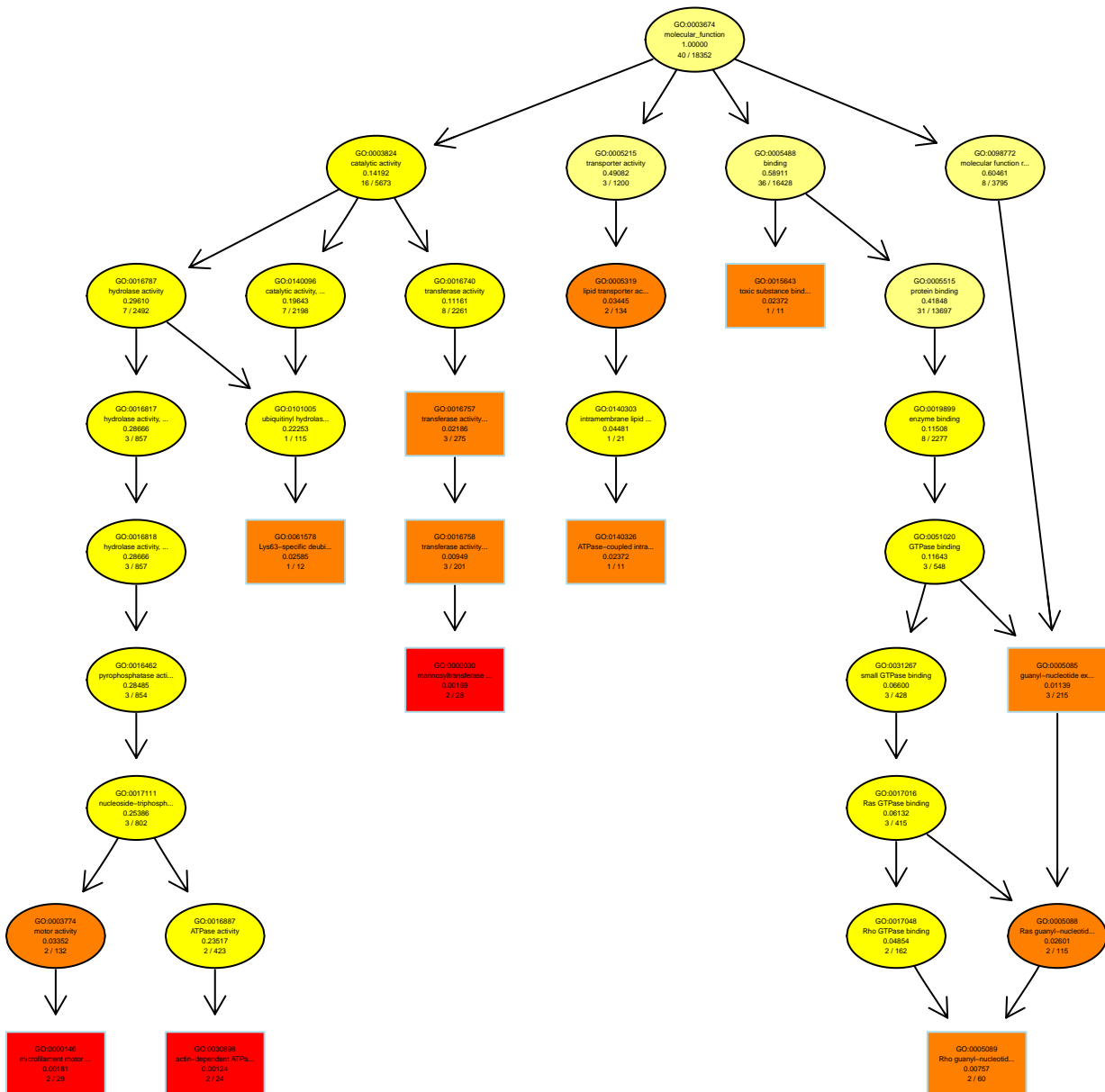

Supplement: Supplementary file 1 [file Data_Sheet_1.ZIP › Additional files/GO Analysis Report/GO_GC_vs_control_up/MF_Pvalue_tree.pdf]

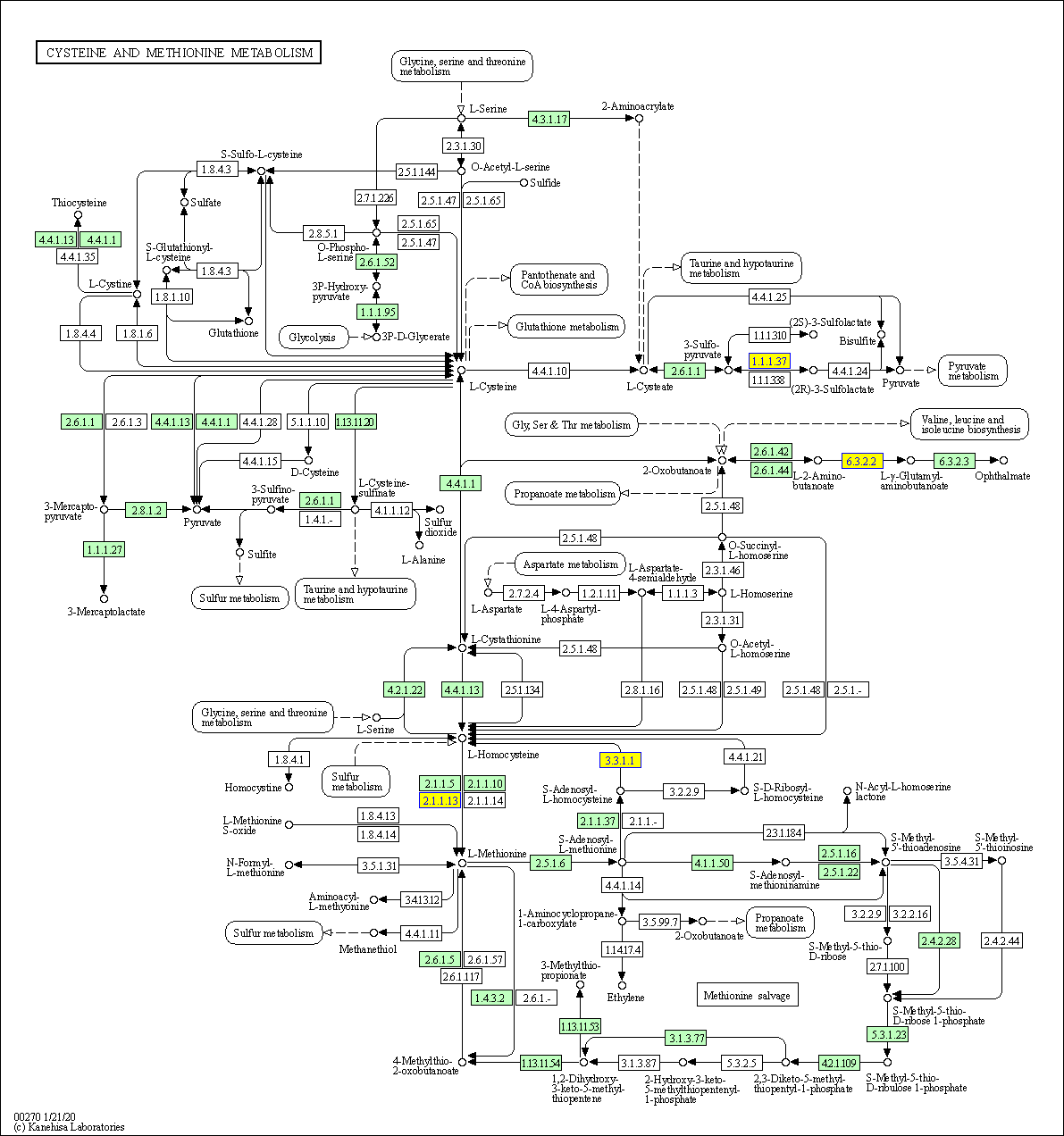

Supplement: Supplementary file 1 [file Data_Sheet_1.ZIP › Additional files/Pathway Analysis Report/Pathway_GC_vs_control_down/hsa00270.png]

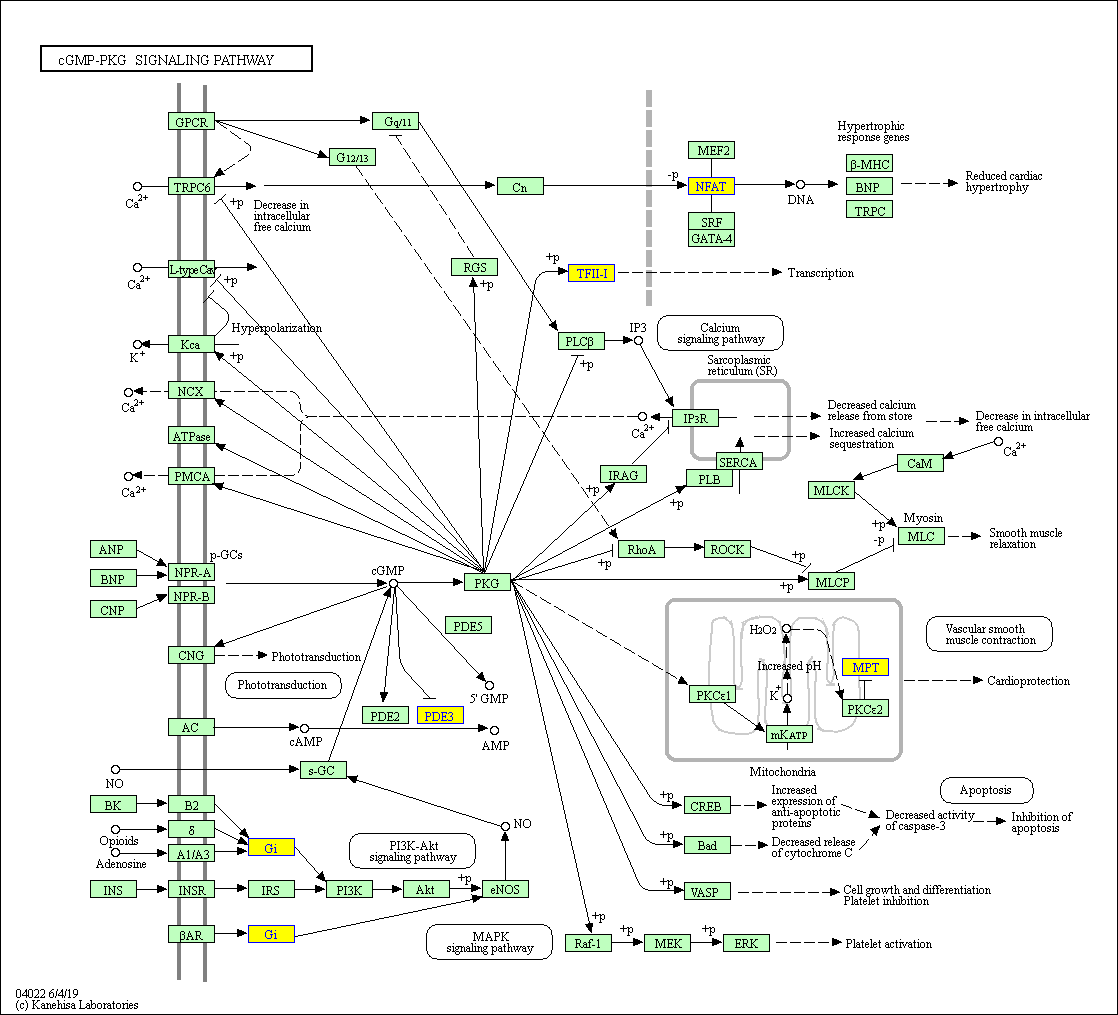

Supplement: Supplementary file 1 [file Data_Sheet_1.ZIP › Additional files/Pathway Analysis Report/Pathway_GC_vs_control_down/hsa04022.png]

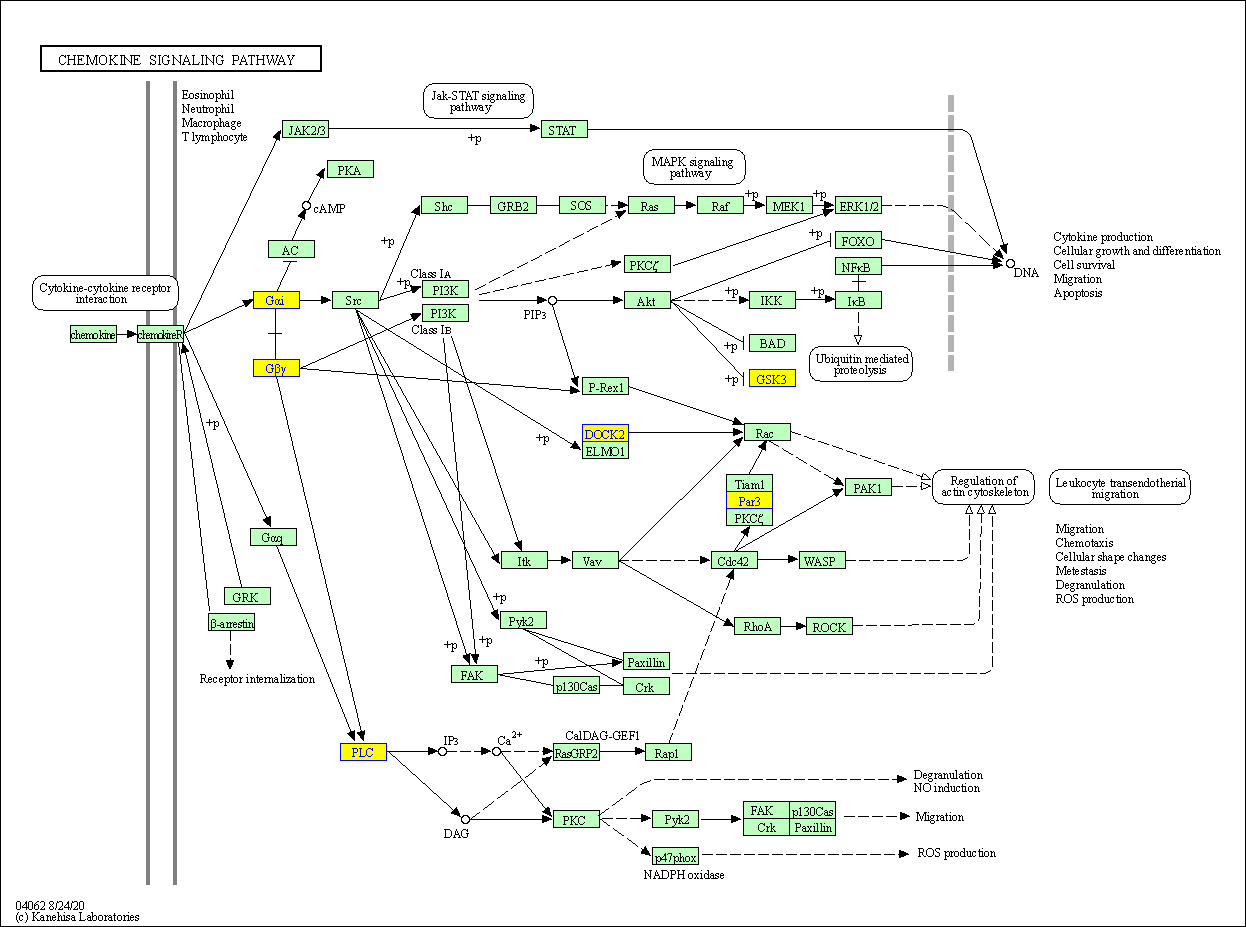

Supplement: Supplementary file 1 [file Data_Sheet_1.ZIP › Additional files/Pathway Analysis Report/Pathway_GC_vs_control_down/hsa04062.png]

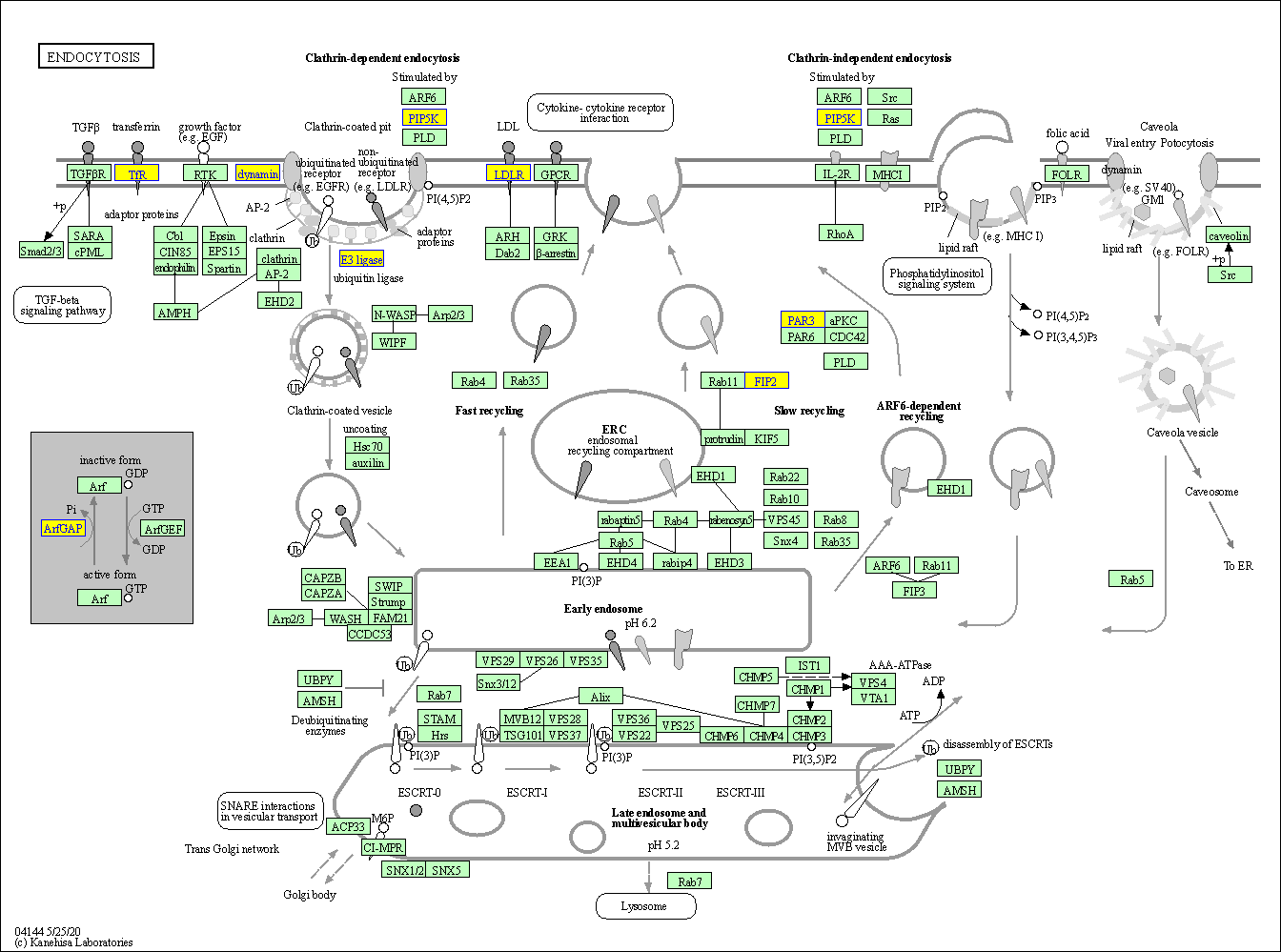

Supplement: Supplementary file 1 [file Data_Sheet_1.ZIP › Additional files/Pathway Analysis Report/Pathway_GC_vs_control_down/hsa04144.png]

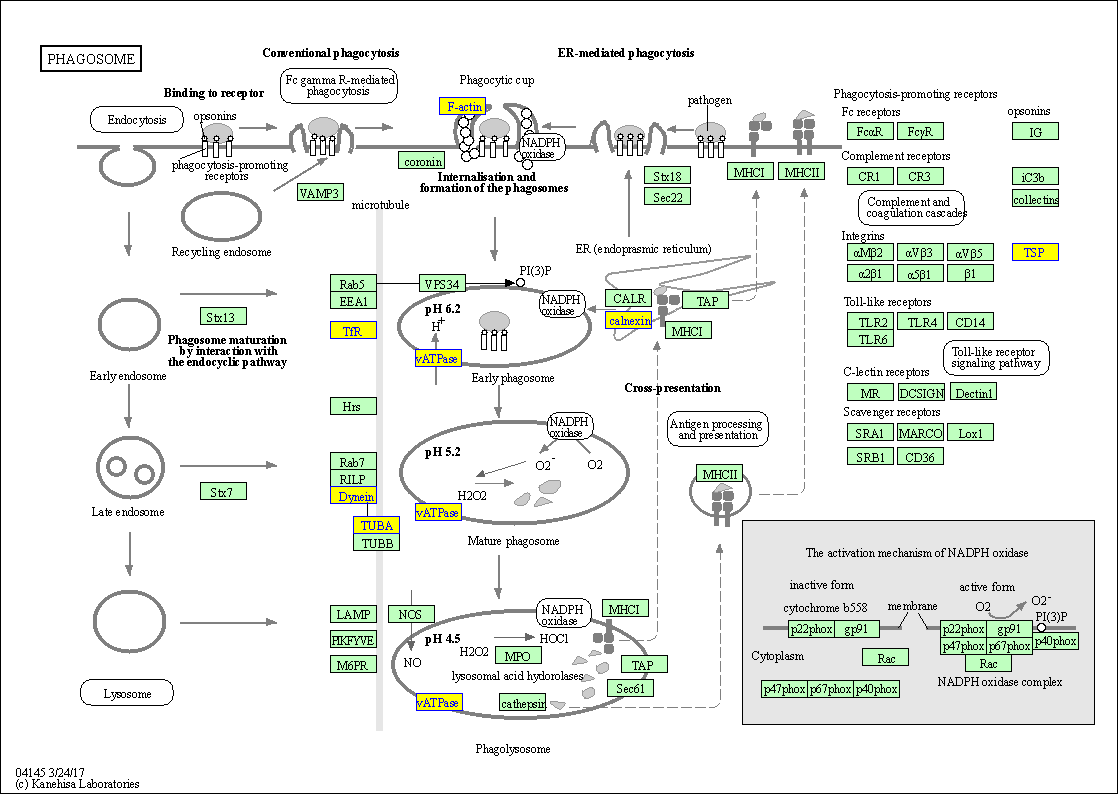

Supplement: Supplementary file 1 [file Data_Sheet_1.ZIP › Additional files/Pathway Analysis Report/Pathway_GC_vs_control_down/hsa04145.png]

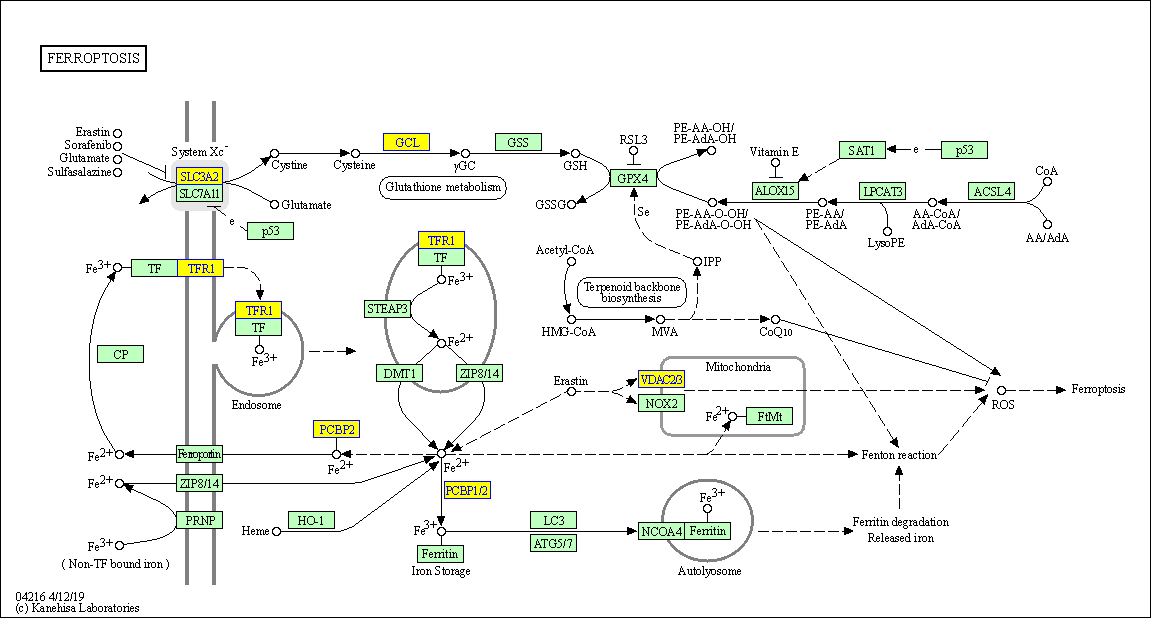

Supplement: Supplementary file 1 [file Data_Sheet_1.ZIP › Additional files/Pathway Analysis Report/Pathway_GC_vs_control_down/hsa04216.png]

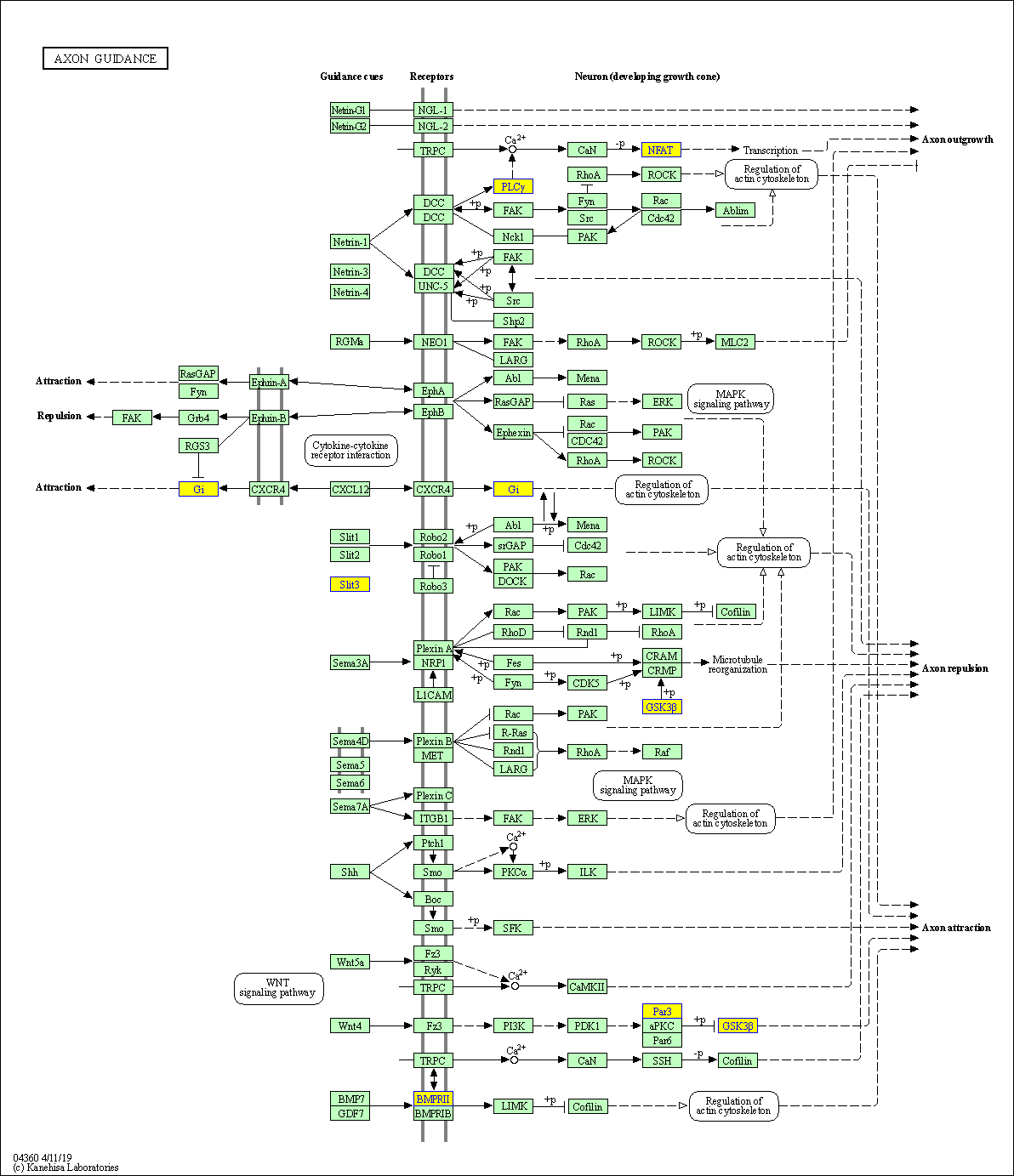

Supplement: Supplementary file 1 [file Data_Sheet_1.ZIP › Additional files/Pathway Analysis Report/Pathway_GC_vs_control_down/hsa04360.png]

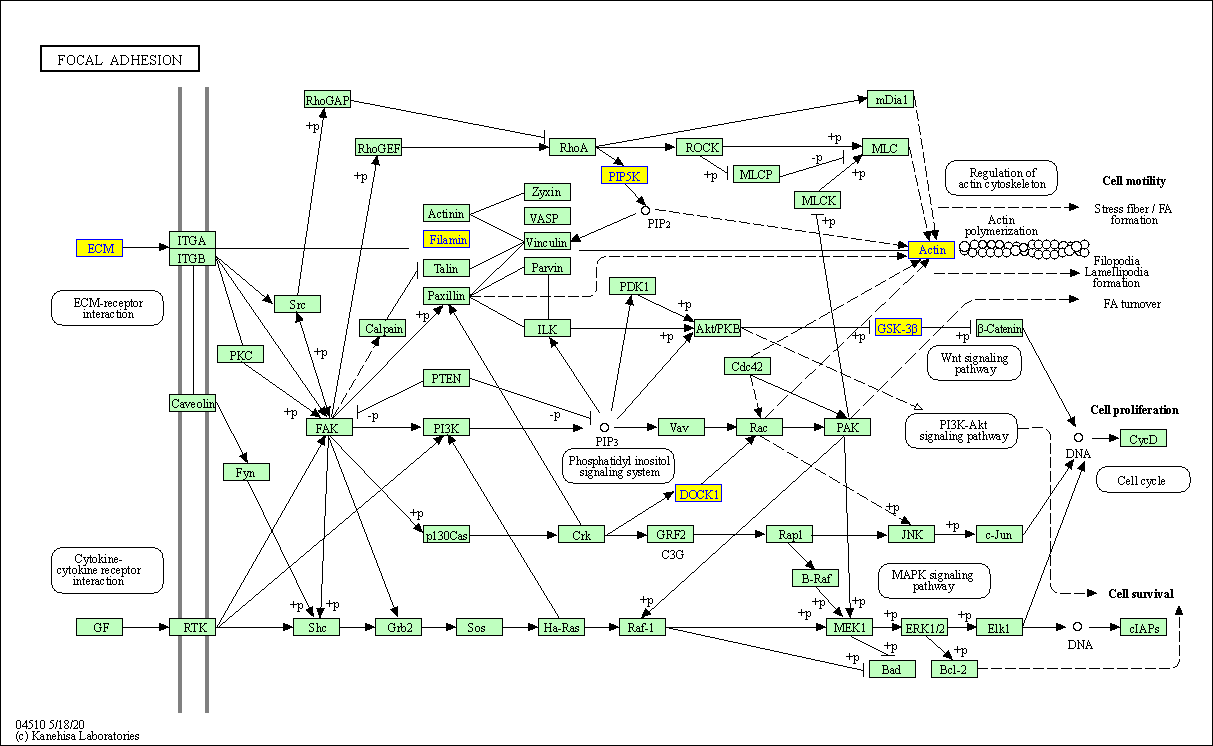

Supplement: Supplementary file 1 [file Data_Sheet_1.ZIP › Additional files/Pathway Analysis Report/Pathway_GC_vs_control_down/hsa04510.png]

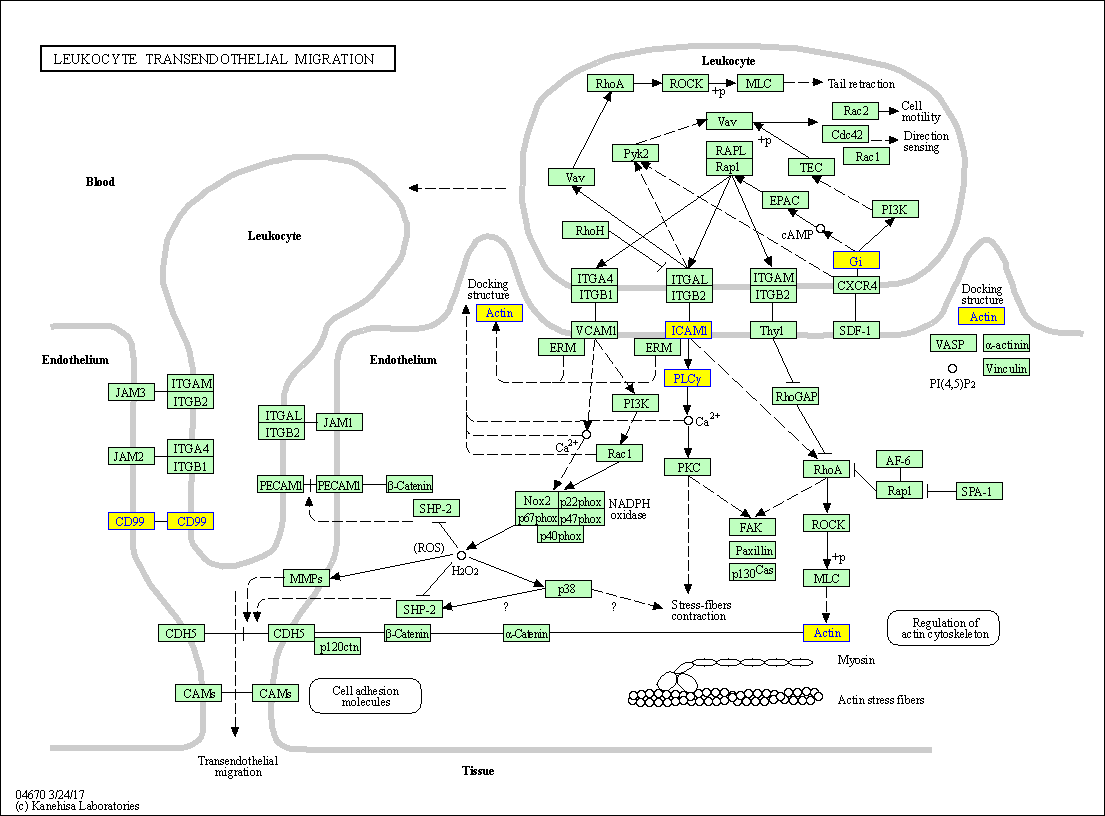

Supplement: Supplementary file 1 [file Data_Sheet_1.ZIP › Additional files/Pathway Analysis Report/Pathway_GC_vs_control_down/hsa04670.png]

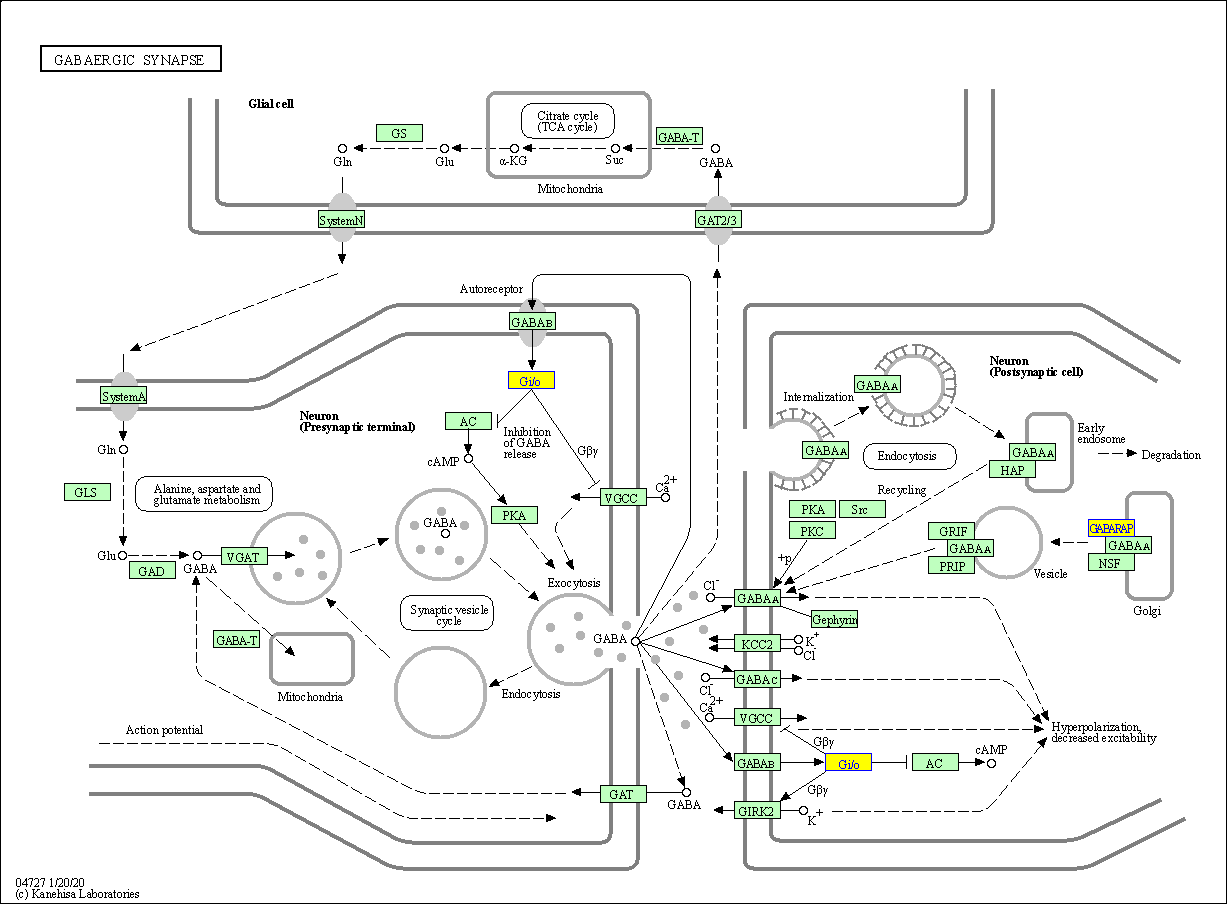

Supplement: Supplementary file 1 [file Data_Sheet_1.ZIP › Additional files/Pathway Analysis Report/Pathway_GC_vs_control_down/hsa04727.png]

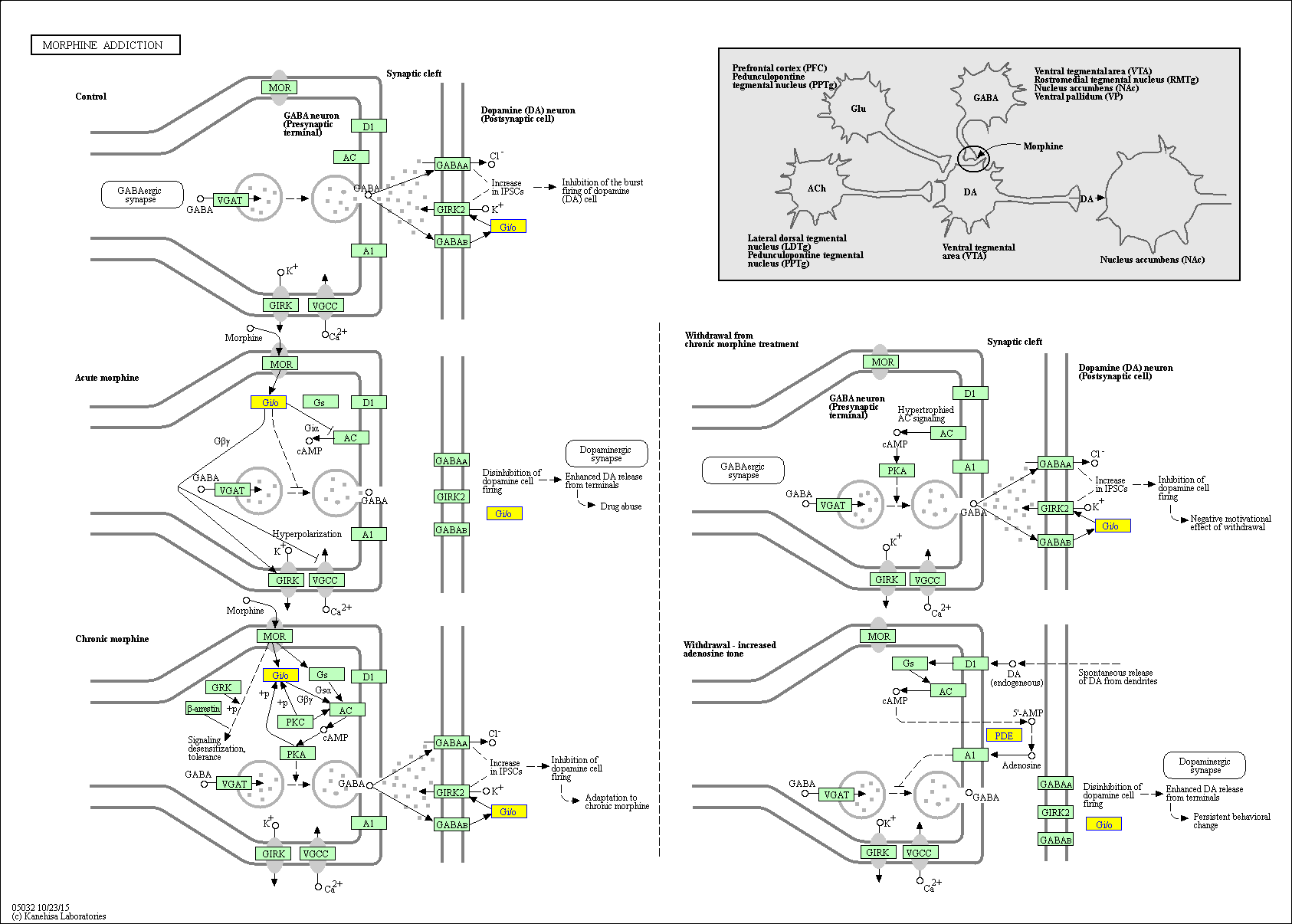

Supplement: Supplementary file 1 [file Data_Sheet_1.ZIP › Additional files/Pathway Analysis Report/Pathway_GC_vs_control_down/hsa05032.png]

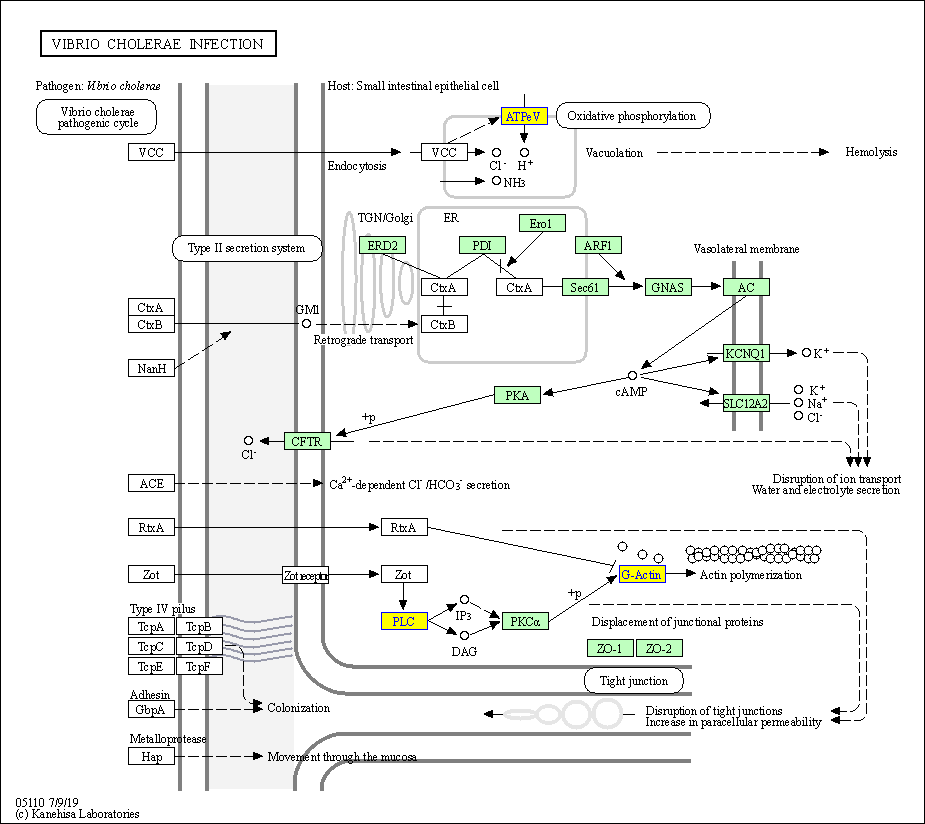

Supplement: Supplementary file 1 [file Data_Sheet_1.ZIP › Additional files/Pathway Analysis Report/Pathway_GC_vs_control_down/hsa05110.png]

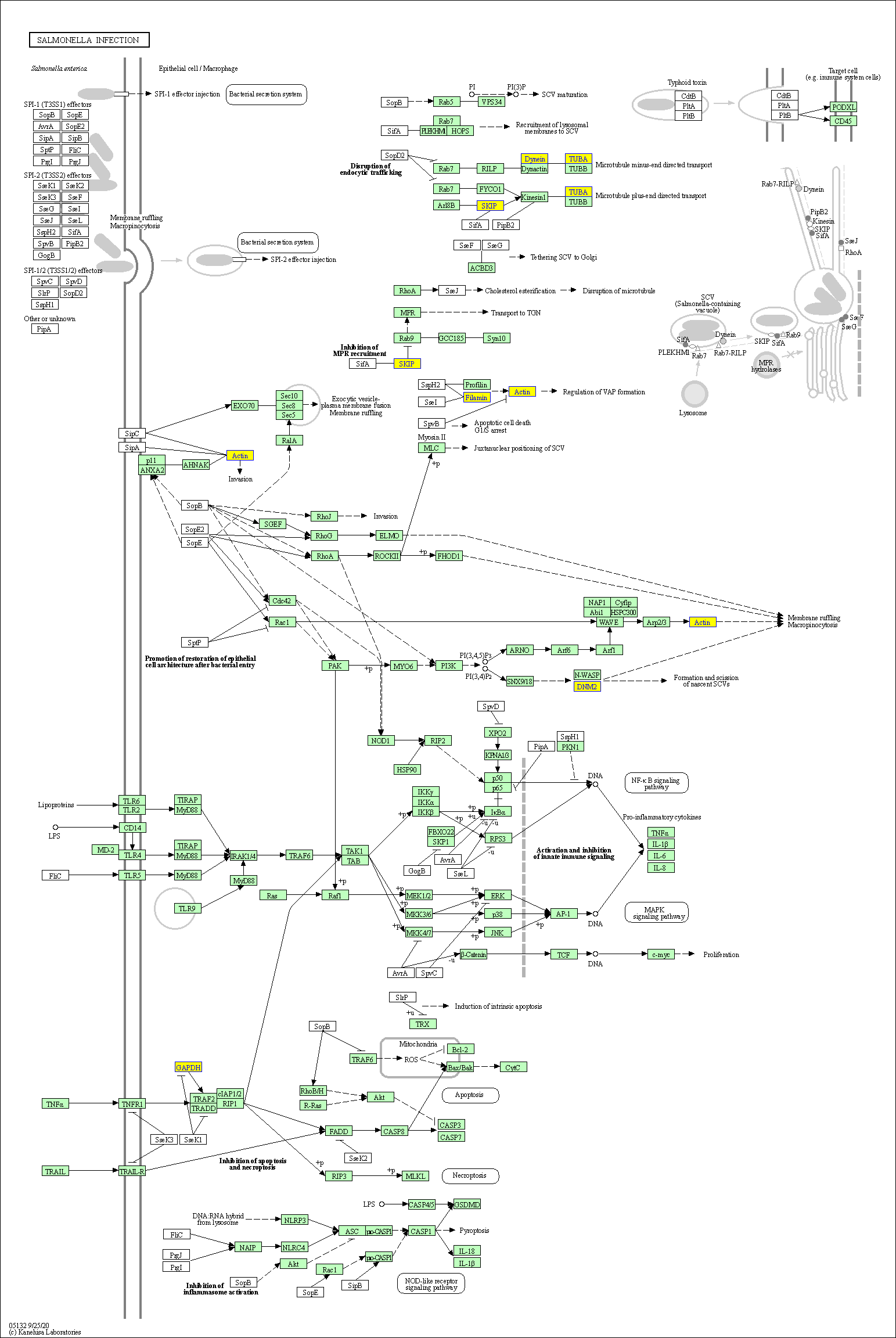

Supplement: Supplementary file 1 [file Data_Sheet_1.ZIP › Additional files/Pathway Analysis Report/Pathway_GC_vs_control_down/hsa05132.png]

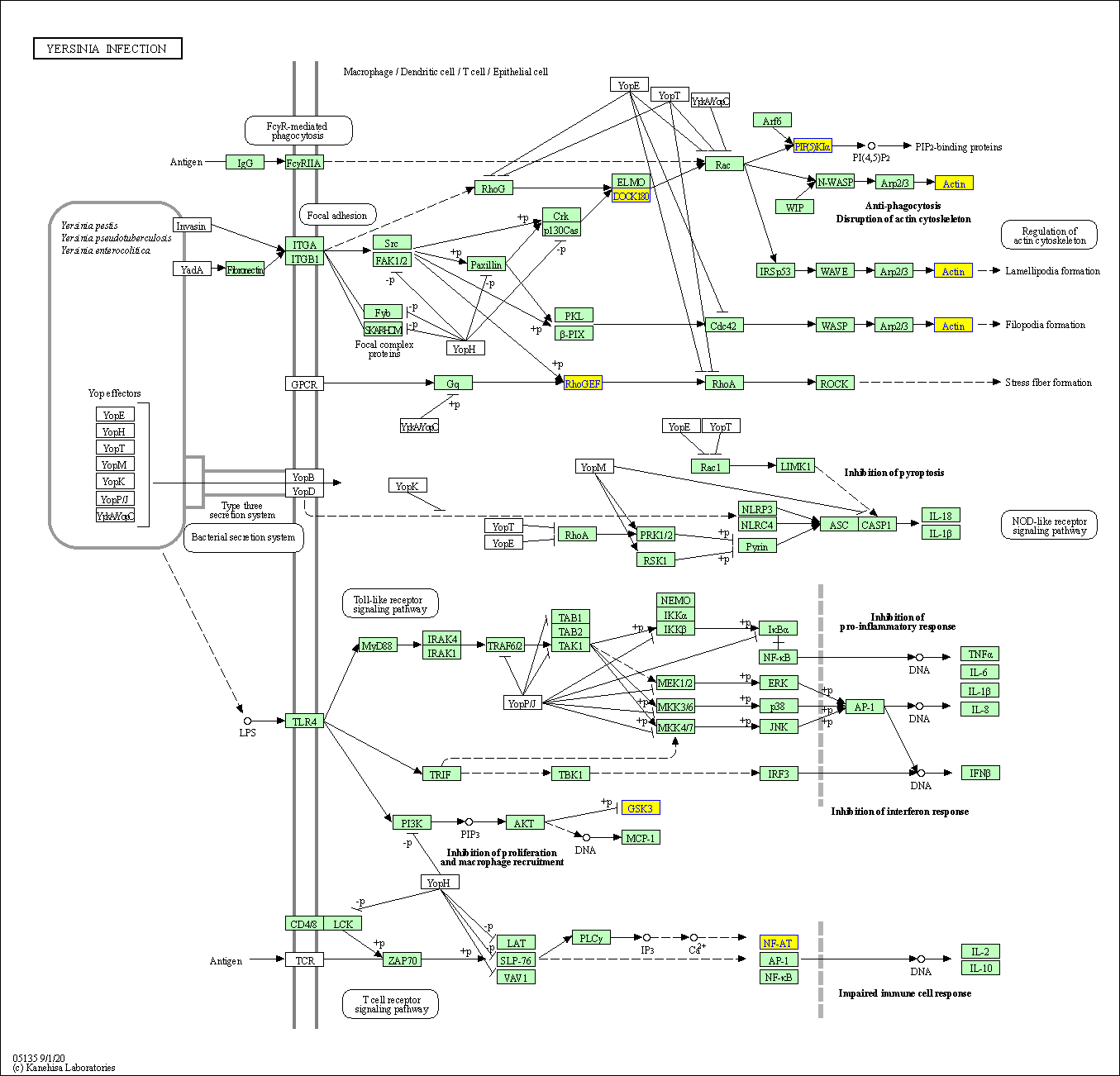

Supplement: Supplementary file 1 [file Data_Sheet_1.ZIP › Additional files/Pathway Analysis Report/Pathway_GC_vs_control_down/hsa05135.png]
