# Supplementary material for: Differentially Expressed Circular RNAs and Their Therapeutic Mechanism in Non-segmental Vitiligo Patients Treated With Methylprednisolone
Source: Front Med (Lausanne). 2022 May 16;9:839066. doi: 10.3389/fmed.2022.839066 (PMC9149005; doi:10.3389/fmed.2022.839066)
Supplement: Supplementary file 1 [file Data_Sheet_1.ZIP › Additional files/Pathway Analysis Report/summary_PATHWAY.doc]

**Pathway analysis**

Pathway analysis is a functional analysis mapping genes to KEGG pathways. The p-value (EASE-score, Fisher-Pvalue or Hypergeometric-Pvalue) denotes the significance of the Pathway correlated to the conditions. Lower the p-value, more significant is the Pathway. (The recommend p-value cut-off is 0.05.)

- **Items explanation:**
- **PathwayID:** Pathway identifiers used in [KEGG](http://www.genome.jp/kegg/).
- **Definition:** The definition of the PathwayID.
- **OriginalWebSite:** The map's website, the nodes with red frame and red symbol are associated with the DE genes, green nodes have no significance. Certain materials may be copyrighted by the holders.
- **p-value:**
  - **EASE-score:** The enrichment p-value of the PathwayID used [EASE method](http://www.ncbi.nlm.nih.gov/pubmed/14519205), EASE Score is modified Fisher’s exact probability by penalizing (subtract) the count of positive agreement by 1.
  - **Fisher-Pvalue:** The enrichment p-value of the PathwayID used Fisher’s exact test.
  - **Hypergeometric-Pvalue:** The enrichment p-value of the PathwayID used Hypergeometric test.
- **SelectionCounts:** Count of the DE genes’ entities directly associated with the listed PathwayID.
- **SelectionSize:** Total number of the DE genes’ entities.
- **Count:** Count of the chosen background population genes’ entities associated with the listed PathwayID
- **Size:** Total number of chosen background population genes’ entities
- **FDR:** the false discovery rate of the PathwayID.
- **Enrichment_Score:** the Enrichment Score value of the PathwayID, it equals "-log10(Pvalue)”
- **GeneRatio:** the Gene Ratio value of the PathwayID, it equals "SelectionCounts / SelectionSize”
- **GENES:** The genes which are from the dataset associated with the PathwayID.
- **Pathway barplot explanation (EnrichmentScore):**

The bar plot shows the top ten Enrichment score (-log10(Pvalue)) value of the significant enrichment pathway.

- **Pathway dotplot explanation (EnrichmentScoreDotPlot):**

The dot plot shows the top ten Enrichment score (-log10(Pvalue)) value of the significant enrichment pathway.

- **Pathway dotplot explanation (GeneRatioDotPlot):**

The dot plot shows the gene ratio value of the top ten most significant enrichment pathways.

- **Pathway map explanation:**

Yellow marked nodes are associated with down-regulated genes, orange marked nodes are associated with up-regulated or only whole dataset genes, green nodes have no significance.
